# Differential Distribution of Type II CRISPR-Cas Systems in Agricultural and Nonagricultural *Campylobacter coli* and *Campylobacter jejuni* Isolates Correlates with Lack of Shared Environments

**DOI:** 10.1093/gbe/evv174

**Published:** 2015-09-02

**Authors:** Bruce M. Pearson, Rogier Louwen, Peter van Baarlen, Arnoud H.M. van Vliet

**Affiliations:** ^1^Institute of Food Research, Gut Health and Food Safety Programme, Norwich Research Park, Norwich, United Kingdom; ^2^Department of Medical Microbiology and Infectious Diseases, Erasmus MC-University Medical Center Rotterdam, Rotterdam, The Netherlands; ^3^Host–Microbe Interactomics Group, Department of Animal Sciences, Wageningen University, Wageningen, The Netherlands

**Keywords:** CRISPR, horizontal gene transfer, comparative genomics, *Campylobacter*, mobile DNA, phage defense

## Abstract

CRISPR (clustered regularly interspaced palindromic repeats)-Cas (CRISPR-associated) systems are sequence-specific adaptive defenses against phages and plasmids which are widespread in prokaryotes. Here we have studied whether phylogenetic relatedness or sharing of environmental niches affects the distribution and dissemination of Type II CRISPR-Cas systems, first in 132 bacterial genomes from 15 phylogenetic classes, ranging from Proteobacteria to Actinobacteria. There was clustering of distinct Type II CRISPR-Cas systems in phylogenetically distinct genera with varying G+C%, which share environmental niches. The distribution of CRISPR-Cas within a genus was studied using a large collection of genome sequences of the closely related *Campylobacter* species *Campylobacter jejuni* (*N* = 3,746) and *Campylobacter coli* (*N* = 486). The Cas gene *cas9* and CRISPR-repeat are almost universally present in *C. jejuni* genomes (98.0% positive) but relatively rare in *C. coli* genomes (9.6% positive). *Campylobacter jejuni* and agricultural *C. coli* isolates share the *C. jejuni* CRISPR-Cas system, which is closely related to, but distinct from the *C. coli* CRISPR-Cas system found in *C. coli* isolates from nonagricultural sources. Analysis of the genomic position of CRISPR-Cas insertion suggests that the *C. jejuni*-type CRISPR-Cas has been transferred to agricultural *C. coli*. Conversely, the absence of the *C. coli*-type CRISPR-Cas in agricultural *C. coli* isolates may be due to these isolates not sharing the same environmental niche, and may be affected by farm hygiene and biosecurity practices in the agricultural sector. Finally, many CRISPR spacer alleles were linked with specific multilocus sequence types, suggesting that these can assist molecular epidemiology applications for *C. jejuni* and *C. coli*.

## Introduction

Horizontal gene transfer and DNA acquisition play an important role in evolution of the prokaryotic genome, and these processes are often mediated by phages, plasmids or other forms of mobile DNA and RNA (Penades et al. 2014). As many traits may not be beneficial or advantageous to the recipient, prokaryotes use defense systems to protect the integrity of their genomes, such as restriction-modification systems, abortive infection systems, and the CRISPR (clustered regularly interspaced palindromic repeats)-Cas (CRISPR-associated) system (Labrie et al. 2010). The CRISPR repeats and CRISPR-associated (Cas) genes (Jansen et al. 2002) were originally used for typing purposes, but have since then been shown to constitute an RNA-guided defense system which targets incoming mobile nucleic acids (Barrangou et al. 2007; Brouns et al. 2008; Marraffini and Sontheimer 2008). CRISPR arrays consist of a regularly interspaced array of repeats, with the spacers lacking sequence conservation, and arrays can differ massively in number of repeats. Although very variable in the actual components, CRISPR-mediated immunity works in general in three steps: 1) Acquisition of new spacers into the array, 2) expression and processing of CRISPR RNA (crRNA), and 3) sequence-specific interference (Makarova, Haft, et al. 2011; Wiedenheft et al. 2012).

With the rapid increase in the availability of genome sequences, it is now clear that CRISPR-Cas systems are widespread in both bacteria and archaea (Kunin et al. 2007; Horvath et al. 2008; Makarova, Haft, et al. 2011; Wiedenheft et al. 2012; Barrangou and Marraffini 2014). Bacteria which have one or more CRISPR-Cas systems still show high levels of variation in terms of CRISPR repeat numbers and spacer sequences between isolates, and there can be a significant proportion of isolates which do not have a CRISPR-Cas system at all (Louwen et al. 2013). The CRISPR-Cas systems have recently been classified into three major lineages (Type I, II, and III), each of which is further divided into sublineages depending on the number and composition of the Cas genes (Makarova, Aravind, et al. 2011; Makarova, Haft, et al. 2011). Two of the Cas genes (*cas1* and *cas2*) are virtually ubiquitous in CRISPR-Cas systems and are thought to function in acquisition and integration of new protospacers (Yosef et al. 2012), with the difference between the types being in the genes encoding the proteins involved in processing of the crRNAs and the guiding, targeting and interference of the incoming nucleic acids (Makarova, Haft, et al. 2011; Wiedenheft et al. 2012; Charpentier et al. 2015). The Type II CRISPR-Cas systems show the lowest level of diversity in components, as the main component is a large protein called Cas9 (also known as Csn1 and Csx12), which mediates both the crRNA processing and the interference stages, together with the separately transcribed tracrRNA, and participates in spacer acquisition (Heler et al. 2015). The canonical Type II system also encodes Cas1 and Cas2 proteins, and the II-A subgroup contains an additional *csn2* or *csn2*-like gene, the II-B subgroup contains an additional *cas4* gene, and the II-C subgroup does not contain any additional genes (Makarova, Haft, et al. 2011; Chylinski et al. 2013; Koonin and Makarova 2013). The II-A and II-C systems are more closely related based on their Cas9 protein sequence, and the length of the repeat and spacer sequences (36 nt and approximately 30 nt, respectively) (Chylinski et al. 2013). A previous study (Fonfara et al. 2013) suggested that the distribution of Type II CRISPR-Cas loci in distantly related bacteria could be explained by horizontal gene transfer, although the ecological parameters involved were not further investigated.

The small operon size and the relatively low diversity in gene components make the Type II CRISPR-Cas systems well suited for comparative genomics analysis. Hence in this study we have investigated the Type II-A and II-C CRISPR-Cas systems to study their phylogeny and distribution in 1) a group of phylogenetically distantly related phyla and genera, and 2) in the human enteric pathogens *Campylobacter jejuni* and *Campylobacter coli*. These two *Campylobacter* species are very closely related and are both found in the agricultural environment, but although the major risk factor for *C. jejuni* is consumption of contaminated poultry meat, the risk factors for *C. coli* are more diverse and include environmental swimming and consumption of game and tripe (Doorduyn et al. 2010). These differences in environmental niches are represented in the distinct population structures of *C. jejuni* and *C. coli*, as *C. jejuni* lineages mostly lack a clear linkage to environment or host, whereas *C. coli* shows clustering into several distinct phylogenetic groups (clades) linked to environmental niches (Sheppard et al. 2013; Skarp-de Haan et al. 2014). Clade 1 of *C. coli* represents the majority of agricultural isolates, and these isolates show up to 20% of genome-wide introgression with *C. jejuni* sequences, whereas Clades 2 and 3 represent the nonagricultural environmental reservoir, the so-called riparian isolates, which show very little incorporation of *C. jejuni* sequences (Sheppard et al. 2013). The relatively small genome size of *C. jejuni* and *C. coli*, coupled with its importance as foodborne bacterial pathogen, has resulted in the public availability of greater than 4,000 genome sequences of *C. jejuni* and *C. coli* (Cody et al. 2013; Maiden et al. 2013). This makes *Campylobacter* an ideal genus for studying the genetic distribution of CRISPR-Cas within species and genera. Using a large collection of publicly available *Campylobacter* genome sequences, we show that almost all *C. jejuni* genomes contain a CRISPR-Cas system, whereas only a small proportion of *C. coli* genomes are CRISPR-Cas positive. In *C. jejuni*, the CRISPR-array is relatively small with on average only five repeats, and many CRISPR spacer alleles show a specific distribution matching that of multilocus sequence typing. Finally, nonagricultural *C. coli* genomes contain a closely related, but distinct Type II-C CRISPR-Cas system, and that the type, distribution and genomic location of the two type II-C CRISPR-Cas systems are dependent on whether the corresponding isolates had an agricultural or nonagricultural origin.

## Materials and Methods

### Bacterial Strains, Media, and Growth Conditions

*Campylobacter jejuni* strain NCTC 11168 (Parkhill et al. 2000) was routinely grown in Brucella media at 37 °C under microaerobic conditions (85% N_2_, 5% O_2_, 10% CO_2_). *Escherichia coli* TOP10 (Novagen) was grown aerobically in Luria Bertani medium at 37 °C. Where appropriate, media were supplemented with ampicillin (final concentration 100 µg ml^−^^1^).

### Comparative Genomics of CRISPR-Cas Systems in Bacterial Genomes

The GenBank database was queried using BLASTP, using the *C. jejuni* Cas9/Csn1 amino acid sequence as query, to identify genomes containing a putative type II-A or II-C CRISPR-Cas system. A total of 132 genomes encompassing 15 phylogenetic classes were included in these analyses (supplementary table S1, Supplementary Material online). The genome sequences or contigs with the CRISPR-Cas system were downloaded from the NCBI (National Center for Biotechnology Information) Genomes database (http://www.ncbi.nlm.nih.gov/genome/browse/, last accessed September 8, 2015) and through the Virginia Tech University PATRIC website (https://www.patricbrc.org/portal/portal/patric/Home, last accessed September 8, 2015) (Wattam et al. 2014), and are listed in supplementary table S1, Supplementary Material online. If the Cas9/Csn1 coding sequence was accompanied by sequences encoding Cas1 and Cas2 orthologs, then the sequences upstream and downstream of the operon were searched for CRISPR arrays using the CRISPRfinder software tool (http://crispr.u-psud.fr/Server/, last accessed September 8, 2015) (Grissa et al. 2007a, 2007b) and the CRISPR Recognition Tool CRT (Bland et al. 2007). Conservation of sequences was visualized with the Weblogo program (http://weblogo.berkeley.edu/logo.cgi, last accessed September 8, 2015) (Crooks et al. 2004). Phylogenetic analyses were performed using Cas9/Csn1 sequences aligned with ClustalX2 (Larkin et al. 2007), and distance matrix and tree construction using Phylip 3.69 and MEGA 5.2.1 (Felsenstein 1989; Tamura et al. 2011). MEGA and Figtree version 1.4.2 (http://tree.bio.ed.ac.uk/software/figtree/, last accessed September 8, 2015) were used for annotation of phylogenetic trees.

### Transcription Start Site Determination by 5′ Rapid Amplification of cDNA Ends

RNA was isolated using a hot phenol procedure (Mattatall and Sanderson 1996; Porcelli et al. 2013) to ensure that small RNAs would not be removed by the extraction procedure. The RNA was treated with DNase I to remove residual genomic DNA. The purity of the RNA was determined using the RNA 6000 Nano Kit (Agilent) according to manufacturer’s instructions. The concentration of the RNA was determined using the Nanodrop Spectrophotometer NS-1000 (Thermo Scientific). Transcription start sites in the CRISPR region of *C. jejuni* NCTC 11168 were determined using 5′ rapid amplification of cDNA ends (RACE), essentially as described previously (Porcelli et al. 2013). Briefly, 12 µg of RNA, isolated from a mid-log phase culture of *C. jejuni* NCTC 11168 using the RNeasy kit (Qiagen, UK), was treated with tobacco acid pyrophosphatase (TAP) and RNA oligonucleotide adaptor (supplementary table S2, Supplementary Material online) was ligated to the 5′-end of the treated RNA (Porcelli et al. 2013). TAP cleaves the 5′-triphosphate of primary transcripts to a monophosphate, thus making them available for ligation of the RNA adaptor. This results in an enrichment of 5′-RACE products for primary transcripts in TAP-treated RNA, in comparison with an untreated control. First-strand cDNA synthesis was performed using random hexamers, followed by polymerase chain reaction (PCR) amplification with gene-specific primers and a 5′-adaptor-specific DNA primer (supplementary table S2, Supplementary Material online). The resulting PCR products were cloned into the pGEM-T_easy_ cloning vector (Promega, UK) and the nucleotide sequence of the inserts was determined using standard protocols.

### Identification of CRISPR-Cas in *C. jejuni* and *C. coli* Genome Sequences

A total of 4,232 complete and draft genome sequences of *C. jejuni* (*N* = 3,746) and *C. coli* (*N* = 486) (supplementary table S3, Supplementary Material online) were obtained from public collections such as the NCBI Genomes database (http://www.ncbi.nlm.nih.gov/genome/browse/, last accessed September 8, 2015) and the *Campylobacter* pubMLST website (http://pubmlst.org/campylobacter/, last accessed September 8, 2015) (Jolley and Maiden 2010), and initially searched using MIST (Kruczkiewicz et al. 2013) and the BLAST+ v 2.28 suite (Altschul et al. 1990) with the *cas9* sequences from *C. jejuni* NCTC 11168 (Parkhill et al. 2000), *C. coli* Clade 2 isolate 2544 (Sheppard et al. 2013) and *C. coli* Clade 3 isolate 76339 (Skarp-de Haan et al. 2014). For screening, the *cas9*, *cas1,* and *cas2* genes were converted into consecutive 60 nt oligonucleotide sequences, and each required to match 90% with sequences in the target genome. Samples were scored positive if greater than 70% of oligonucleotides were present in the screened genome. The multilocus sequence type (MLST) clonal complex designation was determined for all genomes using MIST (Kruczkiewicz et al. 2013) with the definition file provided by the *Campylobacter* pubMLST website. *Campylobacter coli* genomes were provisionally assigned to Clades 1–3 based on a phylogenetic tree produced using feature frequency profiling (Sims et al. 2009; van Vliet and Kusters 2015) using the complete genome sequences, with clades identified by the *C. coli* genomes previously published (Sheppard et al. 2013). The individual spacers of the CRISPR arrays from 1,919 *C. jejuni* and 23 *C. coli* genomes were identified using the CRISPR recognition tool (Bland et al. 2007), combined with previously described spacer sequences and assigned to a total of 1,065 alleles (supplementary table S5, Supplementary Material online), extending the scheme initiated previously (Kovanen et al. 2014). First and last spacer alleles were coupled with the *C. jejuni*/*C. coli* MLST sequence types and clonal complexes. All 4,232 *C. jejuni* and *C. coli* genomes were searched for the presence of these 1,065 alleles using BLAST+/MIST, with 5′-TGGTAAAAT and 3′-GTTTT linkers added to the query sequence, representing the 3′-end of the upstream spacer, and the 5′-end of the downstream spacer to ensure that any hits were with a CRISPR array. To assess which genomes are predicted to encode a full-length Cas9 protein, all 4,232 genomes were (re)annotated using Prokka 1.12beta and searched with the *C. jejuni* NCTC 11168 and *C. coli* 76339 Cas9 protein sequences using BLAST (Basic Local Alignment Search Tool).

### Prediction of Putative Targets of *C. jejuni* CRISPR Spacers

The 1,065 *Campylobacter* CRISPR spacer alleles were used as query for the CRISPRTarget website (http://brownlabtools.otago.ac.nz/CRISPRTarget/crispr_analysis.html, last accessed September 8, 2015) (Biswas et al. 2013), and used to search the GenBank-Phage, Refseq-Plasmid, and Refseq-Viral databases. The results list was manually curated by removing duplicate hits to plasmids such as pVir, pTet and prophages such as CJIE4, and only *Campylobacter* targets were included.

## Results

### Type II CRISPR-Cas Systems Can Be Delineated on Cas-Proteins and Repeat Sequences

**Fig. 1.—:**
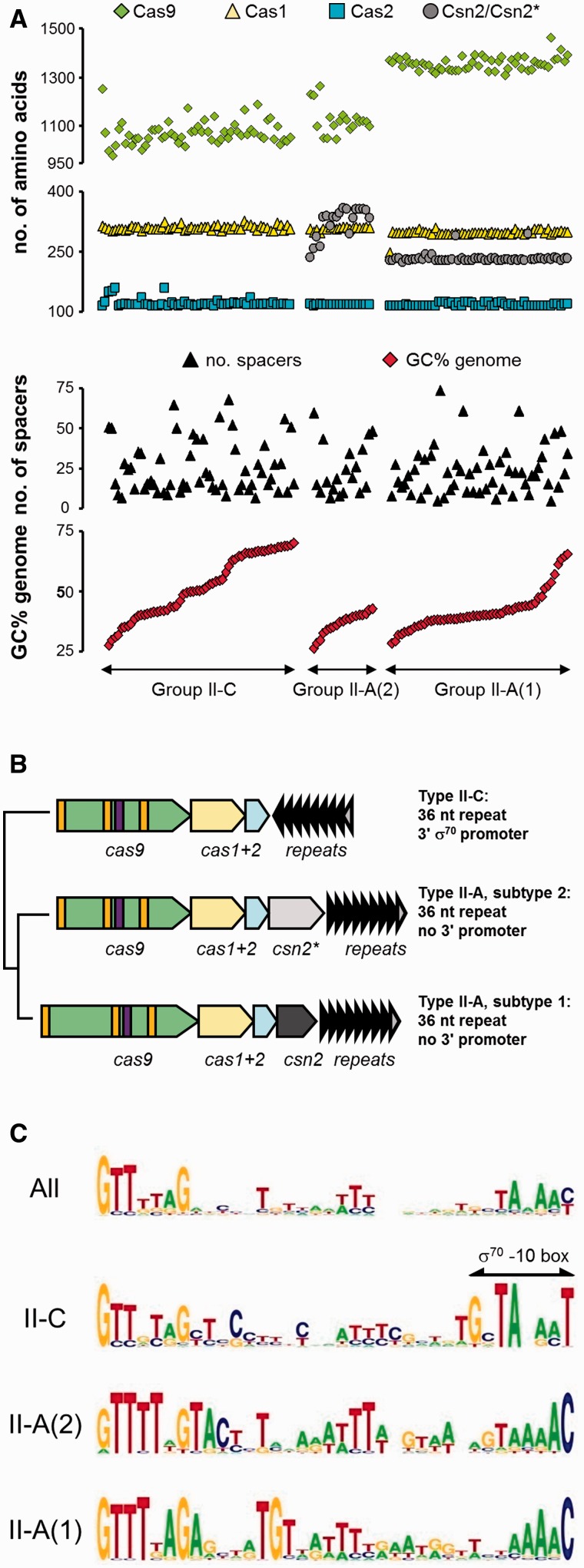
Type II-A and Type II-C CRISPR-Cas systems differ in Cas gene content and characteristics, repeat sequence and genetic organization. (*A*) Graph showing predicted number of amino acids of the Cas9, Cas1, Cas2, and Csn2/Csn2-like proteins of 132 CRISPR-Cas systems from 15 phylogenetic classes (supplementary table S1, Supplementary Material online). Green diamonds: Cas9, yellow triangles: Cas1, blue squares: Cas2, gray circles: Csn2/Csn2-like (indicated as Csn2*), as well as the number of spacers in the CRISPR array of the specific isolate/genome included. The order of the 132 species is from low to high G+C percentage of the genome (shown at the bottom). (*B*) Schematic representation of the organization of the two different Type II-A and the Type II-C CRISPR-Cas systems. Orange lines in the *cas9* genes indicate the relative position of the three encoded RuvC domains, the purple box shows the encoded HNH domain. The position of the CRISPR repeats and their transcriptional orientation compared with the Cas-genes is shown by the direction of the arrows (antisense and convergent for Type II-C, downstream and same direction for the Type II-A systems). The gray arrowhead indicates the repeat showing sequence diversity. (*C*) The Type II-C and II-A CRISPR-Cas systems have different repeat sequences, as shown by Weblogo representations of sequence conservation in CRISPR-repeats. The top logo shows the sequence conservation in all 132 CRISPR repeats, the three logo’s below show the subtypes. The position of the predicted -10 TATA box of the σ^70^ promoter in the Type II-C repeat is indicated above the sequence. The logo for Type II-C does not include the repeats from five species, for which the σ^70^ promoter is predicted to be located elsewhere in the repeat (table 1 and supplementary table S1, Supplementary Material online).

Phylogenetic relationships or ecological separation and the opposite, shared habitats, may both be forces driving CRISPR-Cas dissemination (Chylinski et al. 2014). We therefore compared if phylogeny or rather, ecology, better fitted the distribution of Type II-A and II-C CRISPR-Cas system variants across a broad collection of Gram-positive and Gram-negative bacteria. We extracted the Type II-A and II-C CRISPR-Cas systems from the genomes of 132 different species, representing 15 phylogenetic classes including Planctomycetes, Firmicutes, Proteobacteria, and Actinobacteria (supplementary table S1, Supplementary Material online). Seventy-four of these were Type II-A based on the presence of a *csn2*-like gene, with the remaining 58 being Type II-C. Analysis of predicted molecular weights of the Cas-proteins confirmed that there are two subtypes of Type II-A systems, as 55 Type II-A systems contained a large Cas9 protein and a small Csn2-like protein (tentatively named Type II-A(1)) (table 1, supplementary table S1, Supplementary Material online, and fig. 1*A*), whereas 19 Type II-A systems had a smaller Cas9 and a larger Csn2-like protein (Type II-A(2)) (table 1, supplementary table S1, Supplementary Material online, and fig. 1*A*). There was no discernible difference in molecular weight between the Cas1 and Cas2 proteins of these two subtypes. In contrast, there was no subdivision of Cas9 molecular weight in the Type II-C systems (fig. 1*A*). The major difference between the Cas9 proteins of the Type II-A(1) and II-A(2) systems is due to a spacer region between the first two RuvC domains (fig. 1*B*) (Magadan et al. 2012). The Type II-A(2) systems were only found in genomes with less than 50% G+C, whereas the majority of genomes with a Type II-A(1) system also have less than 50% G+C; in contrast, almost half of the genomes with a Type II-C system were greater than 50% G+C (fig. 1*A*). There was no difference in the average number of repeats per Type II system included (each approximately 20 ± 15 spacers), although there was significant variation per individual species/genome (fig. 1*A*).

**Table 1:** Overview of the Cas-Proteins and CRISPR Repeat Sequences from Representative Examples of Type II CRISPR-Cas Systems

Species (Family)/CRISPR-Cas Type	Cas9 (aa)^a^	Cas1 (aa)	Cas2 (aa)	Csn2 (aa)^b^	CRISPR Repeat Sequence^c^
**CRISPR-Cas Type II-C**	1,077 ± 49	303 ± 5	112 ± 10	Absent	
*Campylobacter jejuni* (ε-proteobacteria)	984	296	143	Absent	GTTTTAGTCCCTTTTTAAATTTCTTTAT**GGTAAAAT**
*Dinoroseobacter shibae* (α-proteobacteria)	1,079	303	114	Absent	GTTGCGGCTGGACCCCGAATTCTGAACA**GCTAAACT**
*Neisseria lactamica* (β-proteobacteria)	1,082	304	108	Absent	GTTGTAGCTCCCTTTCTCATTTCGCAGT**GCTACAAT**
*Haemophilus parainfluenzae* (γ-proteobacteria)	1,054	305	108	Absent	GTTGTAGCTCCCTTTTTCATTTCGCAGT**GCTATAAT**
*Clostridium perfringens* (Firmicutes)	1,065	299	107	Absent	GTTATAGTTCCTAGTAAATTCTCGATAT**GCTATAAT**
*Bacillus smithii* (Firmicutes)	1,088	299	106	Absent	GTCATAGTTCCCCTAAGATTATTGCTGT**GATATGAT**
*Ilyobacter polytropus* (Fusobacteria)	1,092	300	106	Absent	GTTGTACTTCCCTAATTATTTTAGCTAT**GTTACAAT**
*Acidothermus cellulolyticus* (Actinobacteria)	1,138	295	108	Absent	GCTGGGGAGCCTGTCTCAATCCCCCG**GCTAAAAT**GG
*Sphaerochaeta globus* (Spirochaetes)	1,179	300	108	Absent	GTTGGGGATGACCGCTGATTTTT**GTTAAGAT**TGACC
**CRISPR-Cas Type II-A (2)**	**1,130 ± 60**	**301 ± 4**	**107 ± 1**	**317 ± 38**	
*Eubacterium ventriosum* (Firmicutes)	1,107	305	108	331	ATTTTAGTACCTGAAGAAATTAAGTTATCGTAAAAC
*Staphylococcus lugdunensis* (Firmicutes)	1,054	300	107	333	GTTTTAGTACTCTGTAATTTTAGGTATAAGTGATAC
*Streptococcus thermophilus* (cr1) (Firmicutes)	1,122	303	107	350	GTTTTTGTACTCTCAAGATTTAAGTAACTGTACAAC
*Mycoplasma canis* (Mollicutes)	1,233	292	104	251	GTTTTAGTGTTGTACAATATTTGGGTAAACAATAAC
**CRISPR-Cas Type II-A (1)**	**1,364 ± 28**	**291 ± 8**	**108 ± 3**	**225 ± 12**	
*Coriobacterium glomerans* (Actinobacteria)	1,384	292	111	226	GTTTTGGAGCAGTGTCGTTCTGACTGGTAATCCAAC
*Listeria innocua* (Firmicutes)	1,334	288	113	223	GTTTTAGAGCTATGTTATTTTGAATGCTAACAAAAC
*Streptococcus thermophilus* (cr3) (Firmicutes)	1,388	289	114	219	GTTTTAGAGCTGTGTTGTTTCGAATGGTTCCAAAAC
*Treponema denticola* (Spirochaetes)	1,395	290	106	224	GTTTGAGAGTTGTGTAATTTAAGATGGATCTCAAAC
**CRISPR-Cas Type II-B**	**1,458 ± 116**	**330 ± 8**	**98 ± 1**	**195 ± 2 ****^b^**	
*Legionella pneumophila* (γ-proteobacteria)	1,372	330	99	197	CCAATAATCCCTCATCTAAAAATCCAACCACTGAAAC
*Franciscella novicida* (γ-proteobacteria)	1,630	319	97	196	CTAACAGTAGTTTACCAAATAATTCAGCAACTGAAAC
*Wolinella succinogenes* (ε-proteobacteria)	1,409	332	96	193	GCAACACTTTATAGCAAATCCGCTTAGCCTGTGAAAC
*Sutterella wadsworthensis* (β-proteobacteria)	1,422	338	98	195	GCGAAGATCATAACGCTACGAGCTATAGCACTGAAAC

^a^Numbers in bold typeface give the average molecular weight ± standard deviation for 55 Type II-A (1), 19 Type II-A (2), and 58 Type II-C species.

^b^The Type II-B protein in the Csn2 column is Cas4.

^c^Predicted -10 boxes (gnTanaaT) are underlined and marked in gray background. A full list of genomes, repeats, and Cas genes is given in supplementary table S1, Supplementary Material online.

Subdivision of the CRISPR-repeat sequences according to the Type II-A(1), Type II-A(2), and Type II-C showed a clear difference between the Type II-C and II-A sequences, due to the presence of a potential σ^70^ promoter sequence at the 3′ end of the repeats (gnTAnaaT), whereas the repeats in the Type II-A(1) and Type II-A(2) systems have a 3′ C-residue and lack the canonical residues of the σ^70^ promoter sequence (-7 T, -12 T) (fig. 1*C*). This was also reflected in the predicted transcriptional orientation of the CRISPR repeat array, which is downstream of the Cas-genes, but convergent on the antisense strand in Type II-C systems, but commonly on the same strand in the Type II-A systems (fig. 1*B* and supplementary table S1, Supplementary Material online). Sequence degeneration was commonly observed in the proximal repeat in the Type II-C systems, and in the terminal repeat of the Type II-A systems (fig. 1*B*). Some CRISPR-repeats from Type II-C systems did not have potential 3′ σ^70^ promoter sequence, such as *Acidothermus cellulolyticus* and *Sphaerochaeta globus*, but further inspection of the repeat sequence highlighted the presence of a putative σ^70^ promoter sequence of a few nucleotides moved toward the 5′-end (table 1 and supplementary table S1, Supplementary Material online).

### Distribution of CRISPR-Cas Systems throughout Distantly Related Bacterial Phyla Is Not Dependent on Phylogenetic Relationships

**Fig. 2.—:**
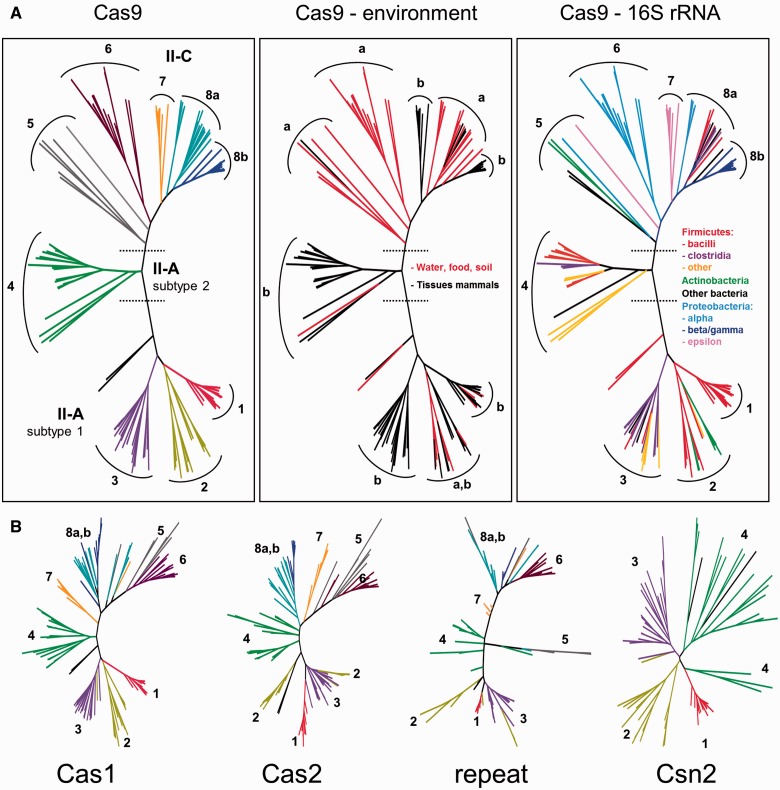
The distribution of CRISPR-Cas systems in 132 bacterial phyla is not dependent on phylogenetic relationships, but driven by shared environmental niches. (*A*) A phylogenetic tree of Cas9 amino acid sequences is depicted as an unrooted tree, with the Type II-A(1), Type II-A(2), and Type II-C subtypes indicated. The left panel has nine different subclades indicated by different colors for comparison with (*B*). The middle panel shows the same tree, but colored by the respective environments where the bacterial species were isolated, with red representing water, food, insects and soil sources, and black the tissues of mammals (e.g., GI-tract, nasopharynx). The right panel shows the phylogenetic assignment of the respective species, showing that Cas9 phylogeny does not follow 16S rDNA-based phylogeny. (*B*) Unrooted phylogenetic trees of the Cas1, Cas2, and Csn2/Csn2-like amino acid sequences and CRISPR repeat DNA sequence, colored like the left panel in (*A*). The phylogenetic trees show similar groupings into subclades. Annotated trees are supplied in supplementary figure S1*a–f*, Supplementary Material online.

Phylogenetic trees of each of the Cas-proteins and the CRISPR-repeat were constructed to check correlations whether the differences in repeats and types of Cas-genes are reflected in the phylogenetic relationships between the 132 bacterial species included in figure 1. This analysis confirmed the subdivision of Type II-A in two Cas9 subclasses across the phyla (fig. 2). This subdivision was also visible for the Cas1 and Cas2 proteins, and also when the 36 nt CRISPR-repeat sequence was used unaligned in a phylogenetic tree (fig. 2). The Type II-C Cas9, Cas1, Cas2 and CRISPR-repeat sequences clustered together, but like the Type II-A CRISPR-Cas shows distinct subclades.

To assess whether shared environments or phylogenetic relatedness are strong drivers for dissemination of CRISPR-Cas between genera/families/orders, we combined the Cas9 tree with the phylogenetic classification of the organisms, which showed that the majority of the Type II-A(1) and II-A(2) are members of the Firmicutes, but do not cluster according to their 16 S rDNA-based relationship (fig. 2*A* and supplementary fig. S1, Supplementary Material online). This is even clearer for the Type II-C systems, as these contain a wide variety of Proteobacteria, Actinobacteria, Firmicutes and Spirochaetes, which do not cluster according to phylogenetic classifications. Instead, when combined with the recorded natural niche or the source of isolation, there was a clear subclustering according to environmental sources (defined as water, plants, insects, food) versus animal tissues (gastrointestinal [GI]-tract, mucosal surfaces). This is again especially visible in the Type II-C systems, which show the same subclusters for Cas9, Cas1, Cas2 and the repeats, which mostly related by environmental niche (fig. 2 and supplementary table S1, Supplementary Material online). Except for subgroups 4 (II-A(2)), 6 and 7 (II-C), the groups contained genomes with different G+C percentages (supplementary table S1, Supplementary Material online). As most species containing a Type II-A(1) or Type II-A(2) systems are GI-tract associated, we could not assess the possible relationship with the environment. As similar subclustering was seen for all Cas genes and the CRISPR repeat (fig. 2 and supplementary fig. S1, Supplementary Material online), we hypothesize that Type II CRISPR-Cas systems are transferred as a complete unit, rather than individual components.

### Differential Distribution of Type II-C CRISPR-Cas Systems in *C. jejuni* and *C. coli*

As horizontal gene transfer has been suggested as an important driver for CRISPR-Cas dispersal (Fonfara et al. 2013), we have performed an in-depth analysis of CRISPR-Cas distribution in *C. jejuni* and *C. coli*, as these two related but distinct species are supposed to have experienced recent gene flows due to a partially shared ecology (Sheppard et al. 2008, 2013). In order to analyze the distribution of CRISPR-Cas in *C. jejuni* and *C. coli*, the CRISPR-Cas systems of *C. coli* Clade 1, Clade 2, and Clade 3 isolates (*C. coli* RM4661, 2544 and 76339, respectively) were compared with the *C. jejuni* NCTC 11168 CRISPR-Cas system (supplementary fig. S2, Supplementary Material online). The *C. jejuni*, *C. coli* Clade 1 and Clade 2 CRISPR-Cas systems consist of a *cas9*–*cas1*–*cas2* operon, but the *C. coli* Clade 3 CRISPR-Cas system only contains a *cas9* gene (BN865_15240c) without *cas1* or *cas2* genes (supplementary fig. S2, Supplementary Material online). None of the selected genomes contained other *cas* genes elsewhere. Alignment of the predicted Cas9 proteins showed that the *C. jejuni* and *C. coli* Clade 1 Cas9 proteins were virtually identical, but are only 61.0% identical (598 of 981 residues) and 88.6% similar to the Cas9 proteins from the Clade 2 and Clade 3 *C. coli* genomes. The three RuvC domains and the HNH domains are conserved, suggesting that these Cas9 proteins have the characteristic nuclease functions (supplementary fig. S3, Supplementary Material online).

**Fig. 3.—:**
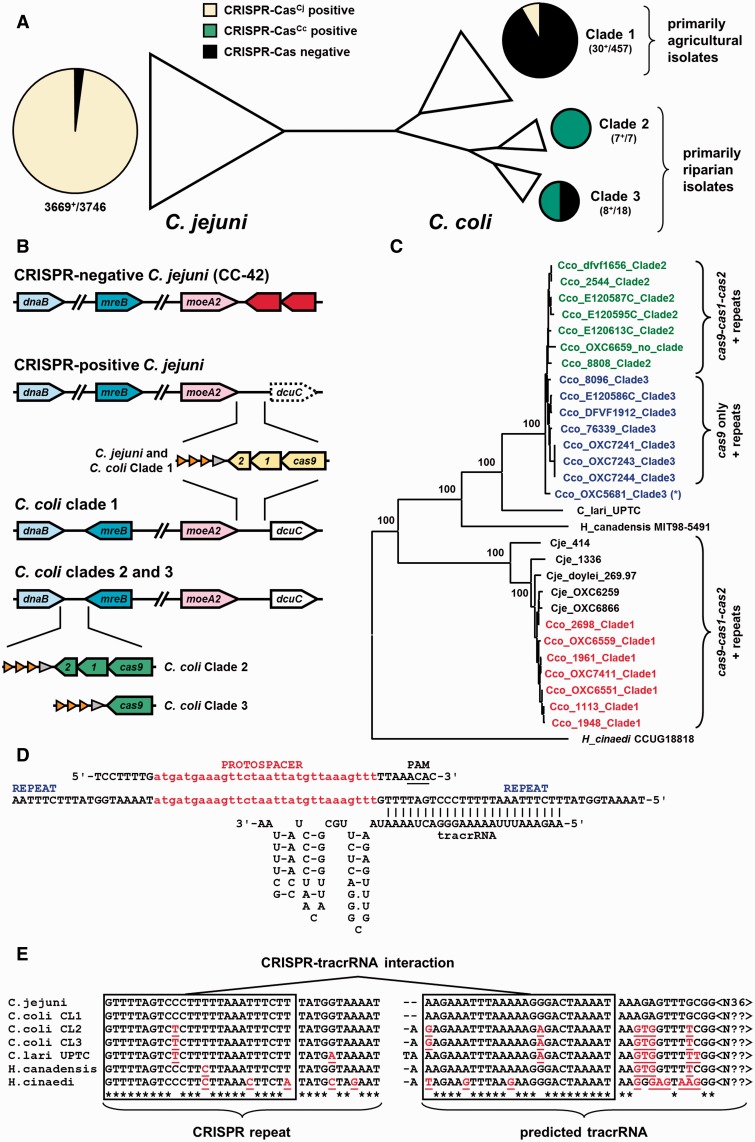
Differential distribution and genomic location of two related CRISPR-Cas systems in 4,232 *C. jejuni* and *C. coli* genomes. (*A*) Distribution of the *C. jejuni*-type CRISPR-Cas (yellow) and *C. coli*-type CRISPR-Cas (green) systems, shown on a schematic representation of the genetic population structure of *C. jejuni* and *C. coli*. Although *C. jejuni* is mostly positive for the *C. jejuni*-type CRISPR-Cas system (98.0%, table 2 and supplementary table S3, Supplementary Material online), it lacks the *C. coli*-type CRISPR-Cas system. In contrast, the majority of the Clade 1 (agricultural) *C. coli* genomes lacks CRISPR-Cas (black), whereas all Clade 2 genomes and half of the Clade 3 genomes are positive for the *C. coli*-type CRISPR-Cas (green). The four *C. coli* genomes not included in the respective clades are not shown. (*B*) Schematic representation of genomic position and surrounding genes for the *C. jejuni*- and *C. coli*-type CRISPR-Cas systems. Gene names are based on the *C. jejuni* NCTC 11168 nomenclature, with as gene numbers Cj0563 (*dnaB*), Cj1282 (*mreB*, also named *rodA*), Cj1519 (*moeA2*), and Cj1528 (*dcuC*). Dashed lines represent that *dcuC* is a pseudogene in most *C. jejuni* genomes. Red genes represent the CJJ81176_1512 and CJJ81176_1513 genes of *C. jejuni* 81-176. Orange arrows are schematic representation of the CRISPR-repeats, the gray arrow represents the (putative) tracrRNA. (*C*) The *C. coli*-type Cas9 and *C. jejuni*-type Cas9 proteins are related but distinct, as shown in a phylogenetic tree created by alignment of predicted amino acid sequences with Mega v5.21 (Tamura et al. 2011) followed by construction of neighbor-joining phylogenetic tree. Bootstrap values are provided at the main nodes, based on 500 iterations. Cco: *C. coli*, Cje: *C. jejuni*; blue names: *C. coli* Clade 3; green: *C. coli* Clade 2; red: *C. coli* Clade 1. For each subgroup, it is indicated whether they contain a *cas9–cas1–cas2* + repeats or *cas9* only + repeats. The asterisk represents a Clade 3 *C. coli* genome that contains a *cas9–cas1–cas2* + repeats configuration. (*D*) Sequence and predicted structural details for the *C. jejuni* CRISPR-Cas system elements. A section of the CRISPR array is shown (center) with the corresponding protospacer (top), including flanking sequences (±8 nt) comprising the PAM at the 3′-end of the protospacer. The tracrRNA sequence, predicted structure, and complementary anti-CRISPR repeat are shown below, based on Briner and Barrangou (2014). (*E*) Comparison of the CRISPR-repeats and predicted tracrRNA part of the *C. jejuni*, three *C. coli* clades, *C. lari*, *H. canadensis* and *H. cinaedi* CRISPR-Cas systems. The *C. coli* Clade 1 CRISPR-Cas components are identical to the *C. jejuni* version, whereas the *C. coli* Clade 2 and Clade 3 systems are identical to each other but different from *C. jejuni*. The changes in the CRISPR-repeat are matched by corresponding changes in the tracrRNA sequence, as indicated by red underlined residues. Asterisks indicate conserved nucleotides, boxes indicate the complementary sequences in CRISPR repeat and tracrRNA. The predicted 5′-end of the tracrRNA is based on the seventh nucleotide downstream of the σ^70^ promoter sequence (TGnTanaaT) downstream of the CRISPR-repeat array, whereas the [N??] at the end of the tracrRNA indicates that the 3′-end has not been mapped and hence exact length of the tracrRNA is unknown.

To assess whether the differences in CRISPR-Cas systems were representative for *C. jejuni* and *C. coli*, we searched 3,746 *C. jejuni* genome sequences and 486 *C. coli* genomes for the *cas9*, *cas1* and *cas2* genes from *C. jejuni* NCTC 11168 and the *cas9*, *cas1* and *cas2* genes of *C. coli* 2544 and *cas9* gene of *C. coli* 76339 (fig. 3*A*, table 2, and supplementary table S3, Supplementary Material online). There was a very clear difference between *C. jejuni* and *C. coli*, as 3,669 out of 3,746 (98.0%) of *C. jejuni* genomes are positive for *C. jejuni*-type *cas9* gene, but all negative for *C. coli*-type *cas9* gene (fig. 3*A* and table 2). In *C. jejuni*, the large majority (68 of 75) of CRISPR-Cas negative isolates belong to the MLST clonal complex CC-42, and this lineage contains only three CRISPR-Cas positive isolates (table 2 and supplementary table S3, Supplementary Material online). All other major *C. jejuni* MLST clonal complexes are greater than 99% positive for the *C. jejuni*-type *cas9* gene, and also contain *cas1* and *cas2* genes (table 2). Around 80% of the *C. jejuni* and 90% of the *C. coli* genomes with a *C. jejuni*-type *cas9* gene are predicted to express a full length Cas9 protein (2,905/3,669 and 27/30, respectively; supplementary table S3, Supplementary Material online), whereas this is 81% for *C. coli* genomes with a *C. coli*-type *cas9* gene (13/16, supplementary table S3, Supplementary Material online).

**Table 2:** Distribution of CRISPR-Cas in *Campylobacter jejuni* and *Campylobacter coli* Lineages

**Species/Clade/MLST**^a^^,^^b^	**Cj CRISPR**	**Cc CRISPR**	**Negative**	**Total**
	**(%)**	**(%)**	**(%)**	
*C. jejuni*				
ST-21	1,093 (100)	0	0	1,093
ST-42	4 (5.6)	0	68 (94.4)	72
St-45	258 (99.6)	0	1 (0.4)	259
ST-48	243 (100)	0	0	243
Other or no clonal complex^c^	2,071 (99.6)	0	8 (0.4)	2,079
Total	3,669 (98.0)	0	77 (2.0)	3,746
*C. coli*				
Clade 1 (primarily ST-828)	29 (6.4)	0	421 (93.6)	450
Clade 1 (primarily ST-1150)	1 (14.3)	0	6 (85.7)	7
Clade 2	0	7 (100)	0	7
Clade 3	0	8 (44.4)	10 (55.6)	18
No clade^d^	0	1 (25)	3 (75)	4
Total	30 (6.2)	16 (3.3)	440 (90.5)	486

Note.—Cj CRISPR, presence of *C. jejuni* NCTC 11168 *cas9* (*cj1523*) ortholog (Gundogdu et al. 2007); Cc CRISPR, presence of *C. coli* 76339 *cas9* (BN865_15240c) ortholog (Skarp-de Haan et al. 2014).

^a^MLST clonal complexes definitions were obtained from http://pubmlst.org/campylobacter.

^b^Number of draft and complete genome sequences obtained from published studies (Richards et al. 2013; Sheppard et al. 2013) and draft genome sequences deposited in NCBI and pubMLST (Jolley and Maiden 2010; Cody et al. 2013).

^c^Other clonal complexes represented are CC-22, 49, 52, 61, 206, 257, 283, 353, 354, 362, 403, 433, 443, 446, 460, 464, 508, 573, 574, 607, 658, 661, 677, 692, 702, 1034, 1275, 1287, and 1332. No clonal complex: *N* = 261.

^d^No clade isolates are also called Clade 1 c (Skarp-de Haan et al. 2014).

Conversely, only 46 out of 476 (9.7%) of *C. coli* genomes are positive for a *cas9* gene (fig. 3*A*). Of these, 30 contain a *C. jejuni*-type *cas9* gene with *cas1* and *cas2* genes, and 16 contain a *C. coli*-type *cas9* gene. The CRISPR-Cas locus of the 16 *C. coli*-type *cas9*-positive genomes was further checked, and eight of these contain only a *C. coli*-type *cas9* gene, whereas the other eight contain a complete *C. coli*-type *cas9*–*cas1*–*cas2* operon (fig. 3*B*, supplementary figs. S2 and S3, table S3, Supplementary Material online). Assignment of *C. coli* genomes to Clades 1–3 using whole-genome comparisons by feature frequency profiling (Sims et al. 2009; van Vliet and Kusters 2015) showed that the distribution of CRISPR-Cas systems matched with assignment to the three clades: CRISPR-positive *C. coli* Clade 1 genomes contain the *C. jejuni*-type *cas9*–*cas1*–*cas2* operon, Clade 2 genomes contain the *C. coli*-type *cas9*–*cas1*–*cas2* operon, and Clade 3 genomes contain only the *C. coli*-type *cas9* gene (fig. 3*B*, table 2, and supplementary table S3, Supplementary Material online). There was only a single exception, with *C. coli* OXC5681 (Clade 3) containing a *C. coli*-type *cas9*–*cas1*–*cas2* operon. None of the genomes investigated contained both the *C. jejuni* and *C. coli*-type CRISPR-Cas systems.

An alignment of Cas9 amino acid sequences of *C. jejuni*, the three *C. coli* clades, and Type II-C Cas9 proteins of other *Campylobacter* and closely related *Helicobacter* species confirmed that the two Cas9 proteins are phylogenetically distinct, and when presented as a phylogenetic tree, are separated by the *Campylobacter lari* and *Helicobacter canadensis* Cas9 proteins (fig. 3*C*). Trees constructed from alignments of the *C. jejuni*, *C. coli*, *C. lari*, *H. canadensis,* and *H. cinaedi* Cas1 and Cas2 proteins gave similar tree topologies supported by high bootstrap scores that were somewhat lower (>96%) for Cas2 (supplementary fig. S3, Supplementary Material online).

### The Genomic Location of the Two *Campylobacter* CRISPR-Cas Types Is Conserved but Differs between the *C. jejuni* and *C. coli* Types

We investigated the flanking sequences of the respective CRISPR-Cas systems to assess whether genetic variability plays a role in the distinct distribution patters of CRISPR-Cas in *C. jejuni* and *C. coli*. In greater than 100 randomly selected CRISPR-positive *C. jejuni* genomes, the CRISPR-Cas locus is always located between homologs of the *moeA2* (*cj1519*) gene and a C4-dicarboxylate transporter pseudogene (*cj1528*), with the *cas*-genes in the opposite orientation to *moeA2* and *cj1528* (fig. 3*B*). In the 68 CRISPR-negative *C. jejuni* strains, most have the configuration of *C. jejuni* 81-176 (CC-42) which lacks the CRISPR-locus and the *cj1528* pseudogene, which have been replaced by two genes encoding hypothetical proteins (CJJ81176_1512 and CJJ81176_1513), or have a transposase-ortholog in that region (fig. 3*B*); none of the 68 genomes has the *moeA2* gene flanking the *cj1528* pseudogene. In the CRISPR-positive *C. coli* Clade 1 genomes, the gene configuration is identical to the *C. jejuni* situation (fig. 3*B* and supplementary fig. S2, Supplementary Material online), except that the *C. coli cj1528* ortholog is often not a pseudogene, but predicted to encode a functional protein.

The genomic location of the *C. coli*-type CRISPR-Cas in Clades 2 and 3 is completely different from the *C. jejuni* and Clade 1 *C. coli* genomes, as in these two clades, the *C. coli*-type CRISPR-Cas is located between the *mreB*/*rodA* and the *dnaB* genes (fig. 3*B* and supplementary fig. S2, Supplementary Material online), a region which contains a tRNA-Met. In the CRISPR-negative genomes of *C. coli* Clade 3, the *mreB* and *dnaB* genes only contain the region with tRNA-Met. The presence of variable sequences between the flanking genes in both *C. coli* and *C. jejuni* suggests acquisition of the CRISPR-Cas system by allelic exchange (fig. 3*B*). The *mreB* and *dnaB* genes are not adjacent but approximately 700 kb apart on the *C. jejuni* genome (fig. 3*B*), which may preclude acquisition of the *C. coli* CRISPR-Cas system by allelic exchange into the *C. jejuni* genome. In contrast, the *C. coli mreB*–*dnaB* and *moeA2*–*cj1528* configurations are conserved, and could allow acquisition of either the *C. jejuni* or *C. coli* type CRISPR-Cas loci by *C. coli* strains. Hence, ecological separation may explain the apparent lack of genetic exchange of CRISPR-Cas systems between riparian and agricultural isolates of *C. coli*.

### Differences in CRISPR Repeat Sequence Are Matched with tracrRNA Sequence Changes

In order to analyze the evolutionary history of the *Campylobacter* Type II CRISPR-Cas system, a more detailed insight into its sequence conservation and functional aspects including crRNA targets and CRISPR expression was first obtained. The transcriptional profile of the *C. jejuni* CRISPR array was previously investigated with RNA-seq-based technology (Chylinski et al. 2013; Dugar et al. 2013; Porcelli et al. 2013), with crRNAs being transcribed from σ^70^ promoters located in the 3′-end of the individual CRISPR repeats. We confirmed this for *C. jejuni* NCTC 11168 using 5′-RACE (supplementary fig. S4, Supplementary Material online). The 73 nt transcript transcribed downstream of the CRISPR array displays a 24 nt perfect complementarity with nucleotides 2–25 of the CRISPR-repeat (supplementary fig. S4, Supplementary Material online), consistent with RNase III cleavage of a crRNA-transcript duplex, and hence represents the tracrRNA system described for Type II CRISPR-Cas systems (fig. 3*D*) (Deltcheva et al. 2011; Chylinski et al. 2013; Fonfara et al. 2013; Briner and Barrangou 2014).

We aligned the CRISPR repeats of *C. jejuni*, the three *C. coli* clades, and the available genomes of *C. lari*, *H. canadensis* and *H. cinaedi*. These were all 36 nt, contained a σ^70^ -10 sequence (gnTAnAAT) at the 3′-end and were very similar with 30 invariable nucleotides between them (fig. 3*E*). The *C. jejuni* and *C. coli* Clade 1 CRISPR repeats were identical, whereas the *C. coli* Clade 2 and Clade 3 repeats were identical to each other, and differed only by a single nucleotide with the *C. jejuni* and *C. coli* Clade 1 repeat (fig. 3*E*). The *C. lari*, *H. canadensis,* and *H. cinaedi* CRISPR repeats show an increasing number of differences with the *C. jejuni* and *C. coli* repeats. Each CRISPR area contains a predicted tracrRNA sequence, with partial sequence complementarity to the CRISPR repeat, preceded by a σ^70^ -10 sequence (gnTAnAAT), located downstream of the CRISPR repeats. The differences between the CRISPR repeats were matched by changes in the tracrRNA sequence (fig. 3*E*) (Briner et al. 2014).

### Identification of Putative *Campylobacter* CRISPR Targets and the PAM Motif

**Fig. 4.—:**
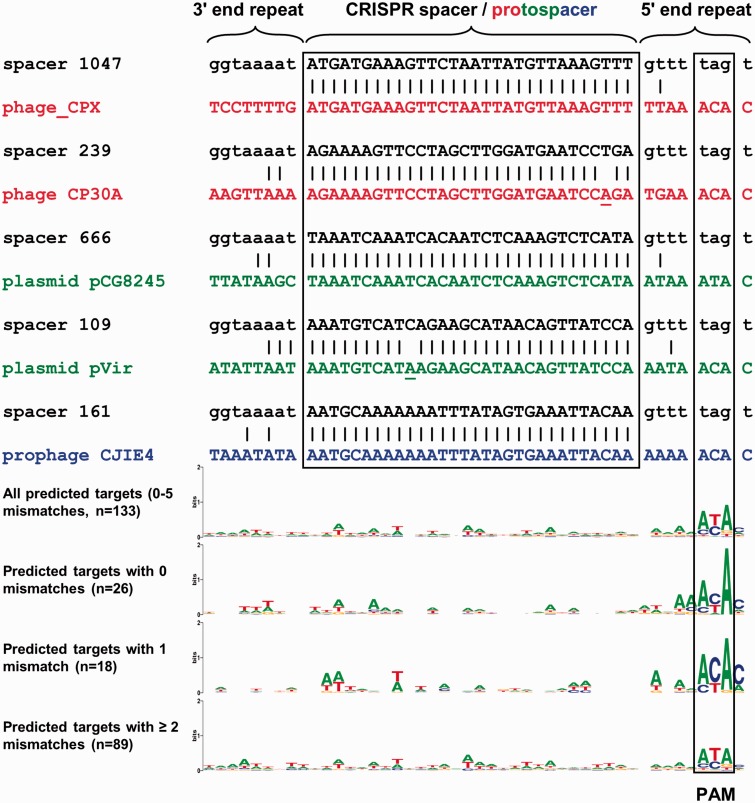
Identification of putative targets for *C. jejuni* and *C. coli* crRNA sequences and identification of a PAM motif (Deveau et al. 2008; Horvath et al. 2008; Mojica et al. 2009). The figure shows examples of protospacers in *Campylobacter* phages (2), plasmids (2), and a prophages/insertion element (1). Sequences in black represent spacer sequences with the allele number from supplementary table S5, Supplementary Material online, red represents phages, green represents plasmids, blue represents chromosomally integrated prophages/insertion elements of *C. jejuni* strain RM1221 (Fouts et al. 2005). The 3′-end of the upstream repeat and the 5′-end of the downstream repeat are shown for each spaces (black), as well as the upstream and downstream sequences of the protospacers. A full list of protospacers is given in supplementary table S5, Supplementary Material online. Weblogo analyses are shown below the alignment, to show a lack of sequence conservation in 133 protospacers and 5′ upstream sequence, but conservation of a 3′ downstream PAM motif (Deveau et al. 2008; Horvath et al. 2008; Mojica et al. 2009). The PAM motif is shown for protospacers with 0, 1, and ≥2 mismatches with the CRISPR spacer (*n* = 26, *n* = 18, and *n* = 89, respectively), and highlights the relative lack of conservation of the 5′-a(c/t)A-3′ PAM motif in protospacers with ≥2 mismatches.

The CRISPR spacer content of 1,919 *C. jejuni* and 23 *C. coli* genomes was extracted using the CRISPR Recognition Tool (Bland et al. 2007), and each unique spacer sequence was assigned an allele number, expanded from the alleles described previously for *C. jejuni* (Kovanen et al. 2014). When combined with other published *C. jejuni* and *C. coli* spacer sequences (Louwen et al. 2013), this gave a total of 1,065 *C. jejuni*/*C. coli* CRISPR spacer alleles (supplementary table S5, Supplementary Material online). The 1,065 spacer allele sequences were used to search databases for putative protospacers in *Campylobacter* phages, plasmids, and genomic insertion elements/prophages (supplementary table S4, Supplementary Material online), resulting in the identification of 133 putative protospacers with up to 5 mismatches (supplementary table S6, Supplementary Material online, and fig. 4), and these protospacers were found in either plasmids, phages, and prophages/insertion elements. Analysis of sequence conservation in the protospacers and the 5′ and 3′ sequences showed that there is no significantly conserved motif(s) at the 5′-end or in the spacer sequences, but that there is a conserved (a/c)(t/c)A motif present four nucleotides downstream of the 3′-end of the protospacer (fig. 4) which has been shown to function as protospacer adjacent motif (PAM) (Deveau et al. 2008; Horvath et al. 2008; Mojica et al. 2009; Fonfara et al. 2013). When further subdivided for the number of mismatches between the protospacer and CRISPR spacer sequence (supplementary table S6, Supplementary Material online), the relatively poor conservation of the PAM motif was mostly due to inclusion of putative targets with two or more mismatches, as the motif with 0 or 1 mismatches is 5′-acA or 5′-a(c/t)A, consistent with the finding that the ACA motif is efficiently cleaved by *C. jejuni* Cas9, but CCA is not (Fonfara et al. 2013).

### Linkage of CRISPR Spacer Alleles to Specific *C. jejuni* MLST-Types

**Fig. 5.—:**
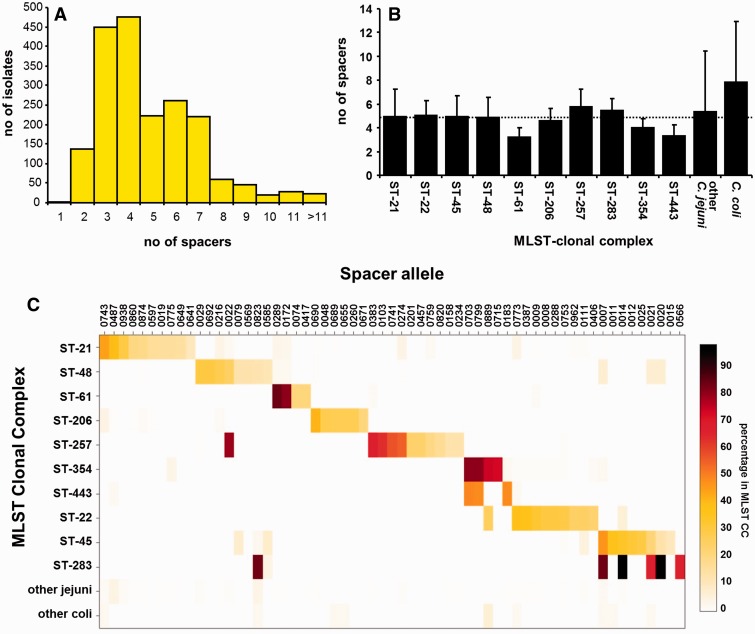
Specific CRISPR spacer alleles are linked with *C. jejuni* MLST-clonal complexes, but CRISPR spacer array size is similar in most MLST-types. (*A*) Distribution of the number of CRISPR spacers in 1,919 *C. jejuni* and 23 *C. coli* genome sequences from which CRISPR sequences could be extracted by the CRISPR Recognition Tool CRT (Bland et al. 2007). (*B*) The average number of CRISPR spacers per genome is shown for ten major *C. jejuni* MLST clonal complexes, representing 1,623 of the 1,919 *C. jejuni* genomes. The average of all 1,942 genomes is 4.9 ± 2.7 spacers, and is indicated by the dashed line. (*C*) Major spacer alleles are associated with specific MLST clonal complexes. All 4,232 *C. jejuni* and *C. coli* genome sequences were searched for the presence of the 1,065 CRISPR spacer alleles shown in supplementary table S5, Supplementary Material online. Spacer alleles were only included if present greater than 10× in total, and in at least 10% of genomes from a single MLST clonal complex. The heatmap is based on the percentage presence per MLST clonal complex, and was generated using PlotLY (https://plot.ly/feed/).

The average size of the CRISPR array in the 1,942 genomes is 4.9 ± 2.7 spacers, and ranges between 1 and 54 spacers (supplementary table S7, Supplementary Material online). The majority of CRISPR arrays contain three or four spacers (fig. 5*A*), and when subdivided according to the MLST typing scheme (in clonal complexes), there was no major difference in the number of spacers between the major *C. jejuni* MLST-types (fig. 5*B*). To assess whether spacer diversity is linked with other sequence diversity in *C. jejuni* and *C. coli*, we first looked at the distribution of proximal and terminal spacer per MLST-clonal complex (table 3). Most of the common proximal and terminal spacer alleles were only found within a single MLST clonal complex, except for ST-45 and ST-283 sharing proximal spacer allele 14, ST-48 and ST-257 sharing proximal spacer allele 22, whereas ST-21 and ST-61 share proximal allele 172 and terminal allele 289, and ST-354 and ST-443 share proximal allele 703 and terminal allele 799 (table 3). This linkage of spacer alleles with MLST clonal complexes was also observed when investigating the prevalence of the 1,065 spacer alleles in the full 4,232 genomes, as many spacer alleles were only detected within a specific MLST clonal complex (fig. 5*C*, supplementary tables S7 and S8, Supplementary Material online). Within the clonal complexes, there was also an association of proximal/distal spacers and spacer content with specific sequence types, as for example with clonal complex ST-21, proximal spacer alleles 775, 487, 23 and 596 are primarily found in sequence types ST-19, ST-21, ST-50 and ST-2135, respectively (supplementary tables S7 and S8, Supplementary Material online).

**Table 3:** Distribution of CRISPR Spacer Alleles in *Campylobacter jejuni* MLST-Clonal Complexes

**Clonal Complex**^a^ (Number of Isolates, *N*)	**No. of Spacers** (Average ± SD)	**First Spacer**^b^^,^^c^	**Last Spacer**^b^^,^^c^
		(Number of Isolates, *N*)	(Number of Isolates, *N*)
All (1,942)	4.9 ± 2.7	N/A	N/A
ST-21 (750)	4.9 ± 2.4	23 (42), **172** (29), 323 (72), 487 (19), 743 (382), 775 (27), 938 (31)	2 (26), 19 (54), 216 (23), **289** (26), 338 (63), 425 (37), 487 (63), 541 (27), 597 (74), 677 (34), 735 (36), 874 (37)
ST-22 (49)	5.1 ± 1.2	9 (20), 406 (15)	387 (17), 962 (14),
ST-45 (186)	5.0 ± 1.7	7 (82), **14** (59)	16 (23), 25 (29)
ST-48 (96)	4.9 ± 1.7	**22** (46), 569 (31)	29 (42)
ST-61 (60)	3.2 ± 0.8	**172** (57)	74 (18), **289** (13)
ST-206 (83)	4.6 ± 1.0	689 (49)	260 (47)
ST-257 (182)	5.7 ± 1.5	**22** (165)	201 (63), 741 (25), 820 (31)
ST-283 (43)	5.5 ± 0.9	**14** (42)	566 (29)
ST-354 (120)	4.0 ± 0.7	**703** (106)	240 (53), **799** (53)
ST-443 (64)	3.3 ± 1.0	**703** (53)	**799** (53)
Other jejuni (286)	5.3 ± 5.1	ND	ND
*Campylobacter coli* (23)	7.8 ± 5.1	ND	ND

^a^Clonal complexes based on the definitions available at http://pubmlst.org/campylobacter/.

^b^Spacer alleles are extended from Kovanen et al. (2014), and given in supplementary table S5, Supplementary Material online.

^c^Spacer alleles given in red (first spacer) or blue (last spacer) are shared between two different clonal complexes.

## Discussion

In the last 8 years, CRISPR-Cas has gone from a relatively obscure system of repeats in prokaryotic and archaeal genomes to one of the hottest subjects in biology. The discovery that it constituted a defense system against phages and plasmids (Barrangou et al. 2007; Brouns et al. 2008; Garneau et al. 2010) was followed by the elucidation of the molecular mechanism of Type II-A CRISPR-Cas activity (Deltcheva et al. 2011). The usage of the Cas9-crRNA-tracrRNA as a programmable dual-RNA-guided DNA endonuclease (Jinek et al. 2012) has opened up an array of possibilities in genome editing (reviewed in (Kim H and Kim JS 2014)) and transcriptional silencing (Bikard et al. 2013; Qi et al. 2013), and especially the genome editing possibilities have led to a rapid increase in PubMed entries on CRISPR/Cas9, from 92 in 2011, 144 in 2012, 342 in 2013 to 715 in 2014. These exciting opportunities created by CRISPR-Cas need to be matched by further investigation on the biology, dissemination, and evolution of CRISPR-Cas systems. Such investigations can now get additional power by the developments in high-throughput DNA sequencing, as for some bacteria there are large collections of genome sequences available in public databases such as GenBank/EMBL/DDBJ and pubMLST.

In this study we have used a collection of greater than 4,000 genome sequences to investigate the distribution of CRISPR-Cas in *C. jejuni* and *C. coli*, two closely related *Campylobacter* species which are jointly responsible for half a million cases of food poisoning in the United Kingdom annually (Nichols et al. 2012; Tam et al. 2012), with similar levels of infections in other Western countries (EFSA 2010). Despite the close phylogenetic relationship between these two species (only 4 nt difference in the 16S rDNA gene), and these species having overlapping environmental niches and hosts, there was a striking difference in the distribution of CRISPR-Cas systems, with *C. jejuni* strains being almost universally positive for CRISPR-Cas (98.0%), whereas only 9.7% of the *C. coli* genomes were positive for CRISPR-Cas (fig. 3*A* and table 2), with there being two related but distinct CRISPR-Cas systems in *C. coli*, one shared with *C. jejuni* and one specific for *C. coli*. Further investigation showed that the distribution of these two CRISPR-Cas systems strictly adheres to the genome sequence-based phylogeny of *C. coli*, which has been subdivided into three separate lineages (Clades 1, 2, and 3), with Clade 1 representing agricultural isolates, and Clades 2 and 3 representing nonagricultural (environmental, riparian) isolates (Sheppard et al. 2013; Skarp-de Haan et al. 2014). The CRISPR-Cas positive Clade 1 *C. coli* isolates only have the *C. jejuni* CRISPR-Cas system, whereas the Clade 2 and 3 isolates contain either a full CRISPR-Cas system (Clade 2) or a CRISPR-Cas9 only version (Clade 3). As this separation is consistent with the strict biosecurity imposed to combat the spread of *Campylobacter* in the food chain, it is possible that this differential distribution is at least in part caused by agricultural and hygienic practices and the biosecurity measures aimed at keeping *Campylobacter* out of the broiler houses (http://www.food.gov.uk/multimedia/pdfs/board/board-papers-2013/fsa-130904.pdf, last accessed September 8, 2015).

The two types of CRISPR-Cas in *C. jejuni* and *C. coli* isolates had specific genomic insertion sites. As both *Campylobacter* species are naturally transformable, this suggests that the CRISPR-Cas systems should be able to spread through populations by allelic exchange after natural transformation. In view of the clear differences between the three clades of *C. coli*, we conclude that the dissemination of CRISPR-Cas systems is dependent on sharing of niches between isolates. The presence of the *C. jejuni* CRISPR-Cas in a small number of Clade 1 *C. coli* isolates suggests that the direction of transfer has been from *C. jejuni* to *C. coli*, consistent with the direction of genome introgression in agricultural *C. coli* isolates (Sheppard et al. 2013; Skarp-de Haan et al. 2014). It is tempting to speculate that there is even little overlap in environmental niches between the Clades 2 and 3, as there was only a single Clade 3 isolate with the full *C. coli* CRISPR-Cas system as found in Clade 2, and no Cas9-only system in Clade 2. However, the number of isolates (table 2) is currently too small to draw such firm conclusions. It has been reported that natural transformation of *C. jejuni* can be prevented by the presence of DNA/RNA endonuclease genes (such as in *C. jejuni* strain RM1221) (Gaasbeek et al. 2009, 2010; Brown et al. 2015), and hence we did check whether there was a correlation between CRISPR-negative *C. coli* genomes and presence of the RM1221 DNases. However, there was no such correlation apparent.

In contrast to *C. coli*, most *C. jejuni* isolates are positive for the CRISPR-Cas system, although this does not imply functionality of the system. We have previously shown an association between the presence of LOS sialylation in *C. jejuni* and a proposed lack of functionality of the CRISPR-Cas system (Louwen et al. 2013), and in *C. jejuni* RM1221 the CRISPR array is not transcribed, probably due to the inactive *cas9* gene (Dugar et al. 2013). We used the genome sequences to predict whether the encoded Cas9 protein was full length (implying functionality), and found that in approximately 80% of genomes this was indeed the case. However, this number needs to be interpreted with caution, as in some cases the *cas9* may be fragmented due to genome assembly into different contigs. The *C. jejuni* isolates completely lacking CRISPR-Cas were virtually all from a single MLST clonal complex (CC-42), and all contained a genetically reorganized region downstream of the *moeA2* gene (fig. 3*B*), which may explain why these isolates have not (re)acquired the CRISPR-Cas system. Although there is no clear link between population structure and environmental niches apparent in *C. jejuni*, it is important to note that the large majority of *C. jejuni* genomes were obtained from the pubMLST collection, and those are mostly clinical isolates from an ongoing survey in Oxfordshire, UK (Cody et al. 2013). There are very few genomes available for water or wildlife isolates of *C. jejuni* with the exception of *C. jejuni* strains 1336 and 414 (Hepworth et al. 2011), and these are both CRISPR-Cas positive. Interestingly, a large scale comparative genomics hybridization study of *C. jejuni* (Stabler et al. 2013) reported that the C9 clade (representing wildlife and water isolates) was 86–88% positive for CRISPR-Cas, whereas all other clades were 100% positive for CRISPR-Cas. Hence there may be a reduced presence of CRISPR-Cas in nonagricultural isolates in *C. jejuni* as well, albeit not as clear as in *C. coli*. It will be necessary to get a better representation of genomes from nonagricultural/wildlife and water *C. jejuni* isolates to draw any final conclusions on whether the situation is different in *C. jejuni*.

We used the availability of CRISPR-arrays to search for potential targets of the *C. jejuni* and *C. coli* CRISPR-Cas systems, and found strong matches with *Campylobacter* bacteriophages, plasmids, and insertion elements/prophages (fig. 4 and supplementary table S5, Supplementary Material online). The conservation of the protospacer-adjacent motif PAM (Deveau et al. 2008; Horvath et al. 2008; Mojica et al. 2009) was dependent on the number of mismatches between spacer and protospacer (fig. 4), and suggests that some of the matches found by bioinformatic analyses may be erroneous. However, as with many arrays of CRISPR-spacers, there were many spacers which did not match any plasmid/phage sequence in the databases, or matched plasmids/phages from phylogenetically distinct genera/phyla. It is not known whether these spacers are functional, nor is it known what the frequency of spacer turnover is in *C. jejuni* and *C. coli*, although a recent study has shown that *C. jejuni* may use a phage-encoded Cas4-like protein for acquisition of new spacers (Hooton and Connerton 2014). Spacer acquisition in *Streptococcus thermophilus* requires the concerted action of Cas9, Cas1, Cas2 and Csn2 (Heler et al. 2015), and hence the *C. coli* Clade 3 CRISPR-Cas9-only system may not be able to acquire new spacers. However, it is conceivable that CRISPR-spacers can potentially also be exchanged by natural transformation and allelic exchange (Kupczok and Bollback 2013), and hence this may be an alternative mechanism for *C. coli* isolates lacking *cas1* and *cas2* to acquire new spacers.

In this study we have extended a previous CRISPR-spacer-based typing analysis for *C. jejuni* (Kovanen et al. 2014) by adding 1,028 new spacer alleles, and have investigated their distribution in *C. jejuni* and *C. coli* genomes. There was a very clear correlation of spacer allele distribution with MLST-clonal complexes, and within these clonal complexes with specific sequence types (fig. 5, table 3, and supplementary tables S7 and S8, Supplementary Material online), suggesting that CRISPR spacers may be usable for typing epidemiology purposes, and suggests a relatively low turnover of spacers in *C. jejuni* and *C. coli*. This matches similar findings with regard to the links between specific spacer alleles and sequence-based typing methods (Briner and Barrangou 2014; Pettengill et al. 2014; Lier et al. 2015). There are also links between CRISPR-Cas and virulence (Louwen et al. 2013, 2014; Sampson et al. 2013, 2014; Fiebig et al. 2015; Jiang et al. 2015), and as CRISPR-Cas expression can affect virulence gene acquisition during infection (Bikard et al. 2012), it is of importance to further study the mechanisms by which CRISPR-Cas is transferred within species/genera, as well as between different phyla, and what the drivers are of such transfer, such as shared environmental niches or phylogenetic relationships. For this we have included a comparison of Type II-A and II-C CRISPR-Cas systems of 132 different bacterial species, covering 15 phyla (figs. 1 and 2, supplementary fig. S1, Supplementary Material online). Although such an analysis can only retrospectively assess the relationship between the CRISPR-Cas systems, there are patterns emerging. Comparison of phylogenetic trees of Cas9, Cas1, Cas2, Csn2/Csn2-like, and CRISPR-repeat showed very similar lineages in all trees, and these lineages often contained species from different phyla, which shared a similar ecological niche when subdivided into those species colonizing mucosal surfaces in mammalian hosts versus those colonizing environmental niches such as soil and plants. Similar studies have been described previously (Chylinski et al. 2013, 2014; Fonfara et al. 2013), but focused on evolution of these systems or analyzed Cas9 phylogeny only. The similarities between the trees obtained using Cas9, Cas1, Cas2, Csn2/Csn2-like, and the CRISPR-repeat strongly suggest that the CRISPR-Cas system is inherited as a complete module (Godde and Bickerton 2006; Kunin et al. 2007; Horvath et al. 2009). As CRISPR-Cas has also been described to prevent natural transformation (Marraffini and Sontheimer 2008; Bikard et al. 2012; Weinberger and Gilmore 2012; Zhang et al. 2013; Sampson et al. 2014), this opens the intriguing possibility of CRISPR-Cas as a form of mobile or even selfish DNA, but this requires functional studies in horizontal transfer of CRISPR-Cas between species from different phyla.

The CRISPR-Cas system of prokaryotes is now recognized as a highly fascinating RNA interference system with significant opportunities for biotechnology tools development. Although its role in phage defense is now well established, and its credentials for genome editing and gene regulation are clear to the scientific community, there is still a need to investigate CRISPR-Cas in the context of genome evolution, transmission, and intraspecies and interspecies dissemination. In this study we have successfully used the publicly available genome sequence resources available for *Campylobacter* to study the distribution of CRISPR-Cas in this important foodborne pathogen, and show a striking difference in CRISPR-Cas distribution between the two major human pathogenic species *C. jejuni* and *C. coli*, and within *C. coli* a marked difference between agricultural isolates. The lack of any evidence of exchange of the two related, but distinct CRISPR-Cas systems in *C. coli* suggests that there is a physical separation between the two types of isolates, and hence that the strict biosecurity in the agricultural sector functions well in that respect.

## Supplementary Material

Supplementary figures S1–S4 and tables S1–S8 are available at *Genome Biology and Evolution* online (http://www.gbe.oxfordjournals.org/).

Supplementary Data
