# Supplementary material for: Differential Distribution of Type II CRISPR-Cas Systems in Agricultural and Nonagricultural Campylobacter coli and Campylobacter jejuni Isolates Correlates with Lack of Shared Environments
Source: Genome Biol Evol. 2015 Sep 2;7(9):2663–79. doi: 10.1093/gbe/evv174 (PMC4607530; doi:10.1093/gbe/evv174)
Supplement: Supplementary Data [file supp_7_9_2663__index.html]

Differential distribution of Type II CRISPR-Cas systems in agricultural and non-agricultural Campylobacter coli and Campylobacter jejuni isolates correlates with lack of shared environments — Differential Distribution of Type II CRISPR-Cas Systems in Agricultural and Nonagricultural Campylobacter coli and Campylobacter jejuni Isolates Correlates with Lack of Shared Environments — Supplementary Data 

# Differential Distribution of Type II CRISPR-Cas Systems in Agricultural and Nonagricultural *Campylobacter coli* and *Campylobacter jejuni* Isolates Correlates with Lack of Shared Environments

## Supplementary Data

files

- Supplementary Data - pdf file
