# Supplementary material for: Differential Distribution of Type II CRISPR-Cas Systems in Agricultural and Nonagricultural Campylobacter coli and Campylobacter jejuni Isolates Correlates with Lack of Shared Environments
Source: Genome Biol Evol. 2015 Sep 2;7(9):2663–79. doi: 10.1093/gbe/evv174 (PMC4607530; doi:10.1093/gbe/evv174)
Supplement: Supplementary Data [file supp_evv174_GBE-150210_R1_Supplement_Figures_S1-S4_Tables_S1-S8.pdf]

SUPPLEMENTARY INFORMATION FOR:

Differential distribution of Type II CRISPR-Cas systems in agricultural and non-agricultural *Campylobacter coli* and *Campylobacter jejuni* isolates correlates with lack of shared environments

Bruce M. Pearson, Rogier Louwen, Peter van Baarlen, Arnoud H.M. van Vliet

CONTENTS

Supplementary Figures

- **Figure S1.** Annotated phylogenetic trees as used for Figure 4. Trees are based on alignments of A) 16S rDNA; B) Cas9 amino acid sequences; C) Cas1 amino acid sequences; D) Cas2 amino acid sequences; E) CRISPR-repeat nucleotide sequence; F) Csn2 amino acid sequences.
- **Figure S2.** Schematic representation of the two CRISPR-Cas systems found in *C. jejuni* and *C. coli* Clades 1, 2 and 3.
- **Figure S3.** *C. jejuni* and *C. coli* have two distinct CRISPR-Cas systems. (A) Phylogenetic tree (B) Alignment of *C. jejuni* and *C. coli* Cas9, Cas1 and Cas2 proteins.
- **Figure S4.** Analysis of transcription of the *C. jejuni* NCTC 11168 CRISPR array.

Supplementary Tables

- **Table S1.** Overview of characteristics of Type II-A and II-C CRISPR-Cas systems in 132 bacterial species.
- **Table S2.** Oligonucleotide primers used in this study.
- **Table S3.** Overview of CRISPR-status of *C. jejuni* and *C. coli* genomes used in this study.
- **Table S4.** EMBL/Genbank/DDBJ and Campylobacter PubMLST accession numbers of *Campylobacter* bacteriophage genomes, plasmid sequences and insertion elements/prophages included in this study.
- **Table S5.** Sequence of the 1,065 *Campylobacter* CRISPR spacer alleles used in this study
- **Table S6.** Phage, plasmid and genomic insertion element targeted by *C. jejuni* and *C. coli* CRISPR spacer sequences
- **Table S7.** Diversity of CRISPR spacer arrays in 1,919 *C. jejuni* and 23 *C. coli* genome sequences.
- **Table S8.** Distribution of CRISPR spacer alleles in *C. jejuni* MLST-clonal complexes.

16S rDNA

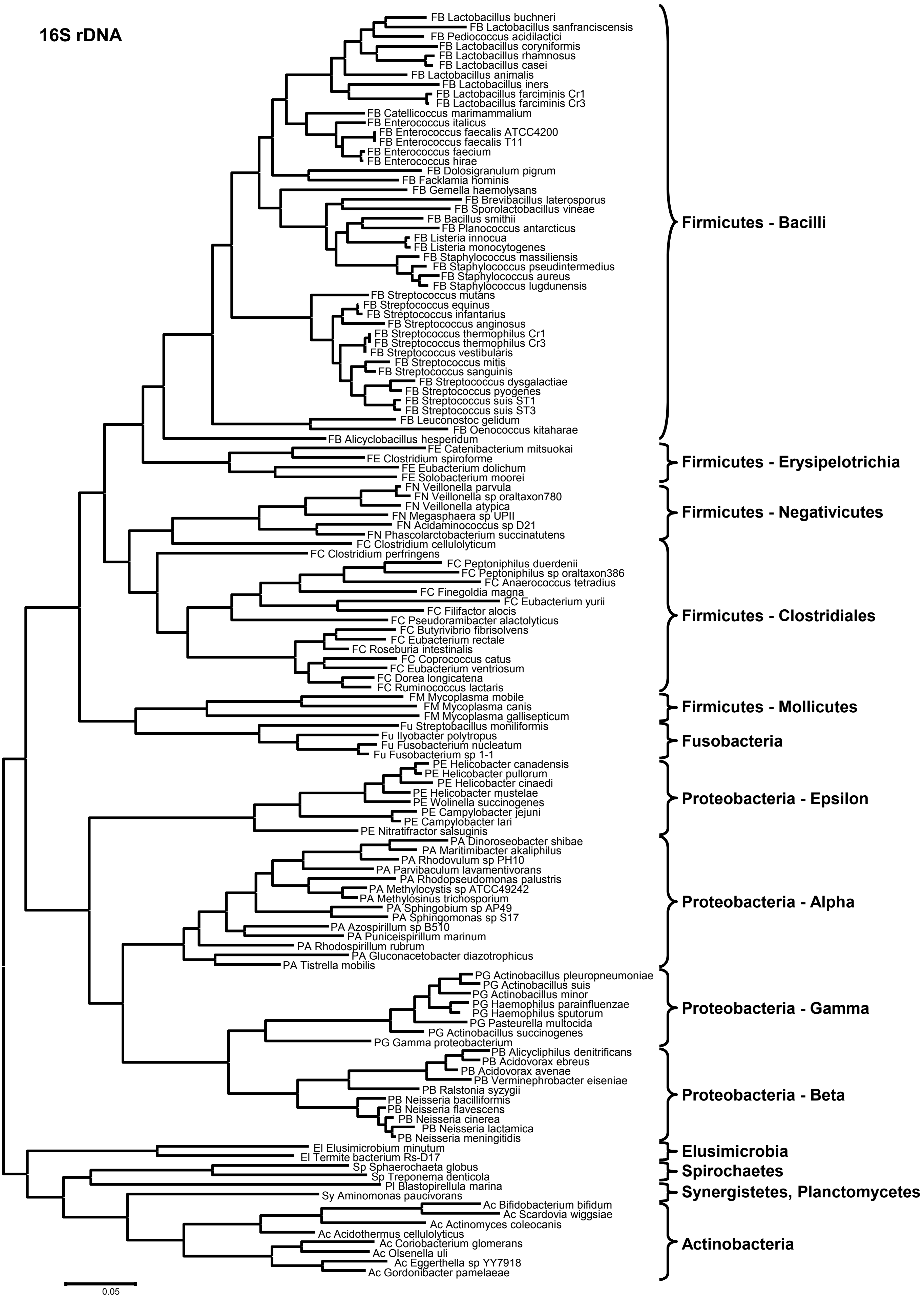

Pearson et al, Figure S1a

Cas9

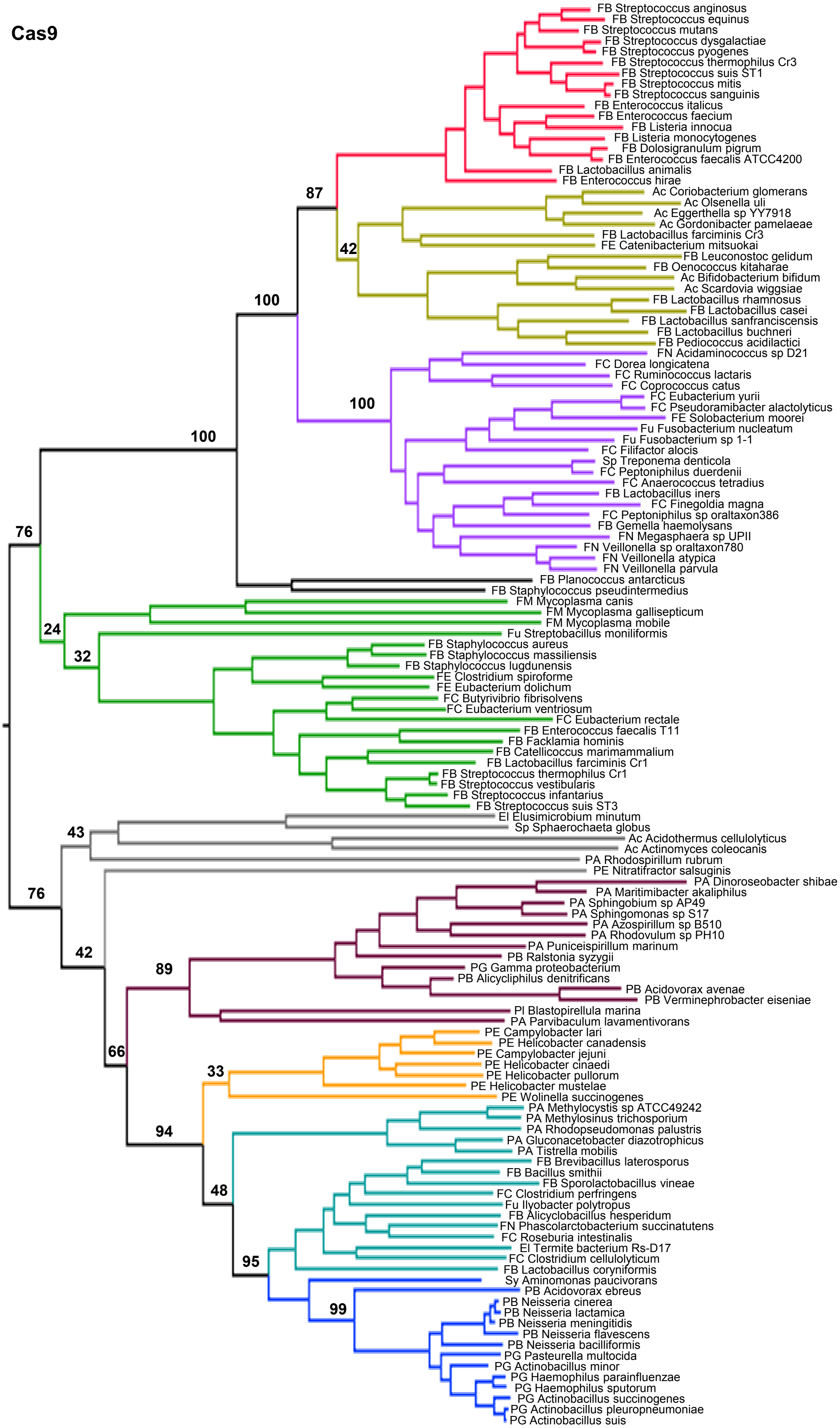

Pearson et al, Figure S1b

Cas1

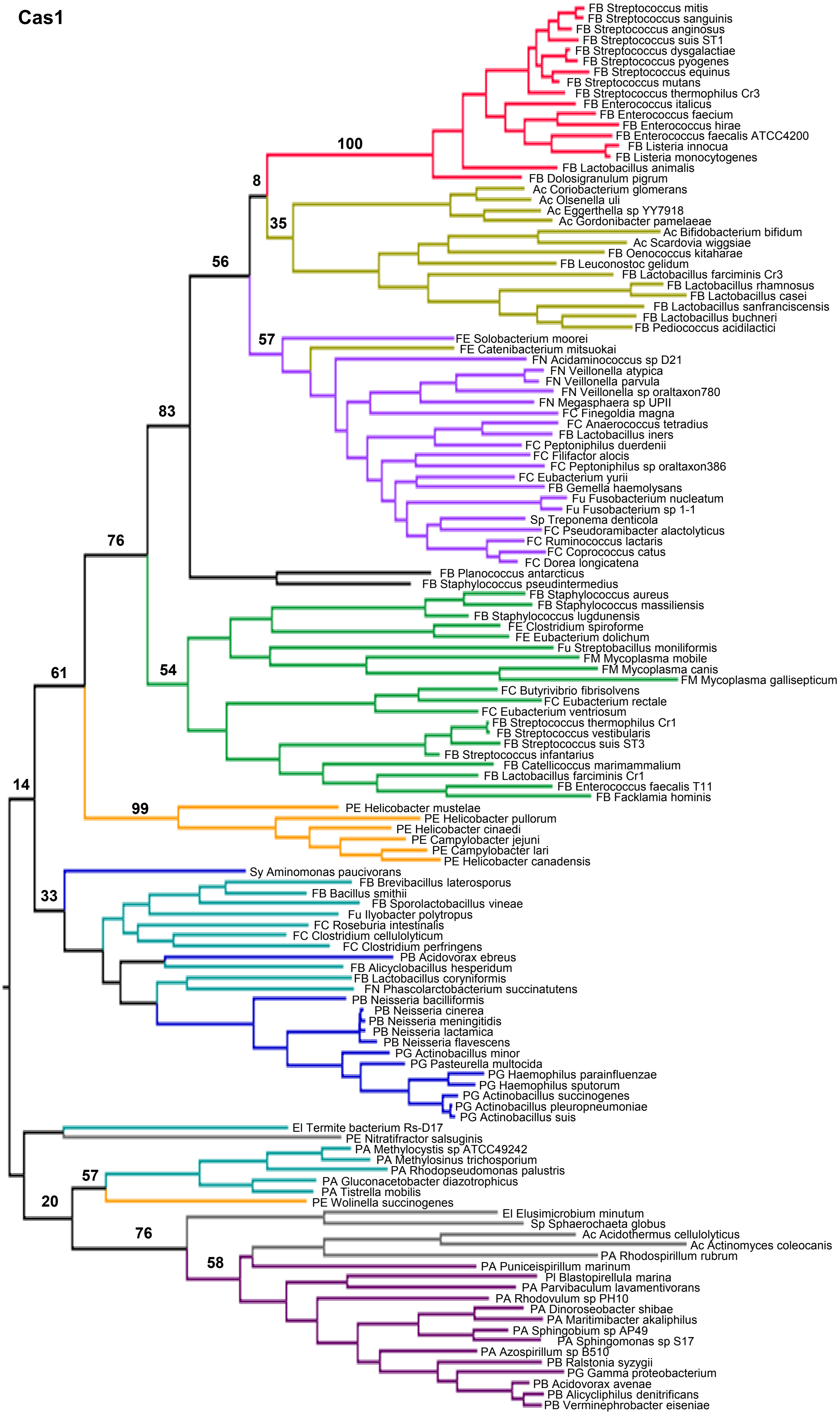

Pearson et al, Figure S1c

Cas2

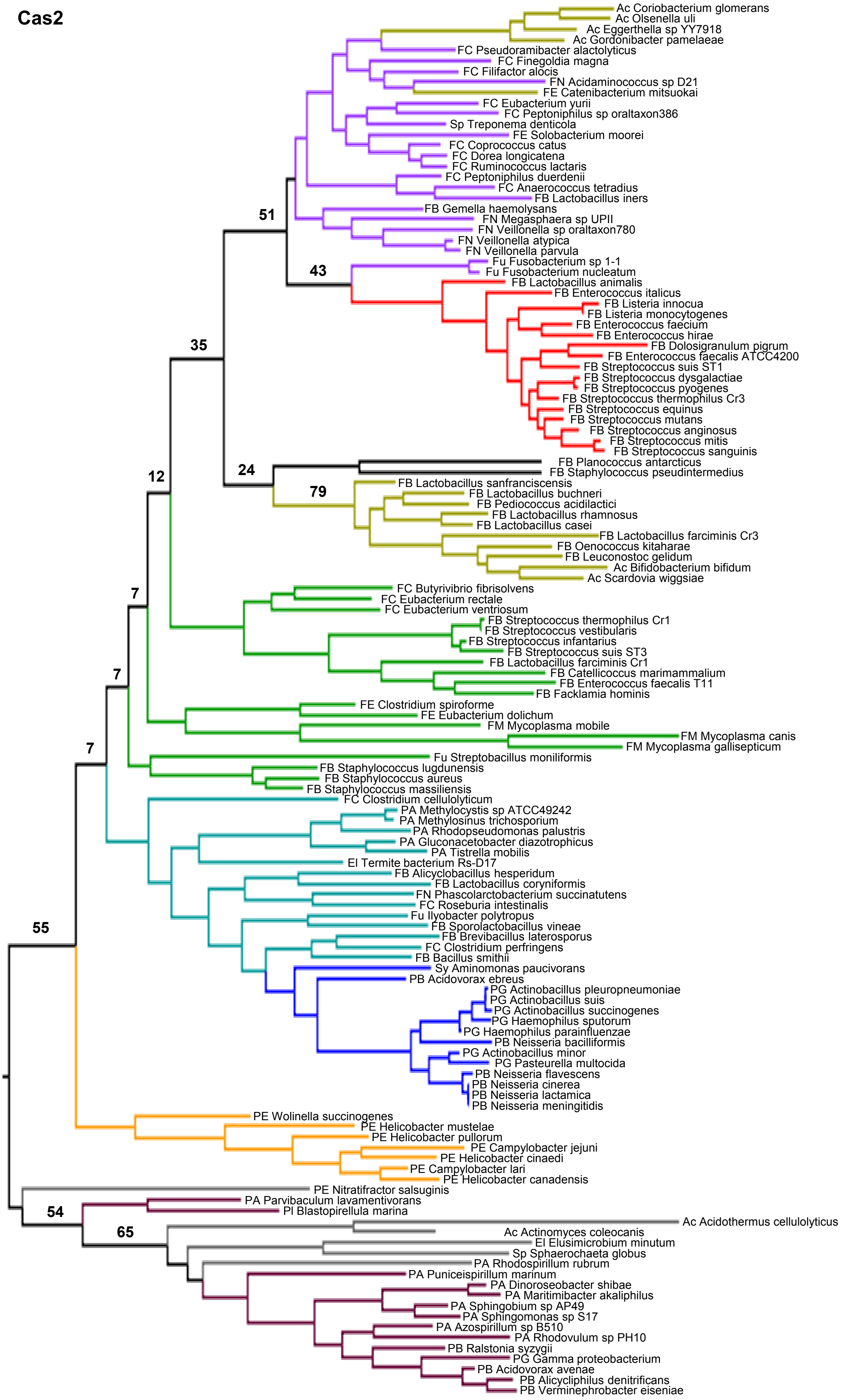

Pearson et al, Figure S1d

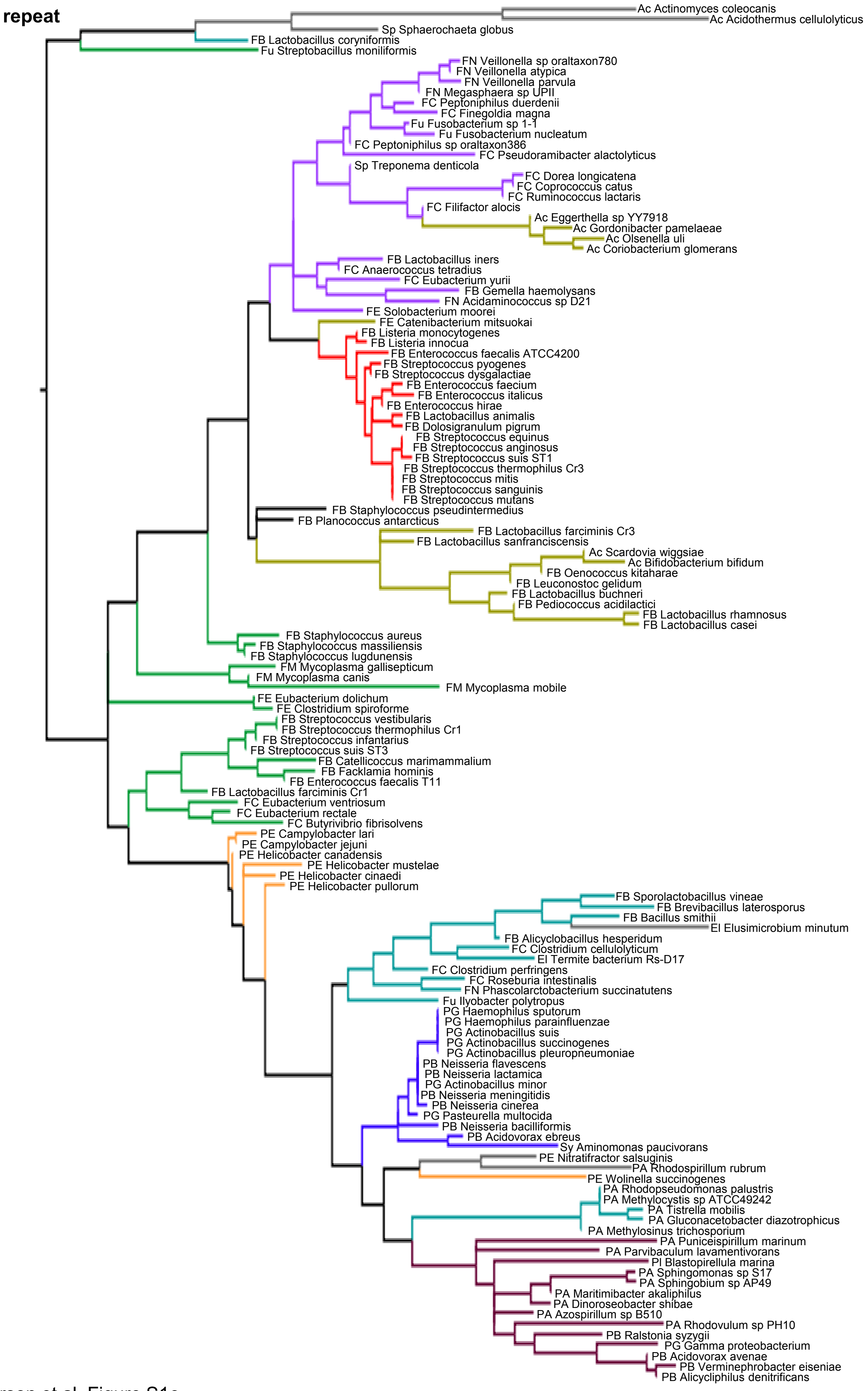

Pearson et al, Figure S1e

Csn2

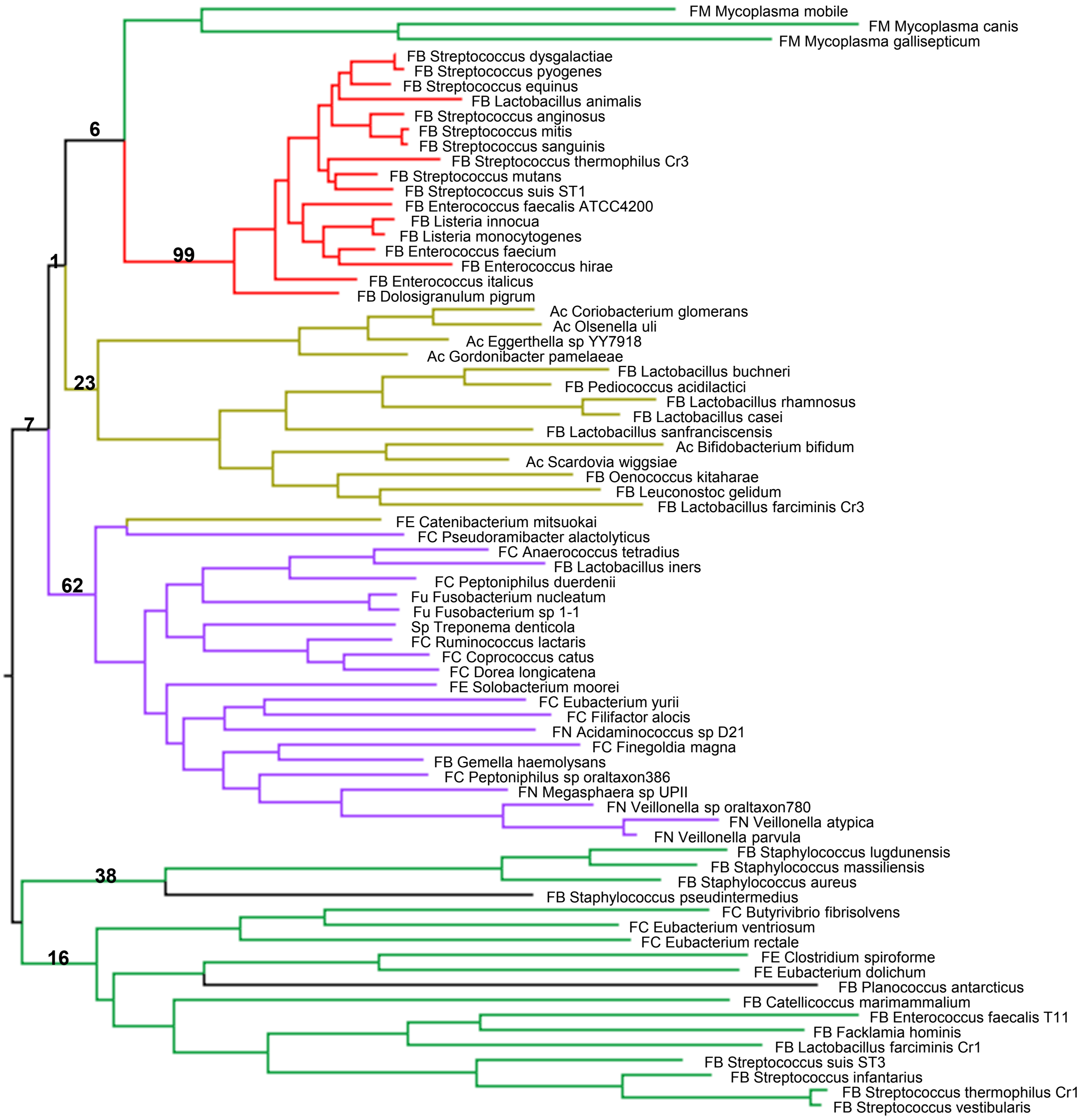

Figure S1. Annotated phylogenetic trees as used for Figure 4. Trees are based on alignments of A) 16S rDNA; B) Cas9 amino acid sequences; C) Cas1 amino acid sequences; D) Cas2 amino acid sequences; E) CRISPR-repeat nucleotide sequence; F) Csn2 amino acid sequences. Values given for Cas-genes represent percentage bootstrap values for 500 replicates. Codes before species names indicate phylogenetic group: Ac, Actinobacteria; FB, Firmicutes - Bacilli, FE; Firmicutes - Erysipelotrichia; FN, Firmicutes - Negativicutes; FC, Firmicutes - Clostridiales; FM, Firmicutes - Mollicutes Fusobacteria; PE, Proteobacteria - Epsilon; PA, Proteobacteria - Alpha; PB, Proteobacteria - Beta; PG, Proteobacteria - Gamma; Sy, Synergistetes; Pl, Planctomycetes; Sp. Spirochaetes; El, Elusimicrobia.

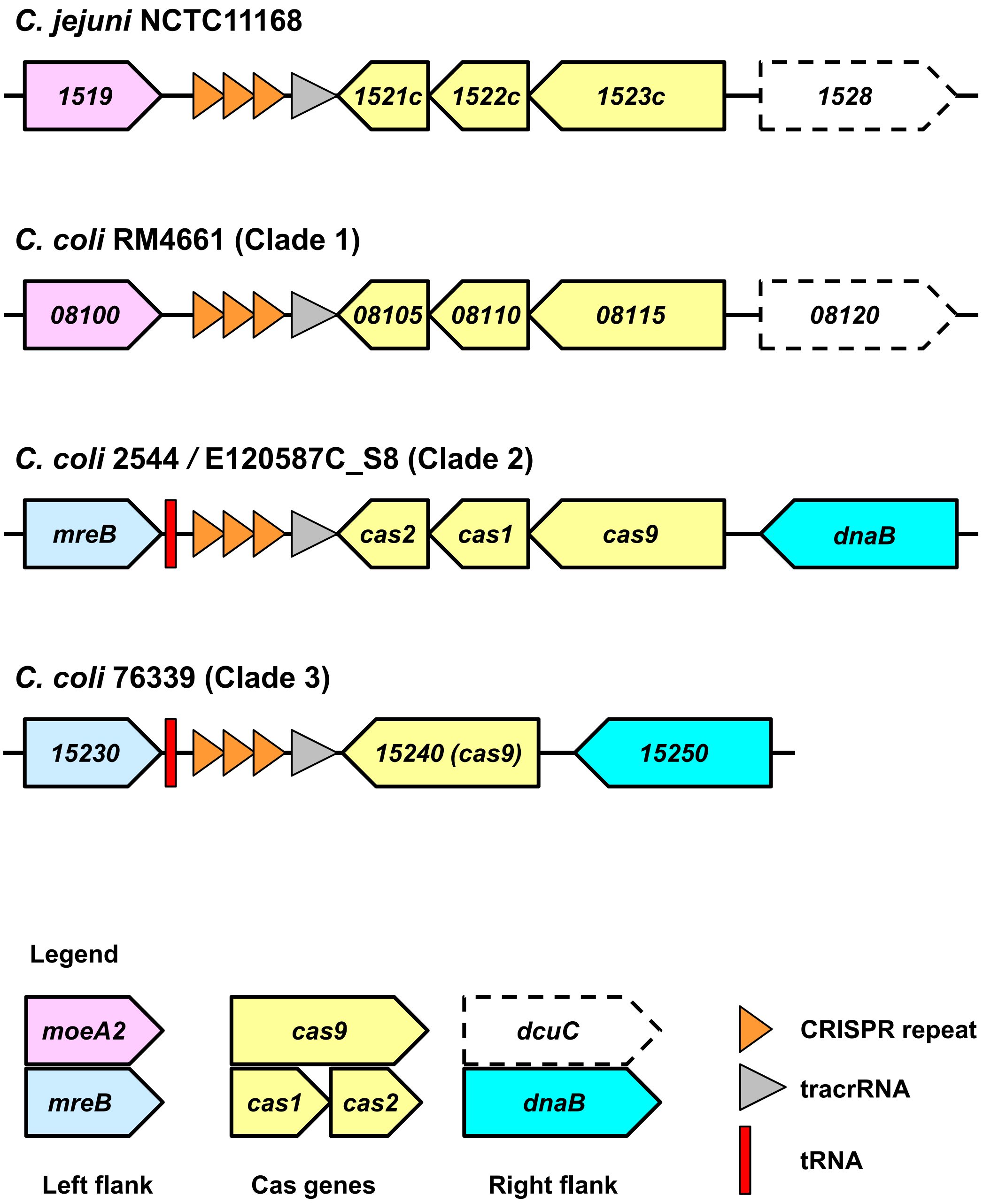

Figure S2. Schematic representation of the two CRISPR-Cas systems found in *C. jejuni* and *C. coli* Clades 1, 2 and 3. Flanking genes and gene numbers of genomes are included where available; there is no annotated *C. coli* Clade 2 genome publicly available and hence gene numbers cannot be included. Figure S2. Schematic representation of the two CRISPR-Cas systems found in *C. jejuni* and *C. coli* Clades 1, 2 and 3. Flanking genes and gene numbers of genomes are included where available; there is no annotated *C. coli* Clade 2 genome publicly available and hence gene numbers cannot be included.

Figure S3

(A)

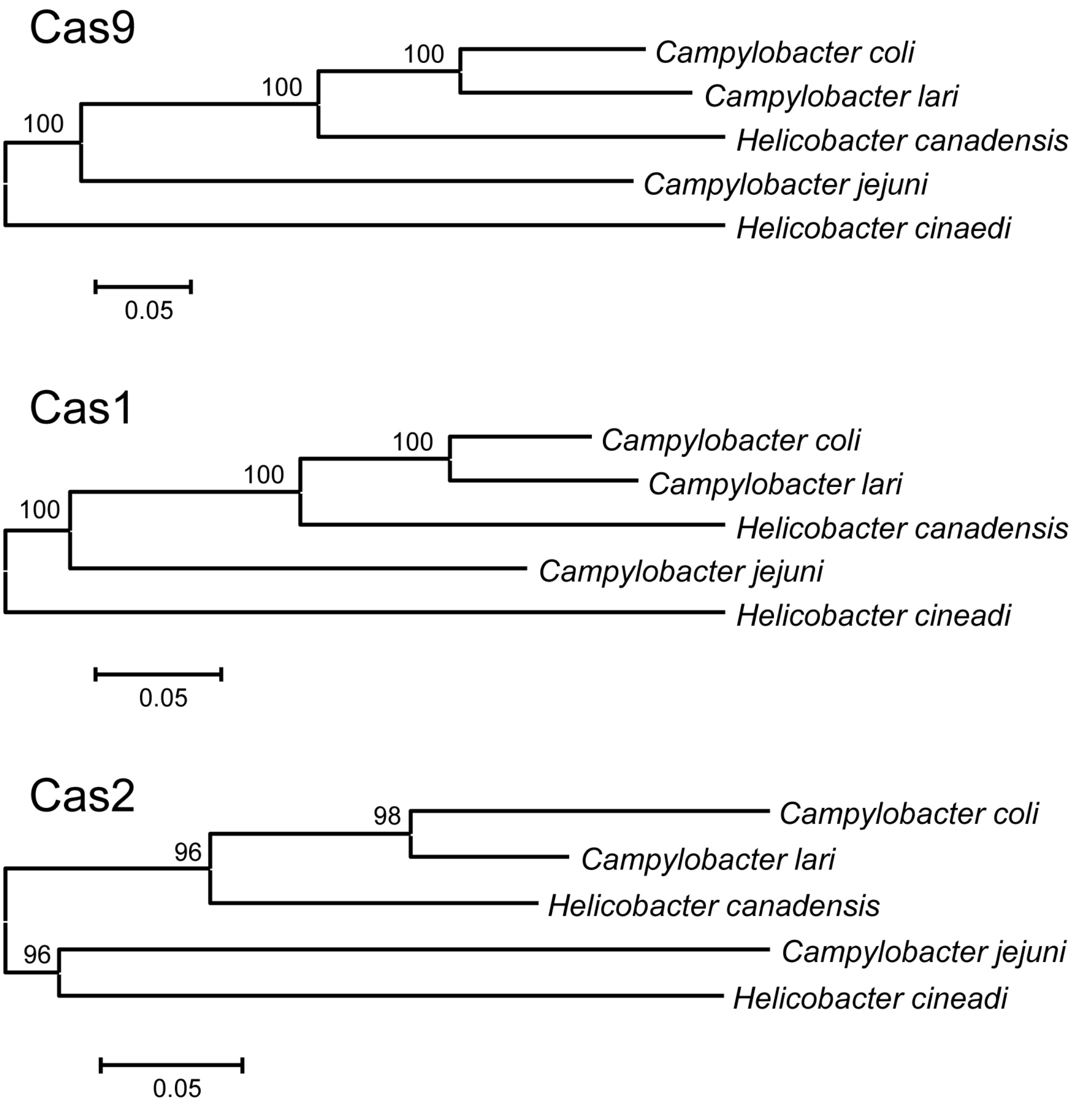

(B)

```
                        RuvC I
C. jejuni Cas9    ARILAFDIGISSIGWAFSENDELKDCGVRIFTKVENPKTGESLALPRRL
C. coli Cas9      MKILGFDIGINSIGWAFVEDNQLQDCGVRLFTKAEDPKTKESLALPRRN
                   :**.*****.****** *:::*:*****:***.*:*** ********

                  R-rich region
C. jejuni Cas9    RSARKRLARRKARLNHLKHLIANEFKLNYEDYQSFDESLAKAYKGSLIS
C. coli Cas9      RSNRRRLGRRRSRLIALKHIISKGLKLNYQDYIANDGELPKAYEGRLIS
                  ** *:**.**::**  ***:*:: :****:** : * .*.***:* ***

C. jejuni Cas9    YELRFRALNELLSKQDFARVILHIAKRRGY--DDIKNSDDKEKGAILKA
C. coli Cas9      YELRYKALNEKIEPKDLARVILHIAKHRGYMNKNEKKSSDNEKGKILSA
                  ****::**** :. :*:*********:***  .: *:*.*:*** **.*

C. jejuni Cas9    KQNEEKLANYQSVGEYLYKEYFQKFKENSKEFTNVRNKKESYERCIAQS
C. coli Cas9      KTNALKLEKYQSVGEYFYKEFFQKYRENTKDFINIRNKEGSYENCVLAS
                  * *  ** :*******:***:***::**:*:* *:***: ***.*:  *
```

```
C. jejuni Cas9   LKDELKLIFKKQREFGFSFSKKFEEEVLSVAFYKRALKDFSHLVGNCSF
C. coli Cas9     LEKELKLILEKQKEWGYSYNDNFIKEILKVAFFQRPLKDFSYLVGACTF
                 *:.*****::**:*:*:*:..:* :*:*.***::*.*****:*** *:*

C. jejuni Cas9   TDEKRAPKNSPLAFMFVALTRIINLLNNLKNTEGILYTKDDLNALLNEV
C. coli Cas9     EDEKRACKNSYSAWEFVALTKIINELKSLEKESGELVSSQIINEILNHI
                  ***** ***  *: *****:*** *:.*:: .* * :.: :* :**.:

C. jejuni Cas9   KNGTLTYKQTKKLLGLSDDYEFKGEK-------GTYFIEFKKYKEFIKA
C. coli Cas9     DKGSITYKKFREYIKLHESMKFKSLKYDKDNVESTKLIEFRKLVEFKKA
                 .:*::***: :: : * :. :**. *       .* :***:*  ** **

C. jejuni Cas9   GEHNLSQDDLNEIAKDITLIKDEIKLKKALAKYDLNQNQIDSLSKLEFK
C. coli Cas9     GEHSLSREELDQIATYITLIKDNEKLKITLEKYSLNNEQIKNLLEIDFN
                 ***.**:::*::**. ******: *** :* **.**::**..* :::*:

C. jejuni Cas9   HLNISFKALKLVTPLMLEGKKYDEACNELNLKVAINEDKKDFLPAFNET
C. coli Cas9     HINLSFKALNLILPLMKEGKRYDEACKLLNLKTKSNNQKFDFLPAFCDS
                 *:*:*****:*: *** ***:*****: ****.  *::* ****** ::

                                                  RuvC II
C. jejuni Cas9   YKDEVTNPVVLRAIKEYRKVLNALLKKYGKVHKINIELAREVGKNHSQR
C. coli Cas9     FAQELTNPIVNRAISEYRKVLNALLKKYGKMHKIHIELARDIGLSKKLR
                 : :*:***:* ***.***************:***:*****::* .:. *

C. jejuni Cas9   KIEKEQNENYKAKKDAELECEKLGLKINSKNILKLRLFKEQKEFCAYSG
C. coli Cas9     KIEKEQKENYENNIWALNECEKFGLKANAKNILKLKLWKEQKEFCIYSG
                 ******:***: :  *  ****:*** *:******:*:******* ***

                                    HNH domain
C. jejuni Cas9   KIKISDLQDEKMLEIDHIYPYSRSFDDSYMNKVLVFTKQNQEKLNQTPF
C. coli Cas9     KISIEHLRDEKALEVDHIYPYSRSFDDSFLNKVLVFTKENQEKLNKTPF
                 **.*..*:*** **:*************::********:******:***

C. jejuni Cas9   AFGNDSAKWQKIEVLAKNLPTKKQKRILDKNYKDKEQKNFKDRNLNDTR
C. coli Cas9     AFGANEERWSKIQALAQNLPYKKKNKILDEAFKGKQQQDFISRNLNDTR
                 *** :. :*.**:.**:*** **:::***: :*.*:*::* .*******

C. jejuni Cas9   IARLVLNYTKDYLDFLPLSDDENTKLNDTQKGSKVHVEAKSGMLTSALR
C. coli Cas9     ISTLIVKYTKEYLDFLPLDEKEDVNLKSGEKGSKIHVQTINGMLTSVLR
                 *: *:::***:*******.:.*:..*:. :****:**:: .*****.**

                              RuvC III
C. jejuni Cas9   TWGFSAKDRNNHLHHAIDAVIIAYANNSIVKAFSDFKKEQESNSAELYA
C. coli Cas9     TWGFAQKDRNNHLHHALDATIVAYSTNAIIKAFSDFKKEQELLKAKLYA
                 ****: **********:**.*:**:.*:*:***********  .*:***

C. jejuni Cas9   KISELDYKNKRKFFEPFSGFRQKVLDKIDEIFVSKPERKKPSGALHEET
C. coli Cas9     ELTSDSYKHQAKFFEPFEGFREQILNQINKLFVSKPPRKRARGALHKET
                 :::. .**:: ******.***:::*::*:::***** **:. ****:**

C. jejuni Cas9   RKEEEFYQSYGGKEGVLKALELGKIRKVNGKIVKNGDMFRVDIFKHKKT
C. coli Cas9     YSKDEMIKKYNSQEGVEIALNCGKIRKIGTKYVENDTMVRIDIF--KKQ
                  .::*: :.*..:***  **: *****:. * *:*. *.*:***  **

C. jejuni Cas9   KFYAVPIYTMDFALKVLPNKAV--ARSKKGEIKDWILMDENYEFCFSLY
C. coli Cas9     KFYAIPIYTMDFALGVLPNKIVIIGKDKKGNPKQWQEIDESYEFCFSLH
                 ****:********* ***** *  .:.***: *:*  :**.*******:

C. jejuni Cas9   DSLILIQTKDMQEPEFVYYNAFTSSTVSLIVSKHDNKFETLSKNQKILF
C. coli Cas9     DDLVLIQKKDMQEPEFAYYNGFDISNSSICVEKHDNKFENLTDNQRLLF
                 *.*:***.********.***.*  *. *: *.*******.*:.**::**
```

Pearson et al, Figure S3

```
C. jejuni Cas9   NANEKEVIAKSIGIQNLKVFEKYIVSALGEVTKAEFRQREDFKK-----
C. coli Cas9     NA-EGNVKAKKIGIQGLRIFEKYIITPLGEKIKADFKPREDIALKTSKK
                 ** * :* **.****.*::*****::.***  **:*: ***:

C. jejuni Cas9   --
C. coli Cas9     GL
```

-+-+-+-+-+-+-+-+-+-+-+-+-+-+-

```
C. jejuni Cas1   MSYDEAFKTLLISSNAKLNLELNHLVIKQDENIAKLFLKDINIIVLESL
C. coli Cas1     MAYDEAFKSILISNQAKLSLEFNHLVIKQDDKEAKFFLKDINFIILESL
                 *:******::***.:***.**:********:: **:******:*:****

C. jejuni Cas1   QISISSALFNAFAKYKIILLTCDETHSINGVFTPFLGHFQSAKIAKEQM
C. coli Cas1     QASITSSLLSALAKYKIILLTCDESHHINGIFSPFLGHFISAKVAKKQI
                 * **:*:*:.*:***********:* ***:*:****** ***:**:*:

C. jejuni Cas1   NVSAQKKAILWQKIIKNKILNQAFILKKHNKIEQSNELINLAKKVSLND
C. coli Cas1     KVTLQRKAILWQKIIKNKISNQAHILKITHHIKEAKELLALAKNVSLND
                 :*: *:************ ***.***  ::*::::**: ***:*****

C. jejuni Cas1   SKNIEAVAAALYFKTLFGTSFSRDELCFENSALNYGYAIIRACIIRAVC
C. coli Cas1     AKNLEATAAALYFKTLFGSNFTRNEICFANSALNYGYAVIRSLIVRAVC
                 :**:**.***********:.*:*:*:** *********:**: *:****

C. jejuni Cas1   ISGLLPWLGIKHDNIYNSFALCDDLIEVFRASVDDCVLKLKG--ESEFL
C. coli Cas1     ISGLLPWQGIKHDNIYNDFNLCDDLIEVFRPLVDLCVFDLKDIKESEFL
                 ******* *********.* **********. ** **:.**.  *****

C. jejuni Cas1   SKDDKRALIGNLQSKINFDGQNYPLNRAINHYVANFKNALLYEDELKIV
C. coli Cas1     DKEAKQRLIGILQEEIIIENKAYPLNRAINFYIQNFKNALLENDELMMV
                 .*: *: *** **.:* ::.: ********.*: ******* :*** :*

C. jejuni Cas1   SKFDD--
C. coli Cas1     DNLYDRR
                 .:: *
```

-+-+-+-+-+-+-+-+-+-+-+-+-+-+-

```
C. jejuni Cas2   MIEDKFMRVLLMFDVPTKSKKEQKLASKFRNNLIKLGYFMLQFSVYMRI
C. coli Cas2     MIEDKFMRVLLMFDIPTKSKKEQKSANKFRVSLVKLGFFMMQFSVYVKI
                 **************:********* *.*** .*:***:**:*****::*

C. jejuni Cas2   CKGLSSAKSSIENVKKILPPYGNVRALIITEKQFDKMELLLGGIVFNEK
C. coli Cas2     CKGMTSAKTAIKSVERILPPCGNIRVLTITEKQFDNMQILLGGVSFNEK
                 ***::***::*:.*::**** **:*.* *******:*::****: ****

C. jejuni Cas2   VNNETNLTLFDIDSHGEFKYKNSNNEEIQINK-KQEKYHQQNLFEF
C. coli Cas2     MNDDKNLVLFEYDEKTQ-DFKYSNDKIYKENKTKSNKIRQASLFEF
                 :*::.**.**: *.: : .:* **::  : ** *.:* :* .****
```

Figure S3. *C. jejuni* and *C. coli* have two distinct CRISPR-Cas systems. (A) Phylogenetic tree of *C. jejuni* and *C. coli* Cas9, Cas1 and Cas2 compared to corresponding Cas-proteins of other Epsilon Proteobacteria. Numbers at nodes give percentage bootstrap values based on 500 iterations. (B) Alignment of *C. jejuni* and *C. coli* Cas9, Cas1 and Cas2 proteins. Asterisks indicate identical residues, full stops and colon indicate conservative substitutions. Important domains of Cas9 are indicated in yellow shading.

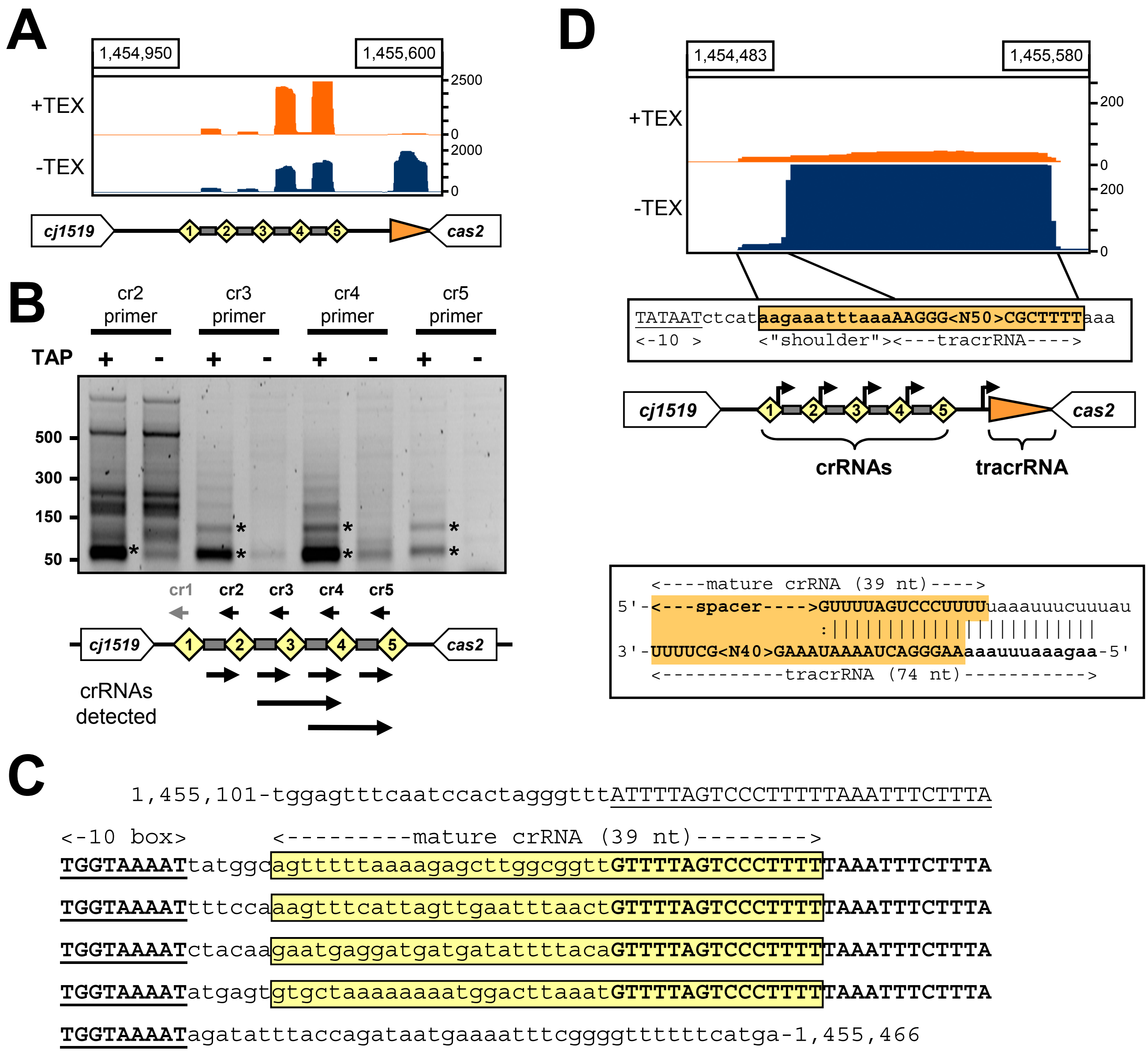

Figure S4. Analysis of transcription of the *C. jejuni* NCTC 11168 CRISPR array. (A) Differential RNA-sequencing analysis of the *C. jejuni* CRISPR region. The red histograms represent the reads from the cDNA library enriched for primary transcripts (+TEX), and the blue histograms representing the non-enriched -TEX cDNA library (Porcelli et al, 2013). A representation of the genomic region is given below the histograms, the nucleotide positions above the histograms. (B) 5' RACE analysis of CRISPR transcription. The resulting PCR products were run on an agarose gel and stained with ethidium bromide. Marker sizes are given to the left, the specific primer used for reverse transcription is named above the + and - TAP lanes. No products were obtained with the CR1 primer, consistent with the lack of a leader transcript. Asterisks indicate TAP-enriched transcripts (crRNAs), and their position is indicated below the gel picture. (C) Functional annotation of the *C. jejuni* CRISPR-region, with mature transcripts, repeat sequence, transcript start sites and σ^{70} promoters (-10 box) highlighted. (D) Transcription profile and characteristics of the *C. jejuni* tracrRNA. The area of interaction between tracrRNA and CRISPR repeat is shown, and is represented in the shoulder (processed) part of the tracrRNA.

Table S1. Overview of characteristics of Type II-A and II-C CRISPR-Cas systems in bacteria

BLASTP searches of Genbank were used to identify orthologs of Cas9 of *Nitratifractor salsuginis, Staphylococcus pseudoepidermidus and Campylobacter jejuni*
Those systems containing orthologs of Cas1, Cas2 and potential Csn2 orthologs were selected for identification of CRISPR-repeat arrays using CRISPRfinder (Grissa et al, Nucl. Acids Res. (2007) 35 (suppl 2): W52-W57)
Only 36 nt CRISPR repeats and Cas9, Cas1 and Cas2-positive CRISPR systems were included. CRISPR repeats with a putative sigma70 -10 sequence (gnTAnaaT) have these indicated by **red bold typeface.**
Grouping into II-A and II-C is based on Chylinski et al, RNA Biology 10:5, 1–12 (2013), with II-A (1) and II-A (2) as defined in this paper. Orientation is in the relative direction to the Cas-genes.
Sources are as found in literature or internet. Blue indicates an environmental source (marine, plant, insects, water), black an association with mammalian GI-tract.

Number	species	Group	Class	Code	gene	aa cas9	MW	gene	aa cas1	MW	gene	aa cas2	MW	gene	aa csn2
001	Acidothermus cellulolyticus 11B	II-C	actinobacteria	Ac	*cas9*	1138	127.3	*cas1*	295	32.2	*cas1*	108	12.1	*n/a*	n/a
002	Actinomyces coleocanis DSM 15436	II-C	actinobacteria	Ac	*cas9*	1105	123.6	*cas1*	299	32.6	*cas1*	106	12.1	*n/a*	n/a
003	Bifidobacterium bifidum S17	II-A(1)	actinobacteria	Ac	*cas9*	1420	163.6	*cas1*	296	34.2	*cas1*	106	12.3	*csn2*	220
004	Coriobacterium glomerans PW2	II-A(1)	actinobacteria	Ac	*cas9*	1384	160.1	*cas1*	292	33.2	*cas1*	111	13.0	*csn2*	226
005	Eggerthella sp YY7918	II-A(1)	actinobacteria	Ac	*cas9*	1380	158.4	*cas1*	292	33.3	*cas1*	111	13.1	*csn2*	227
006	Gordonibacter pamelaeae 7-10-1-b	II-A(1)	actinobacteria	Ac	*cas9*	1371	155.5	*cas1*	292	33.2	*cas1*	111	12.9	*csn2*	226
007	Olsenella uli DSM7084	II-A(1)	actinobacteria	Ac	*cas9*	1399	161.1	*cas1*	292	33.0	*cas1*	111	13.0	*csn2*	226
008	Scardovia wiggsiae F0424	II-A(1)	actinobacteria	Ac	*cas9*	1471	168.5	*cas1*	288	33.1	*cas1*	106	12.4	*csn2*	222
009	Elusimicrobium minutum Pei191	II-C	elusimicrobia	EL	*cas9*	1195	137.3	*cas1*	298	33.9	*cas1*	108	13.0	*n/a*	n/a
010	Termite group 1 bacterium phylotype Rs-D17	II-C	elusimicrobia	EL	*cas9*	1032	120.1	*cas1*	298	33.8	*cas1*	109	13.2	*n/a*	n/a
011	Alicyclobacillus hesperidum URH17-3-68	II-C	firmicutes-bacilli	FB	*cas9*	1146	131.1	*cas1*	307	34.7	*cas1*	106	12.7	*n/a*	n/a
012	Bacillus smithii 7-3-47FAA	II-C	firmicutes-bacilli	FB	*cas9*	1088	127.0	*cas1*	299	34.4	*cas1*	106	12.6	*n/a*	n/a
013	Brevibacillus laterosporus GI-9	II-C	firmicutes-bacilli	FB	*cas9*	1092	127.4	*cas1*	299	34.8	*cas1*	106	12.6	*n/a*	n/a
014	Listeria innocua Clip11262	II-A(1)	firmicutes-bacilli	FB	*cas9*	1334	154.8	*cas1*	288	33.8	*cas1*	113	13.3	*csn2*	223
015	Listeria monocytogenes F6900	II-A(1)	firmicutes-bacilli	FB	*cas9*	1334	154.8	*cas1*	288	33.6	*cas1*	113	13.2	*csn2*	220
016	Planococcus antarcticus DSM14505	II-A(1)	firmicutes-bacilli	FB	*cas9*	1333	155.4	*cas1*	294	34.3	*cas1*	106	12.6	*csn2*	288
017	Sporolactobacillus vineae DSM21990	II-C	firmicutes-bacilli	FB	*cas9*	1084	126.6	*cas1*	300	34.7	*cas1*	106	12.5	*n/a*	n/a
018	Staphylococcus aureus M06-0171	II-A(2)	firmicutes-bacilli	FB	*cas9*	1053	124.0	*cas1*	301	35.4	*cas1*	107	12.6	*csn2*	329
019	Staphylococcus lugdunensis M23590	II-A(2)	firmicutes-bacilli	FB	*cas9*	1054	123.8	*cas1*	300	35.2	*cas1*	107	12.9	*csn2*	333
020	Staphylococcus massiliensis S46	II-A(2)	firmicutes-bacilli	FB	*cas9*	1052	122.8	*cas1*	301	35.3	*cas1*	107	12.8	*csn2*	330
021	Staphylococcus pseudintermedius ED99	II-A(1)	firmicutes-bacilli	FB	*cas9*	1334	157.1	*cas1*	291	34.0	*cas1*	105	12.1	*csn2*	282
022	Anaerococcus tetradius ATCC 35098	II-A(1)	firmicutes-clostridia	FC	*cas9*	1361	160.3	*cas1*	292	34.3	*cas1*	106	12.6	*csn2*	222
023	Butyrivibrio fibrisolvens 16/4	II-A(2)	firmicutes-clostridia	FC	*cas9*	1105	127.9	*cas1*	302	35.6	*cas1*	107	12.8	*csn2*	327
024	Clostridium cellulolyticum H10	II-C	firmicutes-clostridia	FC	*cas9*	1021	118.6	*cas1*	298	33.6	*cas1*	115	13.5	*n/a*	n/a
025	Clostridium perfringens C str. JGS1495	II-C	firmicutes-clostridia	FC	*cas9*	1065	124.6	*cas1*	299	33.9	*cas1*	107	12.8	*n/a*	n/a
026	Coprococcus catus GD/7	II-A(1)	firmicutes-clostridia	FC	*cas9*	1338	155.1	*cas1*	290	33.5	*cas1*	106	12.3	*csn2*	223
027	Dorea longicatena DSM 13814	II-A(1)	firmicutes-clostridia	FC	*cas9*	1340	156.7	*cas1*	290	33.8	*cas1*	106	12.3	*csn2*	223
028	Eubacterium rectale ATCC 33656	II-A(2)	firmicutes-clostridia	FC	*cas9*	1104	128.9	*cas1*	302	35.0	*cas1*	108	13.0	*csn2*	327
029	Eubacterium ventriosum ATCC 27560	II-A(2)	firmicutes-clostridia	FC	*cas9*	1107	130.9	*cas1*	305	35.6	*cas1*	108	12.8	*csn2*	331
030	Eubacterium yurii subsp margaretiae ATCC43715	II-A(1)	firmicutes-clostridia	FC	*cas9*	1391	162.5	*cas1*	290	33.6	*cas1*	108	12.6	*csn2*	223
031	Filifactor alocis ATCC35896	II-A(1)	firmicutes-clostridia	FC	*cas9*	1365	160.8	*cas1*	292	34.2	*cas1*	106	12.6	*csn2*	222
032	Finegoldia magna SY403409CC001050417	II-A(1)	firmicutes-clostridia	FC	*cas9*	1348	158.8	*cas1*	290	33.7	*cas1*	106	12.5	*csn2*	215
033	Peptoniphilus duerdenii ATCC BAA-1640	II-A(1)	firmicutes-clostridia	FC	*cas9*	1364	159.9	*cas1*	292	33.9	*cas1*	106	12.7	*csn2*	222
034	Peptoniphilus sp oral taxon 386 str F0131	II-A(1)	firmicutes-clostridia	FC	*cas9*	1380	162.1	*cas1*	291	34.0	*cas1*	106	12.5	*csn2*	226
035	Pseudoramibacter alactolyticus ATCC 23263	II-A(1)	firmicutes-clostridia	FC	*cas9*	1348	155.6	*cas1*	299	34.5	*cas1*	106	12.2	*csn2*	225
036	Roseburia intestinalis L1-82	II-C	firmicutes-clostridia	FC	*cas9*	1124	129.9	*cas1*	301	34.3	*cas1*	106	12.4	*n/a*	n/a
037	Ruminococcus lactaris ATCC 29176	II-A(1)	firmicutes-clostridia	FC	*cas9*	1341	156.2	*cas1*	290	33.4	*cas1*	106	12.3	*csn2*	222
038	Catenibacterium mitsuokai DSM 15897	II-A(1)	firmicutes-erysipelotrichia	FE	*cas9*	1329	156.2	*cas1*	287	33.4	*cas1*	106	12.5	*csn2*	234
039	Clostridium spiroforme DSM1552	II-A(2)	firmicutes-erysipelotrichia	FE	*cas9*	1105	128.5	*cas1*	297	35.1	*cas1*	107	12.8	*csn2*	281
040	Eubacterium dolichum DSM3991	II-A(2)	firmicutes-erysipelotrichia	FE	*cas9*	1091	126.4	*cas1*	300	34.8	*cas1*	107	13.0	*csn2*	287
041	Solobacterium moorei F0204	II-A(1)	firmicutes-erysipelotrichia	FE	*cas9*	1323	154.7	*cas1*	302	34.8	*cas1*	106	12.2	*csn2*	219
042	Catellicoccus marimammalium M35 04 3	II-A(2)	firmicutes-bacilli	FB	*cas9*	1140	133.7	*cas1*	304	35.8	*cas1*	107	13.1	*csn2*	308
043	Dolosigranulum pigrum ATCC51524	II-A(1)	firmicutes-bacilli	FB	*cas9*	1332	156.1	*cas1*	289	33.9	*cas1*	105	12.2	*csn2*	225
044	Enterococcus faecalis ATCC 4200	II-A(1)	firmicutes-bacilli	FB	*cas9*	1337	155.3	*cas1*	288	33.5	*cas1*	109	13.0	*csn2*	219
045	Enterococcus faecalis T11	II-A(2)	firmicutes-bacilli	FB	*cas9*	1150	134.0	*cas1*	304	35.0	*cas1*	107	12.7	*csn2*	352

MW	CRISPR repeat sequence (5' -> 3')	orientation to cas genes	no. of spacers	Source	Accession number genome	GC% genome	GC% spacer	species	group	phylogenetic Cas9 cluster
n/a	GCTGGGGAGCCTGTCTCAATCCCCCG**GCTAAAAT**GG	antisense	23	acidic hot springs	CP000481	66.9	61.1	Acidothermus cellulolyticus 11B	II-C	5
n/a	GTTGGGGATGACCGCTGATTTTT**GTTAAGAT**TGACC	antisense	39	vagina of dog	NZ_DS999543	49.6	36.1	Actinomyces coleocanis DSM 15436	II-C	5
24.4	GTTTCAGATGCCTGTCAGATCAATGACTTTGACCAC	sense	44	intestine	NC_014616	62.8	44.4	Bifidobacterium bifidum S17	II-A(1)	2
26.1	GTTTTGGAGCAGTGTCGTTCTGACTGGTAATCCAAC	sense	9	gut of firebug	NC_015389	60.4	47.2	Coriobacterium glomerans PW2	II-A(1)	2
26.6	GTTTTGGAGCAGTGGAATTCTGACTGGTAGTCAAAC	sense	43	human colon	AP012211	56.2	44.4	Eggerthella sp YY7918	II-A(1)	2
26.4	GTTTTGGAGCAGTGACAAACTGACTGGTAGTCAAAC	sense	17	human gut	FP929047	64.0	44.4	Gordonibacter pamelaeae 7-10-1-b	II-A(1)	2
26.0	GTTTTGGGGCAGTGTCGTTTCGACTGGTAATCAAAC	sense	30	sheep rumen, pig intestine	CP002106	64.7	47.2	Olsenella uli DSM7084	II-A(1)	2
25.8	GTTTCAGATGTCTGTCAGATCAATGAGGTAGACCAC	sense	unknown	oral cavity, dental caries	AGZS01000003	52.9	44.4	Scardovia wiggsiae F0424	II-A(1)	2
n/a	GTTAGGGTTGCCCTCCGAGAATTGATTTTA**TAGAAT**	antisense	13	insect intestinal tract	NC_010644	40.0	38.9	Elusimicrobium minutum Pei191	II-C	5
n/a	GTTATAGTTTCCTTCCTCTCTCAGATGT**GCTATAAT**	sense	21	symbiont of termite gut	NS_000191	35.2	36.1	Termite group 1 bacterium phylotype Rs-D17	II-C	8a
n/a	GTCATAGTTCCCTCACAAGCCTCGATGT**GGTATGAT**	antisense	53	soil	AKZN01000027	53.8	47.2	Alicyclobacillus hesperidum URH17-3-68	II-C	8a
n/a	GTCATAGTTCCCCTAAGATTATTGCTGT**GATATGAT**	antisense	27	food sources, milk, cheese	ACWF01000099	40.7	36.1	Bacillus smithii 7-3-47FAA	II-C	8a
n/a	GCTATAGTTCCTTACTAAAACTCGATAT**GATATGAT**	antisense	5	soil	CAGD01000017	40.8	30.6	Brevibacillus laterosporus GI-9	II-C	8a
26.0	GTTTTAGAGCTATGTTATTTTGAATGCTAACAAAAC	sense	10	soil, food sources	NC_003212	37.4	27.8	Listeria innocua Clip11262	II-A(1)	1
25.6	GTTTTAGAGCTATGTTATTTTGAATGCTACCAAAAC	sense	19	soil, food sources, infections	NZ_AARU02000001	37.7	30.6	Listeria monocytogenes F6900	II-A(1)	1
33.5	GTTTTAGACCAATGTAATTTTAGAGAGTAGTAAAAC	sense	4	lake in Antarctica	NZ_AJYB01000006	43.0	27.8	Planococcus antarcticus DSM14505	II-A(1)	N/A
n/a	GCCATACTTCCCCTGTAAAACTTGATGT**GATACGAT**	antisense	12	vineyard soil	NZ_BAEY01000027	49.5	41.7	Sporolactobacillus vineae DSM21990	II-C	8a
38.7	GTTTTAGTACTCTGTAATTTTAGGTATGAGGTAGAC	sense	12	skin, nasopharynx	HE980450	32.7	33.3	Staphylococcus aureus M06-0171	II-A(2)	4
39.5	GTTTTAGTACTCTGTAATTTTAGGTATAAGTGATAC	sense	2	skin	NZ_AEQA01000016	33.7	27.8	Staphylococcus lugdunensis M23590	II-A(2)	4
39.0	GTTTTAGTACTCTGTAATTTTAGGTATAAGTGATTC	sense	3	skin, brain abcess	NZ_AMSQ01000003	36.3	27.8	Staphylococcus massiliensis S46	II-A(2)	4
33.5	GTTTTAGCACTATGTTTATTTAGAAAGAGGTAAAAC	sense	23	necrotic tissue, skin	CP002478	37.6	27.8	Staphylococcus pseudintermedius ED99	II-A(1)	N/A
26.6	GTTTGAGAGTTATGTAATTTAAGTAGTAAGTAAAAC	sense	20	vagina, ovaries	NZ_GG666295	34.1	25.0	Anaerococcus tetradius ATCC 35098	II-A(1)	3
38.5	GTTTTAGTACCCGGGAAAATTAAGTGATTGGAAAAC	sense	22	GI tract of animals	FP929036	38.6	36.1	Butyrivibrio fibrisolvens 16/4	II-A(2)	4
n/a	GTTATAGCTCCAATTCAGGCTCCGATAT**GCTATAAT**	antisense	8	compost, intestine?	NC_011898	37.4	38.9	Clostridium cellulolyticum H10	II-C	8a
n/a	GTTATAGTTCCTAGTAAATTCTCGATAT**GCTATAAT**	antisense	46	vegetation, marine, GI tract	NZ_ABDU01000001	28.6	27.8	Clostridium perfringens C str. JGS1495	II-C	8a
26.6	GTTTGAGAATGATGTAAAAATGTATGGTACTCAAGC	sense	16	GI tract, faeces	FP929038	43.2	33.3	Coprococcus catus GD/7	II-A(1)	3
26.3	GTTTGAGAATGATGTAAAAATGTATGGTACACAAGC	sense	19	colonic microbiota	NZ_DS264384	41.4	33.3	Dorea longicatena DSM 13814	II-A(1)	3
38.5	ATTTTAGTAACTGAATAATTTACGTGACTGTAAAAC	sense	44	GI tract	NC_012781	41.5	25.0	Eubacterium rectale ATCC 33656	II-A(2)	4
39.4	ATTTTAGTACCTGAAGAAATTAAGTTATCGTAAAAC	sense	14	colonic microbiota	NZ_DS264262	34.9	25.0	Eubacterium ventriosum ATCC 27560	II-A(2)	4
26.2	GTTTGAGAACCTTGTAAATCAATAAGTATGTAAAAC	sense	16	subgingival dental plaque	NZ_AEES01000067	32.3	27.8	Eubacterium yurii subsp margaretiae ATCC43	II-A(1)	3
26.4	GTTTGAGAGTAGTGTAATTTCATATGGTAGTCAAAC	sense	26	peridontal pockets	CP002390	35.4	33.3	Filifactor alocis ATCC35896	II-A(1)	3
25.6	GTTTGAGAATGATGTAATTTCATATAGGTATTAAAC	sense	11	microbiota	AFUI01000017	32.0	25.0	Finegoldia magna SY403409CC001050417	II-A(1)	3
26.1	GTTTGAGAGTTATGTAATTTCATATAGGACTAAAAC	sense	29	commensal flora GI tract, oral	NZ_GL397071	34.2	27.8	Peptoniphilus duerdenii ATCC BAA-1640	II-A(1)	3
27.1	GTTTGAGAGCAATGTAATTTAAGATAGGTCTAAAAC	sense	9	commensal flora GI tract, oral	NZ_GL349422	30.9	30.6	Peptoniphilus sp oral taxon 386 str F0131	II-A(1)	3
26.5	GTTTGAGAACCTTGTTATTTACGATACTTCTTTAGC	sense	11	GI tract microbiota?	NZ_GL622359	51.6	33.3	Pseudoramibacter alactolyticus ATCC 23263	II-A(1)	3
n/a	GTTGTAATTCCCTGTTATCACTTGGTAT**GGTATAAT**	antisense	61	faeces, GI tract	NZ_GG692713	42.6	33.3	Roseburia intestinalis L1-82	II-C	8a
26.3	GTTTGAGAATGATGTAAAAATGTATGGTACTCAAAC	sense	18	rumen of herbivores	NZ_DS990163	42.7	30.6	Ruminococcus lactaris ATCC 29176	II-A(1)	3
28.0	GTTTTAGGGTTATGTTATTTTGAACTGAATTAAAAC	sense	36	GI tract, faeces	NZ_ACCK01000001	36.8	25.0	Catenibacterium mitsuokai DSM 15897	II-A(1)	2
33.4	GTTTTGTTACCATATGGATTTTTGCTAGAATGAGAC	sense	6	GI tract	NZ_ABIK02000004	28.6	33.3	Clostridium spiroforme DSM1552	II-A(2)	4
33.7	GTTTTGTTACCATATGGATTTTTGCTAGATTAAGAC	sense	15	faeces	NZ_ABAW02000024	38.1	30.6	Eubacterium dolichum DSM3991	II-A(2)	4
25.6	GTTTGAGAACTATGTAAATTATGCTGGTAGCAAAAC	sense	unknown	microbiota	NZ_AECQ01000042	36.8	33.3	Solobacterium moorei F0204	II-A(1)	3
37.1	GTTCTTGTACTTTCTAGATTTTCATATTAGTAAAAC	sense	8	gull feces	NZ_AMYT01000017	33.9	25.0	Catellicoccus marimammalium M35 04 3	II-A(2)	4
25.6	GTTTTAGAGCTGTGTTGTTTTGAATGACTCCAAAAC	sense	1	human infections	NZ_AGEF01000010	39.5	36.1	Dolosigranulum pigrum ATCC51524	II-A(1)	1
25.4	GTTTTAGAGTCATGTTGTTTAGAATGGTACCAAAAC	sense	7	intestine mammals	NZ_GG670371	37.4	33.3	Enterococcus faecalis ATCC 4200	II-A(1)	1
42.7	GTTTTTGTACTCTCAATAATTTCTTATCAGTAAAAC	sense	20	intestine mammals	NZ_GG688647	37.6	25.0	Enterococcus faecalis T11	II-A(2)	4

046	Enterococcus faecium Com12	II-A(1)	firmicutes-bacilli	FB	*cas9*	1340	156.7	*cas1*	288	33.8	*cas1*	114	13.4	*csn2*	221
047	Enterococcus hirae ATCC 9790	II-A(1)	firmicutes-bacilli	FB	*cas9*	1336	156.8	*cas1*	288	33.6	*cas1*	114	13.3	*csn2*	219
048	Enterococcus italicus DSM 15952	II-A(1)	firmicutes-bacilli	FB	*cas9*	1330	154.9	*cas1*	288	33.9	*cas1*	114	13.3	*csn2*	219
049	Facklamia hominis CCUG36813	II-A(2)	firmicutes-bacilli	FB	*cas9*	1142	133.1	*cas1*	305	36.1	*cas1*	107	12.8	*csn2*	350
050	Gemella haemolysans M341	II-A(1)	firmicutes-bacilli	FB	*cas9*	1391	161.6	*cas1*	291	34.3	*cas1*	106	12.5	*csn2*	224
051	Lactobacillus animalis KCTC 3501	II-A(1)	firmicutes-bacilli	FB	*cas9*	1314	152.0	*cas1*	290	33.9	*cas1*	110	12.8	*csn2*	223
052	Lactobacillus buchneri CD034	II-A(1)	firmicutes-bacilli	FB	*cas9*	1371	158.3	*cas1*	301	34.6	*cas1*	106	12.6	*csn2*	223
053	Lactobacillus casei BL23	II-A(1)	firmicutes-bacilli	FB	*cas9*	1361	156.0	*cas1*	301	33.6	*cas1*	106	12.5	*csn2*	225
054	Lactobacillus coryniformis subsp torquens KCTC 353[illegible]	II-C	firmicutes-bacilli	FB	*cas9*	1119	129.0	*cas1*	300	34.3	*cas1*	107	12.6	*n/a*	n/a
055	Lactobacillus farciminis KCTC 3681	II-A(2)	firmicutes-bacilli	FB	*cas9*	1126	130.4	*cas1*	302	35.1	*cas1*	107	12.8	*csn2*	340
056	Lactobacillus farciminis KCTC 3681	II-A(1)	firmicutes-bacilli	FB	*cas9*	1356	159.1	*cas1*	292	34.0	*cas1*	106	12.4	*csn2*	234
057	Lactobacillus iners LactinV 11V1-d	II-A(1)	firmicutes-bacilli	FB	*cas9*	1369	160.2	*cas1*	292	34.0	*cas1*	106	12.5	*csn2*	221
058	Lactobacillus rhamnosus R0011	II-A(1)	firmicutes-bacilli	FB	*cas9*	1361	156.0	*cas1*	301	33.5	*cas1*	106	12.5	*csn2*	226
059	Lactobacillus sanfranciscensis TMW 11304	II-A(1)	firmicutes-bacilli	FB	*cas9*	1331	154.9	*cas1*	301	34.7	*cas1*	106	12.6	*csn2*	224
060	Leuconostoc gelidum KCTC3527	II-A(1)	firmicutes-bacilli	FB	*cas9*	1355	156.1	*cas1*	290	33.3	*cas1*	106	12.5	*csn2*	224
061	Oenococcus kitaharae DSM17330	II-A(1)	firmicutes-bacilli	FB	*cas9*	1389	159.3	*cas1*	289	33.1	*cas1*	106	12.4	*csn2*	220
062	Pediococcus acidilactici 7 4	II-A(1)	firmicutes-bacilli	FB	*cas9*	1363	157.6	*cas1*	301	34.1	*cas1*	106	12.8	*csn2*	223
063	Streptococcus anginosus F0211	II-A(1)	firmicutes-bacilli	FB	*cas9*	1345	157.2	*cas1*	288	33.5	*cas1*	114	13.5	*csn2*	220
064	Streptococcus dysgalactiae ATCC 12394	II-A(1)	firmicutes-bacilli	FB	*cas9*	1371	158.2	*cas1*	289	33.4	*cas1*	113	13.4	*csn2*	220
065	Streptococcus equinus ATCC 9812	II-A(1)	firmicutes-bacilli	FB	*cas9*	1377	159.9	*cas1*	288	33.7	*cas1*	114	13.5	*csn2*	220
066	Streptococcus infantarius ATCC BAA-102	II-A(2)	firmicutes-bacilli	FB	*cas9*	1129	130.9	*cas1*	304	35.2	*cas1*	107	12.6	*csn2*	349
067	Streptococcus mitis SK321	II-A(1)	firmicutes-bacilli	FB	*cas9*	1392	161.8	*cas1*	288	33.5	*cas1*	114	13.6	*csn2*	220
068	Streptococcus mutans NN2025	II-A(1)	firmicutes-bacilli	FB	*cas9*	1345	156.7	*cas1*	288	33.7	*cas1*	114	13.3	*csn2*	220
069	Streptococcus pyogenes M1 GAS	II-A(1)	firmicutes-bacilli	FB	*cas9*	1368	158.5	*cas1*	289	33.4	*cas1*	113	13.4	*csn2*	220
070	Streptococcus sanguinis SK330	II-A(1)	firmicutes-bacilli	FB	*cas9*	1392	161.7	*cas1*	288	33.5	*cas1*	114	13.5	*csn2*	220
071	Streptococcus suis ST1	II-A(1)	firmicutes-bacilli	FB	*cas9*	1381	160.6	*cas1*	288	33.6	*cas1*	110	13.0	*csn2*	221
072	Streptococcus suis ST3	II-A(2)	firmicutes-bacilli	FB	*cas9*	1122	129.5	*cas1*	304	35.3	*cas1*	107	12.7	*csn2*	348
073	Streptococcus thermophilus LMD-9 (Cr3)	II-A(1)	firmicutes-bacilli	FB	*cas9*	1388	161.1	*cas1*	289	33.8	*cas1*	114	13.5	*csn2*	219
074	Streptococcus thermophilus LMG18311 (Cr1)	II-A(2)	firmicutes-bacilli	FB	*cas9*	1122	129.9	*cas1*	303	35.2	*cas1*	107	12.7	*csn2*	350
075	Streptococcus vestibularis ATCC 49124	II-A(2)	firmicutes-bacilli	FB	*cas9*	1128	130.6	*cas1*	303	35.3	*cas1*	107	12.7	*csn2*	350
076	Acidaminococcus sp. D21	II-A(1)	firmicutes-negativicutes	FN	*cas9*	1358	157.6	*cas1*	290	33.8	*cas1*	106	12.3	*csn2*	222
077	Megasphaera sp UPII 135-E	II-A(1)	firmicutes-negativicutes	FN	*cas9*	1352	156.0	*cas1*	291	33.3	*cas1*	106	12.4	*csn2*	224
078	Phascolarctobacterium succinatutens YIT 12067	II-C	firmicutes-negativicutes	FN	*cas9*	1087	124.7	*cas1*	300	33.8	*cas1*	106	12.5	*n/a*	n/a
079	Veillonella atypica ACS-134-V-Col7a	II-A(1)	firmicutes-negativicutes	FN	*cas9*	1398	160.6	*cas1*	291	33.7	*cas1*	106	12.5	*csn2*	224
080	Veillonella parvula ATCC17745	II-A(1)	firmicutes-negativicutes	FN	*cas9*	1398	160.2	*cas1*	291	33.5	*cas1*	106	12.4	*csn2*	224
081	Veillonella sp oraltaxon780 F0422	II-A(1)	firmicutes-negativicutes	FN	*cas9*	1375	159.5	*cas1*	292	33.4	*cas1*	106	12.5	*csn2*	224
082	Fusobacterium nucleatum ATCC 49256	II-A(1)	fusobacteria	FU	*cas9*	1374	164.1	*cas1*	239	27.9	*cas1*	106	12.5	*csn2*	220
083	Fusobacterium periodonticum 1 1 41FAA	II-A(1)	fusobacteria	FU	*cas9*	1367	163.1	*cas1*	292	34.3	*cas1*	106	12.5	*csn2*	220
084	Ilyobacter polytropus DSM 2926	II-C	fusobacteria	FU	*cas9*	1092	127.3	*cas1*	300	34.0	*cas1*	106	12.5	*n/a*	n/a
085	Streptobacillus moniliformis DSM12112	II-C	fusobacteria	FU	*cas9*	1259	147.4	*cas1*	308	36.3	*cas1*	104	12.3	*n/a*	n/a
086	Azospirillum sp B510	II-C	alpha-proteobacteria	PA	*cas9*	1102	123.4	*cas1*	316	34.1	*cas1*	108	12.9	*n/a*	n/a
087	Dinoroseobacter shibae DFL 12	II-C	alpha-proteobacteria	PA	*cas9*	1079	121.6	*cas1*	303	32.1	*cas1*	114	13.4	*n/a*	n/a
088	Gluconacetobacter diazotrophicus PAl 5	II-C	alpha-proteobacteria	PA	*cas9*	1046	118.6	*cas1*	297	32.3	*cas1*	109	13.0	*n/a*	n/a
089	Maritimibacter alkaliphilus HTCC2654	II-C	alpha-proteobacteria	PA	*cas9*	1072	120.3	*cas1*	303	32.2	*cas1*	113	13.3	*n/a*	n/a
090	Methylocystis sp ATCC 49242	II-C	alpha-proteobacteria	PA	*cas9*	1080	122.9	*cas1*	301	32.7	*cas1*	109	12.7	*n/a*	n/a
091	Methylosinus trichosporium OB3b	II-C	alpha-proteobacteria	PA	*cas9*	1082	122.4	*cas1*	301	32.6	*cas1*	109	12.8	*n/a*	n/a
092	Parvibaculum lavamentivorans DS-1	II-C	alpha-proteobacteria	PA	*cas9*	1037	117.4	*cas1*	311	34.1	*cas1*	117	13.6	*n/a*	n/a
093	Candidatum Puniceispirillum marinum SAR116	II-C	alpha-proteobacteria	PA	*cas9*	1035	117.6	*cas1*	303	33.8	*cas1*	106	12.2	*n/a*	n/a
094	Rhodopseudomonas palustris BisB18	II-C	alpha-proteobacteria	PA	*cas9*	1066	119.6	*cas1*	299	32.9	*cas1*	109	12.9	*n/a*	n/a
095	Rhodospirillum rubrum ATCC 11170	II-C	alpha-proteobacteria	PA	*cas9*	1173	128.4	*cas1*	319	34.8	*cas1*	112	13.1	*n/a*	n/a
096	Rhodovulum sp PH10	II-C	alpha-proteobacteria	PA	*cas9*	1059	119.2	*cas1*	300	32.2	*cas1*	108	12.6	*n/a*	n/a
097	Sphingobium sp. AP49	II-C	alpha-proteobacteria	PA	*cas9*	1110	124.6	*cas1*	307	32.8	*cas1*	112	13.4	*n/a*	n/a
098	Sphingomonas sp S17	II-C	alpha-proteobacteria	PA	*cas9*	1090	123.8	*cas1*	310	33.3	*cas1*	124	14.4	*n/a*	n/a
099	Tistrella mobilis KA081020-065 plasmid pTM3	II-C	alpha-proteobacteria	PA	*cas9*	1049	118.4	*cas1*	297	32.1	*cas1*	109	12.9	*n/a*	n/a
100	Acidovorax avenae subsp avenae ATCC 19860	II-C	beta-proteobacteria	PB	*cas9*	1045	118.4	*cas1*	309	33.6	*cas1*	108	12.4	*n/a*	n/a

25.9	GTTTTAGAGCTATGCTGATTTGAATGCTTCCAAAAC	sense	6	intestine mammals	NZ_GG670306	38.1	36.1	Enterococcus faecium Com12	II-A(1)	1
25.6	GTTTTAGAGCTATGTTGTTTTGAATGCTTCCAAAAC	sense	2	intestine	CP003504	36.9	33.3	Enterococcus hirae ATCC 9790	II-A(1)	1
25.8	GTTTTAGAGCTATGTTGAATCGAATGCTTCCAAAAC	sense	23	cheese	NZ_GL622241	39.2	36.1	Enterococcus italicus DSM 15952	II-A(1)	1
41.2	GTTTTTGTACTCTCGATAATTTTCTATCAGTAAAAC	sense	5	microbiota	AGZD01000007	39.0	27.8	Facklamia hominis CCUG36813	II-A(2)	4
26.8	GTTTGAGAGATATGTAAATTTTGAATTCTACTAAAC	sense	12	GI tract, respiratory tract	NZ_ACRO01000001	30.7	25.0	Gemella haemolysans M341	II-A(1)	3
25.7	GTTTTAGAGCTATGTTGTTTTGTATGACTCCAAAAC	sense	31	GI tract	AEOF01000001	41.1	33.3	Lactobacillus animalis KCTC 3501	II-A(1)	1
25.5	GTTTTAGAAGGATGTTAAATCAATAAGGTTAAACCC	sense	16	cheese, food sources	NC_018610	44.2	30.6	Lactobacillus buchneri CD034	II-A(1)	2
26.1	GTCTCAGGTAGATGTCGAATCAATCAGTTCAAGAGC	sense	21	human intestine, mouth	NC_010999	46.3	44.4	Lactobacillus casei BL23	II-A(1)	2
n/a	GCTATTGATTCCTTCAGTTTTCA**GCTAAAAT**AGATC	sense	46	cheese, GI tract	AEOS01000001	42.9	33.3	Lactobacillus coryniformis subsp torquens KC	II-C	8a
40.2	GTTTTTGTACCTTAAAGAATCTAGAAATAGTAAAAC	sense	5	GI tract, probiotic	AEOT01000001	36.4	25.0	Lactobacillus farciminis KCTC 3681	II-A(2)	4
27.4	GTTTTAGAAGTATGTCTTTCTATTTACTTAAGAACC	sense	8	GI tract, probiotic	AEOT01000001	36.4	27.8	Lactobacillus farciminis KCTC 3681	II-A(1)	2
25.8	GTTTGAGAGTTATGTAATTTATGCACTAGGTAAAAC	sense	4	urogenital area	NZ_AEHN01000016	32.6	30.6	Lactobacillus iners LactinV 11V1-d	II-A(1)	3
26.3	GTCTCAGGTAGATGTCAGATCAATCAGTTCAAGAAC	sense	28	fermented food sources	AGKC01000001	46.7	41.7	Lactobacillus rhamnosus R0011	II-A(1)	2
26.5	GTTTTAGAAGTACGTCATTCTAATGAGATTAAGAGC	sense	2	sourdough, food sources	NC_015978	34.7	33.3	Lactobacillus sanfranciscensis TMW 11304	II-A(1)	2
25.4	GCTTCAGATGTGTGTCAGATCAATGAGGTTTAAACC	sense	29	kimchi (Korean fermented food)	NZ_AEMI01000021	36.6	41.7	Leuconostoc gelidum KCTC3527	II-A(1)	2
25.6	GCTTCAGATGTGTGTCAGATCAATGAGGTAGAACCC	sense	57	compost distilled shochu residue	NZ_CM001398	42.7	47.2	Oenococcus kitaharae DSM17330	II-A(1)	2
25.6	GTTTCAGAAGGATGTTAAATCAATAAGGTTAAGATC	sense	18	GI tract, probiotic	NZ_GG730083	42.0	30.6	Pediococcus acidilactici 7 4	II-A(1)	2
25.8	GTTTTAGAGCTGTGCTGTTTCGAATGGTTCCAAAAC	sense	17	human orifices	NZ_AECT01000001	38.4	41.7	Streptococcus anginosus F0211	II-A(1)	1
25.6	GTTTTAGAGCTATGTTGTTTTGAATGGTCCCAAAAC	sense	25	skin, mastitis	CP002215	39.5	36.1	Streptococcus dysgalactiae ATCC 12394	II-A(1)	1
25.6	GTTTTAGAGCTGTGCTGTTTCGAATGGTTCCAAAAC	sense	18	skin animals	NZ_GL698429	37.3	41.7	Streptococcus equinus ATCC 9812	II-A(1)	1
41.5	GTTTTTGTACTCTCAAGATTTAAGTAACCGTAAAAC	sense	30	faeces, dairy, blood	NZ_DS572674	37.6	30.6	Streptococcus infantarius ATCC BAA-102	II-A(2)	4
25.6	GTTTTAGAGCTGTGTTGTTTCGAATGGTTCCAAAAC	sense	8	mouth, nasopharynx	NZ_AEDT01000001	40.0	38.9	Streptococcus mitis SK321	II-A(1)	1
26.0	GTTTTAGAGCTGTGTTGTTTCGAATGGTTCCAAAAC	sense	70	oral	NC_013928	36.8	38.9	Streptococcus mutans NN2025	II-A(1)	1
25.6	GTTTTAGAGCTATGCTGTTTTGAATGGTCCCAAAAC	sense	6	throat, skin	NC_002737	38.5	38.9	Streptococcus pyogenes M1 GAS	II-A(1)	1
25.7	GTTTTAGAGCTGTGTTGTTTCGAATGGTTCCAAAAC	sense	11	dental, mouth	AFBD01000001	42.9	38.9	Streptococcus sanguinis SK330	II-A(1)	1
26.0	GTTTTAGAGCTGTGCTGTTTCGAATGGTTTCAAAAC	sense	3	skin, zoonotic, meat	CP002651	41.4	38.9	Streptococcus suis ST1	II-A(1)	1
40.8	GTTTTTGTACTCTCAAGATTTAAGTAACAGTAAAAC	sense	42	skin, zoonotic, meat	NC_015433	41.3	27.8	Streptococcus suis ST3	II-A(2)	4
25.5	GTTTTAGAGCTGTGTTGTTTCGAATGGTTCCAAAAC	sense	8	yoghurt, non-pathogenic	NC_008500	39.1	38.9	Streptococcus thermophilus LMD-9 (Cr3)	II-A(1)	1
41.6	GTTTTTGTACTCTCAAGATTTAAGTAACTGTACAAC	sense	33	yoghurt, non-pathogenic	CP000023	39.1	30.6	Streptococcus thermophilus LMG18311 (Cr1)	II-A(2)	4
41.7	GTTTTTGTACTCTCAAGATTTAAGTAACTGTACAAC	sense	9	oral cavity	NZ_GL831112	39.6	30.6	Streptococcus vestibularis ATCC 49124	II-A(2)	4
25.6	GTTTGAGAGATATGTAAATTCAAAGGATAATCAAAC	sense	39	human colon	NZ_ACGB01000044	50.2	27.8	Acidaminococcus sp. D21	II-A(1)	3
26.0	GTTTGAGAGTAATGTAATTCTATAAATGTCTAAAAC	sense	5	vagina	NZ_AFUG01000001	38.8	25.0	Megasphaera sp UPII 135-E	II-A(1)	3
n/a	GTTGTAGTTCCCCGGTGGTTCTTGGTAT**GGTATAAT**	antisense	11	GI tract microbiota	NZ_GL830843	47.6	44.4	Phascolarctobacterium succinatutens YIT 120	II-C	8a
26.1	GTTTGAGAGTAGTGTAATTCTGTAAATCTCTAAAAC	sense	32	microbiota	NZ_AEDS01000047	39.0	30.6	Veillonella atypica ACS-134-V-Col7a	II-A(1)	3
25.8	GTTTGAGAGTAGTGTGATTCTGTAAATGTCTCAAAC	sense	21	intestine flora mammals	ADFU01000012	38.6	36.1	Veillonella parvula ATCC17745	II-A(1)	3
25.9	GTTTGAGAGTAGTGTAATTCTGTAAACCTCTAAAAC	sense	12	oral flora mammals	AFUJ01000012	39.4	33.3	Veillonella sp oraltaxon780 F0422	II-A(1)	3
26.7	GTTTGAGAGTAATGTTATTTTAAATAGATTCAAAAC	sense	3	oropharynx, gut?	NZ_AABF02000001	27.3	22.2	Fusobacterium nucleatum ATCC 49256	II-A(1)	3
26.6	GTTTGAGAGTAATGTTATTTTAGATAGATATAAAAC	sense	23	oropharynx, gut?	NZ_GG770374	27.9	22.2	Fusobacterium periodonticum 1 1 41FAA	II-A(1)	3
n/a	GTTGTACTTCCCTAATTATTTTAGCTAT**GTTACAAT**	antisense	20	human infections	NC_014632	34.5	27.8	Ilyobacter polytropus DSM 2926	II-C	8a
n/a	GATACAGATTCTT**GGTAAATT**TGAATAATGTGAATG	antisense	47	throat, nasopharynx rat	NC_013515	26.3	27.8	Streptobacillus moniliformis DSM12112	II-C/II-A(2)	4
n/a	GTTGCGGCTGGACCCCCGATCCCCATCG**GCTACACT**	antisense	24	rice plants, stem	NC_013854	67.6	66.7	Azospirillum sp B510	II-C	6
n/a	GTTGCGGCTGGACCCCGAATTCTGAACA**GCTAAACT**	antisense	18	aquatic, dinoflagellates	NC_009952	66.0	52.8	Dinoroseobacter shibae DFL 12	II-C	6
n/a	GCCGTGGTTTCCCTACCGATTTGCCGAT**GGTAGGCT**	antisense	10	endosymbiont sugarcane	NC_010123	66.3	58.3	Gluconacetobacter diazotrophicus PAl 5	II-C	8a
n/a	GTTGCGGCTGGACCTCGAATTCTGAACT**GCTACGCT**	antisense	10	marine, aquatic	NZ_CH902578	64.1	55.6	Maritimibacter alkaliphilus HTCC2654	II-C	6
n/a	GCCGTGGCTTCCCTACCGATTTCCCCGT**GGTAGGCT**	antisense	33	groundwater, bioremedation	NZ_AEVM01000001	62.8	63.9	Methylocystis sp ATCC 49242	II-C	8a
n/a	GCCGTGGCTTCCCTGCCGATTTCCCTGT**GGTAGGCT**	antisense	23	groundwater, bioremedation	NZ_ADVE01000001	65.9	63.9	Methylosinus trichosporium OB3b	II-C	8a
n/a	GCTGCGGATTGCGGCCGTCTCTCGATTT**GCTACTCT**	antisense	48	sewage treatment plant	NC_009719	62.3	58.3	Parvibaculum lavamentivorans DS-1	II-C	6
n/a	GTTGCTCTAGGCTCTCAATCACCAGAGT**GCTATACT**	antisense	29	surface seawater	NC_014010	48.9	47.2	Candidatum Puniceispirillum marinum SAR11	II-C	6
n/a	GCCGTGGCTTCCCTACCGATTTCCCCGT**GGTAGGCT**	antisense	19	marine, soil	NC_007925	65.0	63.9	Rhodopseudomonas palustris BisB18	II-C	8a
n/a	GTTCCATGGCCCCGTCCCACACCGCCAT**GGTAGAGT**	sense	7	environment, soil	NC_007641	65.3	63.9	Rhodospirillum rubrum ATCC 11170	II-C	5
n/a	GTTGCGGTTGGGCCGCGCCGCGTTCCCTGCTAGACC	antisense	11	marine	AKZI01000059	69.3	72.2	Rhodovulum sp PH10	II-C	6
n/a	GTTGCCGCTGGACCGCGATCTCTGAGCG**GCTATGAT**	antisense	7	soil, rhizosphere	AJVL01000060	64.0	61.1	Sphingobium sp. AP49	II-C	6
n/a	GTTGCCGCTGGACCGCAATCTCTGAGCG**GCTATGCT**	antisense	2	Andes lake	AFGG01000001	65.7	61.1	Sphingomonas sp S17	II-C	6
n/a	GCCGTGGTTTCCCTACCGATTTGCCAGT**GGTAGGTT**	antisense	6	soil, sea sediments	CP003239	68.1	55.6	Tistrella mobilis KA081020-065 plasmid pTM3	II-C	8a
n/a	GTTCCGGCCAGTGCGCATATCCCAGTGATC**TAGACT**	antisense	47	fruit	NC_015138	68.8	55.6	Acidovorax avenae subsp avenae ATCC 1986	II-C	6

101	Acidovorax ebreus TPSY	II-C	beta-proteobacteria	PB	*cas9*	1131	128.4	*cas1*	302	33.5	*cas1*	110	12.7	*n/a*	n/a
102	Alicycliphilus denitrificans BC	II-C	beta-proteobacteria	PB	*cas9*	1029	116.3	*cas1*	309	33.5	*cas1*	108	12.6	*n/a*	n/a
103	Neisseria bacilliformis ATCC BAA-1200	II-C	beta-proteobacteria	PB	*cas9*	1077	123.6	*cas1*	304	34.4	*cas1*	108	12.6	*n/a*	n/a
104	Neisseria cinerea ATCC 14685	II-C	beta-proteobacteria	PB	*cas9*	1082	124.3	*cas1*	304	34.4	*cas1*	108	12.6	*n/a*	n/a
105	Neisseria flavescens SK114	II-C	beta-proteobacteria	PB	*cas9*	1081	124.4	*cas1*	304	34.5	*cas1*	108	12.6	*n/a*	n/a
106	Neisseria lactamica ST-640	II-C	beta-proteobacteria	PB	*cas9*	1082	124.4	*cas1*	304	34.6	*cas1*	108	12.6	*n/a*	n/a
107	Neisseria meningitidis Z2491	II-C	beta-proteobacteria	PB	*cas9*	1082	124.3	*cas1*	304	34.5	*cas1*	108	12.6	*n/a*	n/a
108	Ralstonia syzygii R24	II-C	beta-proteobacteria	PB	*cas9*	1062	120.2	*cas1*	307	33.7	*cas1*	110	12.6	*n/a*	n/a
109	Verminephrobacter eiseniae EF01-2	II-C	beta-proteobacteria	PB	*cas9*	1068	121.5	*cas1*	309	33.4	*cas1*	108	12.7	*n/a*	n/a
110	Campylobacter jejuni subsp jejuni NCTC 11168	II-C	epsilon-proteobacteria	PE	*cas9*	984	114.9	*cas1*	296	33.6	*cas1*	143	16.9	*n/a*	n/a
111	Campylobacter lari CF89-12	II-C	epsilon-proteobacteria	PE	*cas9*	1003	116.8	*cas1*	300	34.5	*cas1*	141	16.5	*n/a*	n/a
112	Helicobacter canadensis MIT 98-5491	II-C	epsilon-proteobacteria	PE	*cas9*	1007	117.2	*cas1*	296	34.3	*cas1*	125	14.6	*n/a*	n/a
113	Helicobacter cinaedi CCUG 18818	II-C	epsilon-proteobacteria	PE	*cas9*	1023	116.4	*cas1*	298	33.8	*cas1*	151	17.8	*n/a*	n/a
114	Helicobacter mustelae NCTC 12198	II-C	epsilon-proteobacteria	PE	*cas9*	1024	118.3	*cas1*	308	35.0	*cas1*	107	12.6	*n/a*	n/a
115	Helicobacter pullorum MIT 98-5489	II-C	epsilon-proteobacteria	PE	*cas9*	1061	122.0	*cas1*	317	35.3	*cas1*	149	17.3	*n/a*	n/a
116	Nitratifractor salsuginis DSM 16511	II-C	epsilon-proteobacteria	PE	*cas9*	1132	131.7	*cas1*	304	35.0	*cas1*	110	13.1	*n/a*	n/a
117	Wolinella succinogenes DSM 1740	II-C	epsilon-proteobacteria	PE	*cas9*	1059	122.5	*cas1*	314	34.6	*cas1*	108	12.8	*n/a*	n/a
118	Actinobacillus minor NM305	II-C	gamma-proteobacteria	PG	*cas9*	1056	121.8	*cas1*	304	35.3	*cas1*	108	12.6	*n/a*	n/a
119	Actinobacillus pleuropneumoniae sv10 D13039	II-C	gamma-proteobacteria	PG	*cas9*	1054	121.2	*cas1*	305	34.9	*cas1*	108	12.8	*n/a*	n/a
120	Actinobacillus succinogenes 130Z	II-C	gamma-proteobacteria	PG	*cas9*	1062	121.8	*cas1*	305	34.7	*cas1*	108	12.7	*n/a*	n/a
121	Actinobacillus suis H91-0380	II-C	gamma-proteobacteria	PG	*cas9*	1054	121.0	*cas1*	305	34.9	*cas1*	108	12.8	*n/a*	n/a
122	Gamma proteobacterium HdN1	II-C	gamma-proteobacteria	PG	*cas9*	1025	116.9	*cas1*	303	33.2	*cas1*	108	12.3	*n/a*	n/a
123	Haemophilus parainfluenzae ATCC 33392	II-C	gamma-proteobacteria	PG	*cas9*	1054	121.5	*cas1*	305	35.0	*cas1*	108	12.6	*n/a*	n/a
124	Haemophilus sputorum HK2154	II-C	gamma-proteobacteria	PG	*cas9*	1052	121.0	*cas1*	305	35.0	*cas1*	108	12.7	*n/a*	n/a
125	Pasteurella multocida subsp multocida str Pm70	II-C	gamma-proteobacteria	PG	*cas9*	1056	121.9	*cas1*	304	34.9	*cas1*	108	12.7	*n/a*	n/a
126	Blastopirellula marina DSM3645	II-C	planctomycetes	PL	*cas9*	1052	119.8	*cas1*	296	33.0	*cas1*	109	13.0	*n/a*	n/a
127	Sphaerochaeta globus str. Buddy	II-C	spirochaetes	SP	*cas9*	1179	134.5	*cas1*	300	34.6	*cas1*	108	12.6	*n/a*	n/a
128	Treponema denticola ATCC 35405	II-A(1)	spirochaetes	SP	*cas9*	1395	162.0	*cas1*	290	33.4	*cas1*	106	12.5	*csn2*	224
129	Aminomonas paucivorans DSM 12260	II-C	synergistia	SY	*cas9*	1052	120.5	*cas1*	295	32.8	*cas1*	112	13.1	*n/a*	n/a
130	Mycoplasma canis PG 14	II-A(2)	firmicutes-mollicutes	FM	*cas9*	1233	144.8	*cas1*	292	34.1	*cas1*	104	12.7	*csn2*	251
131	Mycoplasma gallisepticum str. F	II-A(2)	firmicutes-mollicutes	FM	*cas9*	1269	148.7	*cas1*	291	34.2	*cas1*	109	13.2	*csn2*	254
132	Mycoplasma mobile 163K	II-A(2)	firmicutes-mollicutes	FM	*cas9*	1236	147.5	*cas1*	300	35.7	*cas1*	111	13.4	*csn2*	227

n/a	GTTGTAGCTCCCTCTCTCACCCCGGATA**GCTACACT**	antisense	14	wastewater, bioremedation	NC_011992	66.8	55.6	Acidovorax ebreus TPSY	II-C	8b
n/a	GTTCCGGCCAGTGCGCATATCCCAGTGATC**TAGAAT**	antisense	52	wastewater, bioremedation	NC_014908	68.0	52.8	Alicycliphilus denitrificans BC	II-C	6
n/a	GTTGTAGCTTCCTCTCTTATCTCGTAGT**GTTACAAT**	antisense	64	nasopharynx, mucosal surfaces	NZ_AFAY01000001	59.6	38.9	Neisseria bacilliformis ATCC BAA-1200	II-C	8b
n/a	GTTGTAGCTCCCATTCTCATTTCGCAGT**GCTACAAT**	antisense	18	nasopharynx, mucosal surfaces	NZ_ACDY02000001	50.8	44.4	Neisseria cinerea ATCC 14685	II-C	8b
n/a	GTTGTAGCTCCCTTTCTCATTTCGCAGT**GCTACAAT**	antisense	39	nasopharynx, mucosal surfaces	NZ_ACQV01000001	49.2	44.4	Neisseria flavescens SK114	II-C	8b
n/a	GTTGTAGCTCCCTTTCTCATTTCGCAGT**GCTACAAT**	antisense	9	nasopharynx, mucosal surfaces	NC_014752	52.3	44.4	Neisseria lactamica ST-640	II-C	8b
n/a	GTTGTAGCTCCCTTTCTCATTTCGCAGT**GCTACAAT**	antisense	16	nasopharynx, mucosal surfaces	NC_003116	51.8	44.4	Neisseria meningitidis Z2491	II-C	8b
n/a	GTTGTAGCCAGAGCGCAATTCCCGATCT**GCTAACCT**	antisense	35	plant pathogen	FR854086	66.1	52.8	Ralstonia syzygii R24	II-C	6
n/a	GTTCCGGCCAGTGCGCATATCCCAGTGA**GCTACAAT**	antisense	10	kidney-structures of earthworms	NC_008771	65.3	55.6	Verminephrobacter eiseniae EF01-2	II-C	6
n/a	GTTTTAGTCCCTTTTTAAATTTCTTTAT**GGTAAAAT**	antisense	4	caecum, intestine	NC_002163	30.5	22.2	Campylobacter jejuni subsp jejuni NCTC 1116	II-C	7
n/a	GTTTTAGTCTCTTTTTAAATTTCTTTAT**GATAAAAT**	antisense	11	intestine	AB598370	29.9	16.7	Campylobacter lari CF89-12	II-C	7
n/a	GTTTTAGTCCCTTCTTAAATTTCTTTAT**GGTAAAAT**	antisense	2	intestine	NZ_CM000776	33.7	25.0	Helicobacter canadensis MIT 98-5491	II-C	7
n/a	GTTTTAGTCCCTTCTTAAACTTCTATAT**GCTAGAAT**	antisense	31	intestine, rectum	NZ_DS990391	38.5	30.6	Helicobacter cinaedi CCUG 18818	II-C	7
n/a	GTTTTAGCCACTTCATAAATATGTTTAT**GCTAAAAT**	antisense	10	gastric	NC_013949	42.5	25.0	Helicobacter mustelae NCTC 12198	II-C	7
n/a	GTTTTAGTCCCTTTTTAAATCTTTCTAT**GCTAAAAT**	antisense	24	intestine	NZ_DS990441	34.1	25.0	Helicobacter pullorum MIT 98-5489	II-C	7
n/a	GTTTTAAGACCCCTCAAAACCCCACCCT**GTTACAAT**	sense	10	hydrothermal vent	NC_014935	53.9	44.4	Nitratifractor salsuginis DSM 16511	II-C	5
n/a	GTTATAGCCGCCTACTCAGCCATTCCTC**GCTATAAT**	antisense	9	rumen	NC_005090	48.5	47.2	Wolinella succinogenes DSM 1740	II-C	7
n/a	GTTGTAGCTCCCTTTCTCATTTCGCAGT**GCTACAAT**	antisense	8	respiratory tract	NZ_ACQL01000001	39.3	44.4	Actinobacillus minor NM305	II-C	8b
n/a	GTTGTAGCTCCCTTTTTCATTTCGCAGT**GCTATAAT**	antisense	4	respiratory tract	NZ_ADOJ01000001	41.2	38.9	Actinobacillus pleuropneumoniae sv10 D1303	II-C	8b
n/a	GTTGTAGCTCCCTTTTTCATTTCGCAGT**GCTATAAT**	antisense	6	respiratory tract	NC_009655	44.9	38.9	Actinobacillus succinogenes 130Z	II-C	8b
n/a	GTTGTAGCTCCCTTTTTCATTTCGCAGT**GCTATAAT**	antisense	10	respiratory tract	NC_018690	40.2	38.9	Actinobacillus suis H91-0380	II-C	8b
n/a	GTTCCGGATGCAGCACATATCCCGATAATG**TAGACT**	antisense	7	environment	NC_014366	53.3	47.2	Gamma proteobacterium HdN1	II-C	6
n/a	GTTGTAGCTCCCTTTTTCATTTCGCAGT**GCTATAAT**	antisense	30	respiratory tract	NZ_GL872339	39.2	38.9	Haemophilus parainfluenzae ATCC 33392	II-C	8b
n/a	GTTGTAGCTCCCTTTTTCATTTCGCAGT**GCTATAAT**	antisense	11	respiratory tract	ALJP01000006	39.5	38.9	Haemophilus sputorum HK2154	II-C	8b
n/a	GTTGTAGTTCCCTCTCTCATTTCGCAGT**GCTACAAT**	antisense	5	respiratory tract, zoonotic	NC_002663	40.4	44.4	Pasteurella multocida subsp multocida str Pm	II-C	8b
n/a	GTTGCGGATTGGTCCCAAGCAGGAATCA**GATACACT**	antisense	3	aquatic habitat	AANZ01000004	57.0	50.0	Blastopirellula marina DSM3645	II-C	6
n/a	GTTGGGGATGACCGCTGATTTTT**GTTAAGAT**TGACC	antisense	42	freshwater sediment	NC_015152	48.9	44.4	Sphaerochaeta globus str. Buddy	II-C	5
26.2	GTTTGAGAGTTGTGTAATTTAAGATGGATCTCAAAC	sense	57	oral spirochete	NC_002967	37.9	33.3	Treponema denticola ATCC 35405	II-A(1)	3
n/a	GTCATAGCTCCCTGCCGCACTCCGAAAT**GCTATGCT**	antisense	6	wastewater	NZ_CM001022	67.6	55.6	Aminomonas paucivorans DSM 12260	II-C	8b
29.3	GTTTTAGTGTTGTACAATATTTGGGTAAACAATAAC	sense	10	respiratory tract	AJFQ01000001	27.0	27.8	Mycoplasma canis PG 14	II-A(2)	4
30.3	GTTTTAGCACTGTACAATACTTGTGTAAGCAATAAC	sense	39	respiratory tract	CP001873	31.4	33.3	Mycoplasma gallisepticum str. F	II-A(2)	4
26.9	GTTTTGGTGTAGTATCATTCTTATGTATTCTTAAAC	sense	56	fish	NC_006908	25.0	27.8	Mycoplasma mobile 163K	II-A(2)	4

Table S2. Oligonucleotide primers used in this study

**Primer designation**	**Sequence (5' > 3')**
RNA oligo	AUAUGCGCGAAUUCCUGUAGAACGAACACUAGAAGAAA
5’ linker specific	GCGCGAATTCCTGTAGA
CRISPR1	AAGGGACTAAAATAAACCCTAG
CRISPR2	AAGGGACTAAAACAACCGCCAA
CRISPR3	AAGGGACTAAAACAGTTAAATTGAAC
CRISPR4	AAGGGACTAAAACTGTAAAATATC
CRISPR5	AAGGGACTAAAACATTTAAGTCC

Table S3. EMBL/Genbank/DDBJ and PubMLST accession numbers of *C. jejuni* and *C. coli* genomes included in this study
a) access to genome sequences via Genbank, PATRIC and pubMLST (http://pubmlst.org/campylobacter/)
b) Accession number Genbank/EMBL/DDBJ or PubMLST ID (http://pubmlst.org/campylobacter/)
c) MLST sequence type (ST) and clonal complex were determined with the definitions file from pubMLST (http://pubmlst.org/campylobacter/)
d) *C. coli* clades as described by Sheppard et al, 2013; CL1, Clade 1;1150, Clade 1 ST-1150 (agricultural); CL2, Clade 2; CL3, Clade 3 (riparian)
e) Cco CRISPR, *C. coli* CRISPR system; Cje CRISPR, *C. jejuni* CRISPR-Cas
f) Full length Cas9 based on BLAST-searches of (re)annotated genome sequences. If not full length, this can be due to distribution over multiple contigs or inactivation

Campylobacter coli

Isolate / Strain	Source (a)	Accession/ID (b)	MLST ST (c)	clonal complex (c)	Clade (d)	Cco CRISPR (e)	Cje CRISPR (e)	full length Cas9 (f)
Campylobacter coli 911	PubMLST	24265	N/A	0000	1150	no	no	none
Campylobacter coli C4B19	PubMLST	24261	N/A	0000	1150	no	no	none
Campylobacter coli 317/04	Genbank/EMBL/DDBJ	AINJ01	5160	1150	1150	no	no	none
Campylobacter coli C28B51	PubMLST	24259	3667	1150	1150	no	no	none
Campylobacter coli OXC6395	PubMLST	16192	1487	1150	1150	no	no	none
Campylobacter coli OXC7352	PubMLST	27877	6973	1150	1150	no	no	none
Campylobacter coli RM4661	Genbank/EMBL/DDBJ	CP007181	1135	0000	1150	no	yes	yes
Campylobacter coli 182	PubMLST	24260	N/A	0000	CL1	no	no	none
Campylobacter coli 2548	Genbank/EMBL/DDBJ	AIML01	1167	0000	CL1	no	no	none
Campylobacter coli 8993	PubMLST	24255	2696	0000	CL1	no	no	none
Campylobacter coli 90-3	Genbank/EMBL/DDBJ	AIMJ01	3862	0000	CL1	no	no	none
Campylobacter coli BID18P	PubMLST	25073	3225	0000	CL1	no	no	none
Campylobacter coli CVM N29710	Genbank/EMBL/DDBJ	CP004066	N/A	0000	CL1	no	no	none
Campylobacter coli CVM N29716	Genbank/EMBL/DDBJ	ANMS01	N/A	0000	CL1	no	no	none
Campylobacter coli Dg138a	PubMLST	26039	2195	0000	CL1	no	no	none
Campylobacter coli Dg52	PubMLST	25979	1109	0000	CL1	no	no	none
Campylobacter coli H143960243	PubMLST	31009	5150	0000	CL1	no	no	none
Campylobacter coli LMG 9860	Genbank/EMBL/DDBJ	AINS01	900	0000	CL1	no	no	none
Campylobacter coli OXC4960	PubMLST	25371	N/A	0000	CL1	no	no	none
Campylobacter coli OXC5391	PubMLST	21351	2195	0000	CL1	no	no	none
Campylobacter coli OXC5441	PubMLST	21386	N/A	0000	CL1	no	no	none
Campylobacter coli OXC5486	PubMLST	23494	5158	0000	CL1	no	no	none
Campylobacter coli OXC5768	PubMLST	21489	5150	0000	CL1	no	no	none
Campylobacter coli OXC6425	PubMLST	16220	4988	0000	CL1	no	no	none
Campylobacter coli OXC6434	PubMLST	16229	2195	0000	CL1	no	no	none
Campylobacter coli OXC6442	PubMLST	16236	5736	0000	CL1	no	no	none
Campylobacter coli OXC6443	PubMLST	16237	5736	0000	CL1	no	no	none
Campylobacter coli OXC6587	PubMLST	12924	1009	0000	CL1	no	no	none
Campylobacter coli OXC6762	PubMLST	18319	5814	0000	CL1	no	no	none
Campylobacter coli OXC7363	PubMLST	23902	1009	0000	CL1	no	no	none
Campylobacter coli OXC7436	PubMLST	23967	4688	0000	CL1	no	no	none
Campylobacter coli OXC7457	PubMLST	23987	6703	0000	CL1	no	no	none
Campylobacter coli OXC7476	PubMLST	24005	2195	0000	CL1	no	no	none
Campylobacter coli OXC7550	PubMLST	25626	6895	0000	CL1	no	no	none
Campylobacter coli OXC7553	PubMLST	24491	6757	0000	CL1	no	no	none
Campylobacter coli OXC7615	PubMLST	24544	6760	0000	CL1	no	no	none
Campylobacter coli OXC7650	PubMLST	24907	6887	0000	CL1	no	no	none
Campylobacter coli OXC7732	PubMLST	24971	1661	0000	CL1	no	no	none
Campylobacter coli OXC7803	PubMLST	24603	2195	0000	CL1	no	no	none
Campylobacter coli OXC8425	PubMLST	28616	7117	0000	CL1	no	no	none
Campylobacter coli OXC8450	PubMLST	28641	7117	0000	CL1	no	no	none
Campylobacter coli OXC8624	PubMLST	28947	2195	0000	CL1	no	no	none
Campylobacter coli RM1875	Genbank/EMBL/DDBJ	CP007183	900	0000	CL1	no	no	none
Campylobacter coli RM2228	Genbank/EMBL/DDBJ	AAFL01	N/A	0000	CL1	no	no	none
Campylobacter coli UKZN016_R	PubMLST	24347	6465	0000	CL1	no	no	none
Campylobacter coli 1091	Genbank/EMBL/DDBJ	AIMV01	1068	0828	CL1	no	no	none
Campylobacter coli 1148	Genbank/EMBL/DDBJ	AIMX01	1068	0828	CL1	no	no	none
Campylobacter coli 1417	Genbank/EMBL/DDBJ	AIMY01	3221	0828	CL1	no	no	none
Campylobacter coli 15-537360	Genbank/EMBL/DDBJ	CP006702	855	0828	CL1	no	no	none
Campylobacter coli 1891	Genbank/EMBL/DDBJ	AINB01	1068	0828	CL1	no	no	none
Campylobacter coli 202/04	Genbank/EMBL/DDBJ	AINH01	1585	0828	CL1	no	no	none
Campylobacter coli 2553	Genbank/EMBL/DDBJ	AIMM01	825	0828	CL1	no	no	none
Campylobacter coli 2680	Genbank/EMBL/DDBJ	AIMN01	3872	0828	CL1	no	no	none
Campylobacter coli 2685	Genbank/EMBL/DDBJ	AIMO01	1082	0828	CL1	no	no	none
Campylobacter coli 2688	Genbank/EMBL/DDBJ	AIMP01	1017	0828	CL1	no	no	none
Campylobacter coli 37/05	Genbank/EMBL/DDBJ	AINK01	1191	0828	CL1	no	no	none
Campylobacter coli 67-8	Genbank/EMBL/DDBJ	AINI01	1061	0828	CL1	no	no	none
Campylobacter coli 84-2	Genbank/EMBL/DDBJ	AIMS01	1113	0828	CL1	no	no	none
Campylobacter coli 86119	Genbank/EMBL/DDBJ	AIMU01	825	0828	CL1	no	no	none
Campylobacter coli ARI_1231_R	PubMLST	24326	827	0828	CL1	no	no	none
Campylobacter coli BB2617	PubMLST	24264	828	0828	CL1	no	no	none
Campylobacter coli BID1QH	PubMLST	25082	6843	0828	CL1	no	no	none
Campylobacter coli C138	PubMLST	24258	867	0828	CL1	no	no	none
Campylobacter coli C256_R	PubMLST	24332	828	0828	CL1	no	no	none
Campylobacter coli C4B30	PubMLST	24262	828	0828	CL1	no	no	none
Campylobacter coli Dg106	PubMLST	26057	1465	0828	CL1	no	no	none
Campylobacter coli Dg121	PubMLST	25961	1438	0828	CL1	no	no	none
Campylobacter coli Dg132b	PubMLST	26000	962	0828	CL1	no	no	none
Campylobacter coli Dg172	PubMLST	26067	1016	0828	CL1	no	no	none
Campylobacter coli Dg185	PubMLST	26047	1438	0828	CL1	no	no	none
Campylobacter coli Dg349	PubMLST	25986	1016	0828	CL1	no	no	none
Campylobacter coli Dg45c	PubMLST	25954	887	0828	CL1	no	no	none
Campylobacter coli Dg64	PubMLST	25953	2273	0828	CL1	no	no	none
Campylobacter coli Dg97	PubMLST	26011	854	0828	CL1	no	no	none
Campylobacter coli Dg9a	PubMLST	25969	827	0828	CL1	no	no	none
Campylobacter coli F79015	PubMLST	24254	867	0828	CL1	no	no	none
Campylobacter coli H122660720	PubMLST	2643	829	0828	CL1	no	no	none
Campylobacter coli H133920799	PubMLST	2657	827	0828	CL1	no	no	none
Campylobacter coli H134000451	PubMLST	2660	1579	0828	CL1	no	no	none
Campylobacter coli H134660451	PubMLST	2665	1666	0828	CL1	no	no	none
Campylobacter coli H142080277	PubMLST	2674	872	0828	CL1	no	no	none
Campylobacter coli H142240934	PubMLST	2676	7254	0828	CL1	no	no	none
Campylobacter coli H142240935	PubMLST	2678	825	0828	CL1	no	no	none

Campylobacter coli H6	Genbank/EMBL/DDBJ	AINT01	3020	0828	CL1	no	no	none
Campylobacter coli H8	Genbank/EMBL/DDBJ	AINU01	901	0828	CL1	no	no	none
Campylobacter coli H9	Genbank/EMBL/DDBJ	AINV01	825	0828	CL1	no	no	none
Campylobacter coli IPSID-1	Genbank/EMBL/DDBJ	CBXC01	4956	0828	CL1	no	no	none
Campylobacter coli JV20	Genbank/EMBL/DDBJ	AEER01	860	0828	CL1	no	no	none
Campylobacter coli K7	Genbank/EMBL/DDBJ	AYKO01	860	0828	CL1	no	no	none
Campylobacter coli LMG 23336	Genbank/EMBL/DDBJ	AINM01	3868	0828	CL1	no	no	none
Campylobacter coli LMG 23341	Genbank/EMBL/DDBJ	AINN01	855	0828	CL1	no	no	none
Campylobacter coli LMG 23342	Genbank/EMBL/DDBJ	AINO01	855	0828	CL1	no	no	none
Campylobacter coli LMG 23344	Genbank/EMBL/DDBJ	AINP01	1586	0828	CL1	no	no	none
Campylobacter coli LMG 9853	Genbank/EMBL/DDBJ	AINR01	3869	0828	CL1	no	no	none
Campylobacter coli LMG 9854	Genbank/EMBL/DDBJ	AINL01	1068	0828	CL1	no	no	none
Campylobacter coli OXC4571	PubMLST	24048	825	0828	CL1	no	no	none
Campylobacter coli OXC4580	PubMLST	24054	827	0828	CL1	no	no	none
Campylobacter coli OXC4611	PubMLST	22130	6233	0828	CL1	no	no	none
Campylobacter coli OXC4617	PubMLST	24072	6233	0828	CL1	no	no	none
Campylobacter coli OXC4647	PubMLST	24093	872	0828	CL1	no	no	none
Campylobacter coli OXC4766	PubMLST	18459	825	0828	CL1	no	no	none
Campylobacter coli OXC4814	PubMLST	22150	827	0828	CL1	no	no	none
Campylobacter coli OXC4825	PubMLST	22154	829	0828	CL1	no	no	none
Campylobacter coli OXC4829	PubMLST	22155	825	0828	CL1	no	no	none
Campylobacter coli OXC4860	PubMLST	22172	827	0828	CL1	no	no	none
Campylobacter coli OXC4892	PubMLST	24458	4425	0828	CL1	no	no	none
Campylobacter coli OXC4949	PubMLST	24472	1055	0828	CL1	no	no	none
Campylobacter coli OXC4959	PubMLST	25563	4425	0828	CL1	no	no	none
Campylobacter coli OXC4968	PubMLST	24659	5164	0828	CL1	no	no	none
Campylobacter coli OXC5146	PubMLST	24761	827	0828	CL1	no	no	none
Campylobacter coli OXC5172	PubMLST	21310	825	0828	CL1	no	no	none
Campylobacter coli OXC5240	PubMLST	24823	2183	0828	CL1	no	no	none
Campylobacter coli OXC5253	PubMLST	24831	825	0828	CL1	no	no	none
Campylobacter coli OXC5254	PubMLST	24832	829	0828	CL1	no	no	none
Campylobacter coli OXC5301	PubMLST	24848	825	0828	CL1	no	no	none
Campylobacter coli OXC5348	PubMLST	21328	827	0828	CL1	no	no	none
Campylobacter coli OXC5353	PubMLST	21331	5149	0828	CL1	no	no	none
Campylobacter coli OXC5363	PubMLST	21334	827	0828	CL1	no	no	none
Campylobacter coli OXC5370	PubMLST	21337	827	0828	CL1	no	no	none
Campylobacter coli OXC5386	PubMLST	21349	825	0828	CL1	no	no	none
Campylobacter coli OXC5473	PubMLST	21405	6131	0828	CL1	no	no	none
Campylobacter coli OXC5482	PubMLST	23492	2273	0828	CL1	no	no	none
Campylobacter coli OXC5605	PubMLST	23502	832	0828	CL1	no	no	none
Campylobacter coli OXC5629	PubMLST	23515	855	0828	CL1	no	no	none
Campylobacter coli OXC5635	PubMLST	23521	1541	0828	CL1	no	no	none
Campylobacter coli OXC5677	PubMLST	21424	6132	0828	CL1	no	no	none
Campylobacter coli OXC5685	PubMLST	21429	1578	0828	CL1	no	no	none
Campylobacter coli OXC5688	PubMLST	21431	827	0828	CL1	no	no	none
Campylobacter coli OXC5705	PubMLST	21443	872	0828	CL1	no	no	none
Campylobacter coli OXC5723	PubMLST	21455	827	0828	CL1	no	no	none
Campylobacter coli OXC5742	PubMLST	21469	872	0828	CL1	no	no	none
Campylobacter coli OXC5763	PubMLST	21484	825	0828	CL1	no	no	none
Campylobacter coli OXC5773	PubMLST	21493	828	0828	CL1	no	no	none
Campylobacter coli OXC5796	PubMLST	23546	872	0828	CL1	no	no	none
Campylobacter coli OXC5810	PubMLST	23558	5165	0828	CL1	no	no	none
Campylobacter coli OXC5827	PubMLST	23574	860	0828	CL1	no	no	none
Campylobacter coli OXC5831	PubMLST	23577	1191	0828	CL1	no	no	none
Campylobacter coli OXC5837	PubMLST	23580	827	0828	CL1	no	no	none
Campylobacter coli OXC5842	PubMLST	23585	2178	0828	CL1	no	no	none
Campylobacter coli OXC5849	PubMLST	23591	830	0828	CL1	no	no	none
Campylobacter coli OXC5853	PubMLST	23593	830	0828	CL1	no	no	none
Campylobacter coli OXC5855	PubMLST	23595	825	0828	CL1	no	no	none
Campylobacter coli OXC5856	PubMLST	23596	830	0828	CL1	no	no	none
Campylobacter coli OXC5864	PubMLST	23602	1191	0828	CL1	no	no	none
Campylobacter coli OXC5870	PubMLST	23606	4425	0828	CL1	no	no	none
Campylobacter coli OXC5922	PubMLST	23645	827	0828	CL1	no	no	none
Campylobacter coli OXC5923	PubMLST	23646	827	0828	CL1	no	no	none
Campylobacter coli OXC6172	PubMLST	29342	827	0828	CL1	no	no	none
Campylobacter coli OXC6253	PubMLST	16051	829	0828	CL1	no	no	none
Campylobacter coli OXC6258	PubMLST	16055	860	0828	CL1	no	no	none
Campylobacter coli OXC6263	PubMLST	16060	830	0828	CL1	no	no	none
Campylobacter coli OXC6267	PubMLST	16064	5642	0828	CL1	no	no	none
Campylobacter coli OXC6276	PubMLST	16073	5733	0828	CL1	no	no	none
Campylobacter coli OXC6296	PubMLST	16093	5737	0828	CL1	no	no	none
Campylobacter coli OXC6297	PubMLST	16094	825	0828	CL1	no	no	none
Campylobacter coli OXC6308	PubMLST	16105	827	0828	CL1	no	no	none
Campylobacter coli OXC6309	PubMLST	16106	5734	0828	CL1	no	no	none
Campylobacter coli OXC6312	PubMLST	16109	827	0828	CL1	no	no	none
Campylobacter coli OXC6337	PubMLST	16134	1600	0828	CL1	no	no	none
Campylobacter coli OXC6338	PubMLST	16135	1628	0828	CL1	no	no	none
Campylobacter coli OXC6343	PubMLST	16140	962	0828	CL1	no	no	none
Campylobacter coli OXC6371	PubMLST	16168	5734	0828	CL1	no	no	none
Campylobacter coli OXC6372	PubMLST	16169	4425	0828	CL1	no	no	none
Campylobacter coli OXC6376	PubMLST	16173	5734	0828	CL1	no	no	none
Campylobacter coli OXC6378	PubMLST	16175	4425	0828	CL1	no	no	none
Campylobacter coli OXC6380	PubMLST	16177	5735	0828	CL1	no	no	none
Campylobacter coli OXC6385	PubMLST	16182	1628	0828	CL1	no	no	none
Campylobacter coli OXC6386	PubMLST	16183	829	0828	CL1	no	no	none
Campylobacter coli OXC6400	PubMLST	16195	825	0828	CL1	no	no	none
Campylobacter coli OXC6416	PubMLST	16211	1145	0828	CL1	no	no	none
Campylobacter coli OXC6424	PubMLST	16219	825	0828	CL1	no	no	none
Campylobacter coli OXC6426	PubMLST	16221	2464	0828	CL1	no	no	none
Campylobacter coli OXC6428	PubMLST	16223	832	0828	CL1	no	no	none
Campylobacter coli OXC6447	PubMLST	16240	827	0828	CL1	no	no	none
Campylobacter coli OXC6460	PubMLST	16252	1541	0828	CL1	no	no	none

Campylobacter coli OXC6471	PubMLST	16263	827	0828	CL1	no	no	none
Campylobacter coli OXC6471_V2	PubMLST	24169	827	0828	CL1	no	no	none
Campylobacter coli OXC6471R	PubMLST	21173	827	0828	CL1	no	no	none
Campylobacter coli OXC6471R_V2	PubMLST	24179	827	0828	CL1	no	no	none
Campylobacter coli OXC6472	PubMLST	16264	827	0828	CL1	no	no	none
Campylobacter coli OXC6472_V2	PubMLST	24170	827	0828	CL1	no	no	none
Campylobacter coli OXC6472R	PubMLST	21174	827	0828	CL1	no	no	none
Campylobacter coli OXC6472R_V2	PubMLST	24180	827	0828	CL1	no	no	none
Campylobacter coli OXC6474	PubMLST	16266	827	0828	CL1	no	no	none
Campylobacter coli OXC6476	PubMLST	16268	860	0828	CL1	no	no	none
Campylobacter coli OXC6504	PubMLST	16296	832	0828	CL1	no	no	none
Campylobacter coli OXC6513	PubMLST	16305	828	0828	CL1	no	no	none
Campylobacter coli OXC6523	PubMLST	16315	5659	0828	CL1	no	no	none
Campylobacter coli OXC6537	PubMLST	16329	1541	0828	CL1	no	no	none
Campylobacter coli OXC6568	PubMLST	12903	872	0828	CL1	no	no	none
Campylobacter coli OXC6576	PubMLST	12911	855	0828	CL1	no	no	none
Campylobacter coli OXC6576_V2	PubMLST	24177	855	0828	CL1	no	no	none
Campylobacter coli OXC6576R	PubMLST	21181	855	0828	CL1	no	no	none
Campylobacter coli OXC6576R_V2	PubMLST	24187	855	0828	CL1	no	no	none
Campylobacter coli OXC6577	PubMLST	12912	872	0828	CL1	no	no	none
Campylobacter coli OXC6597	PubMLST	16348	855	0828	CL1	no	no	none
Campylobacter coli OXC6601	PubMLST	16352	1614	0828	CL1	no	no	none
Campylobacter coli OXC6630	PubMLST	16379	1145	0828	CL1	no	no	none
Campylobacter coli OXC6651	PubMLST	18208	1181	0828	CL1	no	no	none
Campylobacter coli OXC6684	PubMLST	18241	962	0828	CL1	no	no	none
Campylobacter coli OXC6685	PubMLST	18242	827	0828	CL1	no	no	none
Campylobacter coli OXC6705	PubMLST	18262	1957	0828	CL1	no	no	none
Campylobacter coli OXC6710	PubMLST	18267	5810	0828	CL1	no	no	none
Campylobacter coli OXC6735	PubMLST	18292	1145	0828	CL1	no	no	none
Campylobacter coli OXC6738	PubMLST	18295	2273	0828	CL1	no	no	none
Campylobacter coli OXC6761	PubMLST	18318	827	0828	CL1	no	no	none
Campylobacter coli OXC6765	PubMLST	18322	832	0828	CL1	no	no	none
Campylobacter coli OXC6785	PubMLST	18342	827	0828	CL1	no	no	none
Campylobacter coli OXC6810	PubMLST	18367	825	0828	CL1	no	no	none
Campylobacter coli OXC6817	PubMLST	18374	962	0828	CL1	no	no	none
Campylobacter coli OXC6825	PubMLST	18382	2183	0828	CL1	no	no	none
Campylobacter coli OXC6847	PubMLST	21566	829	0828	CL1	no	no	none
Campylobacter coli OXC6864	PubMLST	21583	829	0828	CL1	no	no	none
Campylobacter coli OXC6873	PubMLST	21592	5349	0828	CL1	no	no	none
Campylobacter coli OXC6901	PubMLST	22096	827	0828	CL1	no	no	none
Campylobacter coli OXC6920	PubMLST	22115	827	0828	CL1	no	no	none
Campylobacter coli OXC6933	PubMLST	21107	827	0828	CL1	no	no	none
Campylobacter coli OXC6937	PubMLST	21111	3753	0828	CL1	no	no	none
Campylobacter coli OXC6962	PubMLST	21136	2273	0828	CL1	no	no	none
Campylobacter coli OXC6984	PubMLST	21157	1055	0828	CL1	no	no	none
Campylobacter coli OXC6987	PubMLST	21160	825	0828	CL1	no	no	none
Campylobacter coli OXC6996	PubMLST	21167	827	0828	CL1	no	no	none
Campylobacter coli OXC7027	PubMLST	22214	1016	0828	CL1	no	no	none
Campylobacter coli OXC7051	PubMLST	22236	1016	0828	CL1	no	no	none
Campylobacter coli OXC7054	PubMLST	22239	825	0828	CL1	no	no	none
Campylobacter coli OXC7070	PubMLST	22254	827	0828	CL1	no	no	none
Campylobacter coli OXC7082	PubMLST	22266	825	0828	CL1	no	no	none
Campylobacter coli OXC7083	PubMLST	22267	855	0828	CL1	no	no	none
Campylobacter coli OXC7097	PubMLST	22279	827	0828	CL1	no	no	none
Campylobacter coli OXC7110	PubMLST	21192	1191	0828	CL1	no	no	none
Campylobacter coli OXC7124	PubMLST	21206	827	0828	CL1	no	no	none
Campylobacter coli OXC7130	PubMLST	21211	2183	0828	CL1	no	no	none
Campylobacter coli OXC7131	PubMLST	21212	828	0828	CL1	no	no	none
Campylobacter coli OXC7154	PubMLST	21235	828	0828	CL1	no	no	none
Campylobacter coli OXC7164	PubMLST	21245	827	0828	CL1	no	no	none
Campylobacter coli OXC7177	PubMLST	22668	4425	0828	CL1	no	no	none
Campylobacter coli OXC7199	PubMLST	22690	827	0828	CL1	no	no	none
Campylobacter coli OXC7200	PubMLST	22691	829	0828	CL1	no	no	none
Campylobacter coli OXC7213	PubMLST	22704	825	0828	CL1	no	no	none
Campylobacter coli OXC7218	PubMLST	22709	6543	0828	CL1	no	no	none
Campylobacter coli OXC7218_R	PubMLST	25550	6543	0828	CL1	no	no	none
Campylobacter coli OXC7228	PubMLST	22719	830	0828	CL1	no	no	none
Campylobacter coli OXC7238	PubMLST	22728	827	0828	CL1	no	no	none
Campylobacter coli OXC7240	PubMLST	22730	1614	0828	CL1	no	no	none
Campylobacter coli OXC7259	PubMLST	22300	4425	0828	CL1	no	no	none
Campylobacter coli OXC7272	PubMLST	22313	827	0828	CL1	no	no	none
Campylobacter coli OXC7301	PubMLST	22342	827	0828	CL1	no	no	none
Campylobacter coli OXC7321	PubMLST	22360	827	0828	CL1	no	no	none
Campylobacter coli OXC7347	PubMLST	23887	1055	0828	CL1	no	no	none
Campylobacter coli OXC7382	PubMLST	23920	1628	0828	CL1	no	no	none
Campylobacter coli OXC7394	PubMLST	23932	827	0828	CL1	no	no	none
Campylobacter coli OXC7402	PubMLST	23938	827	0828	CL1	no	no	none
Campylobacter coli OXC7429	PubMLST	23962	825	0828	CL1	no	no	none
Campylobacter coli OXC7444	PubMLST	23975	1628	0828	CL1	no	no	none
Campylobacter coli OXC7447	PubMLST	23977	855	0828	CL1	no	no	none
Campylobacter coli OXC7453	PubMLST	23983	825	0828	CL1	no	no	none
Campylobacter coli OXC7474	PubMLST	24003	6755	0828	CL1	no	no	none
Campylobacter coli OXC7495	PubMLST	24023	825	0828	CL1	no	no	none
Campylobacter coli OXC7506	PubMLST	24032	1614	0828	CL1	no	no	none
Campylobacter coli OXC7522	PubMLST	24126	825	0828	CL1	no	no	none
Campylobacter coli OXC7542	PubMLST	24483	3016	0828	CL1	no	no	none
Campylobacter coli OXC7563	PubMLST	24500	860	0828	CL1	no	no	none
Campylobacter coli OXC7572	PubMLST	24509	828	0828	CL1	no	no	none
Campylobacter coli OXC7575	PubMLST	24512	855	0828	CL1	no	no	none
Campylobacter coli OXC7580	PubMLST	24516	825	0828	CL1	no	no	none
Campylobacter coli OXC7581	PubMLST	25553	825	0828	CL1	no	no	none
Campylobacter coli OXC7593	PubMLST	24525	872	0828	CL1	no	no	none

Campylobacter coli OXC7598	PubMLST	24530	872	0828	CL1	no	no	none
Campylobacter coli OXC7609	PubMLST	24539	6705	0828	CL1	no	no	none
Campylobacter coli OXC7618	PubMLST	24547	827	0828	CL1	no	no	none
Campylobacter coli OXC7622	PubMLST	24551	829	0828	CL1	no	no	none
Campylobacter coli OXC7623	PubMLST	24552	4425	0828	CL1	no	no	none
Campylobacter coli OXC7629	PubMLST	24558	6706	0828	CL1	no	no	none
Campylobacter coli OXC7636	PubMLST	24565	828	0828	CL1	no	no	none
Campylobacter coli OXC7648	PubMLST	24905	2178	0828	CL1	no	no	none
Campylobacter coli OXC7649	PubMLST	24906	4956	0828	CL1	no	no	none
Campylobacter coli OXC7685	PubMLST	25465	827	0828	CL1	no	no	none
Campylobacter coli OXC7687	PubMLST	24938	827	0828	CL1	no	no	none
Campylobacter coli OXC7694	PubMLST	24942	2689	0828	CL1	no	no	none
Campylobacter coli OXC7701	PubMLST	24949	827	0828	CL1	no	no	none
Campylobacter coli OXC7702	PubMLST	24950	828	0828	CL1	no	no	none
Campylobacter coli OXC7706	PubMLST	24953	827	0828	CL1	no	no	none
Campylobacter coli OXC7713	PubMLST	27865	829	0828	CL1	no	no	none
Campylobacter coli OXC7725	PubMLST	24966	825	0828	CL1	no	no	none
Campylobacter coli OXC7739	PubMLST	25651	827	0828	CL1	no	no	none
Campylobacter coli OXC7771	PubMLST	27881	825	0828	CL1	no	no	none
Campylobacter coli OXC7773	PubMLST	24581	825	0828	CL1	no	no	none
Campylobacter coli OXC7806	PubMLST	24604	827	0828	CL1	no	no	none
Campylobacter coli OXC7823	PubMLST	24620	4425	0828	CL1	no	no	none
Campylobacter coli OXC7824	PubMLST	24621	1585	0828	CL1	no	no	none
Campylobacter coli OXC7832	PubMLST	24629	825	0828	CL1	no	no	none
Campylobacter coli OXC7840	PubMLST	24637	828	0828	CL1	no	no	none
Campylobacter coli OXC7842	PubMLST	24639	962	0828	CL1	no	no	none
Campylobacter coli OXC7859	PubMLST	24994	962	0828	CL1	no	no	none
Campylobacter coli OXC7860	PubMLST	24995	962	0828	CL1	no	no	none
Campylobacter coli OXC7861	PubMLST	24996	829	0828	CL1	no	no	none
Campylobacter coli OXC7862	PubMLST	24997	4425	0828	CL1	no	no	none
Campylobacter coli OXC7873	PubMLST	25008	827	0828	CL1	no	no	none
Campylobacter coli OXC7876	PubMLST	25011	962	0828	CL1	no	no	none
Campylobacter coli OXC7883	PubMLST	25016	4425	0828	CL1	no	no	none
Campylobacter coli OXC7890	PubMLST	25021	828	0828	CL1	no	no	none
Campylobacter coli OXC7907	PubMLST	25037	4425	0828	CL1	no	no	none
Campylobacter coli OXC7908	PubMLST	25038	827	0828	CL1	no	no	none
Campylobacter coli OXC7913	PubMLST	25043	827	0828	CL1	no	no	none
Campylobacter coli OXC7920	PubMLST	25050	4425	0828	CL1	no	no	none
Campylobacter coli OXC7925	PubMLST	25054	872	0828	CL1	no	no	none
Campylobacter coli OXC7933	PubMLST	25061	860	0828	CL1	no	no	none
Campylobacter coli OXC7934	PubMLST	25062	860	0828	CL1	no	no	none
Campylobacter coli OXC7939	PubMLST	25064	829	0828	CL1	no	no	none
Campylobacter coli OXC7940	PubMLST	25065	829	0828	CL1	no	no	none
Campylobacter coli OXC7943	PubMLST	25066	4425	0828	CL1	no	no	none
Campylobacter coli OXC7944	PubMLST	25067	827	0828	CL1	no	no	none
Campylobacter coli OXC7952	PubMLST	25477	827	0828	CL1	no	no	none
Campylobacter coli OXC7982	PubMLST	25396	827	0828	CL1	no	no	none
Campylobacter coli OXC7985	PubMLST	25577	1614	0828	CL1	no	no	none
Campylobacter coli OXC7988	PubMLST	25400	1774	0828	CL1	no	no	none
Campylobacter coli OXC7995	PubMLST	25403	855	0828	CL1	no	no	none
Campylobacter coli OXC8003	PubMLST	25407	4425	0828	CL1	no	no	none
Campylobacter coli OXC8018	PubMLST	25586	825	0828	CL1	no	no	none
Campylobacter coli OXC8020	PubMLST	25587	827	0828	CL1	no	no	none
Campylobacter coli OXC8021	PubMLST	25495	827	0828	CL1	no	no	none
Campylobacter coli OXC8041	PubMLST	25506	827	0828	CL1	no	no	none
Campylobacter coli OXC8042	PubMLST	25507	827	0828	CL1	no	no	none
Campylobacter coli OXC8061	PubMLST	25603	827	0828	CL1	no	no	none
Campylobacter coli OXC8067	PubMLST	25604	855	0828	CL1	no	no	none
Campylobacter coli OXC8071	PubMLST	25605	827	0828	CL1	no	no	none
Campylobacter coli OXC8075	PubMLST	25609	825	0828	CL1	no	no	none
Campylobacter coli OXC8091	PubMLST	25524	827	0828	CL1	no	no	none
Campylobacter coli OXC8096	PubMLST	25525	827	0828	CL1	no	no	none
Campylobacter coli OXC8105	PubMLST	25531	827	0828	CL1	no	no	none
Campylobacter coli OXC8108	PubMLST	25613	827	0828	CL1	no	no	none
Campylobacter coli OXC8117	PubMLST	25618	825	0828	CL1	no	no	none
Campylobacter coli OXC8122	PubMLST	25540	827	0828	CL1	no	no	none
Campylobacter coli OXC8131	PubMLST	25559	1586	0828	CL1	no	no	none
Campylobacter coli OXC8142	PubMLST	25631	825	0828	CL1	no	no	none
Campylobacter coli OXC8144	PubMLST	25633	6543	0828	CL1	no	no	none
Campylobacter coli OXC8148	PubMLST	25636	830	0828	CL1	no	no	none
Campylobacter coli OXC8152	PubMLST	25638	872	0828	CL1	no	no	none
Campylobacter coli OXC8154	PubMLST	25447	1055	0828	CL1	no	no	none
Campylobacter coli OXC8156	PubMLST	25639	827	0828	CL1	no	no	none
Campylobacter coli OXC8161	PubMLST	25542	827	0828	CL1	no	no	none
Campylobacter coli OXC8178	PubMLST	25643	827	0828	CL1	no	no	none
Campylobacter coli OXC8179	PubMLST	25443	6979	0828	CL1	no	no	none
Campylobacter coli OXC8180	PubMLST	25644	827	0828	CL1	no	no	none
Campylobacter coli OXC8199	PubMLST	27904	825	0828	CL1	no	no	none
Campylobacter coli OXC8203	PubMLST	27908	829	0828	CL1	no	no	none
Campylobacter coli OXC8210	PubMLST	27915	827	0828	CL1	no	no	none
Campylobacter coli OXC8215	PubMLST	27918	2483	0828	CL1	no	no	none
Campylobacter coli OXC8219	PubMLST	27922	827	0828	CL1	no	no	none
Campylobacter coli OXC8224	PubMLST	27926	827	0828	CL1	no	no	none
Campylobacter coli OXC8240	PubMLST	27940	825	0828	CL1	no	no	none
Campylobacter coli OXC8243	PubMLST	27943	6984	0828	CL1	no	no	none
Campylobacter coli OXC8244	PubMLST	27944	6984	0828	CL1	no	no	none
Campylobacter coli OXC8248	PubMLST	27947	829	0828	CL1	no	no	none
Campylobacter coli OXC8261	PubMLST	27957	827	0828	CL1	no	no	none
Campylobacter coli OXC8272	PubMLST	27965	832	0828	CL1	no	no	none
Campylobacter coli OXC8274	PubMLST	27967	828	0828	CL1	no	no	none
Campylobacter coli OXC8293	PubMLST	27984	872	0828	CL1	no	no	none
Campylobacter coli OXC8294	PubMLST	27985	872	0828	CL1	no	no	none

Campylobacter coli OXC8340	PubMLST	28031	4291	0828	CL1	no	no	none
Campylobacter coli OXC8369	PubMLST	28055	827	0828	CL1	no	no	none
Campylobacter coli OXC8392	PubMLST	28585	4443	0828	CL1	no	no	none
Campylobacter coli OXC8402	PubMLST	28594	825	0828	CL1	no	no	none
Campylobacter coli OXC8416	PubMLST	28607	962	0828	CL1	no	no	none
Campylobacter coli OXC8423	PubMLST	28614	827	0828	CL1	no	no	none
Campylobacter coli OXC8434	PubMLST	28625	827	0828	CL1	no	no	none
Campylobacter coli OXC8444	PubMLST	28635	962	0828	CL1	no	no	none
Campylobacter coli OXC8447	PubMLST	28638	828	0828	CL1	no	no	none
Campylobacter coli OXC8458	PubMLST	28649	827	0828	CL1	no	no	none
Campylobacter coli OXC8462R	PubMLST	28655	829	0828	CL1	no	no	none
Campylobacter coli OXC8503	PubMLST	28694	860	0828	CL1	no	no	none
Campylobacter coli OXC8513	PubMLST	28704	860	0828	CL1	no	no	none
Campylobacter coli OXC8521	PubMLST	28712	825	0828	CL1	no	no	none
Campylobacter coli OXC8542	PubMLST	28732	1614	0828	CL1	no	no	none
Campylobacter coli OXC8550	PubMLST	28739	1614	0828	CL1	no	no	none
Campylobacter coli OXC8552	PubMLST	28741	1595	0828	CL1	no	no	none
Campylobacter coli OXC8557	PubMLST	28746	872	0828	CL1	no	no	none
Campylobacter coli OXC8565	PubMLST	28904	831	0828	CL1	no	no	none
Campylobacter coli OXC8574	PubMLST	28911	832	0828	CL1	no	no	none
Campylobacter coli OXC8628	PubMLST	28991	825	0828	CL1	no	no	none
Campylobacter coli OXC8648	PubMLST	28962	825	0828	CL1	no	no	none
Campylobacter coli OXC8654	PubMLST	28968	855	0828	CL1	no	no	none
Campylobacter coli OXC8655	PubMLST	28969	855	0828	CL1	no	no	none
Campylobacter coli OXC8663	PubMLST	28998	7304	0828	CL1	no	no	none
Campylobacter coli OXC8667	PubMLST	29001	860	0828	CL1	no	no	none
Campylobacter coli OXC8679	PubMLST	29011	828	0828	CL1	no	no	none
Campylobacter coli OXC8688	PubMLST	29020	1055	0828	CL1	no	no	none
Campylobacter coli OXC8696	PubMLST	29027	898	0828	CL1	no	no	none
Campylobacter coli OXC8697	PubMLST	29028	2273	0828	CL1	no	no	none
Campylobacter coli OXC8698	PubMLST	29029	827	0828	CL1	no	no	none
Campylobacter coli OXC8701	PubMLST	29030	898	0828	CL1	no	no	none
Campylobacter coli OXC8705	PubMLST	29034	1055	0828	CL1	no	no	none
Campylobacter coli OXC8719	PubMLST	29046	4425	0828	CL1	no	no	none
Campylobacter coli OXC8720	PubMLST	29081	827	0828	CL1	no	no	none
Campylobacter coli OXC8724	PubMLST	29082	828	0828	CL1	no	no	none
Campylobacter coli OXC8730	PubMLST	29055	6543	0828	CL1	no	no	none
Campylobacter coli OXC8739	PubMLST	29064	3020	0828	CL1	no	no	none
Campylobacter coli OXC8740	PubMLST	28897	827	0828	CL1	no	no	none
Campylobacter coli OXC8748	PubMLST	30439	855	0828	CL1	no	no	none
Campylobacter coli OXC8750	PubMLST	30441	827	0828	CL1	no	no	none
Campylobacter coli OXC8751	PubMLST	30442	827	0828	CL1	no	no	none
Campylobacter coli OXC8752	PubMLST	30443	827	0828	CL1	no	no	none
Campylobacter coli OXC8753	PubMLST	30444	827	0828	CL1	no	no	none
Campylobacter coli OXC8763	PubMLST	30453	827	0828	CL1	no	no	none
Campylobacter coli OXC8788	PubMLST	30477	827	0828	CL1	no	no	none
Campylobacter coli OXC8790	PubMLST	30479	827	0828	CL1	no	no	none
Campylobacter coli OXC8804	PubMLST	30493	2301	0828	CL1	no	no	none
Campylobacter coli OXC8807	PubMLST	30496	825	0828	CL1	no	no	none
Campylobacter coli OXC8814	PubMLST	30502	825	0828	CL1	no	no	none
Campylobacter coli OXC8815	PubMLST	30503	1068	0828	CL1	no	no	none
Campylobacter coli OXC8822	PubMLST	30510	827	0828	CL1	no	no	none
Campylobacter coli OXC8847	PubMLST	30533	872	0828	CL1	no	no	none
Campylobacter coli OXC8851	PubMLST	30537	827	0828	CL1	no	no	none
Campylobacter coli OXC8860	PubMLST	30546	825	0828	CL1	no	no	none
Campylobacter coli OXC8872	PubMLST	30558	825	0828	CL1	no	no	none
Campylobacter coli OXC8887	PubMLST	30571	825	0828	CL1	no	no	none
Campylobacter coli OXC8942	PubMLST	30625	827	0828	CL1	no	no	none
Campylobacter coli OXC8952	PubMLST	30635	827	0828	CL1	no	no	none
Campylobacter coli OXC8954	PubMLST	30637	827	0828	CL1	no	no	none
Campylobacter coli OXC8957	PubMLST	30640	829	0828	CL1	no	no	none
Campylobacter coli OXC8958	PubMLST	30641	827	0828	CL1	no	no	none
Campylobacter coli OXC8960	PubMLST	30642	855	0828	CL1	no	no	none
Campylobacter coli OXC8965	PubMLST	30647	855	0828	CL1	no	no	none
Campylobacter coli OXC8981	PubMLST	30663	825	0828	CL1	no	no	none
Campylobacter coli PW1	PubMLST	24256	887	0828	CL1	no	no	none
Campylobacter coli RM5611	Genbank/EMBL/DDBJ	CP007179	1068	0828	CL1	no	no	none
Campylobacter coli Z156	Genbank/EMBL/DDBJ	AINX01	854	0828	CL1	no	no	none
Campylobacter coli Z163	Genbank/EMBL/DDBJ	AIMK01	3336	0828	CL1	no	no	none
Campylobacter coli 132-6	Genbank/EMBL/DDBJ	AINA01	3861	0000	CL1	no	yes	yes
Campylobacter coli 151-9	Genbank/EMBL/DDBJ	AINQ01	1102	0000	CL1	no	yes	yes
Campylobacter coli 7--1	Genbank/EMBL/DDBJ	AIMZ01	3860	0000	CL1	no	yes	yes
Campylobacter coli Dg277	PubMLST	26045	1143	0000	CL1	no	yes	yes
Campylobacter coli OXC6551	PubMLST	12886	5755	0000	CL1	no	yes	yes
Campylobacter coli OXC8567	PubMLST	28906	1680	0000	CL1	no	yes	yes
Campylobacter coli 1098	Genbank/EMBL/DDBJ	AIMW01	1104	0828	CL1	no	yes	yes
Campylobacter coli 111-3	Genbank/EMBL/DDBJ	AIMI01	1467	0828	CL1	no	yes	yes
Campylobacter coli 1909	Genbank/EMBL/DDBJ	AINC01	1104	0828	CL1	no	yes	yes
Campylobacter coli 1948	Genbank/EMBL/DDBJ	AINE01	1104	0828	CL1	no	yes	yes
Campylobacter coli 1961	Genbank/EMBL/DDBJ	AING01	1104	0828	CL1	no	yes	yes
Campylobacter coli 2692	Genbank/EMBL/DDBJ	AIMQ01	860	0828	CL1	no	yes	yes
Campylobacter coli 2698	Genbank/EMBL/DDBJ	AIMR01	829	0828	CL1	no	yes	yes
Campylobacter coli 59-2	Genbank/EMBL/DDBJ	AIND01	890	0828	CL1	no	yes	yes
Campylobacter coli 80352	Genbank/EMBL/DDBJ	AIMT01	1017	0828	CL1	no	yes	yes
Campylobacter coli Dg100a	PubMLST	26028	1464	0828	CL1	no	yes	yes
Campylobacter coli Dg319	PubMLST	26059	1127	0828	CL1	no	yes	yes
Campylobacter coli Dg46b	PubMLST	25938	872	0828	CL1	no	yes	yes
Campylobacter coli H56	Genbank/EMBL/DDBJ	AINW01	1096	0828	CL1	no	yes	yes
Campylobacter coli K3	Genbank/EMBL/DDBJ	AYKN01	7123	0828	CL1	no	yes	yes
Campylobacter coli OXC5257	PubMLST	24835	854	0828	CL1	no	yes	yes
Campylobacter coli OXC6559	PubMLST	12894	5757	0828	CL1	no	yes	yes
Campylobacter coli OXC6725	PubMLST	18282	1096	0828	CL1	no	yes	yes

Campylobacter coli OXC7411	PubMLST	23946	6267	0828	CL1	no	yes	yes
Campylobacter coli OXC7612	PubMLST	24541	854	0828	CL1	no	yes	yes
Campylobacter coli OXC8728	PubMLST	29053	827	0828	CL1	no	yes	yes
Campylobacter coli 1957	Genbank/EMBL/DDBJ	AINF01	2698	0828	CL1	no	yes	no
Campylobacter coli OXC6094	PubMLST	29302	1096	0828	CL1	no	yes	no
Campylobacter coli OXC6744	PubMLST	18301	5813	0828	CL1	no	yes	no
Campylobacter coli 8808	PubMLST	24266	N/A	0000	CL2	yes	no	yes
Campylobacter coli dfvf1656	PubMLST	24269	1572	0000	CL2	yes	no	yes
Campylobacter coli E120587CC_S8	PubMLST	25924	N/A	0000	CL2	yes	no	yes
Campylobacter coli E120613CC_S3	PubMLST	25926	6936	0000	CL2	yes	no	yes
Campylobacter coli 2544	PubMLST	24267	2326	0000	CL2	yes	no	no
Campylobacter coli 6873	PubMLST	24268	2016	0000	CL2	yes	no	no
Campylobacter coli E120595CC_S4	PubMLST	25925	N/A	0000	CL2	yes	no	no
Campylobacter coli 76339	Genbank/EMBL/DDBJ	HG326877	N/A	0000	CL3	Cas9 only	no	yes
Campylobacter coli 8096	PubMLST	24271	7243	0000	CL3	Cas9 only	no	yes
Campylobacter coli dfvf1912	PubMLST	24272	1576	0000	CL3	Cas9 only	no	yes
Campylobacter coli E120586CC_S2	PubMLST	25923	6935	0000	CL3	Cas9 only	no	yes
Campylobacter coli OXC7241	PubMLST	22283	6698	0000	CL3	Cas9 only	no	yes
Campylobacter coli OXC7243	PubMLST	22285	6698	0000	CL3	Cas9 only	no	yes
Campylobacter coli OXC7244	PubMLST	22286	6698	0000	CL3	Cas9 only	no	yes
Campylobacter coli 4944	PubMLST	24273	1670	0000	CL3	no	no	none
Campylobacter coli E120061CC_S5	PubMLST	25917	6931	0000	CL3	no	no	none
Campylobacter coli E120064CC_S6	PubMLST	25918	6932	0000	CL3	no	no	none
Campylobacter coli E120065CC_S7	PubMLST	25919	6933	0000	CL3	no	no	none
Campylobacter coli E120529CC_S1	PubMLST	25922	6288	0000	CL3	no	no	none
Campylobacter coli FSA05.280042	PubMLST	24270	1992	0000	CL3	no	no	none
Campylobacter coli OXC7653	PubMLST	24908	6975	0000	CL3	no	no	none
Campylobacter coli OXC8422	PubMLST	28613	7181	0000	CL3	no	no	none
Campylobacter coli OXC8548	PubMLST	28737	7183	0000	CL3	no	no	none
Campylobacter coli RM4931	PubMLST	24274	1643	0000	CL3	no	no	none
Campylobacter coli OXC5681	PubMLST	21427	6133	0000	CL3	yes	no	yes
Campylobacter coli Duck323_8866	PubMLST	24263	3311	0000	none	no	no	none
Campylobacter coli H142940463	PubMLST	2604	7346	0000	none	no	no	none
Campylobacter coli H143040419	PubMLST	2606	1764	0000	none	no	no	none
Campylobacter coli OXC6659	PubMLST	18216	1243	0000	none	yes	no	yes

Campylobacter jejuni

Isolate / Strain	Source (a)	Accession/ID (b)	MLST ST (c)	clonal complex (c)	Clade (d)	Cc CRISPR (e)	Cj CRISPR (e)	full length Cas9 (f)
Campylobacter jejuni OXC4676	PubMLST	24111	4359	0000	jejuni	no	no	none
Campylobacter jejuni OXC4676_2	PubMLST	24112	4359	0000	jejuni	no	no	none
Campylobacter jejuni OXC6327	PubMLST	16124	3534	0000	jejuni	no	no	none
Campylobacter jejuni OXC6569	PubMLST	12904	5597	0000	jejuni	no	no	none
Campylobacter jejuni OXC7055	PubMLST	22240	N/A	0000	jejuni	no	no	none
Campylobacter jejuni OXC7494	PubMLST	24022	6756	0000	jejuni	no	no	none
Campylobacter jejuni 81-176	Genbank/EMBL/DDBJ	NC_005012	604	0042	jejuni	no	no	none
Campylobacter jejuni Dg146	PubMLST	25949	42	0042	jejuni	no	no	none
Campylobacter jejuni Dg153	PubMLST	24195	42	0042	jejuni	no	no	none
Campylobacter jejuni Dg209	PubMLST	25981	42	0042	jejuni	no	no	none
Campylobacter jejuni Dg214	PubMLST	26035	42	0042	jejuni	no	no	none
Campylobacter jejuni Dg241	PubMLST	25942	42	0042	jejuni	no	no	none
Campylobacter jejuni Dg245	PubMLST	26056	42	0042	jejuni	no	no	none
Campylobacter jejuni Dg247	PubMLST	25957	42	0042	jejuni	no	no	none
Campylobacter jejuni Dg250	PubMLST	25996	42	0042	jejuni	no	no	none
Campylobacter jejuni Dg364	PubMLST	26033	42	0042	jejuni	no	no	none
Campylobacter jejuni H121240497	PubMLST	2630	42	0042	jejuni	no	no	none
Campylobacter jejuni H121240500	PubMLST	2631	42	0042	jejuni	no	no	none
Campylobacter jejuni H121240501	PubMLST	2632	42	0042	jejuni	no	no	none
Campylobacter jejuni H122720461	PubMLST	2644	42	0042	jejuni	no	no	none
Campylobacter jejuni HM301572	PubMLST	24208	42	0042	jejuni	no	no	none
Campylobacter jejuni NC15_R	PubMLST	24322	42	0042	jejuni	no	no	none
Campylobacter jejuni OXC4625	PubMLST	24077	4016	0042	jejuni	no	no	none
Campylobacter jejuni OXC4768	PubMLST	18461	42	0042	jejuni	no	no	none
Campylobacter jejuni OXC4866	PubMLST	22177	42	0042	jejuni	no	no	none
Campylobacter jejuni OXC4881	PubMLST	22186	42	0042	jejuni	no	no	none
Campylobacter jejuni OXC4927	PubMLST	25092	42	0042	jejuni	no	no	none
Campylobacter jejuni OXC5013	PubMLST	24686	42	0042	jejuni	no	no	none
Campylobacter jejuni OXC5034	PubMLST	24701	42	0042	jejuni	no	no	none
Campylobacter jejuni OXC5037	PubMLST	24703	7162	0042	jejuni	no	no	none
Campylobacter jejuni OXC5392	PubMLST	21352	42	0042	jejuni	no	no	none
Campylobacter jejuni OXC5785	PubMLST	23537	42	0042	jejuni	no	no	none
Campylobacter jejuni OXC5890	PubMLST	23621	42	0042	jejuni	no	no	none
Campylobacter jejuni OXC6333	PubMLST	16130	42	0042	jejuni	no	no	none
Campylobacter jejuni OXC6350	PubMLST	16147	42	0042	jejuni	no	no	none
Campylobacter jejuni OXC6388	PubMLST	16185	42	0042	jejuni	no	no	none
Campylobacter jejuni OXC6409	PubMLST	16204	42	0042	jejuni	no	no	none
Campylobacter jejuni OXC6415	PubMLST	16210	42	0042	jejuni	no	no	none
Campylobacter jejuni OXC6417	PubMLST	16212	42	0042	jejuni	no	no	none
Campylobacter jejuni OXC6512	PubMLST	16304	42	0042	jejuni	no	no	none
Campylobacter jejuni OXC6827	PubMLST	18384	42	0042	jejuni	no	no	none
Campylobacter jejuni OXC7052	PubMLST	22237	42	0042	jejuni	no	no	none
Campylobacter jejuni OXC7107	PubMLST	21189	42	0042	jejuni	no	no	none
Campylobacter jejuni OXC7133	PubMLST	21214	42	0042	jejuni	no	no	none
Campylobacter jejuni OXC7156	PubMLST	21237	42	0042	jejuni	no	no	none
Campylobacter jejuni OXC7191	PubMLST	22682	42	0042	jejuni	no	no	none
Campylobacter jejuni OXC7194	PubMLST	22685	42	0042	jejuni	no	no	none
Campylobacter jejuni OXC7207	PubMLST	22698	42	0042	jejuni	no	no	none
Campylobacter jejuni OXC7215	PubMLST	22706	42	0042	jejuni	no	no	none
Campylobacter jejuni OXC7248	PubMLST	22290	42	0042	jejuni	no	no	none
Campylobacter jejuni OXC7265	PubMLST	22306	42	0042	jejuni	no	no	none
Campylobacter jejuni OXC7324	PubMLST	22363	42	0042	jejuni	no	no	none
Campylobacter jejuni OXC7400	PubMLST	23936	42	0042	jejuni	no	no	none
Campylobacter jejuni OXC7416	PubMLST	23950	42	0042	jejuni	no	no	none

Campylobacter jejuni OXC7616	PubMLST	24545	42	0042	jejuni	no	no	none
Campylobacter jejuni OXC7705	PubMLST	24952	42	0042	jejuni	no	no	none
Campylobacter jejuni OXC7710	PubMLST	25537	42	0042	jejuni	no	no	none
Campylobacter jejuni OXC7779	PubMLST	24586	42	0042	jejuni	no	no	none
Campylobacter jejuni OXC7898	PubMLST	25551	42	0042	jejuni	no	no	none
Campylobacter jejuni OXC7991	PubMLST	25401	42	0042	jejuni	no	no	none
Campylobacter jejuni OXC8058	PubMLST	25511	42	0042	jejuni	no	no	none
Campylobacter jejuni OXC8069	PubMLST	25418	42	0042	jejuni	no	no	none
Campylobacter jejuni OXC8153	PubMLST	25446	42	0042	jejuni	no	no	none
Campylobacter jejuni OXC8289	PubMLST	27980	42	0042	jejuni	no	no	none
Campylobacter jejuni OXC8303	PubMLST	27994	459	0042	jejuni	no	no	none
Campylobacter jejuni OXC8383	PubMLST	28576	42	0042	jejuni	no	no	none
Campylobacter jejuni OXC8410	PubMLST	28601	42	0042	jejuni	no	no	none
Campylobacter jejuni OXC8527	PubMLST	28718	42	0042	jejuni	no	no	none
Campylobacter jejuni OXC8555	PubMLST	28744	42	0042	jejuni	no	no	none
Campylobacter jejuni OXC8659	PubMLST	28994	42	0042	jejuni	no	no	none
Campylobacter jejuni OXC8870	PubMLST	30556	42	0042	jejuni	no	no	none
Campylobacter jejuni OXC8923	PubMLST	30606	42	0042	jejuni	no	no	none
Campylobacter jejuni OXC8931	PubMLST	30614	1751	0042	jejuni	no	no	none
Campylobacter jejuni OXC9011	PubMLST	30691	42	0042	jejuni	no	no	none
Campylobacter jejuni OXC5941	PubMLST	23683	45	0045	jejuni	no	no	none
Campylobacter jejuni OXC6540	PubMLST	16332	587	0362	jejuni	no	no	none
Campylobacter jejuni OXC8644	PubMLST	29075	658	0658	jejuni	no	no	none
Campylobacter jejuni 1336	Genbank/EMBL/DDBJ	ADGL01	841	0000	jejuni	no	yes	no
Campylobacter jejuni 2008-894	Genbank/EMBL/DDBJ	AIOQ01	1962	0000	jejuni	no	yes	no
Campylobacter jejuni 305	Genbank/EMBL/DDBJ	ADHL01	N/A	0000	jejuni	no	yes	no
Campylobacter jejuni 414	Genbank/EMBL/DDBJ	ADGM01	3704	0000	jejuni	no	yes	no
Campylobacter jejuni BV1336	PubMLST	28292	841	0000	jejuni	no	yes	no
Campylobacter jejuni BV414	PubMLST	28291	3704	0000	jejuni	no	yes	no
Campylobacter jejuni Dg334	PubMLST	25946	7279	0000	jejuni	no	yes	no
Campylobacter jejuni Dg356	PubMLST	24192	5130	0000	jejuni	no	yes	no
Campylobacter jejuni Dg63a	PubMLST	25975	7258	0000	jejuni	no	yes	no
Campylobacter jejuni Dg69	PubMLST	25962	5130	0000	jejuni	no	yes	no
Campylobacter jejuni Dg80a	PubMLST	25987	5130	0000	jejuni	no	yes	no
Campylobacter jejuni Dg81	PubMLST	25995	5130	0000	jejuni	no	yes	no
Campylobacter jejuni Dg86	PubMLST	26004	5130	0000	jejuni	no	yes	no
Campylobacter jejuni E120052CJ_S11	PubMLST	25915	N/A	0000	jejuni	no	yes	no
Campylobacter jejuni E120399CJ_S10	PubMLST	25921	6934	0000	jejuni	no	yes	no
Campylobacter jejuni K1	Genbank/EMBL/DDBJ	AUUO01	3630	0000	jejuni	no	yes	no
Campylobacter jejuni LMG 23216	Genbank/EMBL/DDBJ	AIOA01	4835	0000	jejuni	no	yes	no
Campylobacter jejuni LMG 23223	Genbank/EMBL/DDBJ	AIOC01	791	0000	jejuni	no	yes	no
Campylobacter jejuni OXC4952	PubMLST	25467	3573	0000	jejuni	no	yes	no
Campylobacter jejuni OXC4962	PubMLST	25372	448	0000	jejuni	no	yes	no
Campylobacter jejuni OXC5191	PubMLST	24786	877	0000	jejuni	no	yes	no
Campylobacter jejuni OXC5212R	PubMLST	29893	N/A	0000	jejuni	no	yes	no
Campylobacter jejuni OXC5246	PubMLST	24827	N/A	0000	jejuni	no	yes	no
Campylobacter jejuni OXC5259	PubMLST	24836	5982	0000	jejuni	no	yes	no
Campylobacter jejuni OXC5426	PubMLST	21375	877	0000	jejuni	no	yes	no
Campylobacter jejuni OXC5457	PubMLST	21395	5142	0000	jejuni	no	yes	no
Campylobacter jejuni OXC5622	PubMLST	23510	1526	0000	jejuni	no	yes	no
Campylobacter jejuni OXC5769	PubMLST	21490	6134	0000	jejuni	no	yes	no
Campylobacter jejuni OXC6320	PubMLST	16117	986	0000	jejuni	no	yes	no
Campylobacter jejuni OXC6336	PubMLST	16133	986	0000	jejuni	no	yes	no
Campylobacter jejuni OXC6408	PubMLST	16203	585	0000	jejuni	no	yes	no
Campylobacter jejuni OXC6556	PubMLST	12891	4811	0000	jejuni	no	yes	no
Campylobacter jejuni OXC6631	PubMLST	16380	2401	0000	jejuni	no	yes	no
Campylobacter jejuni OXC6687	PubMLST	18244	N/A	0000	jejuni	no	yes	no
Campylobacter jejuni OXC6699	PubMLST	18256	1071	0000	jejuni	no	yes	no
Campylobacter jejuni OXC6832	PubMLST	18388	436	0000	jejuni	no	yes	no
Campylobacter jejuni OXC7076	PubMLST	22260	2491	0000	jejuni	no	yes	no
Campylobacter jejuni OXC7077	PubMLST	22261	3453	0000	jejuni	no	yes	no
Campylobacter jejuni OXC7125	PubMLST	21207	2133	0000	jejuni	no	yes	no
Campylobacter jejuni OXC7145	PubMLST	21226	6138	0000	jejuni	no	yes	no
Campylobacter jejuni OXC7149	PubMLST	21230	877	0000	jejuni	no	yes	no
Campylobacter jejuni OXC7232	PubMLST	22722	1080	0000	jejuni	no	yes	no
Campylobacter jejuni OXC7311	PubMLST	22352	6699	0000	jejuni	no	yes	no
Campylobacter jejuni OXC7333	PubMLST	22371	448	0000	jejuni	no	yes	no
Campylobacter jejuni OXC7574	PubMLST	24511	N/A	0000	jejuni	no	yes	no
Campylobacter jejuni OXC8113	PubMLST	25533	6980	0000	jejuni	no	yes	no
Campylobacter jejuni OXC8194	PubMLST	27899	N/A	0000	jejuni	no	yes	no
Campylobacter jejuni OXC8323	PubMLST	28014	5155	0000	jejuni	no	yes	no
Campylobacter jejuni OXC8339	PubMLST	28030	3573	0000	jejuni	no	yes	no
Campylobacter jejuni OXC8385	PubMLST	28578	N/A	0000	jejuni	no	yes	no
Campylobacter jejuni OXC8499	PubMLST	28690	2133	0000	jejuni	no	yes	no
Campylobacter jejuni OXC8523	PubMLST	28714	7177	0000	jejuni	no	yes	no
Campylobacter jejuni OXC8662	PubMLST	28997	3630	0000	jejuni	no	yes	no
Campylobacter jejuni OXC8986	PubMLST	30668	N/A	0000	jejuni	no	yes	no
Campylobacter jejuni X	Genbank/EMBL/DDBJ	AVFM02	N/A	0000	jejuni	no	yes	no
Campylobacter jejuni ARI_1434_R	PubMLST	24315	5018	0021	jejuni	no	yes	no
Campylobacter jejuni CAMSA2024	PubMLST	22828	21	0021	jejuni	no	yes	no
Campylobacter jejuni CAMSA260	PubMLST	22786	21	0021	jejuni	no	yes	no
Campylobacter jejuni CAMSA341	PubMLST	22794	21	0021	jejuni	no	yes	no
Campylobacter jejuni G1	Genbank/EMBL/DDBJ	JRLT01	44	0021	jejuni	no	yes	no
Campylobacter jejuni H140740343	PubMLST	29452	50	0021	jejuni	no	yes	no
Campylobacter jejuni H140940806	PubMLST	29453	50	0021	jejuni	no	yes	no
Campylobacter jejuni H142940464	PubMLST	2605	50	0021	jejuni	no	yes	no
Campylobacter jejuni JCM 2013	Genbank/EMBL/DDBJ	BALI01	7244	0021	jejuni	no	yes	no
Campylobacter jejuni Le_204R	Genbank/EMBL/DDBJ	CCDB01	50	0021	jejuni	no	yes	no
Campylobacter jejuni Le_755	Genbank/EMBL/DDBJ	CCDC01	50	0021	jejuni	no	yes	no
Campylobacter jejuni Ma_1	Genbank/EMBL/DDBJ	CCCZ01	50	0021	jejuni	no	yes	no
Campylobacter jejuni Ma_B	Genbank/EMBL/DDBJ	CCDD01	50	0021	jejuni	no	yes	no
Campylobacter jejuni OXC4568	PubMLST	24045	148	0021	jejuni	no	yes	no

Campylobacter jejuni OXC4776	PubMLST	18466	50	0021	jejuni	no	yes	no
Campylobacter jejuni OXC4801	PubMLST	22141	50	0021	jejuni	no	yes	no
Campylobacter jejuni OXC5045	PubMLST	24708	50	0021	jejuni	no	yes	no
Campylobacter jejuni OXC5094	PubMLST	24733	50	0021	jejuni	no	yes	no
Campylobacter jejuni OXC5127	PubMLST	24749	148	0021	jejuni	no	yes	no
Campylobacter jejuni OXC5166	PubMLST	24767	21	0021	jejuni	no	yes	no
Campylobacter jejuni OXC5168	PubMLST	24769	262	0021	jejuni	no	yes	no
Campylobacter jejuni OXC5333	PubMLST	21317	50	0021	jejuni	no	yes	no
Campylobacter jejuni OXC5397	PubMLST	21357	5242	0021	jejuni	no	yes	no
Campylobacter jejuni OXC5413	PubMLST	21367	21	0021	jejuni	no	yes	no
Campylobacter jejuni OXC5431	PubMLST	21378	5018	0021	jejuni	no	yes	no
Campylobacter jejuni OXC5664	PubMLST	21414	50	0021	jejuni	no	yes	no
Campylobacter jejuni OXC5713	PubMLST	21450	21	0021	jejuni	no	yes	no
Campylobacter jejuni OXC5724	PubMLST	21456	19	0021	jejuni	no	yes	no
Campylobacter jejuni OXC6340	PubMLST	16137	262	0021	jejuni	no	yes	no
Campylobacter jejuni OXC6355	PubMLST	16152	19	0021	jejuni	no	yes	no
Campylobacter jejuni OXC6429	PubMLST	16224	5018	0021	jejuni	no	yes	no
Campylobacter jejuni OXC6464	PubMLST	16256	5018	0021	jejuni	no	yes	no
Campylobacter jejuni OXC6505	PubMLST	16297	141	0021	jejuni	no	yes	no
Campylobacter jejuni OXC6521	PubMLST	16313	47	0021	jejuni	no	yes	no
Campylobacter jejuni OXC6562	PubMLST	12897	21	0021	jejuni	no	yes	no
Campylobacter jejuni OXC6633	PubMLST	16382	53	0021	jejuni	no	yes	no
Campylobacter jejuni OXC6672	PubMLST	18229	47	0021	jejuni	no	yes	no
Campylobacter jejuni OXC7140	PubMLST	21221	21	0021	jejuni	no	yes	no
Campylobacter jejuni OXC7312	PubMLST	22353	190	0021	jejuni	no	yes	no
Campylobacter jejuni OXC7420	PubMLST	23954	19	0021	jejuni	no	yes	no
Campylobacter jejuni OXC7508	PubMLST	24034	21	0021	jejuni	no	yes	no
Campylobacter jejuni OXC7523	PubMLST	25405	19	0021	jejuni	no	yes	no
Campylobacter jejuni OXC7538	PubMLST	24481	5018	0021	jejuni	no	yes	no
Campylobacter jejuni OXC7543	PubMLST	24484	19	0021	jejuni	no	yes	no
Campylobacter jejuni OXC7596	PubMLST	24528	19	0021	jejuni	no	yes	no
Campylobacter jejuni OXC7778	PubMLST	24585	3633	0021	jejuni	no	yes	no
Campylobacter jejuni OXC7797	PubMLST	24598	47	0021	jejuni	no	yes	no
Campylobacter jejuni OXC7815	PubMLST	24613	47	0021	jejuni	no	yes	no
Campylobacter jejuni OXC7897	PubMLST	25028	19	0021	jejuni	no	yes	no
Campylobacter jejuni OXC7955	PubMLST	25573	6894	0021	jejuni	no	yes	no
Campylobacter jejuni OXC7960	PubMLST	25378	19	0021	jejuni	no	yes	no
Campylobacter jejuni OXC8034	PubMLST	25591	5018	0021	jejuni	no	yes	no
Campylobacter jejuni OXC8035	PubMLST	25503	5018	0021	jejuni	no	yes	no
Campylobacter jejuni OXC8273	PubMLST	27966	3574	0021	jejuni	no	yes	no
Campylobacter jejuni OXC8338	PubMLST	28029	141	0021	jejuni	no	yes	no
Campylobacter jejuni OXC8451	PubMLST	28642	883	0021	jejuni	no	yes	no
Campylobacter jejuni OXC8529	PubMLST	28720	262	0021	jejuni	no	yes	no
Campylobacter jejuni OXC8598	PubMLST	28983	19	0021	jejuni	no	yes	no
Campylobacter jejuni OXC8625R	PubMLST	29083	21	0021	jejuni	no	yes	no
Campylobacter jejuni OXC8630	PubMLST	28993	19	0021	jejuni	no	yes	no
Campylobacter jejuni OXC8784	PubMLST	30473	19	0021	jejuni	no	yes	no
Campylobacter jejuni OXC8848	PubMLST	30534	53	0021	jejuni	no	yes	no
Campylobacter jejuni OXC8875	PubMLST	30561	53	0021	jejuni	no	yes	no
Campylobacter jejuni OXC8921	PubMLST	30604	262	0021	jejuni	no	yes	no
Campylobacter jejuni OXC8933	PubMLST	30616	21	0021	jejuni	no	yes	no
Campylobacter jejuni Po_1	Genbank/EMBL/DDBJ	CCDA01	50	0021	jejuni	no	yes	no
Campylobacter jejuni Po_2	Genbank/EMBL/DDBJ	CCDE01	50	0021	jejuni	no	yes	no
Campylobacter jejuni 1997-11	Genbank/EMBL/DDBJ	AIOZ01	22	0022	jejuni	no	yes	no
Campylobacter jejuni BJ-CJD101	Genbank/EMBL/DDBJ	ARWV01	3652	0022	jejuni	no	yes	no
Campylobacter jejuni OXC7048	PubMLST	22233	22	0022	jejuni	no	yes	no
Campylobacter jejuni 327	Genbank/EMBL/DDBJ	ADHM01	230	0045	jejuni	no	yes	no
Campylobacter jejuni 55037	Genbank/EMBL/DDBJ	AIOH01	45	0045	jejuni	no	yes	no
Campylobacter jejuni Dg283	PubMLST	25960	1326	0045	jejuni	no	yes	no
Campylobacter jejuni M1	Genbank/EMBL/DDBJ	CP001900	137	0045	jejuni	no	yes	no
Campylobacter jejuni OXC4736	PubMLST	18439	1326	0045	jejuni	no	yes	no
Campylobacter jejuni OXC5171	PubMLST	24772	45	0045	jejuni	no	yes	no
Campylobacter jejuni OXC5330	PubMLST	21314	1003	0045	jejuni	no	yes	no
Campylobacter jejuni OXC5343	PubMLST	21325	2109	0045	jejuni	no	yes	no
Campylobacter jejuni OXC6358	PubMLST	16155	233	0045	jejuni	no	yes	no
Campylobacter jejuni OXC6614	PubMLST	16364	233	0045	jejuni	no	yes	no
Campylobacter jejuni OXC6938	PubMLST	21112	2109	0045	jejuni	no	yes	no
Campylobacter jejuni OXC7102	PubMLST	21184	25	0045	jejuni	no	yes	no
Campylobacter jejuni OXC7371	PubMLST	23909	2109	0045	jejuni	no	yes	no
Campylobacter jejuni OXC7376	PubMLST	23914	2109	0045	jejuni	no	yes	no
Campylobacter jejuni OXC7584	PubMLST	24518	583	0045	jejuni	no	yes	no
Campylobacter jejuni OXC7881	PubMLST	25014	137	0045	jejuni	no	yes	no
Campylobacter jejuni OXC8026	PubMLST	25589	45	0045	jejuni	no	yes	no
Campylobacter jejuni OXC8195	PubMLST	27900	45	0045	jejuni	no	yes	no
Campylobacter jejuni OXC8336	PubMLST	28027	45	0045	jejuni	no	yes	no
Campylobacter jejuni OXC8372	PubMLST	27872	2109	0045	jejuni	no	yes	no
Campylobacter jejuni OXC8375	PubMLST	27875	2109	0045	jejuni	no	yes	no
Campylobacter jejuni OXC8474	PubMLST	28667	233	0045	jejuni	no	yes	no
Campylobacter jejuni Dg15b	PubMLST	25970	48	0048	jejuni	no	yes	no
Campylobacter jejuni H121320384	PubMLST	2633	48	0048	jejuni	no	yes	no
Campylobacter jejuni LMG 23218	Genbank/EMBL/DDBJ	AIOB01	48	0048	jejuni	no	yes	no
Campylobacter jejuni OXC4582	PubMLST	24056	48	0048	jejuni	no	yes	no
Campylobacter jejuni OXC4629	PubMLST	24079	48	0048	jejuni	no	yes	no
Campylobacter jejuni OXC4630	PubMLST	24080	48	0048	jejuni	no	yes	no
Campylobacter jejuni OXC4734	PubMLST	18437	48	0048	jejuni	no	yes	no
Campylobacter jejuni OXC4735	PubMLST	18438	48	0048	jejuni	no	yes	no
Campylobacter jejuni OXC4792	PubMLST	22135	5359	0048	jejuni	no	yes	no
Campylobacter jejuni OXC4799	PubMLST	22140	5359	0048	jejuni	no	yes	no
Campylobacter jejuni OXC4803	PubMLST	22143	48	0048	jejuni	no	yes	no
Campylobacter jejuni OXC4832	PubMLST	22157	48	0048	jejuni	no	yes	no
Campylobacter jejuni OXC4840	PubMLST	22159	48	0048	jejuni	no	yes	no
Campylobacter jejuni OXC4851	PubMLST	22164	48	0048	jejuni	no	yes	no

Campylobacter jejuni OXC4852	PubMLST	22165	48	0048	jejuni	no	yes	no
Campylobacter jejuni OXC4872	PubMLST	22180	48	0048	jejuni	no	yes	no
Campylobacter jejuni OXC4895	PubMLST	24460	48	0048	jejuni	no	yes	no
Campylobacter jejuni OXC5028	PubMLST	24696	48	0048	jejuni	no	yes	no
Campylobacter jejuni OXC5035	PubMLST	24702	48	0048	jejuni	no	yes	no
Campylobacter jejuni OXC5038	PubMLST	24704	48	0048	jejuni	no	yes	no
Campylobacter jejuni OXC5183	PubMLST	24779	48	0048	jejuni	no	yes	no
Campylobacter jejuni OXC5228	PubMLST	24814	48	0048	jejuni	no	yes	no
Campylobacter jejuni OXC5244	PubMLST	24826	48	0048	jejuni	no	yes	no
Campylobacter jejuni OXC5247	PubMLST	24828	48	0048	jejuni	no	yes	no
Campylobacter jejuni OXC5304	PubMLST	25471	48	0048	jejuni	no	yes	no
Campylobacter jejuni OXC5358	PubMLST	21333	48	0048	jejuni	no	yes	no
Campylobacter jejuni OXC5456	PubMLST	21394	48	0048	jejuni	no	yes	no
Campylobacter jejuni OXC5477	PubMLST	21409	48	0048	jejuni	no	yes	no
Campylobacter jejuni OXC5483	PubMLST	23658	48	0048	jejuni	no	yes	no
Campylobacter jejuni OXC5484	PubMLST	23493	48	0048	jejuni	no	yes	no
Campylobacter jejuni OXC5636	PubMLST	23522	48	0048	jejuni	no	yes	no
Campylobacter jejuni OXC5640	PubMLST	23525	48	0048	jejuni	no	yes	no
Campylobacter jejuni OXC5694	PubMLST	21435	48	0048	jejuni	no	yes	no
Campylobacter jejuni OXC5707	PubMLST	21444	48	0048	jejuni	no	yes	no
Campylobacter jejuni OXC5712	PubMLST	21449	48	0048	jejuni	no	yes	no
Campylobacter jejuni OXC5745	PubMLST	21472	48	0048	jejuni	no	yes	no
Campylobacter jejuni OXC5838	PubMLST	23581	5173	0048	jejuni	no	yes	no
Campylobacter jejuni OXC5861	PubMLST	23669	48	0048	jejuni	no	yes	no
Campylobacter jejuni OXC5878	PubMLST	23611	48	0048	jejuni	no	yes	no
Campylobacter jejuni OXC5930	PubMLST	23651	48	0048	jejuni	no	yes	no
Campylobacter jejuni OXC6200	PubMLST	29355	48	0048	jejuni	no	yes	no
Campylobacter jejuni OXC6269	PubMLST	16066	48	0048	jejuni	no	yes	no
Campylobacter jejuni OXC6284	PubMLST	16081	48	0048	jejuni	no	yes	no
Campylobacter jejuni OXC6295	PubMLST	16092	48	0048	jejuni	no	yes	no
Campylobacter jejuni OXC6354	PubMLST	16151	48	0048	jejuni	no	yes	no
Campylobacter jejuni OXC6487	PubMLST	16279	48	0048	jejuni	no	yes	no
Campylobacter jejuni OXC6488	PubMLST	16280	48	0048	jejuni	no	yes	no
Campylobacter jejuni OXC6501	PubMLST	16293	48	0048	jejuni	no	yes	no
Campylobacter jejuni OXC6547	PubMLST	12882	48	0048	jejuni	no	yes	no
Campylobacter jejuni OXC6638	PubMLST	16387	48	0048	jejuni	no	yes	no
Campylobacter jejuni OXC6646	PubMLST	18203	48	0048	jejuni	no	yes	no
Campylobacter jejuni OXC6700	PubMLST	18257	48	0048	jejuni	no	yes	no
Campylobacter jejuni OXC6718	PubMLST	18275	48	0048	jejuni	no	yes	no
Campylobacter jejuni OXC6721	PubMLST	18278	48	0048	jejuni	no	yes	no
Campylobacter jejuni OXC6770	PubMLST	18327	48	0048	jejuni	no	yes	no
Campylobacter jejuni OXC6805	PubMLST	18362	48	0048	jejuni	no	yes	no
Campylobacter jejuni OXC6870	PubMLST	21589	48	0048	jejuni	no	yes	no
Campylobacter jejuni OXC6903	PubMLST	22098	492	0048	jejuni	no	yes	no
Campylobacter jejuni OXC6904	PubMLST	22099	48	0048	jejuni	no	yes	no
Campylobacter jejuni OXC6905	PubMLST	22100	48	0048	jejuni	no	yes	no
Campylobacter jejuni OXC6925	PubMLST	22120	48	0048	jejuni	no	yes	no
Campylobacter jejuni OXC6983	PubMLST	21156	48	0048	jejuni	no	yes	no
Campylobacter jejuni OXC6986	PubMLST	21159	48	0048	jejuni	no	yes	no
Campylobacter jejuni OXC6992	PubMLST	21164	48	0048	jejuni	no	yes	no
Campylobacter jejuni OXC6995	PubMLST	21166	48	0048	jejuni	no	yes	no
Campylobacter jejuni OXC7022	PubMLST	22209	48	0048	jejuni	no	yes	no
Campylobacter jejuni OXC7063	PubMLST	22247	48	0048	jejuni	no	yes	no
Campylobacter jejuni OXC7064	PubMLST	22248	48	0048	jejuni	no	yes	no
Campylobacter jejuni OXC7066	PubMLST	22250	48	0048	jejuni	no	yes	no
Campylobacter jejuni OXC7081	PubMLST	22265	48	0048	jejuni	no	yes	no
Campylobacter jejuni OXC7111	PubMLST	21193	48	0048	jejuni	no	yes	no
Campylobacter jejuni OXC7114	PubMLST	21196	48	0048	jejuni	no	yes	no
Campylobacter jejuni OXC7115	PubMLST	21197	48	0048	jejuni	no	yes	no
Campylobacter jejuni OXC7119	PubMLST	21201	48	0048	jejuni	no	yes	no
Campylobacter jejuni OXC7122	PubMLST	21204	48	0048	jejuni	no	yes	no
Campylobacter jejuni OXC7139	PubMLST	21220	48	0048	jejuni	no	yes	no
Campylobacter jejuni OXC7227	PubMLST	22718	5173	0048	jejuni	no	yes	no
Campylobacter jejuni OXC7279	PubMLST	22320	48	0048	jejuni	no	yes	no
Campylobacter jejuni OXC7282	PubMLST	22323	48	0048	jejuni	no	yes	no
Campylobacter jejuni OXC7288	PubMLST	22329	48	0048	jejuni	no	yes	no
Campylobacter jejuni OXC7295	PubMLST	22336	48	0048	jejuni	no	yes	no
Campylobacter jejuni OXC7341	PubMLST	22378	48	0048	jejuni	no	yes	no
Campylobacter jejuni OXC7430	PubMLST	23963	5173	0048	jejuni	no	yes	no
Campylobacter jejuni OXC7435	PubMLST	25622	48	0048	jejuni	no	yes	no
Campylobacter jejuni OXC7465	PubMLST	23994	48	0048	jejuni	no	yes	no
Campylobacter jejuni OXC7709	PubMLST	24955	48	0048	jejuni	no	yes	no
Campylobacter jejuni OXC7711	PubMLST	24956	48	0048	jejuni	no	yes	no
Campylobacter jejuni OXC7723	PubMLST	27879	48	0048	jejuni	no	yes	no
Campylobacter jejuni OXC8159	PubMLST	25646	48	0048	jejuni	no	yes	no
Campylobacter jejuni OXC8196	PubMLST	27901	48	0048	jejuni	no	yes	no
Campylobacter jejuni OXC8226	PubMLST	27928	48	0048	jejuni	no	yes	no
Campylobacter jejuni OXC8380	PubMLST	28573	492	0048	jejuni	no	yes	no
Campylobacter jejuni OXC8430	PubMLST	28621	48	0048	jejuni	no	yes	no
Campylobacter jejuni OXC8431	PubMLST	28622	48	0048	jejuni	no	yes	no
Campylobacter jejuni OXC8432	PubMLST	28623	48	0048	jejuni	no	yes	no
Campylobacter jejuni OXC8517	PubMLST	28708	48	0048	jejuni	no	yes	no
Campylobacter jejuni OXC8519	PubMLST	28710	48	0048	jejuni	no	yes	no
Campylobacter jejuni OXC8525	PubMLST	28716	48	0048	jejuni	no	yes	no
Campylobacter jejuni OXC8636	PubMLST	29073	48	0048	jejuni	no	yes	no
Campylobacter jejuni OXC8640	PubMLST	28956	2248	0048	jejuni	no	yes	no
Campylobacter jejuni OXC8776	PubMLST	30465	48	0048	jejuni	no	yes	no
Campylobacter jejuni OXC8777	PubMLST	30466	48	0048	jejuni	no	yes	no
Campylobacter jejuni OXC8971	PubMLST	30653	48	0048	jejuni	no	yes	no
Campylobacter jejuni OXC9002	PubMLST	30683	48	0048	jejuni	no	yes	no
Campylobacter jejuni OXC9010	PubMLST	30690	48	0048	jejuni	no	yes	no
Campylobacter jejuni OXC7307	PubMLST	22348	52	0052	jejuni	no	yes	no

Campylobacter jejuni OXC7743	PubMLST	24978	52	0052	jejuni	no	yes	no
Campylobacter jejuni OXC8074	PubMLST	25608	52	0052	jejuni	no	yes	no
Campylobacter jejuni OXC8081	PubMLST	25421	52	0052	jejuni	no	yes	no
Campylobacter jejuni OXC8251	PubMLST	27949	52	0052	jejuni	no	yes	no
Campylobacter jejuni OXC8319	PubMLST	28010	52	0052	jejuni	no	yes	no
Campylobacter jejuni OXC5454	PubMLST	21392	222	0206	jejuni	no	yes	no
Campylobacter jejuni OXC7203	PubMLST	22694	46	0206	jejuni	no	yes	no
Campylobacter jejuni OXC7350	PubMLST	23890	122	0206	jejuni	no	yes	no
Campylobacter jejuni OXC7576	PubMLST	24513	572	0206	jejuni	no	yes	no
Campylobacter jejuni OXC7578	PubMLST	24515	572	0206	jejuni	no	yes	no
Campylobacter jejuni OXC7730	PubMLST	25538	221	0206	jejuni	no	yes	no
Campylobacter jejuni C120544	PubMLST	28872	257	0257	jejuni	no	yes	no
Campylobacter jejuni H133640229	PubMLST	29440	257	0257	jejuni	no	yes	no
Campylobacter jejuni H142840351	PubMLST	2603	257	0257	jejuni	no	yes	no
Campylobacter jejuni H143140405	PubMLST	2610	257	0257	jejuni	no	yes	no
Campylobacter jejuni H143140406	PubMLST	2613	257	0257	jejuni	no	yes	no
Campylobacter jejuni H143140408	PubMLST	2614	257	0257	jejuni	no	yes	no
Campylobacter jejuni H143140410	PubMLST	2615	257	0257	jejuni	no	yes	no
Campylobacter jejuni OXC5235	PubMLST	24818	929	0257	jejuni	no	yes	no
Campylobacter jejuni OXC6120	PubMLST	29317	824	0257	jejuni	no	yes	no
Campylobacter jejuni OXC6170	PubMLST	29341	257	0257	jejuni	no	yes	no
Campylobacter jejuni OXC6183	PubMLST	29346	257	0257	jejuni	no	yes	no
Campylobacter jejuni OXC6183R	PubMLST	29891	257	0257	jejuni	no	yes	no
Campylobacter jejuni OXC7491	PubMLST	24019	824	0257	jejuni	no	yes	no
Campylobacter jejuni OXC7492	PubMLST	24020	824	0257	jejuni	no	yes	no
Campylobacter jejuni OXC7607	PubMLST	24537	824	0257	jejuni	no	yes	no
Campylobacter jejuni OXC7712	PubMLST	24957	824	0257	jejuni	no	yes	no
Campylobacter jejuni OXC7829	PubMLST	24626	267	0283	jejuni	no	yes	no
Campylobacter jejuni ARI_915_R	PubMLST	24318	5	0353	jejuni	no	yes	no
Campylobacter jejuni BID17C	PubMLST	25070	353	0353	jejuni	no	yes	no
Campylobacter jejuni BID19R	PubMLST	24236	2851	0353	jejuni	no	yes	no
Campylobacter jejuni BID1NQ	PubMLST	25079	353	0353	jejuni	no	yes	no
Campylobacter jejuni BID1Q5	PubMLST	25080	353	0353	jejuni	no	yes	no
Campylobacter jejuni BID1QG	PubMLST	25081	581	0353	jejuni	no	yes	no
Campylobacter jejuni H134120022	PubMLST	2661	5	0353	jejuni	no	yes	no
Campylobacter jejuni HM115771	PubMLST	24199	5	0353	jejuni	no	yes	no
Campylobacter jejuni OXC4602	PubMLST	24069	5	0353	jejuni	no	yes	no
Campylobacter jejuni OXC4643	PubMLST	24090	5	0353	jejuni	no	yes	no
Campylobacter jejuni OXC4775	PubMLST	18465	5	0353	jejuni	no	yes	no
Campylobacter jejuni OXC4781	PubMLST	22132	5	0353	jejuni	no	yes	no
Campylobacter jejuni OXC4859	PubMLST	22171	5	0353	jejuni	no	yes	no
Campylobacter jejuni OXC4879	PubMLST	22184	5	0353	jejuni	no	yes	no
Campylobacter jejuni OXC4951	PubMLST	25370	5	0353	jejuni	no	yes	no
Campylobacter jejuni OXC4972	PubMLST	24663	5	0353	jejuni	no	yes	no
Campylobacter jejuni OXC4975	PubMLST	24665	5	0353	jejuni	no	yes	no
Campylobacter jejuni OXC4989	PubMLST	24672	5	0353	jejuni	no	yes	no
Campylobacter jejuni OXC5056	PubMLST	24715	5	0353	jejuni	no	yes	no
Campylobacter jejuni OXC5089	PubMLST	24730	5	0353	jejuni	no	yes	no
Campylobacter jejuni OXC5120	PubMLST	24743	5	0353	jejuni	no	yes	no
Campylobacter jejuni OXC5167	PubMLST	24768	5	0353	jejuni	no	yes	no
Campylobacter jejuni OXC5176	PubMLST	24773	5	0353	jejuni	no	yes	no
Campylobacter jejuni OXC5198	PubMLST	24790	5	0353	jejuni	no	yes	no
Campylobacter jejuni OXC5214	PubMLST	24802	5	0353	jejuni	no	yes	no
Campylobacter jejuni OXC5227	PubMLST	24813	5	0353	jejuni	no	yes	no
Campylobacter jejuni OXC5355	PubMLST	21332	1232	0353	jejuni	no	yes	no
Campylobacter jejuni OXC5381	PubMLST	21346	7176	0353	jejuni	no	yes	no
Campylobacter jejuni OXC5427	PubMLST	21376	5	0353	jejuni	no	yes	no
Campylobacter jejuni OXC5428	PubMLST	21377	5	0353	jejuni	no	yes	no
Campylobacter jejuni OXC5472	PubMLST	21404	5	0353	jejuni	no	yes	no
Campylobacter jejuni OXC5480	PubMLST	21411	2122	0353	jejuni	no	yes	no
Campylobacter jejuni OXC5499	PubMLST	23500	5	0353	jejuni	no	yes	no
Campylobacter jejuni OXC5633	PubMLST	23519	2076	0353	jejuni	no	yes	no
Campylobacter jejuni OXC5634	PubMLST	23520	5	0353	jejuni	no	yes	no
Campylobacter jejuni OXC5638	PubMLST	23523	581	0353	jejuni	no	yes	no
Campylobacter jejuni OXC5679	PubMLST	21425	5	0353	jejuni	no	yes	no
Campylobacter jejuni OXC5680	PubMLST	21426	5	0353	jejuni	no	yes	no
Campylobacter jejuni OXC5690	PubMLST	21433	5	0353	jejuni	no	yes	no
Campylobacter jejuni OXC5701	PubMLST	21440	353	0353	jejuni	no	yes	no
Campylobacter jejuni OXC5721	PubMLST	21454	5	0353	jejuni	no	yes	no
Campylobacter jejuni OXC5726	PubMLST	21458	5	0353	jejuni	no	yes	no
Campylobacter jejuni OXC5780	PubMLST	21500	5	0353	jejuni	no	yes	no
Campylobacter jejuni OXC5813	PubMLST	23561	5	0353	jejuni	no	yes	no
Campylobacter jejuni OXC5839	PubMLST	23582	3510	0353	jejuni	no	yes	no
Campylobacter jejuni OXC5886	PubMLST	23617	5	0353	jejuni	no	yes	no
Campylobacter jejuni OXC6100	PubMLST	29305	5	0353	jejuni	no	yes	no
Campylobacter jejuni OXC6260	PubMLST	16057	5	0353	jejuni	no	yes	no
Campylobacter jejuni OXC6319	PubMLST	16116	356	0353	jejuni	no	yes	no
Campylobacter jejuni OXC6438	PubMLST	16233	5	0353	jejuni	no	yes	no
Campylobacter jejuni OXC6473	PubMLST	16265	5	0353	jejuni	no	yes	no
Campylobacter jejuni OXC6478	PubMLST	16270	5	0353	jejuni	no	yes	no
Campylobacter jejuni OXC6494	PubMLST	16286	353	0353	jejuni	no	yes	no
Campylobacter jejuni OXC6497	PubMLST	16289	5	0353	jejuni	no	yes	no
Campylobacter jejuni OXC6507	PubMLST	16299	5	0353	jejuni	no	yes	no
Campylobacter jejuni OXC6520	PubMLST	16312	5	0353	jejuni	no	yes	no
Campylobacter jejuni OXC6566	PubMLST	12901	5	0353	jejuni	no	yes	no
Campylobacter jejuni OXC6566_V2	PubMLST	24173	5	0353	jejuni	no	yes	no
Campylobacter jejuni OXC6566R	PubMLST	21177	5	0353	jejuni	no	yes	no
Campylobacter jejuni OXC6566R_V2	PubMLST	24183	5	0353	jejuni	no	yes	no
Campylobacter jejuni OXC6575	PubMLST	12910	5	0353	jejuni	no	yes	no
Campylobacter jejuni OXC6575_V2	PubMLST	24174	5	0353	jejuni	no	yes	no
Campylobacter jejuni OXC6575R	PubMLST	21178	5	0353	jejuni	no	yes	no
Campylobacter jejuni OXC6575R_V2	PubMLST	24184	5	0353	jejuni	no	yes	no

Campylobacter jejuni OXC6584	PubMLST	12921	5758	0353	jejuni	no	yes	no
Campylobacter jejuni OXC6585	PubMLST	12922	5	0353	jejuni	no	yes	no
Campylobacter jejuni OXC6588	PubMLST	12925	5	0353	jejuni	no	yes	no
Campylobacter jejuni OXC6591	PubMLST	16342	5	0353	jejuni	no	yes	no
Campylobacter jejuni OXC6607	PubMLST	16357	353	0353	jejuni	no	yes	no
Campylobacter jejuni OXC6707	PubMLST	18264	5	0353	jejuni	no	yes	no
Campylobacter jejuni OXC6757	PubMLST	18314	5	0353	jejuni	no	yes	no
Campylobacter jejuni OXC6814	PubMLST	18371	356	0353	jejuni	no	yes	no
Campylobacter jejuni OXC6818	PubMLST	18375	356	0353	jejuni	no	yes	no
Campylobacter jejuni OXC6822	PubMLST	18379	356	0353	jejuni	no	yes	no
Campylobacter jejuni OXC6855	PubMLST	21574	5	0353	jejuni	no	yes	no
Campylobacter jejuni OXC6913	PubMLST	22108	5	0353	jejuni	no	yes	no
Campylobacter jejuni OXC6928	PubMLST	22123	5	0353	jejuni	no	yes	no
Campylobacter jejuni OXC6968	PubMLST	21142	5	0353	jejuni	no	yes	no
Campylobacter jejuni OXC6977	PubMLST	21150	5	0353	jejuni	no	yes	no
Campylobacter jejuni OXC7024	PubMLST	22211	5	0353	jejuni	no	yes	no
Campylobacter jejuni OXC7047	PubMLST	22232	5	0353	jejuni	no	yes	no
Campylobacter jejuni OXC7069	PubMLST	22253	5	0353	jejuni	no	yes	no
Campylobacter jejuni OXC7101	PubMLST	21183	356	0353	jejuni	no	yes	no
Campylobacter jejuni OXC7184	PubMLST	22675	5	0353	jejuni	no	yes	no
Campylobacter jejuni OXC7209	PubMLST	22700	5	0353	jejuni	no	yes	no
Campylobacter jejuni OXC7222	PubMLST	22713	5	0353	jejuni	no	yes	no
Campylobacter jejuni OXC7263	PubMLST	22304	5	0353	jejuni	no	yes	no
Campylobacter jejuni OXC7300	PubMLST	22341	595	0353	jejuni	no	yes	no
Campylobacter jejuni OXC7332	PubMLST	22370	5	0353	jejuni	no	yes	no
Campylobacter jejuni OXC7359	PubMLST	23898	5	0353	jejuni	no	yes	no
Campylobacter jejuni OXC7360	PubMLST	23899	5	0353	jejuni	no	yes	no
Campylobacter jejuni OXC7448	PubMLST	23978	5	0353	jejuni	no	yes	no
Campylobacter jejuni OXC7485	PubMLST	24013	5	0353	jejuni	no	yes	no
Campylobacter jejuni OXC7556	PubMLST	24493	5	0353	jejuni	no	yes	no
Campylobacter jejuni OXC7569	PubMLST	24506	6759	0353	jejuni	no	yes	no
Campylobacter jejuni OXC7597	PubMLST	24529	581	0353	jejuni	no	yes	no
Campylobacter jejuni OXC7619	PubMLST	24548	5	0353	jejuni	no	yes	no
Campylobacter jejuni OXC7643	PubMLST	24900	5	0353	jejuni	no	yes	no
Campylobacter jejuni OXC7654	PubMLST	24909	581	0353	jejuni	no	yes	no
Campylobacter jejuni OXC7698	PubMLST	24946	5	0353	jejuni	no	yes	no
Campylobacter jejuni OXC7759	PubMLST	24571	5	0353	jejuni	no	yes	no
Campylobacter jejuni OXC7768	PubMLST	24577	5	0353	jejuni	no	yes	no
Campylobacter jejuni OXC7783	PubMLST	25654	5	0353	jejuni	no	yes	no
Campylobacter jejuni OXC7790	PubMLST	24593	5	0353	jejuni	no	yes	no
Campylobacter jejuni OXC7848	PubMLST	24645	5	0353	jejuni	no	yes	no
Campylobacter jejuni OXC7874	PubMLST	25009	6889	0353	jejuni	no	yes	no
Campylobacter jejuni OXC7891	PubMLST	25022	5	0353	jejuni	no	yes	no
Campylobacter jejuni OXC7902	PubMLST	25033	5	0353	jejuni	no	yes	no
Campylobacter jejuni OXC8158	PubMLST	25641	5	0353	jejuni	no	yes	no
Campylobacter jejuni OXC8185	PubMLST	27890	6759	0353	jejuni	no	yes	no
Campylobacter jejuni OXC8266	PubMLST	29065	356	0353	jejuni	no	yes	no
Campylobacter jejuni OXC8277	PubMLST	27970	356	0353	jejuni	no	yes	no
Campylobacter jejuni OXC8299	PubMLST	27990	5	0353	jejuni	no	yes	no
Campylobacter jejuni OXC8308	PubMLST	27999	5	0353	jejuni	no	yes	no
Campylobacter jejuni OXC8311	PubMLST	28002	5	0353	jejuni	no	yes	no
Campylobacter jejuni OXC8328	PubMLST	28019	5	0353	jejuni	no	yes	no
Campylobacter jejuni OXC8347	PubMLST	28565	5	0353	jejuni	no	yes	no
Campylobacter jejuni OXC8374	PubMLST	27874	5	0353	jejuni	no	yes	no
Campylobacter jejuni OXC8463	PubMLST	28656	5	0353	jejuni	no	yes	no
Campylobacter jejuni OXC8497	PubMLST	28688	581	0353	jejuni	no	yes	no
Campylobacter jejuni OXC8507	PubMLST	28698	5	0353	jejuni	no	yes	no
Campylobacter jejuni OXC8518	PubMLST	28709	5	0353	jejuni	no	yes	no
Campylobacter jejuni OXC8560	PubMLST	29070	5	0353	jejuni	no	yes	no
Campylobacter jejuni OXC8576	PubMLST	28913	581	0353	jejuni	no	yes	no
Campylobacter jejuni OXC8651	PubMLST	28965	5	0353	jejuni	no	yes	no
Campylobacter jejuni OXC8722	PubMLST	29048	2122	0353	jejuni	no	yes	no
Campylobacter jejuni OXC8742	PubMLST	29078	5	0353	jejuni	no	yes	no
Campylobacter jejuni OXC8861	PubMLST	30547	6759	0353	jejuni	no	yes	no
Campylobacter jejuni OXC8891	PubMLST	30575	6759	0353	jejuni	no	yes	no
Campylobacter jejuni OXC8892	PubMLST	30576	6759	0353	jejuni	no	yes	no
Campylobacter jejuni OXC8893	PubMLST	30577	581	0353	jejuni	no	yes	no
Campylobacter jejuni OXC8940	PubMLST	30623	5	0353	jejuni	no	yes	no
Campylobacter jejuni OXC4648	PubMLST	25087	2288	0354	jejuni	no	yes	no
Campylobacter jejuni OXC4727	PubMLST	18434	354	0354	jejuni	no	yes	no
Campylobacter jejuni OXC5128	PubMLST	24750	354	0354	jejuni	no	yes	no
Campylobacter jejuni OXC5206	PubMLST	24796	354	0354	jejuni	no	yes	no
Campylobacter jejuni OXC5207	PubMLST	24797	354	0354	jejuni	no	yes	no
Campylobacter jejuni OXC5243	PubMLST	24825	354	0354	jejuni	no	yes	no
Campylobacter jejuni OXC5385	PubMLST	21348	354	0354	jejuni	no	yes	no
Campylobacter jejuni OXC5400	PubMLST	21359	354	0354	jejuni	no	yes	no
Campylobacter jejuni OXC5466	PubMLST	21402	354	0354	jejuni	no	yes	no
Campylobacter jejuni OXC5475	PubMLST	21407	354	0354	jejuni	no	yes	no
Campylobacter jejuni OXC5695	PubMLST	21436	354	0354	jejuni	no	yes	no
Campylobacter jejuni OXC5741	PubMLST	21468	354	0354	jejuni	no	yes	no
Campylobacter jejuni OXC6158	PubMLST	29337	1073	0354	jejuni	no	yes	no
Campylobacter jejuni OXC6608	PubMLST	16358	1073	0354	jejuni	no	yes	no
Campylobacter jejuni OXC6801	PubMLST	18358	354	0354	jejuni	no	yes	no
Campylobacter jejuni OXC7204	PubMLST	22695	354	0354	jejuni	no	yes	no
Campylobacter jejuni OXC7408	PubMLST	23943	354	0354	jejuni	no	yes	no
Campylobacter jejuni OXC7409	PubMLST	23944	354	0354	jejuni	no	yes	no
Campylobacter jejuni OXC7594	PubMLST	24526	354	0354	jejuni	no	yes	no
Campylobacter jejuni OXC8160	PubMLST	28560	354	0354	jejuni	no	yes	no
Campylobacter jejuni RM1221	Genbank/EMBL/DDBJ	NC_003912	354	0354	jejuni	no	yes	no
Campylobacter jejuni S3	Genbank/EMBL/DDBJ	CP001960	354	0354	jejuni	no	yes	no
Campylobacter jejuni UKZN015_R	PubMLST	24346	6464	0354	jejuni	no	yes	no
Campylobacter jejuni 260.94	Genbank/EMBL/DDBJ	AANK01	362	0362	jejuni	no	yes	no

Campylobacter jejuni BID19O	PubMLST	25075	6841	0362	jejuni	no	yes	no
Campylobacter jejuni ICDCCJ07001	Genbank/EMBL/DDBJ	NC_014801	2993	0362	jejuni	no	yes	no
Campylobacter jejuni OXC6484	PubMLST	16276	403	0403	jejuni	no	yes	no
Campylobacter jejuni OXC6484_V2	PubMLST	24171	403	0403	jejuni	no	yes	no
Campylobacter jejuni OXC6484R	PubMLST	21175	403	0403	jejuni	no	yes	no
Campylobacter jejuni OXC6484R_V2	PubMLST	24181	403	0403	jejuni	no	yes	no
Campylobacter jejuni OXC6485	PubMLST	16277	403	0403	jejuni	no	yes	no
Campylobacter jejuni OXC6485_V2	PubMLST	24172	403	0403	jejuni	no	yes	no
Campylobacter jejuni OXC6485R	PubMLST	21176	403	0403	jejuni	no	yes	no
Campylobacter jejuni OXC6485R_V2	PubMLST	24182	403	0403	jejuni	no	yes	no
Campylobacter jejuni OXC8681	PubMLST	29013	403	0403	jejuni	no	yes	no
Campylobacter jejuni OXC6410	PubMLST	16205	5728	0433	jejuni	no	yes	no
Campylobacter jejuni OXC4661	PubMLST	24102	51	0443	jejuni	no	yes	no
Campylobacter jejuni OXC4667	PubMLST	24106	51	0443	jejuni	no	yes	no
Campylobacter jejuni OXC4931	PubMLST	24468	3738	0460	jejuni	no	yes	no
Campylobacter jejuni OXC5018	PubMLST	24689	2844	0460	jejuni	no	yes	no
Campylobacter jejuni OXC5660	PubMLST	21413	2844	0460	jejuni	no	yes	no
Campylobacter jejuni OXC5672	PubMLST	21419	2844	0460	jejuni	no	yes	no
Campylobacter jejuni OXC5753	PubMLST	21477	5144	0460	jejuni	no	yes	no
Campylobacter jejuni OXC5845	PubMLST	23666	4403	0460	jejuni	no	yes	no
Campylobacter jejuni OXC6427	PubMLST	16222	2844	0460	jejuni	no	yes	no
Campylobacter jejuni OXC6653	PubMLST	18210	2844	0460	jejuni	no	yes	no
Campylobacter jejuni OXC6662	PubMLST	18219	5808	0460	jejuni	no	yes	no
Campylobacter jejuni OXC6663	PubMLST	18220	2844	0460	jejuni	no	yes	no
Campylobacter jejuni OXC6670	PubMLST	18227	2844	0460	jejuni	no	yes	no
Campylobacter jejuni OXC7008	PubMLST	22195	606	0460	jejuni	no	yes	no
Campylobacter jejuni OXC8229	PubMLST	27931	2844	0460	jejuni	no	yes	no
Campylobacter jejuni OXC8470	PubMLST	28663	2844	0460	jejuni	no	yes	no
Campylobacter jejuni ARI_1021_R	PubMLST	24320	464	0464	jejuni	no	yes	no
Campylobacter jejuni CJ6657_R	PubMLST	24305	464	0464	jejuni	no	yes	no
Campylobacter jejuni CJ6849_R	PubMLST	24338	464	0464	jejuni	no	yes	no
Campylobacter jejuni H120940503	PubMLST	2629	5812	0464	jejuni	no	yes	no
Campylobacter jejuni H121820081	PubMLST	2635	5136	0464	jejuni	no	yes	no
Campylobacter jejuni H121940252	PubMLST	2638	5136	0464	jejuni	no	yes	no
Campylobacter jejuni H141140403	PubMLST	29456	5136	0464	jejuni	no	yes	no
Campylobacter jejuni H143940392	PubMLST	31010	5136	0464	jejuni	no	yes	no
Campylobacter jejuni OXC4599	PubMLST	24067	464	0464	jejuni	no	yes	no
Campylobacter jejuni OXC4623	PubMLST	24076	464	0464	jejuni	no	yes	no
Campylobacter jejuni OXC4763	PubMLST	18457	464	0464	jejuni	no	yes	no
Campylobacter jejuni OXC4876	PubMLST	22181	464	0464	jejuni	no	yes	no
Campylobacter jejuni OXC4924	PubMLST	25091	464	0464	jejuni	no	yes	no
Campylobacter jejuni OXC5066	PubMLST	24720	464	0464	jejuni	no	yes	no
Campylobacter jejuni OXC5121	PubMLST	24744	464	0464	jejuni	no	yes	no
Campylobacter jejuni OXC5342	PubMLST	21324	464	0464	jejuni	no	yes	no
Campylobacter jejuni OXC5371	PubMLST	21338	464	0464	jejuni	no	yes	no
Campylobacter jejuni OXC5375	PubMLST	21341	464	0464	jejuni	no	yes	no
Campylobacter jejuni OXC5417	PubMLST	21370	464	0464	jejuni	no	yes	no
Campylobacter jejuni OXC5459	PubMLST	21396	5136	0464	jejuni	no	yes	no
Campylobacter jejuni OXC5479	PubMLST	21410	5136	0464	jejuni	no	yes	no
Campylobacter jejuni OXC5645	PubMLST	23529	464	0464	jejuni	no	yes	no
Campylobacter jejuni OXC5715	PubMLST	21451	5136	0464	jejuni	no	yes	no
Campylobacter jejuni OXC5727	PubMLST	21459	464	0464	jejuni	no	yes	no
Campylobacter jejuni OXC5765	PubMLST	21486	464	0464	jejuni	no	yes	no
Campylobacter jejuni OXC5777	PubMLST	21497	464	0464	jejuni	no	yes	no
Campylobacter jejuni OXC5807	PubMLST	23555	5136	0464	jejuni	no	yes	no
Campylobacter jejuni OXC5834	PubMLST	23578	5136	0464	jejuni	no	yes	no
Campylobacter jejuni OXC5851	PubMLST	23592	464	0464	jejuni	no	yes	no
Campylobacter jejuni OXC5910	PubMLST	23636	464	0464	jejuni	no	yes	no
Campylobacter jejuni OXC5921	PubMLST	23644	5136	0464	jejuni	no	yes	no
Campylobacter jejuni OXC5940	PubMLST	23682	464	0464	jejuni	no	yes	no
Campylobacter jejuni OXC6288	PubMLST	16085	464	0464	jejuni	no	yes	no
Campylobacter jejuni OXC6352	PubMLST	16149	464	0464	jejuni	no	yes	no
Campylobacter jejuni OXC6356	PubMLST	16153	464	0464	jejuni	no	yes	no
Campylobacter jejuni OXC6423	PubMLST	16218	464	0464	jejuni	no	yes	no
Campylobacter jejuni OXC6475	PubMLST	16267	5136	0464	jejuni	no	yes	no
Campylobacter jejuni OXC6541	PubMLST	16333	5136	0464	jejuni	no	yes	no
Campylobacter jejuni OXC6550	PubMLST	12885	464	0464	jejuni	no	yes	no
Campylobacter jejuni OXC6554	PubMLST	12889	464	0464	jejuni	no	yes	no
Campylobacter jejuni OXC6555	PubMLST	12890	464	0464	jejuni	no	yes	no
Campylobacter jejuni OXC6572	PubMLST	12907	464	0464	jejuni	no	yes	no
Campylobacter jejuni OXC6581	PubMLST	12918	464	0464	jejuni	no	yes	no
Campylobacter jejuni OXC6621	PubMLST	16370	464	0464	jejuni	no	yes	no
Campylobacter jejuni OXC6635	PubMLST	16384	5136	0464	jejuni	no	yes	no
Campylobacter jejuni OXC6640	PubMLST	16389	464	0464	jejuni	no	yes	no
Campylobacter jejuni OXC6657	PubMLST	18214	464	0464	jejuni	no	yes	no
Campylobacter jejuni OXC6692	PubMLST	18249	464	0464	jejuni	no	yes	no
Campylobacter jejuni OXC6694	PubMLST	18251	5136	0464	jejuni	no	yes	no
Campylobacter jejuni OXC6701	PubMLST	18258	464	0464	jejuni	no	yes	no
Campylobacter jejuni OXC6722	PubMLST	18279	464	0464	jejuni	no	yes	no
Campylobacter jejuni OXC6732	PubMLST	18289	5812	0464	jejuni	no	yes	no
Campylobacter jejuni OXC6748	PubMLST	18305	5136	0464	jejuni	no	yes	no
Campylobacter jejuni OXC6756	PubMLST	18313	464	0464	jejuni	no	yes	no
Campylobacter jejuni OXC6758	PubMLST	18315	464	0464	jejuni	no	yes	no
Campylobacter jejuni OXC6767	PubMLST	18324	464	0464	jejuni	no	yes	no
Campylobacter jejuni OXC6774	PubMLST	18331	5136	0464	jejuni	no	yes	no
Campylobacter jejuni OXC6778	PubMLST	18335	464	0464	jejuni	no	yes	no
Campylobacter jejuni OXC6780	PubMLST	18337	464	0464	jejuni	no	yes	no
Campylobacter jejuni OXC6813	PubMLST	18370	464	0464	jejuni	no	yes	no
Campylobacter jejuni OXC6828	PubMLST	18385	464	0464	jejuni	no	yes	no
Campylobacter jejuni OXC6841	PubMLST	21560	5136	0464	jejuni	no	yes	no
Campylobacter jejuni OXC6848	PubMLST	21567	5136	0464	jejuni	no	yes	no
Campylobacter jejuni OXC6849	PubMLST	21568	464	0464	jejuni	no	yes	no

Campylobacter jejuni OXC6853	PubMLST	21572	5136	0464	jejuni	no	yes	no
Campylobacter jejuni OXC6914	PubMLST	22109	5136	0464	jejuni	no	yes	no
Campylobacter jejuni OXC6923	PubMLST	22118	5136	0464	jejuni	no	yes	no
Campylobacter jejuni OXC6926	PubMLST	22121	5136	0464	jejuni	no	yes	no
Campylobacter jejuni OXC6935	PubMLST	21109	5136	0464	jejuni	no	yes	no
Campylobacter jejuni OXC6940	PubMLST	21114	5136	0464	jejuni	no	yes	no
Campylobacter jejuni OXC6943	PubMLST	21117	464	0464	jejuni	no	yes	no
Campylobacter jejuni OXC6955	PubMLST	21129	5136	0464	jejuni	no	yes	no
Campylobacter jejuni OXC6958	PubMLST	21132	5136	0464	jejuni	no	yes	no
Campylobacter jejuni OXC6969	PubMLST	21143	5136	0464	jejuni	no	yes	no
Campylobacter jejuni OXC6971	PubMLST	21145	5136	0464	jejuni	no	yes	no
Campylobacter jejuni OXC6980	PubMLST	21153	5136	0464	jejuni	no	yes	no
Campylobacter jejuni OXC6993	PubMLST	22188	5136	0464	jejuni	no	yes	no
Campylobacter jejuni OXC7011	PubMLST	22198	5136	0464	jejuni	no	yes	no
Campylobacter jejuni OXC7057	PubMLST	22242	464	0464	jejuni	no	yes	no
Campylobacter jejuni OXC7062	PubMLST	22246	5136	0464	jejuni	no	yes	no
Campylobacter jejuni OXC7116	PubMLST	21198	5136	0464	jejuni	no	yes	no
Campylobacter jejuni OXC7121	PubMLST	21203	5136	0464	jejuni	no	yes	no
Campylobacter jejuni OXC7127	PubMLST	21208	5136	0464	jejuni	no	yes	no
Campylobacter jejuni OXC7135	PubMLST	21216	5136	0464	jejuni	no	yes	no
Campylobacter jejuni OXC7166	PubMLST	21247	5136	0464	jejuni	no	yes	no
Campylobacter jejuni OXC7171	PubMLST	22662	5136	0464	jejuni	no	yes	no
Campylobacter jejuni OXC7172	PubMLST	22663	5136	0464	jejuni	no	yes	no
Campylobacter jejuni OXC7175	PubMLST	22666	5136	0464	jejuni	no	yes	no
Campylobacter jejuni OXC7183	PubMLST	22674	464	0464	jejuni	no	yes	no
Campylobacter jejuni OXC7202	PubMLST	22693	5136	0464	jejuni	no	yes	no
Campylobacter jejuni OXC7221	PubMLST	22712	5136	0464	jejuni	no	yes	no
Campylobacter jejuni OXC7224	PubMLST	22715	5136	0464	jejuni	no	yes	no
Campylobacter jejuni OXC7237	PubMLST	22727	5136	0464	jejuni	no	yes	no
Campylobacter jejuni OXC7286	PubMLST	22327	5136	0464	jejuni	no	yes	no
Campylobacter jejuni OXC7290	PubMLST	22331	5136	0464	jejuni	no	yes	no
Campylobacter jejuni OXC7297	PubMLST	22338	5136	0464	jejuni	no	yes	no
Campylobacter jejuni OXC7298	PubMLST	22339	5136	0464	jejuni	no	yes	no
Campylobacter jejuni OXC7304	PubMLST	22345	5136	0464	jejuni	no	yes	no
Campylobacter jejuni OXC7313	PubMLST	22354	5136	0464	jejuni	no	yes	no
Campylobacter jejuni OXC7314	PubMLST	22355	5136	0464	jejuni	no	yes	no
Campylobacter jejuni OXC7318	PubMLST	25552	5136	0464	jejuni	no	yes	no
Campylobacter jejuni OXC7379	PubMLST	23917	5136	0464	jejuni	no	yes	no
Campylobacter jejuni OXC7408_R	PubMLST	25451	5136	0464	jejuni	no	yes	no
Campylobacter jejuni OXC7421	PubMLST	23955	5136	0464	jejuni	no	yes	no
Campylobacter jejuni OXC7438	PubMLST	23969	5136	0464	jejuni	no	yes	no
Campylobacter jejuni OXC7467	PubMLST	23996	5136	0464	jejuni	no	yes	no
Campylobacter jejuni OXC7500	PubMLST	24027	5136	0464	jejuni	no	yes	no
Campylobacter jejuni OXC7502	PubMLST	24028	5136	0464	jejuni	no	yes	no
Campylobacter jejuni OXC7521	PubMLST	24125	5136	0464	jejuni	no	yes	no
Campylobacter jejuni OXC7534	PubMLST	24477	5136	0464	jejuni	no	yes	no
Campylobacter jejuni OXC7541	PubMLST	24482	5136	0464	jejuni	no	yes	no
Campylobacter jejuni OXC7544	PubMLST	24485	5136	0464	jejuni	no	yes	no
Campylobacter jejuni OXC7549	PubMLST	24488	5136	0464	jejuni	no	yes	no
Campylobacter jejuni OXC7557	PubMLST	24494	5136	0464	jejuni	no	yes	no
Campylobacter jejuni OXC7564	PubMLST	24501	5136	0464	jejuni	no	yes	no
Campylobacter jejuni OXC7568	PubMLST	24505	5136	0464	jejuni	no	yes	no
Campylobacter jejuni OXC7570	PubMLST	24507	5136	0464	jejuni	no	yes	no
Campylobacter jejuni OXC7589	PubMLST	24523	5136	0464	jejuni	no	yes	no
Campylobacter jejuni OXC7600	PubMLST	24532	5136	0464	jejuni	no	yes	no
Campylobacter jejuni OXC7617	PubMLST	24546	5136	0464	jejuni	no	yes	no
Campylobacter jejuni OXC7633	PubMLST	24562	5136	0464	jejuni	no	yes	no
Campylobacter jejuni OXC7635	PubMLST	24564	5136	0464	jejuni	no	yes	no
Campylobacter jejuni OXC7642	PubMLST	24899	5136	0464	jejuni	no	yes	no
Campylobacter jejuni OXC7658	PubMLST	24913	464	0464	jejuni	no	yes	no
Campylobacter jejuni OXC7681	PubMLST	27878	5136	0464	jejuni	no	yes	no
Campylobacter jejuni OXC7734	PubMLST	24972	5136	0464	jejuni	no	yes	no
Campylobacter jejuni OXC7736	PubMLST	24974	5136	0464	jejuni	no	yes	no
Campylobacter jejuni OXC7770	PubMLST	24579	5136	0464	jejuni	no	yes	no
Campylobacter jejuni OXC7808	PubMLST	24606	5136	0464	jejuni	no	yes	no
Campylobacter jejuni OXC7811	PubMLST	24609	5136	0464	jejuni	no	yes	no
Campylobacter jejuni OXC7816	PubMLST	24614	5136	0464	jejuni	no	yes	no
Campylobacter jejuni OXC7847	PubMLST	24644	5136	0464	jejuni	no	yes	no
Campylobacter jejuni OXC7851	PubMLST	24648	5136	0464	jejuni	no	yes	no
Campylobacter jejuni OXC7868	PubMLST	25003	464	0464	jejuni	no	yes	no
Campylobacter jejuni OXC7888	PubMLST	25019	464	0464	jejuni	no	yes	no
Campylobacter jejuni OXC7912	PubMLST	25042	464	0464	jejuni	no	yes	no
Campylobacter jejuni OXC7936	PubMLST	25567	5136	0464	jejuni	no	yes	no
Campylobacter jejuni OXC7945	PubMLST	25068	464	0464	jejuni	no	yes	no
Campylobacter jejuni OXC7951	PubMLST	25571	464	0464	jejuni	no	yes	no
Campylobacter jejuni OXC7959	PubMLST	25377	464	0464	jejuni	no	yes	no
Campylobacter jejuni OXC7989	PubMLST	25481	5136	0464	jejuni	no	yes	no
Campylobacter jejuni OXC7992	PubMLST	25483	464	0464	jejuni	no	yes	no
Campylobacter jejuni OXC7994	PubMLST	25484	5136	0464	jejuni	no	yes	no
Campylobacter jejuni OXC8008	PubMLST	25579	5136	0464	jejuni	no	yes	no
Campylobacter jejuni OXC8014	PubMLST	25584	5136	0464	jejuni	no	yes	no
Campylobacter jejuni OXC8028	PubMLST	25590	5136	0464	jejuni	no	yes	no
Campylobacter jejuni OXC8028R	PubMLST	25647	5136	0464	jejuni	no	yes	no
Campylobacter jejuni OXC8037	PubMLST	25412	464	0464	jejuni	no	yes	no
Campylobacter jejuni OXC8039	PubMLST	25592	5136	0464	jejuni	no	yes	no
Campylobacter jejuni OXC8044	PubMLST	25413	5136	0464	jejuni	no	yes	no
Campylobacter jejuni OXC8048	PubMLST	25509	464	0464	jejuni	no	yes	no
Campylobacter jejuni OXC8052	PubMLST	25510	464	0464	jejuni	no	yes	no
Campylobacter jejuni OXC8053	PubMLST	25598	464	0464	jejuni	no	yes	no
Campylobacter jejuni OXC8057	PubMLST	25601	5136	0464	jejuni	no	yes	no
Campylobacter jejuni OXC8068	PubMLST	25417	464	0464	jejuni	no	yes	no
Campylobacter jejuni OXC8079	PubMLST	25420	464	0464	jejuni	no	yes	no

Campylobacter jejuni OXC8093	PubMLST	25611	5136	0464	jejuni	no	yes	no
Campylobacter jejuni OXC8119	PubMLST	25535	5136	0464	jejuni	no	yes	no
Campylobacter jejuni OXC8123	PubMLST	25556	5136	0464	jejuni	no	yes	no
Campylobacter jejuni OXC8149	PubMLST	25637	5136	0464	jejuni	no	yes	no
Campylobacter jejuni OXC8167	PubMLST	25545	5136	0464	jejuni	no	yes	no
Campylobacter jejuni OXC8168	PubMLST	25648	5136	0464	jejuni	no	yes	no
Campylobacter jejuni OXC8201	PubMLST	27906	5136	0464	jejuni	no	yes	no
Campylobacter jejuni OXC8218	PubMLST	27921	5136	0464	jejuni	no	yes	no
Campylobacter jejuni OXC8221	PubMLST	27923	5136	0464	jejuni	no	yes	no
Campylobacter jejuni OXC8235	PubMLST	27937	5812	0464	jejuni	no	yes	no
Campylobacter jejuni OXC8288	PubMLST	27979	5136	0464	jejuni	no	yes	no
Campylobacter jejuni OXC8322	PubMLST	28013	5136	0464	jejuni	no	yes	no
Campylobacter jejuni OXC8341	PubMLST	28032	5136	0464	jejuni	no	yes	no
Campylobacter jejuni OXC8357	PubMLST	28566	5136	0464	jejuni	no	yes	no
Campylobacter jejuni OXC8363	PubMLST	28052	5136	0464	jejuni	no	yes	no
Campylobacter jejuni OXC8389	PubMLST	28582	5136	0464	jejuni	no	yes	no
Campylobacter jejuni OXC8397	PubMLST	28898	5136	0464	jejuni	no	yes	no
Campylobacter jejuni OXC8397R	PubMLST	29066	5136	0464	jejuni	no	yes	no
Campylobacter jejuni OXC8454	PubMLST	28645	5136	0464	jejuni	no	yes	no
Campylobacter jejuni OXC8461	PubMLST	28652	5136	0464	jejuni	no	yes	no
Campylobacter jejuni OXC8464	PubMLST	28657	5136	0464	jejuni	no	yes	no
Campylobacter jejuni OXC8469	PubMLST	28662	464	0464	jejuni	no	yes	no
Campylobacter jejuni OXC8475	PubMLST	28668	5136	0464	jejuni	no	yes	no
Campylobacter jejuni OXC8479	PubMLST	28672	5136	0464	jejuni	no	yes	no
Campylobacter jejuni OXC8496	PubMLST	28687	5136	0464	jejuni	no	yes	no
Campylobacter jejuni OXC8506	PubMLST	28697	5136	0464	jejuni	no	yes	no
Campylobacter jejuni OXC8510	PubMLST	28701	5136	0464	jejuni	no	yes	no
Campylobacter jejuni OXC8512	PubMLST	28703	5136	0464	jejuni	no	yes	no
Campylobacter jejuni OXC8522	PubMLST	28713	5136	0464	jejuni	no	yes	no
Campylobacter jejuni OXC8533	PubMLST	28723	5136	0464	jejuni	no	yes	no
Campylobacter jejuni OXC8577	PubMLST	28914	5136	0464	jejuni	no	yes	no
Campylobacter jejuni OXC8604	PubMLST	28936	5136	0464	jejuni	no	yes	no
Campylobacter jejuni OXC8632	PubMLST	28950	5136	0464	jejuni	no	yes	no
Campylobacter jejuni OXC8669	PubMLST	29002	5136	0464	jejuni	no	yes	no
Campylobacter jejuni OXC8680	PubMLST	29012	5136	0464	jejuni	no	yes	no
Campylobacter jejuni OXC8686	PubMLST	29018	5136	0464	jejuni	no	yes	no
Campylobacter jejuni OXC8690	PubMLST	29022	5136	0464	jejuni	no	yes	no
Campylobacter jejuni OXC8706	PubMLST	29035	5136	0464	jejuni	no	yes	no
Campylobacter jejuni OXC8741	PubMLST	29077	5136	0464	jejuni	no	yes	no
Campylobacter jejuni OXC8769	PubMLST	30458	5136	0464	jejuni	no	yes	no
Campylobacter jejuni OXC8782	PubMLST	30471	5136	0464	jejuni	no	yes	no
Campylobacter jejuni OXC8792	PubMLST	30481	464	0464	jejuni	no	yes	no
Campylobacter jejuni OXC8793	PubMLST	30482	464	0464	jejuni	no	yes	no
Campylobacter jejuni OXC8805	PubMLST	30494	5136	0464	jejuni	no	yes	no
Campylobacter jejuni OXC8813	PubMLST	30501	5136	0464	jejuni	no	yes	no
Campylobacter jejuni OXC8818	PubMLST	30506	464	0464	jejuni	no	yes	no
Campylobacter jejuni OXC8819	PubMLST	30507	5136	0464	jejuni	no	yes	no
Campylobacter jejuni OXC8823	PubMLST	30511	5136	0464	jejuni	no	yes	no
Campylobacter jejuni OXC8830	PubMLST	30518	464	0464	jejuni	no	yes	no
Campylobacter jejuni OXC8840	PubMLST	30526	464	0464	jejuni	no	yes	no
Campylobacter jejuni OXC8845	PubMLST	30531	464	0464	jejuni	no	yes	no
Campylobacter jejuni OXC8855	PubMLST	30541	5136	0464	jejuni	no	yes	no
Campylobacter jejuni OXC8886	PubMLST	30570	464	0464	jejuni	no	yes	no
Campylobacter jejuni OXC5762	PubMLST	21483	508	0508	jejuni	no	yes	no
Campylobacter jejuni OXC6271	PubMLST	16068	508	0508	jejuni	no	yes	no
Campylobacter jejuni OXC6273	PubMLST	16070	508	0508	jejuni	no	yes	no
Campylobacter jejuni OXC6364	PubMLST	16161	132	0508	jejuni	no	yes	no
Campylobacter jejuni OXC6561	PubMLST	12896	132	0508	jejuni	no	yes	no
Campylobacter jejuni OXC6639	PubMLST	16388	132	0508	jejuni	no	yes	no
Campylobacter jejuni OXC6660	PubMLST	18217	508	0508	jejuni	no	yes	no
Campylobacter jejuni OXC6773	PubMLST	18330	508	0508	jejuni	no	yes	no
Campylobacter jejuni OXC7422	PubMLST	23956	132	0508	jejuni	no	yes	no
Campylobacter jejuni OXC7473	PubMLST	24002	508	0508	jejuni	no	yes	no
Campylobacter jejuni OXC7503	PubMLST	24029	132	0508	jejuni	no	yes	no
Campylobacter jejuni OXC7527	PubMLST	24131	508	0508	jejuni	no	yes	no
Campylobacter jejuni OXC7531	PubMLST	24474	508	0508	jejuni	no	yes	no
Campylobacter jejuni OXC7536	PubMLST	24479	508	0508	jejuni	no	yes	no
Campylobacter jejuni OXC7789	PubMLST	24592	508	0508	jejuni	no	yes	no
Campylobacter jejuni OXC7977	PubMLST	25392	508	0508	jejuni	no	yes	no
Campylobacter jejuni OXC8110	PubMLST	25615	508	0508	jejuni	no	yes	no
Campylobacter jejuni OXC8283	PubMLST	27975	508	0508	jejuni	no	yes	no
Campylobacter jejuni OXC8310	PubMLST	28001	508	0508	jejuni	no	yes	no
Campylobacter jejuni OXC8313	PubMLST	28004	508	0508	jejuni	no	yes	no
Campylobacter jejuni OXC8377	PubMLST	28571	508	0508	jejuni	no	yes	no
Campylobacter jejuni OXC8902	PubMLST	30585	508	0508	jejuni	no	yes	no
Campylobacter jejuni OXC8990	PubMLST	30672	508	0508	jejuni	no	yes	no
Campylobacter jejuni OXC5211	PubMLST	24800	573	0573	jejuni	no	yes	no
Campylobacter jejuni OXC5286	PubMLST	24844	573	0573	jejuni	no	yes	no
Campylobacter jejuni OXC5795	PubMLST	23545	573	0573	jejuni	no	yes	no
Campylobacter jejuni OXC5894	PubMLST	23624	573	0573	jejuni	no	yes	no
Campylobacter jejuni OXC6433	PubMLST	16228	573	0573	jejuni	no	yes	no
Campylobacter jejuni OXC6435	PubMLST	16230	573	0573	jejuni	no	yes	no
Campylobacter jejuni OXC6542	PubMLST	16334	573	0573	jejuni	no	yes	no
Campylobacter jejuni OXC6595	PubMLST	16346	573	0573	jejuni	no	yes	no
Campylobacter jejuni OXC6595_V2	PubMLST	24175	573	0573	jejuni	no	yes	no
Campylobacter jejuni OXC6595R	PubMLST	21179	573	0573	jejuni	no	yes	no
Campylobacter jejuni OXC6595R_V2	PubMLST	24185	573	0573	jejuni	no	yes	no
Campylobacter jejuni OXC6610	PubMLST	16360	573	0573	jejuni	no	yes	no
Campylobacter jejuni OXC6610_V2	PubMLST	24176	573	0573	jejuni	no	yes	no
Campylobacter jejuni OXC6610R	PubMLST	21180	573	0573	jejuni	no	yes	no
Campylobacter jejuni OXC6610R_V2	PubMLST	24186	573	0573	jejuni	no	yes	no
Campylobacter jejuni OXC6788	PubMLST	18345	573	0573	jejuni	no	yes	no

Campylobacter jejuni OXC6794	PubMLST	18351	573	0573	jejuni	no	yes	no
Campylobacter jejuni OXC6878	PubMLST	21597	573	0573	jejuni	no	yes	no
Campylobacter jejuni OXC6965	PubMLST	21139	573	0573	jejuni	no	yes	no
Campylobacter jejuni OXC7123	PubMLST	21205	573	0573	jejuni	no	yes	no
Campylobacter jejuni OXC7676	PubMLST	24930	573	0573	jejuni	no	yes	no
Campylobacter jejuni OXC8334	PubMLST	28025	573	0573	jejuni	no	yes	no
Campylobacter jejuni OXC8439	PubMLST	28630	573	0573	jejuni	no	yes	no
Campylobacter jejuni OXC8440	PubMLST	28631	573	0573	jejuni	no	yes	no
Campylobacter jejuni OXC8583	PubMLST	28919	573	0573	jejuni	no	yes	no
Campylobacter jejuni OXC8758	PubMLST	30448	573	0573	jejuni	no	yes	no
Campylobacter jejuni OXC4969	PubMLST	24660	3172	0574	jejuni	no	yes	no
Campylobacter jejuni OXC8770	PubMLST	30459	3015	0574	jejuni	no	yes	no
Campylobacter jejuni OXC4564	PubMLST	24042	1900	0658	jejuni	no	yes	no
Campylobacter jejuni OXC5396	PubMLST	21356	1395	0658	jejuni	no	yes	no
Campylobacter jejuni OXC5733	PubMLST	21463	312	0658	jejuni	no	yes	no
Campylobacter jejuni OXC5882	PubMLST	23614	312	0658	jejuni	no	yes	no
Campylobacter jejuni OXC6506	PubMLST	16298	1044	0658	jejuni	no	yes	no
Campylobacter jejuni OXC6578	PubMLST	12913	312	0658	jejuni	no	yes	no
Campylobacter jejuni OXC6578_V2	PubMLST	24178	312	0658	jejuni	no	yes	no
Campylobacter jejuni OXC6578R	PubMLST	21182	312	0658	jejuni	no	yes	no
Campylobacter jejuni OXC6578R_V2	PubMLST	24188	312	0658	jejuni	no	yes	no
Campylobacter jejuni OXC7010	PubMLST	22197	312	0658	jejuni	no	yes	no
Campylobacter jejuni OXC7459	PubMLST	23989	1044	0658	jejuni	no	yes	no
Campylobacter jejuni OXC7900	PubMLST	25031	1044	0658	jejuni	no	yes	no
Campylobacter jejuni OXC8329	PubMLST	28020	1044	0658	jejuni	no	yes	no
Campylobacter jejuni OXC8460	PubMLST	28651	312	0658	jejuni	no	yes	no
Campylobacter jejuni OXC8461R	PubMLST	28653	312	0658	jejuni	no	yes	no
Campylobacter jejuni H120260533	PubMLST	29415	7330	0661	jejuni	no	yes	no
Campylobacter jejuni H122720463	PubMLST	2646	677	0677	jejuni	no	yes	no
Campylobacter jejuni LMG 9872	Genbank/EMBL/DDBJ	AIPM01	677	0677	jejuni	no	yes	no
Campylobacter jejuni OXC5341	PubMLST	21323	794	0677	jejuni	no	yes	no
Campylobacter jejuni OXC6332	PubMLST	16129	677	0677	jejuni	no	yes	no
Campylobacter jejuni OXC7095	PubMLST	22277	677	0677	jejuni	no	yes	no
Campylobacter jejuni OXC7345	PubMLST	23885	677	0677	jejuni	no	yes	no
Campylobacter jejuni OXC7358	PubMLST	23897	677	0677	jejuni	no	yes	no
Campylobacter jejuni OXC8290	PubMLST	27981	6514	0677	jejuni	no	yes	no
Campylobacter jejuni OXC8926	PubMLST	30609	6514	0677	jejuni	no	yes	no
Campylobacter jejuni OXC9015	PubMLST	30695	677	0677	jejuni	no	yes	no
Campylobacter jejuni UA5070	Genbank/EMBL/DDBJ	CCXG01	677	0677	jejuni	no	yes	no
Campylobacter jejuni OXC6480	PubMLST	16272	1301	0692	jejuni	no	yes	no
Campylobacter jejuni OXC6777	PubMLST	18334	1301	0692	jejuni	no	yes	no
Campylobacter jejuni OXC7456	PubMLST	23986	1301	0692	jejuni	no	yes	no
Campylobacter jejuni OXC5404	PubMLST	21360	5152	0702	jejuni	no	yes	no
Campylobacter jejuni OXC5669	PubMLST	21416	5153	0702	jejuni	no	yes	no
Campylobacter jejuni OXC6431	PubMLST	16226	1709	1034	jejuni	no	yes	no
Campylobacter jejuni OXC6463	PubMLST	16255	1709	1034	jejuni	no	yes	no
Campylobacter jejuni OXC6593	PubMLST	21507	1709	1034	jejuni	no	yes	no
Campylobacter jejuni OXC7192	PubMLST	22683	5763	1034	jejuni	no	yes	no
Campylobacter jejuni OXC7219	PubMLST	22710	1709	1034	jejuni	no	yes	no
Campylobacter jejuni OXC7299	PubMLST	22340	6235	1034	jejuni	no	yes	no
Campylobacter jejuni OXC7893_R	PubMLST	28058	5763	1034	jejuni	no	yes	no
Campylobacter doylei 26997	Genbank/EMBL/DDBJ	NC_009707	1845	0000	doy	no	yes	yes
Campylobacter doylei ATCC 49349	Genbank/EMBL/DDBJ	JNJR01	62	0000	doy	no	yes	yes
Campylobacter jejuni 1213	Genbank/EMBL/DDBJ	AIPG01	N/A	0000	jejuni	no	yes	yes
Campylobacter jejuni 1798	Genbank/EMBL/DDBJ	AIPI01	N/A	0000	jejuni	no	yes	yes
Campylobacter jejuni 1854	Genbank/EMBL/DDBJ	AIPJ01	922	0000	jejuni	no	yes	yes
Campylobacter jejuni 1997-10	Genbank/EMBL/DDBJ	AIOY01	4839	0000	jejuni	no	yes	yes
Campylobacter jejuni 2008-979	Genbank/EMBL/DDBJ	AIOU01	2274	0000	jejuni	no	yes	yes
Campylobacter jejuni 255	Genbank/EMBL/DDBJ	ARWS01	2157	0000	jejuni	no	yes	yes
Campylobacter jejuni 30318	Genbank/EMBL/DDBJ	AUUJ01	4397	0000	jejuni	no	yes	yes
Campylobacter jejuni 60004	Genbank/EMBL/DDBJ	AIOE01	4836	0000	jejuni	no	yes	yes
Campylobacter jejuni 7487	PubMLST	24253	N/A	0000	jejuni	no	yes	yes
Campylobacter jejuni ARI_813_R	PubMLST	24336	N/A	0000	jejuni	no	yes	yes
Campylobacter jejuni BID17E	PubMLST	25071	6838	0000	jejuni	no	yes	yes
Campylobacter jejuni BID18W	PubMLST	25074	6838	0000	jejuni	no	yes	yes
Campylobacter jejuni BID1MV	PubMLST	25076	6839	0000	jejuni	no	yes	yes
Campylobacter jejuni CAMSA2113_rr	PubMLST	25352	N/A	0000	jejuni	no	yes	yes
Campylobacter jejuni CAMSA3844_rr	PubMLST	25360	N/A	0000	jejuni	no	yes	yes
Campylobacter jejuni CAMSA3962_rr	PubMLST	25364	N/A	0000	jejuni	no	yes	yes
Campylobacter jejuni Dg102	PubMLST	26036	586	0000	jejuni	no	yes	yes
Campylobacter jejuni Dg105	PubMLST	26050	N/A	0000	jejuni	no	yes	yes
Campylobacter jejuni Dg147	PubMLST	26008	6562	0000	jejuni	no	yes	yes
Campylobacter jejuni Dg150	PubMLST	26046	6562	0000	jejuni	no	yes	yes
Campylobacter jejuni Dg156	PubMLST	26053	6562	0000	jejuni	no	yes	yes
Campylobacter jejuni Dg157	PubMLST	26060	N/A	0000	jejuni	no	yes	yes
Campylobacter jejuni Dg164	PubMLST	26032	N/A	0000	jejuni	no	yes	yes
Campylobacter jejuni Dg179	PubMLST	25984	N/A	0000	jejuni	no	yes	yes
Campylobacter jejuni Dg189	PubMLST	24194	6562	0000	jejuni	no	yes	yes
Campylobacter jejuni Dg18a	PubMLST	25977	N/A	0000	jejuni	no	yes	yes
Campylobacter jejuni Dg192	PubMLST	24196	6564	0000	jejuni	no	yes	yes
Campylobacter jejuni Dg196	PubMLST	24191	6561	0000	jejuni	no	yes	yes
Campylobacter jejuni Dg197	PubMLST	25992	6562	0000	jejuni	no	yes	yes
Campylobacter jejuni Dg201	PubMLST	25993	N/A	0000	jejuni	no	yes	yes
Campylobacter jejuni Dg204	PubMLST	26010	6561	0000	jejuni	no	yes	yes
Campylobacter jejuni Dg210	PubMLST	26027	7276	0000	jejuni	no	yes	yes
Campylobacter jejuni Dg217	PubMLST	25945	7276	0000	jejuni	no	yes	yes
Campylobacter jejuni Dg22	PubMLST	25974	3471	0000	jejuni	no	yes	yes
Campylobacter jejuni Dg226	PubMLST	26042	6562	0000	jejuni	no	yes	yes
Campylobacter jejuni Dg233	PubMLST	25997	N/A	0000	jejuni	no	yes	yes
Campylobacter jejuni Dg234	PubMLST	26049	N/A	0000	jejuni	no	yes	yes
Campylobacter jejuni Dg23b	PubMLST	25941	6562	0000	jejuni	no	yes	yes
Campylobacter jejuni Dg276	PubMLST	26065	N/A	0000	jejuni	no	yes	yes

Campylobacter jejuni Dg289	PubMLST	26014	N/A	0000	jejuni	no	yes	yes
Campylobacter jejuni Dg295a	PubMLST	26030	N/A	0000	jejuni	no	yes	yes
Campylobacter jejuni Dg300	PubMLST	25968	6562	0000	jejuni	no	yes	yes
Campylobacter jejuni Dg301b	PubMLST	25972	N/A	0000	jejuni	no	yes	yes
Campylobacter jejuni Dg302	PubMLST	25976	6562	0000	jejuni	no	yes	yes
Campylobacter jejuni Dg310	PubMLST	25998	586	0000	jejuni	no	yes	yes
Campylobacter jejuni Dg338	PubMLST	26069	6562	0000	jejuni	no	yes	yes
Campylobacter jejuni Dg346	PubMLST	26017	7278	0000	jejuni	no	yes	yes
Campylobacter jejuni Dg347	PubMLST	26023	N/A	0000	jejuni	no	yes	yes
Campylobacter jejuni Dg348	PubMLST	26025	N/A	0000	jejuni	no	yes	yes
Campylobacter jejuni Dg358	PubMLST	26001	6564	0000	jejuni	no	yes	yes
Campylobacter jejuni Dg36b	PubMLST	25967	N/A	0000	jejuni	no	yes	yes
Campylobacter jejuni Dg381	PubMLST	26002	N/A	0000	jejuni	no	yes	yes
Campylobacter jejuni Dg43a	PubMLST	25978	N/A	0000	jejuni	no	yes	yes
Campylobacter jejuni Dg47b	PubMLST	25971	586	0000	jejuni	no	yes	yes
Campylobacter jejuni Dg61	PubMLST	24189	6561	0000	jejuni	no	yes	yes
Campylobacter jejuni Dg62a	PubMLST	24190	6561	0000	jejuni	no	yes	yes
Campylobacter jejuni Dg78a	PubMLST	25939	5129	0000	jejuni	no	yes	yes
Campylobacter jejuni H143040420	PubMLST	2607	441	0000	jejuni	no	yes	yes
Campylobacter jejuni HM908634	PubMLST	24203	N/A	0000	jejuni	no	yes	yes
Campylobacter jejuni LMG 23210	Genbank/EMBL/DDBJ	AIPN01	380	0000	jejuni	no	yes	yes
Campylobacter jejuni NCCP 15742	Genbank/EMBL/DDBJ	APJU01	N/A	0000	jejuni	no	yes	yes
Campylobacter jejuni OXC4557	PubMLST	25084	693	0000	jejuni	no	yes	yes
Campylobacter jejuni OXC4605	PubMLST	22127	N/A	0000	jejuni	no	yes	yes
Campylobacter jejuni OXC4645	PubMLST	24091	436	0000	jejuni	no	yes	yes
Campylobacter jejuni OXC4653	PubMLST	24096	436	0000	jejuni	no	yes	yes
Campylobacter jejuni OXC4746	PubMLST	18448	436	0000	jejuni	no	yes	yes
Campylobacter jejuni OXC4802	PubMLST	22142	1409	0000	jejuni	no	yes	yes
Campylobacter jejuni OXC4808	PubMLST	22145	879	0000	jejuni	no	yes	yes
Campylobacter jejuni OXC4906	PubMLST	24466	4766	0000	jejuni	no	yes	yes
Campylobacter jejuni OXC4916	PubMLST	25466	2274	0000	jejuni	no	yes	yes
Campylobacter jejuni OXC4991	PubMLST	24674	4397	0000	jejuni	no	yes	yes
Campylobacter jejuni OXC5033	PubMLST	24700	2131	0000	jejuni	no	yes	yes
Campylobacter jejuni OXC5088	PubMLST	24729	4430	0000	jejuni	no	yes	yes
Campylobacter jejuni OXC5279	PubMLST	24841	531	0000	jejuni	no	yes	yes
Campylobacter jejuni OXC5302	PubMLST	24849	N/A	0000	jejuni	no	yes	yes
Campylobacter jejuni OXC5336	PubMLST	21320	2131	0000	jejuni	no	yes	yes
Campylobacter jejuni OXC5337	PubMLST	21321	2131	0000	jejuni	no	yes	yes
Campylobacter jejuni OXC5338	PubMLST	21322	N/A	0000	jejuni	no	yes	yes
Campylobacter jejuni OXC5440	PubMLST	21385	N/A	0000	jejuni	no	yes	yes
Campylobacter jejuni OXC5642	PubMLST	23527	5244	0000	jejuni	no	yes	yes
Campylobacter jejuni OXC5648	PubMLST	23532	N/A	0000	jejuni	no	yes	yes
Campylobacter jejuni OXC5743	PubMLST	21470	586	0000	jejuni	no	yes	yes
Campylobacter jejuni OXC5759	PubMLST	21481	1374	0000	jejuni	no	yes	yes
Campylobacter jejuni OXC5798	PubMLST	23548	441	0000	jejuni	no	yes	yes
Campylobacter jejuni OXC5803	PubMLST	23553	N/A	0000	jejuni	no	yes	yes
Campylobacter jejuni OXC5824	PubMLST	23572	3268	0000	jejuni	no	yes	yes
Campylobacter jejuni OXC5828	PubMLST	23663	N/A	0000	jejuni	no	yes	yes
Campylobacter jejuni OXC5858	PubMLST	23598	5244	0000	jejuni	no	yes	yes
Campylobacter jejuni OXC5892	PubMLST	23623	2304	0000	jejuni	no	yes	yes
Campylobacter jejuni OXC5897	PubMLST	23627	4684	0000	jejuni	no	yes	yes
Campylobacter jejuni OXC5904	PubMLST	23678	986	0000	jejuni	no	yes	yes
Campylobacter jejuni OXC6256	PubMLST	16053	2274	0000	jejuni	no	yes	yes
Campylobacter jejuni OXC6279	PubMLST	16076	2258	0000	jejuni	no	yes	yes
Campylobacter jejuni OXC6281	PubMLST	16078	2274	0000	jejuni	no	yes	yes
Campylobacter jejuni OXC6322	PubMLST	16119	2274	0000	jejuni	no	yes	yes
Campylobacter jejuni OXC6353	PubMLST	16150	5729	0000	jejuni	no	yes	yes
Campylobacter jejuni OXC6360	PubMLST	16157	2274	0000	jejuni	no	yes	yes
Campylobacter jejuni OXC6374	PubMLST	16171	5739	0000	jejuni	no	yes	yes
Campylobacter jejuni OXC6446	PubMLST	16239	3029	0000	jejuni	no	yes	yes
Campylobacter jejuni OXC6486	PubMLST	16278	2274	0000	jejuni	no	yes	yes
Campylobacter jejuni OXC6528	PubMLST	16320	5738	0000	jejuni	no	yes	yes
Campylobacter jejuni OXC6544	PubMLST	10935	1911	0000	jejuni	no	yes	yes
Campylobacter jejuni OXC6546	PubMLST	12881	449	0000	jejuni	no	yes	yes
Campylobacter jejuni OXC6557	PubMLST	12892	5756	0000	jejuni	no	yes	yes
Campylobacter jejuni OXC6628	PubMLST	16377	441	0000	jejuni	no	yes	yes
Campylobacter jejuni OXC6634	PubMLST	16383	882	0000	jejuni	no	yes	yes
Campylobacter jejuni OXC6674	PubMLST	18231	441	0000	jejuni	no	yes	yes
Campylobacter jejuni OXC6675	PubMLST	18232	986	0000	jejuni	no	yes	yes
Campylobacter jejuni OXC6686	PubMLST	18243	4373	0000	jejuni	no	yes	yes
Campylobacter jejuni OXC6695	PubMLST	18252	441	0000	jejuni	no	yes	yes
Campylobacter jejuni OXC6733	PubMLST	18290	5807	0000	jejuni	no	yes	yes
Campylobacter jejuni OXC6830	PubMLST	18386	5818	0000	jejuni	no	yes	yes
Campylobacter jejuni OXC6831	PubMLST	18387	2274	0000	jejuni	no	yes	yes
Campylobacter jejuni OXC6842	PubMLST	21561	2274	0000	jejuni	no	yes	yes
Campylobacter jejuni OXC6844	PubMLST	21563	2118	0000	jejuni	no	yes	yes
Campylobacter jejuni OXC6871	PubMLST	21590	5326	0000	jejuni	no	yes	yes
Campylobacter jejuni OXC6897	PubMLST	22092	2274	0000	jejuni	no	yes	yes
Campylobacter jejuni OXC6912	PubMLST	22107	1962	0000	jejuni	no	yes	yes
Campylobacter jejuni OXC6944	PubMLST	21118	986	0000	jejuni	no	yes	yes
Campylobacter jejuni OXC6997	PubMLST	21168	6136	0000	jejuni	no	yes	yes
Campylobacter jejuni OXC7013	PubMLST	22200	436	0000	jejuni	no	yes	yes
Campylobacter jejuni OXC7205	PubMLST	22696	5686	0000	jejuni	no	yes	yes
Campylobacter jejuni OXC7258	PubMLST	22299	2274	0000	jejuni	no	yes	yes
Campylobacter jejuni OXC7276	PubMLST	22317	441	0000	jejuni	no	yes	yes
Campylobacter jejuni OXC7386	PubMLST	23924	449	0000	jejuni	no	yes	yes
Campylobacter jejuni OXC7399	PubMLST	25450	436	0000	jejuni	no	yes	yes
Campylobacter jejuni OXC7454	PubMLST	23984	879	0000	jejuni	no	yes	yes
Campylobacter jejuni OXC7458	PubMLST	23988	3923	0000	jejuni	no	yes	yes
Campylobacter jejuni OXC7475	PubMLST	24004	2304	0000	jejuni	no	yes	yes
Campylobacter jejuni OXC7478	PubMLST	24007	5707	0000	jejuni	no	yes	yes
Campylobacter jejuni OXC7515	PubMLST	24120	2274	0000	jejuni	no	yes	yes

Campylobacter jejuni OXC7517	PubMLST	24122	2258	0000	jejuni	no	yes	yes
Campylobacter jejuni OXC7554	PubMLST	24492	6758	0000	jejuni	no	yes	yes
Campylobacter jejuni OXC7592	PubMLST	24524	2274	0000	jejuni	no	yes	yes
Campylobacter jejuni OXC7663	PubMLST	24918	2274	0000	jejuni	no	yes	yes
Campylobacter jejuni OXC7719	PubMLST	24961	2274	0000	jejuni	no	yes	yes
Campylobacter jejuni OXC7817	PubMLST	27885	6974	0000	jejuni	no	yes	yes
Campylobacter jejuni OXC7825	PubMLST	24622	2274	0000	jejuni	no	yes	yes
Campylobacter jejuni OXC7852	PubMLST	24649	449	0000	jejuni	no	yes	yes
Campylobacter jejuni OXC7863	PubMLST	24998	N/A	0000	jejuni	no	yes	yes
Campylobacter jejuni OXC7901	PubMLST	25032	131	0000	jejuni	no	yes	yes
Campylobacter jejuni OXC8005	PubMLST	25489	2274	0000	jejuni	no	yes	yes
Campylobacter jejuni OXC8009	PubMLST	25580	2274	0000	jejuni	no	yes	yes
Campylobacter jejuni OXC8024	PubMLST	25410	6449	0000	jejuni	no	yes	yes
Campylobacter jejuni OXC8054	PubMLST	25599	N/A	0000	jejuni	no	yes	yes
Campylobacter jejuni OXC8056	PubMLST	25414	6449	0000	jejuni	no	yes	yes
Campylobacter jejuni OXC8087	PubMLST	25521	436	0000	jejuni	no	yes	yes
Campylobacter jejuni OXC8182	PubMLST	25649	5970	0000	jejuni	no	yes	yes
Campylobacter jejuni OXC8191	PubMLST	27896	407	0000	jejuni	no	yes	yes
Campylobacter jejuni OXC8193	PubMLST	27898	N/A	0000	jejuni	no	yes	yes
Campylobacter jejuni OXC8242	PubMLST	27942	1374	0000	jejuni	no	yes	yes
Campylobacter jejuni OXC8255	PubMLST	27953	1409	0000	jejuni	no	yes	yes
Campylobacter jejuni OXC8271	PubMLST	27964	N/A	0000	jejuni	no	yes	yes
Campylobacter jejuni OXC8275	PubMLST	27968	4067	0000	jejuni	no	yes	yes
Campylobacter jejuni OXC8297	PubMLST	27988	436	0000	jejuni	no	yes	yes
Campylobacter jejuni OXC8321	PubMLST	28012	6976	0000	jejuni	no	yes	yes
Campylobacter jejuni OXC8324	PubMLST	28015	441	0000	jejuni	no	yes	yes
Campylobacter jejuni OXC8332	PubMLST	28023	441	0000	jejuni	no	yes	yes
Campylobacter jejuni OXC8373	PubMLST	27873	6982	0000	jejuni	no	yes	yes
Campylobacter jejuni OXC8390	PubMLST	28583	2258	0000	jejuni	no	yes	yes
Campylobacter jejuni OXC8391	PubMLST	28584	2258	0000	jejuni	no	yes	yes
Campylobacter jejuni OXC8404	PubMLST	28596	2274	0000	jejuni	no	yes	yes
Campylobacter jejuni OXC8417	PubMLST	28608	2274	0000	jejuni	no	yes	yes
Campylobacter jejuni OXC8441	PubMLST	28632	7182	0000	jejuni	no	yes	yes
Campylobacter jejuni OXC8491	PubMLST	28683	1030	0000	jejuni	no	yes	yes
Campylobacter jejuni OXC8504	PubMLST	28695	881	0000	jejuni	no	yes	yes
Campylobacter jejuni OXC8603	PubMLST	28935	2328	0000	jejuni	no	yes	yes
Campylobacter jejuni OXC8607	PubMLST	28937	2328	0000	jejuni	no	yes	yes
Campylobacter jejuni OXC8608	PubMLST	28938	2328	0000	jejuni	no	yes	yes
Campylobacter jejuni OXC8610	PubMLST	28940	2274	0000	jejuni	no	yes	yes
Campylobacter jejuni OXC8622	PubMLST	28946	2211	0000	jejuni	no	yes	yes
Campylobacter jejuni OXC8631	PubMLST	28949	881	0000	jejuni	no	yes	yes
Campylobacter jejuni OXC8633	PubMLST	28951	881	0000	jejuni	no	yes	yes
Campylobacter jejuni OXC8638	PubMLST	28954	1962	0000	jejuni	no	yes	yes
Campylobacter jejuni OXC8639	PubMLST	28955	1962	0000	jejuni	no	yes	yes
Campylobacter jejuni OXC8642	PubMLST	29087	N/A	0000	jejuni	no	yes	yes
Campylobacter jejuni OXC8692	PubMLST	29024	1045	0000	jejuni	no	yes	yes
Campylobacter jejuni OXC8711	PubMLST	29040	7306	0000	jejuni	no	yes	yes
Campylobacter jejuni OXC8717	PubMLST	29045	881	0000	jejuni	no	yes	yes
Campylobacter jejuni OXC8726	PubMLST	29051	441	0000	jejuni	no	yes	yes
Campylobacter jejuni OXC8732	PubMLST	29057	7306	0000	jejuni	no	yes	yes
Campylobacter jejuni OXC8733	PubMLST	29058	7306	0000	jejuni	no	yes	yes
Campylobacter jejuni OXC8755	PubMLST	30428	881	0000	jejuni	no	yes	yes
Campylobacter jejuni OXC8759	PubMLST	30449	N/A	0000	jejuni	no	yes	yes
Campylobacter jejuni OXC8761	PubMLST	30451	881	0000	jejuni	no	yes	yes
Campylobacter jejuni OXC8773	PubMLST	30462	2328	0000	jejuni	no	yes	yes
Campylobacter jejuni OXC8774	PubMLST	30463	2328	0000	jejuni	no	yes	yes
Campylobacter jejuni OXC8780	PubMLST	30469	441	0000	jejuni	no	yes	yes
Campylobacter jejuni OXC8799	PubMLST	30488	441	0000	jejuni	no	yes	yes
Campylobacter jejuni OXC8811	PubMLST	30500	441	0000	jejuni	no	yes	yes
Campylobacter jejuni OXC8817	PubMLST	30505	881	0000	jejuni	no	yes	yes
Campylobacter jejuni OXC8821	PubMLST	30509	441	0000	jejuni	no	yes	yes
Campylobacter jejuni OXC8827	PubMLST	30515	881	0000	jejuni	no	yes	yes
Campylobacter jejuni OXC8849	PubMLST	30535	N/A	0000	jejuni	no	yes	yes
Campylobacter jejuni OXC8864	PubMLST	30550	2133	0000	jejuni	no	yes	yes
Campylobacter jejuni OXC8865	PubMLST	30551	881	0000	jejuni	no	yes	yes
Campylobacter jejuni OXC8890	PubMLST	30574	881	0000	jejuni	no	yes	yes
Campylobacter jejuni OXC8903	PubMLST	30586	N/A	0000	jejuni	no	yes	yes
Campylobacter jejuni OXC8908	PubMLST	30591	881	0000	jejuni	no	yes	yes
Campylobacter jejuni OXC8910	PubMLST	30593	441	0000	jejuni	no	yes	yes
Campylobacter jejuni OXC8911	PubMLST	30594	881	0000	jejuni	no	yes	yes
Campylobacter jejuni OXC8927	PubMLST	30610	881	0000	jejuni	no	yes	yes
Campylobacter jejuni OXC8934	PubMLST	30617	881	0000	jejuni	no	yes	yes
Campylobacter jejuni OXC8949	PubMLST	30632	N/A	0000	jejuni	no	yes	yes
Campylobacter jejuni OXC8955	PubMLST	30638	N/A	0000	jejuni	no	yes	yes
Campylobacter jejuni OXC8959	PubMLST	30430	881	0000	jejuni	no	yes	yes
Campylobacter jejuni OXC8989	PubMLST	30671	881	0000	jejuni	no	yes	yes
Campylobacter jejuni UCT046_R	PubMLST	24316	6094	0000	jejuni	no	yes	yes
Campylobacter jejuni W260a	PubMLST	24251	2381	0000	jejuni	no	yes	yes
Campylobacter jejuni 00-2425	Genbank/EMBL/DDBJ	NC_022362	21	0021	jejuni	no	yes	yes
Campylobacter jejuni 00-2426	Genbank/EMBL/DDBJ	NC_022352	21	0021	jejuni	no	yes	yes
Campylobacter jejuni 00-2538	Genbank/EMBL/DDBJ	NC_022351	21	0021	jejuni	no	yes	yes
Campylobacter jejuni 00-2544	Genbank/EMBL/DDBJ	NC_022353	21	0021	jejuni	no	yes	yes
Campylobacter jejuni 04197	Genbank/EMBL/DDBJ	CAFU01	21	0021	jejuni	no	yes	yes
Campylobacter jejuni 04199	Genbank/EMBL/DDBJ	CAFV01	21	0021	jejuni	no	yes	yes
Campylobacter jejuni 1_12S	Genbank/EMBL/DDBJ	CCCY01	50	0021	jejuni	no	yes	yes
Campylobacter jejuni 110-21	Genbank/EMBL/DDBJ	AIPC01	982	0021	jejuni	no	yes	yes
Campylobacter jejuni 1928	Genbank/EMBL/DDBJ	AIPL01	806	0021	jejuni	no	yes	yes
Campylobacter jejuni 2008-1025	Genbank/EMBL/DDBJ	AIOP01	50	0021	jejuni	no	yes	yes
Campylobacter jejuni 2008-831	Genbank/EMBL/DDBJ	AIOV01	50	0021	jejuni	no	yes	yes
Campylobacter jejuni 5329_H135060411-1	PubMLST	28832	21	0021	jejuni	no	yes	yes
Campylobacter jejuni 5330_H135060412-1	PubMLST	28833	21	0021	jejuni	no	yes	yes
Campylobacter jejuni 5331_H135080149-1	PubMLST	28834	21	0021	jejuni	no	yes	yes

Campylobacter jejuni 5332_H135080150-1	PubMLST	28835	21	0021	jejuni	no	yes	yes
Campylobacter jejuni 5333_H135100351-1	PubMLST	28836	21	0021	jejuni	no	yes	yes
Campylobacter jejuni 5334_H135100352-1	PubMLST	28837	21	0021	jejuni	no	yes	yes
Campylobacter jejuni 5335_H135100353-1	PubMLST	28838	21	0021	jejuni	no	yes	yes
Campylobacter jejuni 5336_H135140732-1	PubMLST	28839	21	0021	jejuni	no	yes	yes
Campylobacter jejuni 5338_H135200111-1	PubMLST	28840	21	0021	jejuni	no	yes	yes
Campylobacter jejuni 6399	Genbank/EMBL/DDBJ	CAFT01	21	0021	jejuni	no	yes	yes
Campylobacter jejuni 84-25	Genbank/EMBL/DDBJ	AANT02	21	0021	jejuni	no	yes	yes
Campylobacter jejuni 87330	Genbank/EMBL/DDBJ	AIPD01	50	0021	jejuni	no	yes	yes
Campylobacter jejuni ARI_1022_R	PubMLST	24321	883	0021	jejuni	no	yes	yes
Campylobacter jejuni ARI_1130_R	PubMLST	24325	50	0021	jejuni	no	yes	yes
Campylobacter jejuni ARI_1232_R	PubMLST	24327	19	0021	jejuni	no	yes	yes
Campylobacter jejuni ARI_1331_R	PubMLST	24328	21	0021	jejuni	no	yes	yes
Campylobacter jejuni ARI_814_R	PubMLST	24337	21	0021	jejuni	no	yes	yes
Campylobacter jejuni ARI_916_R	PubMLST	24319	50	0021	jejuni	no	yes	yes
Campylobacter jejuni BID1NL	PubMLST	25123	21	0021	jejuni	no	yes	yes
Campylobacter jejuni C_389_R	PubMLST	24334	53	0021	jejuni	no	yes	yes
Campylobacter jejuni C_391_R	PubMLST	24335	53	0021	jejuni	no	yes	yes
Campylobacter jejuni C101794	PubMLST	28876	50	0021	jejuni	no	yes	yes
Campylobacter jejuni C120949	PubMLST	28878	50	0021	jejuni	no	yes	yes
Campylobacter jejuni C121664	PubMLST	28879	50	0021	jejuni	no	yes	yes
Campylobacter jejuni C130520	PubMLST	28880	50	0021	jejuni	no	yes	yes
Campylobacter jejuni C258_cj_R	PubMLST	24333	2135	0021	jejuni	no	yes	yes
Campylobacter jejuni CAMSA100	PubMLST	22774	21	0021	jejuni	no	yes	yes
Campylobacter jejuni CAMSA1000	PubMLST	22813	21	0021	jejuni	no	yes	yes
Campylobacter jejuni CAMSA1004	PubMLST	22814	21	0021	jejuni	no	yes	yes
Campylobacter jejuni CAMSA1006	PubMLST	22815	21	0021	jejuni	no	yes	yes
Campylobacter jejuni CAMSA1008	PubMLST	22816	21	0021	jejuni	no	yes	yes
Campylobacter jejuni CAMSA1020	PubMLST	22817	21	0021	jejuni	no	yes	yes
Campylobacter jejuni CAMSA1021	PubMLST	22818	21	0021	jejuni	no	yes	yes
Campylobacter jejuni CAMSA1028	PubMLST	22819	21	0021	jejuni	no	yes	yes
Campylobacter jejuni CAMSA1044	PubMLST	22820	21	0021	jejuni	no	yes	yes
Campylobacter jejuni CAMSA1052	PubMLST	22821	21	0021	jejuni	no	yes	yes
Campylobacter jejuni CAMSA1055	PubMLST	22822	21	0021	jejuni	no	yes	yes
Campylobacter jejuni CAMSA1073	PubMLST	22823	21	0021	jejuni	no	yes	yes
Campylobacter jejuni CAMSA1080	PubMLST	22824	21	0021	jejuni	no	yes	yes
Campylobacter jejuni CAMSA141	PubMLST	22775	21	0021	jejuni	no	yes	yes
Campylobacter jejuni CAMSA148	PubMLST	22776	21	0021	jejuni	no	yes	yes
Campylobacter jejuni CAMSA148_R	PubMLST	24310	21	0021	jejuni	no	yes	yes
Campylobacter jejuni CAMSA15	PubMLST	18467	21	0021	jejuni	no	yes	yes
Campylobacter jejuni CAMSA15_R	PubMLST	24311	21	0021	jejuni	no	yes	yes
Campylobacter jejuni CAMSA153	PubMLST	22777	21	0021	jejuni	no	yes	yes
Campylobacter jejuni CAMSA163	PubMLST	22778	21	0021	jejuni	no	yes	yes
Campylobacter jejuni CAMSA182	PubMLST	22779	21	0021	jejuni	no	yes	yes
Campylobacter jejuni CAMSA2002	PubMLST	22825	21	0021	jejuni	no	yes	yes
Campylobacter jejuni CAMSA2005	PubMLST	22826	21	0021	jejuni	no	yes	yes
Campylobacter jejuni CAMSA2021	PubMLST	22827	21	0021	jejuni	no	yes	yes
Campylobacter jejuni CAMSA2033	PubMLST	22829	21	0021	jejuni	no	yes	yes
Campylobacter jejuni CAMSA2033_rr	PubMLST	25349	21	0021	jejuni	no	yes	yes
Campylobacter jejuni CAMSA2036	PubMLST	22830	21	0021	jejuni	no	yes	yes
Campylobacter jejuni CAMSA2038	PubMLST	22831	21	0021	jejuni	no	yes	yes
Campylobacter jejuni CAMSA2049	PubMLST	22832	21	0021	jejuni	no	yes	yes
Campylobacter jejuni CAMSA2056	PubMLST	22833	21	0021	jejuni	no	yes	yes
Campylobacter jejuni CAMSA2057	PubMLST	22834	21	0021	jejuni	no	yes	yes
Campylobacter jejuni CAMSA206	PubMLST	22780	21	0021	jejuni	no	yes	yes
Campylobacter jejuni CAMSA2075	PubMLST	22835	21	0021	jejuni	no	yes	yes
Campylobacter jejuni CAMSA2086	PubMLST	22836	21	0021	jejuni	no	yes	yes
Campylobacter jejuni CAMSA2099	PubMLST	22837	21	0021	jejuni	no	yes	yes
Campylobacter jejuni CAMSA2099_rr	PubMLST	25350	21	0021	jejuni	no	yes	yes
Campylobacter jejuni CAMSA2102	PubMLST	22838	21	0021	jejuni	no	yes	yes
Campylobacter jejuni CAMSA2112	PubMLST	22839	21	0021	jejuni	no	yes	yes
Campylobacter jejuni CAMSA2112_rr	PubMLST	25351	21	0021	jejuni	no	yes	yes
Campylobacter jejuni CAMSA2113	PubMLST	22840	21	0021	jejuni	no	yes	yes
Campylobacter jejuni CAMSA2115	PubMLST	22841	21	0021	jejuni	no	yes	yes
Campylobacter jejuni CAMSA2147	PubMLST	22842	21	0021	jejuni	no	yes	yes
Campylobacter jejuni CAMSA2153	PubMLST	22843	21	0021	jejuni	no	yes	yes
Campylobacter jejuni CAMSA2164	PubMLST	22844	21	0021	jejuni	no	yes	yes
Campylobacter jejuni CAMSA2175	PubMLST	22845	21	0021	jejuni	no	yes	yes
Campylobacter jejuni CAMSA2198	PubMLST	22846	21	0021	jejuni	no	yes	yes
Campylobacter jejuni CAMSA2200	PubMLST	22847	21	0021	jejuni	no	yes	yes
Campylobacter jejuni CAMSA2200_rr	PubMLST	25353	21	0021	jejuni	no	yes	yes
Campylobacter jejuni CAMSA2201	PubMLST	22848	21	0021	jejuni	no	yes	yes
Campylobacter jejuni CAMSA226	PubMLST	22781	21	0021	jejuni	no	yes	yes
Campylobacter jejuni CAMSA244	PubMLST	22782	21	0021	jejuni	no	yes	yes
Campylobacter jejuni CAMSA245	PubMLST	22783	21	0021	jejuni	no	yes	yes
Campylobacter jejuni CAMSA25	PubMLST	22146	21	0021	jejuni	no	yes	yes
Campylobacter jejuni CAMSA257	PubMLST	22784	21	0021	jejuni	no	yes	yes
Campylobacter jejuni CAMSA259	PubMLST	22785	21	0021	jejuni	no	yes	yes
Campylobacter jejuni CAMSA26	PubMLST	22147	21	0021	jejuni	no	yes	yes
Campylobacter jejuni CAMSA272	PubMLST	22787	21	0021	jejuni	no	yes	yes
Campylobacter jejuni CAMSA275	PubMLST	22788	21	0021	jejuni	no	yes	yes
Campylobacter jejuni CAMSA289	PubMLST	22789	21	0021	jejuni	no	yes	yes
Campylobacter jejuni CAMSA293	PubMLST	22790	21	0021	jejuni	no	yes	yes
Campylobacter jejuni CAMSA301	PubMLST	22791	21	0021	jejuni	no	yes	yes
Campylobacter jejuni CAMSA311	PubMLST	22792	21	0021	jejuni	no	yes	yes
Campylobacter jejuni CAMSA322	PubMLST	22793	21	0021	jejuni	no	yes	yes
Campylobacter jejuni CAMSA343	PubMLST	22795	21	0021	jejuni	no	yes	yes
Campylobacter jejuni CAMSA350	PubMLST	22796	21	0021	jejuni	no	yes	yes
Campylobacter jejuni CAMSA354	PubMLST	22797	21	0021	jejuni	no	yes	yes
Campylobacter jejuni CAMSA354_rr	PubMLST	25348	21	0021	jejuni	no	yes	yes
Campylobacter jejuni CAMSA357	PubMLST	22798	21	0021	jejuni	no	yes	yes
Campylobacter jejuni CAMSA3605	PubMLST	22849	21	0021	jejuni	no	yes	yes

Campylobacter jejuni CAMSA361	PubMLST	22799	21	0021	jejuni	no	yes	yes
Campylobacter jejuni CAMSA3615	PubMLST	22850	21	0021	jejuni	no	yes	yes
Campylobacter jejuni CAMSA362	PubMLST	22800	21	0021	jejuni	no	yes	yes
Campylobacter jejuni CAMSA3632	PubMLST	22851	21	0021	jejuni	no	yes	yes
Campylobacter jejuni CAMSA3632_rr	PubMLST	25354	21	0021	jejuni	no	yes	yes
Campylobacter jejuni CAMSA3634	PubMLST	22852	21	0021	jejuni	no	yes	yes
Campylobacter jejuni CAMSA3643	PubMLST	22853	21	0021	jejuni	no	yes	yes
Campylobacter jejuni CAMSA3644	PubMLST	22854	21	0021	jejuni	no	yes	yes
Campylobacter jejuni CAMSA3665	PubMLST	22855	21	0021	jejuni	no	yes	yes
Campylobacter jejuni CAMSA3665_rr	PubMLST	25355	21	0021	jejuni	no	yes	yes
Campylobacter jejuni CAMSA3666	PubMLST	22856	21	0021	jejuni	no	yes	yes
Campylobacter jejuni CAMSA3669	PubMLST	22857	21	0021	jejuni	no	yes	yes
Campylobacter jejuni CAMSA3671	PubMLST	22858	21	0021	jejuni	no	yes	yes
Campylobacter jejuni CAMSA3674	PubMLST	22859	21	0021	jejuni	no	yes	yes
Campylobacter jejuni CAMSA3681	PubMLST	22860	21	0021	jejuni	no	yes	yes
Campylobacter jejuni CAMSA3681_rr	PubMLST	25356	21	0021	jejuni	no	yes	yes
Campylobacter jejuni CAMSA3683	PubMLST	22861	21	0021	jejuni	no	yes	yes
Campylobacter jejuni CAMSA3690	PubMLST	22862	21	0021	jejuni	no	yes	yes
Campylobacter jejuni CAMSA3701	PubMLST	22863	21	0021	jejuni	no	yes	yes
Campylobacter jejuni CAMSA3703	PubMLST	22864	21	0021	jejuni	no	yes	yes
Campylobacter jejuni CAMSA3731	PubMLST	22865	21	0021	jejuni	no	yes	yes
Campylobacter jejuni CAMSA374	PubMLST	22801	21	0021	jejuni	no	yes	yes
Campylobacter jejuni CAMSA3741	PubMLST	22866	21	0021	jejuni	no	yes	yes
Campylobacter jejuni CAMSA3741_rr	PubMLST	25357	21	0021	jejuni	no	yes	yes
Campylobacter jejuni CAMSA375	PubMLST	22802	21	0021	jejuni	no	yes	yes
Campylobacter jejuni CAMSA3786	PubMLST	22867	21	0021	jejuni	no	yes	yes
Campylobacter jejuni CAMSA379	PubMLST	22803	21	0021	jejuni	no	yes	yes
Campylobacter jejuni CAMSA3797	PubMLST	22868	21	0021	jejuni	no	yes	yes
Campylobacter jejuni CAMSA3801	PubMLST	22869	21	0021	jejuni	no	yes	yes
Campylobacter jejuni CAMSA3802	PubMLST	22870	21	0021	jejuni	no	yes	yes
Campylobacter jejuni CAMSA3802_rr	PubMLST	25358	21	0021	jejuni	no	yes	yes
Campylobacter jejuni CAMSA382	PubMLST	22804	21	0021	jejuni	no	yes	yes
Campylobacter jejuni CAMSA3837	PubMLST	22872	21	0021	jejuni	no	yes	yes
Campylobacter jejuni CAMSA3837_rr	PubMLST	25359	21	0021	jejuni	no	yes	yes
Campylobacter jejuni CAMSA3844	PubMLST	22873	21	0021	jejuni	no	yes	yes
Campylobacter jejuni CAMSA3845	PubMLST	22874	21	0021	jejuni	no	yes	yes
Campylobacter jejuni CAMSA3845_rr	PubMLST	25361	21	0021	jejuni	no	yes	yes
Campylobacter jejuni CAMSA3851	PubMLST	22875	21	0021	jejuni	no	yes	yes
Campylobacter jejuni CAMSA3874	PubMLST	22876	21	0021	jejuni	no	yes	yes
Campylobacter jejuni CAMSA3888	PubMLST	22877	21	0021	jejuni	no	yes	yes
Campylobacter jejuni CAMSA3889	PubMLST	22878	21	0021	jejuni	no	yes	yes
Campylobacter jejuni CAMSA3897	PubMLST	22879	21	0021	jejuni	no	yes	yes
Campylobacter jejuni CAMSA3914	PubMLST	22880	21	0021	jejuni	no	yes	yes
Campylobacter jejuni CAMSA3914_R	PubMLST	24340	21	0021	jejuni	no	yes	yes
Campylobacter jejuni CAMSA3914_rr	PubMLST	25362	21	0021	jejuni	no	yes	yes
Campylobacter jejuni CAMSA3916	PubMLST	22881	21	0021	jejuni	no	yes	yes
Campylobacter jejuni CAMSA3916_R	PubMLST	24341	21	0021	jejuni	no	yes	yes
Campylobacter jejuni CAMSA3916_rr	PubMLST	25363	21	0021	jejuni	no	yes	yes
Campylobacter jejuni CAMSA3928	PubMLST	22882	21	0021	jejuni	no	yes	yes
Campylobacter jejuni CAMSA3943	PubMLST	22883	21	0021	jejuni	no	yes	yes
Campylobacter jejuni CAMSA3962	PubMLST	22884	21	0021	jejuni	no	yes	yes
Campylobacter jejuni CAMSA40	PubMLST	22153	21	0021	jejuni	no	yes	yes
Campylobacter jejuni CAMSA4001	PubMLST	22885	21	0021	jejuni	no	yes	yes
Campylobacter jejuni CAMSA4031	PubMLST	22886	21	0021	jejuni	no	yes	yes
Campylobacter jejuni CAMSA4031_rr	PubMLST	25365	21	0021	jejuni	no	yes	yes
Campylobacter jejuni CAMSA4054	PubMLST	22887	21	0021	jejuni	no	yes	yes
Campylobacter jejuni CAMSA4057	PubMLST	22888	21	0021	jejuni	no	yes	yes
Campylobacter jejuni CAMSA4076	PubMLST	22889	21	0021	jejuni	no	yes	yes
Campylobacter jejuni CAMSA4124	PubMLST	22890	21	0021	jejuni	no	yes	yes
Campylobacter jejuni CAMSA4124_rr	PubMLST	25366	21	0021	jejuni	no	yes	yes
Campylobacter jejuni CAMSA4133	PubMLST	22891	21	0021	jejuni	no	yes	yes
Campylobacter jejuni CAMSA4140	PubMLST	22892	21	0021	jejuni	no	yes	yes
Campylobacter jejuni CAMSA416	PubMLST	22805	21	0021	jejuni	no	yes	yes
Campylobacter jejuni CAMSA418	PubMLST	22806	21	0021	jejuni	no	yes	yes
Campylobacter jejuni CAMSA4186	PubMLST	22893	21	0021	jejuni	no	yes	yes
Campylobacter jejuni CAMSA4186_rr	PubMLST	25367	21	0021	jejuni	no	yes	yes
Campylobacter jejuni CAMSA4196	PubMLST	22894	21	0021	jejuni	no	yes	yes
Campylobacter jejuni CAMSA4196_rr	PubMLST	25368	21	0021	jejuni	no	yes	yes
Campylobacter jejuni CAMSA4206	PubMLST	22895	21	0021	jejuni	no	yes	yes
Campylobacter jejuni CAMSA421	PubMLST	22807	21	0021	jejuni	no	yes	yes
Campylobacter jejuni CAMSA4212	PubMLST	22896	21	0021	jejuni	no	yes	yes
Campylobacter jejuni CAMSA4221	PubMLST	22897	21	0021	jejuni	no	yes	yes
Campylobacter jejuni CAMSA4221_rr	PubMLST	25369	21	0021	jejuni	no	yes	yes
Campylobacter jejuni CAMSA4243	PubMLST	22898	21	0021	jejuni	no	yes	yes
Campylobacter jejuni CAMSA454	PubMLST	22808	21	0021	jejuni	no	yes	yes
Campylobacter jejuni CAMSA457	PubMLST	22809	21	0021	jejuni	no	yes	yes
Campylobacter jejuni CAMSA458	PubMLST	22810	21	0021	jejuni	no	yes	yes
Campylobacter jejuni CAMSA463	PubMLST	22811	21	0021	jejuni	no	yes	yes
Campylobacter jejuni CAMSA463_R	PubMLST	24308	21	0021	jejuni	no	yes	yes
Campylobacter jejuni CAMSA471	PubMLST	22812	21	0021	jejuni	no	yes	yes
Campylobacter jejuni CAMSA471_R	PubMLST	24309	21	0021	jejuni	no	yes	yes
Campylobacter jejuni CAMSA48	PubMLST	22166	21	0021	jejuni	no	yes	yes
Campylobacter jejuni CAMSA55	PubMLST	22167	21	0021	jejuni	no	yes	yes
Campylobacter jejuni CAMSA59	PubMLST	22179	21	0021	jejuni	no	yes	yes
Campylobacter jejuni CAMSA64	PubMLST	22769	21	0021	jejuni	no	yes	yes
Campylobacter jejuni CAMSA75	PubMLST	22770	21	0021	jejuni	no	yes	yes
Campylobacter jejuni CAMSA8	PubMLST	16344	21	0021	jejuni	no	yes	yes
Campylobacter jejuni CAMSA81	PubMLST	22771	21	0021	jejuni	no	yes	yes
Campylobacter jejuni CAMSA95	PubMLST	22772	21	0021	jejuni	no	yes	yes
Campylobacter jejuni CAMSA98	PubMLST	22773	21	0021	jejuni	no	yes	yes
Campylobacter jejuni CF93-6	Genbank/EMBL/DDBJ	AANJ01	883	0021	jejuni	no	yes	yes
Campylobacter jejuni CJ6656_R	PubMLST	24304	53	0021	jejuni	no	yes	yes

Campylobacter jejuni CJ6752_R	PubMLST	24306	2135	0021	jejuni	no	yes	yes
Campylobacter jejuni CJ6850_R	PubMLST	24339	21	0021	jejuni	no	yes	yes
Campylobacter jejuni CJ6946_R	PubMLST	24312	50	0021	jejuni	no	yes	yes
Campylobacter jejuni CJ7015_R	PubMLST	24342	50	0021	jejuni	no	yes	yes
Campylobacter jejuni CJ7256_R	PubMLST	24344	19	0021	jejuni	no	yes	yes
Campylobacter jejuni DFVF1099	Genbank/EMBL/DDBJ	ADHK01	21	0021	jejuni	no	yes	yes
Campylobacter jejuni Dg14b	PubMLST	25951	21	0021	jejuni	no	yes	yes
Campylobacter jejuni Dg16b	PubMLST	25973	21	0021	jejuni	no	yes	yes
Campylobacter jejuni Dg194	PubMLST	26061	21	0021	jejuni	no	yes	yes
Campylobacter jejuni Dg311	PubMLST	26006	53	0021	jejuni	no	yes	yes
Campylobacter jejuni Dg326	PubMLST	25940	21	0021	jejuni	no	yes	yes
Campylobacter jejuni Dg328	PubMLST	26066	21	0021	jejuni	no	yes	yes
Campylobacter jejuni Dg361	PubMLST	25994	21	0021	jejuni	no	yes	yes
Campylobacter jejuni Dg370	PubMLST	26041	50	0021	jejuni	no	yes	yes
Campylobacter jejuni Dg375	PubMLST	26048	19	0021	jejuni	no	yes	yes
Campylobacter jejuni Dg7a	PubMLST	25965	21	0021	jejuni	no	yes	yes
Campylobacter jejuni E110057	PubMLST	28874	50	0021	jejuni	no	yes	yes
Campylobacter jejuni E120350	PubMLST	28875	50	0021	jejuni	no	yes	yes
Campylobacter jejuni E60490	PubMLST	25854	50	0021	jejuni	no	yes	yes
Campylobacter jejuni G113	Genbank/EMBL/DDBJ	AQPK01	43	0021	jejuni	no	yes	yes
Campylobacter jejuni H121820083	PubMLST	2637	50	0021	jejuni	no	yes	yes
Campylobacter jejuni H122580779	PubMLST	2640	50	0021	jejuni	no	yes	yes
Campylobacter jejuni H131020098	PubMLST	2649	53	0021	jejuni	no	yes	yes
Campylobacter jejuni H131020102	PubMLST	2650	53	0021	jejuni	no	yes	yes
Campylobacter jejuni H133640222	PubMLST	2651	50	0021	jejuni	no	yes	yes
Campylobacter jejuni H133640223	PubMLST	2652	50	0021	jejuni	no	yes	yes
Campylobacter jejuni H133640225	PubMLST	2653	50	0021	jejuni	no	yes	yes
Campylobacter jejuni H133640226	PubMLST	2654	50	0021	jejuni	no	yes	yes
Campylobacter jejuni H133640228	PubMLST	2655	50	0021	jejuni	no	yes	yes
Campylobacter jejuni H134900243	PubMLST	2666	21	0021	jejuni	no	yes	yes
Campylobacter jejuni H135060411	PubMLST	2667	21	0021	jejuni	no	yes	yes
Campylobacter jejuni H135060412	PubMLST	2669	21	0021	jejuni	no	yes	yes
Campylobacter jejuni H140620012	PubMLST	2670	50	0021	jejuni	no	yes	yes
Campylobacter jejuni H140940807	PubMLST	2671	50	0021	jejuni	no	yes	yes
Campylobacter jejuni H143820656	PubMLST	31014	917	0021	jejuni	no	yes	yes
Campylobacter jejuni H143820657	PubMLST	31006	917	0021	jejuni	no	yes	yes
Campylobacter jejuni H143900544	PubMLST	31011	917	0021	jejuni	no	yes	yes
Campylobacter jejuni HM0627643	PubMLST	24216	21	0021	jejuni	no	yes	yes
Campylobacter jejuni HM179023	PubMLST	24219	21	0021	jejuni	no	yes	yes
Campylobacter jejuni HM179091	PubMLST	24217	21	0021	jejuni	no	yes	yes
Campylobacter jejuni HM235411	PubMLST	24198	21	0021	jejuni	no	yes	yes
Campylobacter jejuni HM314036	PubMLST	24207	21	0021	jejuni	no	yes	yes
Campylobacter jejuni HM326144	PubMLST	24213	21	0021	jejuni	no	yes	yes
Campylobacter jejuni HM331258	PubMLST	24209	21	0021	jejuni	no	yes	yes
Campylobacter jejuni HM79979	PubMLST	24211	21	0021	jejuni	no	yes	yes
Campylobacter jejuni HM904280	PubMLST	24201	21	0021	jejuni	no	yes	yes
Campylobacter jejuni HM905080	PubMLST	24205	21	0021	jejuni	no	yes	yes
Campylobacter jejuni HM905487	PubMLST	24210	21	0021	jejuni	no	yes	yes
Campylobacter jejuni HM905980	PubMLST	24215	21	0021	jejuni	no	yes	yes
Campylobacter jejuni HM907529	PubMLST	24212	21	0021	jejuni	no	yes	yes
Campylobacter jejuni HM908193	PubMLST	24204	21	0021	jejuni	no	yes	yes
Campylobacter jejuni HM908265	PubMLST	24220	21	0021	jejuni	no	yes	yes
Campylobacter jejuni HM908538	PubMLST	24218	21	0021	jejuni	no	yes	yes
Campylobacter jejuni HM909114	PubMLST	24206	21	0021	jejuni	no	yes	yes
Campylobacter jejuni HM909204	PubMLST	24202	21	0021	jejuni	no	yes	yes
Campylobacter jejuni HM912030	PubMLST	24214	21	0021	jejuni	no	yes	yes
Campylobacter jejuni IA3902	Genbank/EMBL/DDBJ	CP001876	8	0021	jejuni	no	yes	yes
Campylobacter jejuni LMG 9879	Genbank/EMBL/DDBJ	AIOI01	47	0021	jejuni	no	yes	yes
Campylobacter jejuni NCTC 11168	Genbank/EMBL/DDBJ	NC_002163	43	0021	jejuni	no	yes	yes
Campylobacter jejuni NS_35_R	PubMLST	24330	19	0021	jejuni	no	yes	yes
Campylobacter jejuni OXC123	PubMLST	5980	262	0021	jejuni	no	yes	yes
Campylobacter jejuni OXC129	PubMLST	5986	19	0021	jejuni	no	yes	yes
Campylobacter jejuni OXC30	PubMLST	5887	50	0021	jejuni	no	yes	yes
Campylobacter jejuni OXC3817	PubMLST	15545	262	0021	jejuni	no	yes	yes
Campylobacter jejuni OXC39	PubMLST	5896	53	0021	jejuni	no	yes	yes
Campylobacter jejuni OXC4	PubMLST	5861	104	0021	jejuni	no	yes	yes
Campylobacter jejuni OXC4075	PubMLST	15723	104	0021	jejuni	no	yes	yes
Campylobacter jejuni OXC4249	PubMLST	15841	50	0021	jejuni	no	yes	yes
Campylobacter jejuni OXC4503	PubMLST	16028	19	0021	jejuni	no	yes	yes
Campylobacter jejuni OXC4567	PubMLST	24044	50	0021	jejuni	no	yes	yes
Campylobacter jejuni OXC4569	PubMLST	24046	21	0021	jejuni	no	yes	yes
Campylobacter jejuni OXC4576	PubMLST	24050	53	0021	jejuni	no	yes	yes
Campylobacter jejuni OXC4578	PubMLST	24052	50	0021	jejuni	no	yes	yes
Campylobacter jejuni OXC4581	PubMLST	24055	21	0021	jejuni	no	yes	yes
Campylobacter jejuni OXC4584	PubMLST	24057	861	0021	jejuni	no	yes	yes
Campylobacter jejuni OXC4590	PubMLST	24061	50	0021	jejuni	no	yes	yes
Campylobacter jejuni OXC4592	PubMLST	24062	53	0021	jejuni	no	yes	yes
Campylobacter jejuni OXC4600	PubMLST	24068	50	0021	jejuni	no	yes	yes
Campylobacter jejuni OXC4624	PubMLST	25655	21	0021	jejuni	no	yes	yes
Campylobacter jejuni OXC4628	PubMLST	24078	50	0021	jejuni	no	yes	yes
Campylobacter jejuni OXC4632	PubMLST	24082	53	0021	jejuni	no	yes	yes
Campylobacter jejuni OXC4633	PubMLST	24083	21	0021	jejuni	no	yes	yes
Campylobacter jejuni OXC4636	PubMLST	24086	53	0021	jejuni	no	yes	yes
Campylobacter jejuni OXC4649	PubMLST	24094	21	0021	jejuni	no	yes	yes
Campylobacter jejuni OXC4651	PubMLST	24095	520	0021	jejuni	no	yes	yes
Campylobacter jejuni OXC4663	PubMLST	24103	21	0021	jejuni	no	yes	yes
Campylobacter jejuni OXC4668	PubMLST	24107	53	0021	jejuni	no	yes	yes
Campylobacter jejuni OXC4673	PubMLST	24110	21	0021	jejuni	no	yes	yes
Campylobacter jejuni OXC4679	PubMLST	24115	883	0021	jejuni	no	yes	yes
Campylobacter jejuni OXC4726	PubMLST	18433	53	0021	jejuni	no	yes	yes
Campylobacter jejuni OXC4744	PubMLST	18446	2135	0021	jejuni	no	yes	yes
Campylobacter jejuni OXC4745	PubMLST	18447	917	0021	jejuni	no	yes	yes

Campylobacter jejuni OXC4749	PubMLST	18451	2135	0021	jejuni	no	yes	yes
Campylobacter jejuni OXC4754	PubMLST	18453	53	0021	jejuni	no	yes	yes
Campylobacter jejuni OXC4762	PubMLST	18456	2135	0021	jejuni	no	yes	yes
Campylobacter jejuni OXC4765	PubMLST	18458	2135	0021	jejuni	no	yes	yes
Campylobacter jejuni OXC4770	PubMLST	18462	2135	0021	jejuni	no	yes	yes
Campylobacter jejuni OXC4791	PubMLST	22134	2135	0021	jejuni	no	yes	yes
Campylobacter jejuni OXC4795	PubMLST	22137	2135	0021	jejuni	no	yes	yes
Campylobacter jejuni OXC4797	PubMLST	22138	50	0021	jejuni	no	yes	yes
Campylobacter jejuni OXC4807	PubMLST	22144	50	0021	jejuni	no	yes	yes
Campylobacter jejuni OXC4811	PubMLST	22148	21	0021	jejuni	no	yes	yes
Campylobacter jejuni OXC4831	PubMLST	22156	2135	0021	jejuni	no	yes	yes
Campylobacter jejuni OXC4836	PubMLST	22158	2135	0021	jejuni	no	yes	yes
Campylobacter jejuni OXC4863	PubMLST	22175	21	0021	jejuni	no	yes	yes
Campylobacter jejuni OXC4864	PubMLST	22176	19	0021	jejuni	no	yes	yes
Campylobacter jejuni OXC4877	PubMLST	22182	50	0021	jejuni	no	yes	yes
Campylobacter jejuni OXC4880	PubMLST	22185	50	0021	jejuni	no	yes	yes
Campylobacter jejuni OXC4882	PubMLST	22187	21	0021	jejuni	no	yes	yes
Campylobacter jejuni OXC4901	PubMLST	24462	2135	0021	jejuni	no	yes	yes
Campylobacter jejuni OXC4904	PubMLST	24464	2135	0021	jejuni	no	yes	yes
Campylobacter jejuni OXC4930	PubMLST	25093	50	0021	jejuni	no	yes	yes
Campylobacter jejuni OXC4932	PubMLST	24469	861	0021	jejuni	no	yes	yes
Campylobacter jejuni OXC4939	PubMLST	24471	520	0021	jejuni	no	yes	yes
Campylobacter jejuni OXC4961	PubMLST	25564	262	0021	jejuni	no	yes	yes
Campylobacter jejuni OXC4973	PubMLST	24664	53	0021	jejuni	no	yes	yes
Campylobacter jejuni OXC4990	PubMLST	24673	2135	0021	jejuni	no	yes	yes
Campylobacter jejuni OXC5002	PubMLST	24679	2135	0021	jejuni	no	yes	yes
Campylobacter jejuni OXC5004	PubMLST	24681	2135	0021	jejuni	no	yes	yes
Campylobacter jejuni OXC5010	PubMLST	24684	50	0021	jejuni	no	yes	yes
Campylobacter jejuni OXC5020	PubMLST	24690	1519	0021	jejuni	no	yes	yes
Campylobacter jejuni OXC5039	PubMLST	24705	2135	0021	jejuni	no	yes	yes
Campylobacter jejuni OXC5043	PubMLST	24707	19	0021	jejuni	no	yes	yes
Campylobacter jejuni OXC5049	PubMLST	24711	50	0021	jejuni	no	yes	yes
Campylobacter jejuni OXC5063	PubMLST	24719	21	0021	jejuni	no	yes	yes
Campylobacter jejuni OXC5067	PubMLST	24721	2135	0021	jejuni	no	yes	yes
Campylobacter jejuni OXC5081	PubMLST	24725	19	0021	jejuni	no	yes	yes
Campylobacter jejuni OXC5082	PubMLST	24726	21	0021	jejuni	no	yes	yes
Campylobacter jejuni OXC5102	PubMLST	24735	21	0021	jejuni	no	yes	yes
Campylobacter jejuni OXC5104	PubMLST	24736	53	0021	jejuni	no	yes	yes
Campylobacter jejuni OXC5110	PubMLST	24738	21	0021	jejuni	no	yes	yes
Campylobacter jejuni OXC5112	PubMLST	24740	21	0021	jejuni	no	yes	yes
Campylobacter jejuni OXC5117	PubMLST	24741	2135	0021	jejuni	no	yes	yes
Campylobacter jejuni OXC5118	PubMLST	24742	21	0021	jejuni	no	yes	yes
Campylobacter jejuni OXC5124	PubMLST	24746	21	0021	jejuni	no	yes	yes
Campylobacter jejuni OXC5125	PubMLST	24747	4526	0021	jejuni	no	yes	yes
Campylobacter jejuni OXC5126	PubMLST	24748	4526	0021	jejuni	no	yes	yes
Campylobacter jejuni OXC5131	PubMLST	24753	50	0021	jejuni	no	yes	yes
Campylobacter jejuni OXC5132	PubMLST	24754	883	0021	jejuni	no	yes	yes
Campylobacter jejuni OXC5133	PubMLST	24755	883	0021	jejuni	no	yes	yes
Campylobacter jejuni OXC5147	PubMLST	24762	21	0021	jejuni	no	yes	yes
Campylobacter jejuni OXC5149	PubMLST	24763	21	0021	jejuni	no	yes	yes
Campylobacter jejuni OXC5169	PubMLST	24770	50	0021	jejuni	no	yes	yes
Campylobacter jejuni OXC5184	PubMLST	24780	21	0021	jejuni	no	yes	yes
Campylobacter jejuni OXC5185	PubMLST	24781	21	0021	jejuni	no	yes	yes
Campylobacter jejuni OXC5189	PubMLST	24784	21	0021	jejuni	no	yes	yes
Campylobacter jejuni OXC5190	PubMLST	24785	883	0021	jejuni	no	yes	yes
Campylobacter jejuni OXC5200	PubMLST	24792	21	0021	jejuni	no	yes	yes
Campylobacter jejuni OXC5203	PubMLST	24793	21	0021	jejuni	no	yes	yes
Campylobacter jejuni OXC5218	PubMLST	24805	53	0021	jejuni	no	yes	yes
Campylobacter jejuni OXC5219	PubMLST	24806	53	0021	jejuni	no	yes	yes
Campylobacter jejuni OXC5220	PubMLST	24807	21	0021	jejuni	no	yes	yes
Campylobacter jejuni OXC5220_R	PubMLST	25561	21	0021	jejuni	no	yes	yes
Campylobacter jejuni OXC5221	PubMLST	24808	53	0021	jejuni	no	yes	yes
Campylobacter jejuni OXC5229	PubMLST	24815	2135	0021	jejuni	no	yes	yes
Campylobacter jejuni OXC5230	PubMLST	24816	19	0021	jejuni	no	yes	yes
Campylobacter jejuni OXC5239	PubMLST	24822	21	0021	jejuni	no	yes	yes
Campylobacter jejuni OXC5251	PubMLST	24830	190	0021	jejuni	no	yes	yes
Campylobacter jejuni OXC5260	PubMLST	24837	1519	0021	jejuni	no	yes	yes
Campylobacter jejuni OXC5298	PubMLST	24846	21	0021	jejuni	no	yes	yes
Campylobacter jejuni OXC5311	PubMLST	25566	53	0021	jejuni	no	yes	yes
Campylobacter jejuni OXC5335	PubMLST	21319	50	0021	jejuni	no	yes	yes
Campylobacter jejuni OXC5344	PubMLST	21326	21	0021	jejuni	no	yes	yes
Campylobacter jejuni OXC5349	PubMLST	21329	50	0021	jejuni	no	yes	yes
Campylobacter jejuni OXC5350	PubMLST	21330	21	0021	jejuni	no	yes	yes
Campylobacter jejuni OXC5364	PubMLST	21335	21	0021	jejuni	no	yes	yes
Campylobacter jejuni OXC5372	PubMLST	21339	19	0021	jejuni	no	yes	yes
Campylobacter jejuni OXC5378	PubMLST	21344	53	0021	jejuni	no	yes	yes
Campylobacter jejuni OXC5393	PubMLST	21353	50	0021	jejuni	no	yes	yes
Campylobacter jejuni OXC5414	PubMLST	21368	50	0021	jejuni	no	yes	yes
Campylobacter jejuni OXC5416	PubMLST	21369	3574	0021	jejuni	no	yes	yes
Campylobacter jejuni OXC5435	PubMLST	21381	50	0021	jejuni	no	yes	yes
Campylobacter jejuni OXC5437	PubMLST	21382	883	0021	jejuni	no	yes	yes
Campylobacter jejuni OXC5438	PubMLST	21383	53	0021	jejuni	no	yes	yes
Campylobacter jejuni OXC5444	PubMLST	21388	19	0021	jejuni	no	yes	yes
Campylobacter jejuni OXC5445	PubMLST	21389	50	0021	jejuni	no	yes	yes
Campylobacter jejuni OXC5451	PubMLST	21390	50	0021	jejuni	no	yes	yes
Campylobacter jejuni OXC5462	PubMLST	21398	50	0021	jejuni	no	yes	yes
Campylobacter jejuni OXC5463	PubMLST	21399	19	0021	jejuni	no	yes	yes
Campylobacter jejuni OXC5465	PubMLST	21401	21	0021	jejuni	no	yes	yes
Campylobacter jejuni OXC5470	PubMLST	21403	44	0021	jejuni	no	yes	yes
Campylobacter jejuni OXC5474	PubMLST	21406	21	0021	jejuni	no	yes	yes
Campylobacter jejuni OXC5476	PubMLST	21408	19	0021	jejuni	no	yes	yes
Campylobacter jejuni OXC5495	PubMLST	23497	21	0021	jejuni	no	yes	yes

Campylobacter jejuni OXC5498	PubMLST	23499	21	0021	jejuni	no	yes	yes
Campylobacter jejuni OXC5617	PubMLST	23507	53	0021	jejuni	no	yes	yes
Campylobacter jejuni OXC5618	PubMLST	23508	21	0021	jejuni	no	yes	yes
Campylobacter jejuni OXC5623	PubMLST	23511	7172	0021	jejuni	no	yes	yes
Campylobacter jejuni OXC5624	PubMLST	25103	982	0021	jejuni	no	yes	yes
Campylobacter jejuni OXC5625	PubMLST	23512	21	0021	jejuni	no	yes	yes
Campylobacter jejuni OXC5628	PubMLST	23514	50	0021	jejuni	no	yes	yes
Campylobacter jejuni OXC5639	PubMLST	23524	883	0021	jejuni	no	yes	yes
Campylobacter jejuni OXC5647	PubMLST	23531	19	0021	jejuni	no	yes	yes
Campylobacter jejuni OXC5649	PubMLST	23533	21	0021	jejuni	no	yes	yes
Campylobacter jejuni OXC5651	PubMLST	23534	50	0021	jejuni	no	yes	yes
Campylobacter jejuni OXC5652	PubMLST	23535	21	0021	jejuni	no	yes	yes
Campylobacter jejuni OXC5671	PubMLST	21418	883	0021	jejuni	no	yes	yes
Campylobacter jejuni OXC5691	PubMLST	21434	50	0021	jejuni	no	yes	yes
Campylobacter jejuni OXC5696	PubMLST	21437	53	0021	jejuni	no	yes	yes
Campylobacter jejuni OXC5708	PubMLST	21445	861	0021	jejuni	no	yes	yes
Campylobacter jejuni OXC5710	PubMLST	21447	21	0021	jejuni	no	yes	yes
Campylobacter jejuni OXC5720	PubMLST	21453	50	0021	jejuni	no	yes	yes
Campylobacter jejuni OXC5725	PubMLST	21457	50	0021	jejuni	no	yes	yes
Campylobacter jejuni OXC5728	PubMLST	21460	2355	0021	jejuni	no	yes	yes
Campylobacter jejuni OXC5731	PubMLST	21461	50	0021	jejuni	no	yes	yes
Campylobacter jejuni OXC5737	PubMLST	21465	266	0021	jejuni	no	yes	yes
Campylobacter jejuni OXC5739	PubMLST	21466	21	0021	jejuni	no	yes	yes
Campylobacter jejuni OXC5754	PubMLST	21478	21	0021	jejuni	no	yes	yes
Campylobacter jejuni OXC5757	PubMLST	21479	21	0021	jejuni	no	yes	yes
Campylobacter jejuni OXC5766	PubMLST	21487	50	0021	jejuni	no	yes	yes
Campylobacter jejuni OXC5767	PubMLST	21488	53	0021	jejuni	no	yes	yes
Campylobacter jejuni OXC5771	PubMLST	21491	50	0021	jejuni	no	yes	yes
Campylobacter jejuni OXC5772	PubMLST	21492	2135	0021	jejuni	no	yes	yes
Campylobacter jejuni OXC5774	PubMLST	21494	190	0021	jejuni	no	yes	yes
Campylobacter jejuni OXC5791	PubMLST	23542	21	0021	jejuni	no	yes	yes
Campylobacter jejuni OXC5799	PubMLST	23549	50	0021	jejuni	no	yes	yes
Campylobacter jejuni OXC5804	PubMLST	23660	53	0021	jejuni	no	yes	yes
Campylobacter jejuni OXC5808	PubMLST	23556	53	0021	jejuni	no	yes	yes
Campylobacter jejuni OXC5811	PubMLST	23559	2135	0021	jejuni	no	yes	yes
Campylobacter jejuni OXC5818	PubMLST	23566	19	0021	jejuni	no	yes	yes
Campylobacter jejuni OXC5819	PubMLST	23567	21	0021	jejuni	no	yes	yes
Campylobacter jejuni OXC5821	PubMLST	23569	2135	0021	jejuni	no	yes	yes
Campylobacter jejuni OXC5822	PubMLST	23570	21	0021	jejuni	no	yes	yes
Campylobacter jejuni OXC5823	PubMLST	23571	53	0021	jejuni	no	yes	yes
Campylobacter jejuni OXC5825	PubMLST	23662	53	0021	jejuni	no	yes	yes
Campylobacter jejuni OXC5826	PubMLST	23573	21	0021	jejuni	no	yes	yes
Campylobacter jejuni OXC5829	PubMLST	23575	50	0021	jejuni	no	yes	yes
Campylobacter jejuni OXC5832	PubMLST	23664	21	0021	jejuni	no	yes	yes
Campylobacter jejuni OXC5835	PubMLST	23579	21	0021	jejuni	no	yes	yes
Campylobacter jejuni OXC5840	PubMLST	23583	19	0021	jejuni	no	yes	yes
Campylobacter jejuni OXC5841	PubMLST	23584	2135	0021	jejuni	no	yes	yes
Campylobacter jejuni OXC5843	PubMLST	23586	861	0021	jejuni	no	yes	yes
Campylobacter jejuni OXC5846	PubMLST	23588	21	0021	jejuni	no	yes	yes
Campylobacter jejuni OXC5848	PubMLST	23590	21	0021	jejuni	no	yes	yes
Campylobacter jejuni OXC5862	PubMLST	23670	21	0021	jejuni	no	yes	yes
Campylobacter jejuni OXC5871	PubMLST	23671	19	0021	jejuni	no	yes	yes
Campylobacter jejuni OXC5879	PubMLST	23612	50	0021	jejuni	no	yes	yes
Campylobacter jejuni OXC5880	PubMLST	23613	53	0021	jejuni	no	yes	yes
Campylobacter jejuni OXC5887	PubMLST	23618	19	0021	jejuni	no	yes	yes
Campylobacter jejuni OXC5888	PubMLST	23619	2135	0021	jejuni	no	yes	yes
Campylobacter jejuni OXC5895	PubMLST	23625	2135	0021	jejuni	no	yes	yes
Campylobacter jejuni OXC5899	PubMLST	23676	19	0021	jejuni	no	yes	yes
Campylobacter jejuni OXC5902	PubMLST	23677	50	0021	jejuni	no	yes	yes
Campylobacter jejuni OXC5905	PubMLST	23631	50	0021	jejuni	no	yes	yes
Campylobacter jejuni OXC5906	PubMLST	23632	50	0021	jejuni	no	yes	yes
Campylobacter jejuni OXC5909	PubMLST	23635	53	0021	jejuni	no	yes	yes
Campylobacter jejuni OXC5918	PubMLST	23643	53	0021	jejuni	no	yes	yes
Campylobacter jejuni OXC5924	PubMLST	23679	21	0021	jejuni	no	yes	yes
Campylobacter jejuni OXC5925	PubMLST	23647	50	0021	jejuni	no	yes	yes
Campylobacter jejuni OXC5926	PubMLST	23648	50	0021	jejuni	no	yes	yes
Campylobacter jejuni OXC5929	PubMLST	23680	21	0021	jejuni	no	yes	yes
Campylobacter jejuni OXC5937	PubMLST	23681	21	0021	jejuni	no	yes	yes
Campylobacter jejuni OXC5938	PubMLST	23656	21	0021	jejuni	no	yes	yes
Campylobacter jejuni OXC6251	PubMLST	16049	21	0021	jejuni	no	yes	yes
Campylobacter jejuni OXC6255	PubMLST	16336	21	0021	jejuni	no	yes	yes
Campylobacter jejuni OXC6257	PubMLST	16054	21	0021	jejuni	no	yes	yes
Campylobacter jejuni OXC6266	PubMLST	16063	50	0021	jejuni	no	yes	yes
Campylobacter jejuni OXC6270	PubMLST	16067	21	0021	jejuni	no	yes	yes
Campylobacter jejuni OXC6275	PubMLST	16072	21	0021	jejuni	no	yes	yes
Campylobacter jejuni OXC6277	PubMLST	16074	50	0021	jejuni	no	yes	yes
Campylobacter jejuni OXC6282	PubMLST	16079	53	0021	jejuni	no	yes	yes
Campylobacter jejuni OXC6285	PubMLST	16082	21	0021	jejuni	no	yes	yes
Campylobacter jejuni OXC6286	PubMLST	16083	50	0021	jejuni	no	yes	yes
Campylobacter jejuni OXC6289	PubMLST	16086	50	0021	jejuni	no	yes	yes
Campylobacter jejuni OXC6292	PubMLST	16089	50	0021	jejuni	no	yes	yes
Campylobacter jejuni OXC6300	PubMLST	16097	2135	0021	jejuni	no	yes	yes
Campylobacter jejuni OXC6301	PubMLST	16098	861	0021	jejuni	no	yes	yes
Campylobacter jejuni OXC6303	PubMLST	16100	19	0021	jejuni	no	yes	yes
Campylobacter jejuni OXC6305	PubMLST	16102	861	0021	jejuni	no	yes	yes
Campylobacter jejuni OXC6310	PubMLST	16107	21	0021	jejuni	no	yes	yes
Campylobacter jejuni OXC6317	PubMLST	16114	21	0021	jejuni	no	yes	yes
Campylobacter jejuni OXC6324	PubMLST	16121	53	0021	jejuni	no	yes	yes
Campylobacter jejuni OXC6325	PubMLST	16122	19	0021	jejuni	no	yes	yes
Campylobacter jejuni OXC6326	PubMLST	16123	50	0021	jejuni	no	yes	yes
Campylobacter jejuni OXC6329	PubMLST	16126	21	0021	jejuni	no	yes	yes
Campylobacter jejuni OXC6331	PubMLST	16128	50	0021	jejuni	no	yes	yes

Campylobacter jejuni OXC6334	PubMLST	16131	21	0021	jejuni	no	yes	yes
Campylobacter jejuni OXC6335	PubMLST	16132	21	0021	jejuni	no	yes	yes
Campylobacter jejuni OXC6347	PubMLST	16144	50	0021	jejuni	no	yes	yes
Campylobacter jejuni OXC6367	PubMLST	16164	53	0021	jejuni	no	yes	yes
Campylobacter jejuni OXC6370	PubMLST	16167	19	0021	jejuni	no	yes	yes
Campylobacter jejuni OXC6379	PubMLST	16176	5726	0021	jejuni	no	yes	yes
Campylobacter jejuni OXC6383	PubMLST	16180	21	0021	jejuni	no	yes	yes
Campylobacter jejuni OXC6384	PubMLST	16181	19	0021	jejuni	no	yes	yes
Campylobacter jejuni OXC6393	PubMLST	16190	5727	0021	jejuni	no	yes	yes
Campylobacter jejuni OXC6394	PubMLST	16191	19	0021	jejuni	no	yes	yes
Campylobacter jejuni OXC6405	PubMLST	16200	21	0021	jejuni	no	yes	yes
Campylobacter jejuni OXC6420	PubMLST	16215	21	0021	jejuni	no	yes	yes
Campylobacter jejuni OXC6449	PubMLST	16242	50	0021	jejuni	no	yes	yes
Campylobacter jejuni OXC6457	PubMLST	16249	50	0021	jejuni	no	yes	yes
Campylobacter jejuni OXC6459	PubMLST	16251	50	0021	jejuni	no	yes	yes
Campylobacter jejuni OXC6461	PubMLST	16253	50	0021	jejuni	no	yes	yes
Campylobacter jejuni OXC6479	PubMLST	16271	883	0021	jejuni	no	yes	yes
Campylobacter jejuni OXC6483	PubMLST	16275	21	0021	jejuni	no	yes	yes
Campylobacter jejuni OXC6489	PubMLST	16281	50	0021	jejuni	no	yes	yes
Campylobacter jejuni OXC6493	PubMLST	16285	50	0021	jejuni	no	yes	yes
Campylobacter jejuni OXC6496	PubMLST	16288	21	0021	jejuni	no	yes	yes
Campylobacter jejuni OXC6500	PubMLST	16292	2135	0021	jejuni	no	yes	yes
Campylobacter jejuni OXC6502	PubMLST	16294	50	0021	jejuni	no	yes	yes
Campylobacter jejuni OXC6508	PubMLST	16300	21	0021	jejuni	no	yes	yes
Campylobacter jejuni OXC6514	PubMLST	16306	3102	0021	jejuni	no	yes	yes
Campylobacter jejuni OXC6516	PubMLST	16308	53	0021	jejuni	no	yes	yes
Campylobacter jejuni OXC6519	PubMLST	16311	21	0021	jejuni	no	yes	yes
Campylobacter jejuni OXC6524	PubMLST	16316	50	0021	jejuni	no	yes	yes
Campylobacter jejuni OXC6527	PubMLST	16319	50	0021	jejuni	no	yes	yes
Campylobacter jejuni OXC6530	PubMLST	16322	50	0021	jejuni	no	yes	yes
Campylobacter jejuni OXC6531	PubMLST	16323	50	0021	jejuni	no	yes	yes
Campylobacter jejuni OXC6538	PubMLST	16330	19	0021	jejuni	no	yes	yes
Campylobacter jejuni OXC6539	PubMLST	16331	21	0021	jejuni	no	yes	yes
Campylobacter jejuni OXC6543	PubMLST	16335	50	0021	jejuni	no	yes	yes
Campylobacter jejuni OXC6548	PubMLST	12883	19	0021	jejuni	no	yes	yes
Campylobacter jejuni OXC6552	PubMLST	12887	3769	0021	jejuni	no	yes	yes
Campylobacter jejuni OXC6558	PubMLST	12893	53	0021	jejuni	no	yes	yes
Campylobacter jejuni OXC6563	PubMLST	12898	21	0021	jejuni	no	yes	yes
Campylobacter jejuni OXC6564	PubMLST	12899	21	0021	jejuni	no	yes	yes
Campylobacter jejuni OXC6565	PubMLST	12900	50	0021	jejuni	no	yes	yes
Campylobacter jejuni OXC6571	PubMLST	12906	50	0021	jejuni	no	yes	yes
Campylobacter jejuni OXC6573	PubMLST	12908	21	0021	jejuni	no	yes	yes
Campylobacter jejuni OXC6590	PubMLST	16341	50	0021	jejuni	no	yes	yes
Campylobacter jejuni OXC6596	PubMLST	16347	21	0021	jejuni	no	yes	yes
Campylobacter jejuni OXC6598	PubMLST	16349	50	0021	jejuni	no	yes	yes
Campylobacter jejuni OXC6600	PubMLST	16351	50	0021	jejuni	no	yes	yes
Campylobacter jejuni OXC6602	PubMLST	16353	21	0021	jejuni	no	yes	yes
Campylobacter jejuni OXC6603	PubMLST	16354	19	0021	jejuni	no	yes	yes
Campylobacter jejuni OXC6604	PubMLST	16355	21	0021	jejuni	no	yes	yes
Campylobacter jejuni OXC6605	PubMLST	22905	21	0021	jejuni	no	yes	yes
Campylobacter jejuni OXC6613	PubMLST	16363	50	0021	jejuni	no	yes	yes
Campylobacter jejuni OXC6615	PubMLST	16365	50	0021	jejuni	no	yes	yes
Campylobacter jejuni OXC6616	PubMLST	16366	21	0021	jejuni	no	yes	yes
Campylobacter jejuni OXC6619	PubMLST	16368	21	0021	jejuni	no	yes	yes
Campylobacter jejuni OXC6625	PubMLST	16374	21	0021	jejuni	no	yes	yes
Campylobacter jejuni OXC6627	PubMLST	16376	21	0021	jejuni	no	yes	yes
Campylobacter jejuni OXC6629	PubMLST	16378	21	0021	jejuni	no	yes	yes
Campylobacter jejuni OXC6636	PubMLST	16385	50	0021	jejuni	no	yes	yes
Campylobacter jejuni OXC6637	PubMLST	16386	21	0021	jejuni	no	yes	yes
Campylobacter jejuni OXC6642	PubMLST	18199	21	0021	jejuni	no	yes	yes
Campylobacter jejuni OXC6643	PubMLST	18200	53	0021	jejuni	no	yes	yes
Campylobacter jejuni OXC6656	PubMLST	18213	53	0021	jejuni	no	yes	yes
Campylobacter jejuni OXC6664	PubMLST	18221	50	0021	jejuni	no	yes	yes
Campylobacter jejuni OXC6665	PubMLST	18222	50	0021	jejuni	no	yes	yes
Campylobacter jejuni OXC6666	PubMLST	18223	50	0021	jejuni	no	yes	yes
Campylobacter jejuni OXC6667	PubMLST	18224	44	0021	jejuni	no	yes	yes
Campylobacter jejuni OXC6671	PubMLST	18228	21	0021	jejuni	no	yes	yes
Campylobacter jejuni OXC6673	PubMLST	18230	50	0021	jejuni	no	yes	yes
Campylobacter jejuni OXC6677	PubMLST	18234	50	0021	jejuni	no	yes	yes
Campylobacter jejuni OXC6678	PubMLST	18235	21	0021	jejuni	no	yes	yes
Campylobacter jejuni OXC6681	PubMLST	18238	21	0021	jejuni	no	yes	yes
Campylobacter jejuni OXC6682	PubMLST	18239	53	0021	jejuni	no	yes	yes
Campylobacter jejuni OXC6689	PubMLST	18246	21	0021	jejuni	no	yes	yes
Campylobacter jejuni OXC6690	PubMLST	18247	21	0021	jejuni	no	yes	yes
Campylobacter jejuni OXC6691	PubMLST	18248	190	0021	jejuni	no	yes	yes
Campylobacter jejuni OXC6702	PubMLST	18259	21	0021	jejuni	no	yes	yes
Campylobacter jejuni OXC6703	PubMLST	18260	19	0021	jejuni	no	yes	yes
Campylobacter jejuni OXC6706	PubMLST	18263	19	0021	jejuni	no	yes	yes
Campylobacter jejuni OXC6711	PubMLST	18268	21	0021	jejuni	no	yes	yes
Campylobacter jejuni OXC6712	PubMLST	18269	21	0021	jejuni	no	yes	yes
Campylobacter jejuni OXC6713	PubMLST	18270	50	0021	jejuni	no	yes	yes
Campylobacter jejuni OXC6714	PubMLST	18271	50	0021	jejuni	no	yes	yes
Campylobacter jejuni OXC6727	PubMLST	18284	5811	0021	jejuni	no	yes	yes
Campylobacter jejuni OXC6728	PubMLST	18285	21	0021	jejuni	no	yes	yes
Campylobacter jejuni OXC6730	PubMLST	18287	21	0021	jejuni	no	yes	yes
Campylobacter jejuni OXC6731	PubMLST	18288	21	0021	jejuni	no	yes	yes
Campylobacter jejuni OXC6734	PubMLST	18291	21	0021	jejuni	no	yes	yes
Campylobacter jejuni OXC6741	PubMLST	18298	53	0021	jejuni	no	yes	yes
Campylobacter jejuni OXC6745	PubMLST	18302	21	0021	jejuni	no	yes	yes
Campylobacter jejuni OXC6752	PubMLST	18309	2135	0021	jejuni	no	yes	yes
Campylobacter jejuni OXC6763	PubMLST	18320	50	0021	jejuni	no	yes	yes
Campylobacter jejuni OXC6764	PubMLST	18321	21	0021	jejuni	no	yes	yes

Campylobacter jejuni OXC6766	PubMLST	18323	262	0021	jejuni	no	yes	yes
Campylobacter jejuni OXC6779	PubMLST	18336	53	0021	jejuni	no	yes	yes
Campylobacter jejuni OXC6781	PubMLST	18338	2135	0021	jejuni	no	yes	yes
Campylobacter jejuni OXC6782	PubMLST	18339	2135	0021	jejuni	no	yes	yes
Campylobacter jejuni OXC6786	PubMLST	18343	2135	0021	jejuni	no	yes	yes
Campylobacter jejuni OXC6789	PubMLST	18346	53	0021	jejuni	no	yes	yes
Campylobacter jejuni OXC6791	PubMLST	18348	21	0021	jejuni	no	yes	yes
Campylobacter jejuni OXC6798	PubMLST	18355	4526	0021	jejuni	no	yes	yes
Campylobacter jejuni OXC6804	PubMLST	18361	21	0021	jejuni	no	yes	yes
Campylobacter jejuni OXC6812	PubMLST	18369	21	0021	jejuni	no	yes	yes
Campylobacter jejuni OXC6815	PubMLST	18372	21	0021	jejuni	no	yes	yes
Campylobacter jejuni OXC6820	PubMLST	18377	50	0021	jejuni	no	yes	yes
Campylobacter jejuni OXC6823	PubMLST	18380	883	0021	jejuni	no	yes	yes
Campylobacter jejuni OXC6824	PubMLST	18381	262	0021	jejuni	no	yes	yes
Campylobacter jejuni OXC6833	PubMLST	18389	104	0021	jejuni	no	yes	yes
Campylobacter jejuni OXC6836	PubMLST	21555	50	0021	jejuni	no	yes	yes
Campylobacter jejuni OXC6845	PubMLST	21564	21	0021	jejuni	no	yes	yes
Campylobacter jejuni OXC6850	PubMLST	21569	21	0021	jejuni	no	yes	yes
Campylobacter jejuni OXC6852	PubMLST	21571	262	0021	jejuni	no	yes	yes
Campylobacter jejuni OXC6854	PubMLST	21573	53	0021	jejuni	no	yes	yes
Campylobacter jejuni OXC6856	PubMLST	21575	53	0021	jejuni	no	yes	yes
Campylobacter jejuni OXC6857	PubMLST	21576	520	0021	jejuni	no	yes	yes
Campylobacter jejuni OXC6860	PubMLST	21579	50	0021	jejuni	no	yes	yes
Campylobacter jejuni OXC6861	PubMLST	21580	50	0021	jejuni	no	yes	yes
Campylobacter jejuni OXC6862	PubMLST	21581	53	0021	jejuni	no	yes	yes
Campylobacter jejuni OXC6865	PubMLST	21584	50	0021	jejuni	no	yes	yes
Campylobacter jejuni OXC6867	PubMLST	21586	21	0021	jejuni	no	yes	yes
Campylobacter jejuni OXC6868	PubMLST	21587	50	0021	jejuni	no	yes	yes
Campylobacter jejuni OXC6872	PubMLST	21591	21	0021	jejuni	no	yes	yes
Campylobacter jejuni OXC6876	PubMLST	21595	53	0021	jejuni	no	yes	yes
Campylobacter jejuni OXC6879	PubMLST	21598	21	0021	jejuni	no	yes	yes
Campylobacter jejuni OXC6886	PubMLST	22082	21	0021	jejuni	no	yes	yes
Campylobacter jejuni OXC6892	PubMLST	22087	21	0021	jejuni	no	yes	yes
Campylobacter jejuni OXC6900	PubMLST	22095	50	0021	jejuni	no	yes	yes
Campylobacter jejuni OXC6909	PubMLST	22104	21	0021	jejuni	no	yes	yes
Campylobacter jejuni OXC6922	PubMLST	22117	50	0021	jejuni	no	yes	yes
Campylobacter jejuni OXC6924	PubMLST	22119	883	0021	jejuni	no	yes	yes
Campylobacter jejuni OXC6927	PubMLST	22122	53	0021	jejuni	no	yes	yes
Campylobacter jejuni OXC6932	PubMLST	21106	50	0021	jejuni	no	yes	yes
Campylobacter jejuni OXC6941	PubMLST	21115	53	0021	jejuni	no	yes	yes
Campylobacter jejuni OXC6945	PubMLST	21119	21	0021	jejuni	no	yes	yes
Campylobacter jejuni OXC6946	PubMLST	21120	50	0021	jejuni	no	yes	yes
Campylobacter jejuni OXC6948	PubMLST	21122	21	0021	jejuni	no	yes	yes
Campylobacter jejuni OXC6949	PubMLST	21123	50	0021	jejuni	no	yes	yes
Campylobacter jejuni OXC6953	PubMLST	21127	21	0021	jejuni	no	yes	yes
Campylobacter jejuni OXC6956	PubMLST	21130	50	0021	jejuni	no	yes	yes
Campylobacter jejuni OXC6959	PubMLST	21133	5727	0021	jejuni	no	yes	yes
Campylobacter jejuni OXC6961	PubMLST	21135	21	0021	jejuni	no	yes	yes
Campylobacter jejuni OXC6964	PubMLST	21138	50	0021	jejuni	no	yes	yes
Campylobacter jejuni OXC6979	PubMLST	21152	21	0021	jejuni	no	yes	yes
Campylobacter jejuni OXC6981	PubMLST	21154	44	0021	jejuni	no	yes	yes
Campylobacter jejuni OXC6985	PubMLST	21158	6135	0021	jejuni	no	yes	yes
Campylobacter jejuni OXC6988	PubMLST	21161	5726	0021	jejuni	no	yes	yes
Campylobacter jejuni OXC6994	PubMLST	21165	50	0021	jejuni	no	yes	yes
Campylobacter jejuni OXC6998	PubMLST	21169	21	0021	jejuni	no	yes	yes
Campylobacter jejuni OXC7000	PubMLST	21171	21	0021	jejuni	no	yes	yes
Campylobacter jejuni OXC7002	PubMLST	22189	21	0021	jejuni	no	yes	yes
Campylobacter jejuni OXC7015	PubMLST	22202	50	0021	jejuni	no	yes	yes
Campylobacter jejuni OXC7020	PubMLST	22207	50	0021	jejuni	no	yes	yes
Campylobacter jejuni OXC7029	PubMLST	22215	50	0021	jejuni	no	yes	yes
Campylobacter jejuni OXC7031	PubMLST	22217	883	0021	jejuni	no	yes	yes
Campylobacter jejuni OXC7035	PubMLST	22221	883	0021	jejuni	no	yes	yes
Campylobacter jejuni OXC7037	PubMLST	22223	21	0021	jejuni	no	yes	yes
Campylobacter jejuni OXC7041	PubMLST	22226	50	0021	jejuni	no	yes	yes
Campylobacter jejuni OXC7046	PubMLST	22231	2135	0021	jejuni	no	yes	yes
Campylobacter jejuni OXC7049	PubMLST	22234	266	0021	jejuni	no	yes	yes
Campylobacter jejuni OXC7058	PubMLST	22243	21	0021	jejuni	no	yes	yes
Campylobacter jejuni OXC7065	PubMLST	22249	50	0021	jejuni	no	yes	yes
Campylobacter jejuni OXC7068	PubMLST	22252	19	0021	jejuni	no	yes	yes
Campylobacter jejuni OXC7071	PubMLST	22255	50	0021	jejuni	no	yes	yes
Campylobacter jejuni OXC7073	PubMLST	22257	21	0021	jejuni	no	yes	yes
Campylobacter jejuni OXC7078	PubMLST	22262	1949	0021	jejuni	no	yes	yes
Campylobacter jejuni OXC7080	PubMLST	22264	50	0021	jejuni	no	yes	yes
Campylobacter jejuni OXC7084	PubMLST	22268	21	0021	jejuni	no	yes	yes
Campylobacter jejuni OXC7089	PubMLST	22660	53	0021	jejuni	no	yes	yes
Campylobacter jejuni OXC7091	PubMLST	22273	262	0021	jejuni	no	yes	yes
Campylobacter jejuni OXC7104	PubMLST	21186	6137	0021	jejuni	no	yes	yes
Campylobacter jejuni OXC7105	PubMLST	21187	6137	0021	jejuni	no	yes	yes
Campylobacter jejuni OXC7118	PubMLST	21200	266	0021	jejuni	no	yes	yes
Campylobacter jejuni OXC7132	PubMLST	21213	50	0021	jejuni	no	yes	yes
Campylobacter jejuni OXC7134	PubMLST	21215	21	0021	jejuni	no	yes	yes
Campylobacter jejuni OXC7136	PubMLST	21217	21	0021	jejuni	no	yes	yes
Campylobacter jejuni OXC7137	PubMLST	21218	21	0021	jejuni	no	yes	yes
Campylobacter jejuni OXC7141	PubMLST	21222	44	0021	jejuni	no	yes	yes
Campylobacter jejuni OXC7142	PubMLST	21223	21	0021	jejuni	no	yes	yes
Campylobacter jejuni OXC7147	PubMLST	21228	21	0021	jejuni	no	yes	yes
Campylobacter jejuni OXC7148	PubMLST	21229	822	0021	jejuni	no	yes	yes
Campylobacter jejuni OXC7150	PubMLST	21231	44	0021	jejuni	no	yes	yes
Campylobacter jejuni OXC7153	PubMLST	21234	21	0021	jejuni	no	yes	yes
Campylobacter jejuni OXC7160	PubMLST	21241	19	0021	jejuni	no	yes	yes
Campylobacter jejuni OXC7163	PubMLST	21244	19	0021	jejuni	no	yes	yes
Campylobacter jejuni OXC7168	PubMLST	21249	19	0021	jejuni	no	yes	yes

Campylobacter jejuni OXC7173	PubMLST	22664	44	0021	jejuni	no	yes	yes
Campylobacter jejuni OXC7187	PubMLST	22678	21	0021	jejuni	no	yes	yes
Campylobacter jejuni OXC7188	PubMLST	22679	50	0021	jejuni	no	yes	yes
Campylobacter jejuni OXC7190	PubMLST	22681	21	0021	jejuni	no	yes	yes
Campylobacter jejuni OXC7195	PubMLST	22686	21	0021	jejuni	no	yes	yes
Campylobacter jejuni OXC7211	PubMLST	22702	19	0021	jejuni	no	yes	yes
Campylobacter jejuni OXC7212	PubMLST	22703	21	0021	jejuni	no	yes	yes
Campylobacter jejuni OXC7214	PubMLST	22705	50	0021	jejuni	no	yes	yes
Campylobacter jejuni OXC7220	PubMLST	22711	50	0021	jejuni	no	yes	yes
Campylobacter jejuni OXC7223	PubMLST	22714	21	0021	jejuni	no	yes	yes
Campylobacter jejuni OXC7230	PubMLST	22720	53	0021	jejuni	no	yes	yes
Campylobacter jejuni OXC7236	PubMLST	22726	53	0021	jejuni	no	yes	yes
Campylobacter jejuni OXC7246	PubMLST	22288	50	0021	jejuni	no	yes	yes
Campylobacter jejuni OXC7247	PubMLST	22289	21	0021	jejuni	no	yes	yes
Campylobacter jejuni OXC7253	PubMLST	22295	19	0021	jejuni	no	yes	yes
Campylobacter jejuni OXC7256	PubMLST	22297	19	0021	jejuni	no	yes	yes
Campylobacter jejuni OXC7260	PubMLST	22301	50	0021	jejuni	no	yes	yes
Campylobacter jejuni OXC7264	PubMLST	22305	50	0021	jejuni	no	yes	yes
Campylobacter jejuni OXC7270	PubMLST	22311	50	0021	jejuni	no	yes	yes
Campylobacter jejuni OXC7285	PubMLST	22326	21	0021	jejuni	no	yes	yes
Campylobacter jejuni OXC7293	PubMLST	22334	50	0021	jejuni	no	yes	yes
Campylobacter jejuni OXC7302	PubMLST	22343	50	0021	jejuni	no	yes	yes
Campylobacter jejuni OXC7305	PubMLST	22346	50	0021	jejuni	no	yes	yes
Campylobacter jejuni OXC7306	PubMLST	22347	21	0021	jejuni	no	yes	yes
Campylobacter jejuni OXC7320	PubMLST	22359	19	0021	jejuni	no	yes	yes
Campylobacter jejuni OXC7329	PubMLST	22367	19	0021	jejuni	no	yes	yes
Campylobacter jejuni OXC7330	PubMLST	22368	21	0021	jejuni	no	yes	yes
Campylobacter jejuni OXC7335	PubMLST	22373	21	0021	jejuni	no	yes	yes
Campylobacter jejuni OXC7336	PubMLST	22374	104	0021	jejuni	no	yes	yes
Campylobacter jejuni OXC7348	PubMLST	23888	19	0021	jejuni	no	yes	yes
Campylobacter jejuni OXC7349	PubMLST	23889	50	0021	jejuni	no	yes	yes
Campylobacter jejuni OXC7351	PubMLST	23891	21	0021	jejuni	no	yes	yes
Campylobacter jejuni OXC7356	PubMLST	23895	883	0021	jejuni	no	yes	yes
Campylobacter jejuni OXC7362	PubMLST	23901	50	0021	jejuni	no	yes	yes
Campylobacter jejuni OXC7365	PubMLST	23904	262	0021	jejuni	no	yes	yes
Campylobacter jejuni OXC7366	PubMLST	23905	21	0021	jejuni	no	yes	yes
Campylobacter jejuni OXC7370	PubMLST	23908	53	0021	jejuni	no	yes	yes
Campylobacter jejuni OXC7372	PubMLST	23910	21	0021	jejuni	no	yes	yes
Campylobacter jejuni OXC7373	PubMLST	23911	21	0021	jejuni	no	yes	yes
Campylobacter jejuni OXC7384	PubMLST	23922	104	0021	jejuni	no	yes	yes
Campylobacter jejuni OXC7387	PubMLST	23925	21	0021	jejuni	no	yes	yes
Campylobacter jejuni OXC7397	PubMLST	23934	21	0021	jejuni	no	yes	yes
Campylobacter jejuni OXC7398	PubMLST	23935	53	0021	jejuni	no	yes	yes
Campylobacter jejuni OXC7405	PubMLST	23941	21	0021	jejuni	no	yes	yes
Campylobacter jejuni OXC7412	PubMLST	23947	21	0021	jejuni	no	yes	yes
Campylobacter jejuni OXC7415	PubMLST	23949	21	0021	jejuni	no	yes	yes
Campylobacter jejuni OXC7417	PubMLST	23951	50	0021	jejuni	no	yes	yes
Campylobacter jejuni OXC7419	PubMLST	23953	21	0021	jejuni	no	yes	yes
Campylobacter jejuni OXC7423	PubMLST	23957	21	0021	jejuni	no	yes	yes
Campylobacter jejuni OXC7427	PubMLST	23960	104	0021	jejuni	no	yes	yes
Campylobacter jejuni OXC7428	PubMLST	23961	50	0021	jejuni	no	yes	yes
Campylobacter jejuni OXC7431	PubMLST	23964	3102	0021	jejuni	no	yes	yes
Campylobacter jejuni OXC7434	PubMLST	25621	21	0021	jejuni	no	yes	yes
Campylobacter jejuni OXC7440	PubMLST	23971	21	0021	jejuni	no	yes	yes
Campylobacter jejuni OXC7460	PubMLST	25623	21	0021	jejuni	no	yes	yes
Campylobacter jejuni OXC7464	PubMLST	23993	21	0021	jejuni	no	yes	yes
Campylobacter jejuni OXC7468	PubMLST	23997	53	0021	jejuni	no	yes	yes
Campylobacter jejuni OXC7470	PubMLST	23999	53	0021	jejuni	no	yes	yes
Campylobacter jejuni OXC7477	PubMLST	24006	50	0021	jejuni	no	yes	yes
Campylobacter jejuni OXC7479	PubMLST	24008	21	0021	jejuni	no	yes	yes
Campylobacter jejuni OXC7481	PubMLST	24010	50	0021	jejuni	no	yes	yes
Campylobacter jejuni OXC7488	PubMLST	24016	50	0021	jejuni	no	yes	yes
Campylobacter jejuni OXC7489	PubMLST	24017	21	0021	jejuni	no	yes	yes
Campylobacter jejuni OXC7499	PubMLST	24026	50	0021	jejuni	no	yes	yes
Campylobacter jejuni OXC7504	PubMLST	24030	982	0021	jejuni	no	yes	yes
Campylobacter jejuni OXC7512	PubMLST	24037	50	0021	jejuni	no	yes	yes
Campylobacter jejuni OXC7514	PubMLST	24119	50	0021	jejuni	no	yes	yes
Campylobacter jejuni OXC7526	PubMLST	24130	53	0021	jejuni	no	yes	yes
Campylobacter jejuni OXC7529	PubMLST	24133	50	0021	jejuni	no	yes	yes
Campylobacter jejuni OXC7530	PubMLST	24134	21	0021	jejuni	no	yes	yes
Campylobacter jejuni OXC7535	PubMLST	24478	50	0021	jejuni	no	yes	yes
Campylobacter jejuni OXC7547	PubMLST	24486	3574	0021	jejuni	no	yes	yes
Campylobacter jejuni OXC7558	PubMLST	24495	21	0021	jejuni	no	yes	yes
Campylobacter jejuni OXC7561	PubMLST	24498	3853	0021	jejuni	no	yes	yes
Campylobacter jejuni OXC7573	PubMLST	24510	53	0021	jejuni	no	yes	yes
Campylobacter jejuni OXC7577	PubMLST	24514	53	0021	jejuni	no	yes	yes
Campylobacter jejuni OXC7595	PubMLST	24527	104	0021	jejuni	no	yes	yes
Campylobacter jejuni OXC7602	PubMLST	25108	21	0021	jejuni	no	yes	yes
Campylobacter jejuni OXC7603	PubMLST	24534	53	0021	jejuni	no	yes	yes
Campylobacter jejuni OXC7606	PubMLST	24536	50	0021	jejuni	no	yes	yes
Campylobacter jejuni OXC7608	PubMLST	24538	21	0021	jejuni	no	yes	yes
Campylobacter jejuni OXC7610	PubMLST	25109	21	0021	jejuni	no	yes	yes
Campylobacter jejuni OXC7613	PubMLST	24542	50	0021	jejuni	no	yes	yes
Campylobacter jejuni OXC7614	PubMLST	24543	50	0021	jejuni	no	yes	yes
Campylobacter jejuni OXC7624	PubMLST	24553	21	0021	jejuni	no	yes	yes
Campylobacter jejuni OXC7627	PubMLST	24556	50	0021	jejuni	no	yes	yes
Campylobacter jejuni OXC7631	PubMLST	24560	21	0021	jejuni	no	yes	yes
Campylobacter jejuni OXC7637	PubMLST	24566	50	0021	jejuni	no	yes	yes
Campylobacter jejuni OXC7639	PubMLST	24568	50	0021	jejuni	no	yes	yes
Campylobacter jejuni OXC7641	PubMLST	24898	50	0021	jejuni	no	yes	yes
Campylobacter jejuni OXC7645	PubMLST	24902	50	0021	jejuni	no	yes	yes
Campylobacter jejuni OXC7651	PubMLST	25555	21	0021	jejuni	no	yes	yes

Campylobacter jejuni OXC7656	PubMLST	24911	3853	0021	jejuni	no	yes	yes
Campylobacter jejuni OXC7657	PubMLST	24912	50	0021	jejuni	no	yes	yes
Campylobacter jejuni OXC7660	PubMLST	24915	50	0021	jejuni	no	yes	yes
Campylobacter jejuni OXC7667	PubMLST	24922	21	0021	jejuni	no	yes	yes
Campylobacter jejuni OXC7669	PubMLST	24924	50	0021	jejuni	no	yes	yes
Campylobacter jejuni OXC7670	PubMLST	24925	50	0021	jejuni	no	yes	yes
Campylobacter jejuni OXC7672	PubMLST	24926	21	0021	jejuni	no	yes	yes
Campylobacter jejuni OXC7677	PubMLST	24931	3853	0021	jejuni	no	yes	yes
Campylobacter jejuni OXC7686	PubMLST	24937	21	0021	jejuni	no	yes	yes
Campylobacter jejuni OXC7688	PubMLST	25627	21	0021	jejuni	no	yes	yes
Campylobacter jejuni OXC7691	PubMLST	25628	21	0021	jejuni	no	yes	yes
Campylobacter jejuni OXC7693	PubMLST	24941	21	0021	jejuni	no	yes	yes
Campylobacter jejuni OXC7695	PubMLST	24943	50	0021	jejuni	no	yes	yes
Campylobacter jejuni OXC7699	PubMLST	24947	50	0021	jejuni	no	yes	yes
Campylobacter jejuni OXC7700	PubMLST	24948	21	0021	jejuni	no	yes	yes
Campylobacter jejuni OXC7714	PubMLST	27866	50	0021	jejuni	no	yes	yes
Campylobacter jejuni OXC7716	PubMLST	24958	50	0021	jejuni	no	yes	yes
Campylobacter jejuni OXC7718	PubMLST	24960	21	0021	jejuni	no	yes	yes
Campylobacter jejuni OXC7720	PubMLST	24962	50	0021	jejuni	no	yes	yes
Campylobacter jejuni OXC7726	PubMLST	24967	19	0021	jejuni	no	yes	yes
Campylobacter jejuni OXC7729	PubMLST	25630	21	0021	jejuni	no	yes	yes
Campylobacter jejuni OXC7731	PubMLST	24970	3853	0021	jejuni	no	yes	yes
Campylobacter jejuni OXC7740	PubMLST	27868	50	0021	jejuni	no	yes	yes
Campylobacter jejuni OXC7746	PubMLST	24981	50	0021	jejuni	no	yes	yes
Campylobacter jejuni OXC7748	PubMLST	24983	19	0021	jejuni	no	yes	yes
Campylobacter jejuni OXC7753	PubMLST	27869	50	0021	jejuni	no	yes	yes
Campylobacter jejuni OXC7758	PubMLST	25110	21	0021	jejuni	no	yes	yes
Campylobacter jejuni OXC7764	PubMLST	24573	21	0021	jejuni	no	yes	yes
Campylobacter jejuni OXC7795	PubMLST	28973	21	0021	jejuni	no	yes	yes
Campylobacter jejuni OXC7801	PubMLST	25111	21	0021	jejuni	no	yes	yes
Campylobacter jejuni OXC7804	PubMLST	27884	50	0021	jejuni	no	yes	yes
Campylobacter jejuni OXC7805	PubMLST	25112	50	0021	jejuni	no	yes	yes
Campylobacter jejuni OXC7810	PubMLST	24608	50	0021	jejuni	no	yes	yes
Campylobacter jejuni OXC7835	PubMLST	24632	50	0021	jejuni	no	yes	yes
Campylobacter jejuni OXC7836	PubMLST	24633	50	0021	jejuni	no	yes	yes
Campylobacter jejuni OXC7837	PubMLST	24634	50	0021	jejuni	no	yes	yes
Campylobacter jejuni OXC7841	PubMLST	24638	883	0021	jejuni	no	yes	yes
Campylobacter jejuni OXC7845	PubMLST	24642	50	0021	jejuni	no	yes	yes
Campylobacter jejuni OXC7854	PubMLST	24989	50	0021	jejuni	no	yes	yes
Campylobacter jejuni OXC7856	PubMLST	24991	21	0021	jejuni	no	yes	yes
Campylobacter jejuni OXC7866	PubMLST	25001	50	0021	jejuni	no	yes	yes
Campylobacter jejuni OXC7867	PubMLST	25002	50	0021	jejuni	no	yes	yes
Campylobacter jejuni OXC7871	PubMLST	25006	50	0021	jejuni	no	yes	yes
Campylobacter jejuni OXC7887	PubMLST	25018	19	0021	jejuni	no	yes	yes
Campylobacter jejuni OXC7892	PubMLST	25023	6891	0021	jejuni	no	yes	yes
Campylobacter jejuni OXC7896	PubMLST	25027	6892	0021	jejuni	no	yes	yes
Campylobacter jejuni OXC7905	PubMLST	25036	21	0021	jejuni	no	yes	yes
Campylobacter jejuni OXC7910	PubMLST	25040	53	0021	jejuni	no	yes	yes
Campylobacter jejuni OXC7915	PubMLST	25045	21	0021	jejuni	no	yes	yes
Campylobacter jejuni OXC7919	PubMLST	25049	21	0021	jejuni	no	yes	yes
Campylobacter jejuni OXC7924	PubMLST	25053	21	0021	jejuni	no	yes	yes
Campylobacter jejuni OXC7926	PubMLST	25055	19	0021	jejuni	no	yes	yes
Campylobacter jejuni OXC7927	PubMLST	25056	21	0021	jejuni	no	yes	yes
Campylobacter jejuni OXC7929	PubMLST	25058	19	0021	jejuni	no	yes	yes
Campylobacter jejuni OXC7930	PubMLST	25117	50	0021	jejuni	no	yes	yes
Campylobacter jejuni OXC7949	PubMLST	25569	50	0021	jejuni	no	yes	yes
Campylobacter jejuni OXC7954	PubMLST	25374	21	0021	jejuni	no	yes	yes
Campylobacter jejuni OXC7957	PubMLST	25376	21	0021	jejuni	no	yes	yes
Campylobacter jejuni OXC7958	PubMLST	25478	50	0021	jejuni	no	yes	yes
Campylobacter jejuni OXC7961	PubMLST	25479	50	0021	jejuni	no	yes	yes
Campylobacter jejuni OXC7966	PubMLST	25381	50	0021	jejuni	no	yes	yes
Campylobacter jejuni OXC7969	PubMLST	25384	21	0021	jejuni	no	yes	yes
Campylobacter jejuni OXC7973	PubMLST	25388	44	0021	jejuni	no	yes	yes
Campylobacter jejuni OXC7976	PubMLST	25391	21	0021	jejuni	no	yes	yes
Campylobacter jejuni OXC7981	PubMLST	25395	6892	0021	jejuni	no	yes	yes
Campylobacter jejuni OXC7990	PubMLST	25482	21	0021	jejuni	no	yes	yes
Campylobacter jejuni OXC7997	PubMLST	25404	2782	0021	jejuni	no	yes	yes
Campylobacter jejuni OXC8004	PubMLST	25488	5727	0021	jejuni	no	yes	yes
Campylobacter jejuni OXC8015	PubMLST	25585	50	0021	jejuni	no	yes	yes
Campylobacter jejuni OXC8019	PubMLST	25494	21	0021	jejuni	no	yes	yes
Campylobacter jejuni OXC8022	PubMLST	25409	50	0021	jejuni	no	yes	yes
Campylobacter jejuni OXC8025	PubMLST	25496	21	0021	jejuni	no	yes	yes
Campylobacter jejuni OXC8033	PubMLST	25502	21	0021	jejuni	no	yes	yes
Campylobacter jejuni OXC8038	PubMLST	25504	883	0021	jejuni	no	yes	yes
Campylobacter jejuni OXC8045R	PubMLST	28558	19	0021	jejuni	no	yes	yes
Campylobacter jejuni OXC8063	PubMLST	25513	50	0021	jejuni	no	yes	yes
Campylobacter jejuni OXC8077	PubMLST	25517	21	0021	jejuni	no	yes	yes
Campylobacter jejuni OXC8082	PubMLST	25520	50	0021	jejuni	no	yes	yes
Campylobacter jejuni OXC8085	PubMLST	25424	262	0021	jejuni	no	yes	yes
Campylobacter jejuni OXC8099	PubMLST	25527	50	0021	jejuni	no	yes	yes
Campylobacter jejuni OXC8103	PubMLST	25530	50	0021	jejuni	no	yes	yes
Campylobacter jejuni OXC8106	PubMLST	25430	53	0021	jejuni	no	yes	yes
Campylobacter jejuni OXC8107	PubMLST	25532	53	0021	jejuni	no	yes	yes
Campylobacter jejuni OXC8111	PubMLST	25616	21	0021	jejuni	no	yes	yes
Campylobacter jejuni OXC8115	PubMLST	25431	21	0021	jejuni	no	yes	yes
Campylobacter jejuni OXC8116	PubMLST	25432	21	0021	jejuni	no	yes	yes
Campylobacter jejuni OXC8129	PubMLST	25460	21	0021	jejuni	no	yes	yes
Campylobacter jejuni OXC8132	PubMLST	25462	883	0021	jejuni	no	yes	yes
Campylobacter jejuni OXC8133	PubMLST	25560	883	0021	jejuni	no	yes	yes
Campylobacter jejuni OXC8138	PubMLST	25464	50	0021	jejuni	no	yes	yes
Campylobacter jejuni OXC8139	PubMLST	27888	262	0021	jejuni	no	yes	yes
Campylobacter jejuni OXC8145	PubMLST	25435	21	0021	jejuni	no	yes	yes

Campylobacter jejuni OXC8162	PubMLST	25438	21	0021	jejuni	no	yes	yes
Campylobacter jejuni OXC8163	PubMLST	25543	21	0021	jejuni	no	yes	yes
Campylobacter jejuni OXC8171	PubMLST	25441	53	0021	jejuni	no	yes	yes
Campylobacter jejuni OXC8183	PubMLST	25645	50	0021	jejuni	no	yes	yes
Campylobacter jejuni OXC8184	PubMLST	27889	266	0021	jejuni	no	yes	yes
Campylobacter jejuni OXC8186	PubMLST	27891	21	0021	jejuni	no	yes	yes
Campylobacter jejuni OXC8190	PubMLST	27895	19	0021	jejuni	no	yes	yes
Campylobacter jejuni OXC8192	PubMLST	27897	50	0021	jejuni	no	yes	yes
Campylobacter jejuni OXC8197	PubMLST	27902	50	0021	jejuni	no	yes	yes
Campylobacter jejuni OXC8198	PubMLST	27903	6983	0021	jejuni	no	yes	yes
Campylobacter jejuni OXC8200	PubMLST	27905	21	0021	jejuni	no	yes	yes
Campylobacter jejuni OXC8202	PubMLST	27907	21	0021	jejuni	no	yes	yes
Campylobacter jejuni OXC8227	PubMLST	27929	50	0021	jejuni	no	yes	yes
Campylobacter jejuni OXC8230	PubMLST	27932	50	0021	jejuni	no	yes	yes
Campylobacter jejuni OXC8231	PubMLST	27933	50	0021	jejuni	no	yes	yes
Campylobacter jejuni OXC8233	PubMLST	27935	21	0021	jejuni	no	yes	yes
Campylobacter jejuni OXC8234	PubMLST	27936	21	0021	jejuni	no	yes	yes
Campylobacter jejuni OXC8241	PubMLST	27941	53	0021	jejuni	no	yes	yes
Campylobacter jejuni OXC8245	PubMLST	28561	21	0021	jejuni	no	yes	yes
Campylobacter jejuni OXC8254	PubMLST	27952	50	0021	jejuni	no	yes	yes
Campylobacter jejuni OXC8257	PubMLST	27955	50	0021	jejuni	no	yes	yes
Campylobacter jejuni OXC8262	PubMLST	27958	21	0021	jejuni	no	yes	yes
Campylobacter jejuni OXC8264	PubMLST	27960	50	0021	jejuni	no	yes	yes
Campylobacter jejuni OXC8269	PubMLST	27963	19	0021	jejuni	no	yes	yes
Campylobacter jejuni OXC8270	PubMLST	28562	19	0021	jejuni	no	yes	yes
Campylobacter jejuni OXC8278	PubMLST	27971	50	0021	jejuni	no	yes	yes
Campylobacter jejuni OXC8279	PubMLST	27972	21	0021	jejuni	no	yes	yes
Campylobacter jejuni OXC8282	PubMLST	27974	50	0021	jejuni	no	yes	yes
Campylobacter jejuni OXC8291	PubMLST	27982	50	0021	jejuni	no	yes	yes
Campylobacter jejuni OXC8296	PubMLST	27987	50	0021	jejuni	no	yes	yes
Campylobacter jejuni OXC8301	PubMLST	27992	50	0021	jejuni	no	yes	yes
Campylobacter jejuni OXC8302	PubMLST	27993	50	0021	jejuni	no	yes	yes
Campylobacter jejuni OXC8312	PubMLST	28003	21	0021	jejuni	no	yes	yes
Campylobacter jejuni OXC8318R	PubMLST	28974	50	0021	jejuni	no	yes	yes
Campylobacter jejuni OXC8320	PubMLST	28011	50	0021	jejuni	no	yes	yes
Campylobacter jejuni OXC8325	PubMLST	28016	50	0021	jejuni	no	yes	yes
Campylobacter jejuni OXC8326	PubMLST	28017	44	0021	jejuni	no	yes	yes
Campylobacter jejuni OXC8327	PubMLST	28018	53	0021	jejuni	no	yes	yes
Campylobacter jejuni OXC8330	PubMLST	28021	53	0021	jejuni	no	yes	yes
Campylobacter jejuni OXC8331	PubMLST	28022	50	0021	jejuni	no	yes	yes
Campylobacter jejuni OXC8335	PubMLST	28026	50	0021	jejuni	no	yes	yes
Campylobacter jejuni OXC8337	PubMLST	28028	53	0021	jejuni	no	yes	yes
Campylobacter jejuni OXC8342	PubMLST	28033	50	0021	jejuni	no	yes	yes
Campylobacter jejuni OXC8346	PubMLST	28037	19	0021	jejuni	no	yes	yes
Campylobacter jejuni OXC8350	PubMLST	28040	21	0021	jejuni	no	yes	yes
Campylobacter jejuni OXC8352	PubMLST	28042	53	0021	jejuni	no	yes	yes
Campylobacter jejuni OXC8359	PubMLST	28047	50	0021	jejuni	no	yes	yes
Campylobacter jejuni OXC8364	PubMLST	28567	50	0021	jejuni	no	yes	yes
Campylobacter jejuni OXC8367	PubMLST	28054	50	0021	jejuni	no	yes	yes
Campylobacter jejuni OXC8368	PubMLST	28569	50	0021	jejuni	no	yes	yes
Campylobacter jejuni OXC8379	PubMLST	28572	50	0021	jejuni	no	yes	yes
Campylobacter jejuni OXC8381	PubMLST	28574	50	0021	jejuni	no	yes	yes
Campylobacter jejuni OXC8384	PubMLST	28577	21	0021	jejuni	no	yes	yes
Campylobacter jejuni OXC8394	PubMLST	28587	53	0021	jejuni	no	yes	yes
Campylobacter jejuni OXC8396	PubMLST	28589	53	0021	jejuni	no	yes	yes
Campylobacter jejuni OXC8398	PubMLST	28590	21	0021	jejuni	no	yes	yes
Campylobacter jejuni OXC8400	PubMLST	28592	21	0021	jejuni	no	yes	yes
Campylobacter jejuni OXC8405	PubMLST	28981	50	0021	jejuni	no	yes	yes
Campylobacter jejuni OXC8405R	PubMLST	29067	50	0021	jejuni	no	yes	yes
Campylobacter jejuni OXC8407	PubMLST	28598	53	0021	jejuni	no	yes	yes
Campylobacter jejuni OXC8411	PubMLST	28602	53	0021	jejuni	no	yes	yes
Campylobacter jejuni OXC8414	PubMLST	28605	53	0021	jejuni	no	yes	yes
Campylobacter jejuni OXC8418	PubMLST	28609	21	0021	jejuni	no	yes	yes
Campylobacter jejuni OXC8419	PubMLST	28610	21	0021	jejuni	no	yes	yes
Campylobacter jejuni OXC8421	PubMLST	28612	21	0021	jejuni	no	yes	yes
Campylobacter jejuni OXC8424	PubMLST	28615	53	0021	jejuni	no	yes	yes
Campylobacter jejuni OXC8427	PubMLST	28618	50	0021	jejuni	no	yes	yes
Campylobacter jejuni OXC8428	PubMLST	28619	861	0021	jejuni	no	yes	yes
Campylobacter jejuni OXC8436	PubMLST	28627	19	0021	jejuni	no	yes	yes
Campylobacter jejuni OXC8438	PubMLST	28629	2135	0021	jejuni	no	yes	yes
Campylobacter jejuni OXC8443	PubMLST	28634	5649	0021	jejuni	no	yes	yes
Campylobacter jejuni OXC8449	PubMLST	28640	53	0021	jejuni	no	yes	yes
Campylobacter jejuni OXC8455	PubMLST	28646	50	0021	jejuni	no	yes	yes
Campylobacter jejuni OXC8456	PubMLST	28647	50	0021	jejuni	no	yes	yes
Campylobacter jejuni OXC8457	PubMLST	28648	50	0021	jejuni	no	yes	yes
Campylobacter jejuni OXC8462	PubMLST	28654	50	0021	jejuni	no	yes	yes
Campylobacter jejuni OXC8472	PubMLST	28665	50	0021	jejuni	no	yes	yes
Campylobacter jejuni OXC8476	PubMLST	28669	262	0021	jejuni	no	yes	yes
Campylobacter jejuni OXC8478	PubMLST	28671	50	0021	jejuni	no	yes	yes
Campylobacter jejuni OXC8481	PubMLST	28674	53	0021	jejuni	no	yes	yes
Campylobacter jejuni OXC8489	PubMLST	28681	53	0021	jejuni	no	yes	yes
Campylobacter jejuni OXC8500	PubMLST	28691	50	0021	jejuni	no	yes	yes
Campylobacter jejuni OXC8501	PubMLST	28692	50	0021	jejuni	no	yes	yes
Campylobacter jejuni OXC8502	PubMLST	28693	50	0021	jejuni	no	yes	yes
Campylobacter jejuni OXC8505	PubMLST	28696	53	0021	jejuni	no	yes	yes
Campylobacter jejuni OXC8511	PubMLST	28702	19	0021	jejuni	no	yes	yes
Campylobacter jejuni OXC8514	PubMLST	28705	50	0021	jejuni	no	yes	yes
Campylobacter jejuni OXC8520	PubMLST	28711	21	0021	jejuni	no	yes	yes
Campylobacter jejuni OXC8528	PubMLST	28719	50	0021	jejuni	no	yes	yes
Campylobacter jejuni OXC8539	PubMLST	28729	50	0021	jejuni	no	yes	yes
Campylobacter jejuni OXC8541	PubMLST	28731	50	0021	jejuni	no	yes	yes
Campylobacter jejuni OXC8547	PubMLST	28736	262	0021	jejuni	no	yes	yes

Campylobacter jejuni OXC8549	PubMLST	28738	21	0021	jejuni	no	yes	yes
Campylobacter jejuni OXC8551	PubMLST	28740	21	0021	jejuni	no	yes	yes
Campylobacter jejuni OXC8559	PubMLST	28900	53	0021	jejuni	no	yes	yes
Campylobacter jejuni OXC8563	PubMLST	28977	19	0021	jejuni	no	yes	yes
Campylobacter jejuni OXC8564	PubMLST	28903	50	0021	jejuni	no	yes	yes
Campylobacter jejuni OXC8569	PubMLST	28908	21	0021	jejuni	no	yes	yes
Campylobacter jejuni OXC8571	PubMLST	28978	21	0021	jejuni	no	yes	yes
Campylobacter jejuni OXC8582	PubMLST	28975	19	0021	jejuni	no	yes	yes
Campylobacter jejuni OXC8584	PubMLST	28920	53	0021	jejuni	no	yes	yes
Campylobacter jejuni OXC8585	PubMLST	28921	7307	0021	jejuni	no	yes	yes
Campylobacter jejuni OXC8586	PubMLST	28922	50	0021	jejuni	no	yes	yes
Campylobacter jejuni OXC8591	PubMLST	28980	21	0021	jejuni	no	yes	yes
Campylobacter jejuni OXC8599	PubMLST	28931	53	0021	jejuni	no	yes	yes
Campylobacter jejuni OXC8605	PubMLST	28984	53	0021	jejuni	no	yes	yes
Campylobacter jejuni OXC8606	PubMLST	28985	53	0021	jejuni	no	yes	yes
Campylobacter jejuni OXC8609	PubMLST	28939	53	0021	jejuni	no	yes	yes
Campylobacter jejuni OXC8614	PubMLST	28942	50	0021	jejuni	no	yes	yes
Campylobacter jejuni OXC8620	PubMLST	28989	50	0021	jejuni	no	yes	yes
Campylobacter jejuni OXC8621	PubMLST	29072	21	0021	jejuni	no	yes	yes
Campylobacter jejuni OXC8623	PubMLST	29086	21	0021	jejuni	no	yes	yes
Campylobacter jejuni OXC8646	PubMLST	28960	50	0021	jejuni	no	yes	yes
Campylobacter jejuni OXC8658	PubMLST	28972	50	0021	jejuni	no	yes	yes
Campylobacter jejuni OXC8665	PubMLST	29000	50	0021	jejuni	no	yes	yes
Campylobacter jejuni OXC8666	PubMLST	29076	19	0021	jejuni	no	yes	yes
Campylobacter jejuni OXC8673	PubMLST	28976	21	0021	jejuni	no	yes	yes
Campylobacter jejuni OXC8674	PubMLST	29006	47	0021	jejuni	no	yes	yes
Campylobacter jejuni OXC8676	PubMLST	29008	883	0021	jejuni	no	yes	yes
Campylobacter jejuni OXC8678	PubMLST	29010	21	0021	jejuni	no	yes	yes
Campylobacter jejuni OXC8684	PubMLST	29016	19	0021	jejuni	no	yes	yes
Campylobacter jejuni OXC8685	PubMLST	29017	19	0021	jejuni	no	yes	yes
Campylobacter jejuni OXC8691	PubMLST	29023	883	0021	jejuni	no	yes	yes
Campylobacter jejuni OXC8693	PubMLST	29025	1519	0021	jejuni	no	yes	yes
Campylobacter jejuni OXC8694	PubMLST	29026	47	0021	jejuni	no	yes	yes
Campylobacter jejuni OXC8699	PubMLST	28896	50	0021	jejuni	no	yes	yes
Campylobacter jejuni OXC8704	PubMLST	29033	21	0021	jejuni	no	yes	yes
Campylobacter jejuni OXC8713	PubMLST	29042	21	0021	jejuni	no	yes	yes
Campylobacter jejuni OXC8714	PubMLST	29043	21	0021	jejuni	no	yes	yes
Campylobacter jejuni OXC8715	PubMLST	29044	21	0021	jejuni	no	yes	yes
Campylobacter jejuni OXC8718	PubMLST	29080	21	0021	jejuni	no	yes	yes
Campylobacter jejuni OXC8727	PubMLST	29052	50	0021	jejuni	no	yes	yes
Campylobacter jejuni OXC8729	PubMLST	29054	883	0021	jejuni	no	yes	yes
Campylobacter jejuni OXC8734	PubMLST	29059	21	0021	jejuni	no	yes	yes
Campylobacter jejuni OXC8738	PubMLST	29063	883	0021	jejuni	no	yes	yes
Campylobacter jejuni OXC8743	PubMLST	30434	50	0021	jejuni	no	yes	yes
Campylobacter jejuni OXC8749	PubMLST	30440	50	0021	jejuni	no	yes	yes
Campylobacter jejuni OXC8756	PubMLST	30446	50	0021	jejuni	no	yes	yes
Campylobacter jejuni OXC8762	PubMLST	30452	21	0021	jejuni	no	yes	yes
Campylobacter jejuni OXC8768	PubMLST	30457	19	0021	jejuni	no	yes	yes
Campylobacter jejuni OXC8775	PubMLST	30464	19	0021	jejuni	no	yes	yes
Campylobacter jejuni OXC8778	PubMLST	30467	21	0021	jejuni	no	yes	yes
Campylobacter jejuni OXC8779	PubMLST	30468	21	0021	jejuni	no	yes	yes
Campylobacter jejuni OXC8783	PubMLST	30472	50	0021	jejuni	no	yes	yes
Campylobacter jejuni OXC8785	PubMLST	30474	50	0021	jejuni	no	yes	yes
Campylobacter jejuni OXC8794	PubMLST	30483	19	0021	jejuni	no	yes	yes
Campylobacter jejuni OXC8816	PubMLST	30504	21	0021	jejuni	no	yes	yes
Campylobacter jejuni OXC8831	PubMLST	30519	50	0021	jejuni	no	yes	yes
Campylobacter jejuni OXC8836	PubMLST	30524	19	0021	jejuni	no	yes	yes
Campylobacter jejuni OXC8837	PubMLST	30425	19	0021	jejuni	no	yes	yes
Campylobacter jejuni OXC8850	PubMLST	30536	21	0021	jejuni	no	yes	yes
Campylobacter jejuni OXC8852	PubMLST	30538	53	0021	jejuni	no	yes	yes
Campylobacter jejuni OXC8854	PubMLST	30540	50	0021	jejuni	no	yes	yes
Campylobacter jejuni OXC8859	PubMLST	30545	53	0021	jejuni	no	yes	yes
Campylobacter jejuni OXC8862	PubMLST	30548	19	0021	jejuni	no	yes	yes
Campylobacter jejuni OXC8863	PubMLST	30549	7380	0021	jejuni	no	yes	yes
Campylobacter jejuni OXC8867	PubMLST	30553	50	0021	jejuni	no	yes	yes
Campylobacter jejuni OXC8873	PubMLST	30559	21	0021	jejuni	no	yes	yes
Campylobacter jejuni OXC8874	PubMLST	30560	3574	0021	jejuni	no	yes	yes
Campylobacter jejuni OXC8878	PubMLST	30564	883	0021	jejuni	no	yes	yes
Campylobacter jejuni OXC8879	PubMLST	30565	883	0021	jejuni	no	yes	yes
Campylobacter jejuni OXC8880	PubMLST	30566	883	0021	jejuni	no	yes	yes
Campylobacter jejuni OXC8884	PubMLST	30424	50	0021	jejuni	no	yes	yes
Campylobacter jejuni OXC8885	PubMLST	30569	50	0021	jejuni	no	yes	yes
Campylobacter jejuni OXC8899	PubMLST	30582	50	0021	jejuni	no	yes	yes
Campylobacter jejuni OXC8901	PubMLST	30584	50	0021	jejuni	no	yes	yes
Campylobacter jejuni OXC8905	PubMLST	30588	21	0021	jejuni	no	yes	yes
Campylobacter jejuni OXC8916	PubMLST	30599	53	0021	jejuni	no	yes	yes
Campylobacter jejuni OXC8922	PubMLST	30605	50	0021	jejuni	no	yes	yes
Campylobacter jejuni OXC8929	PubMLST	30612	53	0021	jejuni	no	yes	yes
Campylobacter jejuni OXC8939	PubMLST	30622	21	0021	jejuni	no	yes	yes
Campylobacter jejuni OXC8951	PubMLST	30634	53	0021	jejuni	no	yes	yes
Campylobacter jejuni OXC8966	PubMLST	30648	50	0021	jejuni	no	yes	yes
Campylobacter jejuni OXC8969	PubMLST	30651	21	0021	jejuni	no	yes	yes
Campylobacter jejuni OXC8980	PubMLST	30662	50	0021	jejuni	no	yes	yes
Campylobacter jejuni OXC8984	PubMLST	30666	50	0021	jejuni	no	yes	yes
Campylobacter jejuni OXC8992	PubMLST	30674	50	0021	jejuni	no	yes	yes
Campylobacter jejuni OXC8999	PubMLST	30680	53	0021	jejuni	no	yes	yes
Campylobacter jejuni OXC9004	PubMLST	30423	50	0021	jejuni	no	yes	yes
Campylobacter jejuni OXC9006	PubMLST	30686	19	0021	jejuni	no	yes	yes
Campylobacter jejuni PT14	Genbank/EMBL/DDBJ	CP003871	50	0021	jejuni	no	yes	yes
Campylobacter jejuni RB922	Genbank/EMBL/DDBJ	CAFS01	21	0021	jejuni	no	yes	yes
Campylobacter jejuni V124000-1	PubMLST	28877	21	0021	jejuni	no	yes	yes
Campylobacter jejuni XY259	Genbank/EMBL/DDBJ	CAFR01	21	0021	jejuni	no	yes	yes

Campylobacter jejuni BID1MW	PubMLST	25122	22	0022	jejuni	no	yes	yes
Campylobacter jejuni Dg253	PubMLST	25980	22	0022	jejuni	no	yes	yes
Campylobacter jejuni Dg258	PubMLST	26044	22	0022	jejuni	no	yes	yes
Campylobacter jejuni Dg65a	PubMLST	25935	22	0022	jejuni	no	yes	yes
Campylobacter jejuni HB93-13	Genbank/EMBL/DDBJ	AANQ01	22	0022	jejuni	no	yes	yes
Campylobacter jejuni OXC4566	PubMLST	24043	22	0022	jejuni	no	yes	yes
Campylobacter jejuni OXC4986	PubMLST	24669	22	0022	jejuni	no	yes	yes
Campylobacter jejuni OXC5048A	PubMLST	24709	22	0022	jejuni	no	yes	yes
Campylobacter jejuni OXC5048B	PubMLST	24710	22	0022	jejuni	no	yes	yes
Campylobacter jejuni OXC5212	PubMLST	25470	22	0022	jejuni	no	yes	yes
Campylobacter jejuni OXC5323	PubMLST	25102	22	0022	jejuni	no	yes	yes
Campylobacter jejuni OXC5383	PubMLST	21347	22	0022	jejuni	no	yes	yes
Campylobacter jejuni OXC5411	PubMLST	21365	22	0022	jejuni	no	yes	yes
Campylobacter jejuni OXC5443	PubMLST	21387	22	0022	jejuni	no	yes	yes
Campylobacter jejuni OXC5933	PubMLST	23654	22	0022	jejuni	no	yes	yes
Campylobacter jejuni OXC6264	PubMLST	16061	22	0022	jejuni	no	yes	yes
Campylobacter jejuni OXC6406	PubMLST	16201	1947	0022	jejuni	no	yes	yes
Campylobacter jejuni OXC6414	PubMLST	16209	22	0022	jejuni	no	yes	yes
Campylobacter jejuni OXC6535	PubMLST	16327	22	0022	jejuni	no	yes	yes
Campylobacter jejuni OXC6626	PubMLST	16375	22	0022	jejuni	no	yes	yes
Campylobacter jejuni OXC6641	PubMLST	16390	22	0022	jejuni	no	yes	yes
Campylobacter jejuni OXC6644	PubMLST	18201	22	0022	jejuni	no	yes	yes
Campylobacter jejuni OXC6645	PubMLST	18202	22	0022	jejuni	no	yes	yes
Campylobacter jejuni OXC6708	PubMLST	18265	22	0022	jejuni	no	yes	yes
Campylobacter jejuni OXC6755	PubMLST	18312	22	0022	jejuni	no	yes	yes
Campylobacter jejuni OXC6796	PubMLST	18353	22	0022	jejuni	no	yes	yes
Campylobacter jejuni OXC6866	PubMLST	21585	22	0022	jejuni	no	yes	yes
Campylobacter jejuni OXC6887	PubMLST	22083	22	0022	jejuni	no	yes	yes
Campylobacter jejuni OXC6888	PubMLST	22084	22	0022	jejuni	no	yes	yes
Campylobacter jejuni OXC6899	PubMLST	22094	22	0022	jejuni	no	yes	yes
Campylobacter jejuni OXC6902	PubMLST	22097	22	0022	jejuni	no	yes	yes
Campylobacter jejuni OXC6929	PubMLST	22124	22	0022	jejuni	no	yes	yes
Campylobacter jejuni OXC6966	PubMLST	21140	22	0022	jejuni	no	yes	yes
Campylobacter jejuni OXC7017	PubMLST	22204	22	0022	jejuni	no	yes	yes
Campylobacter jejuni OXC7085	PubMLST	22269	22	0022	jejuni	no	yes	yes
Campylobacter jejuni OXC7158	PubMLST	21239	22	0022	jejuni	no	yes	yes
Campylobacter jejuni OXC7178	PubMLST	22669	22	0022	jejuni	no	yes	yes
Campylobacter jejuni OXC7364	PubMLST	23903	22	0022	jejuni	no	yes	yes
Campylobacter jejuni OXC7367	PubMLST	23906	22	0022	jejuni	no	yes	yes
Campylobacter jejuni OXC7375	PubMLST	23913	22	0022	jejuni	no	yes	yes
Campylobacter jejuni OXC7463	PubMLST	23992	22	0022	jejuni	no	yes	yes
Campylobacter jejuni OXC7490	PubMLST	24018	22	0022	jejuni	no	yes	yes
Campylobacter jejuni OXC7626	PubMLST	24555	22	0022	jejuni	no	yes	yes
Campylobacter jejuni OXC7666	PubMLST	24921	22	0022	jejuni	no	yes	yes
Campylobacter jejuni OXC7697	PubMLST	24945	22	0022	jejuni	no	yes	yes
Campylobacter jejuni OXC7809	PubMLST	24607	22	0022	jejuni	no	yes	yes
Campylobacter jejuni OXC7818	PubMLST	24615	22	0022	jejuni	no	yes	yes
Campylobacter jejuni OXC7838	PubMLST	24635	22	0022	jejuni	no	yes	yes
Campylobacter jejuni OXC7921	PubMLST	25116	660	0022	jejuni	no	yes	yes
Campylobacter jejuni OXC7942	PubMLST	25568	22	0022	jejuni	no	yes	yes
Campylobacter jejuni OXC7953	PubMLST	25572	22	0022	jejuni	no	yes	yes
Campylobacter jejuni OXC8031	PubMLST	25500	22	0022	jejuni	no	yes	yes
Campylobacter jejuni OXC8043	PubMLST	25593	22	0022	jejuni	no	yes	yes
Campylobacter jejuni OXC8169	PubMLST	25546	22	0022	jejuni	no	yes	yes
Campylobacter jejuni OXC8305	PubMLST	27996	22	0022	jejuni	no	yes	yes
Campylobacter jejuni OXC8348	PubMLST	28038	22	0022	jejuni	no	yes	yes
Campylobacter jejuni OXC8453	PubMLST	28644	22	0022	jejuni	no	yes	yes
Campylobacter jejuni OXC8771	PubMLST	30460	22	0022	jejuni	no	yes	yes
Campylobacter jejuni OXC8800	PubMLST	30489	22	0022	jejuni	no	yes	yes
Campylobacter jejuni OXC8868	PubMLST	30554	22	0022	jejuni	no	yes	yes
Campylobacter jejuni OXC8987	PubMLST	30669	22	0022	jejuni	no	yes	yes
Campylobacter jejuni OXC8994	PubMLST	30675	22	0022	jejuni	no	yes	yes
Campylobacter jejuni 129-258	Genbank/EMBL/DDBJ	AINY01	459	0042	jejuni	no	yes	yes
Campylobacter jejuni OXC7092	PubMLST	22274	447	0042	jejuni	no	yes	yes
Campylobacter jejuni OXC7879	PubMLST	25013	447	0042	jejuni	no	yes	yes
Campylobacter jejuni OXC8579	PubMLST	28916	447	0042	jejuni	no	yes	yes
Campylobacter jejuni 4031	Genbank/EMBL/DDBJ	HG428754	45	0045	jejuni	no	yes	yes
Campylobacter jejuni C8	PubMLST	24249	45	0045	jejuni	no	yes	yes
Campylobacter jejuni Dg104	PubMLST	26043	7259	0045	jejuni	no	yes	yes
Campylobacter jejuni Dg109	PubMLST	26019	7259	0045	jejuni	no	yes	yes
Campylobacter jejuni Dg117	PubMLST	26064	7259	0045	jejuni	no	yes	yes
Campylobacter jejuni Dg122	PubMLST	25999	4791	0045	jejuni	no	yes	yes
Campylobacter jejuni Dg128	PubMLST	26007	7259	0045	jejuni	no	yes	yes
Campylobacter jejuni Dg130	PubMLST	26015	7259	0045	jejuni	no	yes	yes
Campylobacter jejuni Dg131	PubMLST	25988	7259	0045	jejuni	no	yes	yes
Campylobacter jejuni Dg137	PubMLST	26031	7259	0045	jejuni	no	yes	yes
Campylobacter jejuni Dg145	PubMLST	24193	45	0045	jejuni	no	yes	yes
Campylobacter jejuni Dg161	PubMLST	26016	45	0045	jejuni	no	yes	yes
Campylobacter jejuni Dg162	PubMLST	26024	45	0045	jejuni	no	yes	yes
Campylobacter jejuni Dg180	PubMLST	26034	4791	0045	jejuni	no	yes	yes
Campylobacter jejuni Dg184	PubMLST	26040	4791	0045	jejuni	no	yes	yes
Campylobacter jejuni Dg187	PubMLST	26054	4791	0045	jejuni	no	yes	yes
Campylobacter jejuni Dg200	PubMLST	25985	45	0045	jejuni	no	yes	yes
Campylobacter jejuni Dg202	PubMLST	26026	45	0045	jejuni	no	yes	yes
Campylobacter jejuni Dg206	PubMLST	26018	45	0045	jejuni	no	yes	yes
Campylobacter jejuni Dg20a	PubMLST	25937	7259	0045	jejuni	no	yes	yes
Campylobacter jejuni Dg224	PubMLST	25989	7259	0045	jejuni	no	yes	yes
Campylobacter jejuni Dg24	PubMLST	25944	583	0045	jejuni	no	yes	yes
Campylobacter jejuni Dg246	PubMLST	26063	7259	0045	jejuni	no	yes	yes
Campylobacter jejuni Dg25a	PubMLST	25955	583	0045	jejuni	no	yes	yes
Campylobacter jejuni Dg260	PubMLST	26051	583	0045	jejuni	no	yes	yes
Campylobacter jejuni Dg26b	PubMLST	25959	7259	0045	jejuni	no	yes	yes

Campylobacter jejuni Dg273	PubMLST	26038	45	0045	jejuni	no	yes	yes
Campylobacter jejuni Dg275	PubMLST	26058	7259	0045	jejuni	no	yes	yes
Campylobacter jejuni Dg282_R	PubMLST	28057	45	0045	jejuni	no	yes	yes
Campylobacter jejuni Dg28a	PubMLST	25963	583	0045	jejuni	no	yes	yes
Campylobacter jejuni Dg292	PubMLST	25964	45	0045	jejuni	no	yes	yes
Campylobacter jejuni Dg294	PubMLST	26022	7256	0045	jejuni	no	yes	yes
Campylobacter jejuni Dg304	PubMLST	25991	7259	0045	jejuni	no	yes	yes
Campylobacter jejuni Dg307	PubMLST	25990	7259	0045	jejuni	no	yes	yes
Campylobacter jejuni Dg345	PubMLST	26009	7256	0045	jejuni	no	yes	yes
Campylobacter jejuni Dg366	PubMLST	26013	583	0045	jejuni	no	yes	yes
Campylobacter jejuni Dg374	PubMLST	26029	137	0045	jejuni	no	yes	yes
Campylobacter jejuni Dg379	PubMLST	26037	137	0045	jejuni	no	yes	yes
Campylobacter jejuni Dg382	PubMLST	26055	137	0045	jejuni	no	yes	yes
Campylobacter jejuni Dg383	PubMLST	26062	45	0045	jejuni	no	yes	yes
Campylobacter jejuni Dg44a	PubMLST	25950	7259	0045	jejuni	no	yes	yes
Campylobacter jejuni Dg57a	PubMLST	25947	7259	0045	jejuni	no	yes	yes
Campylobacter jejuni Dg95	PubMLST	26020	7259	0045	jejuni	no	yes	yes
Campylobacter jejuni OXC4559	PubMLST	24039	583	0045	jejuni	no	yes	yes
Campylobacter jejuni OXC4721	PubMLST	18430	137	0045	jejuni	no	yes	yes
Campylobacter jejuni OXC4850	PubMLST	22163	583	0045	jejuni	no	yes	yes
Campylobacter jejuni OXC4893	PubMLST	24459	45	0045	jejuni	no	yes	yes
Campylobacter jejuni OXC4907	PubMLST	24467	137	0045	jejuni	no	yes	yes
Campylobacter jejuni OXC5032	PubMLST	24699	137	0045	jejuni	no	yes	yes
Campylobacter jejuni OXC5090	PubMLST	24731	45	0045	jejuni	no	yes	yes
Campylobacter jejuni OXC5091	PubMLST	24732	583	0045	jejuni	no	yes	yes
Campylobacter jejuni OXC5129	PubMLST	24751	334	0045	jejuni	no	yes	yes
Campylobacter jejuni OXC5130	PubMLST	24752	45	0045	jejuni	no	yes	yes
Campylobacter jejuni OXC5164	PubMLST	24766	45	0045	jejuni	no	yes	yes
Campylobacter jejuni OXC5180	PubMLST	24777	45	0045	jejuni	no	yes	yes
Campylobacter jejuni OXC5192	PubMLST	24787	45	0045	jejuni	no	yes	yes
Campylobacter jejuni OXC5208	PubMLST	24798	6017	0045	jejuni	no	yes	yes
Campylobacter jejuni OXC5224	PubMLST	24810	583	0045	jejuni	no	yes	yes
Campylobacter jejuni OXC5225	PubMLST	24811	45	0045	jejuni	no	yes	yes
Campylobacter jejuni OXC5231	PubMLST	24817	45	0045	jejuni	no	yes	yes
Campylobacter jejuni OXC5236	PubMLST	24819	45	0045	jejuni	no	yes	yes
Campylobacter jejuni OXC5318	PubMLST	25100	137	0045	jejuni	no	yes	yes
Campylobacter jejuni OXC5331	PubMLST	21315	137	0045	jejuni	no	yes	yes
Campylobacter jejuni OXC5332	PubMLST	21316	45	0045	jejuni	no	yes	yes
Campylobacter jejuni OXC5420	PubMLST	21372	45	0045	jejuni	no	yes	yes
Campylobacter jejuni OXC5434	PubMLST	21380	137	0045	jejuni	no	yes	yes
Campylobacter jejuni OXC5744	PubMLST	21471	137	0045	jejuni	no	yes	yes
Campylobacter jejuni OXC5816	PubMLST	23564	45	0045	jejuni	no	yes	yes
Campylobacter jejuni OXC5850	PubMLST	23667	45	0045	jejuni	no	yes	yes
Campylobacter jejuni OXC6137	PubMLST	29328	45	0045	jejuni	no	yes	yes
Campylobacter jejuni OXC6259	PubMLST	16056	45	0045	jejuni	no	yes	yes
Campylobacter jejuni OXC6278	PubMLST	16075	45	0045	jejuni	no	yes	yes
Campylobacter jejuni OXC6293	PubMLST	16090	45	0045	jejuni	no	yes	yes
Campylobacter jejuni OXC6294	PubMLST	16091	583	0045	jejuni	no	yes	yes
Campylobacter jejuni OXC6313	PubMLST	16110	45	0045	jejuni	no	yes	yes
Campylobacter jejuni OXC6314	PubMLST	16111	45	0045	jejuni	no	yes	yes
Campylobacter jejuni OXC6321	PubMLST	16118	45	0045	jejuni	no	yes	yes
Campylobacter jejuni OXC6330	PubMLST	16127	583	0045	jejuni	no	yes	yes
Campylobacter jejuni OXC6339	PubMLST	16136	45	0045	jejuni	no	yes	yes
Campylobacter jejuni OXC6351	PubMLST	16148	45	0045	jejuni	no	yes	yes
Campylobacter jejuni OXC6365	PubMLST	16162	45	0045	jejuni	no	yes	yes
Campylobacter jejuni OXC6419	PubMLST	16214	45	0045	jejuni	no	yes	yes
Campylobacter jejuni OXC6437	PubMLST	16232	45	0045	jejuni	no	yes	yes
Campylobacter jejuni OXC6448	PubMLST	16241	137	0045	jejuni	no	yes	yes
Campylobacter jejuni OXC6515	PubMLST	16307	845	0045	jejuni	no	yes	yes
Campylobacter jejuni OXC6536	PubMLST	16328	45	0045	jejuni	no	yes	yes
Campylobacter jejuni OXC6592	PubMLST	16343	137	0045	jejuni	no	yes	yes
Campylobacter jejuni OXC6594	PubMLST	16345	45	0045	jejuni	no	yes	yes
Campylobacter jejuni OXC6624	PubMLST	16373	45	0045	jejuni	no	yes	yes
Campylobacter jejuni OXC6797	PubMLST	18354	45	0045	jejuni	no	yes	yes
Campylobacter jejuni OXC6803	PubMLST	18360	45	0045	jejuni	no	yes	yes
Campylobacter jejuni OXC6819	PubMLST	18376	583	0045	jejuni	no	yes	yes
Campylobacter jejuni OXC6894	PubMLST	22089	45	0045	jejuni	no	yes	yes
Campylobacter jejuni OXC6895	PubMLST	22090	45	0045	jejuni	no	yes	yes
Campylobacter jejuni OXC6896	PubMLST	22091	45	0045	jejuni	no	yes	yes
Campylobacter jejuni OXC6916	PubMLST	22111	45	0045	jejuni	no	yes	yes
Campylobacter jejuni OXC6970	PubMLST	21144	583	0045	jejuni	no	yes	yes
Campylobacter jejuni OXC7014	PubMLST	22201	45	0045	jejuni	no	yes	yes
Campylobacter jejuni OXC7018	PubMLST	22205	45	0045	jejuni	no	yes	yes
Campylobacter jejuni OXC7025	PubMLST	22212	45	0045	jejuni	no	yes	yes
Campylobacter jejuni OXC7026	PubMLST	22213	2219	0045	jejuni	no	yes	yes
Campylobacter jejuni OXC7032	PubMLST	22218	583	0045	jejuni	no	yes	yes
Campylobacter jejuni OXC7061	PubMLST	22245	45	0045	jejuni	no	yes	yes
Campylobacter jejuni OXC7067	PubMLST	22251	137	0045	jejuni	no	yes	yes
Campylobacter jejuni OXC7074	PubMLST	22258	45	0045	jejuni	no	yes	yes
Campylobacter jejuni OXC7090	PubMLST	22272	45	0045	jejuni	no	yes	yes
Campylobacter jejuni OXC7093	PubMLST	22275	11	0045	jejuni	no	yes	yes
Campylobacter jejuni OXC7099	PubMLST	22281	45	0045	jejuni	no	yes	yes
Campylobacter jejuni OXC7103	PubMLST	21185	45	0045	jejuni	no	yes	yes
Campylobacter jejuni OXC7113	PubMLST	21195	538	0045	jejuni	no	yes	yes
Campylobacter jejuni OXC7129	PubMLST	21210	45	0045	jejuni	no	yes	yes
Campylobacter jejuni OXC7167	PubMLST	21248	6886	0045	jejuni	no	yes	yes
Campylobacter jejuni OXC7169	PubMLST	21250	45	0045	jejuni	no	yes	yes
Campylobacter jejuni OXC7170	PubMLST	22661	137	0045	jejuni	no	yes	yes
Campylobacter jejuni OXC7179	PubMLST	22670	45	0045	jejuni	no	yes	yes
Campylobacter jejuni OXC7185	PubMLST	22676	45	0045	jejuni	no	yes	yes
Campylobacter jejuni OXC7186	PubMLST	22677	45	0045	jejuni	no	yes	yes
Campylobacter jejuni OXC7216	PubMLST	22707	45	0045	jejuni	no	yes	yes

Campylobacter jejuni OXC7225	PubMLST	22716	45	0045	jejuni	no	yes	yes
Campylobacter jejuni OXC7229	PubMLST	24127	45	0045	jejuni	no	yes	yes
Campylobacter jejuni OXC7233	PubMLST	22723	45	0045	jejuni	no	yes	yes
Campylobacter jejuni OXC7235	PubMLST	22725	45	0045	jejuni	no	yes	yes
Campylobacter jejuni OXC7239	PubMLST	22729	45	0045	jejuni	no	yes	yes
Campylobacter jejuni OXC7249	PubMLST	22291	137	0045	jejuni	no	yes	yes
Campylobacter jejuni OXC7267	PubMLST	22308	45	0045	jejuni	no	yes	yes
Campylobacter jejuni OXC7275	PubMLST	22316	45	0045	jejuni	no	yes	yes
Campylobacter jejuni OXC7281	PubMLST	22322	137	0045	jejuni	no	yes	yes
Campylobacter jejuni OXC7283	PubMLST	22324	137	0045	jejuni	no	yes	yes
Campylobacter jejuni OXC7284	PubMLST	22325	45	0045	jejuni	no	yes	yes
Campylobacter jejuni OXC7303	PubMLST	22344	45	0045	jejuni	no	yes	yes
Campylobacter jejuni OXC7309	PubMLST	22350	45	0045	jejuni	no	yes	yes
Campylobacter jejuni OXC7315	PubMLST	22356	45	0045	jejuni	no	yes	yes
Campylobacter jejuni OXC7326	PubMLST	22365	45	0045	jejuni	no	yes	yes
Campylobacter jejuni OXC7331	PubMLST	22369	137	0045	jejuni	no	yes	yes
Campylobacter jejuni OXC7340	PubMLST	22377	45	0045	jejuni	no	yes	yes
Campylobacter jejuni OXC7342	PubMLST	23883	45	0045	jejuni	no	yes	yes
Campylobacter jejuni OXC7344	PubMLST	23884	334	0045	jejuni	no	yes	yes
Campylobacter jejuni OXC7346	PubMLST	23886	334	0045	jejuni	no	yes	yes
Campylobacter jejuni OXC7369	PubMLST	23907	45	0045	jejuni	no	yes	yes
Campylobacter jejuni OXC7377	PubMLST	23915	2109	0045	jejuni	no	yes	yes
Campylobacter jejuni OXC7401	PubMLST	23937	45	0045	jejuni	no	yes	yes
Campylobacter jejuni OXC7425	PubMLST	23959	137	0045	jejuni	no	yes	yes
Campylobacter jejuni OXC7484	PubMLST	24012	45	0045	jejuni	no	yes	yes
Campylobacter jejuni OXC7496	PubMLST	24024	45	0045	jejuni	no	yes	yes
Campylobacter jejuni OXC7587	PubMLST	24521	583	0045	jejuni	no	yes	yes
Campylobacter jejuni OXC7634	PubMLST	24563	45	0045	jejuni	no	yes	yes
Campylobacter jejuni OXC7659	PubMLST	24914	45	0045	jejuni	no	yes	yes
Campylobacter jejuni OXC7708	PubMLST	25629	45	0045	jejuni	no	yes	yes
Campylobacter jejuni OXC7735	PubMLST	24973	45	0045	jejuni	no	yes	yes
Campylobacter jejuni OXC7745	PubMLST	24980	45	0045	jejuni	no	yes	yes
Campylobacter jejuni OXC7752	PubMLST	24986	538	0045	jejuni	no	yes	yes
Campylobacter jejuni OXC7766	PubMLST	24575	45	0045	jejuni	no	yes	yes
Campylobacter jejuni OXC7776	PubMLST	24583	45	0045	jejuni	no	yes	yes
Campylobacter jejuni OXC7780	PubMLST	24587	137	0045	jejuni	no	yes	yes
Campylobacter jejuni OXC7782	PubMLST	24589	137	0045	jejuni	no	yes	yes
Campylobacter jejuni OXC7786	PubMLST	24590	137	0045	jejuni	no	yes	yes
Campylobacter jejuni OXC7788	PubMLST	24591	45	0045	jejuni	no	yes	yes
Campylobacter jejuni OXC7793	PubMLST	24596	45	0045	jejuni	no	yes	yes
Campylobacter jejuni OXC7812	PubMLST	24610	45	0045	jejuni	no	yes	yes
Campylobacter jejuni OXC7813	PubMLST	24611	45	0045	jejuni	no	yes	yes
Campylobacter jejuni OXC7911	PubMLST	25041	45	0045	jejuni	no	yes	yes
Campylobacter jejuni OXC7922	PubMLST	25051	45	0045	jejuni	no	yes	yes
Campylobacter jejuni OXC7923	PubMLST	25052	45	0045	jejuni	no	yes	yes
Campylobacter jejuni OXC7972	PubMLST	25387	45	0045	jejuni	no	yes	yes
Campylobacter jejuni OXC7975	PubMLST	25390	1701	0045	jejuni	no	yes	yes
Campylobacter jejuni OXC7979	PubMLST	25394	137	0045	jejuni	no	yes	yes
Campylobacter jejuni OXC7987	PubMLST	25399	2197	0045	jejuni	no	yes	yes
Campylobacter jejuni OXC7998	PubMLST	25597	45	0045	jejuni	no	yes	yes
Campylobacter jejuni OXC8007	PubMLST	25491	45	0045	jejuni	no	yes	yes
Campylobacter jejuni OXC8016	PubMLST	25493	137	0045	jejuni	no	yes	yes
Campylobacter jejuni OXC8046	PubMLST	25508	230	0045	jejuni	no	yes	yes
Campylobacter jejuni OXC8055	PubMLST	25600	334	0045	jejuni	no	yes	yes
Campylobacter jejuni OXC8062	PubMLST	25512	334	0045	jejuni	no	yes	yes
Campylobacter jejuni OXC8065	PubMLST	25515	45	0045	jejuni	no	yes	yes
Campylobacter jejuni OXC8072	PubMLST	25606	45	0045	jejuni	no	yes	yes
Campylobacter jejuni OXC8080	PubMLST	25519	583	0045	jejuni	no	yes	yes
Campylobacter jejuni OXC8100	PubMLST	25528	137	0045	jejuni	no	yes	yes
Campylobacter jejuni OXC8118	PubMLST	25433	137	0045	jejuni	no	yes	yes
Campylobacter jejuni OXC8128	PubMLST	25459	583	0045	jejuni	no	yes	yes
Campylobacter jejuni OXC8170	PubMLST	25547	45	0045	jejuni	no	yes	yes
Campylobacter jejuni OXC8207	PubMLST	27912	45	0045	jejuni	no	yes	yes
Campylobacter jejuni OXC8213	PubMLST	27916	45	0045	jejuni	no	yes	yes
Campylobacter jejuni OXC8228	PubMLST	27930	45	0045	jejuni	no	yes	yes
Campylobacter jejuni OXC8236	PubMLST	27938	11	0045	jejuni	no	yes	yes
Campylobacter jejuni OXC8238	PubMLST	27939	45	0045	jejuni	no	yes	yes
Campylobacter jejuni OXC8246	PubMLST	27945	137	0045	jejuni	no	yes	yes
Campylobacter jejuni OXC8267	PubMLST	27961	45	0045	jejuni	no	yes	yes
Campylobacter jejuni OXC8362	PubMLST	28050	45	0045	jejuni	no	yes	yes
Campylobacter jejuni OXC8388	PubMLST	28581	137	0045	jejuni	no	yes	yes
Campylobacter jejuni OXC8483	PubMLST	28676	45	0045	jejuni	no	yes	yes
Campylobacter jejuni OXC8536	PubMLST	28726	583	0045	jejuni	no	yes	yes
Campylobacter jejuni OXC8578	PubMLST	28915	45	0045	jejuni	no	yes	yes
Campylobacter jejuni OXC8615	PubMLST	29071	45	0045	jejuni	no	yes	yes
Campylobacter jejuni OXC8796	PubMLST	30485	45	0045	jejuni	no	yes	yes
Campylobacter jejuni OXC8809	PubMLST	30498	1003	0045	jejuni	no	yes	yes
Campylobacter jejuni OXC8820	PubMLST	30508	137	0045	jejuni	no	yes	yes
Campylobacter jejuni OXC8841	PubMLST	30527	45	0045	jejuni	no	yes	yes
Campylobacter jejuni OXC8842	PubMLST	30528	137	0045	jejuni	no	yes	yes
Campylobacter jejuni OXC8843	PubMLST	30529	137	0045	jejuni	no	yes	yes
Campylobacter jejuni OXC8844	PubMLST	30530	45	0045	jejuni	no	yes	yes
Campylobacter jejuni OXC8857	PubMLST	30543	45	0045	jejuni	no	yes	yes
Campylobacter jejuni OXC8888	PubMLST	30572	45	0045	jejuni	no	yes	yes
Campylobacter jejuni OXC8894	PubMLST	30578	45	0045	jejuni	no	yes	yes
Campylobacter jejuni OXC8896	PubMLST	30579	45	0045	jejuni	no	yes	yes
Campylobacter jejuni OXC8900	PubMLST	30583	137	0045	jejuni	no	yes	yes
Campylobacter jejuni OXC8906	PubMLST	30589	45	0045	jejuni	no	yes	yes
Campylobacter jejuni OXC8919	PubMLST	30602	45	0045	jejuni	no	yes	yes
Campylobacter jejuni OXC8936	PubMLST	30619	1701	0045	jejuni	no	yes	yes
Campylobacter jejuni OXC8937	PubMLST	30620	45	0045	jejuni	no	yes	yes
Campylobacter jejuni OXC8956	PubMLST	30639	137	0045	jejuni	no	yes	yes

Campylobacter jejuni OXC8961	PubMLST	30643	583	0045	jejuni	no	yes	yes
Campylobacter jejuni OXC8968	PubMLST	30650	538	0045	jejuni	no	yes	yes
Campylobacter jejuni OXC8970	PubMLST	30652	230	0045	jejuni	no	yes	yes
Campylobacter jejuni OXC8972	PubMLST	30654	45	0045	jejuni	no	yes	yes
Campylobacter jejuni OXC8993	PubMLST	30429	45	0045	jejuni	no	yes	yes
Campylobacter jejuni OXC8996	PubMLST	30677	137	0045	jejuni	no	yes	yes
Campylobacter jejuni OXC8997	PubMLST	30678	230	0045	jejuni	no	yes	yes
Campylobacter jejuni UoH4028	PubMLST	2681	45	0045	jejuni	no	yes	yes
Campylobacter jejuni UoH4031	PubMLST	2692	45	0045	jejuni	no	yes	yes
Campylobacter jejuni UoH4947	PubMLST	2682	45	0045	jejuni	no	yes	yes
Campylobacter jejuni UoH4948	PubMLST	2683	45	0045	jejuni	no	yes	yes
Campylobacter jejuni UoH540	PubMLST	2695	45	0045	jejuni	no	yes	yes
Campylobacter jejuni UoH543	PubMLST	2697	45	0045	jejuni	no	yes	yes
Campylobacter jejuni UoH544	PubMLST	2698	45	0045	jejuni	no	yes	yes
Campylobacter jejuni UoH6236	PubMLST	2685	45	0045	jejuni	no	yes	yes
Campylobacter jejuni UoH6237	PubMLST	2684	45	0045	jejuni	no	yes	yes
Campylobacter jejuni UoH6497	PubMLST	2691	45	0045	jejuni	no	yes	yes
Campylobacter jejuni UoH6498	PubMLST	2690	45	0045	jejuni	no	yes	yes
Campylobacter jejuni UoH6538	PubMLST	2686	45	0045	jejuni	no	yes	yes
Campylobacter jejuni UoH6541	PubMLST	2689	45	0045	jejuni	no	yes	yes
Campylobacter jejuni UoHIHV116260	PubMLST	2693	45	0045	jejuni	no	yes	yes
Campylobacter jejuni UoHIHV116292	PubMLST	2694	45	0045	jejuni	no	yes	yes
Campylobacter jejuni UoHT-71726	PubMLST	2699	45	0045	jejuni	no	yes	yes
Campylobacter jejuni UoHT-71727	PubMLST	2700	45	0045	jejuni	no	yes	yes
Campylobacter jejuni UoHT-71731	PubMLST	2701	45	0045	jejuni	no	yes	yes
Campylobacter jejuni UoHT-71732	PubMLST	2702	45	0045	jejuni	no	yes	yes
Campylobacter jejuni 1893	Genbank/EMBL/DDBJ	AIPK01	38	0048	jejuni	no	yes	yes
Campylobacter jejuni 1997-4	Genbank/EMBL/DDBJ	AIOW01	475	0048	jejuni	no	yes	yes
Campylobacter jejuni 86605	Genbank/EMBL/DDBJ	AIOJ01	4840	0048	jejuni	no	yes	yes
Campylobacter jejuni ARI_1129_R	PubMLST	24324	48	0048	jejuni	no	yes	yes
Campylobacter jejuni ARI_1332_R	PubMLST	24329	48	0048	jejuni	no	yes	yes
Campylobacter jejuni CJ2	Genbank/EMBL/DDBJ	AUUM01	918	0048	jejuni	no	yes	yes
Campylobacter jejuni CJ7016_R	PubMLST	24343	48	0048	jejuni	no	yes	yes
Campylobacter jejuni D2600	Genbank/EMBL/DDBJ	AGTF01	429	0048	jejuni	no	yes	yes
Campylobacter jejuni H133940557	PubMLST	2659	918	0048	jejuni	no	yes	yes
Campylobacter jejuni H143900545	PubMLST	31016	48	0048	jejuni	no	yes	yes
Campylobacter jejuni H143900546	PubMLST	31013	48	0048	jejuni	no	yes	yes
Campylobacter jejuni H22082	Genbank/EMBL/DDBJ	AEIP01	474	0048	jejuni	no	yes	yes
Campylobacter jejuni OXC4562	PubMLST	24041	48	0048	jejuni	no	yes	yes
Campylobacter jejuni OXC4603	PubMLST	24070	48	0048	jejuni	no	yes	yes
Campylobacter jejuni OXC4638	PubMLST	24087	475	0048	jejuni	no	yes	yes
Campylobacter jejuni OXC4639	PubMLST	24088	475	0048	jejuni	no	yes	yes
Campylobacter jejuni OXC4680	PubMLST	24116	48	0048	jejuni	no	yes	yes
Campylobacter jejuni OXC4767	PubMLST	18460	48	0048	jejuni	no	yes	yes
Campylobacter jejuni OXC4779	PubMLST	18469	48	0048	jejuni	no	yes	yes
Campylobacter jejuni OXC4813	PubMLST	22149	48	0048	jejuni	no	yes	yes
Campylobacter jejuni OXC4841	PubMLST	22160	475	0048	jejuni	no	yes	yes
Campylobacter jejuni OXC4846	PubMLST	22162	48	0048	jejuni	no	yes	yes
Campylobacter jejuni OXC4981	PubMLST	24667	48	0048	jejuni	no	yes	yes
Campylobacter jejuni OXC4985	PubMLST	24668	475	0048	jejuni	no	yes	yes
Campylobacter jejuni OXC4988	PubMLST	24671	48	0048	jejuni	no	yes	yes
Campylobacter jejuni OXC5150	PubMLST	24764	48	0048	jejuni	no	yes	yes
Campylobacter jejuni OXC5188	PubMLST	24783	5183	0048	jejuni	no	yes	yes
Campylobacter jejuni OXC5213	PubMLST	24801	918	0048	jejuni	no	yes	yes
Campylobacter jejuni OXC5242	PubMLST	24824	48	0048	jejuni	no	yes	yes
Campylobacter jejuni OXC5334	PubMLST	21318	48	0048	jejuni	no	yes	yes
Campylobacter jejuni OXC5405	PubMLST	21361	48	0048	jejuni	no	yes	yes
Campylobacter jejuni OXC5422	PubMLST	21374	6601	0048	jejuni	no	yes	yes
Campylobacter jejuni OXC5433	PubMLST	21379	48	0048	jejuni	no	yes	yes
Campylobacter jejuni OXC5614	PubMLST	23505	475	0048	jejuni	no	yes	yes
Campylobacter jejuni OXC5626	PubMLST	23513	475	0048	jejuni	no	yes	yes
Campylobacter jejuni OXC5675	PubMLST	21422	48	0048	jejuni	no	yes	yes
Campylobacter jejuni OXC5711	PubMLST	21448	48	0048	jejuni	no	yes	yes
Campylobacter jejuni OXC5716	PubMLST	21452	38	0048	jejuni	no	yes	yes
Campylobacter jejuni OXC5814	PubMLST	23562	48	0048	jejuni	no	yes	yes
Campylobacter jejuni OXC5874	PubMLST	23609	48	0048	jejuni	no	yes	yes
Campylobacter jejuni OXC5877	PubMLST	23610	475	0048	jejuni	no	yes	yes
Campylobacter jejuni OXC6265	PubMLST	16062	48	0048	jejuni	no	yes	yes
Campylobacter jejuni OXC6272	PubMLST	16069	844	0048	jejuni	no	yes	yes
Campylobacter jejuni OXC6328	PubMLST	16125	48	0048	jejuni	no	yes	yes
Campylobacter jejuni OXC6345	PubMLST	16142	48	0048	jejuni	no	yes	yes
Campylobacter jejuni OXC6377	PubMLST	16174	48	0048	jejuni	no	yes	yes
Campylobacter jejuni OXC6389	PubMLST	16186	475	0048	jejuni	no	yes	yes
Campylobacter jejuni OXC6403	PubMLST	16198	48	0048	jejuni	no	yes	yes
Campylobacter jejuni OXC6411	PubMLST	16206	48	0048	jejuni	no	yes	yes
Campylobacter jejuni OXC6413	PubMLST	16208	429	0048	jejuni	no	yes	yes
Campylobacter jejuni OXC6421	PubMLST	16216	48	0048	jejuni	no	yes	yes
Campylobacter jejuni OXC6549	PubMLST	12884	48	0048	jejuni	no	yes	yes
Campylobacter jejuni OXC6648	PubMLST	18205	48	0048	jejuni	no	yes	yes
Campylobacter jejuni OXC6652	PubMLST	18209	48	0048	jejuni	no	yes	yes
Campylobacter jejuni OXC6693	PubMLST	18250	48	0048	jejuni	no	yes	yes
Campylobacter jejuni OXC6837	PubMLST	21556	48	0048	jejuni	no	yes	yes
Campylobacter jejuni OXC6881	PubMLST	21600	48	0048	jejuni	no	yes	yes
Campylobacter jejuni OXC6917	PubMLST	22112	48	0048	jejuni	no	yes	yes
Campylobacter jejuni OXC6918	PubMLST	22113	48	0048	jejuni	no	yes	yes
Campylobacter jejuni OXC6942	PubMLST	21116	429	0048	jejuni	no	yes	yes
Campylobacter jejuni OXC6973	PubMLST	21146	48	0048	jejuni	no	yes	yes
Campylobacter jejuni OXC7005	PubMLST	22192	48	0048	jejuni	no	yes	yes
Campylobacter jejuni OXC7012	PubMLST	22199	48	0048	jejuni	no	yes	yes
Campylobacter jejuni OXC7016	PubMLST	22203	48	0048	jejuni	no	yes	yes
Campylobacter jejuni OXC7043	PubMLST	22228	918	0048	jejuni	no	yes	yes
Campylobacter jejuni OXC7050	PubMLST	22235	48	0048	jejuni	no	yes	yes

Campylobacter jejuni OXC7112	PubMLST	21194	48	0048	jejuni	no	yes	yes
Campylobacter jejuni OXC7143	PubMLST	21224	48	0048	jejuni	no	yes	yes
Campylobacter jejuni OXC7151	PubMLST	21232	48	0048	jejuni	no	yes	yes
Campylobacter jejuni OXC7165	PubMLST	21246	48	0048	jejuni	no	yes	yes
Campylobacter jejuni OXC7174	PubMLST	22665	48	0048	jejuni	no	yes	yes
Campylobacter jejuni OXC7176	PubMLST	22667	48	0048	jejuni	no	yes	yes
Campylobacter jejuni OXC7181	PubMLST	22672	48	0048	jejuni	no	yes	yes
Campylobacter jejuni OXC7206	PubMLST	22697	48	0048	jejuni	no	yes	yes
Campylobacter jejuni OXC7208	PubMLST	22699	48	0048	jejuni	no	yes	yes
Campylobacter jejuni OXC7262	PubMLST	22303	48	0048	jejuni	no	yes	yes
Campylobacter jejuni OXC7269	PubMLST	22310	48	0048	jejuni	no	yes	yes
Campylobacter jejuni OXC7280	PubMLST	22321	48	0048	jejuni	no	yes	yes
Campylobacter jejuni OXC7374	PubMLST	23912	6754	0048	jejuni	no	yes	yes
Campylobacter jejuni OXC7383	PubMLST	23921	48	0048	jejuni	no	yes	yes
Campylobacter jejuni OXC7388	PubMLST	23926	48	0048	jejuni	no	yes	yes
Campylobacter jejuni OXC7404	PubMLST	23940	918	0048	jejuni	no	yes	yes
Campylobacter jejuni OXC7461	PubMLST	23990	48	0048	jejuni	no	yes	yes
Campylobacter jejuni OXC7483	PubMLST	25624	48	0048	jejuni	no	yes	yes
Campylobacter jejuni OXC7486	PubMLST	24014	48	0048	jejuni	no	yes	yes
Campylobacter jejuni OXC7524	PubMLST	24128	48	0048	jejuni	no	yes	yes
Campylobacter jejuni OXC7525	PubMLST	24129	6754	0048	jejuni	no	yes	yes
Campylobacter jejuni OXC7571	PubMLST	24508	48	0048	jejuni	no	yes	yes
Campylobacter jejuni OXC7582	PubMLST	25107	918	0048	jejuni	no	yes	yes
Campylobacter jejuni OXC7647	PubMLST	24904	48	0048	jejuni	no	yes	yes
Campylobacter jejuni OXC7673	PubMLST	24927	6754	0048	jejuni	no	yes	yes
Campylobacter jejuni OXC7680	PubMLST	24934	918	0048	jejuni	no	yes	yes
Campylobacter jejuni OXC7692	PubMLST	24940	48	0048	jejuni	no	yes	yes
Campylobacter jejuni OXC7703	PubMLST	25434	48	0048	jejuni	no	yes	yes
Campylobacter jejuni OXC7721	PubMLST	24963	475	0048	jejuni	no	yes	yes
Campylobacter jejuni OXC7747	PubMLST	24982	48	0048	jejuni	no	yes	yes
Campylobacter jejuni OXC7750	PubMLST	24985	48	0048	jejuni	no	yes	yes
Campylobacter jejuni OXC7769	PubMLST	24578	48	0048	jejuni	no	yes	yes
Campylobacter jejuni OXC7772	PubMLST	24580	48	0048	jejuni	no	yes	yes
Campylobacter jejuni OXC7799	PubMLST	24600	48	0048	jejuni	no	yes	yes
Campylobacter jejuni OXC7820	PubMLST	24617	48	0048	jejuni	no	yes	yes
Campylobacter jejuni OXC7821	PubMLST	24618	48	0048	jejuni	no	yes	yes
Campylobacter jejuni OXC7834	PubMLST	24631	48	0048	jejuni	no	yes	yes
Campylobacter jejuni OXC7914	PubMLST	25044	48	0048	jejuni	no	yes	yes
Campylobacter jejuni OXC8040	PubMLST	25505	48	0048	jejuni	no	yes	yes
Campylobacter jejuni OXC8064	PubMLST	25514	48	0048	jejuni	no	yes	yes
Campylobacter jejuni OXC8066	PubMLST	25416	48	0048	jejuni	no	yes	yes
Campylobacter jejuni OXC8120	PubMLST	25536	48	0048	jejuni	no	yes	yes
Campylobacter jejuni OXC8124	PubMLST	25557	48	0048	jejuni	no	yes	yes
Campylobacter jejuni OXC8125	PubMLST	25457	48	0048	jejuni	no	yes	yes
Campylobacter jejuni OXC8206	PubMLST	27911	48	0048	jejuni	no	yes	yes
Campylobacter jejuni OXC8232	PubMLST	27934	48	0048	jejuni	no	yes	yes
Campylobacter jejuni OXC8317	PubMLST	28008	48	0048	jejuni	no	yes	yes
Campylobacter jejuni OXC8343	PubMLST	28034	48	0048	jejuni	no	yes	yes
Campylobacter jejuni OXC8353	PubMLST	28051	48	0048	jejuni	no	yes	yes
Campylobacter jejuni OXC8413	PubMLST	28604	918	0048	jejuni	no	yes	yes
Campylobacter jejuni OXC8473	PubMLST	28666	48	0048	jejuni	no	yes	yes
Campylobacter jejuni OXC8490	PubMLST	28682	48	0048	jejuni	no	yes	yes
Campylobacter jejuni OXC8509	PubMLST	28700	48	0048	jejuni	no	yes	yes
Campylobacter jejuni OXC8535	PubMLST	28725	48	0048	jejuni	no	yes	yes
Campylobacter jejuni OXC8561	PubMLST	28901	48	0048	jejuni	no	yes	yes
Campylobacter jejuni OXC8595	PubMLST	28928	48	0048	jejuni	no	yes	yes
Campylobacter jejuni OXC8612	PubMLST	28987	48	0048	jejuni	no	yes	yes
Campylobacter jejuni OXC8652	PubMLST	28966	48	0048	jejuni	no	yes	yes
Campylobacter jejuni OXC8708	PubMLST	29037	2343	0048	jejuni	no	yes	yes
Campylobacter jejuni OXC8710	PubMLST	29039	48	0048	jejuni	no	yes	yes
Campylobacter jejuni OXC8791	PubMLST	30480	48	0048	jejuni	no	yes	yes
Campylobacter jejuni OXC8795	PubMLST	30484	48	0048	jejuni	no	yes	yes
Campylobacter jejuni OXC8812	PubMLST	30427	48	0048	jejuni	no	yes	yes
Campylobacter jejuni OXC8826	PubMLST	30514	48	0048	jejuni	no	yes	yes
Campylobacter jejuni OXC8869	PubMLST	30555	48	0048	jejuni	no	yes	yes
Campylobacter jejuni OXC8904	PubMLST	30587	48	0048	jejuni	no	yes	yes
Campylobacter jejuni OXC8915	PubMLST	30598	48	0048	jejuni	no	yes	yes
Campylobacter jejuni OXC8920	PubMLST	30603	48	0048	jejuni	no	yes	yes
Campylobacter jejuni OXC8925	PubMLST	30608	48	0048	jejuni	no	yes	yes
Campylobacter jejuni OXC8948	PubMLST	30631	492	0048	jejuni	no	yes	yes
Campylobacter jejuni OXC8983	PubMLST	30665	48	0048	jejuni	no	yes	yes
Campylobacter jejuni P110B	Genbank/EMBL/DDBJ	AEIO01	474	0048	jejuni	no	yes	yes
Campylobacter jejuni OXC5329	PubMLST	21313	49	0049	jejuni	no	yes	yes
Campylobacter jejuni OXC5368	PubMLST	21336	5483	0049	jejuni	no	yes	yes
Campylobacter jejuni OXC5854	PubMLST	23594	49	0049	jejuni	no	yes	yes
Campylobacter jejuni OXC7197	PubMLST	22688	49	0049	jejuni	no	yes	yes
Campylobacter jejuni OXC7469	PubMLST	23998	49	0049	jejuni	no	yes	yes
Campylobacter jejuni OXC7559	PubMLST	24496	49	0049	jejuni	no	yes	yes
Campylobacter jejuni OXC7885	PubMLST	25114	49	0049	jejuni	no	yes	yes
Campylobacter jejuni OXC8127	PubMLST	25558	49	0049	jejuni	no	yes	yes
Campylobacter jejuni OXC8298	PubMLST	27989	7179	0049	jejuni	no	yes	yes
Campylobacter jejuni OXC8671	PubMLST	29004	7305	0049	jejuni	no	yes	yes
Campylobacter jejuni OXC8876	PubMLST	30562	5483	0049	jejuni	no	yes	yes
Campylobacter jejuni OXC8914	PubMLST	30597	5483	0049	jejuni	no	yes	yes
Campylobacter jejuni OXC8995	PubMLST	30676	49	0049	jejuni	no	yes	yes
Campylobacter jejuni 10186	Genbank/EMBL/DDBJ	AUUG01	1919	0052	jejuni	no	yes	yes
Campylobacter jejuni 20176	Genbank/EMBL/DDBJ	AUUQ01	161	0052	jejuni	no	yes	yes
Campylobacter jejuni CG8421	Genbank/EMBL/DDBJ	ABGQ01	1919	0052	jejuni	no	yes	yes
Campylobacter jejuni LMG 9081	Genbank/EMBL/DDBJ	AIOM01	52	0052	jejuni	no	yes	yes
Campylobacter jejuni OXC4646	PubMLST	24092	775	0052	jejuni	no	yes	yes
Campylobacter jejuni OXC5005	PubMLST	24682	52	0052	jejuni	no	yes	yes
Campylobacter jejuni OXC5136	PubMLST	24756	775	0052	jejuni	no	yes	yes

Campylobacter jejuni OXC5178	PubMLST	24775	775	0052	jejuni	no	yes	yes
Campylobacter jejuni OXC5205	PubMLST	24795	52	0052	jejuni	no	yes	yes
Campylobacter jejuni OXC5215	PubMLST	24803	52	0052	jejuni	no	yes	yes
Campylobacter jejuni OXC5608	PubMLST	23503	52	0052	jejuni	no	yes	yes
Campylobacter jejuni OXC5674	PubMLST	21421	52	0052	jejuni	no	yes	yes
Campylobacter jejuni OXC5747	PubMLST	21473	775	0052	jejuni	no	yes	yes
Campylobacter jejuni OXC5748	PubMLST	21474	775	0052	jejuni	no	yes	yes
Campylobacter jejuni OXC5751	PubMLST	21476	775	0052	jejuni	no	yes	yes
Campylobacter jejuni OXC5857	PubMLST	23597	775	0052	jejuni	no	yes	yes
Campylobacter jejuni OXC5898	PubMLST	23628	52	0052	jejuni	no	yes	yes
Campylobacter jejuni OXC5900	PubMLST	23629	52	0052	jejuni	no	yes	yes
Campylobacter jejuni OXC5917	PubMLST	23642	52	0052	jejuni	no	yes	yes
Campylobacter jejuni OXC6368	PubMLST	16165	52	0052	jejuni	no	yes	yes
Campylobacter jejuni OXC6432	PubMLST	16227	614	0052	jejuni	no	yes	yes
Campylobacter jejuni OXC6441	PubMLST	16235	52	0052	jejuni	no	yes	yes
Campylobacter jejuni OXC6669	PubMLST	18226	775	0052	jejuni	no	yes	yes
Campylobacter jejuni OXC6737	PubMLST	18294	52	0052	jejuni	no	yes	yes
Campylobacter jejuni OXC6742	PubMLST	18299	52	0052	jejuni	no	yes	yes
Campylobacter jejuni OXC6768	PubMLST	18325	2275	0052	jejuni	no	yes	yes
Campylobacter jejuni OXC6908	PubMLST	22103	52	0052	jejuni	no	yes	yes
Campylobacter jejuni OXC7044	PubMLST	22229	52	0052	jejuni	no	yes	yes
Campylobacter jejuni OXC7189	PubMLST	22680	52	0052	jejuni	no	yes	yes
Campylobacter jejuni OXC7528	PubMLST	24132	52	0052	jejuni	no	yes	yes
Campylobacter jejuni OXC7545	PubMLST	25104	2100	0052	jejuni	no	yes	yes
Campylobacter jejuni OXC7567	PubMLST	24504	775	0052	jejuni	no	yes	yes
Campylobacter jejuni OXC7715	PubMLST	27867	52	0052	jejuni	no	yes	yes
Campylobacter jejuni OXC7906	PubMLST	25069	52	0052	jejuni	no	yes	yes
Campylobacter jejuni OXC7947	PubMLST	25476	52	0052	jejuni	no	yes	yes
Campylobacter jejuni OXC7956	PubMLST	25375	52	0052	jejuni	no	yes	yes
Campylobacter jejuni OXC8023	PubMLST	25588	52	0052	jejuni	no	yes	yes
Campylobacter jejuni OXC8172	PubMLST	25548	52	0052	jejuni	no	yes	yes
Campylobacter jejuni OXC8181	PubMLST	25444	52	0052	jejuni	no	yes	yes
Campylobacter jejuni OXC8187	PubMLST	27892	52	0052	jejuni	no	yes	yes
Campylobacter jejuni OXC8209	PubMLST	27914	52	0052	jejuni	no	yes	yes
Campylobacter jejuni OXC8280	PubMLST	28563	52	0052	jejuni	no	yes	yes
Campylobacter jejuni OXC8286	PubMLST	28564	52	0052	jejuni	no	yes	yes
Campylobacter jejuni OXC8349	PubMLST	28039	52	0052	jejuni	no	yes	yes
Campylobacter jejuni OXC8366	PubMLST	28568	52	0052	jejuni	no	yes	yes
Campylobacter jejuni OXC8426	PubMLST	28617	52	0052	jejuni	no	yes	yes
Campylobacter jejuni OXC8596	PubMLST	28929	775	0052	jejuni	no	yes	yes
Campylobacter jejuni OXC8617	PubMLST	28944	52	0052	jejuni	no	yes	yes
Campylobacter jejuni 140-16	Genbank/EMBL/DDBJ	AIPF01	5161	0061	jejuni	no	yes	yes
Campylobacter jejuni 1997-7	Genbank/EMBL/DDBJ	AIOX01	61	0061	jejuni	no	yes	yes
Campylobacter jejuni 2008-872	Genbank/EMBL/DDBJ	AIOR01	61	0061	jejuni	no	yes	yes
Campylobacter jejuni 7802A	PubMLST	24250	61	0061	jejuni	no	yes	yes
Campylobacter jejuni Dg327	PubMLST	25943	61	0061	jejuni	no	yes	yes
Campylobacter jejuni NC16_R	PubMLST	24323	61	0061	jejuni	no	yes	yes
Campylobacter jejuni NS_36_R	PubMLST	24331	61	0061	jejuni	no	yes	yes
Campylobacter jejuni OXC4560	PubMLST	24040	61	0061	jejuni	no	yes	yes
Campylobacter jejuni OXC4579	PubMLST	24053	61	0061	jejuni	no	yes	yes
Campylobacter jejuni OXC4595	PubMLST	24064	61	0061	jejuni	no	yes	yes
Campylobacter jejuni OXC4622	PubMLST	24075	61	0061	jejuni	no	yes	yes
Campylobacter jejuni OXC4658	PubMLST	25088	61	0061	jejuni	no	yes	yes
Campylobacter jejuni OXC4670	PubMLST	24109	61	0061	jejuni	no	yes	yes
Campylobacter jejuni OXC4722	PubMLST	18431	61	0061	jejuni	no	yes	yes
Campylobacter jejuni OXC4789	PubMLST	22133	61	0061	jejuni	no	yes	yes
Campylobacter jejuni OXC4793	PubMLST	22136	61	0061	jejuni	no	yes	yes
Campylobacter jejuni OXC4798	PubMLST	22139	61	0061	jejuni	no	yes	yes
Campylobacter jejuni OXC5111	PubMLST	24739	61	0061	jejuni	no	yes	yes
Campylobacter jejuni OXC5162	PubMLST	24765	61	0061	jejuni	no	yes	yes
Campylobacter jejuni OXC5177	PubMLST	24774	61	0061	jejuni	no	yes	yes
Campylobacter jejuni OXC5439	PubMLST	21384	61	0061	jejuni	no	yes	yes
Campylobacter jejuni OXC5493	PubMLST	23496	61	0061	jejuni	no	yes	yes
Campylobacter jejuni OXC5613	PubMLST	23504	61	0061	jejuni	no	yes	yes
Campylobacter jejuni OXC5665	PubMLST	21415	61	0061	jejuni	no	yes	yes
Campylobacter jejuni OXC5682	PubMLST	21428	61	0061	jejuni	no	yes	yes
Campylobacter jejuni OXC5844	PubMLST	23587	61	0061	jejuni	no	yes	yes
Campylobacter jejuni OXC5860	PubMLST	23600	61	0061	jejuni	no	yes	yes
Campylobacter jejuni OXC5875	PubMLST	23672	61	0061	jejuni	no	yes	yes
Campylobacter jejuni OXC6268	PubMLST	16065	61	0061	jejuni	no	yes	yes
Campylobacter jejuni OXC6283	PubMLST	16080	61	0061	jejuni	no	yes	yes
Campylobacter jejuni OXC6346	PubMLST	16143	61	0061	jejuni	no	yes	yes
Campylobacter jejuni OXC6348	PubMLST	16145	81	0061	jejuni	no	yes	yes
Campylobacter jejuni OXC6402	PubMLST	16197	61	0061	jejuni	no	yes	yes
Campylobacter jejuni OXC6453	PubMLST	16245	61	0061	jejuni	no	yes	yes
Campylobacter jejuni OXC6490	PubMLST	16282	61	0061	jejuni	no	yes	yes
Campylobacter jejuni OXC6570	PubMLST	12905	61	0061	jejuni	no	yes	yes
Campylobacter jejuni OXC6579	PubMLST	12916	61	0061	jejuni	no	yes	yes
Campylobacter jejuni OXC6589	PubMLST	12926	61	0061	jejuni	no	yes	yes
Campylobacter jejuni OXC6632	PubMLST	16381	61	0061	jejuni	no	yes	yes
Campylobacter jejuni OXC6688	PubMLST	18245	61	0061	jejuni	no	yes	yes
Campylobacter jejuni OXC6769	PubMLST	18326	61	0061	jejuni	no	yes	yes
Campylobacter jejuni OXC6775	PubMLST	18332	61	0061	jejuni	no	yes	yes
Campylobacter jejuni OXC6808	PubMLST	18365	61	0061	jejuni	no	yes	yes
Campylobacter jejuni OXC6882	PubMLST	22078	61	0061	jejuni	no	yes	yes
Campylobacter jejuni OXC6936	PubMLST	21110	61	0061	jejuni	no	yes	yes
Campylobacter jejuni OXC6978	PubMLST	21151	61	0061	jejuni	no	yes	yes
Campylobacter jejuni OXC7019	PubMLST	22206	61	0061	jejuni	no	yes	yes
Campylobacter jejuni OXC7021	PubMLST	22208	61	0061	jejuni	no	yes	yes
Campylobacter jejuni OXC7023	PubMLST	22210	61	0061	jejuni	no	yes	yes
Campylobacter jejuni OXC7045	PubMLST	22230	61	0061	jejuni	no	yes	yes
Campylobacter jejuni OXC7075	PubMLST	22259	1076	0061	jejuni	no	yes	yes

Campylobacter jejuni OXC7294	PubMLST	22335	61	0061	jejuni	no	yes	yes
Campylobacter jejuni OXC7325	PubMLST	22364	61	0061	jejuni	no	yes	yes
Campylobacter jejuni OXC7343	PubMLST	25449	61	0061	jejuni	no	yes	yes
Campylobacter jejuni OXC7361	PubMLST	23900	61	0061	jejuni	no	yes	yes
Campylobacter jejuni OXC7426	PubMLST	25620	61	0061	jejuni	no	yes	yes
Campylobacter jejuni OXC7433	PubMLST	23966	61	0061	jejuni	no	yes	yes
Campylobacter jejuni OXC7452	PubMLST	23982	61	0061	jejuni	no	yes	yes
Campylobacter jejuni OXC7472	PubMLST	24001	61	0061	jejuni	no	yes	yes
Campylobacter jejuni OXC7482	PubMLST	24011	61	0061	jejuni	no	yes	yes
Campylobacter jejuni OXC7498	PubMLST	24025	1076	0061	jejuni	no	yes	yes
Campylobacter jejuni OXC7611	PubMLST	24540	61	0061	jejuni	no	yes	yes
Campylobacter jejuni OXC7644	PubMLST	24901	61	0061	jejuni	no	yes	yes
Campylobacter jejuni OXC7652	PubMLST	25452	61	0061	jejuni	no	yes	yes
Campylobacter jejuni OXC7833	PubMLST	24630	61	0061	jejuni	no	yes	yes
Campylobacter jejuni OXC7839	PubMLST	24636	61	0061	jejuni	no	yes	yes
Campylobacter jejuni OXC7864	PubMLST	24999	432	0061	jejuni	no	yes	yes
Campylobacter jejuni OXC7938	PubMLST	25063	1738	0061	jejuni	no	yes	yes
Campylobacter jejuni OXC7964	PubMLST	25380	432	0061	jejuni	no	yes	yes
Campylobacter jejuni OXC8216	PubMLST	27919	61	0061	jejuni	no	yes	yes
Campylobacter jejuni OXC8263	PubMLST	27959	6985	0061	jejuni	no	yes	yes
Campylobacter jejuni OXC8300	PubMLST	27991	61	0061	jejuni	no	yes	yes
Campylobacter jejuni OXC8318	PubMLST	28009	61	0061	jejuni	no	yes	yes
Campylobacter jejuni OXC8344	PubMLST	28035	61	0061	jejuni	no	yes	yes
Campylobacter jejuni OXC8376	PubMLST	28570	61	0061	jejuni	no	yes	yes
Campylobacter jejuni OXC8401	PubMLST	28593	61	0061	jejuni	no	yes	yes
Campylobacter jejuni OXC8406	PubMLST	28597	61	0061	jejuni	no	yes	yes
Campylobacter jejuni OXC8446	PubMLST	28637	61	0061	jejuni	no	yes	yes
Campylobacter jejuni OXC8487	PubMLST	29084	61	0061	jejuni	no	yes	yes
Campylobacter jejuni OXC8494	PubMLST	29068	61	0061	jejuni	no	yes	yes
Campylobacter jejuni OXC8643	PubMLST	28958	61	0061	jejuni	no	yes	yes
Campylobacter jejuni OXC8716	PubMLST	29079	61	0061	jejuni	no	yes	yes
Campylobacter jejuni OXC8744	PubMLST	30435	61	0061	jejuni	no	yes	yes
Campylobacter jejuni OXC8798	PubMLST	30487	61	0061	jejuni	no	yes	yes
Campylobacter jejuni OXC8839	PubMLST	30426	61	0061	jejuni	no	yes	yes
Campylobacter jejuni OXC8941	PubMLST	30624	61	0061	jejuni	no	yes	yes
Campylobacter jejuni LMG 23211	Genbank/EMBL/DDBJ	AIPO01	220	0179	jejuni	no	yes	yes
Campylobacter jejuni OXC6482	PubMLST	16274	220	0179	jejuni	no	yes	yes
Campylobacter jejuni OXC8466	PubMLST	28659	220	0179	jejuni	no	yes	yes
Campylobacter jejuni 1577	Genbank/EMBL/DDBJ	AIPH01	122	0206	jejuni	no	yes	yes
Campylobacter jejuni 2008-988	Genbank/EMBL/DDBJ	AIOS01	572	0206	jejuni	no	yes	yes
Campylobacter jejuni CJ6947_R	PubMLST	24313	206	0206	jejuni	no	yes	yes
Campylobacter jejuni H134340385	PubMLST	2662	122	0206	jejuni	no	yes	yes
Campylobacter jejuni H141140404	PubMLST	2673	122	0206	jejuni	no	yes	yes
Campylobacter jejuni LMG 23264	Genbank/EMBL/DDBJ	AIOF01	46	0206	jejuni	no	yes	yes
Campylobacter jejuni OXC4666	PubMLST	24105	572	0206	jejuni	no	yes	yes
Campylobacter jejuni OXC4724	PubMLST	18432	2078	0206	jejuni	no	yes	yes
Campylobacter jejuni OXC4747	PubMLST	18449	572	0206	jejuni	no	yes	yes
Campylobacter jejuni OXC4748	PubMLST	18450	572	0206	jejuni	no	yes	yes
Campylobacter jejuni OXC4756	PubMLST	18454	2078	0206	jejuni	no	yes	yes
Campylobacter jejuni OXC4976	PubMLST	24666	572	0206	jejuni	no	yes	yes
Campylobacter jejuni OXC5029	PubMLST	24697	572	0206	jejuni	no	yes	yes
Campylobacter jejuni OXC5199	PubMLST	24791	572	0206	jejuni	no	yes	yes
Campylobacter jejuni OXC5248	PubMLST	24829	572	0206	jejuni	no	yes	yes
Campylobacter jejuni OXC5255	PubMLST	24833	273	0206	jejuni	no	yes	yes
Campylobacter jejuni OXC5261	PubMLST	24838	273	0206	jejuni	no	yes	yes
Campylobacter jejuni OXC5312	PubMLST	25472	122	0206	jejuni	no	yes	yes
Campylobacter jejuni OXC5319	PubMLST	25475	222	0206	jejuni	no	yes	yes
Campylobacter jejuni OXC5321	PubMLST	25101	273	0206	jejuni	no	yes	yes
Campylobacter jejuni OXC5346	PubMLST	21327	206	0206	jejuni	no	yes	yes
Campylobacter jejuni OXC5373	PubMLST	21340	273	0206	jejuni	no	yes	yes
Campylobacter jejuni OXC5377	PubMLST	21343	222	0206	jejuni	no	yes	yes
Campylobacter jejuni OXC5406	PubMLST	21362	122	0206	jejuni	no	yes	yes
Campylobacter jejuni OXC5455	PubMLST	21393	5138	0206	jejuni	no	yes	yes
Campylobacter jejuni OXC5698	PubMLST	21438	572	0206	jejuni	no	yes	yes
Campylobacter jejuni OXC5734	PubMLST	21464	572	0206	jejuni	no	yes	yes
Campylobacter jejuni OXC5740	PubMLST	21467	206	0206	jejuni	no	yes	yes
Campylobacter jejuni OXC5884	PubMLST	23615	5819	0206	jejuni	no	yes	yes
Campylobacter jejuni OXC5891	PubMLST	23622	572	0206	jejuni	no	yes	yes
Campylobacter jejuni OXC5939	PubMLST	23657	206	0206	jejuni	no	yes	yes
Campylobacter jejuni OXC6252	PubMLST	16050	572	0206	jejuni	no	yes	yes
Campylobacter jejuni OXC6306	PubMLST	16103	273	0206	jejuni	no	yes	yes
Campylobacter jejuni OXC6307	PubMLST	16104	273	0206	jejuni	no	yes	yes
Campylobacter jejuni OXC6315	PubMLST	16112	206	0206	jejuni	no	yes	yes
Campylobacter jejuni OXC6382	PubMLST	16179	572	0206	jejuni	no	yes	yes
Campylobacter jejuni OXC6387	PubMLST	16184	227	0206	jejuni	no	yes	yes
Campylobacter jejuni OXC6397	PubMLST	16194	122	0206	jejuni	no	yes	yes
Campylobacter jejuni OXC6422	PubMLST	16217	572	0206	jejuni	no	yes	yes
Campylobacter jejuni OXC6430	PubMLST	16225	572	0206	jejuni	no	yes	yes
Campylobacter jejuni OXC6454	PubMLST	16246	572	0206	jejuni	no	yes	yes
Campylobacter jejuni OXC6462	PubMLST	16254	572	0206	jejuni	no	yes	yes
Campylobacter jejuni OXC6465	PubMLST	16257	572	0206	jejuni	no	yes	yes
Campylobacter jejuni OXC6470	PubMLST	16262	572	0206	jejuni	no	yes	yes
Campylobacter jejuni OXC6477	PubMLST	16269	572	0206	jejuni	no	yes	yes
Campylobacter jejuni OXC6495	PubMLST	16287	572	0206	jejuni	no	yes	yes
Campylobacter jejuni OXC6522	PubMLST	16314	206	0206	jejuni	no	yes	yes
Campylobacter jejuni OXC6525	PubMLST	16317	122	0206	jejuni	no	yes	yes
Campylobacter jejuni OXC6532	PubMLST	16324	273	0206	jejuni	no	yes	yes
Campylobacter jejuni OXC6534	PubMLST	16326	122	0206	jejuni	no	yes	yes
Campylobacter jejuni OXC6553	PubMLST	12888	273	0206	jejuni	no	yes	yes
Campylobacter jejuni OXC6580	PubMLST	12917	572	0206	jejuni	no	yes	yes
Campylobacter jejuni OXC6623	PubMLST	16372	222	0206	jejuni	no	yes	yes
Campylobacter jejuni OXC6661	PubMLST	18218	273	0206	jejuni	no	yes	yes

Campylobacter jejuni OXC6676	PubMLST	18233	5809	0206	jejuni	no	yes	yes
Campylobacter jejuni OXC6719	PubMLST	18276	572	0206	jejuni	no	yes	yes
Campylobacter jejuni OXC6720	PubMLST	18277	572	0206	jejuni	no	yes	yes
Campylobacter jejuni OXC6723	PubMLST	18280	5819	0206	jejuni	no	yes	yes
Campylobacter jejuni OXC6739	PubMLST	18296	122	0206	jejuni	no	yes	yes
Campylobacter jejuni OXC6754	PubMLST	18311	122	0206	jejuni	no	yes	yes
Campylobacter jejuni OXC6793	PubMLST	18350	572	0206	jejuni	no	yes	yes
Campylobacter jejuni OXC6840	PubMLST	21559	122	0206	jejuni	no	yes	yes
Campylobacter jejuni OXC6858	PubMLST	21577	273	0206	jejuni	no	yes	yes
Campylobacter jejuni OXC6891	PubMLST	22086	572	0206	jejuni	no	yes	yes
Campylobacter jejuni OXC6939	PubMLST	21113	572	0206	jejuni	no	yes	yes
Campylobacter jejuni OXC6947	PubMLST	21121	206	0206	jejuni	no	yes	yes
Campylobacter jejuni OXC6950	PubMLST	21124	122	0206	jejuni	no	yes	yes
Campylobacter jejuni OXC7006	PubMLST	22193	206	0206	jejuni	no	yes	yes
Campylobacter jejuni OXC7009	PubMLST	22196	122	0206	jejuni	no	yes	yes
Campylobacter jejuni OXC7036	PubMLST	22222	122	0206	jejuni	no	yes	yes
Campylobacter jejuni OXC7039	PubMLST	22225	122	0206	jejuni	no	yes	yes
Campylobacter jejuni OXC7042	PubMLST	22227	122	0206	jejuni	no	yes	yes
Campylobacter jejuni OXC7056	PubMLST	22241	273	0206	jejuni	no	yes	yes
Campylobacter jejuni OXC7094	PubMLST	22276	227	0206	jejuni	no	yes	yes
Campylobacter jejuni OXC7100	PubMLST	22282	273	0206	jejuni	no	yes	yes
Campylobacter jejuni OXC7108	PubMLST	21190	572	0206	jejuni	no	yes	yes
Campylobacter jejuni OXC7109	PubMLST	21191	572	0206	jejuni	no	yes	yes
Campylobacter jejuni OXC7117	PubMLST	21199	227	0206	jejuni	no	yes	yes
Campylobacter jejuni OXC7159	PubMLST	21240	122	0206	jejuni	no	yes	yes
Campylobacter jejuni OXC7161	PubMLST	21242	572	0206	jejuni	no	yes	yes
Campylobacter jejuni OXC7182	PubMLST	22673	122	0206	jejuni	no	yes	yes
Campylobacter jejuni OXC7193	PubMLST	22684	122	0206	jejuni	no	yes	yes
Campylobacter jejuni OXC7251	PubMLST	22293	122	0206	jejuni	no	yes	yes
Campylobacter jejuni OXC7287	PubMLST	22328	572	0206	jejuni	no	yes	yes
Campylobacter jejuni OXC7289	PubMLST	22330	122	0206	jejuni	no	yes	yes
Campylobacter jejuni OXC7292	PubMLST	22333	122	0206	jejuni	no	yes	yes
Campylobacter jejuni OXC7310	PubMLST	22351	273	0206	jejuni	no	yes	yes
Campylobacter jejuni OXC7334	PubMLST	22372	122	0206	jejuni	no	yes	yes
Campylobacter jejuni OXC7355	PubMLST	23894	122	0206	jejuni	no	yes	yes
Campylobacter jejuni OXC7441	PubMLST	23972	227	0206	jejuni	no	yes	yes
Campylobacter jejuni OXC7509	PubMLST	24035	122	0206	jejuni	no	yes	yes
Campylobacter jejuni OXC7532	PubMLST	24475	122	0206	jejuni	no	yes	yes
Campylobacter jejuni OXC7560	PubMLST	24497	206	0206	jejuni	no	yes	yes
Campylobacter jejuni OXC7588	PubMLST	24522	273	0206	jejuni	no	yes	yes
Campylobacter jejuni OXC7599	PubMLST	24531	122	0206	jejuni	no	yes	yes
Campylobacter jejuni OXC7620	PubMLST	24549	227	0206	jejuni	no	yes	yes
Campylobacter jejuni OXC7621	PubMLST	24550	122	0206	jejuni	no	yes	yes
Campylobacter jejuni OXC7668	PubMLST	24923	273	0206	jejuni	no	yes	yes
Campylobacter jejuni OXC7678	PubMLST	24932	122	0206	jejuni	no	yes	yes
Campylobacter jejuni OXC7704	PubMLST	24951	206	0206	jejuni	no	yes	yes
Campylobacter jejuni OXC7717	PubMLST	24959	122	0206	jejuni	no	yes	yes
Campylobacter jejuni OXC7724	PubMLST	24965	273	0206	jejuni	no	yes	yes
Campylobacter jejuni OXC7727	PubMLST	24968	122	0206	jejuni	no	yes	yes
Campylobacter jejuni OXC7791	PubMLST	24594	122	0206	jejuni	no	yes	yes
Campylobacter jejuni OXC7800	PubMLST	24601	273	0206	jejuni	no	yes	yes
Campylobacter jejuni OXC7802	PubMLST	24602	572	0206	jejuni	no	yes	yes
Campylobacter jejuni OXC7814	PubMLST	24612	572	0206	jejuni	no	yes	yes
Campylobacter jejuni OXC7822	PubMLST	24619	122	0206	jejuni	no	yes	yes
Campylobacter jejuni OXC7869	PubMLST	25004	227	0206	jejuni	no	yes	yes
Campylobacter jejuni OXC7872	PubMLST	25007	122	0206	jejuni	no	yes	yes
Campylobacter jejuni OXC7899	PubMLST	25030	206	0206	jejuni	no	yes	yes
Campylobacter jejuni OXC7903	PubMLST	25034	273	0206	jejuni	no	yes	yes
Campylobacter jejuni OXC7918	PubMLST	25048	122	0206	jejuni	no	yes	yes
Campylobacter jejuni OXC7950	PubMLST	25570	122	0206	jejuni	no	yes	yes
Campylobacter jejuni OXC7968	PubMLST	25383	206	0206	jejuni	no	yes	yes
Campylobacter jejuni OXC7971	PubMLST	25386	572	0206	jejuni	no	yes	yes
Campylobacter jejuni OXC7986	PubMLST	25398	122	0206	jejuni	no	yes	yes
Campylobacter jejuni OXC7993	PubMLST	25402	122	0206	jejuni	no	yes	yes
Campylobacter jejuni OXC7996	PubMLST	25485	122	0206	jejuni	no	yes	yes
Campylobacter jejuni OXC8002	PubMLST	25487	206	0206	jejuni	no	yes	yes
Campylobacter jejuni OXC8006	PubMLST	25490	273	0206	jejuni	no	yes	yes
Campylobacter jejuni OXC8010	PubMLST	25492	2149	0206	jejuni	no	yes	yes
Campylobacter jejuni OXC8011	PubMLST	25581	122	0206	jejuni	no	yes	yes
Campylobacter jejuni OXC8027	PubMLST	25497	2149	0206	jejuni	no	yes	yes
Campylobacter jejuni OXC8029	PubMLST	25498	206	0206	jejuni	no	yes	yes
Campylobacter jejuni OXC8045	PubMLST	25594	206	0206	jejuni	no	yes	yes
Campylobacter jejuni OXC8050	PubMLST	25596	273	0206	jejuni	no	yes	yes
Campylobacter jejuni OXC8051	PubMLST	28559	206	0206	jejuni	no	yes	yes
Campylobacter jejuni OXC8078	PubMLST	25518	6893	0206	jejuni	no	yes	yes
Campylobacter jejuni OXC8090	PubMLST	25523	572	0206	jejuni	no	yes	yes
Campylobacter jejuni OXC8092	PubMLST	25610	122	0206	jejuni	no	yes	yes
Campylobacter jejuni OXC8094	PubMLST	25427	122	0206	jejuni	no	yes	yes
Campylobacter jejuni OXC8134	PubMLST	25463	122	0206	jejuni	no	yes	yes
Campylobacter jejuni OXC8141	PubMLST	25445	206	0206	jejuni	no	yes	yes
Campylobacter jejuni OXC8143	PubMLST	25632	122	0206	jejuni	no	yes	yes
Campylobacter jejuni OXC8147	PubMLST	25635	206	0206	jejuni	no	yes	yes
Campylobacter jejuni OXC8151	PubMLST	25436	227	0206	jejuni	no	yes	yes
Campylobacter jejuni OXC8177	PubMLST	25448	122	0206	jejuni	no	yes	yes
Campylobacter jejuni OXC8225	PubMLST	27927	572	0206	jejuni	no	yes	yes
Campylobacter jejuni OXC8247	PubMLST	27946	122	0206	jejuni	no	yes	yes
Campylobacter jejuni OXC8284	PubMLST	27976	227	0206	jejuni	no	yes	yes
Campylobacter jejuni OXC8307	PubMLST	27998	122	0206	jejuni	no	yes	yes
Campylobacter jejuni OXC8345	PubMLST	28036	46	0206	jejuni	no	yes	yes
Campylobacter jejuni OXC8351	PubMLST	28041	122	0206	jejuni	no	yes	yes
Campylobacter jejuni OXC8354	PubMLST	28043	122	0206	jejuni	no	yes	yes
Campylobacter jejuni OXC8358	PubMLST	28046	46	0206	jejuni	no	yes	yes

Campylobacter jejuni OXC8360	PubMLST	28048	46	0206	jejuni	no	yes	yes
Campylobacter jejuni OXC8378	PubMLST	27876	273	0206	jejuni	no	yes	yes
Campylobacter jejuni OXC8399	PubMLST	28591	206	0206	jejuni	no	yes	yes
Campylobacter jejuni OXC8408	PubMLST	28599	122	0206	jejuni	no	yes	yes
Campylobacter jejuni OXC8412	PubMLST	28603	122	0206	jejuni	no	yes	yes
Campylobacter jejuni OXC8415	PubMLST	28606	273	0206	jejuni	no	yes	yes
Campylobacter jejuni OXC8452	PubMLST	28643	122	0206	jejuni	no	yes	yes
Campylobacter jejuni OXC8471	PubMLST	28664	122	0206	jejuni	no	yes	yes
Campylobacter jejuni OXC8480	PubMLST	28673	206	0206	jejuni	no	yes	yes
Campylobacter jejuni OXC8482	PubMLST	28675	122	0206	jejuni	no	yes	yes
Campylobacter jejuni OXC8538	PubMLST	28728	122	0206	jejuni	no	yes	yes
Campylobacter jejuni OXC8558	PubMLST	28747	122	0206	jejuni	no	yes	yes
Campylobacter jejuni OXC8566	PubMLST	28905	122	0206	jejuni	no	yes	yes
Campylobacter jejuni OXC8570	PubMLST	28909	122	0206	jejuni	no	yes	yes
Campylobacter jejuni OXC8590	PubMLST	28924	122	0206	jejuni	no	yes	yes
Campylobacter jejuni OXC8594	PubMLST	28927	122	0206	jejuni	no	yes	yes
Campylobacter jejuni OXC8629	PubMLST	28992	122	0206	jejuni	no	yes	yes
Campylobacter jejuni OXC8647	PubMLST	28961	122	0206	jejuni	no	yes	yes
Campylobacter jejuni OXC8650	PubMLST	28964	122	0206	jejuni	no	yes	yes
Campylobacter jejuni OXC8664	PubMLST	28999	206	0206	jejuni	no	yes	yes
Campylobacter jejuni OXC8670	PubMLST	29003	122	0206	jejuni	no	yes	yes
Campylobacter jejuni OXC8677	PubMLST	29009	122	0206	jejuni	no	yes	yes
Campylobacter jejuni OXC8703	PubMLST	29032	122	0206	jejuni	no	yes	yes
Campylobacter jejuni OXC8707	PubMLST	29036	122	0206	jejuni	no	yes	yes
Campylobacter jejuni OXC8731	PubMLST	29056	206	0206	jejuni	no	yes	yes
Campylobacter jejuni OXC8754	PubMLST	30445	273	0206	jejuni	no	yes	yes
Campylobacter jejuni OXC8801	PubMLST	30490	122	0206	jejuni	no	yes	yes
Campylobacter jejuni OXC8803	PubMLST	30492	122	0206	jejuni	no	yes	yes
Campylobacter jejuni OXC8832	PubMLST	30520	122	0206	jejuni	no	yes	yes
Campylobacter jejuni OXC8834	PubMLST	30522	122	0206	jejuni	no	yes	yes
Campylobacter jejuni OXC8835	PubMLST	30523	572	0206	jejuni	no	yes	yes
Campylobacter jejuni OXC8853	PubMLST	30539	227	0206	jejuni	no	yes	yes
Campylobacter jejuni OXC8856	PubMLST	30542	122	0206	jejuni	no	yes	yes
Campylobacter jejuni OXC8858	PubMLST	30544	122	0206	jejuni	no	yes	yes
Campylobacter jejuni OXC8871	PubMLST	30557	2149	0206	jejuni	no	yes	yes
Campylobacter jejuni OXC8882	PubMLST	30432	122	0206	jejuni	no	yes	yes
Campylobacter jejuni OXC8913	PubMLST	30596	122	0206	jejuni	no	yes	yes
Campylobacter jejuni OXC8917	PubMLST	30600	122	0206	jejuni	no	yes	yes
Campylobacter jejuni OXC8918	PubMLST	30601	122	0206	jejuni	no	yes	yes
Campylobacter jejuni OXC8928	PubMLST	30611	122	0206	jejuni	no	yes	yes
Campylobacter jejuni OXC8932	PubMLST	30615	122	0206	jejuni	no	yes	yes
Campylobacter jejuni OXC8938	PubMLST	30621	122	0206	jejuni	no	yes	yes
Campylobacter jejuni OXC8945	PubMLST	30628	273	0206	jejuni	no	yes	yes
Campylobacter jejuni OXC8950	PubMLST	30633	206	0206	jejuni	no	yes	yes
Campylobacter jejuni OXC8953	PubMLST	30636	206	0206	jejuni	no	yes	yes
Campylobacter jejuni 48321	PubMLST	24252	257	0257	jejuni	no	yes	yes
Campylobacter jejuni C120381	PubMLST	28868	257	0257	jejuni	no	yes	yes
Campylobacter jejuni C120401	PubMLST	28869	257	0257	jejuni	no	yes	yes
Campylobacter jejuni C120408	PubMLST	28870	257	0257	jejuni	no	yes	yes
Campylobacter jejuni C120453	PubMLST	28871	257	0257	jejuni	no	yes	yes
Campylobacter jejuni C120553	PubMLST	28873	257	0257	jejuni	no	yes	yes
Campylobacter jejuni CJ7257_R	PubMLST	24345	257	0257	jejuni	no	yes	yes
Campylobacter jejuni Dg272	PubMLST	25952	990	0257	jejuni	no	yes	yes
Campylobacter jejuni Dg287	PubMLST	25956	990	0257	jejuni	no	yes	yes
Campylobacter jejuni H122620624	PubMLST	2642	990	0257	jejuni	no	yes	yes
Campylobacter jejuni H122860616	PubMLST	2647	257	0257	jejuni	no	yes	yes
Campylobacter jejuni H143140404	PubMLST	2608	257	0257	jejuni	no	yes	yes
Campylobacter jejuni HM115713	PubMLST	24200	2030	0257	jejuni	no	yes	yes
Campylobacter jejuni OXC4570	PubMLST	24047	257	0257	jejuni	no	yes	yes
Campylobacter jejuni OXC4577	PubMLST	24051	990	0257	jejuni	no	yes	yes
Campylobacter jejuni OXC4587	PubMLST	24059	990	0257	jejuni	no	yes	yes
Campylobacter jejuni OXC4593	PubMLST	24063	257	0257	jejuni	no	yes	yes
Campylobacter jejuni OXC4608	PubMLST	22128	257	0257	jejuni	no	yes	yes
Campylobacter jejuni OXC4620	PubMLST	24073	257	0257	jejuni	no	yes	yes
Campylobacter jejuni OXC4637	PubMLST	25656	2030	0257	jejuni	no	yes	yes
Campylobacter jejuni OXC4657	PubMLST	24099	257	0257	jejuni	no	yes	yes
Campylobacter jejuni OXC4672	PubMLST	25089	257	0257	jejuni	no	yes	yes
Campylobacter jejuni OXC4677	PubMLST	24113	257	0257	jejuni	no	yes	yes
Campylobacter jejuni OXC4678	PubMLST	24114	257	0257	jejuni	no	yes	yes
Campylobacter jejuni OXC4738	PubMLST	18441	2030	0257	jejuni	no	yes	yes
Campylobacter jejuni OXC4740	PubMLST	18442	257	0257	jejuni	no	yes	yes
Campylobacter jejuni OXC4760	PubMLST	18455	257	0257	jejuni	no	yes	yes
Campylobacter jejuni OXC4774	PubMLST	18464	257	0257	jejuni	no	yes	yes
Campylobacter jejuni OXC4822	PubMLST	22152	257	0257	jejuni	no	yes	yes
Campylobacter jejuni OXC4858	PubMLST	22170	257	0257	jejuni	no	yes	yes
Campylobacter jejuni OXC4869	PubMLST	22178	2030	0257	jejuni	no	yes	yes
Campylobacter jejuni OXC4878	PubMLST	22183	257	0257	jejuni	no	yes	yes
Campylobacter jejuni OXC4899	PubMLST	24461	257	0257	jejuni	no	yes	yes
Campylobacter jejuni OXC4899_R	PubMLST	25657	257	0257	jejuni	no	yes	yes
Campylobacter jejuni OXC4923	PubMLST	25090	257	0257	jejuni	no	yes	yes
Campylobacter jejuni OXC4945	PubMLST	25094	2030	0257	jejuni	no	yes	yes
Campylobacter jejuni OXC4950	PubMLST	24473	257	0257	jejuni	no	yes	yes
Campylobacter jejuni OXC4956	PubMLST	25096	257	0257	jejuni	no	yes	yes
Campylobacter jejuni OXC4963	PubMLST	25565	257	0257	jejuni	no	yes	yes
Campylobacter jejuni OXC5000	PubMLST	24677	2030	0257	jejuni	no	yes	yes
Campylobacter jejuni OXC5001	PubMLST	24678	257	0257	jejuni	no	yes	yes
Campylobacter jejuni OXC5009	PubMLST	24683	2030	0257	jejuni	no	yes	yes
Campylobacter jejuni OXC5015	PubMLST	24688	2030	0257	jejuni	no	yes	yes
Campylobacter jejuni OXC5022	PubMLST	24691	257	0257	jejuni	no	yes	yes
Campylobacter jejuni OXC5023	PubMLST	24692	257	0257	jejuni	no	yes	yes
Campylobacter jejuni OXC5025	PubMLST	24693	2030	0257	jejuni	no	yes	yes
Campylobacter jejuni OXC5027	PubMLST	24695	2030	0257	jejuni	no	yes	yes

Campylobacter jejuni OXC5030	PubMLST	24698	257	0257	jejuni	no	yes	yes
Campylobacter jejuni OXC5041	PubMLST	24706	2030	0257	jejuni	no	yes	yes
Campylobacter jejuni OXC5052	PubMLST	24713	2030	0257	jejuni	no	yes	yes
Campylobacter jejuni OXC5054	PubMLST	24714	2030	0257	jejuni	no	yes	yes
Campylobacter jejuni OXC5058	PubMLST	24717	257	0257	jejuni	no	yes	yes
Campylobacter jejuni OXC5097	PubMLST	24734	257	0257	jejuni	no	yes	yes
Campylobacter jejuni OXC5109	PubMLST	24737	2030	0257	jejuni	no	yes	yes
Campylobacter jejuni OXC5122	PubMLST	24745	257	0257	jejuni	no	yes	yes
Campylobacter jejuni OXC5182	PubMLST	24778	257	0257	jejuni	no	yes	yes
Campylobacter jejuni OXC5187	PubMLST	24782	257	0257	jejuni	no	yes	yes
Campylobacter jejuni OXC5209	PubMLST	24799	257	0257	jejuni	no	yes	yes
Campylobacter jejuni OXC5285	PubMLST	24843	990	0257	jejuni	no	yes	yes
Campylobacter jejuni OXC5315	PubMLST	25098	2030	0257	jejuni	no	yes	yes
Campylobacter jejuni OXC5376	PubMLST	21342	257	0257	jejuni	no	yes	yes
Campylobacter jejuni OXC5390	PubMLST	21350	257	0257	jejuni	no	yes	yes
Campylobacter jejuni OXC5395	PubMLST	21355	257	0257	jejuni	no	yes	yes
Campylobacter jejuni OXC5398	PubMLST	21358	257	0257	jejuni	no	yes	yes
Campylobacter jejuni OXC5410	PubMLST	21364	257	0257	jejuni	no	yes	yes
Campylobacter jejuni OXC5418	PubMLST	21371	990	0257	jejuni	no	yes	yes
Campylobacter jejuni OXC5421	PubMLST	21373	257	0257	jejuni	no	yes	yes
Campylobacter jejuni OXC5497	PubMLST	23498	257	0257	jejuni	no	yes	yes
Campylobacter jejuni OXC5632	PubMLST	23518	257	0257	jejuni	no	yes	yes
Campylobacter jejuni OXC5641	PubMLST	23526	2030	0257	jejuni	no	yes	yes
Campylobacter jejuni OXC5643	PubMLST	23528	257	0257	jejuni	no	yes	yes
Campylobacter jejuni OXC5703	PubMLST	21442	257	0257	jejuni	no	yes	yes
Campylobacter jejuni OXC5709	PubMLST	21446	2030	0257	jejuni	no	yes	yes
Campylobacter jejuni OXC5779	PubMLST	21499	257	0257	jejuni	no	yes	yes
Campylobacter jejuni OXC5781	PubMLST	21501	257	0257	jejuni	no	yes	yes
Campylobacter jejuni OXC5788	PubMLST	23540	257	0257	jejuni	no	yes	yes
Campylobacter jejuni OXC5794	PubMLST	23659	2030	0257	jejuni	no	yes	yes
Campylobacter jejuni OXC5817	PubMLST	23565	257	0257	jejuni	no	yes	yes
Campylobacter jejuni OXC5820	PubMLST	23568	990	0257	jejuni	no	yes	yes
Campylobacter jejuni OXC5833	PubMLST	23665	6603	0257	jejuni	no	yes	yes
Campylobacter jejuni OXC5859	PubMLST	23599	2030	0257	jejuni	no	yes	yes
Campylobacter jejuni OXC5865	PubMLST	23603	2030	0257	jejuni	no	yes	yes
Campylobacter jejuni OXC5866	PubMLST	23604	257	0257	jejuni	no	yes	yes
Campylobacter jejuni OXC5869	PubMLST	23605	2030	0257	jejuni	no	yes	yes
Campylobacter jejuni OXC5872	PubMLST	23607	257	0257	jejuni	no	yes	yes
Campylobacter jejuni OXC5883	PubMLST	23674	6604	0257	jejuni	no	yes	yes
Campylobacter jejuni OXC5885	PubMLST	23616	257	0257	jejuni	no	yes	yes
Campylobacter jejuni OXC5889	PubMLST	23620	2030	0257	jejuni	no	yes	yes
Campylobacter jejuni OXC5903	PubMLST	23630	257	0257	jejuni	no	yes	yes
Campylobacter jejuni OXC5907	PubMLST	23633	257	0257	jejuni	no	yes	yes
Campylobacter jejuni OXC5915	PubMLST	23640	257	0257	jejuni	no	yes	yes
Campylobacter jejuni OXC5916	PubMLST	23641	990	0257	jejuni	no	yes	yes
Campylobacter jejuni OXC5932	PubMLST	23653	2030	0257	jejuni	no	yes	yes
Campylobacter jejuni OXC6250	PubMLST	16048	257	0257	jejuni	no	yes	yes
Campylobacter jejuni OXC6254	PubMLST	16052	257	0257	jejuni	no	yes	yes
Campylobacter jejuni OXC6290	PubMLST	16087	257	0257	jejuni	no	yes	yes
Campylobacter jejuni OXC6291	PubMLST	16088	2030	0257	jejuni	no	yes	yes
Campylobacter jejuni OXC6344	PubMLST	16141	257	0257	jejuni	no	yes	yes
Campylobacter jejuni OXC6362	PubMLST	16159	257	0257	jejuni	no	yes	yes
Campylobacter jejuni OXC6375	PubMLST	16172	257	0257	jejuni	no	yes	yes
Campylobacter jejuni OXC6390	PubMLST	16187	257	0257	jejuni	no	yes	yes
Campylobacter jejuni OXC6401	PubMLST	16196	257	0257	jejuni	no	yes	yes
Campylobacter jejuni OXC6404	PubMLST	16199	2030	0257	jejuni	no	yes	yes
Campylobacter jejuni OXC6407	PubMLST	16202	257	0257	jejuni	no	yes	yes
Campylobacter jejuni OXC6412	PubMLST	16207	257	0257	jejuni	no	yes	yes
Campylobacter jejuni OXC6450	PubMLST	16243	257	0257	jejuni	no	yes	yes
Campylobacter jejuni OXC6467	PubMLST	16259	2030	0257	jejuni	no	yes	yes
Campylobacter jejuni OXC6469	PubMLST	16261	2030	0257	jejuni	no	yes	yes
Campylobacter jejuni OXC6491	PubMLST	16283	257	0257	jejuni	no	yes	yes
Campylobacter jejuni OXC6499	PubMLST	16291	2030	0257	jejuni	no	yes	yes
Campylobacter jejuni OXC6526	PubMLST	16318	990	0257	jejuni	no	yes	yes
Campylobacter jejuni OXC6582	PubMLST	12919	2030	0257	jejuni	no	yes	yes
Campylobacter jejuni OXC6583	PubMLST	12920	257	0257	jejuni	no	yes	yes
Campylobacter jejuni OXC6612	PubMLST	16362	257	0257	jejuni	no	yes	yes
Campylobacter jejuni OXC6650	PubMLST	18207	257	0257	jejuni	no	yes	yes
Campylobacter jejuni OXC6654	PubMLST	18211	257	0257	jejuni	no	yes	yes
Campylobacter jejuni OXC6696	PubMLST	18253	257	0257	jejuni	no	yes	yes
Campylobacter jejuni OXC6709	PubMLST	18266	2030	0257	jejuni	no	yes	yes
Campylobacter jejuni OXC6715	PubMLST	18272	257	0257	jejuni	no	yes	yes
Campylobacter jejuni OXC6724	PubMLST	18281	257	0257	jejuni	no	yes	yes
Campylobacter jejuni OXC6736	PubMLST	18293	257	0257	jejuni	no	yes	yes
Campylobacter jejuni OXC6747	PubMLST	18304	990	0257	jejuni	no	yes	yes
Campylobacter jejuni OXC6749	PubMLST	18306	2030	0257	jejuni	no	yes	yes
Campylobacter jejuni OXC6750	PubMLST	18307	824	0257	jejuni	no	yes	yes
Campylobacter jejuni OXC6760	PubMLST	18317	257	0257	jejuni	no	yes	yes
Campylobacter jejuni OXC6772	PubMLST	18329	257	0257	jejuni	no	yes	yes
Campylobacter jejuni OXC6783	PubMLST	18340	2030	0257	jejuni	no	yes	yes
Campylobacter jejuni OXC6784	PubMLST	18341	2030	0257	jejuni	no	yes	yes
Campylobacter jejuni OXC6792	PubMLST	18349	2030	0257	jejuni	no	yes	yes
Campylobacter jejuni OXC6816	PubMLST	18373	257	0257	jejuni	no	yes	yes
Campylobacter jejuni OXC6863	PubMLST	21582	257	0257	jejuni	no	yes	yes
Campylobacter jejuni OXC6880	PubMLST	21599	257	0257	jejuni	no	yes	yes
Campylobacter jejuni OXC6893	PubMLST	22088	257	0257	jejuni	no	yes	yes
Campylobacter jejuni OXC6967	PubMLST	21141	257	0257	jejuni	no	yes	yes
Campylobacter jejuni OXC6974	PubMLST	21147	257	0257	jejuni	no	yes	yes
Campylobacter jejuni OXC6975	PubMLST	21148	257	0257	jejuni	no	yes	yes
Campylobacter jejuni OXC6982	PubMLST	21155	257	0257	jejuni	no	yes	yes
Campylobacter jejuni OXC6989	PubMLST	21162	2030	0257	jejuni	no	yes	yes
Campylobacter jejuni OXC6999	PubMLST	21170	257	0257	jejuni	no	yes	yes

Campylobacter jejuni OXC7034	PubMLST	22220	257	0257	jejuni	no	yes	yes
Campylobacter jejuni OXC7096	PubMLST	22278	257	0257	jejuni	no	yes	yes
Campylobacter jejuni OXC7106	PubMLST	21188	257	0257	jejuni	no	yes	yes
Campylobacter jejuni OXC7120	PubMLST	21202	257	0257	jejuni	no	yes	yes
Campylobacter jejuni OXC7138	PubMLST	21219	257	0257	jejuni	no	yes	yes
Campylobacter jejuni OXC7146	PubMLST	21227	257	0257	jejuni	no	yes	yes
Campylobacter jejuni OXC7196	PubMLST	22687	257	0257	jejuni	no	yes	yes
Campylobacter jejuni OXC7245	PubMLST	22287	2030	0257	jejuni	no	yes	yes
Campylobacter jejuni OXC7257	PubMLST	22298	257	0257	jejuni	no	yes	yes
Campylobacter jejuni OXC7268	PubMLST	22309	257	0257	jejuni	no	yes	yes
Campylobacter jejuni OXC7291	PubMLST	22332	257	0257	jejuni	no	yes	yes
Campylobacter jejuni OXC7296	PubMLST	22337	257	0257	jejuni	no	yes	yes
Campylobacter jejuni OXC7308	PubMLST	22349	257	0257	jejuni	no	yes	yes
Campylobacter jejuni OXC7338	PubMLST	22375	257	0257	jejuni	no	yes	yes
Campylobacter jejuni OXC7354	PubMLST	23893	257	0257	jejuni	no	yes	yes
Campylobacter jejuni OXC7357	PubMLST	23896	257	0257	jejuni	no	yes	yes
Campylobacter jejuni OXC7390	PubMLST	23928	2030	0257	jejuni	no	yes	yes
Campylobacter jejuni OXC7410	PubMLST	23945	257	0257	jejuni	no	yes	yes
Campylobacter jejuni OXC7424	PubMLST	23958	257	0257	jejuni	no	yes	yes
Campylobacter jejuni OXC7442	PubMLST	23973	990	0257	jejuni	no	yes	yes
Campylobacter jejuni OXC7451	PubMLST	23981	990	0257	jejuni	no	yes	yes
Campylobacter jejuni OXC7507	PubMLST	24033	257	0257	jejuni	no	yes	yes
Campylobacter jejuni OXC7533	PubMLST	24476	824	0257	jejuni	no	yes	yes
Campylobacter jejuni OXC7566	PubMLST	24503	2030	0257	jejuni	no	yes	yes
Campylobacter jejuni OXC7585	PubMLST	24519	2030	0257	jejuni	no	yes	yes
Campylobacter jejuni OXC7601	PubMLST	24533	257	0257	jejuni	no	yes	yes
Campylobacter jejuni OXC7604	PubMLST	24535	2030	0257	jejuni	no	yes	yes
Campylobacter jejuni OXC7625	PubMLST	24554	257	0257	jejuni	no	yes	yes
Campylobacter jejuni OXC7655	PubMLST	24910	257	0257	jejuni	no	yes	yes
Campylobacter jejuni OXC7665	PubMLST	24920	257	0257	jejuni	no	yes	yes
Campylobacter jejuni OXC7674	PubMLST	24928	990	0257	jejuni	no	yes	yes
Campylobacter jejuni OXC7707	PubMLST	24954	2030	0257	jejuni	no	yes	yes
Campylobacter jejuni OXC7737	PubMLST	27880	257	0257	jejuni	no	yes	yes
Campylobacter jejuni OXC7738	PubMLST	24975	2030	0257	jejuni	no	yes	yes
Campylobacter jejuni OXC7754	PubMLST	27870	2030	0257	jejuni	no	yes	yes
Campylobacter jejuni OXC7756	PubMLST	24570	2030	0257	jejuni	no	yes	yes
Campylobacter jejuni OXC7761	PubMLST	24572	257	0257	jejuni	no	yes	yes
Campylobacter jejuni OXC7767	PubMLST	24576	2030	0257	jejuni	no	yes	yes
Campylobacter jejuni OXC7777	PubMLST	24584	257	0257	jejuni	no	yes	yes
Campylobacter jejuni OXC7781	PubMLST	24588	990	0257	jejuni	no	yes	yes
Campylobacter jejuni OXC7785	PubMLST	25455	990	0257	jejuni	no	yes	yes
Campylobacter jejuni OXC7787	PubMLST	27882	990	0257	jejuni	no	yes	yes
Campylobacter jejuni OXC7792	PubMLST	24595	2030	0257	jejuni	no	yes	yes
Campylobacter jejuni OXC7796	PubMLST	24597	257	0257	jejuni	no	yes	yes
Campylobacter jejuni OXC7826	PubMLST	24623	257	0257	jejuni	no	yes	yes
Campylobacter jejuni OXC7828	PubMLST	24625	257	0257	jejuni	no	yes	yes
Campylobacter jejuni OXC7844	PubMLST	24641	257	0257	jejuni	no	yes	yes
Campylobacter jejuni OXC7846	PubMLST	24643	990	0257	jejuni	no	yes	yes
Campylobacter jejuni OXC7850	PubMLST	24647	257	0257	jejuni	no	yes	yes
Campylobacter jejuni OXC7853	PubMLST	24988	990	0257	jejuni	no	yes	yes
Campylobacter jejuni OXC7875	PubMLST	25010	2030	0257	jejuni	no	yes	yes
Campylobacter jejuni OXC7878	PubMLST	25113	2030	0257	jejuni	no	yes	yes
Campylobacter jejuni OXC7886	PubMLST	25115	990	0257	jejuni	no	yes	yes
Campylobacter jejuni OXC7889	PubMLST	25020	6890	0257	jejuni	no	yes	yes
Campylobacter jejuni OXC7894	PubMLST	25025	990	0257	jejuni	no	yes	yes
Campylobacter jejuni OXC7904	PubMLST	25035	990	0257	jejuni	no	yes	yes
Campylobacter jejuni OXC7909	PubMLST	25039	2030	0257	jejuni	no	yes	yes
Campylobacter jejuni OXC7937	PubMLST	25119	990	0257	jejuni	no	yes	yes
Campylobacter jejuni OXC7948	PubMLST	25652	257	0257	jejuni	no	yes	yes
Campylobacter jejuni OXC7962	PubMLST	25480	257	0257	jejuni	no	yes	yes
Campylobacter jejuni OXC7965	PubMLST	25574	257	0257	jejuni	no	yes	yes
Campylobacter jejuni OXC7974	PubMLST	25389	257	0257	jejuni	no	yes	yes
Campylobacter jejuni OXC7983	PubMLST	25576	257	0257	jejuni	no	yes	yes
Campylobacter jejuni OXC8000	PubMLST	25578	6890	0257	jejuni	no	yes	yes
Campylobacter jejuni OXC8030	PubMLST	25499	2030	0257	jejuni	no	yes	yes
Campylobacter jejuni OXC8083	PubMLST	25422	257	0257	jejuni	no	yes	yes
Campylobacter jejuni OXC8089	PubMLST	25426	257	0257	jejuni	no	yes	yes
Campylobacter jejuni OXC8104	PubMLST	25429	990	0257	jejuni	no	yes	yes
Campylobacter jejuni OXC8112	PubMLST	25617	2030	0257	jejuni	no	yes	yes
Campylobacter jejuni OXC8175	PubMLST	25642	257	0257	jejuni	no	yes	yes
Campylobacter jejuni OXC8214	PubMLST	27917	990	0257	jejuni	no	yes	yes
Campylobacter jejuni OXC8217	PubMLST	27920	257	0257	jejuni	no	yes	yes
Campylobacter jejuni OXC8222	PubMLST	27924	257	0257	jejuni	no	yes	yes
Campylobacter jejuni OXC8256	PubMLST	27954	257	0257	jejuni	no	yes	yes
Campylobacter jejuni OXC8370	PubMLST	28056	257	0257	jejuni	no	yes	yes
Campylobacter jejuni OXC8371	PubMLST	27871	257	0257	jejuni	no	yes	yes
Campylobacter jejuni OXC8429	PubMLST	28620	257	0257	jejuni	no	yes	yes
Campylobacter jejuni OXC8445	PubMLST	28636	2254	0257	jejuni	no	yes	yes
Campylobacter jejuni OXC8465	PubMLST	28658	2030	0257	jejuni	no	yes	yes
Campylobacter jejuni OXC8477	PubMLST	28670	2254	0257	jejuni	no	yes	yes
Campylobacter jejuni OXC8484	PubMLST	28677	257	0257	jejuni	no	yes	yes
Campylobacter jejuni OXC8493	PubMLST	28685	990	0257	jejuni	no	yes	yes
Campylobacter jejuni OXC8531	PubMLST	28721	257	0257	jejuni	no	yes	yes
Campylobacter jejuni OXC8537	PubMLST	28727	257	0257	jejuni	no	yes	yes
Campylobacter jejuni OXC8544	PubMLST	28733	2030	0257	jejuni	no	yes	yes
Campylobacter jejuni OXC8575	PubMLST	28912	257	0257	jejuni	no	yes	yes
Campylobacter jejuni OXC8597	PubMLST	28930	257	0257	jejuni	no	yes	yes
Campylobacter jejuni OXC8600	PubMLST	28932	990	0257	jejuni	no	yes	yes
Campylobacter jejuni OXC8602	PubMLST	28934	990	0257	jejuni	no	yes	yes
Campylobacter jejuni OXC8613	PubMLST	28941	2030	0257	jejuni	no	yes	yes
Campylobacter jejuni OXC8634	PubMLST	28952	257	0257	jejuni	no	yes	yes
Campylobacter jejuni OXC8635	PubMLST	28953	257	0257	jejuni	no	yes	yes

Campylobacter jejuni OXC8661	PubMLST	28996	257	0257	jejuni	no	yes	yes
Campylobacter jejuni OXC8672	PubMLST	29005	2030	0257	jejuni	no	yes	yes
Campylobacter jejuni OXC8683	PubMLST	29015	2254	0257	jejuni	no	yes	yes
Campylobacter jejuni OXC8689	PubMLST	29021	257	0257	jejuni	no	yes	yes
Campylobacter jejuni OXC8747	PubMLST	30438	257	0257	jejuni	no	yes	yes
Campylobacter jejuni OXC8757	PubMLST	30447	990	0257	jejuni	no	yes	yes
Campylobacter jejuni OXC8772	PubMLST	30461	286	0257	jejuni	no	yes	yes
Campylobacter jejuni OXC8786	PubMLST	30475	257	0257	jejuni	no	yes	yes
Campylobacter jejuni OXC8787	PubMLST	30476	990	0257	jejuni	no	yes	yes
Campylobacter jejuni OXC8797	PubMLST	30486	2254	0257	jejuni	no	yes	yes
Campylobacter jejuni OXC8825	PubMLST	30513	257	0257	jejuni	no	yes	yes
Campylobacter jejuni OXC8866	PubMLST	30552	257	0257	jejuni	no	yes	yes
Campylobacter jejuni OXC8909	PubMLST	30592	257	0257	jejuni	no	yes	yes
Campylobacter jejuni OXC8962	PubMLST	30644	257	0257	jejuni	no	yes	yes
Campylobacter jejuni OXC8979	PubMLST	30661	257	0257	jejuni	no	yes	yes
Campylobacter jejuni OXC9000	PubMLST	30681	257	0257	jejuni	no	yes	yes
Campylobacter jejuni OXC9001	PubMLST	30682	257	0257	jejuni	no	yes	yes
Campylobacter jejuni 81116	Genbank/EMBL/DDBJ	NC_009839	267	0283	jejuni	no	yes	yes
Campylobacter jejuni Dg198	PubMLST	26068	267	0283	jejuni	no	yes	yes
Campylobacter jejuni Dg66a	PubMLST	25958	267	0283	jejuni	no	yes	yes
Campylobacter jejuni OXC4683	PubMLST	24118	267	0283	jejuni	no	yes	yes
Campylobacter jejuni OXC4778	PubMLST	18468	267	0283	jejuni	no	yes	yes
Campylobacter jejuni OXC4933	PubMLST	24470	267	0283	jejuni	no	yes	yes
Campylobacter jejuni OXC5011	PubMLST	24685	267	0283	jejuni	no	yes	yes
Campylobacter jejuni OXC5014	PubMLST	24687	267	0283	jejuni	no	yes	yes
Campylobacter jejuni OXC5068	PubMLST	24722	267	0283	jejuni	no	yes	yes
Campylobacter jejuni OXC5908	PubMLST	23634	267	0283	jejuni	no	yes	yes
Campylobacter jejuni OXC6369	PubMLST	16166	267	0283	jejuni	no	yes	yes
Campylobacter jejuni OXC6373	PubMLST	16170	267	0283	jejuni	no	yes	yes
Campylobacter jejuni OXC6510	PubMLST	16302	267	0283	jejuni	no	yes	yes
Campylobacter jejuni OXC6511	PubMLST	16303	267	0283	jejuni	no	yes	yes
Campylobacter jejuni OXC6826	PubMLST	18383	267	0283	jejuni	no	yes	yes
Campylobacter jejuni OXC6884	PubMLST	22080	267	0283	jejuni	no	yes	yes
Campylobacter jejuni OXC7030	PubMLST	22216	267	0283	jejuni	no	yes	yes
Campylobacter jejuni OXC7053	PubMLST	22238	267	0283	jejuni	no	yes	yes
Campylobacter jejuni OXC7060	PubMLST	22244	267	0283	jejuni	no	yes	yes
Campylobacter jejuni OXC7086	PubMLST	22270	267	0283	jejuni	no	yes	yes
Campylobacter jejuni OXC7144	PubMLST	21225	267	0283	jejuni	no	yes	yes
Campylobacter jejuni OXC7152	PubMLST	21233	267	0283	jejuni	no	yes	yes
Campylobacter jejuni OXC7155	PubMLST	21236	267	0283	jejuni	no	yes	yes
Campylobacter jejuni OXC7198	PubMLST	22689	267	0283	jejuni	no	yes	yes
Campylobacter jejuni OXC7234	PubMLST	22724	267	0283	jejuni	no	yes	yes
Campylobacter jejuni OXC7255	PubMLST	22296	6234	0283	jejuni	no	yes	yes
Campylobacter jejuni OXC7278	PubMLST	22319	267	0283	jejuni	no	yes	yes
Campylobacter jejuni OXC7322	PubMLST	22361	267	0283	jejuni	no	yes	yes
Campylobacter jejuni OXC7391	PubMLST	23929	267	0283	jejuni	no	yes	yes
Campylobacter jejuni OXC7392	PubMLST	23930	267	0283	jejuni	no	yes	yes
Campylobacter jejuni OXC7393	PubMLST	23931	267	0283	jejuni	no	yes	yes
Campylobacter jejuni OXC7432	PubMLST	23965	267	0283	jejuni	no	yes	yes
Campylobacter jejuni OXC7551	PubMLST	24489	267	0283	jejuni	no	yes	yes
Campylobacter jejuni OXC7963	PubMLST	25379	267	0283	jejuni	no	yes	yes
Campylobacter jejuni OXC8073	PubMLST	25607	267	0283	jejuni	no	yes	yes
Campylobacter jejuni OXC8135	PubMLST	25653	267	0283	jejuni	no	yes	yes
Campylobacter jejuni OXC8136	PubMLST	27886	267	0283	jejuni	no	yes	yes
Campylobacter jejuni OXC8137	PubMLST	27887	267	0283	jejuni	no	yes	yes
Campylobacter jejuni OXC8292	PubMLST	27983	267	0283	jejuni	no	yes	yes
Campylobacter jejuni OXC8947	PubMLST	30630	267	0283	jejuni	no	yes	yes
Campylobacter jejuni OXC8988	PubMLST	30670	267	0283	jejuni	no	yes	yes
Campylobacter jejuni OXC8991	PubMLST	30673	267	0283	jejuni	no	yes	yes
Campylobacter jejuni OXC9003	PubMLST	30684	267	0283	jejuni	no	yes	yes
Campylobacter jejuni OXC9007	PubMLST	30687	267	0283	jejuni	no	yes	yes
Campylobacter jejuni OXC9008	PubMLST	30688	267	0283	jejuni	no	yes	yes
Campylobacter jejuni OXC9012	PubMLST	30692	267	0283	jejuni	no	yes	yes
Campylobacter jejuni OXC9013	PubMLST	30693	267	0283	jejuni	no	yes	yes
Campylobacter jejuni 1997-14	Genbank/EMBL/DDBJ	AIPA01	5159	0353	jejuni	no	yes	yes
Campylobacter jejuni 51037	Genbank/EMBL/DDBJ	AIPB01	939	0353	jejuni	no	yes	yes
Campylobacter jejuni 51494	Genbank/EMBL/DDBJ	AINZ01	4834	0353	jejuni	no	yes	yes
Campylobacter jejuni 53161	Genbank/EMBL/DDBJ	AION01	4838	0353	jejuni	no	yes	yes
Campylobacter jejuni 87459	Genbank/EMBL/DDBJ	AIPE01	452	0353	jejuni	no	yes	yes
Campylobacter jejuni H134660450	PubMLST	2663	524	0353	jejuni	no	yes	yes
Campylobacter jejuni LMG 23269	Genbank/EMBL/DDBJ	AIOG01	4837	0353	jejuni	no	yes	yes
Campylobacter jejuni OXC4573	PubMLST	24049	5	0353	jejuni	no	yes	yes
Campylobacter jejuni OXC4585	PubMLST	24058	353	0353	jejuni	no	yes	yes
Campylobacter jejuni OXC4634	PubMLST	24084	3515	0353	jejuni	no	yes	yes
Campylobacter jejuni OXC4664	PubMLST	24104	400	0353	jejuni	no	yes	yes
Campylobacter jejuni OXC4719	PubMLST	18429	2122	0353	jejuni	no	yes	yes
Campylobacter jejuni OXC4995	PubMLST	24676	5	0353	jejuni	no	yes	yes
Campylobacter jejuni OXC5070	PubMLST	25469	1474	0353	jejuni	no	yes	yes
Campylobacter jejuni OXC5071	PubMLST	24723	1474	0353	jejuni	no	yes	yes
Campylobacter jejuni OXC5226	PubMLST	24812	400	0353	jejuni	no	yes	yes
Campylobacter jejuni OXC5288	PubMLST	24845	5866	0353	jejuni	no	yes	yes
Campylobacter jejuni OXC5299	PubMLST	24847	2882	0353	jejuni	no	yes	yes
Campylobacter jejuni OXC5316	PubMLST	25474	7178	0353	jejuni	no	yes	yes
Campylobacter jejuni OXC5686	PubMLST	21430	400	0353	jejuni	no	yes	yes
Campylobacter jejuni OXC5750	PubMLST	21475	400	0353	jejuni	no	yes	yes
Campylobacter jejuni OXC5786	PubMLST	23538	2122	0353	jejuni	no	yes	yes
Campylobacter jejuni OXC5830	PubMLST	23576	400	0353	jejuni	no	yes	yes
Campylobacter jejuni OXC5911	PubMLST	23637	5247	0353	jejuni	no	yes	yes
Campylobacter jejuni OXC5931	PubMLST	23652	353	0353	jejuni	no	yes	yes
Campylobacter jejuni OXC5934	PubMLST	23655	2084	0353	jejuni	no	yes	yes
Campylobacter jejuni OXC6318	PubMLST	16115	356	0353	jejuni	no	yes	yes
Campylobacter jejuni OXC6455	PubMLST	16247	5730	0353	jejuni	no	yes	yes

Campylobacter jejuni OXC6456	PubMLST	16248	353	0353	jejuni	no	yes	yes
Campylobacter jejuni OXC6586	PubMLST	12923	400	0353	jejuni	no	yes	yes
Campylobacter jejuni OXC6611	PubMLST	16361	353	0353	jejuni	no	yes	yes
Campylobacter jejuni OXC6622	PubMLST	16371	595	0353	jejuni	no	yes	yes
Campylobacter jejuni OXC6658	PubMLST	18215	353	0353	jejuni	no	yes	yes
Campylobacter jejuni OXC6704	PubMLST	18261	356	0353	jejuni	no	yes	yes
Campylobacter jejuni OXC6776	PubMLST	18333	5815	0353	jejuni	no	yes	yes
Campylobacter jejuni OXC6806	PubMLST	18363	5816	0353	jejuni	no	yes	yes
Campylobacter jejuni OXC6851	PubMLST	21570	353	0353	jejuni	no	yes	yes
Campylobacter jejuni OXC7033	PubMLST	22219	356	0353	jejuni	no	yes	yes
Campylobacter jejuni OXC7226	PubMLST	22717	400	0353	jejuni	no	yes	yes
Campylobacter jejuni OXC7231	PubMLST	22721	400	0353	jejuni	no	yes	yes
Campylobacter jejuni OXC7271	PubMLST	22312	2036	0353	jejuni	no	yes	yes
Campylobacter jejuni OXC7380	PubMLST	23918	400	0353	jejuni	no	yes	yes
Campylobacter jejuni OXC7385	PubMLST	23923	6701	0353	jejuni	no	yes	yes
Campylobacter jejuni OXC7455	PubMLST	23985	353	0353	jejuni	no	yes	yes
Campylobacter jejuni OXC7516	PubMLST	24121	400	0353	jejuni	no	yes	yes
Campylobacter jejuni OXC7520	PubMLST	24124	353	0353	jejuni	no	yes	yes
Campylobacter jejuni OXC7586	PubMLST	24520	5	0353	jejuni	no	yes	yes
Campylobacter jejuni OXC7630	PubMLST	24559	353	0353	jejuni	no	yes	yes
Campylobacter jejuni OXC7646	PubMLST	24903	356	0353	jejuni	no	yes	yes
Campylobacter jejuni OXC7661	PubMLST	24916	6888	0353	jejuni	no	yes	yes
Campylobacter jejuni OXC7662	PubMLST	24917	3696	0353	jejuni	no	yes	yes
Campylobacter jejuni OXC7722	PubMLST	24964	353	0353	jejuni	no	yes	yes
Campylobacter jejuni OXC7830	PubMLST	24627	353	0353	jejuni	no	yes	yes
Campylobacter jejuni OXC8070	PubMLST	25516	2122	0353	jejuni	no	yes	yes
Campylobacter jejuni OXC8121	PubMLST	25539	6981	0353	jejuni	no	yes	yes
Campylobacter jejuni OXC8146	PubMLST	25634	2036	0353	jejuni	no	yes	yes
Campylobacter jejuni OXC8205	PubMLST	27910	356	0353	jejuni	no	yes	yes
Campylobacter jejuni OXC8249	PubMLST	27948	2122	0353	jejuni	no	yes	yes
Campylobacter jejuni OXC8287	PubMLST	27978	4697	0353	jejuni	no	yes	yes
Campylobacter jejuni OXC8295	PubMLST	27986	353	0353	jejuni	no	yes	yes
Campylobacter jejuni OXC8355	PubMLST	28044	2084	0353	jejuni	no	yes	yes
Campylobacter jejuni OXC8393	PubMLST	28586	353	0353	jejuni	no	yes	yes
Campylobacter jejuni OXC8486	PubMLST	28679	353	0353	jejuni	no	yes	yes
Campylobacter jejuni OXC8524	PubMLST	28715	353	0353	jejuni	no	yes	yes
Campylobacter jejuni OXC8554	PubMLST	28743	353	0353	jejuni	no	yes	yes
Campylobacter jejuni OXC8601	PubMLST	28933	353	0353	jejuni	no	yes	yes
Campylobacter jejuni OXC8641	PubMLST	28957	353	0353	jejuni	no	yes	yes
Campylobacter jejuni OXC8649	PubMLST	28963	400	0353	jejuni	no	yes	yes
Campylobacter jejuni OXC8723	PubMLST	29049	5011	0353	jejuni	no	yes	yes
Campylobacter jejuni OXC8736	PubMLST	29061	353	0353	jejuni	no	yes	yes
Campylobacter jejuni OXC8745	PubMLST	30436	400	0353	jejuni	no	yes	yes
Campylobacter jejuni OXC8767	PubMLST	30456	353	0353	jejuni	no	yes	yes
Campylobacter jejuni OXC8808	PubMLST	30497	400	0353	jejuni	no	yes	yes
Campylobacter jejuni OXC8895	PubMLST	30905	2122	0353	jejuni	no	yes	yes
Campylobacter jejuni OXC8898	PubMLST	30581	2122	0353	jejuni	no	yes	yes
Campylobacter jejuni OXC8898R	PubMLST	31005	2122	0353	jejuni	no	yes	yes
Campylobacter jejuni ARI_1433_R	PubMLST	24314	2033	0354	jejuni	no	yes	yes
Campylobacter jejuni BID17Q	PubMLST	25072	1038	0354	jejuni	no	yes	yes
Campylobacter jejuni C121803	PubMLST	28867	354	0354	jejuni	no	yes	yes
Campylobacter jejuni C130436	PubMLST	28866	354	0354	jejuni	no	yes	yes
Campylobacter jejuni C131242	PubMLST	28865	3155	0354	jejuni	no	yes	yes
Campylobacter jejuni C131405	PubMLST	28864	354	0354	jejuni	no	yes	yes
Campylobacter jejuni C131690	PubMLST	28863	3155	0354	jejuni	no	yes	yes
Campylobacter jejuni CJ6753_R	PubMLST	24307	354	0354	jejuni	no	yes	yes
Campylobacter jejuni H140940808	PubMLST	2672	354	0354	jejuni	no	yes	yes
Campylobacter jejuni NW	Genbank/EMBL/DDBJ	AGTE01	354	0354	jejuni	no	yes	yes
Campylobacter jejuni OXC4596	PubMLST	24065	354	0354	jejuni	no	yes	yes
Campylobacter jejuni OXC4598	PubMLST	24066	2033	0354	jejuni	no	yes	yes
Campylobacter jejuni OXC4641	PubMLST	24089	2033	0354	jejuni	no	yes	yes
Campylobacter jejuni OXC4655	PubMLST	24097	354	0354	jejuni	no	yes	yes
Campylobacter jejuni OXC4656	PubMLST	24098	354	0354	jejuni	no	yes	yes
Campylobacter jejuni OXC4741	PubMLST	18443	354	0354	jejuni	no	yes	yes
Campylobacter jejuni OXC4743	PubMLST	18445	354	0354	jejuni	no	yes	yes
Campylobacter jejuni OXC4857	PubMLST	22169	354	0354	jejuni	no	yes	yes
Campylobacter jejuni OXC4970	PubMLST	24661	354	0354	jejuni	no	yes	yes
Campylobacter jejuni OXC4971	PubMLST	24662	354	0354	jejuni	no	yes	yes
Campylobacter jejuni OXC5080	PubMLST	24724	354	0354	jejuni	no	yes	yes
Campylobacter jejuni OXC5087	PubMLST	24728	354	0354	jejuni	no	yes	yes
Campylobacter jejuni OXC5139	PubMLST	24758	1723	0354	jejuni	no	yes	yes
Campylobacter jejuni OXC5143	PubMLST	24759	1723	0354	jejuni	no	yes	yes
Campylobacter jejuni OXC5195	PubMLST	24789	354	0354	jejuni	no	yes	yes
Campylobacter jejuni OXC5262	PubMLST	24839	354	0354	jejuni	no	yes	yes
Campylobacter jejuni OXC5282	PubMLST	24842	354	0354	jejuni	no	yes	yes
Campylobacter jejuni OXC5604	PubMLST	23501	2033	0354	jejuni	no	yes	yes
Campylobacter jejuni OXC5627	PubMLST	21412	2033	0354	jejuni	no	yes	yes
Campylobacter jejuni OXC5673	PubMLST	21420	354	0354	jejuni	no	yes	yes
Campylobacter jejuni OXC5700	PubMLST	21439	354	0354	jejuni	no	yes	yes
Campylobacter jejuni OXC5797	PubMLST	23547	2033	0354	jejuni	no	yes	yes
Campylobacter jejuni OXC5802	PubMLST	23552	1038	0354	jejuni	no	yes	yes
Campylobacter jejuni OXC5847	PubMLST	23589	2033	0354	jejuni	no	yes	yes
Campylobacter jejuni OXC5927	PubMLST	23649	2033	0354	jejuni	no	yes	yes
Campylobacter jejuni OXC6132	PubMLST	29325	354	0354	jejuni	no	yes	yes
Campylobacter jejuni OXC6287	PubMLST	16084	5718	0354	jejuni	no	yes	yes
Campylobacter jejuni OXC6298	PubMLST	16095	354	0354	jejuni	no	yes	yes
Campylobacter jejuni OXC6299	PubMLST	16096	354	0354	jejuni	no	yes	yes
Campylobacter jejuni OXC6418	PubMLST	16213	354	0354	jejuni	no	yes	yes
Campylobacter jejuni OXC6451	PubMLST	16244	354	0354	jejuni	no	yes	yes
Campylobacter jejuni OXC6517	PubMLST	16309	354	0354	jejuni	no	yes	yes
Campylobacter jejuni OXC6518	PubMLST	16310	354	0354	jejuni	no	yes	yes
Campylobacter jejuni OXC6529	PubMLST	16321	354	0354	jejuni	no	yes	yes

Campylobacter jejuni OXC6567	PubMLST	12902	354	0354	jejuni	no	yes	yes
Campylobacter jejuni OXC6647	PubMLST	18204	354	0354	jejuni	no	yes	yes
Campylobacter jejuni OXC6698	PubMLST	18255	354	0354	jejuni	no	yes	yes
Campylobacter jejuni OXC6716	PubMLST	18273	354	0354	jejuni	no	yes	yes
Campylobacter jejuni OXC6717	PubMLST	18274	354	0354	jejuni	no	yes	yes
Campylobacter jejuni OXC6729	PubMLST	18286	1517	0354	jejuni	no	yes	yes
Campylobacter jejuni OXC6743	PubMLST	18300	354	0354	jejuni	no	yes	yes
Campylobacter jejuni OXC6753	PubMLST	18310	354	0354	jejuni	no	yes	yes
Campylobacter jejuni OXC6802	PubMLST	18359	2863	0354	jejuni	no	yes	yes
Campylobacter jejuni OXC6846	PubMLST	21565	969	0354	jejuni	no	yes	yes
Campylobacter jejuni OXC6859	PubMLST	21578	354	0354	jejuni	no	yes	yes
Campylobacter jejuni OXC6877	PubMLST	21596	354	0354	jejuni	no	yes	yes
Campylobacter jejuni OXC6885	PubMLST	22081	354	0354	jejuni	no	yes	yes
Campylobacter jejuni OXC6915	PubMLST	22110	354	0354	jejuni	no	yes	yes
Campylobacter jejuni OXC6934	PubMLST	21108	2033	0354	jejuni	no	yes	yes
Campylobacter jejuni OXC6991	PubMLST	21163	1038	0354	jejuni	no	yes	yes
Campylobacter jejuni OXC7274	PubMLST	22315	354	0354	jejuni	no	yes	yes
Campylobacter jejuni OXC7407	PubMLST	23942	354	0354	jejuni	no	yes	yes
Campylobacter jejuni OXC7414	PubMLST	23948	354	0354	jejuni	no	yes	yes
Campylobacter jejuni OXC7439	PubMLST	23970	354	0354	jejuni	no	yes	yes
Campylobacter jejuni OXC7471	PubMLST	24000	354	0354	jejuni	no	yes	yes
Campylobacter jejuni OXC7583	PubMLST	24517	354	0354	jejuni	no	yes	yes
Campylobacter jejuni OXC7628	PubMLST	24557	354	0354	jejuni	no	yes	yes
Campylobacter jejuni OXC7638	PubMLST	24567	354	0354	jejuni	no	yes	yes
Campylobacter jejuni OXC7664	PubMLST	24919	1517	0354	jejuni	no	yes	yes
Campylobacter jejuni OXC7679	PubMLST	24933	1038	0354	jejuni	no	yes	yes
Campylobacter jejuni OXC7741	PubMLST	24976	354	0354	jejuni	no	yes	yes
Campylobacter jejuni OXC7742	PubMLST	24977	354	0354	jejuni	no	yes	yes
Campylobacter jejuni OXC7765	PubMLST	24574	354	0354	jejuni	no	yes	yes
Campylobacter jejuni OXC7819	PubMLST	24616	354	0354	jejuni	no	yes	yes
Campylobacter jejuni OXC7843	PubMLST	24640	354	0354	jejuni	no	yes	yes
Campylobacter jejuni OXC7849	PubMLST	24646	354	0354	jejuni	no	yes	yes
Campylobacter jejuni OXC7916	PubMLST	25046	354	0354	jejuni	no	yes	yes
Campylobacter jejuni OXC7917	PubMLST	25047	354	0354	jejuni	no	yes	yes
Campylobacter jejuni OXC7928	PubMLST	25057	354	0354	jejuni	no	yes	yes
Campylobacter jejuni OXC7970	PubMLST	25385	354	0354	jejuni	no	yes	yes
Campylobacter jejuni OXC7978	PubMLST	25393	354	0354	jejuni	no	yes	yes
Campylobacter jejuni OXC8013	PubMLST	25583	354	0354	jejuni	no	yes	yes
Campylobacter jejuni OXC8049	PubMLST	25595	354	0354	jejuni	no	yes	yes
Campylobacter jejuni OXC8084	PubMLST	25423	354	0354	jejuni	no	yes	yes
Campylobacter jejuni OXC8088	PubMLST	25522	354	0354	jejuni	no	yes	yes
Campylobacter jejuni OXC8095	PubMLST	25428	354	0354	jejuni	no	yes	yes
Campylobacter jejuni OXC8126	PubMLST	25458	354	0354	jejuni	no	yes	yes
Campylobacter jejuni OXC8130	PubMLST	25461	2033	0354	jejuni	no	yes	yes
Campylobacter jejuni OXC8174	PubMLST	25549	2033	0354	jejuni	no	yes	yes
Campylobacter jejuni OXC8188	PubMLST	27893	354	0354	jejuni	no	yes	yes
Campylobacter jejuni OXC8189	PubMLST	27894	354	0354	jejuni	no	yes	yes
Campylobacter jejuni OXC8204	PubMLST	27909	354	0354	jejuni	no	yes	yes
Campylobacter jejuni OXC8276	PubMLST	27969	354	0354	jejuni	no	yes	yes
Campylobacter jejuni OXC8281	PubMLST	27973	354	0354	jejuni	no	yes	yes
Campylobacter jejuni OXC8306	PubMLST	27997	354	0354	jejuni	no	yes	yes
Campylobacter jejuni OXC8333	PubMLST	28024	354	0354	jejuni	no	yes	yes
Campylobacter jejuni OXC8356	PubMLST	28045	2863	0354	jejuni	no	yes	yes
Campylobacter jejuni OXC8387	PubMLST	28580	354	0354	jejuni	no	yes	yes
Campylobacter jejuni OXC8420	PubMLST	28611	354	0354	jejuni	no	yes	yes
Campylobacter jejuni OXC8437	PubMLST	28628	354	0354	jejuni	no	yes	yes
Campylobacter jejuni OXC8467	PubMLST	28660	354	0354	jejuni	no	yes	yes
Campylobacter jejuni OXC8495	PubMLST	28686	354	0354	jejuni	no	yes	yes
Campylobacter jejuni OXC8508	PubMLST	28699	354	0354	jejuni	no	yes	yes
Campylobacter jejuni OXC8546	PubMLST	28735	354	0354	jejuni	no	yes	yes
Campylobacter jejuni OXC8562	PubMLST	28902	354	0354	jejuni	no	yes	yes
Campylobacter jejuni OXC8618	PubMLST	28945	354	0354	jejuni	no	yes	yes
Campylobacter jejuni OXC8619	PubMLST	28988	354	0354	jejuni	no	yes	yes
Campylobacter jejuni OXC8660	PubMLST	28995	354	0354	jejuni	no	yes	yes
Campylobacter jejuni OXC8721	PubMLST	29047	354	0354	jejuni	no	yes	yes
Campylobacter jejuni OXC8746	PubMLST	30437	354	0354	jejuni	no	yes	yes
Campylobacter jejuni OXC8764	PubMLST	30904	354	0354	jejuni	no	yes	yes
Campylobacter jejuni OXC8766	PubMLST	30455	354	0354	jejuni	no	yes	yes
Campylobacter jejuni OXC8781	PubMLST	30470	354	0354	jejuni	no	yes	yes
Campylobacter jejuni OXC8802	PubMLST	30491	354	0354	jejuni	no	yes	yes
Campylobacter jejuni OXC8828	PubMLST	30516	3155	0354	jejuni	no	yes	yes
Campylobacter jejuni OXC8877	PubMLST	30563	354	0354	jejuni	no	yes	yes
Campylobacter jejuni OXC8881	PubMLST	30567	354	0354	jejuni	no	yes	yes
Campylobacter jejuni OXC8883	PubMLST	30568	354	0354	jejuni	no	yes	yes
Campylobacter jejuni P140074	PubMLST	28862	354	0354	jejuni	no	yes	yes
Campylobacter jejuni BID1N7	PubMLST	25078	362	0362	jejuni	no	yes	yes
Campylobacter jejuni HN-CJD07035	Genbank/EMBL/DDBJ	ARYE01	362	0362	jejuni	no	yes	yes
Campylobacter jejuni ICDCCJ07002	Genbank/EMBL/DDBJ	APNP01	2993	0362	jejuni	no	yes	yes
Campylobacter jejuni ICDCCJ07004	Genbank/EMBL/DDBJ	APNQ01	2993	0362	jejuni	no	yes	yes
Campylobacter jejuni OXC4728	PubMLST	18435	362	0362	jejuni	no	yes	yes
Campylobacter jejuni ATCC 33560	Genbank/EMBL/DDBJ	AIJN01	403	0403	jejuni	no	yes	yes
Campylobacter jejuni BID1AV	PubMLST	24237	3219	0403	jejuni	no	yes	yes
Campylobacter jejuni BID1N5	PubMLST	25077	2294	0403	jejuni	no	yes	yes
Campylobacter jejuni OXC5170	PubMLST	24771	403	0403	jejuni	no	yes	yes
Campylobacter jejuni OXC5893	PubMLST	23675	5246	0403	jejuni	no	yes	yes
Campylobacter jejuni OXC5912	PubMLST	23638	403	0403	jejuni	no	yes	yes
Campylobacter jejuni OXC6349	PubMLST	16146	933	0403	jejuni	no	yes	yes
Campylobacter jejuni OXC6683	PubMLST	18240	270	0403	jejuni	no	yes	yes
Campylobacter jejuni OXC6740	PubMLST	18297	403	0403	jejuni	no	yes	yes
Campylobacter jejuni OXC6771	PubMLST	18328	270	0403	jejuni	no	yes	yes
Campylobacter jejuni OXC6869	PubMLST	21588	5246	0403	jejuni	no	yes	yes
Campylobacter jejuni OXC6883	PubMLST	22079	985	0403	jejuni	no	yes	yes

Campylobacter jejuni OXC6907	PubMLST	22102	932	0403	jejuni	no	yes	yes
Campylobacter jejuni OXC6911	PubMLST	22106	932	0403	jejuni	no	yes	yes
Campylobacter jejuni OXC7266	PubMLST	22307	270	0403	jejuni	no	yes	yes
Campylobacter jejuni OXC7548	PubMLST	24487	403	0403	jejuni	no	yes	yes
Campylobacter jejuni OXC7555	PubMLST	25105	403	0403	jejuni	no	yes	yes
Campylobacter jejuni OXC7579	PubMLST	25106	403	0403	jejuni	no	yes	yes
Campylobacter jejuni OXC7632	PubMLST	24561	270	0403	jejuni	no	yes	yes
Campylobacter jejuni OXC7671	PubMLST	25650	270	0403	jejuni	no	yes	yes
Campylobacter jejuni OXC7757	PubMLST	25454	270	0403	jejuni	no	yes	yes
Campylobacter jejuni OXC7794	PubMLST	27883	403	0403	jejuni	no	yes	yes
Campylobacter jejuni OXC7999	PubMLST	25486	270	0403	jejuni	no	yes	yes
Campylobacter jejuni OXC8164	PubMLST	25544	403	0403	jejuni	no	yes	yes
Campylobacter jejuni OXC8208	PubMLST	27913	403	0403	jejuni	no	yes	yes
Campylobacter jejuni OXC8616	PubMLST	28943	403	0403	jejuni	no	yes	yes
Campylobacter jejuni OXC8627	PubMLST	28948	7303	0403	jejuni	no	yes	yes
Campylobacter jejuni OXC8675	PubMLST	29007	985	0403	jejuni	no	yes	yes
Campylobacter jejuni OXC8702	PubMLST	29031	270	0403	jejuni	no	yes	yes
Campylobacter jejuni OXC8735	PubMLST	29060	985	0403	jejuni	no	yes	yes
Campylobacter jejuni OXC8737	PubMLST	29062	985	0403	jejuni	no	yes	yes
Campylobacter jejuni OXC8838	PubMLST	30525	1775	0403	jejuni	no	yes	yes
Campylobacter jejuni OXC8889	PubMLST	30573	270	0403	jejuni	no	yes	yes
Campylobacter jejuni OXC8930	PubMLST	30613	270	0403	jejuni	no	yes	yes
Campylobacter jejuni Dg280	PubMLST	26052	2574	0433	jejuni	no	yes	yes
Campylobacter jejuni OXC7932	PubMLST	25060	2574	0433	jejuni	no	yes	yes
Campylobacter jejuni H121820082	PubMLST	2636	51	0443	jejuni	no	yes	yes
Campylobacter jejuni LMG 9217	Genbank/EMBL/DDBJ	AIOO01	443	0443	jejuni	no	yes	yes
Campylobacter jejuni OXC4589	PubMLST	24060	51	0443	jejuni	no	yes	yes
Campylobacter jejuni OXC4594	PubMLST	25086	51	0443	jejuni	no	yes	yes
Campylobacter jejuni OXC4631	PubMLST	24081	51	0443	jejuni	no	yes	yes
Campylobacter jejuni OXC4669	PubMLST	24108	51	0443	jejuni	no	yes	yes
Campylobacter jejuni OXC4967	PubMLST	25097	443	0443	jejuni	no	yes	yes
Campylobacter jejuni OXC4987	PubMLST	24670	51	0443	jejuni	no	yes	yes
Campylobacter jejuni OXC5026	PubMLST	24694	51	0443	jejuni	no	yes	yes
Campylobacter jejuni OXC5051	PubMLST	24712	51	0443	jejuni	no	yes	yes
Campylobacter jejuni OXC5057	PubMLST	24716	51	0443	jejuni	no	yes	yes
Campylobacter jejuni OXC5059	PubMLST	24718	51	0443	jejuni	no	yes	yes
Campylobacter jejuni OXC5083	PubMLST	24727	51	0443	jejuni	no	yes	yes
Campylobacter jejuni OXC5138	PubMLST	24757	51	0443	jejuni	no	yes	yes
Campylobacter jejuni OXC5223	PubMLST	24809	51	0443	jejuni	no	yes	yes
Campylobacter jejuni OXC5303	PubMLST	24850	51	0443	jejuni	no	yes	yes
Campylobacter jejuni OXC5314	PubMLST	25473	51	0443	jejuni	no	yes	yes
Campylobacter jejuni OXC5317	PubMLST	25099	51	0443	jejuni	no	yes	yes
Campylobacter jejuni OXC5325	PubMLST	21311	51	0443	jejuni	no	yes	yes
Campylobacter jejuni OXC5460	PubMLST	21397	443	0443	jejuni	no	yes	yes
Campylobacter jejuni OXC5620	PubMLST	23509	51	0443	jejuni	no	yes	yes
Campylobacter jejuni OXC5646	PubMLST	23530	51	0443	jejuni	no	yes	yes
Campylobacter jejuni OXC5676	PubMLST	21423	51	0443	jejuni	no	yes	yes
Campylobacter jejuni OXC5702	PubMLST	21441	51	0443	jejuni	no	yes	yes
Campylobacter jejuni OXC5758	PubMLST	21480	51	0443	jejuni	no	yes	yes
Campylobacter jejuni OXC5764	PubMLST	21485	51	0443	jejuni	no	yes	yes
Campylobacter jejuni OXC5801	PubMLST	23551	51	0443	jejuni	no	yes	yes
Campylobacter jejuni OXC5805	PubMLST	23554	51	0443	jejuni	no	yes	yes
Campylobacter jejuni OXC5809	PubMLST	23557	51	0443	jejuni	no	yes	yes
Campylobacter jejuni OXC5873	PubMLST	23608	51	0443	jejuni	no	yes	yes
Campylobacter jejuni OXC5928	PubMLST	23650	6602	0443	jejuni	no	yes	yes
Campylobacter jejuni OXC6274	PubMLST	16071	51	0443	jejuni	no	yes	yes
Campylobacter jejuni OXC6302	PubMLST	16099	51	0443	jejuni	no	yes	yes
Campylobacter jejuni OXC6311	PubMLST	16108	51	0443	jejuni	no	yes	yes
Campylobacter jejuni OXC6316	PubMLST	16113	51	0443	jejuni	no	yes	yes
Campylobacter jejuni OXC6342	PubMLST	16139	51	0443	jejuni	no	yes	yes
Campylobacter jejuni OXC6357	PubMLST	16154	51	0443	jejuni	no	yes	yes
Campylobacter jejuni OXC6366	PubMLST	16163	51	0443	jejuni	no	yes	yes
Campylobacter jejuni OXC6396	PubMLST	16193	51	0443	jejuni	no	yes	yes
Campylobacter jejuni OXC6440	PubMLST	16234	51	0443	jejuni	no	yes	yes
Campylobacter jejuni OXC6481	PubMLST	16273	2361	0443	jejuni	no	yes	yes
Campylobacter jejuni OXC6503	PubMLST	16295	51	0443	jejuni	no	yes	yes
Campylobacter jejuni OXC6609	PubMLST	16359	51	0443	jejuni	no	yes	yes
Campylobacter jejuni OXC6620	PubMLST	16369	51	0443	jejuni	no	yes	yes
Campylobacter jejuni OXC6649	PubMLST	18206	51	0443	jejuni	no	yes	yes
Campylobacter jejuni OXC6668	PubMLST	18225	51	0443	jejuni	no	yes	yes
Campylobacter jejuni OXC6726	PubMLST	18283	51	0443	jejuni	no	yes	yes
Campylobacter jejuni OXC6759	PubMLST	18316	51	0443	jejuni	no	yes	yes
Campylobacter jejuni OXC6787	PubMLST	18344	51	0443	jejuni	no	yes	yes
Campylobacter jejuni OXC6834	PubMLST	18390	51	0443	jejuni	no	yes	yes
Campylobacter jejuni OXC6906	PubMLST	22101	4243	0443	jejuni	no	yes	yes
Campylobacter jejuni OXC6910	PubMLST	22105	51	0443	jejuni	no	yes	yes
Campylobacter jejuni OXC6919	PubMLST	22114	51	0443	jejuni	no	yes	yes
Campylobacter jejuni OXC6951	PubMLST	21125	51	0443	jejuni	no	yes	yes
Campylobacter jejuni OXC6952	PubMLST	21126	51	0443	jejuni	no	yes	yes
Campylobacter jejuni OXC6963	PubMLST	21137	5794	0443	jejuni	no	yes	yes
Campylobacter jejuni OXC7001	PubMLST	21172	51	0443	jejuni	no	yes	yes
Campylobacter jejuni OXC7003	PubMLST	22190	51	0443	jejuni	no	yes	yes
Campylobacter jejuni OXC7004	PubMLST	22191	51	0443	jejuni	no	yes	yes
Campylobacter jejuni OXC7038	PubMLST	22224	51	0443	jejuni	no	yes	yes
Campylobacter jejuni OXC7087	PubMLST	22271	51	0443	jejuni	no	yes	yes
Campylobacter jejuni OXC7157	PubMLST	21238	51	0443	jejuni	no	yes	yes
Campylobacter jejuni OXC7201	PubMLST	22692	1703	0443	jejuni	no	yes	yes
Campylobacter jejuni OXC7250	PubMLST	22292	51	0443	jejuni	no	yes	yes
Campylobacter jejuni OXC7273	PubMLST	22314	51	0443	jejuni	no	yes	yes
Campylobacter jejuni OXC7339	PubMLST	22376	443	0443	jejuni	no	yes	yes
Campylobacter jejuni OXC7378	PubMLST	23916	51	0443	jejuni	no	yes	yes
Campylobacter jejuni OXC7437	PubMLST	23968	51	0443	jejuni	no	yes	yes

Campylobacter jejuni OXC7450	PubMLST	23980	2361	0443	jejuni	no	yes	yes
Campylobacter jejuni OXC7480	PubMLST	24009	51	0443	jejuni	no	yes	yes
Campylobacter jejuni OXC7487	PubMLST	24015	51	0443	jejuni	no	yes	yes
Campylobacter jejuni OXC7493	PubMLST	24021	51	0443	jejuni	no	yes	yes
Campylobacter jejuni OXC7518	PubMLST	24123	51	0443	jejuni	no	yes	yes
Campylobacter jejuni OXC7537	PubMLST	24480	51	0443	jejuni	no	yes	yes
Campylobacter jejuni OXC7749	PubMLST	24984	51	0443	jejuni	no	yes	yes
Campylobacter jejuni OXC7775	PubMLST	24582	51	0443	jejuni	no	yes	yes
Campylobacter jejuni OXC7857	PubMLST	24992	51	0443	jejuni	no	yes	yes
Campylobacter jejuni OXC7980	PubMLST	25575	51	0443	jejuni	no	yes	yes
Campylobacter jejuni OXC8012	PubMLST	25582	51	0443	jejuni	no	yes	yes
Campylobacter jejuni OXC8076	PubMLST	25419	51	0443	jejuni	no	yes	yes
Campylobacter jejuni OXC8109	PubMLST	25614	51	0443	jejuni	no	yes	yes
Campylobacter jejuni OXC8150	PubMLST	25541	51	0443	jejuni	no	yes	yes
Campylobacter jejuni OXC8155	PubMLST	25437	51	0443	jejuni	no	yes	yes
Campylobacter jejuni OXC8165	PubMLST	25439	51	0443	jejuni	no	yes	yes
Campylobacter jejuni OXC8166	PubMLST	25440	51	0443	jejuni	no	yes	yes
Campylobacter jejuni OXC8260	PubMLST	27956	51	0443	jejuni	no	yes	yes
Campylobacter jejuni OXC8268	PubMLST	27962	51	0443	jejuni	no	yes	yes
Campylobacter jejuni OXC8304	PubMLST	27995	6986	0443	jejuni	no	yes	yes
Campylobacter jejuni OXC8309	PubMLST	28000	51	0443	jejuni	no	yes	yes
Campylobacter jejuni OXC8316	PubMLST	28007	51	0443	jejuni	no	yes	yes
Campylobacter jejuni OXC8365	PubMLST	28053	51	0443	jejuni	no	yes	yes
Campylobacter jejuni OXC8382	PubMLST	28575	51	0443	jejuni	no	yes	yes
Campylobacter jejuni OXC8395	PubMLST	28588	51	0443	jejuni	no	yes	yes
Campylobacter jejuni OXC8403	PubMLST	28595	51	0443	jejuni	no	yes	yes
Campylobacter jejuni OXC8433	PubMLST	28624	51	0443	jejuni	no	yes	yes
Campylobacter jejuni OXC8435	PubMLST	28626	51	0443	jejuni	no	yes	yes
Campylobacter jejuni OXC8442	PubMLST	28633	51	0443	jejuni	no	yes	yes
Campylobacter jejuni OXC8448	PubMLST	28639	51	0443	jejuni	no	yes	yes
Campylobacter jejuni OXC8459	PubMLST	28650	51	0443	jejuni	no	yes	yes
Campylobacter jejuni OXC8515	PubMLST	28706	51	0443	jejuni	no	yes	yes
Campylobacter jejuni OXC8516	PubMLST	28707	51	0443	jejuni	no	yes	yes
Campylobacter jejuni OXC8526	PubMLST	28717	51	0443	jejuni	no	yes	yes
Campylobacter jejuni OXC8534	PubMLST	28724	51	0443	jejuni	no	yes	yes
Campylobacter jejuni OXC8556	PubMLST	28745	51	0443	jejuni	no	yes	yes
Campylobacter jejuni OXC8625	PubMLST	28990	51	0443	jejuni	no	yes	yes
Campylobacter jejuni OXC8682	PubMLST	29014	6522	0443	jejuni	no	yes	yes
Campylobacter jejuni OXC8712	PubMLST	29041	51	0443	jejuni	no	yes	yes
Campylobacter jejuni OXC8829	PubMLST	30517	51	0443	jejuni	no	yes	yes
Campylobacter jejuni OXC8924	PubMLST	30607	51	0443	jejuni	no	yes	yes
Campylobacter jejuni OXC8973	PubMLST	30655	51	0443	jejuni	no	yes	yes
Campylobacter jejuni LMG 23263	Genbank/EMBL/DDBJ	AIOD01	3504	0446	jejuni	no	yes	yes
Campylobacter jejuni OXC6697	PubMLST	18254	862	0446	jejuni	no	yes	yes
Campylobacter jejuni OXC6843	PubMLST	21562	446	0446	jejuni	no	yes	yes
Campylobacter jejuni OXC6874	PubMLST	21593	446	0446	jejuni	no	yes	yes
Campylobacter jejuni OXC6875	PubMLST	21594	446	0446	jejuni	no	yes	yes
Campylobacter jejuni OXC6954	PubMLST	21128	2850	0446	jejuni	no	yes	yes
Campylobacter jejuni OXC7604_R	PubMLST	25554	3269	0446	jejuni	no	yes	yes
Campylobacter jejuni OXC7744	PubMLST	24979	450	0446	jejuni	no	yes	yes
Campylobacter jejuni OXC7941	PubMLST	25120	450	0446	jejuni	no	yes	yes
Campylobacter jejuni OXC8468	PubMLST	28661	5626	0446	jejuni	no	yes	yes
Campylobacter jejuni OXC8545	PubMLST	28734	3552	0446	jejuni	no	yes	yes
Campylobacter jejuni 10227	Genbank/EMBL/DDBJ	AUUI01	460	0460	jejuni	no	yes	yes
Campylobacter jejuni BID1NV	PubMLST	25124	535	0460	jejuni	no	yes	yes
Campylobacter jejuni CJ1	Genbank/EMBL/DDBJ	AUUL01	3919	0460	jejuni	no	yes	yes
Campylobacter jejuni H122360313	PubMLST	2680	460	0460	jejuni	no	yes	yes
Campylobacter jejuni OXC5322	PubMLST	25373	1932	0460	jejuni	no	yes	yes
Campylobacter jejuni OXC7328	PubMLST	22366	535	0460	jejuni	no	yes	yes
Campylobacter jejuni OXC7449	PubMLST	23979	113	0460	jejuni	no	yes	yes
Campylobacter jejuni OXC7683	PubMLST	24936	460	0460	jejuni	no	yes	yes
Campylobacter jejuni OXC7751	PubMLST	25453	670	0460	jejuni	no	yes	yes
Campylobacter jejuni OXC7877	PubMLST	25012	1932	0460	jejuni	no	yes	yes
Campylobacter jejuni OXC7967	PubMLST	25382	1932	0460	jejuni	no	yes	yes
Campylobacter jejuni OXC8361	PubMLST	28049	670	0460	jejuni	no	yes	yes
Campylobacter jejuni OXC8498	PubMLST	28689	5484	0460	jejuni	no	yes	yes
Campylobacter jejuni OXC8709	PubMLST	29038	670	0460	jejuni	no	yes	yes
Campylobacter jejuni OXC8833	PubMLST	30521	1074	0460	jejuni	no	yes	yes
Campylobacter jejuni OXC9016	PubMLST	30433	670	0460	jejuni	no	yes	yes
Campylobacter jejuni CJ5	Genbank/EMBL/DDBJ	AUUK01	464	0464	jejuni	no	yes	yes
Campylobacter jejuni H142240928	PubMLST	2675	464	0464	jejuni	no	yes	yes
Campylobacter jejuni OXC4621	PubMLST	24074	464	0464	jejuni	no	yes	yes
Campylobacter jejuni OXC4660	PubMLST	24101	464	0464	jejuni	no	yes	yes
Campylobacter jejuni OXC4773	PubMLST	18463	464	0464	jejuni	no	yes	yes
Campylobacter jejuni OXC5238	PubMLST	24821	464	0464	jejuni	no	yes	yes
Campylobacter jejuni OXC5630	PubMLST	23516	2315	0464	jejuni	no	yes	yes
Campylobacter jejuni OXC5631	PubMLST	23517	2315	0464	jejuni	no	yes	yes
Campylobacter jejuni OXC5863	PubMLST	23601	464	0464	jejuni	no	yes	yes
Campylobacter jejuni OXC6458	PubMLST	16250	5731	0464	jejuni	no	yes	yes
Campylobacter jejuni OXC6468	PubMLST	16260	5732	0464	jejuni	no	yes	yes
Campylobacter jejuni OXC6800	PubMLST	18357	2146	0464	jejuni	no	yes	yes
Campylobacter jejuni OXC6807	PubMLST	18364	464	0464	jejuni	no	yes	yes
Campylobacter jejuni OXC6839	PubMLST	21558	464	0464	jejuni	no	yes	yes
Campylobacter jejuni OXC7381	PubMLST	23919	464	0464	jejuni	no	yes	yes
Campylobacter jejuni OXC7418	PubMLST	23952	6702	0464	jejuni	no	yes	yes
Campylobacter jejuni OXC7446	PubMLST	23976	464	0464	jejuni	no	yes	yes
Campylobacter jejuni OXC7565	PubMLST	24502	6704	0464	jejuni	no	yes	yes
Campylobacter jejuni OXC7755	PubMLST	24987	464	0464	jejuni	no	yes	yes
Campylobacter jejuni OXC7882	PubMLST	25015	2146	0464	jejuni	no	yes	yes
Campylobacter jejuni OXC7931	PubMLST	25059	464	0464	jejuni	no	yes	yes
Campylobacter jejuni OXC8097	PubMLST	25612	464	0464	jejuni	no	yes	yes
Campylobacter jejuni OXC8098	PubMLST	25526	5849	0464	jejuni	no	yes	yes

Campylobacter jejuni OXC8114	PubMLST	25534	464	0464	jejuni	no	yes	yes
Campylobacter jejuni OXC8386	PubMLST	28579	464	0464	jejuni	no	yes	yes
Campylobacter jejuni OXC8488	PubMLST	28680	464	0464	jejuni	no	yes	yes
Campylobacter jejuni OXC8656	PubMLST	28970	2146	0464	jejuni	no	yes	yes
Campylobacter jejuni OXC8657	PubMLST	28971	2146	0464	jejuni	no	yes	yes
Campylobacter jejuni OXC8687	PubMLST	29019	2146	0464	jejuni	no	yes	yes
Campylobacter jejuni P854	Genbank/EMBL/DDBJ	AKFN01	573	0573	jejuni	no	yes	yes
Campylobacter jejuni 30286	Genbank/EMBL/DDBJ	AUUH01	305	0574	jejuni	no	yes	yes
Campylobacter jejuni BID1EV	PubMLST	25121	1040	0574	jejuni	no	yes	yes
Campylobacter jejuni CG8486	Genbank/EMBL/DDBJ	AASY01	2943	0574	jejuni	no	yes	yes
Campylobacter jejuni CJ3	Genbank/EMBL/DDBJ	AUUN01	574	0574	jejuni	no	yes	yes
Campylobacter jejuni OXC4682	PubMLST	24117	574	0574	jejuni	no	yes	yes
Campylobacter jejuni OXC4731	PubMLST	18436	574	0574	jejuni	no	yes	yes
Campylobacter jejuni OXC4742	PubMLST	18444	574	0574	jejuni	no	yes	yes
Campylobacter jejuni OXC4844	PubMLST	22161	919	0574	jejuni	no	yes	yes
Campylobacter jejuni OXC4856	PubMLST	22168	574	0574	jejuni	no	yes	yes
Campylobacter jejuni OXC4911	PubMLST	25562	574	0574	jejuni	no	yes	yes
Campylobacter jejuni OXC5145	PubMLST	24760	574	0574	jejuni	no	yes	yes
Campylobacter jejuni OXC5193	PubMLST	24788	574	0574	jejuni	no	yes	yes
Campylobacter jejuni OXC5256	PubMLST	24834	574	0574	jejuni	no	yes	yes
Campylobacter jejuni OXC5380	PubMLST	21345	2140	0574	jejuni	no	yes	yes
Campylobacter jejuni OXC5394	PubMLST	21354	574	0574	jejuni	no	yes	yes
Campylobacter jejuni OXC5452	PubMLST	21391	574	0574	jejuni	no	yes	yes
Campylobacter jejuni OXC5616	PubMLST	23506	574	0574	jejuni	no	yes	yes
Campylobacter jejuni OXC5776	PubMLST	21496	574	0574	jejuni	no	yes	yes
Campylobacter jejuni OXC5782	PubMLST	23536	574	0574	jejuni	no	yes	yes
Campylobacter jejuni OXC5787	PubMLST	23539	574	0574	jejuni	no	yes	yes
Campylobacter jejuni OXC5792	PubMLST	23543	574	0574	jejuni	no	yes	yes
Campylobacter jejuni OXC5812	PubMLST	23560	574	0574	jejuni	no	yes	yes
Campylobacter jejuni OXC5815	PubMLST	23563	574	0574	jejuni	no	yes	yes
Campylobacter jejuni OXC5914	PubMLST	23639	574	0574	jejuni	no	yes	yes
Campylobacter jejuni OXC6304	PubMLST	16101	574	0574	jejuni	no	yes	yes
Campylobacter jejuni OXC6323	PubMLST	16120	574	0574	jejuni	no	yes	yes
Campylobacter jejuni OXC6361	PubMLST	16158	574	0574	jejuni	no	yes	yes
Campylobacter jejuni OXC6391	PubMLST	16188	574	0574	jejuni	no	yes	yes
Campylobacter jejuni OXC6392	PubMLST	16189	574	0574	jejuni	no	yes	yes
Campylobacter jejuni OXC6509	PubMLST	16301	1040	0574	jejuni	no	yes	yes
Campylobacter jejuni OXC6574	PubMLST	12909	574	0574	jejuni	no	yes	yes
Campylobacter jejuni OXC6680	PubMLST	18237	574	0574	jejuni	no	yes	yes
Campylobacter jejuni OXC6746	PubMLST	18303	574	0574	jejuni	no	yes	yes
Campylobacter jejuni OXC6790	PubMLST	18347	574	0574	jejuni	no	yes	yes
Campylobacter jejuni OXC6799	PubMLST	18356	574	0574	jejuni	no	yes	yes
Campylobacter jejuni OXC6809	PubMLST	18366	574	0574	jejuni	no	yes	yes
Campylobacter jejuni OXC6821	PubMLST	18378	574	0574	jejuni	no	yes	yes
Campylobacter jejuni OXC6838	PubMLST	21557	574	0574	jejuni	no	yes	yes
Campylobacter jejuni OXC6890	PubMLST	22085	574	0574	jejuni	no	yes	yes
Campylobacter jejuni OXC6898	PubMLST	22093	574	0574	jejuni	no	yes	yes
Campylobacter jejuni OXC6957	PubMLST	21131	574	0574	jejuni	no	yes	yes
Campylobacter jejuni OXC6976	PubMLST	21149	574	0574	jejuni	no	yes	yes
Campylobacter jejuni OXC7079	PubMLST	22263	574	0574	jejuni	no	yes	yes
Campylobacter jejuni OXC7098	PubMLST	22280	574	0574	jejuni	no	yes	yes
Campylobacter jejuni OXC7128	PubMLST	21209	574	0574	jejuni	no	yes	yes
Campylobacter jejuni OXC7319	PubMLST	22358	574	0574	jejuni	no	yes	yes
Campylobacter jejuni OXC7389	PubMLST	23927	574	0574	jejuni	no	yes	yes
Campylobacter jejuni OXC7395	PubMLST	23933	305	0574	jejuni	no	yes	yes
Campylobacter jejuni OXC7505	PubMLST	24031	574	0574	jejuni	no	yes	yes
Campylobacter jejuni OXC7640	PubMLST	24569	574	0574	jejuni	no	yes	yes
Campylobacter jejuni OXC7675	PubMLST	24929	574	0574	jejuni	no	yes	yes
Campylobacter jejuni OXC7682	PubMLST	24935	574	0574	jejuni	no	yes	yes
Campylobacter jejuni OXC7827	PubMLST	24624	574	0574	jejuni	no	yes	yes
Campylobacter jejuni OXC7865	PubMLST	25000	574	0574	jejuni	no	yes	yes
Campylobacter jejuni OXC7984	PubMLST	25397	574	0574	jejuni	no	yes	yes
Campylobacter jejuni OXC8059	PubMLST	25602	574	0574	jejuni	no	yes	yes
Campylobacter jejuni OXC8223	PubMLST	27925	574	0574	jejuni	no	yes	yes
Campylobacter jejuni OXC8252	PubMLST	27950	574	0574	jejuni	no	yes	yes
Campylobacter jejuni OXC8253	PubMLST	27951	574	0574	jejuni	no	yes	yes
Campylobacter jejuni OXC8285	PubMLST	27977	574	0574	jejuni	no	yes	yes
Campylobacter jejuni OXC8532	PubMLST	28722	574	0574	jejuni	no	yes	yes
Campylobacter jejuni OXC8540	PubMLST	28730	1040	0574	jejuni	no	yes	yes
Campylobacter jejuni OXC8553	PubMLST	28742	1040	0574	jejuni	no	yes	yes
Campylobacter jejuni OXC8572	PubMLST	28979	574	0574	jejuni	no	yes	yes
Campylobacter jejuni OXC8580	PubMLST	28917	574	0574	jejuni	no	yes	yes
Campylobacter jejuni OXC8592	PubMLST	28925	574	0574	jejuni	no	yes	yes
Campylobacter jejuni OXC8789	PubMLST	30478	1040	0574	jejuni	no	yes	yes
Campylobacter jejuni OXC9009	PubMLST	30689	574	0574	jejuni	no	yes	yes
Campylobacter jejuni OXC4610	PubMLST	22129	1707	0607	jejuni	no	yes	yes
Campylobacter jejuni OXC5326	PubMLST	21312	607	0607	jejuni	no	yes	yes
Campylobacter jejuni OXC5412	PubMLST	21366	5134	0607	jejuni	no	yes	yes
Campylobacter jejuni OXC5464	PubMLST	21400	607	0607	jejuni	no	yes	yes
Campylobacter jejuni OXC5732	PubMLST	21462	607	0607	jejuni	no	yes	yes
Campylobacter jejuni OXC5789	PubMLST	23541	904	0607	jejuni	no	yes	yes
Campylobacter jejuni OXC6262	PubMLST	16059	904	0607	jejuni	no	yes	yes
Campylobacter jejuni OXC6341	PubMLST	16138	904	0607	jejuni	no	yes	yes
Campylobacter jejuni OXC6359	PubMLST	16156	904	0607	jejuni	no	yes	yes
Campylobacter jejuni OXC6466	PubMLST	16258	904	0607	jejuni	no	yes	yes
Campylobacter jejuni OXC6560	PubMLST	12895	904	0607	jejuni	no	yes	yes
Campylobacter jejuni OXC6930	PubMLST	22125	4056	0607	jejuni	no	yes	yes
Campylobacter jejuni OXC6931	PubMLST	22126	4056	0607	jejuni	no	yes	yes
Campylobacter jejuni OXC6960	PubMLST	21134	4056	0607	jejuni	no	yes	yes
Campylobacter jejuni OXC7072	PubMLST	22256	904	0607	jejuni	no	yes	yes
Campylobacter jejuni OXC7210	PubMLST	22701	2103	0607	jejuni	no	yes	yes
Campylobacter jejuni OXC7217	PubMLST	22708	607	0607	jejuni	no	yes	yes

Campylobacter jejuni OXC7252	PubMLST	22294	904	0607	jejuni	no	yes	yes
Campylobacter jejuni OXC7323	PubMLST	22362	6700	0607	jejuni	no	yes	yes
Campylobacter jejuni OXC7443	PubMLST	23974	2103	0607	jejuni	no	yes	yes
Campylobacter jejuni OXC7511	PubMLST	24036	904	0607	jejuni	no	yes	yes
Campylobacter jejuni OXC7552	PubMLST	24490	904	0607	jejuni	no	yes	yes
Campylobacter jejuni OXC8017	PubMLST	25408	904	0607	jejuni	no	yes	yes
Campylobacter jejuni OXC8314	PubMLST	28005	904	0607	jejuni	no	yes	yes
Campylobacter jejuni OXC8315	PubMLST	28006	904	0607	jejuni	no	yes	yes
Campylobacter jejuni OXC8485	PubMLST	28678	607	0607	jejuni	no	yes	yes
Campylobacter jejuni OXC8593	PubMLST	28926	607	0607	jejuni	no	yes	yes
Campylobacter jejuni OXC8645	PubMLST	28959	607	0607	jejuni	no	yes	yes
Campylobacter jejuni OXC8935	PubMLST	30618	904	0607	jejuni	no	yes	yes
Campylobacter jejuni 1997-1	Genbank/EMBL/DDBJ	AIOT01	658	0658	jejuni	no	yes	yes
Campylobacter jejuni Dg88b	PubMLST	26012	658	0658	jejuni	no	yes	yes
Campylobacter jejuni OXC4604	PubMLST	24071	1044	0658	jejuni	no	yes	yes
Campylobacter jejuni OXC4616	PubMLST	22131	658	0658	jejuni	no	yes	yes
Campylobacter jejuni OXC4635	PubMLST	24085	658	0658	jejuni	no	yes	yes
Campylobacter jejuni OXC4737	PubMLST	18440	1044	0658	jejuni	no	yes	yes
Campylobacter jejuni OXC4816	PubMLST	22151	1044	0658	jejuni	no	yes	yes
Campylobacter jejuni OXC4861	PubMLST	22173	658	0658	jejuni	no	yes	yes
Campylobacter jejuni OXC4862	PubMLST	22174	658	0658	jejuni	no	yes	yes
Campylobacter jejuni OXC4903	PubMLST	24463	1044	0658	jejuni	no	yes	yes
Campylobacter jejuni OXC4905	PubMLST	24465	1044	0658	jejuni	no	yes	yes
Campylobacter jejuni OXC5179	PubMLST	24776	1044	0658	jejuni	no	yes	yes
Campylobacter jejuni OXC5263	PubMLST	24840	658	0658	jejuni	no	yes	yes
Campylobacter jejuni OXC5689	PubMLST	21432	658	0658	jejuni	no	yes	yes
Campylobacter jejuni OXC5761	PubMLST	21482	523	0658	jejuni	no	yes	yes
Campylobacter jejuni OXC5775	PubMLST	21495	658	0658	jejuni	no	yes	yes
Campylobacter jejuni OXC5793	PubMLST	23544	523	0658	jejuni	no	yes	yes
Campylobacter jejuni OXC5852	PubMLST	23668	658	0658	jejuni	no	yes	yes
Campylobacter jejuni OXC5876	PubMLST	23673	5245	0658	jejuni	no	yes	yes
Campylobacter jejuni OXC6363	PubMLST	16160	658	0658	jejuni	no	yes	yes
Campylobacter jejuni OXC6381	PubMLST	16178	523	0658	jejuni	no	yes	yes
Campylobacter jejuni OXC6436	PubMLST	16231	1044	0658	jejuni	no	yes	yes
Campylobacter jejuni OXC6492	PubMLST	16284	1044	0658	jejuni	no	yes	yes
Campylobacter jejuni OXC6533	PubMLST	16325	658	0658	jejuni	no	yes	yes
Campylobacter jejuni OXC6545	PubMLST	12880	658	0658	jejuni	no	yes	yes
Campylobacter jejuni OXC6618	PubMLST	16367	1900	0658	jejuni	no	yes	yes
Campylobacter jejuni OXC6679	PubMLST	18236	658	0658	jejuni	no	yes	yes
Campylobacter jejuni OXC6835	PubMLST	21554	1044	0658	jejuni	no	yes	yes
Campylobacter jejuni OXC7180	PubMLST	22671	658	0658	jejuni	no	yes	yes
Campylobacter jejuni OXC7242	PubMLST	22284	658	0658	jejuni	no	yes	yes
Campylobacter jejuni OXC7261	PubMLST	22302	658	0658	jejuni	no	yes	yes
Campylobacter jejuni OXC7277	PubMLST	22318	1044	0658	jejuni	no	yes	yes
Campylobacter jejuni OXC7316	PubMLST	22357	1044	0658	jejuni	no	yes	yes
Campylobacter jejuni OXC7353	PubMLST	23892	658	0658	jejuni	no	yes	yes
Campylobacter jejuni OXC7462	PubMLST	23991	1044	0658	jejuni	no	yes	yes
Campylobacter jejuni OXC7466	PubMLST	23995	1044	0658	jejuni	no	yes	yes
Campylobacter jejuni OXC7513	PubMLST	24038	658	0658	jejuni	no	yes	yes
Campylobacter jejuni OXC7689	PubMLST	24939	658	0658	jejuni	no	yes	yes
Campylobacter jejuni OXC7728	PubMLST	24969	523	0658	jejuni	no	yes	yes
Campylobacter jejuni OXC7798	PubMLST	24599	1044	0658	jejuni	no	yes	yes
Campylobacter jejuni OXC7807	PubMLST	24605	658	0658	jejuni	no	yes	yes
Campylobacter jejuni OXC7855	PubMLST	24990	658	0658	jejuni	no	yes	yes
Campylobacter jejuni OXC7858	PubMLST	24993	658	0658	jejuni	no	yes	yes
Campylobacter jejuni OXC7870	PubMLST	25005	658	0658	jejuni	no	yes	yes
Campylobacter jejuni OXC7884	PubMLST	25017	658	0658	jejuni	no	yes	yes
Campylobacter jejuni OXC7935	PubMLST	25118	657	0658	jejuni	no	yes	yes
Campylobacter jejuni OXC7946	PubMLST	25456	658	0658	jejuni	no	yes	yes
Campylobacter jejuni OXC8036	PubMLST	25411	1044	0658	jejuni	no	yes	yes
Campylobacter jejuni OXC8086	PubMLST	25425	658	0658	jejuni	no	yes	yes
Campylobacter jejuni OXC8101	PubMLST	25529	658	0658	jejuni	no	yes	yes
Campylobacter jejuni OXC8157	PubMLST	25640	1044	0658	jejuni	no	yes	yes
Campylobacter jejuni OXC8409	PubMLST	28600	658	0658	jejuni	no	yes	yes
Campylobacter jejuni OXC8530	PubMLST	28982	658	0658	jejuni	no	yes	yes
Campylobacter jejuni OXC8530R	PubMLST	29085	658	0658	jejuni	no	yes	yes
Campylobacter jejuni OXC8573	PubMLST	28910	1044	0658	jejuni	no	yes	yes
Campylobacter jejuni OXC8581	PubMLST	28918	1044	0658	jejuni	no	yes	yes
Campylobacter jejuni OXC8587	PubMLST	28923	1044	0658	jejuni	no	yes	yes
Campylobacter jejuni OXC8611	PubMLST	28986	658	0658	jejuni	no	yes	yes
Campylobacter jejuni OXC8760	PubMLST	30450	2180	0658	jejuni	no	yes	yes
Campylobacter jejuni OXC8765	PubMLST	30454	658	0658	jejuni	no	yes	yes
Campylobacter jejuni OXC8846	PubMLST	30532	658	0658	jejuni	no	yes	yes
Campylobacter jejuni OXC8943	PubMLST	30626	1044	0658	jejuni	no	yes	yes
Campylobacter jejuni OXC8946	PubMLST	30629	1044	0658	jejuni	no	yes	yes
Campylobacter jejuni OXC1211	PubMLST	13782	661	0661	jejuni	no	yes	yes
Campylobacter jejuni OXC1550	PubMLST	14042	661	0661	jejuni	no	yes	yes
Campylobacter jejuni OXC6795	PubMLST	18352	814	0661	jejuni	no	yes	yes
Campylobacter jejuni OXC6921	PubMLST	22116	814	0661	jejuni	no	yes	yes
Campylobacter jejuni OXC7007	PubMLST	22194	814	0661	jejuni	no	yes	yes
Campylobacter jejuni OXC7895	PubMLST	25026	814	0661	jejuni	no	yes	yes
Campylobacter jejuni OXC6655	PubMLST	18212	991	0692	jejuni	no	yes	yes
Campylobacter jejuni OXC7162	PubMLST	21243	4776	0692	jejuni	no	yes	yes
Campylobacter jejuni OXC7562	PubMLST	24499	692	0692	jejuni	no	yes	yes
Campylobacter jejuni OXC8543	PubMLST	28899	991	0692	jejuni	no	yes	yes
Campylobacter jejuni OXC8543R	PubMLST	29069	991	0692	jejuni	no	yes	yes
Campylobacter jejuni OXC4948	PubMLST	25095	2314	1034	jejuni	no	yes	yes
Campylobacter jejuni OXC4958	PubMLST	25468	2314	1034	jejuni	no	yes	yes
Campylobacter jejuni OXC5003	PubMLST	24680	2314	1034	jejuni	no	yes	yes
Campylobacter jejuni OXC5490	PubMLST	23495	977	1034	jejuni	no	yes	yes
Campylobacter jejuni OXC5778	PubMLST	21498	1709	1034	jejuni	no	yes	yes
Campylobacter jejuni OXC5800	PubMLST	23550	1709	1034	jejuni	no	yes	yes

Campylobacter jejuni OXC6261	PubMLST	16058	5717	1034	jejuni	no	yes	yes
Campylobacter jejuni OXC6498	PubMLST	16290	1709	1034	jejuni	no	yes	yes
Campylobacter jejuni OXC6599	PubMLST	16350	2314	1034	jejuni	no	yes	yes
Campylobacter jejuni OXC6811	PubMLST	18368	5817	1034	jejuni	no	yes	yes
Campylobacter jejuni OXC7696	PubMLST	24944	1709	1034	jejuni	no	yes	yes
Campylobacter jejuni OXC8492	PubMLST	28684	2314	1034	jejuni	no	yes	yes
Campylobacter jejuni LMG 23357	Genbank/EMBL/DDBJ	AIOK01	4883	1275	jejuni	no	yes	yes
Campylobacter jejuni OXC8001	PubMLST	25406	945	1287	jejuni	no	yes	yes
Campylobacter jejuni OXC8060	PubMLST	25415	945	1287	jejuni	no	yes	yes
Campylobacter jejuni Dg195	PubMLST	24197	6563	1332	jejuni	no	yes	yes
Campylobacter jejuni Dg368	PubMLST	26021	6563	1332	jejuni	no	yes	yes
Campylobacter jejuni OXC5407	PubMLST	21363	5154	1332	jejuni	no	yes	yes

Table S4. EMBL/Genbank/DDBJ Accession numbers of *Campylobacter jejuni/coli* genomes with CRISPR elements, and *Campylobacter* bacteriophage genomes, plasmid sequences and insertion elements/prophages included in this study

Nucleotide numbers for the CJIE1-4 of *Campylobacter jejuni* RM1221 insertion elements/prophages are included.

Campylobacter jejuni/coli phages	Accession number
Campylobacter phage CP8	KF148616
Campylobacter phage CP30A	NC_018861
Campylobacter phage CPX	JN132397
Campylobacter phage CP21	HE815464
Campylobacter phage vB_CcoM-IBB_35	HM246720
Campylobacter phage CPt10	FN667789
Campylobacter phage CP81	FR823450
Campylobacter phage CP220	FN667788
Campylobacter phage NCTC12673	GU296433

Campylobacter jejuni/coli plasmids	Accession number
Campylobacter coli plasmid p3384	NC_007142
Campylobacter coli plasmid p3386	NC_007143
Campylobacter coli plasmid pCC31	NC_006134
Campylobacter coli RM2228 plasmid pCC2228-1	NC_008049
Campylobacter coli RM2228 plasmid pCC2228-2	NC_008050
Campylobacter coli RM2228 plasmid pCC2228-3	NC_008051
Campylobacter jejuni 81-176 virulence plasmid pVir	CP000550
Campylobacter jejuni plasmid pCG8245	AY701528
Campylobacter jejuni plasmid pCJ01	AF301164
Campylobacter jejuni plasmid pCJ1170	DQ518173
Campylobacter jejuni plasmid pCJ419	AY256846
Campylobacter jejuni strain S4-2 CC plasmid pTIW94	KF192842
Campylobacter jejuni 81-176 plasmid pTet	NC_006135
Campylobacter jejuni IA3902 plasmid pVir	CP001877
Campylobacter jejuni S3 plasmid pTet	CP001961
Campylobacter coli CVM N29710 plasmid pN29710-1	CP004067
Campylobacter coli CVM N29710 plasmid pN29710-2	CP004068

Campylobacter jejuni insertion elements/prophages	Accession number
Campylobacter jejuni RM1221 (CJIE1, CJIE2, CJIE3, CJIE4)	NC_003912
CJIE1: positions 207005-244247 on RM1221 genome	
CJIE2: positions 498203-538770 on RM1221 genome	
CJIE3: positions 1021082-1071873 on RM1221 genome	
CJIE4: positions 1335703-1371932 on RM1221 genome	

CRISPR sequences included	Accession number
Campylobacter jejuni strain R138 CRISPR region	HQ378324
Campylobacter jejuni strain GB38 CRISPR region	HQ378323
Campylobacter jejuni strain GB37 CRISPR region	HQ378322
Campylobacter jejuni strain GB35 CRISPR region	HQ378321
Campylobacter jejuni strain GB33 CRISPR region	HQ378320
Campylobacter jejuni strain GB31 CRISPR region	HQ378319
Campylobacter jejuni strain GB29 CRISPR region	HQ378318
Campylobacter jejuni strain GB28 CRISPR region	HQ378317
Campylobacter jejuni strain GB27 CRISPR region	HQ378316
Campylobacter jejuni strain GB26 CRISPR region	HQ378315
Campylobacter jejuni strain GB25 CRISPR region	HQ378314
Campylobacter jejuni strain GB24 CRISPR region	HQ378313
Campylobacter jejuni strain GB23 CRISPR region	HQ378312
Campylobacter jejuni strain GB22 CRISPR region	HQ378311
Campylobacter jejuni strain GB19 CRISPR region	HQ378310
Campylobacter jejuni strain GB16 CRISPR region	HQ378309
Campylobacter jejuni strain GB14 CRISPR region	HQ378308
Campylobacter jejuni strain GB5 CRISPR region	HQ378307

Campylobacter jejuni strain GB4 CRISPR region	HQ378306
Campylobacter jejuni strain GB3 CRISPR region	HQ378305
Campylobacter jejuni strain GB1 CRISPR region	HQ378304
Campylobacter jejuni strain R43 CRISPR region	HQ378303
Campylobacter jejuni strain R16 CRISPR region	HQ378302
Campylobacter jejuni strain R54 CRISPR region	HQ378301
Campylobacter jejuni strain R50 CRISPR region	HQ378300
Campylobacter jejuni strain R48 CRISPR region	HQ378299
Campylobacter jejuni strain R27 CRISPR region	HQ378298
Campylobacter jejuni strain R19 CRISPR region	HQ378297
Campylobacter jejuni strain R17 CRISPR region	HQ378296
Campylobacter jejuni strain R12 CRISPR region	HQ378295
Campylobacter jejuni strain R23 CRISPR region	HQ378294
Campylobacter jejuni strain R31 CRISPR region	HQ378293
Campylobacter jejuni strain R10 CRISPR region	HQ378292
Campylobacter jejuni strain R28 CRISPR region	HQ378291
Campylobacter jejuni strain R4 CRISPR region	HQ378290
Campylobacter jejuni strain R123 CRISPR region	HQ378289
Campylobacter jejuni strain R122 CRISPR region	HQ378288
Campylobacter jejuni strain R121 CRISPR region	HQ378287
Campylobacter jejuni strain R120 CRISPR region	HQ378286
Campylobacter jejuni strain R118 CRISPR region	HQ378285
Campylobacter jejuni strain R117 CRISPR region	HQ378284
Campylobacter jejuni strain R115 CRISPR region	HQ378283
Campylobacter jejuni strain R113 CRISPR region	HQ378282
Campylobacter jejuni strain R111 CRISPR region	HQ378281
Campylobacter jejuni strain R110 CRISPR region	HQ378280
Campylobacter jejuni strain R109 CRISPR region	HQ378279
Campylobacter jejuni strain R108 CRISPR region	HQ378278
Campylobacter jejuni strain R99 CRISPR region	HQ378277
Campylobacter jejuni strain R88 CRISPR region	HQ378276
Campylobacter jejuni strain R78 CRISPR region	HQ378275
Campylobacter jejuni strain R71 CRISPR region	HQ378274
Campylobacter jejuni strain R98 CRISPR region	HQ378273
Campylobacter jejuni strain R65 CRISPR region	HQ378272
Campylobacter jejuni strain R61 CRISPR region	HQ378271
Campylobacter jejuni strain R96 CRISPR region	HQ378270
Campylobacter jejuni strain R100 CRISPR region	HQ378269
Campylobacter jejuni strain R9 CRISPR region	HQ378268
Campylobacter jejuni strain R62 CRISPR region	HQ378267
Campylobacter jejuni strain R59 CRISPR region	HQ378266
Campylobacter jejuni strain R53 CRISPR region	HQ378265
Campylobacter jejuni strain R37 CRISPR region	HQ378264
Campylobacter jejuni strain R35 CRISPR region	HQ378263
Campylobacter jejuni strain R30 CRISPR region	HQ378262
Campylobacter jejuni strain R26 CRISPR region	HQ378261
Campylobacter jejuni strain R24 CRISPR region	HQ378260
Campylobacter jejuni strain R14 CRISPR region	HQ378259
Campylobacter jejuni strain R133 CRISPR region	HQ378258
Campylobacter jejuni strain R132 CRISPR region	HQ378257
Campylobacter jejuni strain R131 CRISPR region	HQ378256
Campylobacter jejuni strain R114 CRISPR region	HQ378255
Campylobacter jejuni strain R105 CRISPR region	HQ378254
Campylobacter jejuni strain R104 CRISPR region	HQ378253
Campylobacter jejuni strain R66 CRISPR region	HQ378252
Campylobacter jejuni strain R8 CRISPR region	HQ378251
Campylobacter jejuni strain R5 CRISPR region	HQ378250
Campylobacter jejuni strain R13 CRISPR region	HQ378249
Campylobacter jejuni strain R1 CRISPR region	HQ378248
Campylobacter jejuni F001 CRISPR sequence	EF017328.1
Campylobacter jejuni F007 CRISPR sequence	EF017341.1

Campylobacter jejuni F009 CRISPR sequence	EF017327.1
Campylobacter jejuni F014 CRISPR sequence	EF017338.1
Campylobacter jejuni F025 CRISPR sequence	EF017329.1
Campylobacter jejuni F041 CRISPR sequence	EF017333.1
Campylobacter jejuni F042 CRISPR sequence	EF017331.1
Campylobacter jejuni F050 CRISPR sequence	EF017345.1
Campylobacter jejuni F053 CRISPR sequence	EF017347.1
Campylobacter jejuni F087 CRISPR sequence	EF017335.1
Campylobacter jejuni F119 CRISPR sequence	EF017346.1
Campylobacter jejuni F162 CRISPR sequence	EF017336.1
Campylobacter jejuni F226 CRISPR sequence	EF017339.1
Campylobacter jejuni F228 CRISPR sequence	EF017343.1
Campylobacter jejuni F280 CRISPR sequence	EF017337.1
Campylobacter jejuni F395 CRISPR sequence	EF017344.1
Campylobacter jejuni F458 CRISPR sequence	EF017340.1
Campylobacter jejuni F459 CRISPR sequence	EF017334.1
Campylobacter jejuni F470 CRISPR sequence	EF017324.1
Campylobacter jejuni F475 CRISPR sequence	EF017342.1

Table S5. *Campylobacter jejuni* and *Campylobacter coli* spacer alleles and prevalence in 4,232 *C,. jejuni* and *C. coli* genome sequences

Spacer allele [a]	Spacer sequence (5' → 3')	Prevalence [b]
**1**	AGGGGTGATATTGTTATGAGTATTATGCAG	3
**2**	AAAACTTGCATTTCTAGCAGTGCTTCCAAA	38
**3**	ATAGAAATTACTACAGCACAAGCTTATACA	4
**4**	AATACAGAAGTTGCATATAACTACGCAGTA	2
**5**	AAATCGTAGAGCTAAAACAATAGCACGAGA	8
**6**	GGAGAAGAAATAAATAAACAAGTTTTAAGT	6
**7**	GCTTTAGGAAATGCTTTAAAACGCTTTGGA	204
**8**	GCAGAAAATGGAGATAGACAAGATTTTCCT	23
**9**	GCATTGCTTTACTACATAGCCAGTCGTGTA	32
**10**	ACTGCTATAAGAGCAGCTACTGGTCAAGAA	9
**11**	TATGAGTTATCAAGTTTTTGTTTTTAATAA	96
**12**	TTTTTGAAACATCAACATCTTTAATCATAT	82
**13**	AAATCCGTTCGTTTTTAACATCATCAATAA	0
**14**	TGATTTATTAGTAACCCATCAAGTTGGCTT	148
**15**	CTAAATAAAGTTTATTTATAGCTTTATCA	26
**16**	AGTTTCCCTTGTGATGATGGAAATTATCGT	23
**17**	GAACCTTTAGAGTATAACGATAATCAAGTA	9
**18**	ACAAACTTAAGCCATAAAGGTATTTGACTG	1
**19**	GTTAAAAGGATTTGCTTTGCGATTGGGAA	177
**20**	GTATTTTGATACTCTGCTTCTTGGTCTTGA	97
**21**	ATTTGCAAGGTATATAAAAAATTTGATTTT	105
**22**	AAACTAATGTGTGGATTTTTGTTTAATGCT	295
**23**	GCACCTGTTTTTGATTGAACGAAGCAACCA	115
**24**	TGTGGTGTGCTTGGGTCTGTATTTATATCA	21
**25**	GAAGATGAGGTTGCGGGTTTAATAACGACG	78
**26**	AAGATTAATGTTAGATAACGAGTTGTATTC	20
**27**	ATTTTGTTTAAGTGATATTTGCAAGATTTT	20
**28**	TTCACTTACTCCATTACCTCTTCTAGCAATGCTGTAGCACT	9
**29**	TGAAAAATTAACACAATTTGATGGAGTTTA	86
**30**	ATAACTTATATATATAATTATAATAAACAT	0
**31**	CTTATTTTTACTGTAGGTAATGGAGAAGAG	5
**32**	AATAATGATATCGAAGTTATTAATCTAATT	20
**33**	TAACCTTGAAACGCTCAAAAACTCAACAGA	30
**34**	TCTCCAAATTTTAATTCAAAAGCATTAAAG	13
**35**	GAAGTTATGGAAATGGACTTTGAAGATGCT	11
**36**	CTGCTATAAGAGCAGCTACTGGTCAAGAA	0
**37**	ACTGCTATAAGAGGAGCTAATGGTCAAGAA	0
38	AAAAACCCATGAACTAGCCTTTGCAACTAA	1
39	AAAAATAAAGCTAAAGAACAGTTAGCAGTT	1
40	AAAAATAAAGCTAAAGAACAGTTAGCTGTT	2
41	AAAAATTATAAATTTTTAATTCAATACGAA	3
42	AAAACAATGGTTAATTAGTGGCAATTCTAA	1
43	AAAACTAGAACCTTCATGCCTTTTTAAACT	1
44	AAAACTTGCATTTCTAGCAGTACTTCCAAA	3
45	AAAACTTGCATTTCTAGCAGTACTTCCGAA	1
46	AAAACTTGCATTTCTTGCGGTGCTTCCAAA	8
47	AAAACTTGCATTTTTAGCAGTGCTTCCAAA	3
48	AAAAGCGATAATCTTGCTTTAGGTAGCTTA	57
49	AAAAGCGATAATCTTGCTTTAGGTAGTTTA	11
50	AAAAGGAAAATTAATGCTGAGAATTTTAAT	2
51	AAAAGTCTGTTTACTATTTGCTCCGTGGTT	1
52	AAAAGTGACAATCTTTCTTTAGGCAGTTTA	1
53	AAAAGTGATAATCTTGCTTTAGGTAGTTTA	12
54	AAAAGTGATAATCTTGTCTTAGGTAGCTTA	1
55	AAAAGTTTCTTGCGTAAAAACATTTAAGTT	24
56	AAAATAACGTATTTTGTTTTTTTGGTTCTT	2
57	AAAATAAGCCCATTTATCTATTAAAAAGCT	64
58	AAAATAATCTTTTTGTCATCAATATCAACA	5
59	AAAATACTTTAGCTATACAAAAATTAGATA	0
60	AAAATAGAATTCTTAAGAAAATATAATAAA	4
61	AAAATATCGATGATAGAATGGCTTCTACTA	6

62	AAAATATTGATGATAGAATGGCTTCAACTA	3
63	AAAATATTTAAGTATCAATGGAGTAGATTT	1
64	AAAATATTTTGGATGAGTTGAAAGAAAGCT	1
65	AAAATCCTTTAGCTATACAAAAATTAGATA	0
66	AAAATCCTTTAGCTATGCAAAAATTAGATA	1
67	AAAATCTTTAAATATCAATGGAGCAGATTT	4
68	AAAATCTTTAAATATCAATGGAGTAGATTT	25
69	AAAATTTGAGAAATGATTTAAATGAAGTA	3
70	AAACCTATTGAAGATATATGGATAGAAGAT	2
71	AAACGGCTCCAAAAGGCAAAGAGTGGGCTT	2
72	AAACGGCTCCGAAAGGCAAAGAGTGGGCAT	1
73	AAACGTGTTTCCTAATATAAACACAGACCC	1
74	AAACGTTTTTCCTGATATAAATACAGACCC	19
75	AAACTACAAGCCCATTTCATAAACTAGCTA	2
76	AAACTTGACGCTAGAAGTATAGCACTAGAA	3
77	AAACTTTTTACAGCTTTGTAGAATATATAA	10
78	AAACTTTTTACAGTTTTGTAGAATATATAA	1
79	AAAGAACTTTCAATAACTGAGGCTTATATG	52
80	AAAGACGAGCTGTGTAAAGATGATTTTATT	2
81	AAAGAGAATGCAAAATGTTTAGAAGCGATT	0
82	AAAGAGCGGGGAGAATAACGATTTAGGTTT	2
83	AAAGAGCGGGGAGAATAATGATTTAGGTTT	2
84	AAAGAGCGGTGAGAATAACGATTTAGGTTT	5
85	AAAGATGAGTGGTTTAATGAGATAGGAAAT	1
86	AAAGGTTATAAATGAAATTAGAAATTATTA	7
87	AAAGTCTTGCAATAACTTACTACCTATCAA	1
88	AAAGTTATTAAGTATGCAGTTAATCCATCA	5
89	AAAGTTTTTTGTGTATTTTCAAGACCAAGA	18
90	AAATAAAACACAATCTTTCATAATTTATCC	1
91	AAATAAGCCCATTTATCTATTAAAAAGCT	1
92	AAATAATTAATATATAATTCTATTATAATT	2
93	AAATAGAAGAGCAAAAACAATAGCACGAGA	0
94	AAATAGGCAGGTTTGATGATAGTATTAAGC	1
95	AAATCAAAAAGGGTTTTTTAATAGATATAG	1
96	AAATCAAAATTTAGCAAATAGTATTGATTT	1
97	AAATCAATTTAATAACGATTTTAATTTAGA	3
98	AAATCCCCAAAATCAGGAGAAGAAAAAGCA	1
99	AAATCGCAGGGCTAAAACAATAGCACGAGA	1
100	AAATCGTAGAGCCAAAACAATAGCACGAGA	15
101	AAATCGTAGAGCTAAAACAATAGCACGAGAGTTTTAG	2
102	AAATCGTAGAGCTAAAACAATTGCAAGAGA	1
103	AAATCGTAGAGCTAAAACTATAGCAAGAGA	168
104	AAATCGTAGGGCTAAAACAATAGCACGAGA	1
105	AAATGAAAAAAAAAGTTTAAAGATAACTTTA	5
106	AAATGAAAAAAAAAGTTTAAAGATAGCCTTA	1
107	AAATGAAAAAAAAAGTTTAAAGATAGCTTTA	281
108	AAATGTAGATAGGAATATAAGTACAATAA	12
109	AAATGTCATCAGAAGCATAACAGTTATCCA	1
110	AAATTACCTTTTGAATATATAAAAAAAGCA	1
111	AAATTTACAGCACAAAATAAAGATTTTAAT	43
112	AAATTTTCTTTAGCATAAATGGCGGCACA	0
113	AACAAGTTGACAGCATAGATAGCGATTTAG	2
114	AACAGAGTTTCAACTTAATGCGATAGTAGA	1
115	AACAGCATTTTTATAAATCTCCCTATAATA	1
116	AACAGTAGAGCCACAGGGTGCGATATGGTA	2
117	AACAGTAGAGCTACAGGGTGCGATATGGTA	1
118	AACAGTAGGGCCACAGGGTGCGATATGGTA	0
119	AACCAAAATAATCGAGAAGCCCACCTATTG	1
120	AACCGAACCAGTATAATATAACTCACCATT	2
121	AACGTCTCAAGCGGCGCATACGTCGAAATC	1
122	AACTAAATCACTATAAATATAATCGCTTTG	1
123	AACTACACGTAAAAAACGAACATTTGAAC	2
124	AACTCTGTCGACTAGCAATTGCCTGGCCCA	1
125	AACTCTTATGCTAGAGAATAAACAACAAAA	1
126	AACTTAAATGGAGTTGCTATAGGCGGTCTT	1

127	AACTTTATTATTTTTACAATATTGTAAAG	1
128	AACTTTGGAAAAGACTAAGAATGATTTAAA	9
129	AAGAAGTTGCTATTTTAAAAGAAAGAGAAC	1
130	AAGAGAAAATAAATTACCGCTTAAAGAGCT	1
131	AAGAGAACCAAGAGGAAATGCAAGGCTAGA	1
132	AAGATTAATGTTAGATAACGAGTTATATTC	3
133	AAGATTATATTCTGTGGCAATATAACTAAA	1
134	AAGATTGAAATAGTAGCTAAGAATAAAATA	15
135	AAGCATTGGCAACAGGTTTGGAGTTTCTCT	2
136	AAGCCAAATCCATAAGACTACCGCTTTTAA	0
137	AAGCGAAAGCTAGAAATAATCAAAAAAGCT	1
138	AAGCGATATGTTATTAAGATTTAAAAGTGA	28
139	AAGCGATATGTTATTAAGGTTTAAAAGTGA	1
140	AAGCGGATCAGAAGTAGTATCAGCAAAAAT	1
141	AAGCTTTTTTCCATAATTATCTAAAACAT	4
142	AAGGGCTATTTCATAAAAGCCAAGTAGGGT	12
143	AAGTATATTTTTAGATAATTTGAATATAAA	8
144	AAGTCAATTTAATAACGATTTTAATTTAGA	17
145	AAGTCTAAACCCCAAATAATCAAGTGATAA	1
146	AAGTCTTTAGCACTTGATAATGGCTTTTGA	2
147	AAGTGCTATAGTAGATAGCTATGGAAATA	1
148	AAGTTTTATTGTCAATAACGCCTTTACTAA	2
149	AATAATGATATTGAAGTTATTAATCTAATT	5
150	AATAATGATATTGAAGTTATTAATTTAGTT	1
151	AATAATTTCTAATTTCATTTGTAACCTTTA	1
152	AATACTTTAAGAAGTATTACAAAAAAAGAT	9
153	AATAGTAAAGTTTTATCAATACTTAAAGAA	1
154	AATATTACAAGGGGTTATTTTAAATGCGTA	0
155	AATCAATTTACATCATTAAAAAATAGTATA	1
156	AATCACACCTTTTAAATTAAACATGGTATC	1
157	AATCCCAATAATTTAACAGCAGGCATTCTA	2
158	AATCCTAATAATTTAACAGCAGGCATTCTA	39
159	AATCGCCCTTTTGATGAGTTGGTTAAAATT	0
160	AATGAGTTATCAAGTTTTTGTATTTAGCAG	0
161	AATGCAAAAAAATTTATAGTGAAATTACAA	1
162	AATGCAAAATGTTTAGAAGCGATTTACAAC	2
163	AATGCAAAATGTTTAGAAGTGATTTACAAC	2
164	AATGCAAAATGTTTAGAAGTGATTTACAGC	2
165	AATGGTAAATCTAATATTAGATGTGTCTTA	1
166	AATGGTTTTCCTGTTGAAACTGAAACTAGT	1
167	AATTACAAAATTTCTAAATCGGTTATAATA	1
168	AATTACAAAATTTCTCAATCGGTTATAATA	1
169	AATTATAAAATTTCTCAATCAGTTATAATA	0
170	AATTCTAAAAAAGCAGGGTATGCTAGTAAA	3
171	AATTGCACGACTTGATGGCACATCATCATT	2
172	AATTGCTAGTCAATTAGGCTTAGATGACGC	109
173	AATTGCTAGTCAATTAGGTTTAGACGATGC	1
174	AATTGCTAGTCAATTAGGTTTAGATGACGC	2
175	AATTGCTAGTCAATTAGGTTTAGATGATGC	1
176	AATTGCTAGTGAAATTCAAGGCGTAGAATA	1
177	AATTTAGCTTTTAGCTAAAAGCTAAATTC	1
178	AATTTGATGGAGTTTATAGAATAAAAGAAT	1
179	AATTTGCAAAAATTTAAAAGAATAGTTTTT	4
180	AATTTTAATACACTGGGGAAACACTGACAA	2
181	AATTTTAATACACTGGGGAAACACTGATAA	3
182	AATTTTGAAGAATCCCCTGTTAAAGTATTT	1
183	AATTTTGCTTTATTTAAATTGTTTTCGCTA	61
184	AATTTTTGAAATAAATAAAGTCAAACATAG	2
185	ACAAAAAAAGAATGTTTAAAATGGTATGAT	1
186	ACAAAAGCGGATTTGCTTAATATATACACT	1
187	ACAACACAAGTCCTTTTCATAGCTTAAGCA	1
188	ACAACAGAAGATGATAATGGAGTTAGATTG	17
189	ACAAGACAATTTATTAAAAATAGTAATGAA	2
190	ACAAGACAGTTTATTAAAAATAGTAATGAA	1
191	ACAATTTCTAATTTCATTTATAACCTTTTA	1

192	ACACAACACAAGTCCATTTCATAGCTTAAG	2
193	ACACTCAGCTGTATTACAACGAGACGAACA	1
194	ACAGTGAAAACAGATACACTAGATCCTGTG	2
195	ACAGTTATATACTAAACAGACTGTTCTAAG	1
196	ACATAATCACTTATTTGCAACTCTCCTTCA	10
197	ACATGCTTATAGGTTGAACCGCCATTAAAC	1
198	ACATTTACACAACAAAATATAGGCATGCCT	3
199	ACCAAAAAGTGATAATCTTGCCTTAGGTAG	1
200	ACCAAAAAGTGATAATCTTGCTTTTGGTAG	0
201	ACCAAATTCTAAACTTTGTCTATCAAGTTT	64
202	ACCAATTTTCTACTACTAATAGCTTCAAAC	1
203	ACCACCATAACAATTAATAAAATATTGAGT	1
204	ACCAGAAAGAGTAGTATCAGGACAATTCGG	1
205	ACCCGCGGCTGCGCGAAATTTAAACGCCAG	1
206	ACCGCTATAAGAGGAGCTACTGGTCAAGAA	1
207	ACCTAAATGTGGTGCAGGTTTAGCAGTTGT	2
208	ACCTCCCCTGTTGATGATGGAAGCTATAGA	1
209	ACCTCCCCTGTTGATGATGGCAGTTATAGA	1
210	ACCTCCCCTGTTGATGATGGTAGTTATAGA	7
211	ACCTCTCCAGTTGATGATGGTAGCTATAGG	5
212	ACCTTTTTTAGCTGTGATTTCTGTTTTAAT	5
213	ACTATTATCGTTTGCTTTTGTAAGTCCTAA	1
214	ACTATTTTAAAAAGAGTTCGTAAAAATCA	2
215	ACTCGTTCCAAATATTCCAATAAGCCATCA	1
216	ACTGCTATAAGAGGAGCTACTGGTCAAGAA	118
217	ACTGCTATAAGAGGAGCTGCTGGTCAAGAA	1
218	ACTTAAATCAATTTCAAATTTATCACAATT	37
219	ACTTAAATTAATTTCGAATTTATCGCAATT	1
220	ACTTAAGATATTCATTTGCGTTAATAAATC	2
221	ACTTACAATATTAATTATACCTCTGTTGGA	12
222	ACTTACACTTTTTATTATTGGTGCAAATAT	12
223	ACTTCACCTGTTGATGATGGTAGTTATAGA	1
224	ACTTCCCCTGTTGATGATGGTAGTTATAGA	13
225	ACTTCCCCTGTTGATGATGGTAGTTATAGG	1
226	ACTTGCTCCATCAGTTTTATACCCTTTTTC	2
227	ACTTTAGGAAATGCTTTAAAACGCTTTGGA	0
228	ACTTTCCCAATCCTCAAAAAGTATCCTATA	1
229	ACTTTCTCGCAATATGGTAAAGTAGTTGTA	14
230	ACTTTCTCGCAATATGGTAAAGTGGTGATA	1
231	ACTTTCTCGCAATATGGTAAAGTGGTTATA	2
232	ACTTTTAAATGGTATCACTTGTAACAATAT	1
233	ACTTTTTCACAATATGGCAAAGTAGTTGTA	5
234	ACTTTTTCACAATATGGTAAAGTAGTGGTT	39
235	ACTTTTTCACAATATGGTAAAGTAGTTGTA	56
236	ACTTTTTCGCAATATGGTAAAGTAGTGGTT	1
237	AGAAAAAAATTGGTTTGTAGACCTTCCGAAT	2
238	AGAAAAACCTTGTATAAATAGGTGTACGAA	1
239	AGAAAAGTTCCTAGCTTGGATGAATCCTGA	3
240	AGAAACATAATTTTTCATAAATTGACAAAG	0
241	AGAAACTATATTAGCAGGCATAAATAATCT	6
242	AGAAACTATATTAGCAGGTATAAATAACCT	25
243	AGAAACTATATTAGCAGGTATAAATAATCT	1
244	AGAACCAACAGGCAACACCTACAGCAACA	9
245	AGAACCTAAGTCATGCTTGCTATTTCCTAA	2
246	AGAACCTTTAGAGTATAACGATAATCAAGTT	0
247	AGAACTAACTTTCCACTTTTTACCTTGATA	2
248	AGAATACCAGAAGGCATTTATAATTTAGAA	6
249	AGAATACCTGAAGGAAATTATAATTTAAAA	3
250	AGAATACCTGAAGGAAATTATAATTTAAGA	0
251	AGAATGAGTTTATATAGCCCATAAAAGCTC	1
252	AGAATGTGTTTTATTTTCAAAAGAAAATTT	1
253	AGAATTTTGATACATTGGGGAAATACAGAC	1
254	AGAGAATTAACTTTAATTGATGCTTACGTC	1
255	AGAGCAAGCGTTCGAATTTTTACAATCAAA	1
256	AGAGCCTTTAGAAAATAATATTTTTATAATA	1

257	AGAGGTGATATAATTTCAAGTATTATGCAA	1
258	AGAGGTGATATTGTTATGAGTATTATGCAG	0
259	AGATAAAGTTTAAGTAGGTTAAAGTTTACA	1
260	AGATAAATTAGAAATAGAAAAATACAATTG	54
261	AGATGCTTTAACAGTATGTTCTTTAGCAGG	93
262	AGATGCTTTAACTGTAACTTCTTTAGCAGG	1
263	AGATTAAAATAGAAAGTAAAAGAAAATGAC	1
264	AGATTGAAGTGTGGTAAAGGCTGTAGCACT	4
265	AGATTTCCATCTATCCCATTTAAATGTATT	0
266	AGATTTTTCATTTAAATGATGATGAGTTAA	9
267	AGCAAAATTAATAACATTAGAAAGTGAAAC	2
268	AGCACCGGAACCACCTAAAGCAAAAAGAGG	0
269	AGCACTGAAATAAATGAGTTTATAGCAAAT	0
270	AGCAGAAGTTAAAGTAAGTGCCACTGGTGC	2
271	AGCAGAAGTTAATGTAAGTGCCACTGGTGC	2
272	AGCATAAGCTTGATGCTTTAATGGAGTCGT	3
273	AGCATTATAGTTAAAATTATTAATAGAAG	2
274	AGCTAAATTTTCATTAACTCTATTAATATA	200
275	AGCTAAATTTTCATTAATCCTGTTAATATA	1
276	AGCTAAATTTTCATTAATTATGCTAATATA	1
277	AGCTGAACCACTAATATTGGAGCAATTACC	1
278	AGCTTTTAAGATTTTATATACACCTGAGCT	17
279	AGCTTTTTGTATTGCGACTTATCTAATTTA	1
280	AGGACCTACGCTCATTCTAGTGTTATAAAC	1
281	AGGATATTTTGCTCAATCTGATGATGGCAC	2
282	AGGCGGTTTAATTAAACTTAAAAATGAAAA	4
283	AGGCGGTTTGATTAAACTTAAAAATGAAAA	13
284	AGGGGAGATATTGTTATGAGTATTATGCAG	57
285	AGGGGCGATATTGTTATGAGTATTATGCAA	6
286	AGGGGCGATATTGTTATGAGTATTATGCGA	2
287	AGGGGTGATATTGTTATGAGCATAATGCAA	2
288	AGGGGTGATATTGTTATGAGTATTATGAAA	21
289	AGGGGTGATATTGTTATGAGTATTATGCAA	116
290	AGGGGTGATATTGTTATGAGTATTATGCGA	15
291	AGGGGTGATATTGTTGTGAGCATTATGCAA	2
292	AGGGGTGATATTGTTGTGAGTATTACGAAG	2
293	AGGGTTTCAAGTAGGTTTAAACAAGGCGGA	1
294	AGGTTATGGATATGGATATAGAAGATGCA	0
295	AGTAACTCTGACCAGAAGTAGAATTAGGAT	2
296	AGTAGTGTTATCAATAATGCAAACGCAAAT	7
297	AGTAGTGTTATCAATAATGCAAATGCAAAT	1
298	AGTATTAAAGATGATATTGTTAGTTTTGGT	3
299	AGTATTAAGCTTACCTATTGTAAAAGCGTC	1
300	AGTATTCATATCATCATTTAATTTATTAAA	1
301	AGTGAAGTTGTTGGAAATATACATAATAGA	3
302	AGTGGTGAAAGTGGTAAAAATTCTGGATTT	1
303	AGTGTGTATGTTGAAATAAATTATAGAGTA	2
304	AGTTAAAACGCCAGGACCGTATATATTTGT	1
305	AGTTATTGAAAATGATATAACTATTTACAA	3
306	AGTTATTTGTTAGGGGGCTAAACCCCCGCG	1
307	AGTTGATTTAGTTGAGATTGATAGCGTAGA	1
308	AGTTGCGCCAAGGGAGCGTAATCCCGCTCT	1
309	AGTTTAAAGATAGCCTTATGAAACACATTA	1
310	AGTTTATAAGAATAAGCAAGATGTAGAAT	1
311	AGTTTTATTATAGCGAGACATAGCCCACCA	5
312	AGTTTTATTATAGCGAGCCATAGACCACCA	2
313	AGTTTTGGCTTAGGGGGCTAAACCCCCGCG	1
314	AGTTTTGGGCTAGGGGGCTAAACCCCCGCG	2
315	ATAACTTATATATATAATTATAACAAACAT	0
316	ATAAGAGTTTGCTTGATATTTAAATTATCT	3
317	ATAAGCACTTCTATTAAGTTGAGCTGCACT	1
318	ATAATAATCTTTCTCTAATGCAAAACTTGA	4
319	ATAATACCAAATAAAAAAGTAAGTTTTGAT	1
320	ATAATACCAAGTAATTATGAAAGTGAAGTT	1
321	ATAATACCTAATAAAAAAGTAAGTTTTGAT	1

322	ATAATTCCTAATAAAAAAGTAAGTTTTGAT	13
323	ATAATTTCTAATTTCATTTATAACCTTTCA	97
324	ATACAAATAATCATCTTTGTTTTGTGGATT	1
325	ATACATACCAAAATAATCCATAAGAGAACC	2
326	ATAGAAATTACAACAGCGCAAGCTTATACA	1
327	ATAGAAATTACTACAGCACAAGCTTATGCA	1
328	ATAGAAATTACTACAGCGCAAGCTTATACA	6
329	ATAGAAATTTTATAGGTTGCGAAATAGACA	1
330	ATAGACAAAGAAAAGAGCTTAAAAAAATAA	2
331	ATAGCGATGGTAATTGGCTTGAAAATATAG	4
332	ATAGGGCTATCCGCTTCGCTCTCAGCTTC	1
333	ATAGTGATGGCAATTGGCTTGAAAATATAG	7
334	ATAGTGATGGTAATTGGCTTGAAAATATAG	13
335	ATAGTTTATATAACTCCATCAAGTCTTATT	1
336	ATAGTTTATATAACTCCTTCAAGTCTTATT	1
337	ATATAATATTGATAAGGCATATTATCACGC	1
338	ATATAATATTTAAAATTTGCTATTGTTTTG	102
339	ATATAATATTTAAAATTTGCTATTGTTTTT	1
340	ATATAATCACTTATTTGCAACTCTCCTTCA	1
341	ATATATAAATTTGGATATGAAGCTTTAAGC	15
342	ATATATAAATTTGGATATGAAGCTTTAAGT	1
343	ATATATAAATTTGGTTATGAAGCTTTAAGT	1
344	ATATATTGAAGATAGCTTTAATATGAGTTA	1
345	ATATCCATCTTTCATATCTTTGATTTTAAC	5
346	ATATCCATCTTTCATATCTTTGATTTTAAT	1
347	ATATTACAAGAGGATATTTTAAGTGTATAG	1
348	ATATTACAAGGGGTTATTTTAAATGCGTA	0
349	ATATTACAAGGGGTTATTTTAAATGTGTA	0
350	ATATTCTGCAAATACTCAATATAACGCATA	2
351	ATATTTTATAGAAGGTGGAGCAAATTTGAA	2
352	ATCAAAATTTGATATTCATGAAGTGATTAA	1
353	ATCACAATTTCTATTATTTTCGTAATCAAT	4
354	ATCACAGGCGCAAATTCGCAGCAAGTAGCT	1
355	ATCACGATTTCTATTATTTTCGTAATCAAC	1
356	ATCAGTCTTAGCTTTATTATTATTAGCCAC	1
357	ATCATCACCACCACAATCACAAAAAAGTAC	1
358	ATCATCATCACTTAAAACCTTAAATTTACC	1
359	ATCATCATCACTTAAAATCTTAAATTTACC	1
360	ATCATTTTTTAAAGGGATATCACTATCATT	1
361	ATCGAGAAGACTTTGTATATAGGTGTATAT	2
362	ATCGCTTAAAACTTTAATAACTGTGTAGAT	2
363	ATCTGGGACACTTTAGATAAGGATGAGTT	1
364	ATCTTATCAAGTTTAAACGCTCAAATAGTT	1
365	ATCTTCATTTTCTCCACCATCTACAACTAG	1
366	ATGAGAATAGCAAAAGCCCAAGCGGTGTGG	2
367	ATGAGTGTGCTAAAAAAAAATGGACTTAAAT	3
368	ATGCAAAATGTTTAGAAGTGATTTACAGC	1
369	ATGGAATATCCAAAGTTTTGCCCGGTATCA	1
370	ATGGAATATCTAAAGTTTTACCCGGAACTA	1
371	ATGGCGAACGCACCTGTTAAAAAAATCGTA	1
372	ATGGCTAGTGCATTACCTTTAGACTTGTTG	4
373	ATGTTACATCTATTTGTAGCATTTACTTGA	1
374	ATTAATGCTAGTTCTATAGAGAACCTTATG	1
375	ATTACAACCCATTCTTTGTTTTCTATATCA	1
376	ATTACCTAATTCATAATCAAAAACAAGAGG	1
377	ATTAGGTGCTAAATTTATTGATATGGTTGG	0
378	ATTATAAGTTTTTTTATAGAAAAAGGCGTA	2
379	ATTATCTTCATAAAGACTTTTTAAAGCATT	1
380	ATTATTACTTACTTAAGGATTAAAAATGAC	7
381	ATTATTTGAAAGATATTAATGATGAAAAAC	1
382	ATTCGAGTATTGTAAACTACAGAGCCCAGG	1
383	ATTGAAATTAACATTCATACCAGCAAGTAA	182
384	ATTGAAATTACTACAGCACAAGCTTATACA	3
385	ATTGCTAGTCAATTAGGTTTAGATGACGC	4
386	ATTGTTAATTTTCTTATTTAAGGATATACC	1

387	ATTTAATGTTGTGTAAAGCGATAATGGTAT	29
388	ATTTAATGTTGTGTAAAGTGATAATGGTAT	2
389	ATTTAGCAGATAGTATTGAATTCTTAATAC	0
390	ATTTAGCAGATAGTATTGAATTCTTAATGC	1
391	ATTTAGCTGATACTATCGAATTAATCGTAA	11
392	ATTTATAAATTTGGATATGAAGCTTTAAGT	1
393	ATTTATAAATTTGGATATGAGGCTTTAAGT	0
394	ATTTATATAACGACCATTTCCAAAATTTTA	5
395	ATTTATGTAATTTTTATAATCAAAGCATTA	6
396	ATTTGTAATGTATATAAAAAATTTGATTTT	3
397	ATTTGTTGAGTGGGTTATGAGCCAAAAGAA	0
398	ATTTTTCAATGTAATGTTTCTAAAATTTGG	1
399	CAAAACATGAAACTTCAGAACCAACTTTTA	1
400	CAAACACTAGATGATTTAGTATTTGTGTAT	2
401	CAAACCCTTCTCCACCCTTTTGGAAGGGTG	1
402	CAAACCTTTATACAAAACTTCCTTTAACAGAA	1
403	CAAAGAAGGTTTAGAAAGTGGTAAAGATTT	3
404	CAAAGTGCTGAAAAAGATAGAAACTTACTT	1
405	CAAATATTTTAGAATAATTTATCAAAAATG	5
406	CAAATGTTAAAACAATACGAGCAAATGATT	17
407	CAACCTTATTATTTTTACAATATTGTAAAG	2
408	CAACTGGTAGCACTTTAACAACTACAGAA	1
409	CAAGAATATATAAATAATAATATAAAAGAA	2
410	CAAGCGATAGTATTATAAATAATGCAATAA	6
411	CAAGTGATAGTATTATAAATAATGCAATAA	4
412	CAAGTGCTATAGTAGATAATTATGGAAATA	1
413	CAAGTGCTATAGTAGATAGCTATGGAAATA	1
414	CAAGTGCTATAGTAGATAGTTATGGAAATA	3
415	CAATAAAGCGTTTAATGAAAAAGACGAGTA	1
416	CAATAGTTGTTAAAAATGGTGATGATATTA	6
417	CAATATAATTACAAAATTTCTCAATCGGT	19
418	CAATTTTAAATTCTTTTCATAGTTCTCAAA	4
419	CAATTTTAATACACTGGGGAAACACTGAC	1
420	CAATTTTAATACACTGGGGAAACACTGAT	0
421	CAATTTTTAATTTTTGAACCAATATTGCCA	2
422	CACACCTTTTAAATTAAACATGGTATCATA	7
423	CACACTAACATCACCAACACTACTGCTTGC	1
424	CACCCGTAGCACTATCCCATCCGTTAAAAT	3
425	CACCGCTTTATATTCATTTTCAAAATCTAA	3
426	CACGAGATACTAATGCTAATATGTTAGGTG	15
427	CACGAGATACTAATGCTAGTATGTTAGGTG	3
428	CACTCATCTTTAAATCTTGAAGCCCAAGTT	0
429	CACTCATCTTTAAATCTTGTAGCCCAAGTT	3
430	CACTCCTTTTAAATTAAAAATGGTACTATA	3
431	CACTCCTTTTAAATTAAAAATGGTATTATA	4
432	CACTCGTCCTTAAATCTTGAAGCCCAAGTT	1
433	CACTGAAGATGTAGGTAGAAGAATGGAAAG	1
434	CAGAATGTATTGAAAAAATAGAATTCTTAA	1
435	CAGAGATAACGAGTTTAATTACACTAGAGA	1
436	CAGATGCGGGTTGGAGCGTGAGTGTTTTTG	1
437	CAGATTAATGTTAGATAACGAGTTATATTC	1
438	CATACCAAGGTATTCCCATTGTTAAATCAA	1
439	CATATTTTAATCCATTGGGGAAACGCTTAT	0
440	CATGACAGCTTTATGGTTTGAATCTCAAAA	1
441	CATTATTTATAATACTATCACTTGCTAAAA	3
442	CATTCCTTTTAAATTAAAAATGGTATTATA	1
443	CATTCTGGCTCCAGTCTTCTTTACTAAAAA	3
444	CATTGGCATCACTTTGAACAACTGGTGACG	1
445	CCAAAAAGTGATAATCTTGCTTTTGGTAG	1
446	CCACCAAGCTCGATTAATTCTTCTAAAGTA	0
447	CCACCAAGTTCTATTAATTCTTGTAAAGTA	1
448	CCACCTACTAGAATACAACTAAGAATAGGG	0
449	CCACTTGATAGAAGAATTTTGATACATTGG	0
450	CCAGCAAGCCGTGCAATTTTAATACACTGG	3
451	CCAGCAAGTCGTGCAATTTTAATACACTGG	86

452	CCAGTTTCTGCTAAATTAAATGCTGAACT	2
453	CCATCAAGTCGTGCAATTTTAATACACTGG	3
454	CCATCAAGTCGTGCAATTTTATTACATTGG	0
455	CCATTCTCATAAAATATTTAGCCATTATT	24
456	CCCGTAAATAGAGCTCAGGTTGCATCAAGT	1
457	CCCTTTTATAATTTATGAAATTGCGTGAA	64
458	CCGTCAGGGCTTAGGGGTAAGTAACCCCGC	2
459	CCGTGCTCTTCGAAAAACCTAGTAAATCTA	2
460	CCTAACATACTAGCATTAGTATCTCGTGCA	1
461	CCTAACATACTAGCATTAGTATCTCGTGCG	2
462	CCTAACATATTAGCATTAGTATCTCGTGCG	14
463	CCTCTTAAATTAGAATTACAATTAGTCAAT	0
464	CCTGGAGTAACTAGATTTGCAAATATCGCA	0
465	CCTGGAGTAACTAGCTTTGCAAATATAGCA	5
466	CCTGTTATTTCACTTAAATGCCTTGCGGTT	11
467	CCTTATGAAAGACACATTTTAATACACTGG	1
468	CCTTCAAGTCGTGCAATTTTAATACACTGG	7
469	CGAAATCGCTAAAATTTAAACTGCGATGAG	1
470	CGACCCAATATTTTTCGTTGCATTATCTAC	1
471	CGATAGTTTGCTTTTATGATGATAAAAAGG	1
472	CGATTTTAGATGAATATGAAAGTTCTTTG	1
473	CGCAAATAGTAACAGTTGTTAACGGAGTTG	1
474	CGCAACTGGTAGCACTTTAACAACTACAGAA	15
475	CGCGTCAAATTCAATTCCATCAGGGACTAT	0
476	CGCTTTTACAATAGGTAAGCTTAATACTAT	2
477	CGTAATTTCTTAACATCACGAAGGTACAAT	2
478	CGTATTTACTATTAATACCTTCACAAACGA	1
479	CGTGAGGTACTTTTTTCTAAACAAGTTGCG	1
480	CGTGGTATTATTTTTGCTTAAGGGCGAAAT	4
481	CTAAAAAATCCAAAAATTAAATTAATTAAA	0
482	CTAAAGTTAGAAATTGAATCTATTAACCAT	1
483	CTAAATGCTGGTAGTGTTTTTATTGAAACA	0
484	CTAACATACTAGCATTAGTATCTCGTGCG	1
485	CTAAGCAATCTTATTTTACCATCTTTTTTA	1
486	CTAATACCTAAAAAGTTGAATAAAGGCTTA	8
487	CTACAAGAATGAGGATGATGATATTTTACA	487
488	CTACAAGAATGAGGATGATGATATTTTACG	2
489	CTACAAGACAAGTTATAAAAAATGAGAGTG	1
490	CTACCCCTATCCACAACCGCCAAGTGATTA	7
491	CTACCCCTATCCACAACCGCCAAGTGATTG	1
492	CTACGCATTCTGCGAAACCTACGCCCGCGA	1
493	CTATATTTTATTTTTTCTTTATCATTGAAA	1
494	CTATCTGCATAAATACTAAGTGCCATAAG	15
495	CTATTATAGTGGATAATTACGACCCTGAT	4
496	CTATTCTCCAATCGTTGAAAGAACAGGCCA	1
497	CTATTCTCCAATCGTTGAAAGAACAGGGCA	0
498	CTATTCTCCAATTGTTGAAAGAACAGGCCA	1
499	CTCAACTTTTCTAAAAACCAAAAACCTATG	1
500	CTCATAGGAAGTTTTAATCTTGGTAACTCA	4
501	CTCTAATGGATTATACGTTCTTCAATCAGG	2
502	CTCTTCTCCATCTTCTTTTAATAAAACATT	0
503	CTGCCATCTCATTTTCAAATAAAGCACTAA	19
504	CTGGAGTTTAAACGGGTGGATTATGGTTAT	1
505	CTGGATTTCTGTAATTTTGAATAATAAATT	2
506	CTGTACGACTTTCAATCTCTTTAATTGTTTG	1
507	CTGTAGAGAGTGTGCTTGTTAGAATATAGA	1
508	CTGTTTTACAATTTTCTAATAGCAATCCTA	1
509	CTGTTTTATTGTCAATAACACCTTTGCTAA	1
510	CTGTTTTATTGTCAATAACGCCTTTACTGA	1
511	CTTAACGCTGGTAGCATTTTTATTGAAACA	1
512	CTTAACGCTGGTAGTATTTTCATTGAAACA	1
513	CTTAACGCTGGTAGTATTTTTATTGAAACA	8
514	CTTAACGCTGGTAGTGTTTTTATTGAAACA	7
515	CTTAATAGAAGTGCTTATAATAATTCAAGT	1
516	CTTAATTTCGGTGCATCACGTAAAAATAAT	1

517	CTTAGTGGAATTATGGCAAATTCTGTTAAT	1
518	CTTATACAAGTTGAATGCGGTGTGAGTGAT	6
519	CTTCTCAACTAATGAAAGTTTAGGTAAGGA	3
520	CTTCTCAACTAATGAGAGTTTAGGCAAGGA	1
521	CTTGATTAAGTTTCATATTGTTTAAAATCT	1
522	CTTGGCGGAATTATGGCAAATTCTGTTAAT	2
523	CTTGGCGGTATTATGGCAAATTCTGTTAAT	5
524	CTTGGCGGTATTATGGCAAATTTAGTCAAC	1
525	CTTGGTGGTATTATGGCAAATTCTGTTAAT	11
526	CTTGGTGGTGCTATTTCTAGTGATTTGATA	2
527	CTTGTGATGATGGAAATTATCGTGGAGATA	2
528	CTTTATGTAATTTTTATAATCAAAGCATCA	4
529	CTTTATGTAATTTTTATAATCAAAGCATTA	1
530	CTTTATTATTTTATAACCACTAATGAAAAC	2
531	CTTTTAAGCTTATACTTATAAAAAAAGTAT	1
532	CTTTTATCTTTTATAGTATTAGGGCTAATA	1
533	CTTTTTCACAATATGGTAAAGTAGTGGTT	1
534	CTTTTTCACAATATGGTAAAGTAGTTGTA	5
535	CTTTTTTATTTTATAACCACAAATAAAAAC	1
536	GAAAATAGTATAAAACTTGAGAGAGATTTA	1
537	GAAAATGGATATTTTTATGCAGTTAGCGGG	4
538	GAAACAATGGTTAATTAGTGGAAATTCTAA	1
539	GAAACAATGGTTAATTAGTGGCAATTCTAA	1
540	GAAACTAGCATAAAACTTGAAAGAGATTTG	1
541	GAAACTAGTATAAAACTTGAGAGAGATTTA	94
542	GAAATAAGCCCATTTATCTATTAAAAAGCT	2
543	GAAATACCAAATCAAATTCAAGCACAATAT	7
544	GAAATAGCAGAGCGTAGATTAAAATCAACT	2
545	GAAATGATTATTAGTATCTTAAATGAAAGC	4
546	GAAATGGTTATTAGTATCTTAAATGAAAGG	1
547	GAAATTCATAATATAGAATATGAGGCGAA	1
548	GAACCTTTAGAGTATAACGATAATCAAGTT	24
549	GAACCTTTAGAGTATAACGATAATCAGGT	2
550	GAACCTTTAGAGTATAACGATAATCAGGTT	1
551	GAACCTTTAGAGTATAATGATAATCAAGTA	5
552	GAAGGTTTAAGACCTATTGTTATGGGTATT	6
553	GAATCTTTAGTTAACACGATACAAAGAGGT	1
554	GAATTATTGGAAATAGAATGTGATTTTATT	1
555	GAATTTTGATACATTGGGGAAATAGCCAC	0
556	GAATTTTGATACATTGGGGAAATAGCCACA	0
557	GACTTTTTACTAATTTTGTAGAATATATTA	1
558	GAGCCCTTAGAAAATAATATTTTTATAATA	4
559	GAGCCTTTAGAAAATAATATTTTTATAATA	8
560	GAGCCTTTAGAGTATAACGATAATCAAGTA	2
561	GAGCCTTTAGAGTATAACGATAATCAAGTT	5
562	GAGGTTATGGATATGGATATAGAAGATGCA	2
563	GATGAAGATGAAGTTGAAGCTAAACAAGAT	4
564	GATGAGGATGCAGTTGAAAATAAAGAAGAT	1
565	GATGATGATGAGATAGAGGCTAAACAAGAT	2
566	GATTGGAATTTAGATGAGATTAGAGAAATA	33
567	GATTTAAAAGATATCTTTAACGCATTAATA	5
568	GATTTATCTAATGTAATTATAACATCAGAA	2
569	GATTTATTAGTAACCCATCAAGTTGGCTT	31
570	GATTTGAAACCGATAATAAAGAATGGAATG	1
571	GATTTTGAAAGACATATTTTGATACATTGG	0
572	GATTTTTATTATACAATTGAAAATTTACAA	4
573	GCAAAATGCTAATATAAAACCTATTATCGA	1
574	GCAAACTACACGAACAAAACGAACCTGAGA	2
575	GCAACTGGTAGCACTTTAACAACTACAGAA	2
576	GCAACTGGTAGCACTTTAACAACTACAGAG	1
577	GCAACTGGTAGCACTTTAACAACTACGGAA	0
578	GCAACTGGTAGCACTTTGACAACTACAGAG	18
579	GCAAGTGCTAATGCTAGTGCAGTTCATATT	11
580	GCAATTTTAATACACTGGGGAAACACTGAC	0
581	GCAATTTTAATACATTGGGGAAACACTGAC	1

582	GCAATTTTAATGCATTGGGGAAACACTGAT	2
583	GCACCATCTGCTTGATTATCTTCTTCTCCT	2
584	GCACCTGTTTTACTTTGTATAAAGCACCCT	1
585	GCACCTGTTTTTGATTGAACAAAACAACCA	51
586	GCACCTGTTTTTGATTGAACAAAGCAACCA	6
587	GCACCTGTTTTTGATTGAATGAAGCAACCA	2
588	GCACCTTTAGAGTATAACGATAATCAAGTT	3
589	GCACTTGTTTTTGATTGAACGAAGCAGCCA	0
590	GCAGGTATTTACTGGCTTATTTTGAGATAA	3
591	GCATAAATCTTTTAAAGGATAATTTCTTATA	3
592	GCATCAAACGCTATTGATTGCAATATAGCT	9
593	GCATTATTTATAATACTATCACTTGCTAA	1
594	GCATTGCTTTGCTACATAGCCAGTCGTGTA	1
595	GCCACCGAACCATCAGGAGACACCTCAAG	1
596	GCCCCTGCTTTTGATTGAACAAAGCAACCA	0
597	GCCCCTGCTTTTGATTGAACAAAGCAGCCA	178
598	GCCCCTGCTTTTGATTGAACGAAACAACCA	1
599	GCCTAGTTTTACAGAACAAGAGCAAAATTT	1
600	GCGATTATGTATACGAAAAAAGTATTCTAG	1
601	GCTACAAAGAGTAACCTCGCCCTATGAGAA	1
602	GCTATATTTGCAAAACTAGATACTCCAGGG	0
603	GCTTAGGGGTAAGTAACCCCGCGTCGGAAA	1
604	GCTTATACTTTAAAACTAAGTGATTTTTGC	1
605	GCTTGGGCGTGATTTATTTGTTCTAATCTT	1
606	GCTTTAGGAAATGCTTTAAAACGCTTCGGT	3
607	GGAAAACATAATATAACGTTAAAAGATAAA	3
608	GGAAAGATAGGGGAATTTTAAGGGGCTTAG	1
609	GGAGAAGAAGACAATAAAGCAGATGGTGCT	1
610	GGAGAATTGTTTCAAAGATGTGTTTATTTT	3
611	GGAGATGTTTTTAATTGCACAAAGCAACCT	2
612	GGAGGCCGGGCATAGGTTTTGGATTTTTGG	2
613	GGCACTCTTTTAATATTATAAACTGGACTT	1
614	GGCTTCAAGATTTAAAGATGAGTGGTTTAA	1
615	GGGACACGAGGAATCCTGTCTGAATCCGGG	1
616	GGGTGATATTGTTATGAGTATTATGCAGAC	3
617	GGTAAGAAGCAAGTTTGATTTTCACGAGTT	1
618	GGTAGCAGAATTATCTTTGCTGGTATCAAT	4
619	GGTAGCAGAATTATCTTTGCTGGTATTAAT	1
620	GGTAGTAAGATTATTTTCACTGGGATAAAT	3
621	GGTAGTAGAATTATCTTTGCTGGTATCAAT	4
622	GGTAGTAGAATTATCTTTGCTGGTATTAAT	4
623	GTAAAATTGTAACTTCTGTATCACCTACCA	2
624	GTAACACAAGTCCATTTCATAGCCTAAGTA	1
625	GTAACTATTGTAAATGGAGTTGAAAGAGTT	39
626	GTAACTATTGTAAATGGGGTTGAAAGAGTT	8
627	GTACCCTTATTTAAACCATTTTGGTCCTTC	1
628	GTAGGAGATGTGCCTTTAAAAGAGTTTGCA	7
629	GTATATTCTTTTAAAATATGTTTAAATGGA	4
630	GTATATTTTCAAGACCAAGAAGCAGAATAT	1
631	GTATTAAAAACTCTATTAATAATCTAGGAG	1
632	GTCTTTGTCTTTCTAATTCCCATTCATTCA	1
633	GTGCGTTATACACGACTGGCTGTGTAGTAA	6
634	GTGTATTTTCAAGACCAAGAAGCAGAATAT	1
635	GTGTTGCTTATGGTCTAGTTCAGGAAGTAG	4
636	GTTAAAATCTTTTGAAACAATAGAATATTT	0
637	GTTAACTCTTCTACAATGGGAGCAAATTCA	4
638	GTTATCGCGGGCGCTGGAACCGTATAGGTT	1
639	GTTGAAGATGAAGTTGAAGCTAAACAAGAT	2
640	GTTGCAATGATATTTGCATTAACTGCACTT	1
641	GTTGGAATGCTTAAGCAGGGGTGGAGTGAA	112
642	GTTGTAATTGTAGGAAAGGAGTTAATATGC	2
643	GTTGTAGTTTTACATAAGGACGATAGCGTT	5
644	GTTTAATGTTGTGTAAAGCGATAATGGTAT	1
645	GTTTCAACTTGACTTGCACCACATAAATAG	13
646	GTTTCAACTTGACTTGCACCGCATAAATAA	1

647	GTTTCAACTTGGCTTGCACCACATAAATAG	2
648	GTTTCCTTCCTATTTACTCTATACTCTAAA	1
649	GTTTCCTTCCTATTTGCTCTATACTCTAAA	165
650	GTTTCTTTCTTATTTACTCTATACTCTAAT	2
651	GTTTCTTTCTTATTTACTCTATGCTCTAAT	1
652	TAAAAAACAACTCACCGTTATAAACCTCCT	1
653	TAAAAAACAGCTAAGATAAAACTACCAGTT	1
654	TAAAAAACCGCTAAGACAAAACTACCGGTT	3
655	TAAAAAAGAGTTTTCCCTTGTAATGTTGCA	55
656	TAAAAAAGAGTTTTCCCTTGTAATGTTGTG	2
657	TAAAAGAAAGATTATCTAAATGGAGACAAG	2
658	TAAAAGAAAGATTATCTAAATGGCGTGAAG	1
659	TAAAAGATTTTGATTTTAGGATTTGGGATA	2
660	TAAAATTAGATAAAAATAATAATATAATCT	0
661	TAAAATTGCTAGTATTGATAGCGAAAGGGC	1
662	TAAACCCTTCTCCACCCTTTTGGAAGGGTG	27
663	TAAACTAGCTAAAGATAATTTAGAAATAG	0
664	TAAACTGTTAAAATCGTTCATATTTTTTG	2
665	TAAAGGCACAGCAGTAGGCCTATAAAATTT	1
666	TAAATCAAATCACAATCTCAAAGTCTCATA	1
667	TAAATCTACCATAAGGATTTGTGCAACTTG	1
668	TAAATGTGATGATACTAGAAAAGAAAATGA	1
669	TAACCTTGAAACACTCAAAAACTCAACAGA	1
670	TAACGGCTTACCCGAGGCTCAGCGAAGTTT	2
671	TAACTCCTTTAAATTAAAAATGGTATTATA	41
672	TAAGAAAAAAACATTAACCTTATTTGTATC	1
673	TAAGAGAATGCAAAATGTTTAGAAGCGATT	2
674	TAAGAGAATGCAAAATGTTTAGAAGTGATT	3
675	TAAGAGAATGTAAAATGTTTAGAAGCGATT	4
676	TAAGTCTTTTTAGTTCTTTCGAAAATGAAA	1
677	TAAGTGATATGTTATTAAGATTTAAAAGTGA	57
678	TAATAAAGCATTTGATTTGAAAGATGAGTA	13
679	TAATCATAATACACTCTTGTTTCAAAACTT	1
680	TAATCCTAATAATTTAACAGCGGGCATTCTA	1
681	TAATCCTAGTGAAATTGCAAAATTAACAAG	0
682	TAATCGGTTGAACACTCATTAAAGTTAAAA	1
683	TAATCGTAGAGCTAAAACTATAGCAAGAGA	0
684	TAATCTCTTTCACAACCGTGTTCTATAGT	1
685	TAATGAACCTTTAGAAGTTAGCGGAGTTAC	0
686	TAATGAGTAAAAAAGATAGTCAAGAATGAT	1
687	TAATGAGTAAAAAAGATAGTCAAGAATGGT	26
688	TAATGAGTAAAAAAGATAGTCAAGAGTGGT	0
689	TAATGGTGTTGTTAATTGGAATGCAAAAAGG	59
690	TAATGGTGTTGTTAATTGGAATGCAAAAGG	90
691	TAATTAAAACTTACATAACTAAAATCAGTC	2
692	TAATTAAATCAATTCCAAGTGAAATTATAG	73
693	TAATTAAATCAATTCCAAGTGAAATTGTAG	1
694	TAATTATTTAAATCATCTTTCCTATTTCCA	0
695	TAATTTACCTATTGAATTTCAATACAATGT	1
696	TAATTTTATATCTTCTAAATTAGATAAGT	8
697	TAATTTTTTGACAATAAGCGACCAAGATGC	12
698	TAATTTTTTGACAATAAGCGATCAAGATGC	0
699	TAATTTTTTGACAATAAGCGGGCAAGATGG	1
700	TACAGAATTTTTTATGAGTCAGCCTGATTT	1
701	TACATTTACTTAAGTCTTTAAACTCAGGGT	11
702	TACCTATAAAAGAGGTTATACCTAAAACTG	1
703	TACGCAGCAACACCAGATGGAAGAACTGAT	171
704	TACGTTATTTTGCATGTGGTGAATATGGTG	2
705	TACTATAATAGAGCAATTACCTATAATGGA	1
706	TACTATTTCTCTTTAGAAAACTAATATTTG	2
707	TACTCCTTTTAAATTAAAAATGGTATTATA	6
708	TAGCCGATTGGAGAAGTGAAAGAAGTTATT	1
709	TAGCTCTTATTGTAGGCTTAAGAATATTGT	1
710	TAGGAAATGTAAATGCTGGAAATTCATTAG	2
711	TAGTAAAATAACTTTGATTAAAATCAATAA	1

712	TAGTAGCTAAGAATAAAATAAGAAACACTG	91
713	TAGTATTTTGATATTCTGCTTCTTGGTCTT	6
714	TAGTTCTCTTTTAGCAAGTTCAGTTAAGCT	1
715	TAGTTTATAAAAATAAGCAAGATGTAGAAT	104
716	TATAAAAATATGAAAATAGTATCTTACGCA	12
717	TATAAAAATATGAAAATAGTATCTTATGCA	3
718	TATAAGGACTTCCATAATTTACACTTAACC	1
719	TATAATAAAACCGCCTGTAATGCTGTTAAA	1
720	TATAATTCTAGTTTCTATGATAGTCAATTG	1
721	TATACAAATTAAAAATTCTTTAGAAAAAGA	3
722	TATACTCTAAAGGCTCTCCCTCCCACACGT	22
723	TATAGCACTAAATTTTATGTAAATAATATA	1
724	TATAGCTTAAAAGCTATCGCCGTAGTTTTA	3
725	TATATAACTCATTTTTACTTGTGCAACAAT	4
726	TATATAACTCATTTTTACTTGTGTAACAAT	2
727	TATATAGTAATATTTCCACTAGCAAAAGCT	3
728	TATATTACAAGGGGTTATTTTAAATGCGTA	44
729	TATATTACAAGGGGTTATTTTAAATGTGTA	5
730	TATATTACAATGGGTTATTTTAAATGCGTA	1
731	TATATTACTACCAAAACTAACAATATCATC	1
732	TATCAATAAATGATAAATATATGTTGTAAG	2
733	TATCGCCCTTTTGATGAGTTAGTTAAAATC	1
734	TATCGCCCTTTTGATGAGTTGGTGAAAATT	1
735	TATCGCCCTTTTGATGAGTTGGTTAAAATT	46
736	TATCGTCCTTTTGATGAGTTAGTTAAAATT	15
737	TATCGTTCTTTTGAAGATTTAGCTAAAATA	5
738	TATCTTTGATAAACTTATTTAAGTTCTCAT	4
739	TATGAAAAAGAATTAATACGCTTTCGGATC	1
740	TATGAGCTATCAAGTTTTTGTATTTAGCAG	1
741	TATGAGTTATCAAGTTTTTGTATTTAGCAG	152
742	TATGGCAGTTTTTAAAAGAGCTTGGCGCTT	3
743	TATGGCAGTTTTTAAAAGAGCTTGGCGGTT	513
744	TATGGCAGTTTTTAAAAGAGCTTGGCGTTT	2
745	TATGGCAGTTTTTAAAAGGGCTTGGCGTTT	2
746	TATGGCAGTTTTTAAGAGAGCTTGGCGTTT	0
747	TATTATGATGAGAACTTAAATAAGTTTATC	8
748	TATTCCTCTAATCTCATCTAAATTCCAATC	1
749	TATTCTACCATTAGCATTAGAACTTTTTAA	3
750	TATTCTGATGTTTTTTAACTACAAATATCA	4
751	TATTTCACAACTTATATCTATAGACTTAAA	1
752	TATTTCTCTAATATCATCTAAATTCCAATC	1
753	TATTTGAACGCTTAAAATCAAGAAAATATT	29
754	TATTTTAACGCTTAAAATCAAGAAAATATT	2
755	TATTTTTACATTTTTAATTTGGAAATATCA	0
756	TCAAAATATAAAAAAGATTATGATAGAGCA	3
757	TCAAATGATTAGCAACTTCATATTCTTGA	2
758	TCAAGCGATAGTATTATAAATAATGCAATAA	1
759	TCAATTTAAAGAGGAAATTATTTGGAATA	72
760	TCACCAGTATCAAATTCAAAGTTTGGGGTTA	3
761	TCACCCCATTTTATAACGCAATCGGTTTT	1
762	TCACGAAGGTACAACTCAGGTTGCATTAAG	2
763	TCAGACTGAGTATCCTTTTGATTTAACAAT	1
764	TCAGCTTTTGCAAGCATTACGCTTTGGTTT	1
765	TCATAAGAAAAGTAAGTCGCTACTATACTA	1
766	TCATACTACTGCTATTGCAGGAATTTTTGG	1
767	TCATCATCAATATCCTTTAAGAAATCATA	2
768	TCATCATCACTTAAAACCTTAAATTTACC	1
769	TCATCATCATTTAAAACCTTAAATTTACC	1
770	TCATGAAACCTTCTGGTATCATAAGCCTAC	1
771	TCATTTTTGTAATCATGCAGAAGATTAATG	8
772	TCCAAATTCTAAACTTTGTCTATCAAGTTT	3
773	TCCATTCTCATAAAATATTTAGCCATTATT	75
774	TCCATTCTCATAAAATATTTGGCCATTATT	1
775	TCCATTCTCATGAAATATTTAGCCATTATT	180
776	TCCATTCTTATAAAATATTTAGCCATTATT	29

777	TCCCAACCCATAAAATAATAATAAATATCT	2
778	TCCCGTAAATAGAGCTCAGGTTGCATCAAA	1
779	TCCGTGTCGCAAATGTTTAATTTGCAGAAT	1
780	TCCTTGTTTTTTAATTTATTTTTATTATAT	1
781	TCCTTGTTTTTTAATTTATTTTTGTTATAT	79
782	TCCTTTAGTAGAATAAGGCGACGCTTGATA	3
783	TCGTCTGGTGAAAATTCTATTACTTCGGGA	1
784	TCTAAAATTGTTTCATTTTCATTAGTAGCT	6
785	TCTGTTGAAAATCATATTTATTTAGGAGAG	1
786	TCTTATAAACAAAGCGGCAATGAAGGCTTA	1
787	TCTTCATAGTTAAAAAACATCAGAATGAAA	5
788	TCTTGAATTAACATCAAATCATTTCCATTT	1
789	TCTTGCATTTGTATATTATAATGTGTAGCA	7
790	TCTTTAGAAAGTGAGTATGGTTCTCTTATA	3
791	TCTTTGCTTACAAACTCTTATGATTATTTA	1
792	TCTTTTAAGCTTATACTTATAAAAAAAGTA	3
793	TCTTTTTACTAATTTTGTAGAATATATTA	3
794	TCTTTTTCACAATATGGTAAAGTAGTTGTA	0
795	TCTTTTTTCTTTGAAAATGGAGGATTGCTA	11
796	TGAAAAATTAAAACAATTTGATGGAGTTTA	1
797	TGAAAAATTAACACAATTTGATGGAGTGTA	2
798	TGAAACAAGAATTATGAAAAGAGGAAGAG	3
799	TGAAACATAATTTTTCATAAATTGACAAAG	170
800	TGAAATTCATAATACAGAGTATGAAGCCAA	1
801	TGAAATTCATAATATAGAATATGAGGCAAA	22
802	TGAAATTCATAATATAGAGTATGAAGCCAA	1
803	TGAAATTGGAAGTTACGAGTGGAGTGAAA	2
804	TGAACCAAAAATGAGAATAATTAATGATAGC	1
805	TGAACTTTTAACTAAAATTAGCAAACTAGA	1
806	TGAACTTTTAACTAAAATTAGTAAACTAGA	2
807	TGAATTATTAACAAAAACAACTAAACTAGA	1
808	TGAGATAGGAAATGTAAATGCTGGAAACTC	1
809	TGAGATAGGAAATGTAAATGCTGGAAATTC	2
810	TGAGATAGGAAATGTTAATGCTGGAAATTC	1
811	TGAGCTTAACGCAGGACTAGAAACAGTTAA	4
812	TGAGGACTTTTTAATAAGTGATAATCAATA	5
813	TGATAATAATTCTCCTAAAATTAAGAATGA	3
814	TGATAATAATTGCTTAAGGGTATCTGCTAT	1
815	TGATAGTGTAAATATTGTCTGCAAGAATCA	1
816	TGATGCAAGTAGCGATATGTGGAATCCTGC	1
817	TGATGTTCTTGAGTATACTAGATGGTGCG	1
818	TGATTGAAGTGTAGTAAAGGCTGTAGCACT	50
819	TGATTGAAGTGTGGTAAAGGCTGTAGCACT	1
820	TGATTTCCATCTATCCCATTTAAATGTATT	44
821	TGATTTTTACGAGTTAAAAAATATTATAGA	4
822	TGATTTTTATGAGTTAAAAAATATTATAGA	1
823	TGCACTGAAATAAATGAGTTTATAGCAAAT	118
824	TGCACTGAAATAAATGAGTTTATAGTAAAT	1
825	TGCAGATGAAACGGCGCCACCAATTAAGTT	1
826	TGCAGGTAATTATAATATTGCATTTAACGA	1
827	TGCATTATTTATAATACTATCGCTTGCTAA	3
828	TGCATTGTTTATAATACTATCACTTGCTAA	4
829	TGCCTTTAAAAGCTCGCAAGATGCTATGAA	1
830	TGCGTTTACTCCGCATTTGTAAGCTATCA	2
831	TGCTAAATTTTCATTAACTCTATTAATATA	0
832	TGCTAGTGAAAATCAATTAAAATTAAGAGA	1
833	TGCTTAACTCTATAAATATGGTCTCTTATT	1
834	TGGAGCTACTTCTGTGGATATAAGTATAGA	3
835	TGGAGTTGAAAGAGTCAATAATGCTAATAT	1
836	TGGAGTTGAAAGAGTTAATAATACAAATAT	26
837	TGGAGTTGAAAGAGTTAATAATACTAATAT	1
838	TGGAGTTGAAAGAGTTAATAATGCTAATAT	1
839	TGGATAAGTTTTTTGTAATGTTTCATATAC	1
840	TGGGAGCTGCTATGGGACAAGCTGTTGCAT	2
841	TGGGGAGAAAATGAGCAATGGATAAGAGAG	1

842	TGGGGTGAAAACTCAATTAGTAAATGATGT	3
843	TGGTTTAAAAAGCGGAGATGTAGGATATAC	0
844	TGGTTTAAAAAGTGGTAATGTAGGATATAC	0
845	TGTAGCAATTTGCATAGTTGCAAAACTATT	4
846	TGTAGCTGGTAAGGTTATAAGAGGTTTATT	6
847	TGTCTTGAATTAACATCAAATCATTTCCAT	1
848	TGTGCGACTTTTAATCTTTTTAATTGTTTG	1
849	TGTGGTGTGCTTGGGTCTGTATTGATATCT	5
850	TGTTAATGATAGACAAACAGCAGACGGAGA	1
851	TGTTAATGATAGACAAACTGCAGACGGAGA	10
852	TGTTCTACTTTCAATCTCTTTGATTGTTTG	1
853	TGTTTAACGCTATAAATGTGGTCTCTTATA	1
854	TTAAAAGTTTAGCTGAACTTGTTAAAAGAG	3
855	TTAAAAGTTTAGCTGAACTTGTTACAAGAG	0
856	TTAAACAAAACGGTTTGTATTTAAATCAAG	1
857	TTAAACAAAATGGTTTATATTTAAATCAAG	1
858	TTAAATGAAAAACAAGGAACTGCTAGTTTA	3
859	TTAAATTGAGTGTAGATTTAGTAAATAATC	2
860	TTAAATTTCAAAGATGAGAGTATAGCTAA	221
861	TTAACAAAAGTTAGAGCCTATGGAGATATT	1
862	TTAACCAAAATCTTAACTTTACCATCCGAA	2
863	TTAACTCTTATGATATTGAAACAAAACTGA	2
864	TTAAGCGATATTTGCAAGATTTTAGAAATA	1
865	TTAAGTAGCACAGGTGCTTCAAACATAATT	1
866	TTAAGTGATATTTGCAAGATTTTAGAAATA	24
867	TTAATACCAAATAAAAAAGTAAGCTTCGAT	1
868	TTAATTCTTATGATATTGAAACAAAACTTA	6
869	TTACAGACTTAGGAGTTATTAATGTTTAGA	1
870	TTACATAATATTGATGTAAAGCATCTAAAG	1
871	TTACGATTTAATGTATAAGCAAGACTTATC	1
872	TTAGAATTTAGTAAAATTGTAAAAAATAGGT	1
873	TTAGACTTAGCAGTTAATGATTTTAAATTA	1
874	TTAGCAACTTATAATAACTCTAATGTTATT	216
875	TTAGTTTCTTGAGGCGTAGTAGCATTTTTA	1
876	TTATAACCGAAAAAAGAGAGCATTACAATA	0
877	TTATAACCGAAAAAAGAGAGCATTACACTA	8
878	TTATAACCGAAAATAGAGAGCATTACACTA	2
879	TTATAACCGAAAATAGAGAGCATTACGCTA	10
880	TTATAACCGAAAATAGGGAGCATTACACTA	1
881	TTATAGCTTTTGTAGAACCAACGCCAATTA	6
882	TTATAGTTATAGTAATGAAATGCGTTTGTTA	1
883	TTATGTTTTAAATAATAGCAGTTCACAAAA	1
884	TTATTAAATGTTAGATTATAATTCTTGGCA	3
885	TTATTATCTTTATTCTTATTATATATAGT	4
886	TTATTATGCTTCTAACATTAGTTTCTTGA	1
887	TTATTGGTGTGTAAGAATAGAAGATAATAA	1
888	TTATTTGAACGCTTAAAATCAAGAAAATATT	0
889	TTATTTTTGTCGCTAATTGCACCTAAAGAC	127
890	TTCAAAAAGCGTAATTGACATGAACGCCCA	0
891	TTCAAAGCATTCTTCATTATCATTCATTAA	1
892	TTCAACGAGTTATTAAATGAGTGGGATAA	1
893	TTCAATTTAAAGAGGAAATTATTTGGAATA	19
894	TTCCTAGTATCATCATCGCTTAATAACATA	1
895	TTCCTAGTCTCATCATCGCTTAATAACATA	1
896	TTCGAGTATTGTAAACAACAGAATTCAGT	1
897	TTCGCAGAATGCGTAGACTTAGACGCTATC	1
898	TTCTAAAGCTTCTATAGATGTTTTATTGTC	1
899	TTCTAAAGCTTCTATAGCTGTTTTATTATC	1
900	TTCTACATCATAAAGATTTGTAGCATCTAT	2
901	TTCTACGATAGTCAATTAGAAAATTCAGTT	1
902	TTCTATGATAGTCAATTGGAAAATTCAGTT	3
903	TTCTGAGTAACTCTGACCTGAAGTACTAT	2
904	TTCTGGTTGTTTTAACTCTACTGGAAACGGT	1
905	TTCTTCATCATCTAAAACCTTAAATTTACC	1
906	TTCTTGTTTCATAATTAAGCCTGAGATTAT	1

907	TTGAAAACGATAATCTTGTGGAACTTTCAC	6
908	TTGAAACTCATAATATAGAAACTGCATTAA	2
909	TTGAGCGTTTAAACTTGATAAGATACCCGC	7
910	TTGAGCTTAGCGAGTTTGTAAGCCTTAGTG	4
911	TTGATGGCAGGGAATTTATATGGATTAATT	1
912	TTGATGGTAAGGAATTTATATGGATTAATT	0
913	TTGATGGTAGGGAATTTATATGGATTAATT	8
914	TTGATTGAAGTGTAGTAAAGGCTGTAGCACT	3
915	TTGATTTTAGGATTTGGGATAATAAATATA	5
916	TTGCATTATTTAAATAAATCCCTTCTAAAA	2
917	TTGGATATTCAAATGAGCGAAATAGTTAAA	1
918	TTGGTTATTATAAGCCTTAACAAATTCAATA	1
919	TTGTAAGTTCTAAATAAAAACTAGCTTGTA	2
920	TTGTCTTATAGTGAATGTGCATTTTTAGGT	1
921	TTTAAAAGAGAATTATCAGAAAGTCTTAGT	1
922	TTTAAAGAGGAAATTATTTGGAATAAAAAG	1
923	TTTAAATTTCAAAGATGAGAATATAGCTAA	21
924	TTTAACTCTTCTACAATGGGAGCAAATTCA	4
925	TTTAATGTTGTGTAAAGTGATAATGGTAT	2
926	TTTAGCCTTCTCACATATTTTATTTAATTC	11
927	TTTAGGGGTAAGTGGGAGTTTAATGAGTTG	1
928	TTTAGGTGGCGCAATTTCTAATGACTTAAT	1
929	TTTATCTGCATCCATAATGGCAATGAGTGA	2
930	TTTATCTTAACAATGACGGCCAAAGGAGCT	3
931	TTTATGCTACGCTAAGCCAAGCCACTAAGG	13
932	TTTATTATTTTATAACAACTAATGAAAAG	1
933	TTTCAAGAGACTTGCTAAAATCTGGCTGGA	1
934	TTTCACAACCTATAAAATTTCTATTTTTAA	5
935	TTTCAGCACTATCATACTTACTTACAACTA	1
936	TTTCAGTGCTAAACATTATAATCCTTTAAA	1
937	TTTCATAGGCACATGATTATGAAATTACAC	1
938	TTTCCAAAGTTTCATTAGTTGAATTTAACT	341
939	TTTCGCAACCTATAAAATTTCTATTTTTAA	2
940	TTTCTTATGAAGTTTTTAAAGGAATTATAC	2
941	TTTGAAGGCATTATAATACTTAAGTCACAA	1
942	TTTGAGCGAACTCTTTTTGAGCATTATAGT	1
943	TTTGCAACAAGTACAAAAGAAACTATATTA	1
944	TTTGCCACTTCTTTACCTATGGTTCCTAAA	2
945	TTTGCGACAAGCACAAAAGAAACTATATTA	1
946	TTTGCGACTTTCAATCTCTTTAATTGTTTG	1
947	TTTGGCGGAGGACAAACAAAAGAAGAAGTA	2
948	TTTGGGTTTATTTGGTAGAACCGAAGATGA	2
949	TTTGTCAAGTTTCAAGCGAAGACGCAAAGC	1
950	TTTGTTGAGTGGGTTATGAGACAAAAGAA	6
951	TTTGTTTTTCTTTATTCTAAAGATTTTTTA	0
952	TTTTAATGATTTAGAAAAGAAACAATACTC	2
953	TTTTAATTCTACAAATTAAGCATTTTCCGC	1
954	TTTTAATTTCTTAATTTTATCAAAAAGTTT	1
955	TTTTCAAGTGGTGGTAATTCTAGCAACTCA	1
956	TTTTCAATAGTATCACTATTTTTAATACTT	1
957	TTTTCTAACTGATGTTTTAGGAGTGCCTTA	13
958	TTTTGATGAGGAATATAGAAAAGAACTTGG	1
959	TTTTGCTGATGTAGTTGATAATGATCAAGT	1
960	TTTTGGTGTGAAAGAATTCACTATGATAGA	1
961	TTTTGTTGAGTGGGTTATGAGACAAAAGAA	3
962	TTTTGTTGAGTGGGTTATGAGCCAAAAGAA	26
963	TTTTGTTGATTGGGTTATGAGCCAAAAGAA	2
964	TTTTTAATAGTATCACTATTTTTAATACTT	0
965	TTTTTCTTTATCATATTCCATAGCTTTGGT	2
966	TTTTTCTTTCTATAAATAGAATTTGAAGTT	1
967	AATAAAGTTTATGAAGGTGATATATTGTAT	1
968	GCACCTGTATCGCCTATAATAAAAACATTG	1
969	AAAAGGTGAGAATTTTAAAGAATGTTATCA	1
970	AATTTTTCATTTTCTAAAGCTAGTCTTCCT	1
971	TTATGATATTTAACTATATCGCTCATTTGC	1

972	AGAATCATTCAAAATTCTTTTATATTAGT	1
973	CTTTTCTACTTGGATAAACAAAAAGTTTA	2
974	TCTTCCAAAACTATTAACACTGTTGCTAAA	1
975	TTAATAAAATCATCATCCACAGTCTTTTTT	2
976	CTGTTACTGATATATTACTTGCCATTTTAA	1
977	TGCGATAATCGGGGTGCGGGGGCGTGAAGT	1
978	CAAGCTCATGCAAACGAAGATGAAAGCGAA	1
979	ATTGCAGGCTGACTTAACCTTTTTTGCTTA	1
980	TGTGTATTCTGTAATGTAATGTGCCTCAT	1
981	CTCGTATTCGTGTCGTGGCAATCTTTTTC	1
982	TTTGATATATTGATTTGCAACTTCTGCTTT	1
983	AATGAAAATACTCTATCATCAACCCAGGAA	1
984	AGAAAATCTACGCTTGAAAGTTTTTCCCT	1
985	ACCAATTTTTCTAACTTCTAGAATACTTTC	2
986	TTTAGAAGAAGAAATATCTTATCTTCAAAG	1
987	GATAATCCCCAAACAGCACAAATAGCTATA	1
988	CAAATGAACCAAATAATCTTAATGGCATTA	1
989	ATAATGCTAAAGTAGGCGGAGCCCCTAAAA	1
990	AGCACAAATAGAAGCCTCTTTAAATTTAAT	1
991	TACATTAAGTCGTAAAATTCTTTGCACGC	1
992	TGATTTATCAAGAACCGCAAGATCCCAAG	0
993	TATAGAAGGCTATCAAAAATCTATTTAGA	0
994	TATTTTGTGAGTGCTATTGAATTTGTTAAG	0
995	TTTAGTGCGTGAGTTATTTCTAATAGTATA	0
996	GATAACACTTCAAGTGATCTAGACGATTTT	0
997	AGCATCTGCTATACATATAGATACTGCTGTA	0
998	TTGCGAATGTGTTTTTGATAAAACATTAGT	0
999	AAATAAAGATATTAAAAATAAATTAGAAAA	0
1000	TCCTCCGCCATTTATTTGGTTTCCATTATG	0
1001	TTTGAAGACTGCCCTGAAGATGAAGCTTTT	0
1002	AGAATTGTATTTTTCTAACAGTAGCACAAG	0
1003	ATGAGGGTAAGCAATTTAATCCATGAAAAA	0
1004	AGAGGGTTTAAACAATACTAGAAGATTTAA	0
1005	TTTATAGGCAAATTTAAAGTTTTTAGCGAT	0
1006	TATTGCCACCCCTATGTTTTAATTCTTGTA	0
1007	TGTATATAGAAAGTGTTAATCGCGTCTTCT	1
1008	TCACCTGTCGCGTTAAATCCACTTGGGCTT	1
1009	TATGGGTTTTTTTCAATCTTCGAAAGTATT	1
1010	AACTCTAGCCTTAGCTGATTATACACAATA	1
1011	GTGTTTGATAAGCGATTTATTTAAAACTAT	1
1012	ATAATATGCCTTATCAATACTACATTGACA	2
1013	GATGTATTAAATATAGTATCATTGTTGTAT	1
1014	TTTAATATATGTTTTAACTTGTCATCAATA	1
1015	TTGAGCTGGTTAAGTGGATATACAGCAAGA	1
1016	GCTTTTGCAGACAACATACAAATTCAAGAT	1
1017	TTTAAACGACTTAGAAGCGATTTTTCAGGA	1
1018	ATTTTTCAATCTTATGCACAAGTTGATGTA	1
1019	ATAAAAATACTTTGCCCTTTTGATGACGAG	1
1020	TGCTCTAATGCAATGTTTGTAAGGCTTGAG	1
1021	AGTGGGTTTAGATTGGGGTAAATTGGCAAA	1
1022	ATAGTGGCTTAATTCTAGCTTGCTATCAAA	1
1023	TAATGGTGTTGTTAATTGGAATGCAAAAGGG	0
1024	ATTTTGCTTTATTTAAATTGTTTTCGCT	0
1025	AGCGATATGTTATTAAGATTTAAAAGTG	0
1026	TATGGGCAGTTTTTAAAAGAGCCTTGGGCGGTTGTTTTAGTC	0
1027	AGGGGTGATATTGTTATGAGTATTATGCAGG	0
1028	CAGCATTAAACAAAAATCCACACATTAG	0
1029	GAATCCACACAACCACCACATGCATAAAGG	0
1030	TTAATTTTTTGACAATAAGCGATCAAGATGC	0
1031	TATGGCAGTTTTTAAAAGAGCTTGGCGGTTG	0
1032	GGAATGCTTAAGCAGGGGTGGAGTGAA	0
1033	TTTCCAAAGTTTCATTAGTTGAATTTAAC	0
1034	CTTTTACTTTATTGAAAGTTTCTAAATCTA	0
1035	TAAGAGAATGTAAAATGTTTAGAAGTGATT	0
1036	ATAGCGATGGTATTTGGCTTGAAAATATAG	0

1037	ATAGCGATGGTAATTGGCTTGAAAATATAGG	0
1038	ATTGCTAGTCAATTAGGCTTAGATGACGC	0
1039	GGGGTGATATTGTTATGAGTATTATGCAA	0
1040	AATGTAAAATGTTTAGAAGTGATTTACAAC	4
1041	AAATCGTCGAGCCAAAACAATAGCACGAGA	8
1042	ATTTCTAATTTCATTTATAACCTTTC	0
1043	GGAATGCTTAAGCAGGGGTGGAGTGA	0
1044	TGATTGAAGTGTAGTAAAGGCTGTAGCAC	0
1045	TATACTCTAAAGGCTCTCCCTCCCACACG	0
1046	AAGAATAGCCTATCTTTTTGATAATTACGA	0
1047	ATGATGAAAGTTCTAATTATGTTAAAGTTT	0
1048	ATGGCAGTTTTTAAAAGAGCTTGGCGGTT	0
1049	AATTTACAGCACAAAATAAAGATTTTAAT	0
1050	ATCCTAGCGAGGAAAGCAAAACTGATGAT	0
1051	ACTTTGGAAAAAACTAAGAATGATTTAAA	0
1052	AATAATGATATCGAAGTTATTAATCTAATTG	0
1053	TATGGCAGTTTTTAAAAGAGCTTGGCGGT	0
1054	AATTGCTAGTCAATTAGGCTTAGATGACGCG	0
1055	CCATTAGTTTTTAAAGGGTGTAAGTATAACG	0
1056	ATTCAATGTTGTGTAAAGCGATAATGGTATG	0
1057	ATGGTGTTGTTAATTGGAATGCAAAAGG	0
1058	AGCCTTTAGAGTATAACGATAATCAAGTC	0
1059	GGGTGATATTGTTATGAGTATTATGCGA	0
1060	CACGCTCACGCGGTATTTTTTACTCTAGAC	0
1061	CCATTAGTTTTTAAAGGGTGTAAGTATAAC	21
1062	ATTCAATGTTGTGTAAAGCGATAATGGTAT	11
1063	TGCACTGAGATAAATGAGTTTATAGCAAAT	1
1064	GCAATTTTAATACACTGGGGAAACACTTAT	0
1065	TGAAATTCATAATATAGAATATGAGGCAAAG	22

a. Spacer alleles 1-37 were described in Kovanen et al, 2014, but incorporated a G on the 3' end derived from the next repeat in the array. This last nucleotide has been removed here.

b. Number of times specific spacer is detected in the 4,232 *C. jejuni* and *C. coli* genome sequences investigated in Figure 5C, Table 3 and Supplementary Tables S7 and S8, based on BLAST+ analysis of the spacer sequence combination with a 5'-TGGTAAAAT and 3'-GTTTT linker to aid specificity.

Table S6. Predicted phage, plasmid and genomic insertion element targets of *C. jejuni* and *C. coli* CRISPR spacer sequences

The sequence of the 30 nt protospacer is included, with 8 nt of the 5' and 3' sequences for determination of potential PAM motifs.

Red underlined residues show differences between crRNA and protospacer. as predicted by CRISPRtarget (http://bioanalysis.otago.ac.nz/CRISPRTarget/crispr_analysis.html).

All 1065 *C. jejuni/C. coli* CRISPR spacer alleles shown in Table S5 were tested against the Genbank Phage, Refseq Viral and Refseq Plasmid databases using default settings of CRISPRtarget

Orange lines denote prophages/insertion elements (i.e. CJIE1-CJIE4 of *C. jejuni* RM1221), yellow lines denote *Campylobacter* phages, white rows denote plasmids.

target	upstream	Protospacer	downstream	Mismatch	CRISPR spacer	Spacer allele	Protospacer_description	Acc numbr
phage	TCCTTTTG	ATGATGAAAGTTCTAATTATGTTAAAGTTT	TTAAACAC	0	ATGATGAAAGTTCTAATTATGTTAAAGTTT	1047	Campylobacter phage CPX	JN132397
phage	TCCTTTTG	ATGATGAAAGTTCTAATTATGTTAAAGTTT	TTAAACAC	0	ATGATGAAAGTTCTAATTATGTTAAAGTTT	1047	Campylobacter phage CP81	FR823450
phage	TCCTTTTG	ATGATGAAAGTTCTAATTATGTTAAAGTTT	TTAAACAC	0	ATGATGAAAGTTCTAATTATGTTAAAGTTT	1047	Campylobacter phage CP8	KF148616
plasmid	AAATCTAA	CTTGATTAAGTTTCATATTGTTTAAAATCT	TTAAATAT	0	CTTGATTAAGTTTCATATTGTTTAAAATCT	521	Campylobacter coli plasmid p3384	NC_007142
plasmid	TCTTTGTA	GCTACAAAGAGTAACCTCGCCCTATGAGAA	AAGGATAC	0	GCTACAAAGAGTAACCTCGCCCTATGAGAA	601	Campylobacter coli RM2228 plasmid pCC2228-1	NC_008049
plasmid	TCTTATTT	GTCTTTGTCTTTCTAATTCCCATTCATTCA	TACAACAC	0	GTCTTTGTCTTTCTAATTCCCATTCATTCA	632	Campylobacter jejuni 81-176 plasmid pTet	NC_007141
plasmid	GTTCTAAA	TAAACCCTTCTCCACCCTTTTGGAAGGGTG	TGAGATAC	0	TAAACCCTTCTCCACCCTTTTGGAAGGGTG	662	Campylobacter coli RM2228 plasmid pCC2228-1	NC_008049
plasmid	TTATAAGC	TAAATCAAATCACAATCTCAAAGTCTCATA	ATAAATAC	0	TAAATCAAATCACAATCTCAAAGTCTCATA	666	Campylobacter jejuni plasmid pCG8245, partial sequence	NG_036035
plasmid	GTCTTACT	TTGGATATTCAAATGAGCGAAATAGTTAAA	TATCACAA	0	TTGGATATTCAAATGAGCGAAATAGTTAAA	917	Campylobacter coli RM2228 plasmid pCC2228-2	NC_008050
plasmid	CAATTAGA	TTTAAAAGAGAATTATCAGAAAGTCTTAGT	AGAAATAC	0	TTTAAAAGAGAATTATCAGAAAGTCTTAGT	921	Campylobacter jejuni plasmid pCJ419	NC_004997
plasmid	GATCATTT	TTAATAAAATCATCATCCACAGTCTTTTTT	TCAACCAC	0	TTAATAAAATCATCATCCACAGTCTTTTTT	975	Campylobacter coli plasmid pCC31	NC_006134
plasmid	CAATGATT	TGCGATAATCGGGGTGCGGGGGCGTGAAGT	TAGCCTAG	0	TGCGATAATCGGGGTGCGGGGGCGTGAAGT	977	Campylobacter jejuni plasmid pCJ01	NC_008438
plasmid	TCAATGAA	AATGAAAATACTCTATCATCAACCCAGGAA	AAACCTAA	0	AATGAAAATACTCTATCATCAACCCAGGAA	983	Campylobacter coli 15-537360 plasmid pCC42yr	NC_022656
plasmid	GGAATTTC	TTTAGAAGAAGAAATATCTTATCTTCAAAG	CCGAACAC	0	TTTAGAAGAAGAAATATCTTATCTTCAAAG	986	Campylobacter jejuni IA3902 plasmid pVir	NC_017284
plasmid	AAACATTA	GATAATCCCCAAACAGCACAAATAGCTATA	GTAAATAC	0	GATAATCCCCAAACAGCACAAATAGCTATA	987	Campylobacter coli plasmid pCC31	NC_006134
plasmid	ATTTAACA	GCTTTTGCAGACAACATACAAATTCAAGAT	GTTCCAAA	0	GCTTTTGCAGACAACATACAAATTCAAGAT	1016	Campylobacter coli RM1875 plasmid pRM1875_35kb	NZ_CP007184
prophage	AAGTCGTG	CAATTTTAATACACTGGGAAACACTGAT	AAAGACAC	0	CAATTTTAATACACTGGGAAACACTGAT	420	Campylobacter phage CJIE4-4	KF751796
prophage	GATTTGAA	AATACTTTAAGAAGTATTACAAAAAAAGAT	GATACAAT	0	AATACTTTAAGAAGTATTACAAAAAAAGAT	152	Campylobacter phage CJIE4-5	KF751797
prophage	TAGTAAAC	AATGCAAAAAAATTTATAGTGAAATTACAA	AAAAACAC	0	AATGCAAAAAAATTTATAGTGAAATTACAA	161	Campylobacter phage CJIE4-2	KF751794
prophage	TGTATTAA	AATTGCACGACTTGATGGCACATCATCATT	ATAAACAT	0	AATTGCACGACTTGATGGCACATCATCATT	171	Campylobacter phage CJIE4-4	KF751796
prophage	AGTCGTGC	AATTTTAATACACTGGGGAAACACTGATAA	AGACACAC	0	AATTTTAATACACTGGGGAAACACTGATAA	181	Campylobacter phage CJIE4-4	KF751796
prophage	AAGATTTA	AGAATACCTGAAGGAAATTATAATTTAAAA	AGACATAG	0	AGAATACCTGAAGGAAATTATAATTTAAAA	249	Campylobacter phage CJIE4-5	KF751797
prophage	ATGAAGTT	CCTTCAAGTCGTGCAATTTTAATACACTGG	GGAAACAC	0	CCTTCAAGTCGTGCAATTTTAATACACTGG	468	Campylobacter phage CJIE4-1	KF751793
prophage	GCTGATGA	GTGTTGCTTATGGTCTAGTTCAGGAAGTAG	AGAGGTAA	0	GTGTTGCTTATGGTCTAGTTCAGGAAGTAG	635	Campylobacter phage CGC-2007 isolate Cj00-0949 cje0215 g	EF694684
prophage	TTAGGACT	TTCAAAAAGCGTAATTGACATGAACGCCCA	TTTAACAC	0	TTCAAAAAGCGTAATTGACATGAACGCCCA	890	Campylobacter phage CJIE4-4	KF751796
prophage	GATAAAGC	TTTTCTAACTGATGTTTTAGGAGTGCCTTA	TAAAACAC	0	TTTTCTAACTGATGTTTTAGGAGTGCCTTA	957	Campylobacter phage CJIE4-3	KF751795
phage	AAGTTAAA	AGAAAAGTTCCTAGCTTGGATGAATCCAGA	TGAAACAC	1	AGAAAAGTTCCTAGCTTGGATGAATCC**T**GA	239	Campylobacter phage CP30A	JX569801
phage	TTAAAGGT	TGTTTAACACTATAAATGTGGTCTCTTATA	ATTGATAC	1	TGTTTAAC**G**CTATAAATGTGGTCTCTTATA	853	Campylobacter phage CP21 complete sequence	HE815464
phage	TCCTTTTG	ATGATGAAAATTCTAATTATGTTAAAGTTT	TTAAACAC	1	ATGATGAAA**G**TTCTAATTATGTTAAAGTTT	1047	Campylobacter phage CP30A	NC_018861
phage	TCCTTTTG	ATGATGAGAGTTCTAATTATGTTAAAGTTT	TCAAACAC	1	ATGATGA**A**AGTTCTAATTATGTTAAAGTTT	1047	Campylobacter phage NCTC12673	NC_015464
plasmid	ATATTAAT	AAATGTCATAAGAAGCATAACAGTTATCCA	AATAACAC	1	AAATGTCAT**C**AGAAGCATAACAGTTATCCA	109	Campylobacter jejuni 81-176 plasmid pVir	NC_008770
plasmid	GTTCTAAA	TAAACCCTTCTCCACCCTTTTGGAAGGGTG	TGAGATAC	1	**C**AAACCCTTCTCCACCCTTTTGGAAGGGTG	401	Campylobacter coli RM2228 plasmid pCC2228-1	NC_008049
plasmid	TAAAATCA	TTATGATATTTAACAATATCGCTCATTTGC	ATATCCAA	1	TTATGATATTTAAC**T**ATATCGCTCATTTGC	971	Campylobacter lari plasmid pUPTC237 DNA	NC_007962
plasmid	TTCTCGTT	AGAATCATTCAAAATTCTTTTTATATTAAT	AACACCAA	1	AGAATCATTCAAAATTCTTTTTATATT**A**GT	972	Campylobacter jejuni 81-176 plasmid pTet	NC_006135
plasmid	TCGCCTTT	TTTGATATATTGATTAGCAACTTCTGCTTT	ACGCCCAA	1	TTTGATATATTGATT**T**GCAACTTCTGCTTT	982	Campylobacter coli RM1875 plasmid pRM1875_35kb	NZ_CP007184
plasmid	ATTTGACA	GCTTTTGCTGACAACATACAAATTCAAGAT	GTTCCTAA	1	GCTTTTGC**A**GACAACATACAAATTCAAGAT	1016	Campylobacter jejuni 81-176 plasmid pVir	NC_008770
prophage	AAGTCGTG	CAATTTTAATACACTGGGGAAACACTGAT	AAAGACAC	1	CAATTTTAATACACTGGGGAAACACTGA**C**	419	Campylobacter phage CJIE4-2	KF751794
prophage	GAATTTTA	TCATCATCATCTAAAACCTTAAATTTACC	AATAACGC	1	TCATCATCAT**T**TAAAACCTTAAATTTACC	769	Campylobacter phage CJIE4-2	KF751794
prophage	AGTCGTGC	AATTTTAATACACTGGGGAAACACTGATAA	AGACACAC	1	AATTTTAATACACTGGGGAAACACTGA**C**AA	180	Campylobacter phage CJIE4-4	KF751796
prophage	AAGATTTA	AGAATACCTGAAGGAAATTATAATTTAAAA	AGACATAG	1	AGAATACCTGAAGGAAATTATAATTTAA**G**A	250	Campylobacter phage CJIE4-4	KF751796
prophage	GAAGAAGA	CAAAGAAGGTTTAGAAAGTGGCAAAGATTT	AAGAATAC	1	CAAAGAAGGTTTAGAAAGTGG**T**AAAGATTT	403	Campylobacter phage CJIE4-5	KF751797
prophage	ATGAAGTT	CCTTCAAGTCGTGCAATTTTAATACACTGG	GGAAACAC	1	CC**A**TCAAGTCGTGCAATTTTAATACACTGG	453	Campylobacter phage CJIE4-1	KF751793
prophage	CAAGTCGT	GCAATTTTAATACACTGGGGAAACACTGAT	AAAGACAC	1	GCAATTTTAATACACTGGGGAAACACTGA**C**	580	Campylobacter phage CJIE4-4	KF751796
prophage	CAAGTCGT	GCAATTTTAATACACTGGGGAAACACTGAT	AAAGACAC	1	GCAATTTTAATACACTGGGGAAACACT**T**AT	1064	Campylobacter phage CJIE4-3	KF751795

phage	AAAAATTA	ACAGTAAAAACAGATACACTAGATCCTGTA	GAAAATAC	2	ACAGTGAAAACAGATACACTAGATCCTGTG	194	Campylobacter phage CP8	KF148616
phage	AAGTTAAA	AGAAAAGTTCCTAGCTTGGATTAATCCAGA	TGAAACAC	2	AGAAAAGTTCCTAGCTTGGATGAATCCTGA	239	Campylobacter phage NCTC12673	GU296433
phage	AAATTAAA	AGAAAAGTTCCTAGCTTGGATTAATCCAGA	TGAAACAC	2	AGAAAAGTTCCTAGCTTGGATGAATCCTGA	239	Campylobacter phage CPX	JN132397
phage	GAATTATT	ATAAGCATTTCTATTAAGTTGTGCTGCACT	TAAAACAC	2	ATAAGCACTTCTATTAAGTTGAGCTGCACT	317	Campylobacter phage CP30A	JX569801
phage	GAATTATT	ATAAGCATTTCTATTAAGTTGTGCTGCACT	TAAAACAC	2	ATAAGCACTTCTATTAAGTTGAGCTGCACT	317	Campylobacter phage CP8	KF148616
phage	TAAATTTC	ATAATACCAAATAAAAAAGTTAGCTTTGAT	GGAAATAC	2	ATAATACCAAATAAAAAAGTAAGTTTTGAT	319	Campylobacter phage CP8	KF148616
phage	CAGCACAA	CTTAATAGAAATGCTTATAATAATTCAAAT	GCTGATAC	2	CTTAATAGAAGTGCTTATAATAATTCAAGT	515	Campylobacter phage CP8	KF148616
phage	AACGAAAC	TACAAGATTTTGATTTTAGAATTTGGGATA	ATATTGAA	2	TAAAAGATTTTGATTTTAGGATTTGGGATA	659	Campylobacter phage vB_CcoM-IBB_35 clone Contig1	HM246720
phage	CCAAGCCA	TATTTTACCTATTGTATTTCAATACAATGT	AAGAAAAT	2	TAATTTACCTATTGAATTTCAATACAATGT	695	Campylobacter phage CP81	FR823450
phage	GAACAGGG	TATTATGATGAGAAGTTAAAGAAGTTTATC	AAAGATAC	2	TATTATGATGAGAACTTAAATAAGTTTATC	747	Campylobacter phage NCTC12673	GU296433
plasmid	CCTAAAAG	AATTTAGCTTTTGGCTAAAAGCTAAATTT	AGGCTTTT	2	AATTTAGCTTTTAGCTAAAAGCTAAATTC	177	Campylobacter jejuni plasmid pCJ01	NC_008438
plasmid	TCAAACCT	GATTTATTAGTAACACATCAAGTTGGGTT	ACAAATAA	2	GATTTATTAGTAACCCATCAAGTTGGCTT	569	Campylobacter jejuni 81-176 plasmid pVir	NC_008770
plasmid	TTCAAACC	TGATTTATTAGTAACACATCAAGTTGGGTT	ACAAATAA	2	TGATTTATTAGTAACCCATCAAGTTGGCTT	14	Campylobacter jejuni 81-176 plasmid pVir	NC_005012
plasmid	TCCCGGAA	CCATTTTTGTAATCATGCAGAAGATTAAGG	CAGTGGTT	2	TCATTTTTGTAATCATGCAGAAGATTAATG	771	Campylobacter coli 15-537360 plasmid pCC42yr	NC_022656
plasmid	TAAAATCA	TTATTATATTTAACTATTTCGCTCATTTGC	ATATCCAA	2	TTATGATATTTAACTATATCGCTCATTTGC	971	Campylobacter coli plasmid p3384	NC_007142
plasmid	TAAAATCG	TTGTGATATTTAACTATTTCGCTCATTTGC	ATATCCAA	2	TTATGATATTTAACTATATCGCTCATTTGC	971	Campylobacter jejuni strain S4-2 CC plasmid pTIW94	NC_021493
plasmid	TAAAATCA	TTATGATATTTAACTATTTCACTCATTTGC	ATATCCTA	2	TTATGATATTTAACTATATCGCTCATTTGC	971	Campylobacter peloridis LMG 23910 plasmid pPEL2	NZ_CP007768
plasmid	TCGCCTTT	TTTTATATATTGATTAGCAACTTCTGCTTT	ACGCCCAA	2	TTTGATATATTGATTTGCAACTTCTGCTTT	982	Campylobacter jejuni 81-176 plasmid pVir	NC_005012
plasmid	ATAACCCC	ACCAATTTTTCTAACTTCGAGAATGCTTTC	GAGAATGC	2	ACCAATTTTTCTAACTTCTAGAATACTTTC	985	Campylobacter coli RM1875 plasmid pRM1875_35kb	NZ_CP007184
prophage	TTTTACCA	TGCATTAAGTCGTAAAATTCTTTGCAAGC	CTGCCTGC	2	TACATTAAGTCGTAAAATTCTTTGCACGC	991	Campylobacter phage CJIE4-2	KF751794
prophage	ATGAAGTT	CCTTCAAGTCGTGCAATTTTAATACACTGG	GGAAACAC	2	CCAGCAAGTCGTGCAATTTTAATACACTGG	451	Campylobacter phage CJIE4-5	KF751797
prophage	CAAGTCGT	GCAATTTTAATACACTGGGGAAACACTGAT	AAAGACAC	2	GCAATTTTAATACATTGGGGAAACACTGAC	581	Campylobacter phage CJIE4-2	KF751794
prophage	CAAGTCGT	GCAATTTTAATACACTGGGGAAACACTGAT	AAAGACAC	2	GCAATTTTAATGCATTGGGGAAACACTGAT	582	Campylobacter phage CJIE4-4	KF751796
prophage	AGAATTTT	ATCATCATCATCTAAAACCTTAAATTTACC	AATAACGC	2	TTCTTCATCATCTAAAACCTTAAATTTACC	905	Campylobacter phage CJIE4-3	KF751795
prophage	CACAGAGC	TTTATGCTACGCTAAGTCAAGCTACTAAGG	ATGTTACA	2	TTTATGCTACGCTAAGCCAAGCCACTAAGG	931	Campylobacter phage CJIE4-2	KF751794
prophage	TTCCTTTC	TTTTAATTTCTTAATTTTATCAAAATGTTA	CTTAAGTA	2	TTTTAATTTCTTAATTTTATCAAAAAGTTT	954	Campylobacter phage CJIE4-1	KF751793
prophage	AAGATTTT	AAAAGGTGATAATTTTAAAGAATGTTACCA	AGTGCCTA	2	AAAAGGTGAGAATTTTAAAGAATGTTATCA	969	Campylobacter phage CJIE4-5	KF751797
prophage	CAAGTCGT	GCAATTTTAATACACTGGGGAAACACTGAC	AAAGACAC	2	GCAATTTTAATACACTGGGGAAACACTTAT	1064	Campylobacter phage CJIE4-1	KF751793
phage	TAAATTTC	ATAATACCAAATAAAAAAGTTAGCTTTGAT	GGAAATAC	3	ATAATACCTAATAAAAAAGTAAGTTTTGAT	321	Campylobacter phage CP8	KF148616
phage	TAAATTTC	ATAATACCAAATAAAAAAGTTAGCTTTGAT	GGAAATAC	3	TTAATACCAAATAAAAAAGTAAGCTTCGAT	867	Campylobacter phage NCTC12673	GU296433
phage	AGAATATT	ATTAAAATACTTTGTCCTTTTGATGATGAG	AGTTCTAA	3	ATAAAAATACTTTGCCCTTTTGATGACGAG	1019	Campylobacter phage NCTC12673	GU296433
plasmid	ATTATCAA	CAGATGTTAAAACAATACGAACAGATGATT	AAAGACAC	3	CAAATGTTAAAACAATACGAGCAAATGATT	406	Campylobacter jejuni 81-176 plasmid pVir	NC_005012
plasmid	TAAAATCA	TTATGGTATTTAACTATATTGCTCATTTGT	ATATCCAA	3	TTATGATATTTAACTATATCGCTCATTTGC	971	Campylobacter jejuni plasmid pCJ419	NC_004997
plasmid	TAAAATCG	TTGTGATATTTAACTATTTCGCTCATTTGA	ATATCCAA	3	TTATGATATTTAACTATATCGCTCATTTGC	971	Campylobacter coli RM2228 plasmid pCC2228-2	NC_008050
plasmid	CTATGATT	TGCGATAATCGGGGTAAGGGGGCGTGAAGC	TTTCCTAG	3	TGCGATAATCGGGGTGCGGGGGCGTGAAGT	977	Campylobacter jejuni plasmid pCJ419	NC_004997
plasmid	ATTACTCC	ACCTATTTTTCTTACTTCAAGAATACTTTC	AAGTATGC	3	ACCAATTTTTCTAACTTCTAGAATACTTTC	985	Campylobacter jejuni IA3902 plasmid pVir	NC_017284
prophage	GAATTTTT	TCTTCATCATCTAAAACCTTAAATTTACC	AATAACAC	3	TCATCATCACTTAAAACCTTAAATTTACC	768	Campylobacter phage CJIE4-1	KF751793
prophage	TCAAGTTG	TAGCTTTAAATTCTTTTCATAGTTCTCAAA	TGAAATAT	3	CAATTTTAAATTCTTTTCATAGTTCTCAAA	418	Campylobacter phage CJIE4-3	KF751795
prophage	ATGAAGTT	CCTTCAAGTCGTGCAATTTTAATACACTGG	GGAAACAC	3	CCAGCAAGCCGTGCAATTTTAATACACTGG	450	Campylobacter phage CJIE4-5	KF751797
prophage	ATGAAGTT	CCTTCAAGTCGTGCAATTTTAATACACTGG	GGAAACAC	3	CCATCAAGTCGTGCAATTTTATTACATTGG	454	Campylobacter phage CJIE4-1	KF751793
prophage	TTATTATC	CACTCTAGCTTTTGCTGATTATACACAATA	CAAACCAA	3	AACTCTAGCCTTAGCTGATTATACACAATA	1010	Campylobacter phage CJIE4-5	KF751797
phage	ATAATACC	CTGTTCTACTTTCAATTTCTTTAATTTTTTC	TAGCATAC	4	CTGTACGACTTTCAATCTCTTTAATTGTTTG	506	Campylobacter phage NCTC12673	GU296433
phage	CTGATAAT	AGTGTATATATTGAAATAAAATATAGAGTT	AGAGATAC	4	AGTGTGTATGTTGAAATAAATTATAGAGTA	303	Campylobacter phage CP8	KF148616
phage	TAAATTTC	ATAATACCAAATAAAAAAGTTAGCTTTGAT	GGAAATAC	4	ATAATTCCTAATAAAAAAGTAAGTTTTGAT	322	Campylobacter phage CP8	KF148616
phage	TAAATTTC	ATAATACCAAATAAAAAAGTTAGCTTTGAT	GGAAATAC	4	ATAATTCCTAATAAAAAAGTAAGTTTTGAT	322	Campylobacter phage CP30A	JX569801
phage	TTCATAAT	TATAAAATTCTTTTTTACTTGTGTAGCAAT	TAAAATAT	4	TATATAACTCATTTTTACTTGTGTAACAAT	726	Campylobacter phage NCTC12673	GU296433
phage	TTAAAGGT	TGTTTAACACTATAAATGTGGTCTCTTATA	ATTGATAC	4	TGCTTAACTCTATAAATATGGTCTCTTATT	833	Campylobacter phage CP21 complete sequence	HE815464
phage	ATTTAAGA	TTTGGTGGAGGTCAAACAAAAGAAGAAATT	AAGAATAC	4	TTTGGCGGAGGACAAACAAAAGAAGAAGTA	947	Campylobacter phage CP8	KF148616
phage	AGAATATT	ATTAAAATACTTTGTCCTTTTGATGATGAA	AATTCTAA	4	ATAAAAATACTTTGCCCTTTTGATGACGAG	1019	Campylobacter phage CP30A	JX569801

plasmid	TTTTGAAT	TGACTTATCAAGAACCACAAGACCCTAAG	GAATATAA	4	TGATTTATCAAGAACCGCAAGATCCCAAG	992	Campylobacter jejuni 81-176 plasmid pVir	NC_005012
plasmid	CGCTAGCT	TATTTTTACGCATAAAATCAACAAAATTTT	TTAGATTT	4	TATTTTAACGCTTAAAATCAAGAAAATATT	754	Campylobacter jejuni IA3902 plasmid pVir	NC_017284
plasmid	GATACAGG	TTTTGGAATAAAAGAATTTACTATGATAGA	TGAACCAC	4	TTTTGGTGTGAAAGAATTCACTATGATAGA	960	Campylobacter lari RM2100 megaplasmid pCL2100	NC_012040
plasmid	GATCATTT	TTAATAAAATCATCATCTATAGTTTTTTTC	TCAACCAC	4	TTAATAAAATCATCATCCACAGTCTTTTTT	975	Campylobacter jejuni 81-176 plasmid pTet	NC_006135
plasmid	CTATGATT	TGCGGTAATTGGGGTAAGGGGGCGTGAAGT	TAGCCTAG	4	TGCGATAATCGGGGTGCGGGGGCGTGAAGT	977	Campylobacter coli plasmid p3386	NC_007143
plasmid	CCCATTAT	CACGCCCACGCGGTATTTTTTACACTCGAT	AACAATGG	4	CACGCTCACGCGGTATTTTTTACTCTAGAC	1060	Campylobacter coli plasmid p3384	NC_007142
prophage	AAGATTTA	AGAATACCTGAAGGAAATTATAATTTAAAA	AGACATAG	4	AGAATACCAGAAGGCATTTATAATTTAGAA	248	Campylobacter phage CJIE4-2	KF751794
prophage	TGAAAGGA	GATAAAAGTTTAAGTAGGTTAAAGTTTACA	CCCCGCTT	4	AGATAAAGTTTAAGTAGGTTAAAGTTTACA	259	Campylobacter phage CJIE4-4	KF751796
prophage	AGAATTTT	TTCTTCATCATCTAAAACCTTAAATTTACC	AATAACAC	4	ATCATCATCACTTAAAACCTTAAATTTACC	358	Campylobacter phage CJIE4-1	KF751793
prophage	TATATTCT	TTTGAAGGTTGTTCTGAAGATGAAGCTTTT	AAATATAA	4	TTTGAAGACTGCCCTGAAGATGAAGCTTTT	1001	Campylobacter phage CJIE4-1	KF751793
phage	TCTAAATT	AAGTTTATTTTTACATAATTTAAATAAACA	ATCTTTGG	5	AAGTATATTTTTAGATAATTTGAATATAAA	143	Campylobacter phage CP220	FN667788
phage	TTTGCTCT	ATTGATATTAGCATTCATACCAGTAAACAA	AAGTATAC	5	ATTGAAATTAACATTCATACCAGCAAGTAA	383	Campylobacter phage CPt10	FN667789
phage	AAAAATGT	TTAAAATATATAAATAATAATACAAAAAAA	AAATTAAT	5	CAAGAATATATAAATAATAATATAAAAGAA	409	Campylobacter phage vB_CcoM-IBB_35	HM246724
phage	TTTGTTTC	TGCATCAAATTCAATTCCATCAGGAATTAA	TGCTATAC	5	CGCGTCAAATTCAATTCCATCAGGGACTAT	475	Campylobacter phage CP81	FR823450
phage	TAATGAGA	GAATCTTTAGTTAATACTATTAAAAGAGGA	CAAGATAT	5	GAATCTTTAGTTAACACGATACAAAGAGGT	553	Campylobacter phage CP8	KF148616
phage	TAATAAAA	TTAAAGAAAGATTGACACAATGGCGTGAAG	AAAGACAT	5	TAAAAGAAAGATTATCTAAATGGCGTGAAG	658	Campylobacter phage NCTC12673	GU296433
phage	AAGAGTTA	CAATAATGCTTTTGATTTAAAAGATGAATA	TTATACAC	5	TAATAAAGCATTTGATTTGAAAGATGAGTA	678	Campylobacter phage CP81	FR823450
phage	TTCATAAT	TATAAAATTCTTTTTTACTTGTGTAGCAAT	TAAAATAT	5	TATATAACTCATTTTTACTTGTGCAACAAT	725	Campylobacter phage NCTC12673	GU296433
phage	ATATCCTT	CATCTTTAATAAACTTATTTAACTCATCAT	CATAACCA	5	TATCTTTGATAAACTTATTTAAGTTCTCAT	738	Campylobacter phage CP81	FR823450
phage	TAAAATCC	TGTACGACTTTCAATCTCTTTAATTTTTTC	TAGCATAC	5	TGTGCGACTTTTAATCTTTTTAATTGTTTG	848	Campylobacter phage CP30A	JX569801
phage	TAAAATCC	TGTACGACTTTCAATCTCTTTAATTTTTTC	TAGCATAC	5	TGTTCTACTTTCAATCTCTTTGATTGTTTG	852	Campylobacter phage CP30A	JX569801
phage	AAGTGAAT	TTGATTTTAGGATTTGGGATAATGTTGAAA	AGAAGTAT	5	TTGATTTTAGGATTTGGGATAATAAATATA	915	Campylobacter phage CP8	KF148616
phage	TCATAACC	ATAACGACTTTCAATCTCTTTAATTTTTTC	TAGCATAC	5	TTTGCGACTTTCAATCTCTTTAATTGTTTG	946	Campylobacter phage CP81	FR823450
phage	AGAATATT	ACTAAAATACTTTGTCCTTTTGATGATGAA	AGTTCTAA	5	ATAAAAATACTTTGCCCTTTTGATGACGAG	1019	Campylobacter phage CPX	JN132397
plasmid	TCGCTAGC	TTATTTTTACGCATAAAATCAACAAAATTTT	TTAGATTT	5	TTATTTGAACGCTTAAAATCAAGAAAATATT	888	Campylobacter jejuni IA3902 plasmid pVir	NC_017284
plasmid	ATCCTGAA	AATTATTATATCGAAGATATTAAACAAATT	GCTTATCC	5	AATAATGATATCGAAGTTATTAATCTAATT	32	Campylobacter coli 15-537360 plasmid pCC42yr	NC_022656
plasmid	TACGCTCT	CCGCCTACCAAAATACAAGAAAGAATAGGG	CGTGATAC	5	CCACCTACTAGAATACAACTAAGAATAGGG	448	Campylobacter jejuni 00-2544 plasmid	NC_022354
plasmid	ATTCAGTT	GCCTAGCTTTACAGAGCAAGAACAAAACCT	TTTGTTTT	5	GCCTAGTTTTACAGAACAAGAGCAAAATTT	599	Campylobacter jejuni plasmid pCJ01	NC_008438
plasmid	CGCTAGCT	TATTTTTACGCATAAAATCAACAAAATTTT	TTAGATTT	5	TATTTGAACGCTTAAAATCAAGAAAATATT	753	Campylobacter jejuni IA3902 plasmid pVir	NC_017284
plasmid	TAAAATCA	TTATGGTATTTAACTATATCGCTCATAGAT	TTTCCTTG	5	TTATGATATTTAACTATATCGCTCATTTGC	971	Campylobacter lari plasmid pCL300	NC_006975
plasmid	GATCATTT	TTAATAAAATCATCATTTATAGTTTTTTTC	TCAACCAC	5	TTAATAAAATCATCATCCACAGTCTTTTTT	975	Campylobacter jejuni strain 01-1512 plasmid pCj1	NZ_CP010073
plasmid	ATCCTGAA	AATTATTATATCGAAGATATTAAACAAATTG	CTTATCCT	5	AATAATGATATCGAAGTTATTAATCTAATTG	1052	Campylobacter coli 15-537360 plasmid pCC42yr	NC_022656
plasmid	CCCACTAT	CACGCCCACGCAGTATTTTTTACACTTGAT	AACAATGG	5	CACGCTCACGCGGTATTTTTTACTCTAGAC	1060	Campylobacter coli CVM N29710 plasmid pN29710-2	NC_022348
plasmid	CCCATTAT	CACGCCCACGCAGTATTTTTTACACTCGAT	AACAATGG	5	CACGCTCACGCGGTATTTTTTACTCTAGAC	1060	Campylobacter coli RM1875 plasmid pRM18753.3kb	NZ_CP007186
plasmid	CCCATTAT	CACGCCCACGCAGTATTTTTTACACTCGAT	AACAATGG	5	CACGCTCACGCGGTATTTTTTACTCTAGAC	1060	Campylobacter coli RM2228 plasmid pCC2228-2	NC_008050
plasmid	CCCATTAT	CACGCCCACGCAGTATTTTTTACACTCGAT	AACAATGG	5	CACGCTCACGCGGTATTTTTTACTCTAGAC	1060	Campylobacter jejuni strain S4-2 CC plasmid pTIW94	NC_021493
prophage	CGTGGGAT	ACTTGCACCATCAGTTTTAAATCCTTTTGG	CACAATGC	5	ACTTGCTCCATCAGTTTTATACCCTTTTTC	226	Campylobacter phage CGC-2007	EF694693
prophage	AGAATTTT	TTCTTCATCATCTAAAACCTTAAATTTACC	AATAACAC	5	ATCATCATCACTTAAAATCTTAAATTTACC	359	Campylobacter phage CJIE4-1	KF751793
prophage	TCACCCAC	AGCTTTATAACGACCACTTTCAAAATTTTA	AAAGATAT	5	ATTTATATAACGACCATTTCCAAAATTTTA	394	Campylobacter phage CJIE4-2	KF751794
prophage	AAAAAGGA	AATAAGATTTATGAAGGAGATATTTTATAT	TCTTTTGA	5	AATAAAGTTTATGAAGGTGATATATTGTAT	967	Campylobacter phage CJIE4-1	KF751793

Table S7. Diversity of CRISPR spacers in 1,919 *C. jejuni* and 23 *C. coli* genome sequences
The first column gives the genome code (Cj = C. jejuni; Cd = C. jejuni subsp doylei; Cc = C. coli). The Project ID is a study-specific identifier for database lookup.
Clonal complex and sequence type (ST) as per the definitions available at the Campylobacter pubMLST website.
Spacer alleles represent spacer sequences listed in Table S5.
The number of spacers and their sequence was determined using the CRISPR Recognition Tool (Bland et al, 2007).

Isolate	Project ID	clonal complex	ST	first spacer	last spacer	number spacers	spacer 1	spacer 2	spacer 3	spacer 4	spacer 5	spacer 6	spacer 7	spacer 8	spacer 9	spacer 10
Cd_ATCC_49349	0106	0000	62	251	473	5	251	600	193	98	473					
Cj_OXC7901	2503	0000	131	939	408	3	939	484	408							
Cj_OXC8191	2993	0000	407	736	798	3	736	45	798							
Cj_OXC6832	0687	0000	436	7	874	4	7	144	818	874						
Cj_OXC4746	0709	0000	436	7	725	7	7	818	28	729	551	387	725			
Cj_OXC7013	1192	0000	436	7	792	7	7	818	28	729	551	725	792			
Cj_OXC7399	2677	0000	436	7	725	7	7	818	28	729	551	387	725			
Cj_OXC8087	2742	0000	436	7	725	8	7	7	818	28	729	551	387	725		
Cj_OXC8297	3070	0000	436	7	387	5	7	818	729	551	387					
Cj_OXC4645	1909	0000	436	938	23	3	938	868	23							
Cj_OXC4653	1913	0000	436	938	23	3	938	868	23							
Cj_OXC5798	1600	0000	441	733	632	7	733	680	963	802	359	322	632			
Cj_OXC6546	0178	0000	449	934	426	4	934	519	691	426						
Cj_OXC7852	2207	0000	449	934	426	4	934	519	691	426						
Cj_Dg47b	2881	0000	586	172	196	3	172	289	196							
Cj_OXC5743	1003	0000	586	384	138	5	384	111	819	938	138					
Cj_OXC4557	2542	0000	693	17	769	4	17	116	62	769						
Cj_OXC4808	1141	0000	879	743	229	4	743	938	487	229						
Cj_OXC7454	1810	0000	879	743	229	4	743	938	487	229						
Cj_OXC6675	0546	0000	986	586	616	3	586	750	616							
Cj_OXC6944	0739	0000	986	586	565	6	586	750	616	447	256	565				
Cj_OXC5904	1715	0000	986	586	616	3	586	750	616							
Cc_RM4661	C051	0000	1135	7	352	15	7	11	21	391	822	592	415	498	322	701
Cc_Dg277	C390	0000	1143	166	871	10	166	293	113	833	482	961	879	132	879	871
Cj_OXC4802	1138	0000	1409	23	827	7	23	776	823	452	431	523	827			
Cc_dfvf1912	C260	0000	1576	1007	1011	1	1007									
Cd_26997	0105	0000	1845	783	672	5	783	485	485	277	672					
Cj_OXC8638	3391	0000	1962	849	83	3	849	451	83							
Cj_OXC8639	3392	0000	1962	849	83	3	849	451	83							
Cj_OXC6844	1042	0000	2118	3	951	3	3	912	951							
Cj_OXC8864	3654	0000	2133	753	441	3	753	29	441							
Cj_OXC8622	3384	0000	2211	923	521	3	923	433	521							
Cj_OXC6279	0245	0000	2258	439	448	2	439	448								
Cj_OXC7517	1938	0000	2258	439	580	3	439	448	580							
Cj_OXC8390	3166	0000	2258	439	537	5	439	448	580	685	537					
Cj_OXC8391	3167	0000	2258	439	537	5	439	448	580	685	537					
Cj_OXC7825	2184	0000	2274	548	701	7	548	548	111	242	286	524	701			
Cj_OXC6446	0381	0000	3029	23	827	5	23	776	452	431	827					
Cj_Dg22	2884	0000	3471	494	100	20	494	766	345	931	957	142	466	933	859	24
Cj_414	0040	0000	3704	664	135	33	664	929	574	704	146	762	459	314	273	273
Cj_BV414	3139	0000	3704	664	135	33	664	929	574	704	146	762	459	314	273	273
Cj_OXC7458	1813	0000	3923	471	267	3	471	789	267							
Cj_OXC5088	2276	0000	4430	87	894	4	87	856	943	894						
Cj_OXC4906	2050	0000	4766	743	24	9	743	487	775	860	649	874	597	618	24	
Cj_Dg78a	2855	0000	5129	494	954	12	494	931	957	142	466	525	697	486	626	606
Cj_OXC5457	0936	0000	5142	441	229	3	441	634	229							
Cj_OXC8323	3096	0000	5155	703	799	3	703	183	799							
Cj_OXC7478	1830	0000	5707	380	656	7	380	921	748	656	748	544	656			
Cc_OXC6551	C064	0000	5755	889	739	8	889	239	656	948	357	317	402	739		
Cj_OXC6557	0188	0000	5756	675	883	6	675	776	111	132	42	883				
Cj_UCT046_R	2015	0000	6094	887	177	5	887	959	532	376	177					
Cc_OXC5681	C156	0000	6133	991	998	16	991	999	1000	1001	1002	1003	1004	1005	1006	992
Cc_OXC7411	C221	0000	6267	987	990	5	987	988	989	989	990					
Cj_OXC8024	2640	0000	6449	965	527	4	965	362	80	527						
Cj_OXC8056	2644	0000	6449	965	527	4	965	362	80	527						
Cj_Dg196	1966	0000	6561	188	870	6	188	278	660	89	795	870				
Cj_Dg61	1964	0000	6561	494	545	17	494	931	957	142	466	525	697	771	907	328
Cj_Dg62a	1965	0000	6561	494	545	16	494	931	957	142	466	525	697	771	907	328
Cj_Dg204	2913	0000	6561	494	957	3	494	931	957							
Cj_Dg189	1969	0000	6562	188	405	7	188	278	660	89	795	795	405			
Cj_Dg23b	2857	0000	6562	188	137	6	188	278	660	89	795	137				
Cj_Dg300	2879	0000	6562	188	405	6	188	278	660	89	795	405				
Cj_Dg302	2886	0000	6562	188	405	6	188	278	660	89	795	405				
Cj_Dg197	2898	0000	6562	188	405	6	188	278	660	89	795	405				
Cj_Dg147	2911	0000	6562	188	795	5	188	278	660	89	795					
Cj_Dg226	2942	0000	6562	188	405	6	188	278	660	89	795	405				
Cj_Dg150	2945	0000	6562	188	795	5	188	278	660	89	795					
Cj_Dg156	2951	0000	6562	188	795	5	188	278	660	89	795					
Cj_Dg338	2964	0000	6562	188	795	5	188	278	660	89	795					
Cj_Dg192	1971	0000	6564	142	378	21	142	466	525	697	486	771	518	626	900	4
Cj_Dg358	2906	0000	6564	142	378	21	142	466	525	697	486	771	518	626	900	4
Cc_E120586C	C375	0000	6935	1008	1022	4	1008	1009	1010	1011						
Cc_E120613C	C378	0000	6936	1016	666	9	1016	1017	1018	1019	1020	973	975	1021	1022	
Cj_OXC8321	3094	0000	6976	7	209	4	7	17	297	209						
Cj_OXC8441	3208	0000	7182	174	773	3	174	219	773							
Cj_Dg63a	2885	0000	7258	14	68	4	14	790	782	68						
Cj_Dg217	2861	0000	7276	14	75	7	14	14	790	68	462	107	75			
Cj_Dg210	2929	0000	7276	14	75	6	14	790	68	462	107	75				
Cj_Dg346	2919	0000	7278	494	605	13	494	931	957	142	466	525	697	486	626	606
Cj_Dg334	2862	0000	7279	494	386	22	494	766	345	931	957	142	599	608	792	879
Cj_OXC8711	3463	0000	7306	7	926	7	7	28	29	53	334	144	926			
Cj_OXC8732	3476	0000	7306	7	926	7	7	28	29	53	334	144	926			
Cj_OXC8733	3477	0000	7306	7	926	7	7	28	29	53	334	144	926			
Cj_OXC8271	3050	0000	N/A	7	111	4	7	11	25	111						
Cj_OXC5828	1700	0000	N/A	22	201	7	22	383	103	274	741	457	201			
Cj_OXC8949	3729	0000	N/A	61	342	4	61	17	127	342						
Cj_OXC8385	3161	0000	N/A	172	74	4	172	289	417	74						
Cj_Dg289	2916	0000	N/A	188	89	4	188	278	660	89						
Cj_Dg348	2927	0000	N/A	188	89	4	188	278	660	89						
Cj_Dg295a	2931	0000	N/A	188	89	4	188	278	660	89						
Cj_Dg164	2933	0000	N/A	188	89	4	188	278	660	89						
Cj_Dg105	2948	0000	N/A	188	89	4	188	278	660	89						
Cj_Dg157	2956	0000	N/A	188	89	4	188	278	660	89						
Cj_OXC5440	0927	0000	N/A	216	29	2	216	29								
Cj_ARI_813_R	2032	0000	N/A	323	19	3	323	712	19							
Cj_Dg233	2903	0000	N/A	345	184	8	345	931	957	563	756	207	545	184		
Cj_Dg234	2947	0000	N/A	345	184	8	345	931	957	563	756	207	545	184		
Cj_E120052CJ_S11	2851	0000	N/A	381	499	54	381	770	313	124	197	652	456	197	778	791
Cj_OXC8903	3687	0000	N/A	490	407	5	490	52	60	7	407					
Cj_Dg36b	2878	0000	N/A	494	144	22	494	931	957	142	466	525	697	486	811	771
Cj_Dg301b	2882	0000	N/A	494	144	21	494	931	957	142	466	525	697	486	811	771
Cj_Dg179	2891	0000	N/A	494	144	21	494	931	957	142	466	525	697	486	811	771
Cj_Dg276	2961	0000	N/A	494	144	21	494	931	957	142	466	525	697	486	811	771
Cj_OXC5338	0870	0000	N/A	596	425	2	596	425								
Cj_OXC8054	2808	0000	N/A	703	799	3	703	183	799							
Cj_305	0037	0000	N/A	743	261	4	743	938	624	261						
Cj_HM908634	1978	0000	N/A	743	338	9	743	938	487	284	57	677	728	759	338	
Cj_CAMSA2113_rr	2587	0000	N/A	743	474	4	743	938	487	474						
Cj_CAMSA3844_rr	2595	0000	N/A	743	677	5	743	938	487	284	677					
Cj_OXC5803	1605	0000	N/A	818	823	4	818	722	851	823						
Cj_OXC8849	3641	0000	N/A	878	916	3	878	561	916							
Cj_Dg18a	2887	0000	N/A	924	724	4	924	910	480	724						
Cj_Dg43a	2888	0000	N/A	924	480	3	924	910	480							
Cj_Dg201	2899	0000	N/A	924	724	4	924	910	480	724						

Isolate	spacer 11	spacer 12	spacer 13	spacer 14	spacer 15	spacer 16	spacer 17	spacer 18	spacer 19	spacer 20	spacer 21	spacer 22
Cc_RM4661	203	104	327	940	352							
Cj_Dg22	761	731	800	606	165	170	490	756	216	100		
Cj_414	903	120	295	477	247	350	840	862	458	670	71	803
Cj_BV414	903	120	295	477	247	350	840	862	458	670	71	803
Cj_Dg78a	82	954										
Cc_OXC5681	993	994	995	996	997	998						
Cj_Dg61	328	552	633	241	410	285	545					
Cj_Dg62a	552	633	241	410	285	545						
Cj_Dg192	40	530	47	476	157	623	270	220	226	806	378	
Cj_Dg358	40	530	47	476	157	623	270	220	226	806	378	
Cj_Dg346	82	436	605									
Cj_Dg334	29	128	147	374	91	814	179	153	299	777	786	386
Cj_E120052CJ_S11	228	638	516	119	369	928	825	958	215	121	72	479
Cj_Dg36b	518	626	572	907	328	552	633	241	410	285	144	144
Cj_Dg301b	518	626	572	907	328	552	633	241	410	285	144	
Cj_Dg179	518	626	572	907	328	552	633	241	410	285	144	
Cj_Dg276	518	626	572	907	328	552	633	241	410	285	144	

Isolate	spacer 23	spacer 24	spacer 25	spacer 26	spacer 27	spacer 28	spacer 29	spacer 30	spacer 31	spacer 32	spacer 33
Cj_414	526	245	612	642	767	56	706	325	123	214	135
Cj_BV414	526	245	612	642	767	56	706	325	123	214	135
Cj_E120052CJ_S11	953	627	875	382	51	280	205	202	603	472	126

Isolate	spacer 34	spacer 35	spacer 36	spacer 37	spacer 38	spacer 39	spacer 40	spacer 41	spacer 42	spacer 43	spacer 44	spacer 45	spacer 46	spacer 47	spacer 48	spacer 49	spacer 50	spacer 51	spacer 52	spacer 53	spacer 54
Cj_E120052CJ_S11	252	399	478	38	308	492	661	682	332	882	779	370	896	238	886	145	904	942	90	897	499

Isolate	number
Cd_ATCC_49349	0106
Cj_OXC7901	2503
Cj_OXC8191	2993
Cj_OXC6832	0687
Cj_OXC4746	0709
Cj_OXC7013	1192
Cj_OXC7399	2677
Cj_OXC8087	2742
Cj_OXC8297	3070
Cj_OXC4645	1909
Cj_OXC4653	1913
Cj_OXC5798	1600
Cj_OXC6546	0178
Cj_OXC7852	2207
Cj_Dg47b	2881
Cj_OXC5743	1003
Cj_OXC4557	2542
Cj_OXC4808	1141
Cj_OXC7454	1810
Cj_OXC6675	0546
Cj_OXC6944	0739
Cj_OXC5904	1715
Cc_RM4661	C051
Cc_Dg277	C390
Cj_OXC4802	1138
Cc_dfvf1912	C260
Cd_26997	0105
Cj_OXC8638	3391
Cj_OXC8639	3392
Cj_OXC6844	1042
Cj_OXC8864	3654
Cj_OXC8622	3384
Cj_OXC6279	0245
Cj_OXC7517	1938
Cj_OXC8390	3166
Cj_OXC8391	3167
Cj_OXC7825	2184
Cj_OXC6446	0381
Cj_Dg22	2884
Cj_414	0040
Cj_BV414	3139
Cj_OXC7458	1813
Cj_OXC5088	2276
Cj_OXC4906	2050
Cj_Dg78a	2855
Cj_OXC5457	0936
Cj_OXC8323	3096
Cj_OXC7478	1830
Cc_OXC6551	C064
Cj_OXC6557	0188
Cj_UCT046_R	2015
Cc_OXC5681	C156
Cc_OXC7411	C221
Cj_OXC8024	2640
Cj_OXC8056	2644
Cj_Dg196	1966
Cj_Dg61	1964
Cj_Dg62a	1965
Cj_Dg204	2913
Cj_Dg189	1969
Cj_Dg23b	2857
Cj_Dg300	2879
Cj_Dg302	2886
Cj_Dg197	2898
Cj_Dg147	2911
Cj_Dg226	2942
Cj_Dg150	2945
Cj_Dg156	2951
Cj_Dg338	2964
Cj_Dg192	1971
Cj_Dg358	2906
Cc_E120586C	C254
Cc_E120613C	C254
Cj_OXC8321	3094
Cj_OXC8441	3208
Cj_Dg63a	2885
Cj_Dg217	2861
Cj_Dg210	2929
Cj_Dg346	2919
Cj_Dg334	2862
Cj_OXC8711	3463
Cj_OXC8732	3476
Cj_OXC8733	3477
Cj_OXC8271	3050
Cj_OXC5828	1700
Cj_OXC8949	3729
Cj_OXC8385	3161
Cj_Dg289	2916
Cj_Dg348	2927
Cj_Dg295a	2931
Cj_Dg164	2933
Cj_Dg105	2948
Cj_Dg157	2956
Cj_OXC5440	0927
Cj_ARI_813_R	2032
Cj_Dg233	2903
Cj_Dg234	2947
Cj_E120052CJ_S11	2851
Cj_OXC8903	3687
Cj_Dg36b	2878
Cj_Dg301b	2882
Cj_Dg179	2891
Cj_Dg276	2961
Cj_OXC5338	0870
Cj_OXC8054	2808
Cj_305	0037
Cj_HM908634	1978
Cj_CAMSA2113_rr	2587
Cj_CAMSA3844_rr	2595
Cj_OXC5803	1605
Cj_OXC8849	3641
Cj_Dg18a	2887
Cj_Dg43a	2888
Cj_Dg201	2899

Cj_Dg381	2907	0000	N/A	924	724	4	924	910	480	724											2907
Cc_76339	C027	0000	N/A	967	970	4	967	968	969	970											C027
Cc_8808	C254	0000	N/A	971	976	14	971	977	978	979	980	981	982	983	984	972	973	974	975	976	C254
Cc_E120587C	C376	0000	N/A	1012	1007	1	1012														C254
Cc_E120595C	C377	0000	N/A	1012	275	5	1012	1013	1014	1015	1015										C254
Cj_HB93-13	0069	0021	8	938	915	3	938	281	915												0069
Cj_OXC129	0175	0021	19	541	874	4	541	860	649	874											0175
Cj_OXC6303	0266	0021	19	541	341	6	541	860	649	874	687	341									0266
Cj_OXC6325	0285	0021	19	541	874	4	541	860	649	874											0285
Cj_OXC6538	0462	0021	19	541	874	4	541	860	649	874											0462
Cj_OXC6703	0572	0021	19	541	341	6	541	860	649	874	687	341									0572
Cj_OXC6706	0574	0021	19	541	341	6	541	860	649	874	687	341									0574
Cj_OXC7160	0850	0021	19	541	403	5	541	860	649	874	403										0850
Cj_OXC7163	0853	0021	19	541	340	7	541	860	649	874	687	341	340								0853
Cj_OXC5476	0948	0021	19	541	874	4	541	860	649	874											0948
Cj_OXC7253	1277	0021	19	541	341	5	541	860	874	687	341										1277
Cj_OXC7256	1279	0021	19	541	874	4	541	860	649	874											1279
Cj_OXC7320	1338	0021	19	541	874	4	541	860	649	874											1338
Cj_OXC7211	1396	0021	19	541	341	6	541	860	649	874	687	341									1396
Cj_OXC5647	1584	0021	19	541	341	6	541	860	649	874	687	341									1584
Cj_OXC5818	1617	0021	19	541	341	6	541	860	649	874	687	341									1617
Cj_OXC5887	1659	0021	19	541	874	4	541	860	649	874											1659
Cj_OXC5871	1708	0021	19	541	874	4	541	860	649	874											1708
Cj_OXC5899	1713	0021	19	541	341	6	541	860	649	874	687	341									1713
Cj_OXC7348	1725	0021	19	541	644	6	541	860	649	874	873	644									1725
Cj_ARI_1232_R	2024	0021	19	541	874	4	541	860	649	874											2024
Cj_CJ7256_R	2040	0021	19	541	874	4	541	860	649	874											2040
Cj_OXC5043	2254	0021	19	541	403	5	541	860	649	874	403										2254
Cj_OXC5081	2272	0021	19	541	341	5	541	860	649	874	341										2272
Cj_OXC7748	2464	0021	19	541	236	8	541	860	649	874	111	425	107	236							2464
Cj_OXC7887	2492	0021	19	541	341	6	541	860	649	874	687	341									2492
Cj_OXC7926	2521	0021	19	541	341	5	541	860	649	687	341										2521
Cj_OXC7929	2524	0021	19	541	341	6	541	860	649	874	687	341									2524
Cj_Dg375	2946	0021	19	541	874	4	541	860	649	874											2946
Cj_OXC8190	2992	0021	19	541	874	4	541	860	649	874											2992
Cj_OXC8346	3118	0021	19	541	653	6	541	860	649	874	962	653									3118
Cj_OXC8045R	3141	0021	19	541	874	4	541	860	649	874											3141
Cj_OXC8582	3408	0021	19	541	341	5	541	860	874	687	341										3408
Cj_OXC8684	3445	0021	19	541	874	4	541	860	649	874											3445
Cj_OXC8685	3446	0021	19	541	874	4	541	860	649	874											3446
Cj_OXC8768	3574	0021	19	541	874	4	541	860	649	874											3574
Cj_OXC8775	3581	0021	19	541	341	6	541	860	649	874	687	341									3581
Cj_OXC8794	3598	0021	19	541	874	4	541	860	649	874											3598
Cj_OXC6548	0180	0021	19	775	465	6	775	541	860	649	874	465									0180
Cj_OXC4503	0221	0021	19	775	300	4	775	649	874	300											0221
Cj_OXC6370	0327	0021	19	775	784	6	775	541	860	649	874	784									0327
Cj_OXC6384	0336	0021	19	775	784	6	775	541	860	649	874	784									0336
Cj_OXC6394	0344	0021	19	775	874	5	775	541	860	649	874										0344
Cj_OXC6603	0480	0021	19	775	874	5	775	541	860	649	874										0480
Cj_OXC7168	0857	0021	19	775	874	5	775	541	860	649	874										0857
Cj_OXC5372	0883	0021	19	775	874	5	775	541	860	649	874										0883
Cj_OXC5463	0940	0021	19	775	465	7	775	541	860	649	874	107	465								0940
Cj_OXC4864	1168	0021	19	775	874	5	775	541	860	649	874										1168
Cj_OXC7068	1241	0021	19	775	396	7	775	860	649	874	597	558	396								1241
Cj_OXC7329	1345	0021	19	775	396	7	775	860	649	874	597	558	396								1345
Cj_OXC5840	1631	0021	19	775	396	7	775	860	649	874	597	558	396								1631
Cj_OXC7420	1785	0021	19	775	784	6	775	541	860	649	874	784									1785
Cj_OXC5230	2360	0021	19	775	403	6	775	541	649	874	49	403									2360
Cj_OXC8269	3049	0021	19	775	465	7	775	541	860	649	874	107	465								3049
Cj_OXC8270	3145	0021	19	775	874	5	775	541	860	649	874										3145
Cj_OXC8666	3493	0021	19	775	651	6	775	541	860	649	874	651									3493
Cj_OXC8837	3549	0021	19	775	558	6	775	860	649	874	597	558									3549
Cj_OXC8836	3631	0021	19	775	49	6	775	541	860	649	874	49									3631
Cj_NS_35_R	2027	0021	19	860	50	4	860	649	874	50											2027
Cj_OXC8563	3410	0021	19	860	874	3	860	649	874												3410
Cj_CAMSA15_R	2010	0021	21	10	338	5	10	24	365	861	338										2010
Cj_CAMSA25	1142	0021	21	148	235	5	148	583	411	101	235										1142
Cj_CAMSA4054	1537	0021	21	148	47	6	148	583	411	101	235	47									1537
Cj_OXC6642	0515	0021	21	172	289	2	172	289													0515
Cj_OXC7190	1377	0021	21	218	487	7	218	274	455	487	261	781	487								1377
Cj_OXC5826	1624	0021	21	451	61	3	451	520	61												1624
Cj_OXC6335	0295	0021	21	487	597	6	487	775	860	649	874	597									0295
Cj_OXC7140	0831	0021	21	487	261	5	487	773	923	836	261										0831
Cj_OXC7153	0844	0021	21	487	210	3	487	773	210												0844
Cj_OXC5344	0874	0021	21	487	210	3	487	773	210												0874
Cj_OXC6872	1068	0021	21	487	24	8	487	775	860	649	597	618	2	24							1068
Cj_CAMSA2056	1484	0021	21	487	210	3	487	773	210												1484
Cj_CAMSA2175	1496	0021	21	487	210	3	487	773	210												1496
Cj_CAMSA3666	1507	0021	21	487	210	3	487	773	210												1507
Cj_CAMSA3741	1517	0021	21	487	597	6	487	775	860	649	874	597									1517
Cj_CAMSA4076	1539	0021	21	487	210	3	487	773	210												1539
Cj_CAMSA3741_rr	2592	0021	21	487	597	6	487	775	860	649	874	597									2592
Cj_OXC8933	3715	0021	21	487	261	5	487	773	923	836	261										3715
Cj_OXC6275	0242	0021	21	625	735	11	625	218	274	455	261	781	597	235	55	866	735				0242
Cj_OXC6483	0411	0021	21	625	735	11	625	218	274	33	261	781	597	235	55	866	735				0411
Cj_OXC6602	0479	0021	21	625	487	8	625	218	274	455	487	261	781	487							0479
Cj_OXC6812	0670	0021	21	625	735	11	625	218	274	33	261	781	597	235	55	866	735				0670
Cj_OXC7137	0828	0021	21	625	487	8	625	218	274	455	487	261	781	487							0828
Cj_OXC6850	1047	0021	21	625	735	11	625	218	274	33	261	781	597	235	55	866	735				1047
Cj_OXC6886	1081	0021	21	625	735	11	625	218	274	33	261	781	597	235	55	866	735				1081
Cj_CAMSA55	1160	0021	21	625	487	7	625	274	455	487	261	781	487								1160
Cj_OXC7335	1351	0021	21	625	735	7	625	218	274	455	55	866	735								1351
Cj_OXC7195	1382	0021	21	625	735	11	625	218	274	33	261	781	597	235	55	866	735				1382
Cj_OXC7223	1406	0021	21	625	735	11	625	218	274	33	261	781	597	235	55	866	735				1406
Cj_CAMSA163	1429	0021	21	625	735	11	625	218	274	455	261	781	597	235	55	866	735				1429
Cj_CAMSA257	1435	0021	21	625	735	10	625	218	274	455	261	781	597	235	866	735					1435
Cj_OXC5652	1588	0021	21	625	735	11	625	218	274	33	261	781	597	235	55	866	735				1588
Cj_OXC5822	1621	0021	21	625	487	8	625	218	274	455	487	261	781	487							1621
Cj_OXC7387	1760	0021	21	625	487	8	625	218	274	455	487	261	781	487							1760
Cj_OXC7397	1768	0021	21	625	735	11	625	218	274	455	261	781	597	235	55	866	735				1768
Cj_OXC7412	1778	0021	21	625	735	11	625	218	274	33	261	781	597	235	55	866	735				1778
Cj_OXC7423	1788	0021	21	625	735	11	625	218	274	33	261	781	597	235	55	866	735				1788
Cj_OXC4633	1901	0021	21	625	735	12	625	218	274	33	261	781	597	235	235	55	866	735			1901
Cj_CJ6850_R	2035	0021	21	625	735	11	625	218	274	33	261	781	597	235	55	866	735				2035
Cj_OXC7927	2522	0021	21	625	735	11	625	218	274	33	261	781	597	235	55	866	735				2522
Cj_OXC8115	2661	0021	21	625	735	11	625	218	274	455	261	781	597	235	55	866	735				2661
Cj_OXC8162	2668	0021	21	625	735	11	625	218	274	33	261	781	597	235	55	866	735				2668
Cj_OXC8163	2759	0021	21	625	735	11	625	218	274	33	261	781	597	235	55	866	735				2759
Cj_OXC7691	2828	0021	21	625	735	11	625	218	274	33	261	781	597	235	55	866	735				2828
Cj_OXC8245	3144	0021	21	625	735	11	625	218	274	33	261	781	597	235	55	866	735				3144
Cj_OXC7795	3406	0021	21	625	735	11	625	218	274	455	261	781	597	235	55	866	735				3406
Cj_OXC8713	3465	0021	21	625	488	8	625	218	274	455	488	261	781	488							3465
Cj_OXC8714	3466	0021	21	625	488	8	625	218	274	455	488	261	781	488							3466
Cj_OXC8704	3457	0021	21	649	597	3	649	874	597												3457
Cj_OXC8718	3497	0021	21	649	597	3	649	874	597												3497
Cj_04199	0006	0021	21	743	542	5	743	938	487	233	542										0006
Cj_6399	0046	0021	21	743	158	4	743	938	487	158											0046
Cj_DFVF1099	0063	0021	21	743	487	3	743	938	487												0063
Cj_xy259	0109	0021	21	743	487	3	743	938	487												0109
Cj_H135060411	0144	0021	21	743	677	6	743	938	487	284	57	677									0144
Cj_H135060412	0145	0021	21	743	677	6	743	938	487	284	57	677									0145
Cj_OXC6562	0192	0021	21	743	261	6	743	487	773	923	836	261									0192

Cj_OXC6563	0193	0021	21	743	283	4	743	487	261	283							0193
Cj_OXC6573	0202	0021	21	743	283	4	743	487	261	283							0202
Cj_OXC6251	0223	0021	21	743	107	10	743	487	775	860	649	874	597	893	735	107	0223
Cj_OXC6257	0227	0021	21	743	541	7	743	487	775	860	874	597	541				0227
Cj_OXC6270	0237	0021	21	743	487	3	743	938	487								0237
Cj_OXC6285	0250	0021	21	743	107	10	743	487	775	860	649	874	597	893	735	107	0250
Cj_OXC6310	0271	0021	21	743	107	10	743	487	775	860	649	874	597	893	735	107	0271
Cj_OXC6317	0277	0021	21	743	542	5	743	938	487	233	542						0277
Cj_OXC6329	0289	0021	21	743	541	7	743	487	775	860	874	597	541				0289
Cj_OXC6334	0294	0021	21	743	597	7	743	487	775	860	649	874	597				0294
Cj_OXC6383	0335	0021	21	743	874	6	743	487	775	860	649	874					0335
Cj_OXC6405	0351	0021	21	743	597	7	743	487	775	860	649	874	597				0351
Cj_OXC6420	0365	0021	21	743	874	6	743	487	775	860	649	874					0365
Cj_OXC6496	0424	0021	21	743	487	3	743	938	487								0424
Cj_OXC6508	0435	0021	21	743	289	5	743	938	487	427	289						0435
Cj_OXC6519	0445	0021	21	743	487	3	743	938	487								0445
Cj_OXC6539	0463	0021	21	743	597	7	743	487	775	860	649	874	597				0463
Cj_OXC6255	0468	0021	21	743	541	7	743	487	775	860	874	597	541				0468
Cj_CAMSA8	0472	0021	21	743	474	4	743	938	487	474							0472
Cj_OXC6596	0475	0021	21	743	487	3	743	938	487								0475
Cj_OXC6619	0493	0021	21	743	110	8	743	487	775	860	874	597	541	110			0493
Cj_OXC6625	0499	0021	21	743	283	4	743	487	261	283							0499
Cj_OXC6627	0501	0021	21	743	283	4	743	487	261	283							0501
Cj_OXC6629	0503	0021	21	743	283	4	743	487	261	283							0503
Cj_OXC6637	0510	0021	21	743	874	6	743	487	775	860	649	874					0510
Cj_OXC6671	0542	0021	21	743	677	6	743	938	487	284	57	677					0542
Cj_OXC6678	0549	0021	21	743	541	7	743	487	775	860	874	597	541				0549
Cj_OXC6681	0552	0021	21	743	283	4	743	487	261	283							0552
Cj_OXC6690	0559	0021	21	743	541	7	743	487	775	860	874	597	541				0559
Cj_OXC6711	0578	0021	21	743	487	3	743	938	487								0578
Cj_OXC6728	0594	0021	21	743	597	7	743	487	775	860	649	874	597				0594
Cj_OXC6730	0596	0021	21	743	111	4	743	938	487	111							0596
Cj_OXC6731	0597	0021	21	743	677	6	743	938	487	284	57	677					0597
Cj_OXC6745	0608	0021	21	743	487	3	743	938	487								0608
Cj_OXC6764	0625	0021	21	743	597	7	743	487	775	860	649	874	597				0625
Cj_OXC6791	0650	0021	21	743	874	6	743	487	775	860	649	874					0650
Cj_OXC6804	0663	0021	21	743	487	3	743	938	487								0663
Cj_CAMSA15	0726	0021	21	743	474	4	743	938	487	474							0726
Cj_OXC6945	0740	0021	21	743	874	6	743	487	775	860	649	874					0740
Cj_OXC6948	0743	0021	21	743	597	7	743	487	775	860	649	874	597				0743
Cj_OXC6961	0756	0021	21	743	541	7	743	487	775	860	874	597	541				0756
Cj_OXC6979	0772	0021	21	743	500	9	743	487	775	860	874	597	541	107	500		0772
Cj_OXC7000	0788	0021	21	743	874	6	743	487	775	860	649	874					0788
Cj_OXC7136	0827	0021	21	743	597	6	743	775	860	649	874	597					0827
Cj_OXC7142	0833	0021	21	743	487	3	743	938	487								0833
Cj_OXC7147	0838	0021	21	743	100	6	743	487	775	860	597	100					0838
Cj_OXC5350	0877	0021	21	743	541	7	743	487	775	860	874	597	541				0877
Cj_OXC5364	0880	0021	21	743	597	6	743	487	775	860	649	597					0880
Cj_OXC5413	0909	0021	21	743	261	6	743	487	773	923	836	261					0909
Cj_OXC5465	0942	0021	21	743	576	9	743	487	775	860	649	597	539	425	576		0942
Cj_OXC5474	0946	0021	21	743	541	7	743	487	775	860	874	597	541				0946
Cj_OXC5713	0985	0021	21	743	235	8	743	487	775	860	649	874	597	235			0985
Cj_OXC5739	1000	0021	21	743	487	3	743	938	487								1000
Cj_OXC5754	1011	0021	21	743	261	6	743	487	773	923	836	261					1011
Cj_OXC5757	1012	0021	21	743	261	6	743	487	773	923	836	261					1012
Cj_OXC6867	1063	0021	21	743	111	4	743	938	487	111							1063
Cj_OXC6892	1086	0021	21	743	487	3	743	938	487								1086
Cj_OXC6909	1102	0021	21	743	597	5	743	860	649	874	597						1102
Cj_CAMSA26	1143	0021	21	743	597	5	743	487	860	874	597						1143
Cj_OXC4811	1144	0021	21	743	57	5	743	938	487	284	57						1144
Cj_CAMSA40	1148	0021	21	743	597	7	743	487	775	860	649	874	597				1148
Cj_CAMSA48	1159	0021	21	743	597	7	743	487	775	860	649	874	597				1159
Cj_CAMSA59	1171	0021	21	743	487	3	743	938	487								1171
Cj_OXC4882	1179	0021	21	743	677	6	743	938	487	284	57	677					1179
Cj_OXC7037	1214	0021	21	743	597	7	743	487	775	860	649	874	597				1214
Cj_OXC7058	1232	0021	21	743	541	7	743	487	775	860	874	597	541				1232
Cj_OXC7073	1245	0021	21	743	735	10	743	743	487	775	860	649	874	597	893	735	1245
Cj_OXC7330	1346	0021	21	743	874	6	743	487	775	860	649	874					1346
Cj_OXC7187	1374	0021	21	743	541	7	743	487	775	860	874	597	541				1374
Cj_OXC7212	1397	0021	21	743	735	10	743	743	487	775	860	649	874	597	893	735	1397
Cj_CAMSA75	1421	0021	21	743	487	3	743	938	487								1421
Cj_CAMSA81	1422	0021	21	743	487	3	743	938	487								1422
Cj_CAMSA95	1423	0021	21	743	487	3	743	938	487								1423
Cj_CAMSA100	1425	0021	21	743	597	7	743	487	775	860	649	874	597				1425
Cj_CAMSA141	1426	0021	21	743	474	4	743	938	487	474							1426
Cj_CAMSA153	1428	0021	21	743	474	4	743	938	487	474							1428
Cj_CAMSA182	1430	0021	21	743	597	7	743	487	775	860	649	874	597				1430
Cj_CAMSA244	1433	0021	21	743	677	6	743	938	487	284	57	677					1433
Cj_CAMSA259	1436	0021	21	743	677	6	743	938	487	284	57	677					1436
Cj_CAMSA272	1438	0021	21	743	487	3	743	938	487								1438
Cj_CAMSA275	1439	0021	21	743	487	3	743	938	487								1439
Cj_CAMSA289	1440	0021	21	743	677	4	743	938	487	677							1440
Cj_CAMSA293	1441	0021	21	743	487	3	743	938	487								1441
Cj_CAMSA301	1442	0021	21	743	597	10	743	487	775	860	649	874	597	649	874	597	1442
Cj_CAMSA322	1444	0021	21	743	677	4	743	938	487	677							1444
Cj_CAMSA341	1445	0021	21	743	261	4	743	923	836	261							1445
Cj_CAMSA343	1446	0021	21	743	487	3	743	938	487								1446
Cj_CAMSA354	1448	0021	21	743	487	3	743	938	487								1448
Cj_CAMSA357	1449	0021	21	743	597	7	743	487	775	860	649	874	597				1449
Cj_CAMSA361	1450	0021	21	743	487	3	743	938	487								1450
Cj_CAMSA375	1453	0021	21	743	860	4	743	487	775	860							1453
Cj_CAMSA379	1454	0021	21	743	487	3	743	938	487								1454
Cj_CAMSA416	1456	0021	21	743	487	3	743	938	487								1456
Cj_CAMSA418	1457	0021	21	743	487	3	743	938	487								1457
Cj_CAMSA421	1458	0021	21	743	597	3	743	487	597								1458
Cj_CAMSA454	1459	0021	21	743	487	3	743	938	487								1459
Cj_CAMSA457	1460	0021	21	743	487	3	743	938	487								1460
Cj_CAMSA1000	1464	0021	21	743	487	3	743	938	487								1464
Cj_CAMSA1004	1465	0021	21	743	425	5	743	938	487	538	425						1465
Cj_CAMSA1006	1466	0021	21	743	677	5	743	938	487	284	677						1466
Cj_CAMSA1020	1468	0021	21	743	597	7	743	487	775	860	649	874	597				1468
Cj_CAMSA1028	1470	0021	21	743	474	4	743	938	487	474							1470
Cj_CAMSA1044	1471	0021	21	743	474	4	743	938	487	474							1471
Cj_CAMSA1052	1472	0021	21	743	487	3	743	938	487								1472
Cj_CAMSA1073	1474	0021	21	743	677	6	743	938	487	284	57	677					1474
Cj_CAMSA1080	1475	0021	21	743	474	4	743	938	487	474							1475
Cj_CAMSA2002	1476	0021	21	743	597	7	743	487	775	860	649	874	597				1476
Cj_CAMSA2005	1477	0021	21	743	487	3	743	938	487								1477
Cj_CAMSA2024	1479	0021	21	743	677	6	743	938	487	284	57	677					1479
Cj_CAMSA2036	1481	0021	21	743	487	3	743	938	487								1481
Cj_CAMSA2038	1482	0021	21	743	474	4	743	938	487	474							1482
Cj_CAMSA2057	1485	0021	21	743	677	6	743	938	487	284	57	677					1485
Cj_CAMSA2086	1487	0021	21	743	597	10	743	487	775	860	649	874	597	649	874	597	1487
Cj_CAMSA2102	1489	0021	21	743	837	4	743	938	487	837							1489
Cj_CAMSA2113	1491	0021	21	743	474	4	743	938	487	474							1491
Cj_CAMSA2115	1492	0021	21	743	487	3	743	938	487								1492
Cj_CAMSA2147	1493	0021	21	743	81	3	743	938	81								1493
Cj_CAMSA2164	1495	0021	21	743	474	4	743	938	487	474							1495
Cj_CAMSA2200	1498	0021	21	743	487	3	743	938	487								1498
Cj_CAMSA3632	1502	0021	21	743	474	4	743	938	487	474							1502
Cj_CAMSA3643	1504	0021	21	743	597	5	743	860	649	874	597						1504
Cj_CAMSA3665	1506	0021	21	743	597	6	743	487	775	860	874	597					1506

Cj_CAMSA3669	1508	0021	21	743	487	3	743	938	487								1508
Cj_CAMSA3671	1509	0021	21	743	597	7	743	487	775	860	649	874	597				1509
Cj_CAMSA3681	1511	0021	21	743	597	6	743	487	775	860	874	597					1511
Cj_CAMSA3683	1512	0021	21	743	597	6	743	487	775	649	874	597					1512
Cj_CAMSA3701	1514	0021	21	743	597	7	743	487	775	860	649	874	597				1514
Cj_CAMSA3731	1516	0021	21	743	487	3	743	938	487								1516
Cj_CAMSA3786	1518	0021	21	743	487	3	743	938	487								1518
Cj_CAMSA3797	1519	0021	21	743	487	3	743	938	487								1519
Cj_CAMSA3801	1520	0021	21	743	487	3	743	938	487								1520
Cj_CAMSA3802	1521	0021	21	743	597	6	743	487	775	860	874	597					1521
Cj_CAMSA3837	1522	0021	21	743	487	3	743	938	487								1522
Cj_CAMSA3844	1523	0021	21	743	677	5	743	938	487	284	677						1523
Cj_CAMSA3845	1524	0021	21	743	487	3	743	938	487								1524
Cj_CAMSA3874	1526	0021	21	743	474	4	743	938	487	474							1526
Cj_CAMSA3888	1527	0021	21	743	474	4	743	938	487	474							1527
Cj_CAMSA3897	1529	0021	21	743	597	7	743	487	775	860	649	874	597				1529
Cj_CAMSA3914	1530	0021	21	743	597	7	743	487	775	860	649	874	597				1530
Cj_CAMSA3928	1532	0021	21	743	487	3	743	938	487								1532
Cj_CAMSA3943	1533	0021	21	743	487	3	743	938	487								1533
Cj_CAMSA4001	1535	0021	21	743	487	3	743	938	487								1535
Cj_CAMSA4031	1536	0021	21	743	597	7	743	487	775	860	649	874	597				1536
Cj_CAMSA4124	1540	0021	21	743	677	5	743	938	487	284	677						1540
Cj_CAMSA4133	1541	0021	21	743	487	3	743	938	487								1541
Cj_CAMSA4140	1542	0021	21	743	575	9	743	487	775	860	649	874	597	274	575		1542
Cj_CAMSA4212	1546	0021	21	743	487	3	743	938	487								1546
Cj_CAMSA4221	1547	0021	21	743	487	3	743	938	487								1547
Cj_CAMSA4243	1548	0021	21	743	597	7	743	487	775	860	649	874	597				1548
Cj_OXC6605	1549	0021	21	743	283	4	743	487	261	283							1549
Cj_OXC5495	1553	0021	21	743	541	7	743	487	775	860	874	597	541				1553
Cj_OXC5498	1555	0021	21	743	541	7	743	487	775	860	874	597	541				1555
Cj_OXC5618	1563	0021	21	743	534	6	743	487	775	860	597	534					1563
Cj_OXC5625	1567	0021	21	743	534	6	743	487	775	860	597	534					1567
Cj_OXC5649	1586	0021	21	743	597	7	743	487	775	860	649	874	597				1586
Cj_OXC5791	1595	0021	21	743	541	7	743	487	775	860	874	597	541				1595
Cj_OXC5835	1628	0021	21	743	735	9	743	487	775	860	649	874	597	893	735		1628
Cj_OXC5846	1635	0021	21	743	534	6	743	487	775	860	597	534					1635
Cj_OXC5848	1637	0021	21	743	161	4	743	938	487	161							1637
Cj_OXC5938	1694	0021	21	743	735	9	743	487	775	860	649	874	597	893	735		1694
Cj_OXC5832	1701	0021	21	743	735	9	743	487	775	860	649	874	597	893	735		1701
Cj_OXC5862	1707	0021	21	743	541	7	743	487	775	860	874	597	541				1707
Cj_OXC5924	1716	0021	21	743	735	9	743	487	775	860	649	874	597	893	735		1716
Cj_OXC5929	1717	0021	21	743	107	10	743	487	775	860	649	874	597	893	735	107	1717
Cj_OXC5937	1718	0021	21	743	541	7	743	487	775	860	874	597	541				1718
Cj_OXC7351	1728	0021	21	743	597	7	743	487	775	860	649	874	597				1728
Cj_OXC7366	1741	0021	21	743	541	7	743	487	775	860	874	597	541				1741
Cj_OXC7373	1747	0021	21	743	597	7	743	487	775	860	649	874	597				1747
Cj_OXC7405	1773	0021	21	743	597	6	743	487	775	649	874	597					1773
Cj_OXC7415	1780	0021	21	743	107	10	743	487	775	860	649	874	597	893	735	107	1780
Cj_OXC7419	1784	0021	21	743	289	5	743	938	487	427	289						1784
Cj_OXC7479	1831	0021	21	743	487	3	743	938	487								1831
Cj_OXC7489	1840	0021	21	743	173	6	743	938	487	522	548	173					1840
Cj_OXC7508	1855	0021	21	743	261	6	743	487	773	923	836	261					1855
Cj_OXC4581	1874	0021	21	743	597	7	743	487	775	860	649	874	597				1874
Cj_OXC4649	1911	0021	21	743	283	4	743	487	261	283							1911
Cj_OXC4663	1919	0021	21	743	597	5	743	487	775	874	597						1919
Cj_OXC4673	1926	0021	21	743	735	9	743	487	775	860	649	874	597	893	735		1926
Cj_HM235411	1973	0021	21	743	338	9	743	938	487	284	57	677	728	759	338		1973
Cj_HM904280	1976	0021	21	743	338	9	743	938	487	284	57	677	728	759	338		1976
Cj_HM909204	1977	0021	21	743	338	9	743	938	487	284	57	677	728	759	338		1977
Cj_HM908193	1979	0021	21	743	338	9	743	938	487	284	57	677	728	759	338		1979
Cj_HM905080	1980	0021	21	743	338	9	743	938	487	284	57	677	728	759	338		1980
Cj_HM909114	1981	0021	21	743	338	9	743	938	487	284	57	677	728	759	338		1981
Cj_HM314036	1982	0021	21	743	338	9	743	938	487	284	57	677	728	759	338		1982
Cj_HM331258	1984	0021	21	743	338	9	743	938	487	284	57	677	728	759	338		1984
Cj_HM905487	1985	0021	21	743	338	9	743	938	487	284	57	677	728	759	338		1985
Cj_HM79979	1986	0021	21	743	338	9	743	938	487	284	57	677	728	759	338		1986
Cj_HM907529	1987	0021	21	743	338	9	743	938	487	284	57	677	728	759	338		1987
Cj_HM326144	1988	0021	21	743	338	9	743	938	487	284	57	677	728	759	338		1988
Cj_HM912030	1989	0021	21	743	338	9	743	938	487	284	57	677	728	759	338		1989
Cj_HM905980	1990	0021	21	743	338	9	743	938	487	284	57	677	728	759	338		1990
Cj_HM0627643	1991	0021	21	743	338	9	743	938	487	284	57	677	728	759	338		1991
Cj_HM179091	1992	0021	21	743	338	9	743	938	487	284	57	677	728	759	338		1992
Cj_HM908538	1993	0021	21	743	338	9	743	938	487	284	57	677	728	759	338		1993
Cj_HM179023	1994	0021	21	743	338	9	743	938	487	284	57	677	728	759	338		1994
Cj_HM908265	1995	0021	21	743	338	9	743	938	487	284	57	677	728	759	338		1995
Cj_CAMSA471_R	2008	0021	21	743	487	3	743	938	487								2008
Cj_ARI_1331_R	2025	0021	21	743	735	9	743	487	775	860	649	874	597	893	735		2025
Cj_ARI_814_R	2033	0021	21	743	677	7	743	938	487	284	57	677	677				2033
Cj_CAMSA3914_R	2036	0021	21	743	597	7	743	487	775	860	649	874	597				2036
Cj_CAMSA3916_R	2037	0021	21	743	487	3	743	938	487								2037
Cj_OXC7608	2113	0021	21	743	874	6	743	487	775	860	649	874					2113
Cj_OXC7624	2122	0021	21	743	487	3	743	938	487								2122
Cj_OXC7764	2140	0021	21	743	677	6	743	938	487	284	57	677					2140
Cj_OXC5063	2266	0021	21	743	541	7	743	487	775	860	874	597	541				2266
Cj_OXC5082	2273	0021	21	743	597	7	743	487	775	860	649	874	597				2273
Cj_OXC5112	2287	0021	21	743	677	6	743	938	487	284	57	677					2287
Cj_OXC5147	2308	0021	21	743	735	9	743	487	775	860	649	874	597	893	735		2308
Cj_OXC5149	2309	0021	21	743	597	7	743	487	775	860	649	874	597				2309
Cj_OXC5166	2313	0021	21	743	261	6	743	487	773	923	836	261					2313
Cj_OXC5184	2326	0021	21	743	742	9	743	487	775	860	874	597	541	111	742		2326
Cj_OXC5185	2327	0021	21	743	677	6	743	938	487	284	57	677					2327
Cj_OXC5189	2330	0021	21	743	597	7	743	487	775	860	649	874	597				2330
Cj_OXC5200	2338	0021	21	743	874	6	743	487	775	860	649	874					2338
Cj_OXC5203	2339	0021	21	743	597	7	743	487	775	860	649	874	597				2339
Cj_OXC5220	2351	0021	21	743	24	10	743	487	775	860	649	874	597	618	2	24	2351
Cj_OXC5239	2366	0021	21	743	597	7	743	487	775	860	649	874	597				2366
Cj_OXC5298	2386	0021	21	743	597	7	743	487	775	860	649	874	597				2386
Cj_OXC7667	2410	0021	21	743	181	9	743	487	775	860	874	597	541	235	181		2410
Cj_OXC7672	2414	0021	21	743	597	6	743	487	775	860	649	597					2414
Cj_OXC7686	2425	0021	21	743	677	5	743	938	284	57	677						2425
Cj_OXC7700	2434	0021	21	743	487	3	743	938	487								2434
Cj_OXC7905	2507	0021	21	743	677	6	743	938	487	284	57	677					2507
Cj_OXC7915	2513	0021	21	743	511	4	743	938	487	511							2513
Cj_OXC7919	2517	0021	21	743	541	7	743	487	775	860	874	597	541				2517
Cj_OXC7924	2520	0021	21	743	534	8	743	487	775	860	649	874	597	534			2520
Cj_OXC7602	2566	0021	21	743	677	6	743	938	487	284	57	677					2566
Cj_OXC7610	2567	0021	21	743	283	4	743	487	261	283							2567
Cj_OXC7758	2568	0021	21	743	597	6	743	487	775	860	874	597					2568
Cj_CAMSA3632_rr	2589	0021	21	743	474	4	743	938	487	474							2589
Cj_CAMSA3665_rr	2590	0021	21	743	597	6	743	487	775	860	874	597					2590
Cj_CAMSA3681_rr	2591	0021	21	743	597	6	743	487	775	860	874	597					2591
Cj_CAMSA3802_rr	2593	0021	21	743	597	6	743	487	775	860	874	597					2593
Cj_CAMSA3845_rr	2596	0021	21	743	487	3	743	938	487								2596
Cj_CAMSA3914_rr	2597	0021	21	743	597	7	743	487	775	860	649	874	597				2597
Cj_CAMSA4031_rr	2600	0021	21	743	597	7	743	487	775	860	649	874	597				2600
Cj_CAMSA4124_rr	2601	0021	21	743	677	5	743	938	487	284	677						2601
Cj_OXC7957	2610	0021	21	743	597	7	743	487	775	860	649	874	597				2610
Cj_OXC7969	2618	0021	21	743	597	7	743	487	775	860	649	874	597				2618
Cj_OXC7976	2625	0021	21	743	24	9	743	487	775	860	649	874	597	801	24		2625
Cj_OXC8116	2662	0021	21	743	742	4	743	938	487	742							2662
Cj_OXC8145	2665	0021	21	743	597	6	743	487	775	860	874	597					2665
Cj_OXC7990	2707	0021	21	743	24	9	743	487	775	860	649	874	597	801	24		2707

Cj_OXC8019	2719	0021	21	743	24	6	743	487	860	649	874	24						2719
Cj_OXC8025	2720	0021	21	743	677	4	743	284	57	677								2720
Cj_OXC8077	2738	0021	21	743	541	7	743	487	775	860	874	597	541					2738
Cj_OXC7651	2769	0021	21	743	57	5	743	938	487	284	57							2769
Cj_OXC5220_R	2774	0021	21	743	24	10	743	487	775	860	649	874	597	618	2	24		2774
Cj_OXC8111	2820	0021	21	743	742	4	743	938	487	742								2820
Cj_OXC7434	2823	0021	21	743	597	6	743	487	775	860	649	597						2823
Cj_OXC7460	2825	0021	21	743	597	6	743	487	775	860	649	597						2825
Cj_OXC7688	2827	0021	21	743	57	5	743	938	487	284	57							2827
Cj_OXC7729	2830	0021	21	743	874	6	743	487	775	860	649	874						2830
Cj_OXC4624	2847	0021	21	743	541	7	743	487	775	860	874	597	541					2847
Cj_Dg14b	2866	0021	21	743	597	6	743	487	775	860	874	597						2866
Cj_Dg7a	2877	0021	21	743	541	6	743	487	860	874	597	541						2877
Cj_Dg16b	2883	0021	21	743	597	6	743	487	775	860	874	597						2883
Cj_Dg361	2900	0021	21	743	541	7	743	487	775	860	874	597	541					2900
Cj_Dg194	2957	0021	21	743	541	7	743	487	775	860	874	597	541					2957
Cj_OXC8186	2988	0021	21	743	338	11	743	938	487	284	57	677	728	810	801	796	338	2988
Cj_OXC8200	3001	0021	21	743	597	7	743	487	775	860	649	874	597					3001
Cj_OXC8202	3003	0021	21	743	597	7	743	487	775	860	649	874	597					3003
Cj_OXC8233	3026	0021	21	743	874	3	743	487	874									3026
Cj_OXC8262	3044	0021	21	743	487	4	743	938	938	487								3044
Cj_OXC8279	3056	0021	21	743	487	3	743	938	487									3056
Cj_OXC8312	3085	0021	21	743	874	6	743	487	775	860	649	874						3085
Cj_OXC8350	3121	0021	21	743	869	8	743	487	775	860	874	597	541	869				3121
Cj_OXC8384	3160	0021	21	743	597	7	743	487	775	860	649	874	597					3160
Cj_OXC8398	3172	0021	21	743	283	4	743	487	261	283								3172
Cj_OXC8418	3189	0021	21	743	283	4	743	487	261	283								3189
Cj_OXC8419	3190	0021	21	743	541	7	743	487	775	860	874	597	541					3190
Cj_OXC8421	3192	0021	21	743	597	7	743	487	775	860	649	874	597					3192
Cj_OXC8520	3280	0021	21	743	677	6	743	938	487	284	57	677						3280
Cj_OXC8551	3305	0021	21	743	162	7	743	487	775	860	874	597	162					3305
Cj_5329_H135060411-1	3311	0021	21	743	677	6	743	938	487	284	57	677						3311
Cj_5330_H135060412-1	3312	0021	21	743	677	6	743	938	487	284	57	677						3312
Cj_5331_H135080149-1	3313	0021	21	743	677	6	743	938	487	284	57	677						3313
Cj_5332_H135080150-1	3314	0021	21	743	677	6	743	938	487	284	57	677						3314
Cj_5333_H135100351-1	3315	0021	21	743	677	6	743	938	487	284	57	677						3315
Cj_5334_H135100352-1	3316	0021	21	743	677	6	743	938	487	284	57	677						3316
Cj_5335_H135100353-1	3317	0021	21	743	677	6	743	938	487	284	57	677						3317
Cj_5336_H135140732-1	3318	0021	21	743	677	6	743	938	487	284	57	677						3318
Cj_5338_H135200111-1	3319	0021	21	743	677	6	743	938	487	284	57	677						3319
Cj_OXC8569	3347	0021	21	743	107	10	743	487	775	860	649	874	597	893	735	107		3347
Cj_OXC8673	3409	0021	21	743	541	7	743	487	775	860	874	597	541					3409
Cj_OXC8571	3411	0021	21	743	162	7	743	487	775	860	874	597	162					3411
Cj_OXC8591	3413	0021	21	743	702	9	743	487	775	860	874	597	541	235	702			3413
Cj_OXC8678	3440	0021	21	743	735	9	743	487	775	860	649	874	597	893	735			3440
Cj_OXC8715	3467	0021	21	743	501	10	743	487	860	649	874	874	597	735	735	501		3467
Cj_OXC8734	3478	0021	21	743	501	10	743	487	860	649	874	874	597	735	735	501		3478
Cj_OXC8621	3490	0021	21	743	735	9	743	487	860	649	874	874	597	893	735			3490
Cj_OXC8625R	3498	0021	21	743	261	6	743	487	773	923	836	261						3498
Cj_OXC8623	3501	0021	21	743	736	8	743	487	775	860	874	597	225	736				3501
Cj_OXC8762	3570	0021	21	743	874	6	743	487	775	860	649	874						3570
Cj_OXC8778	3584	0021	21	743	597	7	743	487	775	860	649	874	597					3584
Cj_OXC8779	3585	0021	21	743	541	7	743	487	775	860	874	597	541					3585
Cj_OXC8850	3642	0021	21	743	541	7	743	487	775	860	874	597	541					3642
Cj_OXC8939	3721	0021	21	743	261	5	743	487	923	836	261							3721
Cj_OXC8969	3739	0021	21	743	735	9	743	487	775	860	649	874	597	893	735			3739
Cj_OXC6845	1043	0021	21	860	534	3	860	597	534									1043
Cj_CAMSA3634	1503	0021	21	860	597	4	860	649	874	597								1503
Cj_OXC7693	2428	0021	21	860	915	5	860	649	874	597	915							2428
Cj_00-2544	0004	0021	21	938	841	4	938	877	43	841								0004
Cj_OXC6689	0558	0021	21	938	2	2	938	2										0558
Cj_OXC6702	0571	0021	21	938	2	2	938	2										0571
Cj_OXC7134	0825	0021	21	938	2	2	938	2										0825
Cj_OXC4863	1167	0021	21	938	2	2	938	2										1167
Cj_OXC7247	1271	0021	21	938	2	2	938	2										1271
Cj_OXC7306	1326	0021	21	938	2	2	938	2										1326
Cj_CAMSA245	1434	0021	21	938	2	2	938	2										1434
Cj_CAMSA311	1443	0021	21	938	839	3	938	487	839									1443
Cj_CAMSA1008	1467	0021	21	938	2	2	938	2										1467
Cj_CAMSA1021	1469	0021	21	938	2	2	938	2										1469
Cj_CAMSA3644	1505	0021	21	938	2	2	938	2										1505
Cj_CAMSA3674	1510	0021	21	938	2	2	938	2										1510
Cj_CAMSA3889	1528	0021	21	938	2	2	938	2										1528
Cj_CAMSA4196	1544	0021	21	938	2	2	938	2										1544
Cj_OXC5819	1618	0021	21	938	2	2	938	2										1618
Cj_OXC7530	1949	0021	21	938	2	2	938	2										1949
Cj_OXC7558	2076	0021	21	938	2	2	938	2										2076
Cj_OXC5102	2282	0021	21	938	2	2	938	2										2282
Cj_OXC7718	2443	0021	21	938	2	2	938	2										2443
Cj_OXC7856	2472	0021	21	938	2	2	938	2										2472
Cj_BID1NL	2581	0021	21	938	2	2	938	2										2581
Cj_CAMSA4196_rr	2603	0021	21	938	2	2	938	2										2603
Cj_OXC7954	2608	0021	21	938	283	5	938	487	427	261	283							2608
Cj_Dg326	2856	0021	21	938	2	2	938	2										2856
Cj_Dg328	2962	0021	21	938	2	2	938	2										2962
Cj_V124000-1	3335	0021	21	938	2	2	938	2										3335
Cj_OXC8816	3613	0021	21	938	2	2	938	2										3613
Cj_OXC8873	3662	0021	21	938	2	2	938	2										3662
Cj_NCTC_11168	0095	0021	43	743	367	4	743	938	487	367								0095
Cj_OXC6667	0538	0021	44	10	876	7	10	30	468	874	342	487	876					0538
Cj_OXC6981	0774	0021	44	10	876	7	10	30	468	874	342	487	876					0774
Cj_OXC7141	0832	0021	44	10	876	7	10	30	468	874	342	487	876					0832
Cj_OXC5470	0944	0021	44	10	876	7	10	30	468	874	342	487	876					0944
Cj_OXC7173	1361	0021	44	10	876	7	10	30	468	874	342	487	876					1361
Cj_OXC7973	2622	0021	44	10	876	7	10	30	468	874	342	487	876					2622
Cj_OXC8326	3099	0021	44	425	464	2	425	464										3099
Cj_OXC7150	0841	0021	44	596	425	2	596	425										0841
Cj_OXC6664	0535	0021	50	9	622	3	9	88	622									0535
Cj_H133640222	0134	0021	50	23	338	3	23	451	338									0134
Cj_H133640223	0135	0021	50	23	338	3	23	451	338									0135
Cj_H133640225	0136	0021	50	23	338	3	23	451	338									0136
Cj_H133640228	0138	0021	50	23	338	3	23	451	338									0138
Cj_OXC6493	0421	0021	50	23	338	3	23	451	338									0421
Cj_OXC6502	0430	0021	50	23	338	3	23	451	338									0430
Cj_OXC6763	0624	0021	50	23	338	3	23	451	338									0624
Cj_OXC6949	0744	0021	50	23	338	3	23	451	338									0744
Cj_OXC5445	0930	0021	50	23	338	3	23	451	338									0930
Cj_OXC6836	1034	0021	50	23	338	3	23	451	338									1034
Cj_OXC7015	1194	0021	50	23	338	3	23	451	338									1194
Cj_OXC7020	1199	0021	50	23	338	3	23	451	338									1199
Cj_OXC7293	1314	0021	50	23	338	3	23	451	338									1314
Cj_OXC7302	1322	0021	50	23	338	3	23	451	338									1322
Cj_CJ7015_R	2038	0021	50	23	338	3	23	451	338									2038
Cj_OXC7613	2115	0021	50	23	338	3	23	451	338									2115
Cj_OXC7637	2133	0021	50	23	338	3	23	451	338									2133
Cj_OXC7836	2193	0021	50	23	338	3	23	451	338									2193
Cj_OXC7837	2194	0021	50	23	338	3	23	451	338									2194
Cj_OXC7854	2470	0021	50	23	338	3	23	451	338									2470
Cj_OXC8138	2691	0021	50	23	338	3	23	451	338									2691
Cj_OXC8015	2796	0021	50	23	338	3	23	451	338									2796
Cj_OXC8227	3020	0021	50	23	338	3	23	451	338									3020
Cj_OXC8231	3024	0021	50	23	338	3	23	451	338									3024

Cj_OXC8291	3066	0021	50	23	338	3	23	451	338						3066
Cj_OXC8296	3069	0021	50	23	338	3	23	451	338						3069
Cj_OXC8455	3219	0021	50	23	338	3	23	451	338						3219
Cj_OXC8456	3220	0021	50	23	338	3	23	451	338						3220
Cj_OXC8457	3221	0021	50	23	338	3	23	451	338						3221
Cj_OXC8462	3226	0021	50	23	338	3	23	451	338						3226
Cj_OXC8472	3236	0021	50	23	338	3	23	451	338						3236
Cj_OXC8478	3242	0021	50	23	338	3	23	451	338						3242
Cj_OXC8539	3297	0021	50	23	338	3	23	451	338						3297
Cj_OXC8699	3339	0021	50	23	338	3	23	451	338						3339
Cj_OXC8564	3345	0021	50	23	338	3	23	451	338						3345
Cj_OXC8614	3380	0021	50	23	338	3	23	451	338						3380
Cj_OXC8620	3422	0021	50	23	338	3	23	451	338						3422
Cj_OXC8727	3473	0021	50	23	338	3	23	451	338						3473
Cj_OXC8405R	3485	0021	50	23	338	3	23	451	338						3485
Cj_OXC9004	3547	0021	50	23	338	3	23	451	338						3547
Cj_OXC8831	3626	0021	50	23	338	3	23	451	338						3626
Cj_OXC8854	3645	0021	50	23	338	3	23	451	338						3645
Cj_OXC7349	1726	0021	50	309	865	6	309	528	133	609	407	865			1726
Cj_PT14	0101	0021	50	323	641	3	323	712	641						0101
Cj_H121820083	0125	0021	50	323	19	4	323	712	641	19					0125
Cj_H140940807	0147	0021	50	323	222	6	323	712	641	19	108	222			0147
Cj_OXC6571	0200	0021	50	323	19	4	323	712	641	19					0200
Cj_OXC6266	0234	0021	50	323	19	4	323	712	641	19					0234
Cj_OXC6286	0251	0021	50	323	19	4	323	712	641	19					0251
Cj_OXC6292	0257	0021	50	323	19	4	323	712	641	19					0257
Cj_OXC6457	0390	0021	50	323	641	3	323	712	641						0390
Cj_OXC6531	0456	0021	50	323	19	4	323	712	641	19					0456
Cj_OXC6615	0490	0021	50	323	573	8	323	712	641	19	108	222	857	573	0490
Cj_OXC5335	0867	0021	50	323	19	4	323	712	641	19					0867
Cj_OXC5349	0876	0021	50	323	19	4	323	712	641	19					0876
Cj_OXC5435	0923	0021	50	323	19	4	323	712	641	19					0923
Cj_OXC5462	0939	0021	50	323	222	5	323	712	641	108	222				0939
Cj_OXC5664	0954	0021	50	323	222	6	323	712	641	19	108	222			0954
Cj_OXC5691	0970	0021	50	323	641	3	323	712	641						0970
Cj_OXC5720	0988	0021	50	323	641	3	323	712	641						0988
Cj_OXC5766	1019	0021	50	323	19	4	323	712	641	19					1019
Cj_OXC5771	1022	0021	50	323	930	5	323	712	641	19	930				1022
Cj_OXC6865	1061	0021	50	323	19	4	323	712	641	19					1061
Cj_OXC6900	1094	0021	50	323	19	3	323	712	19						1094
Cj_OXC7071	1243	0021	50	323	19	4	323	712	641	19					1243
Cj_OXC7214	1398	0021	50	323	19	3	323	712	19						1398
Cj_OXC5628	1569	0021	50	323	19	4	323	712	641	19					1569
Cj_OXC5906	1672	0021	50	323	19	4	323	712	641	19					1672
Cj_OXC5925	1685	0021	50	323	222	6	323	712	641	19	108	222			1685
Cj_OXC7477	1829	0021	50	323	379	6	323	641	19	892	185	379			1829
Cj_OXC7512	1858	0021	50	323	641	3	323	712	641						1858
Cj_OXC4578	1872	0021	50	323	222	6	323	712	641	19	108	222			1872
Cj_OXC4590	1880	0021	50	323	222	6	323	712	641	19	108	222			1880
Cj_OXC4600	1887	0021	50	323	19	4	323	712	641	19					1887
Cj_OXC7514	1935	0021	50	323	19	3	323	712	19						1935
Cj_OXC7529	1948	0021	50	323	19	4	323	712	641	19					1948
Cj_ARI_916_R	2017	0021	50	323	641	3	323	712	641						2017
Cj_OXC7535	2061	0021	50	323	641	3	323	712	641						2061
Cj_OXC7639	2135	0021	50	323	19	4	323	712	641	19					2135
Cj_OXC5010	2231	0021	50	323	19	4	323	712	641	19					2231
Cj_OXC5049	2258	0021	50	323	641	3	323	712	641						2258
Cj_OXC5131	2300	0021	50	323	930	5	323	712	641	19	930				2300
Cj_OXC5169	2316	0021	50	323	930	5	323	712	641	19	930				2316
Cj_OXC7670	2413	0021	50	323	19	4	323	712	641	19					2413
Cj_OXC7699	2433	0021	50	323	19	4	323	712	641	19					2433
Cj_OXC7866	2478	0021	50	323	19	4	323	712	641	19					2478
Cj_OXC7805	2570	0021	50	323	19	4	323	712	641	19					2570
Cj_OXC7930	2575	0021	50	323	19	4	323	712	641	19					2575
Cj_OXC7961	2704	0021	50	323	19	4	323	712	641	19					2704
Cj_OXC8063	2734	0021	50	323	222	4	323	19	108	222					2734
Cj_E60490	2850	0021	50	323	19	4	323	712	641	19					2850
Cj_Dg370	2941	0021	50	323	19	4	323	712	641	19					2941
Cj_OXC7714	2965	0021	50	323	19	4	323	712	641	19					2965
Cj_OXC8257	3042	0021	50	323	19	4	323	712	641	19					3042
Cj_OXC8278	3055	0021	50	323	19	4	323	712	641	19					3055
Cj_OXC8367	3135	0021	50	323	641	3	323	712	641						3135
Cj_OXC8368	3152	0021	50	323	19	3	323	712	19						3152
Cj_OXC8427	3195	0021	50	323	222	4	323	19	108	222					3195
Cj_OXC8501	3263	0021	50	323	222	6	323	712	641	19	108	222			3263
Cj_OXC8514	3274	0021	50	323	222	6	323	712	641	19	108	222			3274
Cj_E110057	3332	0021	50	323	19	4	323	712	641	19					3332
Cj_E120350	3333	0021	50	323	19	4	323	712	641	19					3333
Cj_C101794	3334	0021	50	323	19	4	323	712	641	19					3334
Cj_C120949	3336	0021	50	323	19	3	323	712	19						3336
Cj_C121664	3337	0021	50	323	19	4	323	712	641	19					3337
Cj_C130520	3338	0021	50	323	19	4	323	712	641	19					3338
Cj_OXC8318R	3407	0021	50	323	19	3	323	712	19						3407
Cj_H140740343	3539	0021	50	323	222	6	323	712	641	19	108	222			3539
Cj_OXC8884	3548	0021	50	323	19	4	323	712	641	19					3548
Cj_OXC8756	3564	0021	50	323	19	4	323	712	641	19					3564
Cj_OXC8901	3685	0021	50	323	641	3	323	712	641						3685
Cj_OXC8980	3745	0021	50	323	19	4	323	712	641	19					3745
Cj_OXC8992	3754	0021	50	323	19	4	323	712	641	19					3754
Cj_OXC5829	1625	0021	50	625	758	7	625	218	291	512	168	720	758		1625
Cj_OXC6326	0286	0021	50	667	665	3	667	668	665						0286
Cj_Le_204R	0076	0021	50	712	19	3	712	641	19						0076
Cj_Le_755	0077	0021	50	712	19	3	712	641	19						0077
Cj_Ma_1	0092	0021	50	712	19	3	712	641	19						0092
Cj_Ma_B	0093	0021	50	712	19	3	712	641	19						0093
Cj_Po_1	0099	0021	50	712	19	3	712	641	19						0099
Cj_Po_2	0100	0021	50	712	19	3	712	641	19						0100
Cj_OXC30	0172	0021	50	712	19	3	712	641	19						0172
Cj_OXC5333	0865	0021	50	712	19	3	712	641	19						0865
Cj_OXC5414	0910	0021	50	712	708	5	712	641	19	151	708				0910
Cj_OXC4801	1137	0021	50	712	19	3	712	641	19						1137
Cj_OXC5045	2255	0021	50	712	19	3	712	641	19						2255
Cj_OXC4249	0220	0021	50	743	640	8	743	261	505	216	673	114	529	640	0220
Cj_ARI_1130_R	2023	0021	50	743	216	4	743	261	505	216					2023
Cj_OXC8331	3104	0021	50	743	254	6	743	261	513	872	621	254			3104
Cj_OXC4930	2551	0021	50	818	963	4	818	722	404	963					2551
Cj_OXC6367	0324	0021	53	172	289	2	172	289							0324
Cj_OXC6516	0442	0021	53	172	289	2	172	289							0442
Cj_OXC6682	0553	0021	53	172	289	2	172	289							0553
Cj_OXC6741	0605	0021	53	172	289	2	172	289							0605
Cj_OXC6789	0648	0021	53	172	289	2	172	289							0648
Cj_OXC4726	0694	0021	53	172	243	4	172	289	780	243					0694
Cj_OXC4754	0713	0021	53	172	289	2	172	289							0713
Cj_OXC5378	0888	0021	53	172	289	2	172	289							0888
Cj_OXC6856	1053	0021	53	172	289	2	172	289							1053
Cj_OXC6862	1059	0021	53	172	289	2	172	289							1059
Cj_OXC6876	1071	0021	53	172	289	2	172	289							1071
Cj_OXC6927	1119	0021	53	172	289	2	172	289							1119
Cj_OXC5823	1622	0021	53	172	289	2	172	289							1622
Cj_OXC5880	1654	0021	53	172	289	2	172	289							1654
Cj_OXC5909	1675	0021	53	172	289	2	172	289							1675
Cj_OXC5825	1699	0021	53	172	289	2	172	289							1699

Cj_OXC7526	1945	0021	53	172	289	2	172	289								1945
Cj_C_389_R	2030	0021	53	172	289	2	172	289								2030
Cj_C_391_R	2031	0021	53	172	289	2	172	289								2031
Cj_OXC4973	2212	0021	53	172	289	2	172	289								2212
Cj_OXC5218	2349	0021	53	172	289	2	172	289								2349
Cj_OXC5219	2350	0021	53	172	289	2	172	289								2350
Cj_OXC8559	3342	0021	53	172	289	2	172	289								3342
Cj_OXC6558	0189	0021	53	743	216	3	743	938	216							0189
Cj_OXC6282	0247	0021	53	743	216	3	743	938	216							0247
Cj_OXC6324	0284	0021	53	743	159	3	743	938	159							0284
Cj_OXC6643	0516	0021	53	743	154	3	743	938	154							0516
Cj_OXC5696	0973	0021	53	743	728	3	743	938	728							0973
Cj_OXC7230	1411	0021	53	743	216	3	743	938	216							1411
Cj_OXC5617	1562	0021	53	743	797	6	743	938	24	77	563	797				1562
Cj_OXC5918	1683	0021	53	743	728	3	743	938	728							1683
Cj_OXC5804	1698	0021	53	743	216	3	743	938	216							1698
Cj_OXC7398	1769	0021	53	743	216	3	743	938	216							1769
Cj_OXC7468	1822	0021	53	743	216	3	743	938	216							1822
Cj_OXC7470	1824	0021	53	743	216	3	743	938	216							1824
Cj_OXC4576	1870	0021	53	743	216	3	743	938	216							1870
Cj_OXC4592	1881	0021	53	743	216	3	743	938	216							1881
Cj_OXC7573	2089	0021	53	743	216	3	743	938	216							2089
Cj_OXC7603	2109	0021	53	743	216	3	743	938	216							2109
Cj_OXC5221	2352	0021	53	743	216	3	743	938	216							2352
Cj_OXC7910	2509	0021	53	743	216	3	743	938	216							2509
Cj_OXC8106	2660	0021	53	743	154	3	743	938	154							2660
Cj_OXC8107	2750	0021	53	743	154	3	743	938	154							2750
Cj_OXC5311	2778	0021	53	743	216	3	743	938	216							2778
Cj_OXC8327	3100	0021	53	743	216	3	743	938	216							3100
Cj_OXC8330	3103	0021	53	743	216	3	743	938	216							3103
Cj_OXC8394	3169	0021	53	743	216	3	743	938	216							3169
Cj_OXC8396	3171	0021	53	743	154	3	743	938	154							3171
Cj_OXC8411	3183	0021	53	743	216	3	743	938	216							3183
Cj_OXC8414	3186	0021	53	743	154	3	743	938	154							3186
Cj_OXC8424	3193	0021	53	743	154	3	743	938	154							3193
Cj_OXC8449	3214	0021	53	743	154	3	743	938	154							3214
Cj_OXC8481	3245	0021	53	743	728	3	743	938	728							3245
Cj_OXC8505	3266	0021	53	743	216	3	743	938	216							3266
Cj_OXC8599	3369	0021	53	743	728	3	743	938	728							3369
Cj_OXC8609	3377	0021	53	743	154	3	743	938	154							3377
Cj_OXC8605	3417	0021	53	743	154	3	743	938	154							3417
Cj_OXC8606	3418	0021	53	743	154	3	743	938	154							3418
Cj_OXC8848	3640	0021	53	743	216	3	743	938	216							3640
Cj_OXC8859	3650	0021	53	743	216	3	743	938	216							3650
Cj_OXC8916	3698	0021	53	743	728	3	743	938	728							3698
Cj_OXC8929	3711	0021	53	743	154	3	743	938	154							3711
Cj_OXC8951	3731	0021	53	743	154	3	743	938	154							3731
Cj_OXC8999	3759	0021	53	743	216	3	743	938	216							3759
Cj_OXC8337	3110	0021	53	773	330	5	773	836	716	274	330					3110
Cj_OXC8489	3252	0021	53	773	330	5	773	836	716	274	330					3252
Cj_OXC4075	0219	0021	104	172	196	3	172	289	196							0219
Cj_OXC6833	0688	0021	104	172	196	3	172	289	196							0688
Cj_OXC7336	1352	0021	104	172	196	3	172	289	196							1352
Cj_OXC7384	1757	0021	104	172	196	3	172	289	196							1757
Cj_OXC7427	1791	0021	104	172	196	3	172	289	196							1791
Cj_OXC6505	0432	0021	141	235	24	3	235	426	24							0432
Cj_OXC8338	3111	0021	141	235	24	3	235	426	24							3111
Cj_OXC5774	1024	0021	190	22	24	3	22	23	24							1024
Cj_OXC5251	2373	0021	190	22	24	3	22	23	24							2373
Cj_OXC3817	0218	0021	262	743	261	6	743	487	773	923	836	261				0218
Cj_OXC6340	0298	0021	262	743	261	3	743	836	261							0298
Cj_OXC6766	0626	0021	262	743	274	7	743	487	773	836	261	716	274			0626
Cj_OXC6824	0681	0021	262	743	274	7	743	487	773	836	261	716	274			0681
Cj_OXC6852	1049	0021	262	743	850	9	743	487	261	46	262	156	650	95	850	1049
Cj_OXC7091	1259	0021	262	743	274	7	743	487	773	836	261	716	274			1259
Cj_OXC7365	1740	0021	262	743	274	7	743	487	773	836	261	716	274			1740
Cj_OXC5168	2315	0021	262	743	261	4	743	923	836	261						2315
Cj_OXC8085	2654	0021	262	743	7	8	743	487	773	836	261	716	274	7		2654
Cj_OXC4961	2776	0021	262	743	274	7	743	487	773	836	261	716	274			2776
Cj_OXC8139	2985	0021	262	743	274	5	743	487	261	716	274					2985
Cj_OXC8476	3240	0021	262	743	716	6	743	487	773	836	261	716				3240
Cj_OXC8529	3288	0021	262	743	261	6	743	487	773	923	836	261				3288
Cj_OXC8547	3303	0021	262	743	289	6	743	487	773	923	261	289				3303
Cj_OXC8921	3703	0021	262	743	836	5	743	487	773	923	836					3703
Cj_OXC7118	0813	0021	266	775	597	3	775	860	597							0813
Cj_OXC5737	0999	0021	266	775	597	3	775	860	597							0999
Cj_OXC7049	1225	0021	266	775	597	3	775	860	597							1225
Cj_OXC8184	2986	0021	266	775	597	3	775	860	597							2986
Cj_OXC4651	1912	0021	520	690	296	4	690	244	290	296						1912
Cj_OXC6301	0264	0021	861	743	962	6	743	22	487	773	938	962				0264
Cj_OXC4584	1876	0021	861	743	962	5	743	22	487	938	962					1876
Cj_OXC4932	2053	0021	861	743	962	6	743	22	487	773	938	962				2053
Cj_OXC8428	3196	0021	861	743	962	6	743	22	487	773	938	962				3196
Cj_OXC6479	0407	0021	883	487	863	4	487	773	752	863						0407
Cj_OXC5671	0958	0021	883	487	639	4	487	773	311	639						0958
Cj_OXC5639	1577	0021	883	487	639	4	487	773	311	639						1577
Cj_OXC8451	3215	0021	883	487	181	3	487	773	181							3215
Cj_CF93-6	0055	0021	883	768	658	5	768	444	768	711	658					0055
Cj_OXC7504	1852	0021	982	743	797	8	743	938	736	24	77	563	26	797		1852
Cj_OXC5624	2561	0021	982	938	915	3	938	281	915							2561
Cj_OXC6300	0263	0021	2135	596	425	2	596	425								0263
Cj_OXC6500	0428	0021	2135	596	425	2	596	425								0428
Cj_OXC6752	0615	0021	2135	596	425	2	596	425								0615
Cj_OXC6781	0641	0021	2135	596	425	2	596	425								0641
Cj_OXC6782	0642	0021	2135	596	425	2	596	425								0642
Cj_OXC6786	0645	0021	2135	596	425	2	596	425								0645
Cj_OXC4744	0707	0021	2135	596	425	2	596	425								0707
Cj_OXC4749	0712	0021	2135	596	425	2	596	425								0712
Cj_OXC4762	0716	0021	2135	596	425	2	596	425								0716
Cj_OXC4765	0718	0021	2135	596	425	2	596	425								0718
Cj_OXC4770	0721	0021	2135	596	425	2	596	425								0721
Cj_OXC5772	1023	0021	2135	596	425	2	596	425								1023
Cj_OXC4791	1130	0021	2135	596	425	2	596	425								1130
Cj_OXC4795	1133	0021	2135	596	425	2	596	425								1133
Cj_OXC4831	1149	0021	2135	596	425	2	596	425								1149
Cj_OXC4836	1151	0021	2135	596	425	2	596	425								1151
Cj_OXC7046	1222	0021	2135	596	425	2	596	425								1222
Cj_OXC5811	1610	0021	2135	596	425	2	596	425								1610
Cj_OXC5821	1620	0021	2135	596	425	2	596	425								1620
Cj_OXC5841	1632	0021	2135	596	425	2	596	425								1632
Cj_OXC5888	1660	0021	2135	596	425	2	596	425								1660
Cj_OXC5895	1666	0021	2135	596	425	2	596	425								1666
Cj_CJ6752_R	2005	0021	2135	596	425	2	596	425								2005
Cj_C258_cj_R	2029	0021	2135	596	425	2	596	425								2029
Cj_OXC4901	2046	0021	2135	596	425	2	596	425								2046
Cj_OXC4904	2048	0021	2135	596	425	3	596	425	425							2048
Cj_OXC4990	2221	0021	2135	596	425	2	596	425								2221
Cj_OXC5002	2226	0021	2135	596	425	2	596	425								2226
Cj_OXC5004	2228	0021	2135	596	425	2	596	425								2228
Cj_OXC5039	2252	0021	2135	596	425	2	596	425								2252
Cj_OXC5067	2268	0021	2135	596	425	2	596	425								2268
Cj_OXC5117	2288	0021	2135	596	425	2	596	425								2288

Cj_OXC5229	2359	0021	2135	596	425	2	596	425										2359
Cj_OXC8438	3205	0021	2135	596	425	2	596	425										3205
Cj_OXC6514	0440	0021	3102	743	809	3	743	261	809									0440
Cj_OXC5416	0911	0021	3574	743	513	3	743	261	513									0911
Cj_OXC7547	2068	0021	3574	743	513	3	743	261	513									2068
Cj_OXC8874	3663	0021	3574	743	513	3	743	261	513									3663
Cj_OXC6552	0183	0021	3769	743	46	6	743	261	513	621	734	46						0183
Cj_OXC6798	0657	0021	4526	487	914	5	487	938	261	513	914							0657
Cj_OXC5125	2294	0021	4526	487	914	5	487	938	261	513	914							2294
Cj_OXC5126	2295	0021	4526	487	914	5	487	938	261	513	914							2295
Cj_OXC5431	0920	0021	5018	743	487	3	743	938	487									0920
Cj_OXC7538	2064	0021	5018	743	487	3	743	938	487									2064
Cj_OXC8035	2726	0021	5018	743	487	3	743	938	487									2726
Cj_OXC8443	3210	0021	5649	334	398	4	334	181	216	398								3210
Cj_OXC6379	0332	0021	5726	775	784	6	775	541	860	649	874	784						0332
Cj_OXC6988	0779	0021	5726	775	465	7	775	541	860	649	874	107	465					0779
Cj_OXC6985	0777	0021	6135	323	945	3	323	712	945									0777
Cj_OXC7896	2499	0021	6892	743	649	5	743	487	775	860	649							2499
Cj_OXC7981	2629	0021	6892	743	649	5	743	487	775	860	649							2629
Cj_OXC7955	2785	0021	6894	625	735	11	625	218	274	33	261	781	597	235	55	866	735	2785
Cj_OXC8198	3000	0021	6983	775	465	7	775	541	860	649	874	107	465					3000
Cj_OXC5623	1566	0021	7172	596	425	2	596	425										1566
Cj_ICDCCJ07002	0073	0021	7244	323	801	4	323	743	24	801								0073
Cj_G113	0067	0022	22	9	550	9	9	952	387	288	8	753	387	130	550			0067
Cj_OXC6641	0514	0022	22	9	387	6	9	288	8	773	753	387						0514
Cj_OXC6644	0517	0022	22	9	387	6	9	288	8	773	753	387						0517
Cj_OXC6645	0518	0022	22	9	387	6	9	288	8	773	753	387						0518
Cj_OXC6866	1062	0022	22	9	387	6	9	288	8	773	753	387						1062
Cj_OXC6887	1082	0022	22	9	387	6	9	288	8	773	753	387						1082
Cj_OXC6888	1083	0022	22	9	387	6	9	288	8	773	753	387						1083
Cj_OXC6902	1095	0022	22	9	387	6	9	288	8	773	753	387						1095
Cj_OXC7085	1255	0022	22	9	387	6	9	288	8	773	753	387						1255
Cj_OXC7178	1365	0022	22	9	387	6	9	288	8	773	753	387						1365
Cj_OXC7463	1817	0022	22	9	387	6	9	288	8	773	753	387						1817
Cj_OXC7490	1841	0022	22	9	387	6	9	288	8	773	753	387						1841
Cj_OXC7809	2171	0022	22	9	387	6	9	288	8	773	753	387						2171
Cj_OXC7838	2195	0022	22	9	387	6	9	288	8	773	753	387						2195
Cj_OXC7942	2780	0022	22	9	387	6	9	288	8	773	753	387						2780
Cj_OXC8043	2802	0022	22	9	387	6	9	288	8	773	753	387						2802
Cj_Dg253	2889	0022	22	9	387	6	9	288	8	773	753	387						2889
Cj_Dg258	2944	0022	22	9	387	6	9	288	8	773	753	387						2944
Cj_OXC8800	3604	0022	22	9	773	5	9	956	288	8	773							3604
Cj_OXC6708	0576	0022	22	14	394	5	14	628	128	579	394							0576
Cj_OXC6796	0655	0022	22	14	9	6	14	628	128	579	394	9						0655
Cj_Dg65a	2853	0022	22	14	394	5	14	628	128	579	394							2853
Cj_OXC8987	3749	0022	22	14	394	5	14	628	128	579	394							3749
Cj_OXC6414	0360	0022	22	406	394	5	406	889	962	50	394							0360
Cj_OXC6626	0500	0022	22	406	962	4	406	889	111	962								0500
Cj_OXC6755	0618	0022	22	406	962	4	406	889	111	962								0618
Cj_OXC6966	0760	0022	22	406	962	4	406	889	111	962								0760
Cj_OXC5383	0891	0022	22	406	962	4	406	889	111	962								0891
Cj_OXC5411	0907	0022	22	406	441	5	406	889	111	962	441							0907
Cj_OXC5443	0928	0022	22	406	962	4	406	889	111	962								0928
Cj_OXC6899	1093	0022	22	406	962	4	406	889	111	962								1093
Cj_OXC5933	1692	0022	22	406	962	4	406	889	111	962								1692
Cj_OXC7666	2409	0022	22	406	962	4	406	889	111	962								2409
Cj_OXC5323	2560	0022	22	406	962	4	406	889	111	962								2560
Cj_OXC5212	2696	0022	22	406	962	4	406	889	111	962								2696
Cj_OXC8169	2762	0022	22	406	962	4	406	889	111	962								2762
Cj_OXC7953	2784	0022	22	406	962	4	406	889	111	962								2784
Cj_OXC8994	3755	0022	22	406	962	4	406	889	111	962								3755
Cj_OXC7158	0848	0022	22	628	54	7	628	128	579	96	489	570	54					0848
Cj_BID1MW	2580	0022	22	676	889	3	676	111	889									2580
Cj_OXC6264	0232	0022	22	773	877	4	773	387	416	877								0232
Cj_OXC7364	1739	0022	22	773	877	4	773	387	416	877								1739
Cj_OXC7367	1742	0022	22	773	877	4	773	387	416	877								1742
Cj_OXC7375	1749	0022	22	773	877	4	773	387	416	877								1749
Cj_OXC7697	2431	0022	22	773	877	4	773	387	416	877								2431
Cj_OXC8868	3658	0022	22	773	877	4	773	387	416	877								3658
Cj_OXC7818	2179	0022	22	889	962	3	889	111	962									2179
Cj_OXC7921	2574	0022	660	932	128	7	932	935	960	182	311	442	128					2574
Cj_BJ-CJD101	0054	0022	3652	9	753	6	9	952	288	8	773	753						0054
Cj_OXC7093	1261	0045	11	14	21	4	14	20	7	21								1261
Cj_OXC8236	3029	0045	11	14	21	4	14	20	7	21								3029
Cj_OXC7102	0798	0045	25	14	21	3	14	20	21									0798
Cj_4031	0039	0045	45	7	25	4	7	11	12	25								0039
Cj_UoH6237	0155	0045	45	7	25	4	7	11	12	25								0155
Cj_UoH6236	0156	0045	45	7	25	4	7	11	12	25								0156
Cj_UoH6538	0157	0045	45	7	25	4	7	11	12	25								0157
Cj_UoH6541	0158	0045	45	7	25	4	7	11	12	25								0158
Cj_UoH6498	0159	0045	45	7	25	4	7	11	12	25								0159
Cj_UoH6497	0160	0045	45	7	25	4	7	11	12	25								0160
Cj_UoH4031	0161	0045	45	7	25	4	7	11	12	25								0161
Cj_UoHIHV116260	0162	0045	45	7	25	4	7	11	12	25								0162
Cj_UoHIHV116292	0163	0045	45	7	25	4	7	11	12	25								0163
Cj_UoH540	0164	0045	45	7	25	4	7	11	12	25								0164
Cj_UoH543	0165	0045	45	7	25	4	7	11	12	25								0165
Cj_UoH544	0166	0045	45	7	25	4	7	11	12	25								0166
Cj_UoHT-71726	0167	0045	45	7	385	6	7	11	21	12	25	385						0167
Cj_UoHT-71727	0168	0045	45	7	385	6	7	11	21	12	25	385						0168
Cj_UoHT-71731	0169	0045	45	7	385	6	7	11	21	12	25	385						0169
Cj_UoHT-71732	0170	0045	45	7	385	6	7	11	21	12	25	385						0170
Cj_OXC6259	0228	0045	45	7	27	6	7	11	12	25	26	27						0228
Cj_OXC6278	0244	0045	45	7	27	6	7	11	12	25	26	27						0244
Cj_OXC6313	0273	0045	45	7	27	6	7	11	12	25	26	27						0273
Cj_OXC6321	0281	0045	45	7	581	6	7	32	33	34	431	581						0281
Cj_OXC6339	0297	0045	45	7	111	5	7	11	12	25	111							0297
Cj_OXC6419	0364	0045	45	7	696	7	7	21	391	12	592	143	696					0364
Cj_OXC6437	0377	0045	45	7	747	6	7	11	391	12	25	747						0377
Cj_OXC6624	0498	0045	45	7	696	6	7	21	391	12	143	696						0498
Cj_OXC6803	0662	0045	45	7	917	5	7	12	686	592	917							0662
Cj_OXC5332	0864	0045	45	7	696	7	7	21	391	12	592	143	696					0864
Cj_OXC6894	1088	0045	45	7	27	6	7	11	12	25	26	27						1088
Cj_OXC6916	1109	0045	45	7	696	6	7	21	12	592	143	696						1109
Cj_OXC7014	1193	0045	45	7	25	4	7	11	12	25								1193
Cj_OXC7018	1197	0045	45	7	25	4	7	11	12	25								1197
Cj_OXC7025	1204	0045	45	7	582	8	7	11	12	25	26	27	163	582				1204
Cj_OXC7074	1246	0045	45	7	753	6	7	11	12	25	710	753						1246
Cj_OXC7090	1258	0045	45	7	25	4	7	11	12	25								1258
Cj_OXC7099	1266	0045	45	7	27	4	7	25	26	27								1266
Cj_OXC7267	1289	0045	45	7	199	6	7	11	12	25	747	199						1289
Cj_OXC7315	1335	0045	45	7	25	4	7	11	12	25								1335
Cj_OXC7186	1373	0045	45	7	25	5	7	11	391	12	25							1373
Cj_OXC7225	1408	0045	45	7	753	6	7	11	12	25	710	753						1408
Cj_OXC7235	1416	0045	45	7	696	7	7	21	391	12	592	143	696					1416
Cj_OXC7239	1419	0045	45	7	25	4	7	11	12	25								1419
Cj_OXC5850	1704	0045	45	7	25	4	7	11	12	25								1704
Cj_OXC7369	1743	0045	45	7	421	8	7	11	12	25	26	27	237	421				1743
Cj_OXC7401	1771	0045	45	7	696	7	7	21	391	12	592	143	696					1771
Cj_OXC7634	2131	0045	45	7	421	8	7	11	12	25	26	27	237	421				2131
Cj_OXC7766	2142	0045	45	7	582	8	7	11	12	25	26	27	163	582				2142
Cj_OXC7776	2149	0045	45	7	674	7	7	11	12	25	26	27	674					2149

Cj_OXC7793	2162	0045	45	7	186	6	7	11	12	25	747	186			2162
Cj_OXC7813	2175	0045	45	7	696	6	7	21	391	12	143	696			2175
Cj_OXC5180	2323	0045	45	7	25	5	7	11	391	12	25				2323
Cj_OXC5231	2361	0045	45	7	26	5	7	11	12	25	26				2361
Cj_OXC7659	2402	0045	45	7	336	7	7	11	12	25	26	27	336		2402
Cj_OXC7735	2454	0045	45	7	25	5	7	11	391	12	25				2454
Cj_OXC7911	2510	0045	45	7	674	7	7	11	12	25	26	27	674		2510
Cj_OXC7922	2518	0045	45	7	782	7	7	11	12	25	26	27	782		2518
Cj_OXC7923	2519	0045	45	7	782	7	7	11	12	25	26	27	782		2519
Cj_OXC8007	2716	0045	45	7	27	6	7	11	12	25	26	27			2716
Cj_OXC8065	2736	0045	45	7	25	4	7	391	12	25					2736
Cj_OXC7998	2806	0045	45	7	696	6	7	21	391	12	143	696			2806
Cj_OXC8072	2812	0045	45	7	25	5	7	11	391	12	25				2812
Cj_OXC7708	2829	0045	45	7	25	5	7	11	391	12	25				2829
Cj_Dg292	2876	0045	45	7	27	6	7	11	12	25	26	27			2876
Cj_Dg200	2892	0045	45	7	111	5	7	11	12	25	111				2892
Cj_Dg161	2918	0045	45	7	747	5	7	11	12	25	747				2918
Cj_Dg206	2920	0045	45	7	111	5	7	11	12	25	111				2920
Cj_Dg162	2926	0045	45	7	747	5	7	11	12	25	747				2926
Cj_Dg202	2928	0045	45	7	111	5	7	11	12	25	111				2928
Cj_Dg273	2939	0045	45	7	111	5	7	11	12	25	111				2939
Cj_Dg383	2958	0045	45	7	111	5	7	11	12	25	111				2958
Cj_OXC8195	2997	0045	45	7	111	5	7	11	12	25	111				2997
Cj_OXC8362	3131	0045	45	7	674	7	7	11	12	25	26	27	674		3131
Cj_Dg282_R	3137	0045	45	7	747	5	7	11	12	25	747				3137
Cj_OXC8483	3247	0045	45	7	27	5	7	11	25	26	27				3247
Cj_OXC6137	3519	0045	45	7	747	5	7	11	12	25	747				3519
Cj_OXC8993	3553	0045	45	7	557	6	7	11	391	12	25	557			3553
Cj_OXC8841	3634	0045	45	7	25	4	7	11	12	25					3634
Cj_OXC8844	3637	0045	45	7	25	4	7	11	12	25					3637
Cj_OXC8857	3648	0045	45	7	25	4	7	11	12	25					3648
Cj_OXC8894	3680	0045	45	7	25	4	7	11	12	25					3680
Cj_OXC8972	3742	0045	45	7	111	5	7	11	12	25	111				3742
Cj_OXC5171	2318	0045	45	11	111	4	11	12	25	111					2318
Cj_OXC5225	2355	0045	45	11	292	5	11	12	25	111	292				2355
Cj_OXC5236	2363	0045	45	11	292	5	11	12	25	111	292				2363
Cj_OXC7972	2621	0045	45	12	111	4	12	25	84	111					2621
Cj_OXC8919	3701	0045	45	12	111	3	12	25	111						3701
Cj_OXC6594	0473	0045	45	14	31	6	14	20	7	11	21	31			0473
Cj_OXC7169	0858	0045	45	14	31	6	14	20	7	11	21	31			0858
Cj_OXC7340	1355	0045	45	14	11	3	14	20	11						1355
Cj_OXC7179	1366	0045	45	14	11	4	14	20	7	11					1366
Cj_OXC7229	1942	0045	45	14	31	6	14	20	7	11	21	31			1942
Cj_OXC5130	2299	0045	45	14	33	6	14	20	7	21	32	33			2299
Cj_OXC5164	2312	0045	45	14	772	4	14	20	21	772					2312
Cj_OXC8170	2763	0045	45	14	361	7	14	20	7	11	21	31	361		2763
Cj_OXC8906	3690	0045	45	14	31	6	14	20	7	11	21	31			3690
Cj_OXC8937	3719	0045	45	14	919	4	14	7	11	919					3719
Cj_UoH4028	0152	0045	45	20	35	7	20	7	21	32	33	34	35		0152
Cj_UoH4947	0153	0045	45	20	35	7	20	7	21	32	33	34	35		0153
Cj_UoH4948	0154	0045	45	20	35	7	20	7	21	32	33	34	35		0154
Cj_OXC6536	0461	0045	45	20	35	5	20	7	21	34	35				0461
Cj_OXC6797	0656	0045	45	20	35	7	20	7	21	32	33	34	35		0656
Cj_OXC7129	0822	0045	45	20	804	7	20	7	21	32	33	34	804		0822
Cj_OXC7216	1400	0045	45	20	35	7	20	7	21	32	33	34	35		1400
Cj_OXC7233	1414	0045	45	20	35	7	20	7	21	32	33	34	35		1414
Cj_OXC4893	2043	0045	45	20	35	5	20	7	33	34	35				2043
Cj_OXC7745	2461	0045	45	20	400	8	20	7	21	32	33	34	35	400	2461
Cj_OXC8238	3030	0045	45	20	400	8	20	7	21	32	33	34	35	400	3030
Cj_OXC6293	0258	0045	45	21	152	4	21	592	25	152					0258
Cj_OXC8578	3353	0045	45	21	152	4	21	592	25	152					3353
Cj_OXC7284	1305	0045	45	32	302	5	32	33	34	35	302				1305
Cj_OXC5816	1615	0045	45	198	443	6	198	749	590	316	727	443			1615
Cj_OXC7788	2157	0045	45	198	155	7	198	749	590	316	727	443	155		2157
Cj_OXC5090	2278	0045	45	198	129	8	198	749	590	316	727	443	279	129	2278
Cj_OXC7061	1234	0045	45	591	69	6	591	424	305	813	301	69			1234
Cj_OXC7484	1835	0045	45	591	69	6	591	424	305	813	301	69			1835
Cj_OXC8228	3021	0045	45	591	69	6	591	424	305	813	301	69			3021
Cj_M1	0091	0045	137	14	21	3	14	7	21						0091
Cj_OXC6448	0382	0045	137	14	16	3	14	15	16						0382
Cj_OXC6592	0471	0045	137	14	629	5	14	585	20	21	629				0471
Cj_OXC4721	0691	0045	137	14	16	3	14	15	16						0691
Cj_OXC5331	0863	0045	137	14	16	3	14	15	16						0863
Cj_OXC5434	0922	0045	137	14	16	3	14	15	16						0922
Cj_OXC5744	1004	0045	137	14	16	3	14	15	16						1004
Cj_OXC7067	1240	0045	137	14	16	3	14	15	16						1240
Cj_OXC7249	1273	0045	137	14	16	3	14	15	16						1273
Cj_OXC7281	1302	0045	137	14	16	3	14	15	16						1302
Cj_OXC7283	1304	0045	137	14	16	3	14	15	16						1304
Cj_OXC7331	1347	0045	137	14	879	4	14	585	20	879					1347
Cj_OXC7170	1358	0045	137	14	16	3	14	15	16						1358
Cj_OXC7425	1790	0045	137	14	16	3	14	15	16						1790
Cj_OXC4907	2051	0045	137	14	16	4	14	823	15	16					2051
Cj_OXC7780	2153	0045	137	14	879	4	14	585	20	879					2153
Cj_OXC7782	2155	0045	137	14	879	4	14	585	20	879					2155
Cj_OXC7786	2156	0045	137	14	16	3	14	15	16						2156
Cj_OXC5032	2246	0045	137	14	16	3	14	15	16						2246
Cj_OXC5318	2558	0045	137	14	16	3	14	15	16						2558
Cj_OXC7979	2628	0045	137	14	16	3	14	15	16						2628
Cj_OXC8118	2663	0045	137	14	16	3	14	15	16						2663
Cj_OXC8016	2718	0045	137	14	16	3	14	15	16						2718
Cj_OXC8100	2747	0045	137	14	879	4	14	585	20	879					2747
Cj_Dg374	2930	0045	137	14	430	8	14	585	20	21	21	879	629	430	2930
Cj_Dg379	2938	0045	137	14	430	8	14	585	20	21	21	879	629	430	2938
Cj_Dg382	2953	0045	137	14	430	8	14	585	20	21	21	879	629	430	2953
Cj_OXC8246	3033	0045	137	14	16	3	14	15	16						3033
Cj_OXC8388	3164	0045	137	14	16	3	14	15	16						3164
Cj_OXC8842	3635	0045	137	14	14	3	14	15	14						3635
Cj_OXC8843	3636	0045	137	14	16	3	14	15	16						3636
Cj_OXC8956	3734	0045	137	14	16	3	14	15	16						3734
Cj_OXC7344	1722	0045	334	737	643	4	737	481	567	643					1722
Cj_OXC7346	1724	0045	334	737	643	4	737	481	567	643					1724
Cj_OXC5129	2298	0045	334	737	643	5	737	481	567	760	643				2298
Cj_OXC8062	2733	0045	334	737	643	5	737	481	567	760	643				2733
Cj_OXC8055	2809	0045	334	737	643	5	737	481	567	760	643				2809
Cj_OXC7113	0808	0045	538	14	620	6	14	585	32	607	793	620			0808
Cj_OXC7752	2467	0045	538	14	620	6	14	585	32	607	793	620			2467
Cj_OXC8968	3738	0045	538	14	620	6	14	585	32	607	793	620			3738
Cj_OXC6970	0764	0045	583	14	79	4	14	585	21	79					0764
Cj_OXC7032	1209	0045	583	14	79	4	14	585	21	79					1209
Cj_OXC5091	2279	0045	583	14	79	4	14	585	21	79					2279
Cj_OXC5224	2354	0045	583	14	79	4	14	585	21	79					2354
Cj_OXC8128	2686	0045	583	14	79	4	14	585	21	79					2686
Cj_OXC8961	3735	0045	583	14	79	4	14	585	21	79					3735
Cj_OXC6819	0676	0045	583	566	533	3	566	79	533						0676
Cj_OXC6515	0441	0045	845	823	109	2	823	109							0441
Cj_OXC8809	3610	0045	1003	77	116	2	77	116							3610
Cj_OXC4736	0700	0045	1326	7	21	3	7	11	21						0700
Cj_Dg283	2873	0045	1326	7	21	3	7	11	21						2873
Cj_OXC7371	1745	0045	2109	14	32	4	14	585	823	32					1745
Cj_OXC7376	1750	0045	2109	14	32	4	14	585	823	32					1750
Cj_OXC8372	2971	0045	2109	14	32	4	14	585	823	32					2971

Cj_OXC8375	2974	0045	2109	14	32	4	14	585	823	32								2974
Cj_OXC7377	1751	0045	2109	191	936	4	191	304	493	936								1751
Cj_Dg122	2905	0045	4791	494	5	10	494	635	418	141	637	885	845	23	801	5		2905
Cj_Dg180	2935	0045	4791	494	5	11	494	635	418	141	637	647	885	845	23	801	5	2935
Cj_Dg184	2940	0045	4791	494	5	11	494	635	418	141	637	647	885	845	23	801	5	2940
Cj_Dg187	2952	0045	4791	494	5	10	494	635	418	141	637	885	845	23	801	5		2952
Cj_OXC5208	2343	0045	6017	14	16	3	14	15	16									2343
Cj_Dg57a	2863	0045	7259	68	107	3	68	462	107									2863
Cj_Dg131	2894	0045	7259	68	107	3	68	462	107									2894
Cj_Dg304	2897	0045	7259	68	107	3	68	462	107									2897
Cj_Dg130	2917	0045	7259	68	107	3	68	462	107									2917
Cj_Dg104	2943	0045	7259	68	107	3	68	462	107									2943
Cj_Dg137	2932	0045	7259	333	105	4	333	333	713	105								2932
Cj_Dg246	2959	0045	7259	333	105	3	333	713	105									2959
Cj_OXC5716	0987	0048	38	848	705	4	848	223	832	705								0987
Cj_OXC6549	0181	0048	48	22	29	3	22	692	29									0181
Cj_OXC6265	0233	0048	48	22	29	4	22	692	216	29								0233
Cj_OXC6328	0288	0048	48	22	29	4	22	692	216	29								0288
Cj_OXC6377	0331	0048	48	22	29	4	22	692	216	29								0331
Cj_OXC6411	0357	0048	48	22	29	4	22	692	216	29								0357
Cj_OXC4779	0728	0048	48	22	29	4	22	692	216	29								0728
Cj_OXC6973	0766	0048	48	22	29	4	22	692	216	29								0766
Cj_OXC7143	0834	0048	48	22	29	4	22	692	216	29								0834
Cj_OXC7165	0854	0048	48	22	212	6	22	692	692	216	29	212						0854
Cj_OXC5334	0866	0048	48	22	29	4	22	692	216	29								0866
Cj_OXC5405	0903	0048	48	22	29	4	22	692	216	29								0903
Cj_OXC4813	1145	0048	48	22	29	4	22	692	216	29								1145
Cj_OXC7012	1191	0048	48	22	100	5	22	692	216	29	100							1191
Cj_OXC7016	1195	0048	48	22	29	4	22	692	216	29								1195
Cj_OXC7280	1301	0048	48	22	230	4	22	692	838	230								1301
Cj_OXC7174	1362	0048	48	22	212	5	22	692	216	29	212							1362
Cj_OXC5874	1650	0048	48	22	29	4	22	692	216	29								1650
Cj_OXC7388	1761	0048	48	22	212	5	22	692	216	29	212							1761
Cj_OXC7486	1837	0048	48	22	29	5	22	692	692	216	29							1837
Cj_OXC4603	1889	0048	48	22	29	4	22	692	216	29								1889
Cj_OXC4680	1932	0048	48	22	29	4	22	692	216	29								1932
Cj_ARI_1129_R	2022	0048	48	22	29	4	22	692	692	29								2022
Cj_CJ7016_R	2039	0048	48	22	29	4	22	692	216	29								2039
Cj_OXC7769	2145	0048	48	22	29	4	22	692	216	29								2145
Cj_OXC5150	2310	0048	48	22	29	4	22	692	216	29								2310
Cj_OXC5228	2358	0048	48	22	29	4	22	692	216	29								2358
Cj_OXC5242	2367	0048	48	22	29	4	22	692	216	29								2367
Cj_OXC8066	2646	0048	48	22	29	4	22	692	216	29								2646
Cj_OXC8040	2728	0048	48	22	29	4	22	692	216	29								2728
Cj_OXC8064	2735	0048	48	22	29	4	22	692	216	29								2735
Cj_OXC7483	2826	0048	48	22	77	6	22	692	216	29	429	77						2826
Cj_OXC8206	3006	0048	48	22	29	4	22	692	216	29								3006
Cj_OXC8232	3025	0048	48	22	216	3	22	692	216									3025
Cj_OXC8317	3090	0048	48	22	519	7	22	692	692	216	29	178	519					3090
Cj_OXC8473	3237	0048	48	22	212	6	22	692	692	216	29	212						3237
Cj_OXC8490	3253	0048	48	22	29	4	22	692	216	29								3253
Cj_OXC8535	3293	0048	48	22	231	5	22	692	216	29	231							3293
Cj_OXC8561	3343	0048	48	22	231	5	22	692	216	29	231							3343
Cj_OXC8710	3462	0048	48	22	540	6	22	692	216	29	77	540						3462
Cj_OXC8812	3551	0048	48	22	29	4	22	692	216	29								3551
Cj_OXC8791	3595	0048	48	22	429	5	22	692	216	29	429							3595
Cj_OXC8795	3599	0048	48	22	29	4	22	692	216	29								3599
Cj_OXC8925	3707	0048	48	22	29	4	22	692	216	29								3707
Cj_OXC6345	0302	0048	48	216	29	2	216	29										0302
Cj_OXC6917	1110	0048	48	216	29	2	216	29										1110
Cj_OXC8920	3702	0048	48	216	429	3	216	29	429									3702
Cj_OXC8983	3746	0048	48	217	29	2	217	29										3746
Cj_OXC6421	0366	0048	48	569	134	6	569	585	20	823	79	134						0366
Cj_OXC6693	0562	0048	48	569	134	6	569	585	20	823	79	134						0562
Cj_OXC4767	0719	0048	48	569	134	8	569	585	20	823	7	21	79	134				0719
Cj_OXC7112	0807	0048	48	569	134	6	569	585	20	823	79	134						0807
Cj_OXC7151	0842	0048	48	569	134	6	569	585	20	823	79	134						0842
Cj_OXC5711	0983	0048	48	569	138	8	569	585	823	7	21	79	100	138				0983
Cj_OXC4846	1155	0048	48	569	274	8	569	585	823	7	21	79	100	274				1155
Cj_OXC7005	1184	0048	48	569	79	5	569	585	20	823	79							1184
Cj_OXC7262	1284	0048	48	569	138	8	569	585	823	7	21	79	100	138				1284
Cj_OXC7269	1291	0048	48	569	134	6	569	585	20	823	79	134						1291
Cj_OXC5814	1613	0048	48	569	138	8	569	585	823	7	21	79	100	138				1613
Cj_OXC7383	1756	0048	48	569	134	6	569	585	20	823	79	134						1756
Cj_OXC7461	1815	0048	48	569	79	7	569	585	20	823	7	21	79					1815
Cj_OXC4562	1862	0048	48	569	138	8	569	585	823	7	21	79	100	138				1862
Cj_OXC7524	1943	0048	48	569	736	5	569	79	134	375	736							1943
Cj_ARI_1332_R	2026	0048	48	569	138	8	569	585	823	7	21	79	100	138				2026
Cj_OXC7799	2166	0048	48	569	138	8	569	585	823	7	21	79	100	138				2166
Cj_OXC7821	2182	0048	48	569	863	8	569	585	20	823	79	283	249	863				2182
Cj_OXC7834	2191	0048	48	569	138	8	569	585	823	7	21	79	100	138				2191
Cj_OXC4981	2215	0048	48	569	138	8	569	585	823	7	21	79	100	138				2215
Cj_OXC7747	2463	0048	48	569	138	8	569	585	823	7	21	79	100	138				2463
Cj_OXC8120	2754	0048	48	569	79	5	569	585	20	823	79							2754
Cj_OXC8595	3366	0048	48	569	79	5	569	585	20	823	79							3366
Cj_OXC8652	3402	0048	48	569	835	7	569	585	20	823	79	411	835					3402
Cj_OXC8612	3420	0048	48	569	79	5	569	585	20	823	79							3420
Cj_OXC8869	3659	0048	48	569	79	5	569	585	20	823	79							3659
Cj_OXC8904	3688	0048	48	569	138	8	569	585	823	7	21	79	100	138				3688
Cj_OXC8915	3697	0048	48	569	134	6	569	585	20	823	79	134						3697
Cj_H143900546	3780	0048	48	569	79	5	569	823	7	21	79							3780
Cj_H143900545	3783	0048	48	569	79	5	569	823	7	21	79							3783
Cj_OXC6648	0521	0048	48	692	736	4	692	216	29	736								0521
Cj_OXC6652	0524	0048	48	692	736	4	692	216	29	736								0524
Cj_OXC6881	1076	0048	48	692	736	4	692	216	29	736								1076
Cj_OXC7820	2181	0048	48	692	29	3	692	216	29									2181
Cj_OXC7750	2466	0048	48	692	29	3	692	216	29									2466
Cj_OXC7703	2664	0048	48	692	29	4	692	692	216	29								2664
Cj_OXC8125	2684	0048	48	692	29	3	692	216	29									2684
Cj_OXC8343	3115	0048	48	692	17	5	692	216	29	460	17							3115
Cj_OXC8826	3621	0048	48	692	29	3	692	216	29									3621
Cj_D2600	0062	0048	429	261	9	3	261	229	9									0062
Cj_OXC6413	0359	0048	429	261	9	3	261	229	9									0359
Cj_OXC8948	3728	0048	492	216	29	2	216	29										3728
Cj_OXC6272	0239	0048	844	22	216	3	22	692	216									0239
Cj_OXC8708	3460	0048	2343	690	440	7	690	290	290	874	364	263	440					3460
Cj_OXC5188	2329	0048	5183	569	79	7	569	585	20	823	7	21	79					2329
Cj_OXC7374	1748	0048	6754	22	29	4	22	692	216	29								1748
Cj_OXC7525	1944	0048	6754	22	29	4	22	692	216	29								1944
Cj_OXC7673	2415	0048	6754	22	29	4	22	692	216	29								2415
Cj_OXC7197	1384	0049	49	698	596	5	698	315	571	258	596							1384
Cj_OXC5854	1639	0049	49	698	315	2	698	315										1639
Cj_OXC7885	2572	0049	49	698	134	5	698	315	596	559	134							2572
Cj_OXC8127	2772	0049	49	698	315	2	698	315										2772
Cj_OXC8995	3756	0049	49	698	449	6	698	315	872	575	855	449						3756
Cj_OXC8671	3434	0049	7305	743	111	6	743	261	23	773	773	111						3434
Cj_OXC6368	0325	0052	52	625	781	7	625	218	274	455	487	261	781					0325
Cj_OXC6441	0380	0052	52	625	781	6	625	218	274	455	261	781						0380
Cj_OXC5900	1669	0052	52	625	261	6	625	218	274	455	487	261						1669
Cj_OXC5917	1682	0052	52	625	261	6	625	218	274	455	487	261						1682
Cj_OXC7528	1947	0052	52	625	781	6	625	274	455	487	261	781						1947
Cj_OXC6737	0602	0052	52	818	204	4	818	722	490	204								0602

Cj_OXC5674	0961	0052	52	818	269	4	818	722	851	269				0961
Cj_OXC6908	1101	0052	52	818	134	3	818	722	134					1101
Cj_OXC7044	1220	0052	52	818	269	4	818	722	851	269				1220
Cj_OXC7307	1327	0052	52	818	134	3	818	722	134					1327
Cj_OXC5205	2340	0052	52	818	134	3	818	722	134					2340
Cj_OXC5215	2348	0052	52	818	140	5	818	722	851	823	140			2348
Cj_OXC8181	2672	0052	52	818	269	4	818	722	851	269				2672
Cj_OXC8172	2764	0052	52	818	823	4	818	722	851	823				2764
Cj_OXC8023	2797	0052	52	818	823	4	818	722	851	823				2797
Cj_OXC7715	2966	0052	52	818	269	4	818	722	851	269				2966
Cj_OXC8209	3009	0052	52	818	269	4	818	722	851	269				3009
Cj_OXC8349	3120	0052	52	818	268	3	818	722	268					3120
Cj_OXC8286	3147	0052	52	818	366	4	818	722	249	366				3147
Cj_OXC8366	3151	0052	52	818	134	3	818	722	134					3151
Cj_OXC8426	3194	0052	52	818	366	4	818	722	249	366				3194
Cj_20176	0032	0052	161	577	529	5	577	331	502	589	529			0032
Cj_OXC4646	1910	0052	775	250	576	3	250	107	576					1910
Cj_10186	0008	0052	1919	732	716	4	732	468	8	716				0008
Cj_OXC6570	0199	0061	61	172	909	4	172	289	422	909				0199
Cj_OXC6283	0248	0061	61	172	49	3	172	289	49					0248
Cj_OXC6346	0303	0061	61	172	909	4	172	289	422	909				0303
Cj_OXC6453	0386	0061	61	172	895	3	172	289	895					0386
Cj_OXC6490	0418	0061	61	172	74	4	172	289	417	74				0418
Cj_OXC6632	0505	0061	61	172	289	2	172	289						0505
Cj_OXC6688	0557	0061	61	172	289	2	172	289						0557
Cj_OXC6769	0629	0061	61	172	74	4	172	289	417	74				0629
Cj_OXC6775	0635	0061	61	172	74	4	172	289	417	74				0635
Cj_OXC6808	0667	0061	61	172	289	2	172	289						0667
Cj_OXC6978	0771	0061	61	172	453	3	172	289	453					0771
Cj_OXC5439	0926	0061	61	172	909	4	172	289	422	909				0926
Cj_OXC5665	0955	0061	61	172	74	4	172	289	417	74				0955
Cj_OXC4789	1129	0061	61	172	537	3	172	289	537					1129
Cj_OXC4798	1135	0061	61	172	537	3	172	289	537					1135
Cj_OXC7019	1198	0061	61	172	289	2	172	289						1198
Cj_OXC7021	1200	0061	61	172	289	2	172	289						1200
Cj_OXC7023	1202	0061	61	172	289	2	172	289						1202
Cj_OXC7294	1315	0061	61	172	736	3	172	289	736					1315
Cj_OXC7325	1342	0061	61	172	701	3	172	289	701					1342
Cj_OXC5493	1552	0061	61	172	356	3	172	289	356					1552
Cj_OXC5860	1643	0061	61	172	909	3	172	422	909					1643
Cj_OXC5875	1709	0061	61	172	913	3	172	289	913					1709
Cj_OXC7433	1796	0061	61	172	74	4	172	289	417	74				1796
Cj_OXC7452	1809	0061	61	172	74	4	172	289	417	74				1809
Cj_OXC7472	1826	0061	61	172	74	4	172	289	417	74				1826
Cj_OXC4560	1861	0061	61	172	383	4	172	289	371	383				1861
Cj_OXC4579	1873	0061	61	172	453	3	172	289	453					1873
Cj_OXC4595	1883	0061	61	172	531	4	172	289	503	531				1883
Cj_OXC4622	1893	0061	61	172	312	3	172	289	312					1893
Cj_OXC4670	1925	0061	61	172	289	2	172	289						1925
Cj_7802A	1999	0061	61	172	491	4	172	289	701	491				1999
Cj_NC16_R	2021	0061	61	172	289	2	172	289						2021
Cj_NS_36_R	2028	0061	61	172	701	4	172	289	905	701				2028
Cj_OXC7611	2114	0061	61	172	74	4	172	289	417	74				2114
Cj_OXC7833	2190	0061	61	172	312	2	172	312						2190
Cj_OXC7839	2196	0061	61	172	74	4	172	289	417	74				2196
Cj_OXC5111	2286	0061	61	172	289	2	172	289						2286
Cj_OXC5162	2311	0061	61	172	289	2	172	289						2311
Cj_OXC5177	2320	0061	61	172	289	2	172	289						2320
Cj_OXC4658	2546	0061	61	172	49	3	172	289	49					2546
Cj_OXC7343	2676	0061	61	172	74	4	172	289	417	74				2676
Cj_OXC7652	2679	0061	61	172	909	4	172	289	422	909				2679
Cj_Dg327	2859	0061	61	172	289	2	172	289						2859
Cj_OXC8216	3012	0061	61	172	74	3	172	417	74					3012
Cj_OXC8318	3091	0061	61	172	909	4	172	289	422	909				3091
Cj_OXC8344	3116	0061	61	172	74	4	172	289	417	74				3116
Cj_OXC8376	3153	0061	61	172	74	4	172	289	417	74				3153
Cj_OXC8401	3175	0061	61	172	74	4	172	289	417	74				3175
Cj_OXC8643	3395	0061	61	172	453	3	172	289	453					3395
Cj_OXC8494	3486	0061	61	172	74	4	172	289	417	74				3486
Cj_OXC8716	3496	0061	61	172	74	4	172	289	417	74				3496
Cj_OXC8487	3499	0061	61	172	74	4	172	289	417	74				3499
Cj_OXC8744	3558	0061	61	172	289	2	172	289						3558
Cj_OXC8941	3723	0061	61	172	909	4	172	289	422	909				3723
Cj_OXC6268	0235	0061	61	289	701	3	289	744	701					0235
Cj_OXC5844	1634	0061	61	289	49	2	289	49						1634
Cj_OXC7644	2393	0061	61	380	451	3	380	384	451					2393
Cj_OXC7938	2527	0061	1738	172	915	3	172	289	915					2527
Cj_OXC8263	3045	0061	6985	172	74	4	172	289	417	74				3045
Cj_OXC8466	3230	0179	220	229	880	7	229	701	827	434	435	763	880	3230
Cj_OXC7193	1380	0206	122	229	179	4	229	503	846	179				1380
Cj_OXC7350	1727	0206	122	229	846	4	229	874	503	846				1727
Cj_OXC7950	2782	0206	122	229	846	3	229	874	846					2782
Cj_OXC8482	3246	0206	122	229	179	4	229	503	846	179				3246
Cj_OXC8803	3607	0206	122	229	179	4	229	503	846	179				3607
Cj_OXC7292	1313	0206	122	690	296	3	690	244	296					1313
Cj_OXC7355	1731	0206	122	690	296	3	690	244	296					1731
Cj_OXC7918	2516	0206	122	690	593	3	690	690	593					2516
Cj_OXC8094	2657	0206	122	690	296	3	690	244	296					2657
Cj_OXC8664	3430	0206	206	48	260	4	48	671	655	260				3430
Cj_OXC6315	0275	0206	206	689	898	5	689	48	655	260	898			0275
Cj_OXC6522	0448	0206	206	689	260	5	689	48	671	655	260			0448
Cj_OXC6947	0742	0206	206	689	260	5	689	48	671	655	260			0742
Cj_OXC5346	0875	0206	206	689	260	5	689	48	671	655	260			0875
Cj_OXC5740	1001	0206	206	689	260	4	689	48	655	260				1001
Cj_OXC7006	1185	0206	206	689	260	5	689	48	671	655	260			1185
Cj_OXC5939	1695	0206	206	689	260	5	689	48	671	655	260			1695
Cj_CJ6947_R	2012	0206	206	689	260	5	689	48	671	655	260			2012
Cj_OXC7560	2078	0206	206	689	561	6	689	48	655	260	490	561		2078
Cj_OXC7704	2435	0206	206	689	655	4	689	48	671	655				2435
Cj_OXC7899	2501	0206	206	689	260	5	689	48	671	655	260			2501
Cj_OXC7968	2617	0206	206	689	260	5	689	48	671	655	260			2617
Cj_OXC8141	2673	0206	206	689	260	5	689	48	671	655	260			2673
Cj_OXC8002	2712	0206	206	689	260	4	689	48	655	260				2712
Cj_OXC8029	2722	0206	206	689	260	4	689	48	655	260				2722
Cj_OXC8045	2803	0206	206	689	260	4	689	48	655	260				2803
Cj_OXC8147	2833	0206	206	689	260	4	689	48	655	260				2833
Cj_OXC8051	3142	0206	206	689	260	4	689	48	655	260				3142
Cj_OXC8399	3173	0206	206	689	260	5	689	48	671	655	260			3173
Cj_OXC8480	3244	0206	206	689	260	4	689	48	655	260				3244
Cj_OXC8731	3475	0206	206	689	260	4	689	48	655	260				3475
Cj_OXC8950	3730	0206	206	689	736	6	689	48	655	260	630	736		3730
Cj_OXC8953	3732	0206	206	689	260	5	689	48	671	655	260			3732
Cj_OXC6387	0337	0206	227	690	543	4	690	290	68	543				0337
Cj_OXC7117	0812	0206	227	690	543	4	690	290	68	543				0812
Cj_OXC7094	1262	0206	227	690	543	4	690	290	68	543				1262
Cj_OXC7869	2481	0206	227	690	543	4	690	290	68	543				2481
Cj_OXC8151	2666	0206	227	690	543	4	690	290	68	543				2666
Cj_OXC8853	3644	0206	227	690	543	4	690	290	68	543				3644
Cj_OXC6858	1055	0206	273	48	260	4	48	671	655	260				1055
Cj_OXC5255	2374	0206	273	48	260	4	48	671	655	260				2374
Cj_OXC5261	2378	0206	273	48	260	4	48	671	655	260				2378
Cj_OXC6553	0184	0206	273	689	260	5	689	48	671	655	260			0184

Cj_OXC6306	0269	0206	273	689	260	4	689	48	655	260								0269
Cj_OXC6307	0270	0206	273	689	260	4	689	48	655	260								0270
Cj_OXC6532	0457	0206	273	689	260	5	689	48	671	655	260							0457
Cj_OXC6661	0532	0206	273	689	260	5	689	48	671	655	260							0532
Cj_OXC5373	0884	0206	273	689	260	5	689	48	671	655	260							0884
Cj_OXC7056	1230	0206	273	689	260	5	689	48	671	655	260							1230
Cj_OXC7100	1267	0206	273	689	260	5	689	48	671	655	260							1267
Cj_OXC7310	1330	0206	273	689	260	5	689	48	671	655	260							1330
Cj_OXC7588	2099	0206	273	689	260	5	689	48	671	655	260							2099
Cj_OXC7800	2167	0206	273	689	260	5	689	48	671	655	260							2167
Cj_OXC7668	2411	0206	273	689	260	5	689	48	671	655	260							2411
Cj_OXC7724	2448	0206	273	689	260	5	689	48	671	655	260							2448
Cj_OXC7903	2505	0206	273	689	260	5	689	48	671	655	260							2505
Cj_OXC5321	2559	0206	273	689	260	5	689	48	671	655	260							2559
Cj_OXC8006	2715	0206	273	689	260	5	689	48	671	655	260							2715
Cj_OXC8050	2805	0206	273	689	260	5	689	48	671	655	260							2805
Cj_OXC8378	2975	0206	273	689	97	6	689	48	671	655	260	97						2975
Cj_OXC8415	3187	0206	273	689	97	6	689	48	671	655	260	97						3187
Cj_OXC8754	3563	0206	273	689	97	6	689	48	671	655	260	97						3563
Cj_OXC8945	3725	0206	273	689	260	5	689	48	671	655	260							3725
Cj_OXC6470	0402	0206	572	10	818	3	10	743	818									0402
Cj_OXC8090	2744	0206	572	10	818	3	10	743	818									2744
Cj_OXC8225	3018	0206	572	10	818	3	10	426	818									3018
Cj_OXC6719	0586	0206	572	578	353	5	578	950	57	559	353							0586
Cj_OXC6720	0587	0206	572	578	353	5	578	950	57	559	353							0587
Cj_OXC7109	0805	0206	572	578	353	5	578	950	57	559	353							0805
Cj_OXC6891	1085	0206	572	578	353	5	578	950	57	559	353							1085
Cj_OXC7576	2091	0206	572	578	229	5	578	950	57	559	229							2091
Cj_OXC7578	2093	0206	572	578	229	5	578	950	57	559	229							2093
Cj_OXC5029	2244	0206	572	578	874	11	578	57	211	891	693	646	699	864	604	347	874	2244
Cj_OXC6580	0207	0206	572	678	902	4	678	645	216	902								0207
Cj_OXC7161	0851	0206	572	678	902	4	678	645	216	902								0851
Cj_OXC7971	2620	0206	572	678	902	4	678	645	216	902								2620
Cj_OXC4724	0693	0206	2078	689	260	5	689	48	671	655	260							0693
Cj_OXC4756	0714	0206	2078	689	260	5	689	48	671	655	260							0714
Cj_OXC8871	3661	0206	2149	48	260	4	48	671	655	260								3661
Cj_OXC8027	2721	0206	2149	743	597	4	743	487	860	597								2721
Cj_OXC6723	0590	0206	5819	689	260	5	689	48	671	655	260							0590
Cj_OXC5884	1656	0206	5819	689	260	5	689	48	671	655	260							1656
Cj_OXC8078	2739	0206	6893	689	260	5	689	48	671	655	260							2739
Cj_H142840351	0110	0257	257	22	759	4	22	383	103	759								0110
Cj_H143140404	0113	0257	257	22	759	4	22	383	103	759								0113
Cj_H143140405	0114	0257	257	22	820	5	22	383	103	759	820							0114
Cj_H143140406	0115	0257	257	22	683	3	22	383	683									0115
Cj_H143140408	0116	0257	257	22	820	5	22	383	103	759	820							0116
Cj_H143140410	0117	0257	257	22	759	4	22	383	103	759								0117
Cj_OXC6583	0210	0257	257	22	229	8	22	383	103	274	759	820	44	229				0210
Cj_OXC6250	0222	0257	257	22	820	5	22	383	274	759	820							0222
Cj_OXC6254	0225	0257	257	22	820	5	22	383	103	759	820							0225
Cj_OXC6290	0255	0257	257	22	741	5	22	22	103	274	741							0255
Cj_OXC6344	0301	0257	257	22	820	6	22	383	103	274	759	820						0301
Cj_OXC6362	0319	0257	257	22	820	5	22	383	103	759	820							0319
Cj_OXC6390	0340	0257	257	22	820	6	22	383	103	274	759	820						0340
Cj_OXC6401	0347	0257	257	22	233	7	22	383	103	274	759	820	233					0347
Cj_OXC6412	0358	0257	257	22	741	4	22	383	103	741								0358
Cj_OXC6450	0384	0257	257	22	29	8	22	383	103	274	759	820	736	29				0384
Cj_OXC6491	0419	0257	257	22	741	3	22	383	741									0419
Cj_OXC6612	0487	0257	257	22	741	3	22	383	741									0487
Cj_OXC6650	0523	0257	257	22	741	3	22	383	741									0523
Cj_OXC6654	0526	0257	257	22	86	8	22	383	103	274	759	820	6	86				0526
Cj_OXC6696	0565	0257	257	22	741	4	22	383	103	741								0565
Cj_OXC6715	0582	0257	257	22	820	5	22	383	103	759	820							0582
Cj_OXC6724	0591	0257	257	22	820	4	22	274	759	820								0591
Cj_OXC6736	0601	0257	257	22	820	5	22	383	103	759	820							0601
Cj_OXC6760	0623	0257	257	22	86	8	22	383	103	274	759	820	6	86				0623
Cj_OXC6816	0674	0257	257	22	234	7	22	383	103	831	160	158	234					0674
Cj_OXC4740	0703	0257	257	22	741	5	22	383	103	274	741							0703
Cj_OXC4774	0723	0257	257	22	234	6	22	383	831	160	158	234						0723
Cj_OXC6967	0761	0257	257	22	160	4	22	383	683	160								0761
Cj_OXC6974	0767	0257	257	22	160	4	22	383	683	160								0767
Cj_OXC6975	0768	0257	257	22	741	4	22	383	103	741								0768
Cj_OXC6982	0775	0257	257	22	192	5	22	103	831	160	192							0775
Cj_OXC6999	0787	0257	257	22	234	7	22	383	103	831	160	158	234					0787
Cj_OXC7120	0815	0257	257	22	820	5	22	103	274	759	820							0815
Cj_OXC7146	0837	0257	257	22	820	3	22	759	820									0837
Cj_OXC5395	0897	0257	257	22	741	3	22	383	741									0897
Cj_OXC5410	0906	0257	257	22	759	5	22	383	103	274	759							0906
Cj_OXC5421	0915	0257	257	22	759	5	22	383	103	274	759							0915
Cj_OXC5703	0978	0257	257	22	201	7	22	383	103	274	741	457	201					0978
Cj_OXC5779	1029	0257	257	22	201	7	22	383	103	274	741	457	201					1029
Cj_OXC5781	1031	0257	257	22	820	6	22	383	103	274	759	820						1031
Cj_OXC6863	1060	0257	257	22	160	4	22	383	683	160								1060
Cj_OXC6880	1075	0257	257	22	741	4	22	383	103	741								1075
Cj_OXC4608	1125	0257	257	22	820	6	22	383	103	274	759	820						1125
Cj_OXC4822	1147	0257	257	22	234	6	22	383	831	160	158	234						1147
Cj_OXC4858	1163	0257	257	22	234	6	22	383	274	741	158	234						1163
Cj_OXC4878	1175	0257	257	22	234	7	22	383	103	831	160	158	234					1175
Cj_OXC7034	1211	0257	257	22	160	4	22	383	683	160								1211
Cj_OXC7096	1264	0257	257	22	820	5	22	103	274	759	820							1264
Cj_OXC7257	1280	0257	257	22	741	4	22	383	103	741								1280
Cj_OXC7291	1312	0257	257	22	741	4	22	383	103	741								1312
Cj_OXC7296	1317	0257	257	22	741	4	22	383	103	741								1317
Cj_OXC7308	1328	0257	257	22	728	6	22	383	103	274	759	728						1328
Cj_OXC7338	1353	0257	257	22	728	6	22	383	103	274	759	728						1353
Cj_OXC7196	1383	0257	257	22	820	6	22	383	103	274	759	820						1383
Cj_OXC5497	1554	0257	257	22	820	6	22	383	103	274	759	820						1554
Cj_OXC5643	1581	0257	257	22	201	7	22	383	103	274	741	457	201					1581
Cj_OXC5788	1593	0257	257	22	820	6	22	383	103	274	759	820						1593
Cj_OXC5817	1616	0257	257	22	201	7	22	383	103	274	741	457	201					1616
Cj_OXC5866	1646	0257	257	22	820	5	22	383	103	759	820							1646
Cj_OXC5872	1648	0257	257	22	820	6	22	383	103	274	759	820						1648
Cj_OXC5885	1657	0257	257	22	160	4	22	103	831	160								1657
Cj_OXC5903	1670	0257	257	22	820	6	22	383	103	274	759	820						1670
Cj_OXC5907	1673	0257	257	22	820	5	22	383	103	759	820							1673
Cj_OXC5915	1680	0257	257	22	201	7	22	383	103	274	741	457	201					1680
Cj_OXC7354	1730	0257	257	22	741	4	22	383	103	741								1730
Cj_OXC7357	1733	0257	257	22	201	7	22	383	103	274	741	457	201					1733
Cj_OXC7410	1777	0257	257	22	233	7	22	383	103	274	759	820	233					1777
Cj_OXC7424	1789	0257	257	22	86	8	22	383	103	274	759	820	6	86				1789
Cj_OXC7507	1854	0257	257	22	234	7	22	383	103	831	160	158	234					1854
Cj_OXC4593	1882	0257	257	22	234	7	22	383	103	831	160	158	234					1882
Cj_OXC4620	1891	0257	257	22	820	6	22	383	103	274	759	820						1891
Cj_OXC4678	1930	0257	257	22	234	7	22	383	103	831	160	158	234					1930
Cj_CJ7257_R	2041	0257	257	22	741	4	22	383	103	741								2041
Cj_OXC7796	2163	0257	257	22	741	4	22	383	103	741								2163
Cj_OXC7826	2185	0257	257	22	820	6	22	383	103	274	759	820						2185
Cj_OXC7844	2199	0257	257	22	741	4	22	383	103	741								2199
Cj_OXC7850	2205	0257	257	22	234	7	22	383	103	831	160	158	234					2205
Cj_OXC5001	2225	0257	257	22	741	3	22	383	741									2225
Cj_OXC5022	2238	0257	257	22	234	7	22	383	103	831	160	158	234					2238
Cj_OXC5023	2239	0257	257	22	234	7	22	383	103	831	160	158	234					2239
Cj_OXC5030	2245	0257	257	22	820	6	22	383	103	274	759	820						2245

Cj_OXC5122	2292	0257	257	22	820	6	22	383	103	274	759	820			2292
Cj_OXC5187	2328	0257	257	22	728	7	22	383	103	274	759	820	728		2328
Cj_OXC5209	2344	0257	257	22	820	6	22	383	103	274	759	820			2344
Cj_OXC7655	2398	0257	257	22	234	7	22	383	103	831	160	158	234		2398
Cj_OXC4672	2547	0257	257	22	234	6	22	383	831	160	158	234			2547
Cj_OXC4923	2548	0257	257	22	741	3	22	383	741						2548
Cj_OXC4956	2554	0257	257	22	201	7	22	383	103	274	741	457	201		2554
Cj_OXC7974	2623	0257	257	22	234	7	22	383	103	831	160	158	234		2623
Cj_OXC7962	2705	0257	257	22	234	7	22	383	103	831	160	158	234		2705
Cj_OXC4963	2777	0257	257	22	234	7	22	383	103	831	160	158	234		2777
Cj_OXC8371	2970	0257	257	22	86	8	22	383	103	274	759	820	6	86	2970
Cj_OXC8217	3013	0257	257	22	820	5	22	383	103	759	820				3013
Cj_OXC8370	3136	0257	257	22	820	6	22	383	103	274	759	820			3136
Cj_OXC8429	3197	0257	257	22	628	7	22	383	103	274	759	820	628		3197
Cj_OXC8537	3295	0257	257	22	741	3	22	383	741						3295
Cj_C120401	3327	0257	257	22	741	5	22	22	103	274	741				3327
Cj_C120553	3331	0257	257	22	741	4	22	103	274	741					3331
Cj_OXC6170	3528	0257	257	22	820	5	22	383	103	759	820				3528
Cj_OXC6183	3532	0257	257	22	265	4	22	383	683	265					3532
Cj_OXC8786	3592	0257	257	22	233	7	22	383	103	274	759	820	233		3592
Cj_OXC8866	3656	0257	257	22	86	8	22	383	103	274	759	820	6	86	3656
Cj_OXC8909	3692	0257	257	22	820	5	22	383	103	759	820				3692
Cj_OXC8979	3744	0257	257	22	234	7	22	383	103	831	160	158	234		3744
Cj_OXC9000	3760	0257	257	22	759	4	22	383	103	759					3760
Cj_OXC9001	3761	0257	257	22	759	4	22	383	103	759					3761
Cj_C120453	3329	0257	257	103	741	3	103	274	741						3329
Cj_OXC5390	0893	0257	257	107	688	3	107	843	688						0893
Cj_OXC7965	2786	0257	257	107	688	3	107	843	688						2786
Cj_OXC6407	0353	0257	257	261	136	5	261	229	9	246	136				0353
Cj_OXC4760	0715	0257	257	261	9	3	261	229	9						0715
Cj_OXC7761	2139	0257	257	261	9	3	261	229	9						2139
Cj_OXC8083	2652	0257	257	261	136	5	261	229	9	246	136				2652
Cj_OXC8089	2656	0257	257	261	136	5	261	229	9	246	136				2656
Cj_OXC8256	3041	0257	257	261	136	5	261	229	9	246	136				3041
Cj_OXC7106	0802	0257	257	681	736	4	681	200	463	736					0802
Cj_OXC8772	3578	0257	286	107	688	3	107	843	688						3578
Cj_OXC5235	2362	0257	929	333	537	3	333	925	537						2362
Cj_OXC8757	3565	0257	990	843	377	3	843	3	377						3565
Cj_OXC6582	0209	0257	2030	22	201	7	22	383	103	274	741	457	201		0209
Cj_OXC6291	0256	0257	2030	22	201	7	22	383	103	274	741	457	201		0256
Cj_OXC6404	0350	0257	2030	22	201	7	22	383	103	274	741	457	201		0350
Cj_OXC6467	0399	0257	2030	22	201	7	22	383	103	274	741	457	201		0399
Cj_OXC6469	0401	0257	2030	22	201	7	22	383	103	274	741	457	201		0401
Cj_OXC6499	0427	0257	2030	22	201	7	22	383	103	274	741	457	201		0427
Cj_OXC6709	0577	0257	2030	22	201	7	22	383	103	274	741	457	201		0577
Cj_OXC6749	0612	0257	2030	22	201	7	22	383	103	274	741	457	201		0612
Cj_OXC6783	0643	0257	2030	22	201	7	22	383	103	274	741	457	201		0643
Cj_OXC6784	0644	0257	2030	22	201	7	22	383	103	274	741	457	201		0644
Cj_OXC6792	0651	0257	2030	22	201	7	22	383	103	274	741	457	201		0651
Cj_OXC4738	0702	0257	2030	22	201	7	22	383	103	274	741	457	201		0702
Cj_OXC6989	0780	0257	2030	22	201	7	22	383	103	274	741	457	201		0780
Cj_OXC5709	0981	0257	2030	22	201	7	22	383	103	274	741	457	201		0981
Cj_OXC4869	1170	0257	2030	22	201	7	22	383	103	274	741	457	201		1170
Cj_OXC7245	1269	0257	2030	22	201	7	22	383	103	274	741	457	201		1269
Cj_OXC5641	1579	0257	2030	22	201	7	22	383	103	274	741	457	201		1579
Cj_OXC5859	1642	0257	2030	22	201	7	22	383	103	274	741	457	201		1642
Cj_OXC5865	1645	0257	2030	22	201	7	22	383	103	274	741	457	201		1645
Cj_OXC5869	1647	0257	2030	22	201	7	22	383	103	274	741	457	201		1647
Cj_OXC5889	1661	0257	2030	22	201	7	22	383	103	274	741	457	201		1661
Cj_OXC5932	1691	0257	2030	22	201	7	22	383	103	274	741	457	201		1691
Cj_OXC5794	1697	0257	2030	22	201	7	22	383	103	274	741	457	201		1697
Cj_OXC7390	1763	0257	2030	22	201	7	22	383	103	274	741	457	201		1763
Cj_HM115713	1975	0257	2030	22	201	7	22	383	103	274	741	457	201		1975
Cj_OXC7566	2083	0257	2030	22	201	7	22	383	103	274	741	457	201		2083
Cj_OXC7585	2096	0257	2030	22	201	7	22	383	103	274	741	457	201		2096
Cj_OXC7604	2110	0257	2030	22	201	7	22	383	103	274	741	457	201		2110
Cj_OXC7756	2137	0257	2030	22	201	7	22	383	103	274	741	457	201		2137
Cj_OXC7767	2143	0257	2030	22	201	7	22	383	103	274	741	457	201		2143
Cj_OXC7792	2161	0257	2030	22	201	7	22	383	103	274	741	457	201		2161
Cj_OXC5000	2224	0257	2030	22	201	7	22	383	103	274	741	457	201		2224
Cj_OXC5009	2230	0257	2030	22	201	7	22	383	103	274	741	457	201		2230
Cj_OXC5015	2235	0257	2030	22	201	7	22	383	103	274	741	457	201		2235
Cj_OXC5025	2240	0257	2030	22	201	7	22	383	103	274	741	457	201		2240
Cj_OXC5027	2242	0257	2030	22	201	7	22	383	103	274	741	457	201		2242
Cj_OXC5041	2253	0257	2030	22	201	7	22	383	103	274	741	457	201		2253
Cj_OXC5052	2260	0257	2030	22	201	7	22	383	103	274	741	457	201		2260
Cj_OXC5054	2261	0257	2030	22	201	7	22	383	103	274	741	457	201		2261
Cj_OXC5109	2284	0257	2030	22	201	7	22	383	103	274	741	457	201		2284
Cj_OXC7707	2437	0257	2030	22	201	7	22	383	103	274	741	457	201		2437
Cj_OXC7738	2456	0257	2030	22	201	7	22	383	103	274	741	457	201		2456
Cj_OXC7875	2486	0257	2030	22	201	7	22	383	103	274	741	457	201		2486
Cj_OXC7909	2508	0257	2030	22	201	7	22	383	103	274	741	457	201		2508
Cj_OXC4945	2552	0257	2030	22	201	7	22	383	103	274	741	457	201		2552
Cj_OXC5315	2556	0257	2030	22	201	7	22	383	103	274	741	457	201		2556
Cj_OXC7878	2571	0257	2030	22	201	7	22	383	103	274	741	457	201		2571
Cj_OXC8030	2723	0257	2030	22	201	7	22	383	103	274	741	457	201		2723
Cj_OXC8112	2821	0257	2030	22	201	7	22	383	103	274	741	457	201		2821
Cj_OXC4637	2848	0257	2030	22	201	7	22	383	103	274	741	457	201		2848
Cj_OXC7754	2969	0257	2030	22	201	7	22	383	103	274	741	457	201		2969
Cj_OXC8465	3229	0257	2030	22	201	6	22	383	274	741	457	201			3229
Cj_OXC8544	3300	0257	2030	22	201	6	22	383	274	741	457	201			3300
Cj_OXC8613	3379	0257	2030	22	201	6	22	383	274	741	457	201			3379
Cj_OXC8672	3435	0257	2030	22	201	6	22	383	274	741	457	201			3435
Cj_OXC8445	3211	0257	2254	261	9	3	261	229	9						3211
Cj_OXC8477	3241	0257	2254	261	9	3	261	229	9						3241
Cj_OXC8683	3444	0257	2254	261	9	3	261	229	9						3444
Cj_OXC8797	3601	0257	2254	261	9	3	261	229	9						3601
Cj_OXC5833	1702	0257	6603	22	201	7	22	383	103	274	741	457	201		1702
Cj_OXC7889	2494	0257	6890	22	741	5	22	383	103	274	741				2494
Cj_OXC8000	2789	0257	6890	22	741	5	22	383	103	274	741				2789
Cj_81116	0047	0283	267	14	566	7	14	585	20	823	7	21	566		0047
Cj_OXC6510	0437	0283	267	14	566	6	14	20	823	7	21	566			0437
Cj_OXC6511	0438	0283	267	14	566	6	14	20	823	7	21	566			0438
Cj_OXC4778	0727	0283	267	14	566	6	14	20	823	7	21	566			0727
Cj_OXC7144	0835	0283	267	14	566	6	14	20	823	7	21	566			0835
Cj_OXC7152	0843	0283	267	14	566	6	14	20	823	7	21	566			0843
Cj_OXC7155	0845	0283	267	14	7	4	14	20	823	7					0845
Cj_OXC6884	1079	0283	267	14	566	6	14	20	823	7	21	566			1079
Cj_OXC7030	1207	0283	267	14	566	6	14	20	823	7	21	566			1207
Cj_OXC7053	1228	0283	267	14	566	6	14	20	823	7	21	566			1228
Cj_OXC7060	1233	0283	267	14	566	6	14	20	823	7	21	566			1233
Cj_OXC7086	1256	0283	267	14	566	6	14	20	823	7	21	566			1256
Cj_OXC7278	1299	0283	267	14	566	6	14	20	823	7	21	566			1299
Cj_OXC7322	1339	0283	267	14	566	6	14	20	823	7	21	566			1339
Cj_OXC7198	1385	0283	267	14	7	4	14	20	823	7					1385
Cj_OXC7234	1415	0283	267	14	566	4	14	823	7	566					1415
Cj_OXC7391	1764	0283	267	14	566	6	14	20	823	7	21	566			1764
Cj_OXC7392	1765	0283	267	14	566	6	14	20	823	7	21	566			1765
Cj_OXC7393	1766	0283	267	14	566	6	14	20	823	7	21	566			1766
Cj_OXC7432	1795	0283	267	14	754	4	14	20	21	754					1795
Cj_OXC4683	1934	0283	267	14	566	6	14	20	823	7	21	566			1934
Cj_OXC4933	2054	0283	267	14	566	6	14	20	823	7	21	566			2054

Cj_OXC7551	2071	0283	267	14	566	6	14	20	823	7	21	566					2071
Cj_OXC7829	2188	0283	267	14	631	6	14	585	823	21	566	631					2188
Cj_OXC5011	2232	0283	267	14	566	6	14	20	823	7	21	566					2232
Cj_OXC5014	2234	0283	267	14	566	6	14	20	823	7	21	566					2234
Cj_OXC7963	2613	0283	267	14	566	6	14	20	824	7	21	566					2613
Cj_OXC8135	2845	0283	267	14	566	6	14	20	823	7	21	566					2845
Cj_Dg66a	2871	0283	267	14	566	6	14	20	823	7	21	566					2871
Cj_Dg198	2963	0283	267	14	566	6	14	20	823	7	21	566					2963
Cj_OXC8136	2983	0283	267	14	566	6	14	20	823	7	21	566					2983
Cj_OXC8137	2984	0283	267	14	566	6	14	20	823	7	21	566					2984
Cj_OXC8292	3067	0283	267	14	566	5	14	20	823	7	566						3067
Cj_OXC8947	3727	0283	267	14	566	6	14	20	823	7	21	566					3727
Cj_OXC8988	3750	0283	267	14	7	4	14	20	823	7							3750
Cj_OXC8991	3753	0283	267	14	7	4	14	20	823	7							3753
Cj_OXC9003	3763	0283	267	14	7	4	14	20	823	7							3763
Cj_OXC9007	3765	0283	267	14	7	4	14	20	823	7							3765
Cj_OXC9008	3766	0283	267	14	7	4	14	20	823	7							3766
Cj_OXC9012	3770	0283	267	14	754	4	14	20	21	754							3770
Cj_OXC9013	3771	0283	267	14	7	4	14	20	823	7							3771
Cj_OXC6369	0326	0283	267	20	102	7	20	823	7	21	566	235	102				0326
Cj_OXC7255	1278	0283	6234	14	257	5	14	20	823	7	257						1278
Cj_OXC7586	2097	0353	5	261	9	3	261	229	9								2097
Cj_OXC4573	1869	0353	5	828	736	4	828	874	559	736							1869
Cj_OXC7830	2189	0353	353	261	46	5	261	753	49	773	46						2189
Cj_OXC8524	3283	0353	353	322	392	3	322	669	392								3283
Cj_OXC7722	2447	0353	353	743	206	4	743	487	253	206							2447
Cj_OXC7630	2127	0353	353	753	517	5	753	753	49	506	517						2127
Cj_OXC6611	0486	0353	353	834	348	4	834	169	483	348							0486
Cj_OXC6658	0530	0353	353	834	348	4	834	169	483	348							0530
Cj_OXC6318	0278	0353	356	10	694	3	10	30	694								0278
Cj_OXC6704	0573	0353	356	10	694	3	10	30	694								0573
Cj_OXC5750	1008	0353	400	221	507	5	221	318	495	176	507						1008
Cj_OXC7226	1409	0353	400	221	92	8	221	318	495	659	171	908	409	92			1409
Cj_OXC7231	1412	0353	400	221	92	8	221	318	495	659	171	908	409	92			1412
Cj_OXC6586	0213	0353	400	712	7	4	712	221	390	7							0213
Cj_OXC5830	1626	0353	400	712	955	4	712	221	927	955							1626
Cj_OXC8649	3399	0353	400	712	858	5	712	221	41	372	858						3399
Cj_OXC8745	3559	0353	400	712	858	5	712	221	41	372	858						3559
Cj_OXC8808	3609	0353	400	712	858	5	712	221	41	372	858						3609
Cj_OXC6622	0496	0353	595	755	174	2	755	174									0496
Cj_OXC5071	2270	0353	1474	728	104	2	728	104									2270
Cj_OXC5070	2695	0353	1474	728	104	2	728	104									2695
Cj_OXC7271	1293	0353	2036	323	298	5	323	641	19	78	298						1293
Cj_OXC8146	2832	0353	2036	428	602	2	428	602									2832
Cj_OXC5633	1573	0353	2076	7	818	5	7	28	29	144	818						1573
Cj_OXC5934	1693	0353	2084	588	922	4	588	509	648	922							1693
Cj_OXC4719	0690	0353	2122	53	584	10	53	334	144	926	818	920	438	842	423	584	0690
Cj_OXC5480	0951	0353	2122	53	818	5	53	334	144	926	818						0951
Cj_OXC5786	1591	0353	2122	53	818	5	53	334	144	926	818						1591
Cj_OXC8249	3035	0353	2122	53	728	3	53	818	728								3035
Cj_OXC8722	3470	0353	2122	53	818	4	53	144	926	818							3470
Cj_OXC8898	3682	0353	2122	53	17	5	53	144	926	818	17						3682
Cj_OXC8895	3774	0353	2122	53	17	5	53	144	926	818	17						3774
Cj_OXC8898R	3775	0353	2122	53	17	5	53	144	926	818	17						3775
Cj_OXC5299	2387	0353	2882	426	242	4	426	468	901	242							2387
Cj_OXC4634	1902	0353	3515	775	134	3	775	490	134								1902
Cj_OXC8723	3471	0353	5011	322	397	4	322	107	794	397							3471
Cj_OXC6455	0388	0353	5730	7	818	6	7	28	29	144	926	818					0388
Cj_OXC6776	0636	0353	5815	878	916	3	878	561	916								0636
Cj_OXC7661	2404	0353	6888	355	564	4	355	363	86	564							2404
Cj_OXC8121	2757	0353	6981	318	937	3	318	495	937								2757
Cj_OXC8802	3606	0354	354	9	561	8	9	387	288	8	773	753	49	561			3606
Cj_NW	0096	0354	354	183	615	5	183	889	678	645	615						0096
Cj_OXC5262	2379	0354	354	183	426	7	183	889	678	645	138	854	426				2379
Cj_OXC5080	2271	0354	354	678	842	3	678	645	842								2271
Cj_H140940808	0148	0354	354	703	799	4	703	889	715	799							0148
Cj_OXC6567	0197	0354	354	703	799	4	703	889	715	799							0197
Cj_OXC6298	0261	0354	354	703	799	4	703	889	715	799							0261
Cj_OXC6299	0262	0354	354	703	799	4	703	889	715	799							0262
Cj_OXC6418	0363	0354	354	703	240	4	703	889	715	240							0363
Cj_OXC6451	0385	0354	354	703	799	4	703	889	715	799							0385
Cj_OXC6517	0443	0354	354	703	240	3	703	889	240								0443
Cj_OXC6518	0444	0354	354	703	240	4	703	889	715	240							0444
Cj_OXC6529	0454	0354	354	703	240	4	703	889	715	240							0454
Cj_OXC6647	0520	0354	354	703	799	4	703	889	715	799							0520
Cj_OXC6698	0567	0354	354	703	240	4	703	889	715	240							0567
Cj_OXC6716	0583	0354	354	703	799	4	703	889	715	799							0583
Cj_OXC6717	0584	0354	354	703	799	4	703	889	715	799							0584
Cj_OXC6743	0607	0354	354	703	799	4	703	889	715	799							0607
Cj_OXC6753	0616	0354	354	703	799	4	703	889	715	799							0616
Cj_OXC6801	0660	0354	354	703	240	4	703	889	715	240							0660
Cj_OXC4727	0695	0354	354	703	240	4	703	889	715	240							0695
Cj_OXC4741	0704	0354	354	703	240	4	703	889	715	240							0704
Cj_OXC4743	0706	0354	354	703	240	4	703	889	715	240							0706
Cj_OXC5385	0892	0354	354	703	240	4	703	889	715	240							0892
Cj_OXC5400	0901	0354	354	703	799	3	703	715	799								0901
Cj_OXC5466	0943	0354	354	703	799	4	703	889	715	799							0943
Cj_OXC5475	0947	0354	354	703	240	3	703	715	240								0947
Cj_OXC5673	0960	0354	354	703	240	4	703	889	715	240							0960
Cj_OXC5695	0972	0354	354	703	799	3	703	715	799								0972
Cj_OXC5700	0975	0354	354	703	799	4	703	889	715	799							0975
Cj_OXC5741	1002	0354	354	703	799	3	703	715	799								1002
Cj_OXC6877	1072	0354	354	703	240	4	703	889	715	240							1072
Cj_OXC6885	1080	0354	354	703	240	4	703	889	715	240							1080
Cj_OXC6915	1108	0354	354	703	799	4	703	889	715	799							1108
Cj_OXC4857	1162	0354	354	703	799	4	703	889	715	799							1162
Cj_OXC7274	1295	0354	354	703	799	4	703	889	715	799							1295
Cj_OXC7204	1389	0354	354	703	240	3	703	715	240								1389
Cj_OXC7408	1775	0354	354	703	240	3	703	715	240								1775
Cj_OXC7409	1776	0354	354	703	240	3	703	715	240								1776
Cj_OXC7414	1779	0354	354	703	240	4	703	889	715	240							1779
Cj_OXC7439	1799	0354	354	703	240	4	703	889	715	240							1799
Cj_OXC7471	1825	0354	354	703	240	4	703	889	715	240							1825
Cj_OXC4596	1884	0354	354	703	240	4	703	889	715	240							1884
Cj_OXC4655	1914	0354	354	703	240	4	703	889	715	240							1914
Cj_OXC4656	1915	0354	354	703	799	4	703	889	715	799							1915
Cj_CJ6753_R	2006	0354	354	703	799	4	703	889	715	799							2006
Cj_OXC7583	2094	0354	354	703	240	4	703	889	715	240							2094
Cj_OXC7594	2102	0354	354	703	799	3	703	715	799								2102
Cj_OXC7628	2126	0354	354	703	240	4	703	889	715	240							2126
Cj_OXC7819	2180	0354	354	703	240	4	703	889	715	240							2180
Cj_OXC7843	2198	0354	354	703	799	4	703	889	715	799							2198
Cj_OXC7849	2204	0354	354	703	799	4	703	889	715	799							2204
Cj_OXC4970	2209	0354	354	703	799	4	703	889	715	799							2209
Cj_OXC4971	2210	0354	354	703	799	4	703	889	715	799							2210
Cj_OXC5087	2275	0354	354	703	240	4	703	889	715	240							2275
Cj_OXC5128	2297	0354	354	703	799	3	703	715	799								2297
Cj_OXC5195	2335	0354	354	703	240	4	703	889	715	240							2335
Cj_OXC5206	2341	0354	354	703	799	4	703	889	715	799							2341
Cj_OXC5207	2342	0354	354	703	799	4	703	889	715	799							2342
Cj_OXC5243	2368	0354	354	703	240	4	703	889	715	240							2368
Cj_OXC5282	2382	0354	354	703	799	4	703	889	715	799							2382

Cj_OXC7742	2458	0354	354	703	799	4	703	889	715	799														2458
Cj_OXC7916	2514	0354	354	703	240	4	703	889	715	240														2514
Cj_OXC7917	2515	0354	354	703	240	4	703	889	715	240														2515
Cj_OXC7928	2523	0354	354	703	799	4	703	889	715	799														2523
Cj_OXC7970	2619	0354	354	703	240	4	703	889	715	240														2619
Cj_OXC7978	2627	0354	354	703	240	4	703	889	715	240														2627
Cj_OXC8084	2653	0354	354	703	240	4	703	889	715	240														2653
Cj_OXC8095	2658	0354	354	703	240	4	703	889	715	240														2658
Cj_OXC8126	2685	0354	354	703	240	4	703	889	715	240														2685
Cj_OXC8013	2794	0354	354	703	799	4	703	889	715	799														2794
Cj_OXC8049	2804	0354	354	703	799	4	703	889	715	799														2804
Cj_OXC8188	2990	0354	354	703	799	4	703	889	715	799														2990
Cj_OXC8189	2991	0354	354	703	240	4	703	889	715	240														2991
Cj_OXC8204	3004	0354	354	703	240	4	703	889	715	240														3004
Cj_OXC8276	3053	0354	354	703	240	4	703	889	715	240														3053
Cj_OXC8281	3057	0354	354	703	240	4	703	889	715	240														3057
Cj_OXC8160	3143	0354	354	703	799	3	703	715	799															3143
Cj_OXC8387	3163	0354	354	703	240	4	703	889	715	240														3163
Cj_OXC8467	3231	0354	354	703	799	4	703	889	715	799														3231
Cj_OXC8495	3257	0354	354	703	799	4	703	889	715	799														3257
Cj_OXC8508	3269	0354	354	703	240	4	703	889	715	240														3269
Cj_OXC8546	3302	0354	354	703	799	4	703	889	715	799														3302
Cj_P140074	3320	0354	354	703	240	4	703	889	715	240														3320
Cj_C131405	3322	0354	354	703	799	4	703	889	715	799														3322
Cj_C130436	3324	0354	354	703	240	4	703	889	715	240														3324
Cj_C121803	3325	0354	354	703	240	4	703	889	715	240														3325
Cj_OXC8562	3344	0354	354	703	799	4	703	889	715	799														3344
Cj_OXC8618	3383	0354	354	703	799	4	703	889	715	799														3383
Cj_OXC8619	3421	0354	354	703	799	4	703	889	715	799														3421
Cj_OXC8660	3427	0354	354	703	240	4	703	889	715	240														3427
Cj_OXC6132	3518	0354	354	703	240	4	703	889	715	240														3518
Cj_OXC8781	3587	0354	354	703	799	4	703	889	715	799														3587
Cj_OXC8877	3666	0354	354	703	240	4	703	889	715	240														3666
Cj_OXC8881	3670	0354	354	703	799	3	703	889	799															3670
Cj_OXC8883	3671	0354	354	703	240	3	703	889	240															3671
Cj_OXC8764	3773	0354	354	703	240	4	703	889	715	240														3773
Cj_OXC7765	2141	0354	354	889	559	5	889	360	807	329	559													2141
Cj_OXC8088	2743	0354	354	889	319	6	889	678	546	547	310	319												2743
Cj_OXC6846	1044	0354	969	889	7	3	889	678	7															1044
Cj_BID17Q	2532	0354	1038	775	735	5	775	508	346	594	735													2532
Cj_OXC6608	0483	0354	1073	490	772	5	490	413	809	621	772													0483
Cj_OXC6729	0595	0354	1517	406	549	6	406	889	944	351	468	549												0595
Cj_OXC7664	2407	0354	1517	406	549	6	406	889	944	351	468	549												2407
Cj_OXC6934	0730	0354	2033	703	799	4	703	889	715	799														0730
Cj_OXC5627	0952	0354	2033	703	240	4	703	889	715	240														0952
Cj_OXC5604	1557	0354	2033	703	799	4	703	889	715	799														1557
Cj_OXC5797	1599	0354	2033	703	799	3	703	889	799															1599
Cj_OXC5847	1636	0354	2033	703	799	4	703	889	715	799														1636
Cj_OXC5927	1687	0354	2033	703	799	4	703	889	715	799														1687
Cj_OXC4598	1885	0354	2033	703	799	4	703	889	715	799														1885
Cj_OXC4641	1907	0354	2033	703	799	4	703	889	715	799														1907
Cj_ARI_1433_R	2013	0354	2033	703	240	4	703	889	715	240														2013
Cj_OXC6802	0661	0354	2863	224	560	6	224	7	235	67	455	560												0661
Cj_OXC8356	3126	0354	2863	224	560	5	224	7	235	455	560													3126
Cj_C131690	3321	0354	3155	703	799	4	703	889	715	799														3321
Cj_C131242	3323	0354	3155	703	799	4	703	889	715	799														3323
Cj_OXC8828	3623	0354	3155	703	240	4	703	889	715	240														3623
Cj_OXC6287	0252	0354	5718	703	240	4	703	889	715	240														0252
Cj_UKZN015_R	2042	0354	6464	889	645	3	889	678	645															2042
Cj_IA3902	0071	0362	2993	76	76	5	76	84	610	654	76													0071
Cj_HN-CJD07035	0070	0362	2993	271	76	5	271	84	610	654	76													0070
Cj_ICDCCJ07001	0072	0362	2993	271	76	5	271	84	610	654	76													0072
Cj_OXC6740	0604	0403	403	58	180	9	58	208	164	261	946	287	266	961	180									0604
Cj_OXC5912	1678	0403	403	58	158	3	58	264	158															1678
Cj_OXC7548	2069	0403	403	58	2	9	58	264	450	3	721	60	235	2	2									2069
Cj_OXC5170	2317	0403	403	58	2	7	58	264	450	3	721	60	2											2317
Cj_OXC7555	2563	0403	403	881	107	17	881	707	787	248	322	2	358	170	828	180	274	99	730	961	548	331	107	2563
Cj_OXC8164	2760	0403	403	881	66	11	881	707	787	248	322	2	274	235	261	368	66							2760
Cj_OXC7794	2980	0403	403	881	27	8	881	707	787	248	322	331	27	27										2980
Cj_OXC8616	3381	0403	403	881	27	8	881	707	787	248	322	331	27	27										3381
Cj_OXC6883	1078	0403	985	235	2	5	235	106	740	774	2													1078
Cj_OXC8735	3479	0403	985	235	619	11	235	884	158	9	906	713	73	962	79	753	619							3479
Cj_OXC8838	3632	0403	1775	5	852	7	5	24	541	24	541	598	852											3632
Cj_OXC6869	1065	0403	5246	657	194	4	657	611	70	194														1065
Cj_OXC5893	1712	0403	5246	657	194	4	657	611	70	194														1712
Cj_Dg280	2950	0433	2574	776	431	3	776	823	431															2950
Cj_OXC6649	0522	0443	51	3	636	4	3	65	716	636														0522
Cj_OXC6759	0622	0443	51	3	65	3	3	555	65															0622
Cj_OXC8165	2669	0443	51	3	48	4	3	556	65	48														2669
Cj_OXC8166	2670	0443	51	3	48	4	3	556	65	48														2670
Cj_H121820082	0124	0443	51	703	799	3	703	183	799															0124
Cj_OXC6274	0241	0443	51	703	799	3	703	183	799															0241
Cj_OXC6302	0265	0443	51	703	799	3	703	183	799															0265
Cj_OXC6311	0272	0443	51	703	799	3	703	183	799															0272
Cj_OXC6357	0314	0443	51	703	799	3	703	183	799															0314
Cj_OXC6396	0345	0443	51	703	799	3	703	183	799															0345
Cj_OXC6620	0494	0443	51	703	799	3	703	183	799															0494
Cj_OXC6668	0539	0443	51	703	799	3	703	183	799															0539
Cj_OXC6834	0689	0443	51	703	799	3	703	183	799															0689
Cj_OXC6951	0746	0443	51	703	799	3	703	183	799															0746
Cj_OXC7001	0789	0443	51	703	799	3	703	183	799															0789
Cj_OXC5676	0963	0443	51	703	799	3	703	183	799															0963
Cj_OXC5702	0977	0443	51	703	799	3	703	183	799															0977
Cj_OXC5758	1013	0443	51	703	799	3	703	183	799															1013
Cj_OXC5764	1017	0443	51	703	799	3	703	183	799															1017
Cj_OXC6910	1103	0443	51	703	799	3	703	183	799															1103
Cj_OXC6919	1112	0443	51	703	799	3	703	183	799															1112
Cj_OXC7003	1182	0443	51	703	799	3	703	183	799															1182
Cj_OXC7004	1183	0443	51	703	799	3	703	183	799															1183
Cj_OXC7038	1215	0443	51	703	799	3	703	183	799															1215
Cj_OXC7250	1274	0443	51	703	799	3	703	183	799															1274
Cj_OXC7273	1294	0443	51	703	799	3	703	183	799															1294
Cj_OXC5646	1583	0443	51	703	799	3	703	183	799															1583
Cj_OXC7378	1752	0443	51	703	799	3	703	183	799															1752
Cj_OXC7480	1832	0443	51	703	799	3	703	183	799															1832
Cj_OXC7487	1838	0443	51	703	799	3	703	183	799															1838
Cj_OXC7493	1844	0443	51	703	799	3	703	183	799															1844
Cj_OXC4589	1879	0443	51	703	799	3	703	183	799															1879
Cj_OXC4631	1899	0443	51	703	799	3	703	183	799															1899
Cj_OXC7537	2063	0443	51	703	799	3	703	183	799															2063
Cj_OXC4987	2218	0443	51	703	799	3	703	183	799															2218
Cj_OXC5138	2304	0443	51	703	799	3	703	183	799															2304
Cj_OXC5303	2389	0443	51	703	799	3	703	183	799															2389
Cj_OXC7749	2465	0443	51	703	799	3	703	183	799															2465
Cj_OXC7857	2473	0443	51	703	799	3	703	183	799															2473
Cj_OXC5317	2557	0443	51	703	799	3	703	183	799															2557
Cj_OXC8155	2667	0443	51	703	799	3	703	183	799															2667
Cj_OXC8150	2758	0443	51	703	799	3	703	183	799															2758
Cj_OXC8012	2793	0443	51	703	799	3	703	183	799															2793
Cj_OXC8260	3043	0443	51	703	799	3	703	183	799															3043
Cj_OXC8268	3048	0443	51	703	799	3	703	183	799															3048
Cj_OXC8365	3134	0443	51	703	799	3	703	183	799															3134

Cj_OXC8382	3158	0443	51	703	799	3	703	183	799																	3158
Cj_OXC8435	3202	0443	51	703	799	3	703	183	799																	3202
Cj_OXC8459	3222	0443	51	703	799	3	703	183	799																	3222
Cj_OXC8526	3285	0443	51	703	799	3	703	183	799																	3285
Cj_OXC8534	3292	0443	51	703	799	3	703	183	799																	3292
Cj_OXC8625	3423	0443	51	703	799	3	703	183	799																	3423
Cj_OXC8712	3464	0443	51	703	799	3	703	183	799																	3464
Cj_OXC8924	3706	0443	51	703	799	3	703	183	799																	3706
Cj_OXC8973	3743	0443	51	703	799	3	703	183	799																	3743
Cj_OXC5460	0938	0443	443	3	393	8	3	555	349	559	888	735	404	393												0938
Cj_OXC7339	1354	0443	443	555	429	7	555	446	46	746	454	964	429													1354
Cj_OXC4967	2555	0443	443	555	429	7	555	446	46	746	454	964	429													2555
Cj_OXC7201	1386	0443	1703	703	799	3	703	183	799																	1386
Cj_OXC6481	0409	0443	2361	487	189	4	487	773	697	189																0409
Cj_OXC7450	1807	0443	2361	487	189	4	487	773	697	189																1807
Cj_OXC8682	3443	0443	6522	678	276	3	678	764	276																	3443
Cj_OXC5928	1688	0443	6602	703	799	3	703	183	799																	1688
Cj_OXC8304	3077	0443	6986	282	361	4	282	899	765	361																3077
Cj_OXC6875	1070	0446	446	743	46	6	743	419	5	966	195	46														1070
Cj_OXC7941	2578	0446	450	1	259	6	1	940	911	941	232	259														2578
Cj_OXC7683	2424	0460	460	432	339	3	432	834	339																	2424
Cj_BID1NV	2582	0460	535	853	510	6	853	842	568	700	152	510														2582
Cj_OXC8709	3461	0460	670	294	740	2	294	740																		3461
Cj_OXC7877	2487	0460	1932	139	85	3	139	718	85																	2487
Cj_CJ1	0058	0460	3919	183	175	2	183	175																		0058
Cj_OXC8498	3260	0460	5484	266	837	3	266	93	837																	3260
Cj_OXC4773	0722	0464	464	107	234	5	107	107	334	7	234															0722
Cj_OXC7381	1755	0464	464	107	234	4	107	334	7	234																1755
Cj_OXC5238	2365	0464	464	107	496	4	107	650	39	496																2365
Cj_OXC7931	2525	0464	464	107	234	6	107	334	107	334	227	234														2525
Cj_OXC8488	3251	0464	464	118	107	3	118	468	107																	3251
Cj_OXC8097	2817	0464	464	183	842	4	183	678	645	842																2817
Cj_OXC6839	1037	0464	464	250	497	12	250	112	736	407	844	389	663	388	420	781	59	497								1037
Cj_OXC7446	1804	0464	464	334	234	3	334	7	234																	1804
Cj_OXC8386	3162	0464	464	805	565	5	805	7	128	242	565															3162
Cj_OXC6800	0659	0464	2146	107	913	5	107	645	514	812	913															0659
Cj_OXC7882	2490	0464	2146	107	913	5	107	645	514	812	913															2490
Cj_OXC8656	3403	0464	2146	107	913	4	107	514	812	913																3403
Cj_OXC8657	3404	0464	2146	107	913	4	107	514	812	913																3404
Cj_OXC8687	3448	0464	2146	645	812	3	645	514	812																	3448
Cj_OXC5630	1570	0464	2315	828	745	5	828	874	528	562	745															1570
Cj_OXC5631	1571	0464	2315	828	745	5	828	874	528	562	745															1571
Cj_OXC6468	0400	0464	5732	467	445	3	467	18	445																	0400
Cj_OXC8098	2745	0464	5849	235	138	2	235	138																		2745
Cj_OXC7565	2082	0464	6704	323	324	6	323	641	614	372	723	324														2082
Cj_OXC4844	1154	0574	919	913	785	5	913	949	801	187	785															1154
Cj_OXC5380	0889	0574	2140	732	343	4	732	815	595	343																0889
Cj_OXC7217	1401	0607	607	738	395	3	738	500	395																	1401
Cj_OXC6262	0231	0607	904	235	138	2	235	138																		0231
Cj_OXC6341	0299	0607	904	235	138	2	235	138																		0299
Cj_OXC6359	0316	0607	904	235	138	2	235	138																		0316
Cj_OXC6466	0398	0607	904	235	138	2	235	138																		0398
Cj_OXC7072	1244	0607	904	235	138	2	235	138																		1244
Cj_OXC5789	1594	0607	904	235	138	2	235	138																		1594
Cj_OXC7511	1857	0607	904	235	138	2	235	138																		1857
Cj_OXC7552	2072	0607	904	235	138	2	235	138																		2072
Cj_OXC8017	2638	0607	904	235	138	2	235	138																		2638
Cj_OXC8314	3087	0607	904	235	138	2	235	138																		3087
Cj_OXC8315	3088	0607	904	235	138	2	235	138																		3088
Cj_OXC8935	3717	0607	904	235	138	2	235	138																		3717
Cj_OXC7210	1395	0607	2103	290	554	5	290	272	728	388	554															1395
Cj_OXC7443	1803	0607	2103	290	709	6	290	272	728	388	111	709														1803
Cj_OXC6930	1122	0607	4056	224	216	2	224	216																		1122
Cj_OXC6931	1123	0607	4056	224	216	2	224	216																		1123
Cj_OXC5412	0908	0607	5134	428	77	4	428	468	805	77																0908
Cj_OXC5793	1597	0658	523	890	475	2	890	475																		1597
Cj_OXC7935	2576	0658	657	22	107	2	22	107																		2576
Cj_OXC6436	0376	0658	1044	23	823	3	23	776	823																	0376
Cj_OXC6492	0420	0658	1044	23	823	3	23	776	823																	0420
Cj_OXC6506	0433	0658	1044	23	823	3	23	776	823																	0433
Cj_OXC4737	0701	0658	1044	23	823	3	23	776	823																	0701
Cj_OXC6835	1033	0658	1044	23	823	3	23	776	823																	1033
Cj_OXC4816	1146	0658	1044	23	823	3	23	776	823																	1146
Cj_OXC7277	1298	0658	1044	23	823	3	23	776	823																	1298
Cj_OXC7316	1336	0658	1044	23	823	3	23	776	823																	1336
Cj_OXC7459	1814	0658	1044	23	823	3	23	776	823																	1814
Cj_OXC7462	1816	0658	1044	23	823	3	23	776	823																	1816
Cj_OXC7466	1820	0658	1044	23	823	3	23	776	823																	1820
Cj_OXC4604	1890	0658	1044	23	823	3	23	776	823																	1890
Cj_OXC4903	2047	0658	1044	23	823	3	23	776	823																	2047
Cj_OXC4905	2049	0658	1044	23	823	3	23	776	823																	2049
Cj_OXC7798	2165	0658	1044	23	823	3	23	776	823																	2165
Cj_OXC5179	2322	0658	1044	23	823	3	23	776	823																	2322
Cj_OXC7900	2502	0658	1044	23	823	3	23	776	823																	2502
Cj_OXC8036	2641	0658	1044	23	823	3	23	776	823																	2641
Cj_OXC8157	2835	0658	1044	23	823	3	23	776	823																	2835
Cj_OXC8581	3356	0658	1044	23	823	3	23	776	823																	3356
Cj_OXC8943	3724	0658	1044	23	823	3	23	776	823																	3724
Cj_OXC8946	3726	0658	1044	23	823	3	23	776	823																	3726
Cj_OXC8329	3102	0658	1044	776	823	2	776	823																		3102
Cj_OXC8573	3349	0658	1044	776	823	2	776	823																		3349
Cj_OXC8587	3361	0658	1044	776	823	2	776	823																		3361
Cj_OXC6618	0492	0658	1900	10	613	5	10	817	808	274	613															0492
Cj_OXC7562	2080	0692	692	23	326	4	23	451	149	326																2080
Cj_OXC6777	0637	0692	1301	23	338	4	23	451	338	338																0637
Cj_OXC7456	1812	0692	1301	23	338	3	23	451	338																	1812
Cj_OXC5404	0902	0702	5152	587	414	4	587	62	726	414																0902
Cj_OXC5669	0956	0702	5153	587	414	5	587	781	62	726	414															0956
Cc_OXC8728	C454	0828	827	689	893	8	689	655	321	461	190	23	714	893												C454
Cc_OXC5257	C297	0828	854	515	1015	5	515	867	821	344	666															C297
Cc_OXC7612	C277	0828	854	821	1012	9	821	412	337	335	150	788	132	131	275											C277
Cc_Dg46b	C379	0828	872	889	553	7	889	239	113	830	947	303	553													C379
Cc_OXC6094	C459	0828	1096	401	601	2	401	601																		C459
Cc_OXC6725	C119	0828	1096	985	322	3	985	986	322																	C119
Cc_Dg319	C393	0828	1127	373	948	16	373	307	437	437	117	470	84	879	918	823	167	918	823	167	255	948				C393
Cc_Dg100a	C388	0828	1464	889	125	8	889	239	695	830	947	303	469	125												C388
Cc_OXC6559	C065	0828	5757	829	461	19	829	535	662	679	719	821	826	63	751	64	266	229	847	296	174	242	122	773	461	C065
Cc_OXC6744	C122	0828	5813	816	504	4	816	115	94	504																C122
Cc_K3	C040	0828	7123	380	736	7	380	536	213	354	617	684	736													C040
Cj_OXC6431	0372	1034	1709	23	338	3	23	451	338																	0372
Cj_OXC6498	0426	1034	1709	23	338	3	23	451	338																	0426
Cj_OXC6593	1032	1034	1709	23	338	3	23	451	338																	1032
Cj_OXC7192	1379	1034	5763	22	107	6	22	487	938	579	781	107														1379
Cj_OXC7893_R	3138	1034	5763	22	107	6	22	487	938	579	781	107														3138
Cj_OXC6811	0669	1034	5817	23	338	3	23	451	338																	0669
Cj_OXC7299	1320	1034	6235	23	338	3	23	451	338																	1320
Cj_OXC8060	2645	1287	945	757	757	3	757	320	757																	2645
Cj_OXC5407	0905	1332	5154	23	77	4	23	451	149	77																0905
Cj_Dg195	1972	1332	6563	451	717	3	451	61	717																	1972
Cj_Dg368	2923	1332	6563	451	717	3	451	61	717																	2923

Table S8. Distribution of CRISPR spacer alleles in *C. jejuni* MLST-clonal complexes

Spacer [a]	n [b]	Primary clonal complex [c,d,e]	Secondary clonal complexes [c,d]
2	38	ST-21 (n=30)	other jejuni (n=8)
7	204	ST-45 (n=120)	ST-283 (n=40), other jejuni (n=21)
9	32	ST-22 (n=21)	
11	96	**ST-45** (n=94)	
12	82	**ST-45** (n=82)	
14	148	ST-45 (n=87)	ST-283 (n=47)
19	177	**ST-21** (n=173)	
20	97	ST-283 (n=46)	ST-45 (n=34), ST-48 (n=17)
21	105	ST-45 (n=56)	ST-283 (n=32), ST-48 (n=16)
22	295	ST-257 (n=208)	ST-48 (n=61)
23	115	ST-21 (n=64)	other jejuni (n=46)
25	78	**ST-45** (n=77)	
29	86	ST-48 (n=74)	other jejuni (n=11)
33	30	ST-21 (n=17)	ST-45 (n=13)
48	57	**ST-206** (n=55)	
57	64	ST-21 (n=51)	ST-206 (n=11)
79	52	ST-48 (n=31)	ST-45 (n=20)
103	168	**ST-257** (n=167)	
107	281	other jejuni (n=257)	
111	43	ST-22 (n=16)	ST-45 (n=13)
158	39	ST-257 (n=34)	
172	109	ST-61 (n=69)	ST-21 (n=31)
183	61	ST-443 (n=54)	
201	64	**ST-257** (n=63)	
216	118	ST-48 (n=68)	ST-21 (n=41)
218	37	ST-21 (n=33)	
234	39	ST-257 (n=34)	
235	56	ST-21 (n=30)	other jejuni (n=23)
260	54	**ST-206** (n=54)	
261	93	ST-21 (n=83)	other jejuni (n=10)
274	200	ST-257 (n=146)	ST-21 (n=44)
284	57	**ST-21** (n=55)	
289	116	ST-61 (n=72)	ST-21 (n=34)
323	97	**ST-21** (n=93)	
338	102	ST-21 (n=83)	other jejuni (n=19)
383	182	**ST-257** (n=178)	
451	86	ST-21 (n=63)	other jejuni (n=22)
457	64	**ST-257** (n=63)	
487	487	ST-21 (n=434)	other jejuni (n=50)
541	94	**ST-21** (n=88)	
566	33	**ST-283** (n=32)	
569	31	**ST-48** (n=31)	
585	51	ST-48 (n=28)	ST-45 (n=21)
597	178	**ST-21** (n=176)	
625	39	ST-21 (n=33)	
641	112	**ST-21** (n=110)	
649	165	**ST-21** (n=164)	
655	55	**ST-206** (n=54)	
671	41	**ST-206** (n=41)	
677	57	**ST-21** (n=55)	
689	59	**ST-206** (n=54)	
690	90	**ST-206** (n=81)	
692	73	**ST-48** (n=73)	
703	171	ST-354 (n=114)	ST-443 (n=55)
712	91	ST-21 (n=79)	other jejuni (n=12)

715	104	**ST-354** (n=104)	
728	44	ST-21 (n=37)	
735	46	**ST-21** (n=45)	
741	152	**ST-257** (n=151)	
743	513	**ST-21** (n=486)	
759	72	ST-257 (n=52)	ST-21 (n=19)
773	75	ST-21 (n=43)	ST-22 (n=25)
775	180	**ST-21** (n=171)	
781	79	other jejuni (n=45)	ST-21 (n=32)
799	170	ST-354 (n=114)	ST-443 (n=54)
818	50	other jejuni (n=40)	
820	44	ST-257 (n=44)	
823	118	other jejuni (n=41)	ST-283 (n=40), ST-48 (n=30)
860	221	**ST-21** (n=216)	
874	216	**ST-21** (n=206)	
889	127	ST-354 (n=105)	ST-22 (n=17)
938	341	**ST-21** (n=323)	

a. Spacer alleles are extended from Kovanen et al, 2014, and given in Table S5. Only spacers detected in ≥ 30 genomes are shown here.

b. Represents the number of *C. jejuni* and *C. coli* genomes (from 4,232 total) in which the sequence of the spacer allele was detected in combination with a 5'-TGGTAAAAT and 3'-GTTTT linker.

c. Clonal complexes based on the definition file available from http://pubmlst.org/campylobacter/. The category "other jejuni" combines all other clonal complexes given in Table S7.

d. Primary clonal complex represents the clonal complex in which the majority of spacer alleles was detected, secondary clonal complexes represent those in which a proportion of >10% of the spacer total was found if n ≥ 10 .

e. Clonal complexes shown in red represent contain ≥ 90% of the detected spacers.
